# Synthesis of Indoline- and 1,2,3,4-Tetrahydroquinoline-Based Symmetrical Triarylmethanes

**DOI:** 10.1021/acs.joc.5c00960

**Published:** 2025-07-07

**Authors:** Yunus Taskesenligil, Murat Aslan, Rabia Ardahanli, Nurullah Saracoglu

**Affiliations:** † Department of Chemistry, 37504 Faculty of Sciences, Atatürk University, Erzurum 25240, Türkiye; ‡ Biotechnology Institute, Ankara University, Ankara 06135, Türkiye

## Abstract

We present a novel and efficient, metal-free methodology for the regioselective synthesis of symmetrical triarylmethanes through C–H alkylation of indolines and 1,2,3,4-tetrahydroquinolines with aryl aldehydes. The transformation employs 1,1,1,3,3,3-hexafluoro-2-propanol (HFIP) as both solvent and promoter, eliminating the need for traditional metal catalysts or directing groups. This method demonstrates exceptional regioselectivity, targeting the C5 position of indolines and the C6 position of tetrahydroquinolines exclusively. The protocol exhibits a broad substrate scope, accommodating diverse aryl aldehydes and heterocyclic substrates under mild conditions. Notably, the reaction proceeds efficiently with both N–H-free and *N*-benzyl-protected substrates. Mechanistic studies, based on control experiments and literature findings, indicate that the reaction proceeds via iminium intermediates and regioselective C–H functionalization. The practical utility of this methodology is demonstrated through gram-scale synthesis, high HFIP recyclability, and successful late-stage functionalization of complex bioactive molecules. This approach represents a significant advancement in the sustainable synthesis of symmetrical triarylmethanes, offering a valuable tool for applications in pharmaceutical and materials chemistry.

## Graphical abstract

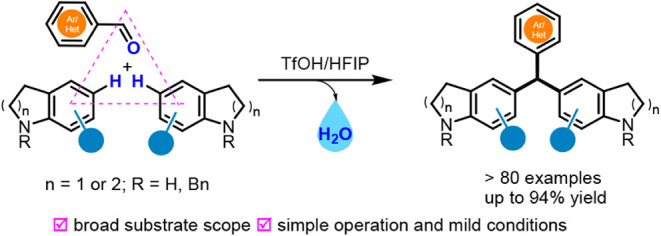

## Introduction

The aniline core is a fundamental building block in bioactive molecules and donor–acceptor compounds, widely used in pharmaceuticals, agrochemicals, and materials science.^1^ Indoline and 1,2,3,4-tetrahydroquinoline (THQ) are aniline derivatives containing a five- or six-membered nitrogen heterocycle fused to a benzene ring. These structures are commonly found in natural products, pharmaceuticals, and functional materials.^2^ Triarylmethanes (TRAMs) and their derivatives play a crucial role in chemical sciences and are particularly significant in synthetic organic chemistry due to their unique molecular structures and physical properties. These scaffolds are found in natural products, medicinal agents, and materials chemistry building blocks (Figure 1). TRAMs have been widely used as ligand scaffolds, as well as in the formulation of many dyes, fluorescent probes, red light-absorbing photoredox catalysts, and red-light fluorescent OLEDs, making them very important synthetic targets.^3^ Within this family, aniline-based TRAMs and their heterocyclic variants are of growing importance because of their unique properties.^4^ For example, rhodamine-based fluorescent dyes, are a subset and oxidation product of the triarylmethane dyes and have been extensively used in biotechnology for bioimaging and therapeutics since their inception (Figure 1).^4^

**Figure 1:**
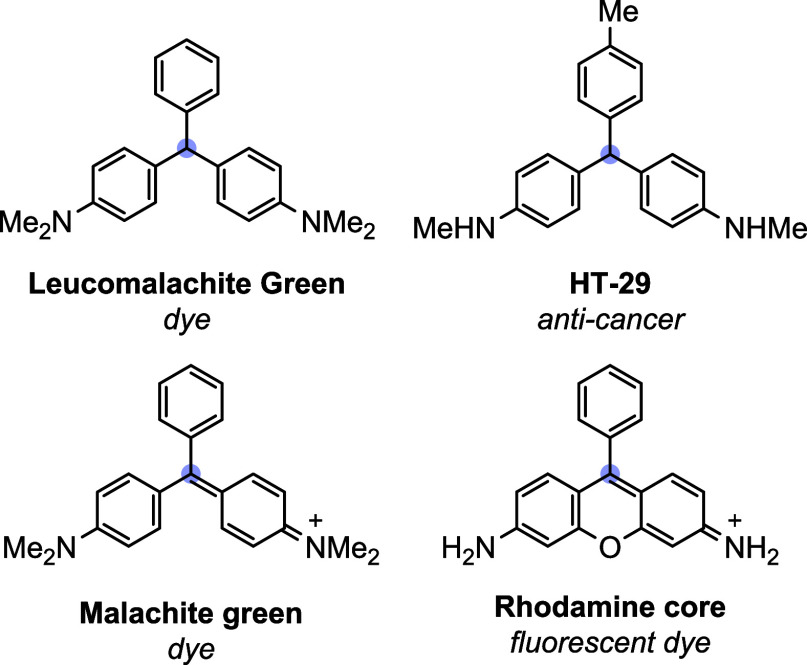
Selected aniline-based triarylmethane motifs.

Furthermore, modified rhodamine dyes with different absorption and emission wavelengths were developed by replacing the central oxygen atom with other elements such as S, P, Ge, Sn, and Te.^5^ Many methods are available to prepare the precursors of aniline-based TRAMs for rhodamines. Hence, developing synthetic strategies for these compounds is highly desirable. Conventionally, the reported routes to aniline-based TRAMs via aryl-aldehydes use catalysts that are commonly based on metals,^6^ Brønsted acids such as sulfonic acid, sulfuric acid, and HCl, or Lewis acids such as zeolite and montmorillonite K-10.^7^ As such, these routes result in significant issues in their application, including the presence of metal-containing residues and the use of corrosive acids or hazardous reagents. To the best of our knowledge, triarylmethane synthesis from indolines and THQs via aryl aldehydes has not been reported.

In 2017, the Ogawa group reported a convenient, novel, and metal-free method for the synthesis of aniline-based TRAMs.^8^ In context, tetrahydroquinoline-based TRAM as a single example (in a yield of 38%) was prepared via one-pot condensation of benzylamine with julolidine using 4,6-dihydroxysalicylic acid as a co-oxidant and *N*-iodosuccinimide (NIS) as an oxidant (Scheme 1a). Independently, Pan and Seidel and their co-workers conducted the reactions of aryl-aldehydes and indolines using benzoic acid as the catalyst and carried out the reaction under reflux or microwave irradiation conditions, to give *N*-benzylindoles via redox isomerization (Scheme 1b).^9^ When salicylaldehydes were employed as the substrate, both groups found that *N*-benzylindoline products occurred in good yields through intermolecular hydride transfer (Scheme 1b). In this scenario, another molecule of indoline serves as the hydride donor, leading to the formation of indole.^9^

**Scheme 1:**
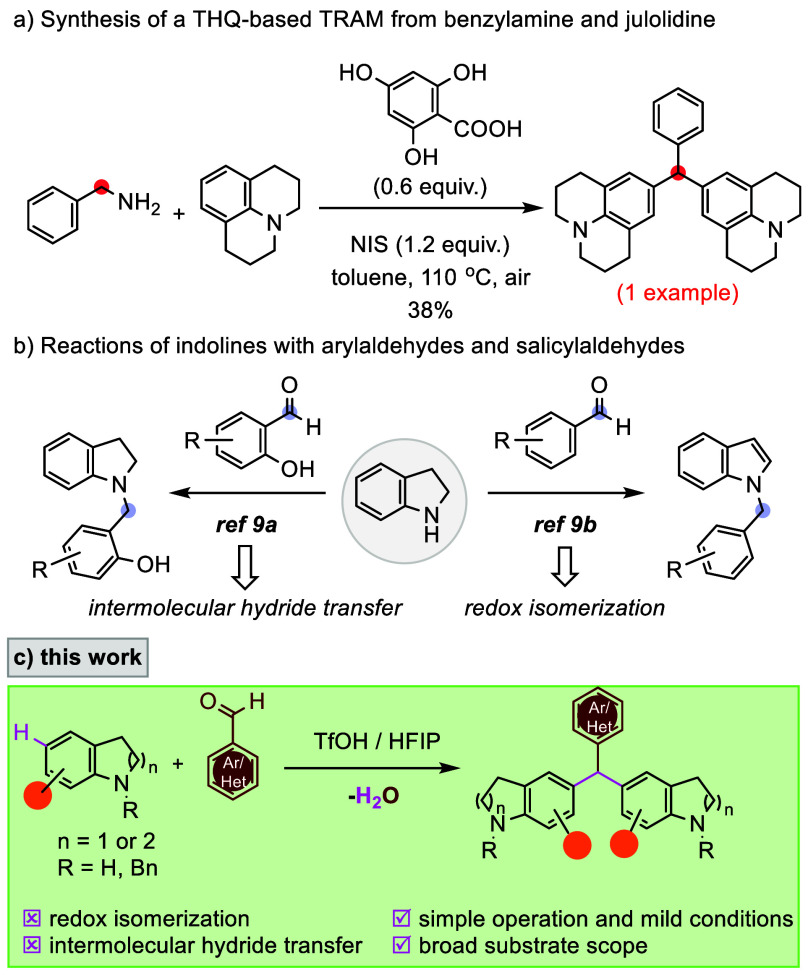
Overview of the Work

For the past decade, 1,1,1,3,3,3-hexafluoro-2-propanol (HFIP) has been a unique solvent that has been reported to frequently outperform traditional solvents such as toluene, dichloromethane, and nitromethane in carrying out a wide range of challenging chemical reactions.^10^ It possesses unique physical and chemical properties, such as stabilizing cationic and radical species, strong hydrogen bond donation, low nucleophilicity, and remaining inertness under redox stress. However, the combination of HFIP with Lewis acids and Brønsted acids effectively supports many reactions such as cyclizations,^11^ hydroarylation reactions,^12^ hydrofunctionalization reactions,^13^ and especially in the Friedel–Crafts reactions.^14^ Recently, we have documented regioselective bond alkylation reactions promoted by HFIP and controlled of *para*-quinone methides (*p*-QMs) with N–H-free indoline and THQ under metal-free conditions and an electrophilic substitution reaction between 4,7-dihydroindole and aryl-aldehydes, which, after an oxidation step, produces 2,2′-bis­(indolyl)­arylmethanes.^15^ HFIP played a crucial role in controlling alkylation selectivity due to its ability to donate hydrogen-bond solvents. As a continuation of our interest in C–H bond functionalization, we report catalyst- and solvent-controlled-mediated and controlled Friedel–Crafts-type regioselective C–H alkylations of indolines and THQs with aryl aldehydes in a wide scope as electrophile sources. This methodology is remarkable for its ability to construct symmetrical triarylmethane scaffolds at the C5 and C6 positions respectively, via the intermolecular dehydrative Friedel–Crafts reaction (or Baeyer condensation) between aryl-aldehydes with indolines and THQs, with N–H free and N-Bn without the need for any directing group at the N-center (Scheme 1c).

## Results and Discussion

The starting indoline and THQ derivatives (1 and 5) required for the present study were prepared with the reduction of indole and quinoline derivatives using NaCNBH_3_ in AcOH.^16^ To establish the optimal reaction conditions for C–H alkylation of indolines and THQs to achieve symmetrical triarylmethanes, we commenced with the screening of the reaction between indoline (**1a**) and 4-bromobenzaldehyde (**2f**) as model substrates (Table 1). Initially, the 2.1 equiv of indoline (**1a**) was tested with aryl aldehyde **2f** in HFIP without a catalyst for 5 h (entry 1). This screening did not produce the desired product **3af**, rather yielding the aminalization product **4af**, which could be characterized by ^1^H NMR spectroscopy. However, **4af** was not isolated in its aminal structure via the silica gel column due to fragmentation during the purification process, despite its high reaction conversion, resulting in the recovery of the starting materials. The reaction was then retested in HFIP by increasing the equivalents (from 2.1 equiv to 4 equiv; and 6 equiv) of indoline (**1a**) (entries 2 and 3). When the reaction was carried out with 6 equiv of **1a** for 24 h, the desired TRAM product **3af** was isolated in an 85% yield (entry 3). Alcohols used instead of HFIP (entries 4–6) provided product **4af** (entries 4–6). Additionally, the effect of Lewis (Sc­(OTf)_3_, Sn­(OTf)_2_, Zn­(OTf)_2_, Yb­(OTf)_3_, In­(OTf)_3_, B­(C_6_F_5_)_3_, BF_3_·OEt_2_, FeCl_3_) or Brønsted acids (TFA, TfOH) on the course of the reaction was investigated, and the obtained results revealed that catalyst screening did not improve the yields at room temperature in HFIP for 24 h (entries 7–16). Since the yield (**3af**; 70%) of the reaction using triflic acid (TfOH) as a catalyst (entry 13) was higher than the others, we also performed the reaction in different solvents such as DCE, DCM, dioxane, toluene, MeCN, and THF instead of HFIP. We observed that while the reaction occurred, the yield of **3af** was lower in these solvents (entries 17–22). Hence, we chose the reaction using HFIP as a solvent or promoter without any catalyst at room temperature with 6 equiv of **1a** as the optimal reaction conditions for the transformation (entry 3).

**Table 1:**
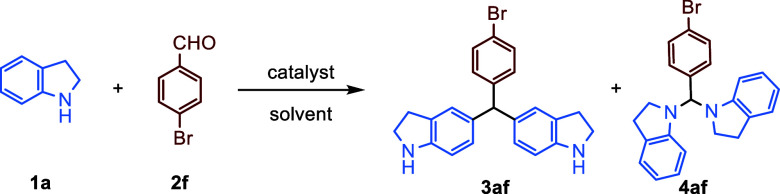
Optimization of the Reaction Conditions^a^

entry	catalyst (10 mol %)	solvent	**1a** (equiv)	time (h)	**3af** (yield %)^b^	**4af** (yield %)^c^
1	-	HFIP	2.1	5	-	93
2	-	HFIP	4	24	60	-
3	-	HFIP	6	24	85	-
4	-	MeOH	6	24	-	85
5	-	EtOH	6	24	-	88
6	-	*i*PrOH	6	24	-	91
7	Sc(OTf)_3_	HFIP	6	24	57	-
8	Sn(OTf)_2_	HFIP	6	24	54	-
9	Zn(OTf)_2_	HFIP	6	24	63	-
10	Yb(OTf)_3_	HFIP	6	24	45	-
11	In(OTf)_3_	HFIP	6	24	60	-
12	BF_3_·OEt_2_	HFIP	6	24	62	-
13	FeCl_3_	HFIP	6	24	58	-
14	B(C_6_F_5_)_3_	HFIP	6	24	54	-
15	TFA	HFIP	6	24	68	-
16	TfOH	HFIP	6	24	70	-
17	TfOH	DCE	6	24	69	-
18	TfOH	DCM	6	24	58	-
19	TfOH	dioxane	6	24	61	-
20	TfOH	toluene	6	24	48	-
21	TfOH	MeCN	6	24	46	-
22	TfOH	THF	6	24	45	-

a Reaction conditions: **1a** (1 mmol), catalyst (10 mol %), **2f** (0.125 mmol), solvent (1 mL), room temperature.

b Isolated yield.

c NMR yield. TFA: Trifluoroacetic acid. TfOH: Trifluoromethanesulfonic acid. DCE = Dichloroethane. DCM = Dichloromethane. THF = Tetrahydrofuran.

With the optimized reaction conditions established, we continued investigating the scope of the transformation for different aryl/heteroarylaldehydes **2a–y** (Scheme 2a–c). First, we focused on the reactivity of indoline (**1a**) with various *para*-, *meta*-, and *ortho*-substituted benzaldehyde derivatives (**2a–q**) (Scheme 2a). Generally, *para*-substituted benzaldehydes, except 4-hydroxybenzaldehyde (**2i**), delivered the corresponding triarylmethane products **3a–h** in moderate to high yields (71–88%) (Scheme 2a). However, benzaldehydes (**2j–l**) including electron-drawing groups such as NO_2_, CF_3_, and CO_2_Me needed 2.5 equiv of indoline (**1a**) to deliver better conversion. It was found that *meta*-substituted benzaldehydes **2m–o** also worked for the alkylation of indoline (**1a**), with good reaction yields (**3m–o**; 69–83%). The ortho-methyl substituted benzaldehyde (**2p**) is also compatible with the reaction conditions to afford product **3ap** in 73% yield. Pleasingly, both aldehyde groups of terephthaldehyde (**2q**), using 12 equiv of **1a**, reacted to give a bis-triarylmethane derivative (**3aq**, 80%). Polycyclic aromatic aldehydes such as 2-naphthaldehyde (**2r**), 9-anthracenecarboxaldehyde (**2s**), and pyrene-1-carbaldehyde (**2t**) did not give the desired triarylmethane products (**6ar–at**) under standard conditions. Interestingly, 2-naphthaldehyde (**2r**) proved to be a feasible substrate for the production of the corresponding product 3ar (79% yield) in the presence of TfOH (10 mol %) catalyst in HFIP. The lack of reactivity observed with 9-anthracenecarboxaldehyde (**2s**), and pyrene-1-carbaldehyde (**2t**) in the present study is interpreted as due to the steric hindrance of the bulky anthracene and pyrene rings (Scheme 2a). Also, various heteroaromatic aldehydes (**2u–y**) containing thiophene, pyrrole, furan, pyridine, and indole rings were examined to determine the influence of different heteroaromatic rings on the generality of this reaction (Scheme 2b). Thiophene-2-carboxaldehyde (**2v**_**2**_) delivered the corresponding product **3av**_**2**_ in 76% yield under optimized reaction conditions. Furthermore, the reaction was carried out at room temperature using TfOH (10 mol %) as the catalyst and furan-2-carboxaldehyde (**2x**_**2**_), which exhibited a behavior distinct from other aldehydes, affording the Piancatelli rearrangement product **8** in 85% yield.^17^ Notably, for the pyrrole-2-carboxaldehyde (**2w**_**2**_) and indole-aldehyde derivatives (**5y**_**2–7**_), the relevant products could not be observed. Most of the starting materials were recovered. This lack of reactivity could be attributed to the increased electron-density in the aldehyde groups possibly due to tautomeric equilibrium or mesomeric structures. We envisioned that protecting the NH group with an electron-withdrawing group would increase the reactivity of the aldehyde group, and we prepared the tosyl-protected pyrrole and indole derivatives. Unfortunately, the reaction between **1a** and these aldehydes did not deliver the corresponding products even after a prolonged reaction time. Using the triflic acid (TfOH) catalyst in HFIP yielded the desired results, affording the products **3ay**_**3–6**_ in a 74–85% yield. Despite using both optimized reaction conditions and conditions with a TfOH catalyst, no product formation was observed when attempting to react 1-tosyl-1*H*-pyrrole-2-carbaldehyde (**2aw**_**2**_) and 1-tosyl-1*H*-indole-7-carbaldehyde (**2ay**_**7**_). It can be concluded that the chemical steric hindrance of the *N*-tosyl groups in the pyrrole and indole rings is effective in these reactions. In addition, product formation was observed for 1-tosyl-1*H*-indole-2-carboxaldehyde (**2y**_**2**_), but the corresponding product **3ay**_**2**_ could not be purified. When 2.5 equiv of **1a** were used, pyridine aldehydes **2u**_**2–4**_ proved compatible with our methodology, affording the corresponding triarylmethane products (**3au**_**2–4**_) high isolated yields (81–90%) (Scheme 2b).

**Scheme 2:**
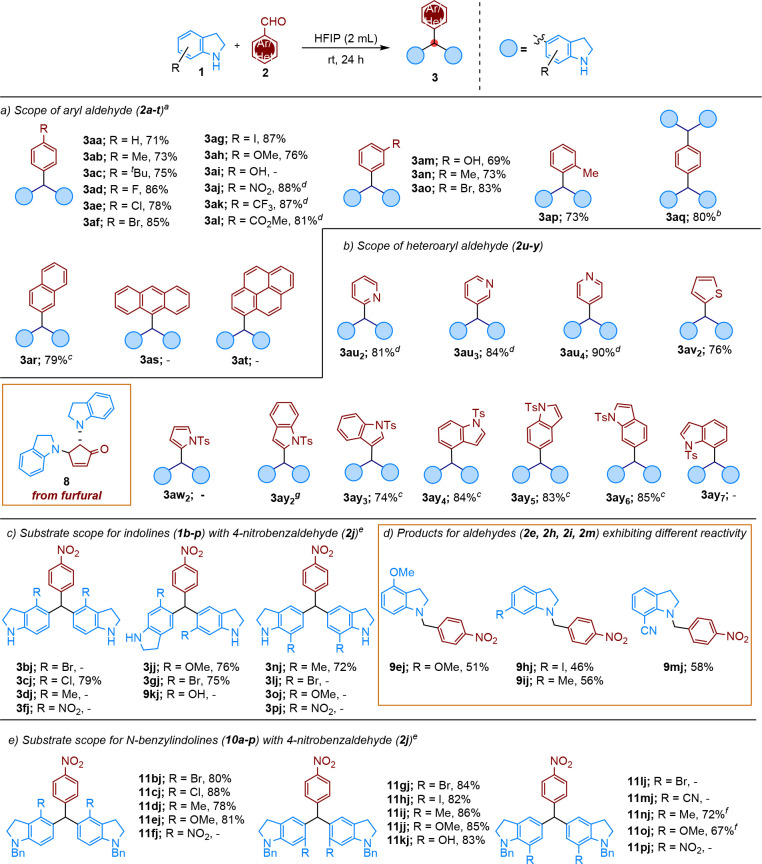
Substrate Scope of Indolines (**1a–p** and **10a–p**) and Aryl/Heteroaryl Aldehydes (**2a–y**)

a Reaction Conditions: **1a** (1.5 mmol, 6.0 equiv), aldehyde (0.25 mmol, 1 equiv).
b
**1a** (3 mmol, 12 equiv), **2q** (0.25 mmol, 1 equiv).
c
**1a** (1.5 mmol, 6 equiv), aldehyde (0.25 mmol, 1 equiv), TfOH (10 mol %).
d
**1a** (0.625 mmol, 2.5 equiv), aldehyde (0.25 mmol, 1 equiv).
e
**10a**–**p** (1.5 mmol, 2.5 equiv), aldehyde (0.60 mmol, 1 equiv), TfOH (10 mol %).
f 80 °C, sealed tube.
g Could not be purified.

Subsequently, the scope of the reaction with a series of C-4, C-6, and C-7 substituted indolines **1b–m** and 4-nitrobenzaldehyde (**2j**) was investigated under the optimized reaction conditions using TfOH (10 mol %) catalyst (Scheme 2c). The selection of 4-nitrobenzaldehyde as the aldehyde derivative was due to its higher reactivity when compared to other options. As shown in Scheme 2c, the substituents on the C-7 position of indoline significantly affect the reaction. Notably, the majority of the reactions were almost nonexistent. The reaction occurred only in the presence of the Me group (**1n**), which was an exception. The decrease in reactivity may be attributed to steric crowding caused by the substituent of the C-7 position. It is noteworthy that only indoline-7-carbonitrile (**1m**) afforded an unexpected *N*-benzylindole derivative **9mj**, with a 58% yield, via reductive alkylation. The result indicates that the presence of the nitrile group at the C-7 does not support alkylation at the C-5 position. The substituents at the C-4 position of indoline **1c** produced the desired product, **3cj**, in 79% yield. However, when substrates **1b** and **1d–f** with Br, Me, and NO_2_ groups at the C-4 position were employed, they failed to give the target product. Additionally, we investigated the reactivity of C-6 substituted indoline substrates. While substrates **1j** and **1k** yielded the target products (**3jj** and **3gj**), 6-hydroxyindoline (**1k**) proved unreactive. Similarly, substrates **1h**–**i** bearing I and Me substituents at C-6 were unsuitable for this transformation. Instead, they afforded unexpected reductive alkylation products (**9hj** and **9ij**) in 46% and 56% yields, respectively (Scheme 2d). To further investigate the influence of the free amine group of the indoline on reductive alkylation, we prepared the benzyl-protected indolines **9a–p** (Scheme 2e). The most reactions between **9a**–**p** and 4-nitrobenzaldehyde (**2j**) delivered products **3aj–pj**, except for four derivatives (nitro-substituted substrates **9f**, **9p**, C4-bromo, and nitrile substrates **9l**, and **9m**). It is noticeable that substrates including electron-withdrawing groups at the C-4 position (NO_2_) and at the C-7 position (NO_2_, Br, CN), failed to give the target products. Methyl and methoxy substituents at the C-7 position of indoline afforded the expected products **9nj** and **9oj** in 72% and 67% yields respectively, at 80 °C in a sealed tube.

Encouraged by the above results, this approach was extended to 2,3,4,5-tetrahydroquinoline substrates under the optimized reaction conditions to explore the scope and limitation of this transformation. Notably, 2,3,4,5-tetrahydroquinoline (**5a**) was subjected to a reaction with **2f** under the determined optimized reaction conditions for indolines, and the desired product 6af was obtained in lower yield of 68% (Scheme 3a, Table 2, entry 4). Therefore, we focused on optimizing the reaction between **5a** and **2f** (Table 2). When the reaction time and the equivalent of THQ were also changed, yields did not improve (Table 2, entries 1–3). No product formation was observed when MeOH and EtOH were used as alcohol solvents instead of HFIP (Table 2, entries 4 and 5). Furthermore, various Lewis and Brønsted acids were also screened (Table 2, entries 7–16). Screening using TfOH as a catalyst resulted in a significant increase in the yield of the product from 69% to 89% (Table 2, entry 16). Reactions were carried out using different solvents (DCE, DCM, dioxane, toluene, MeCN, and THF) instead of HFIP to investigate the effect of the solvent (Table 2, entries 17–23). However, none of these solvents proved to be more than HFIP.

**Scheme 3:**
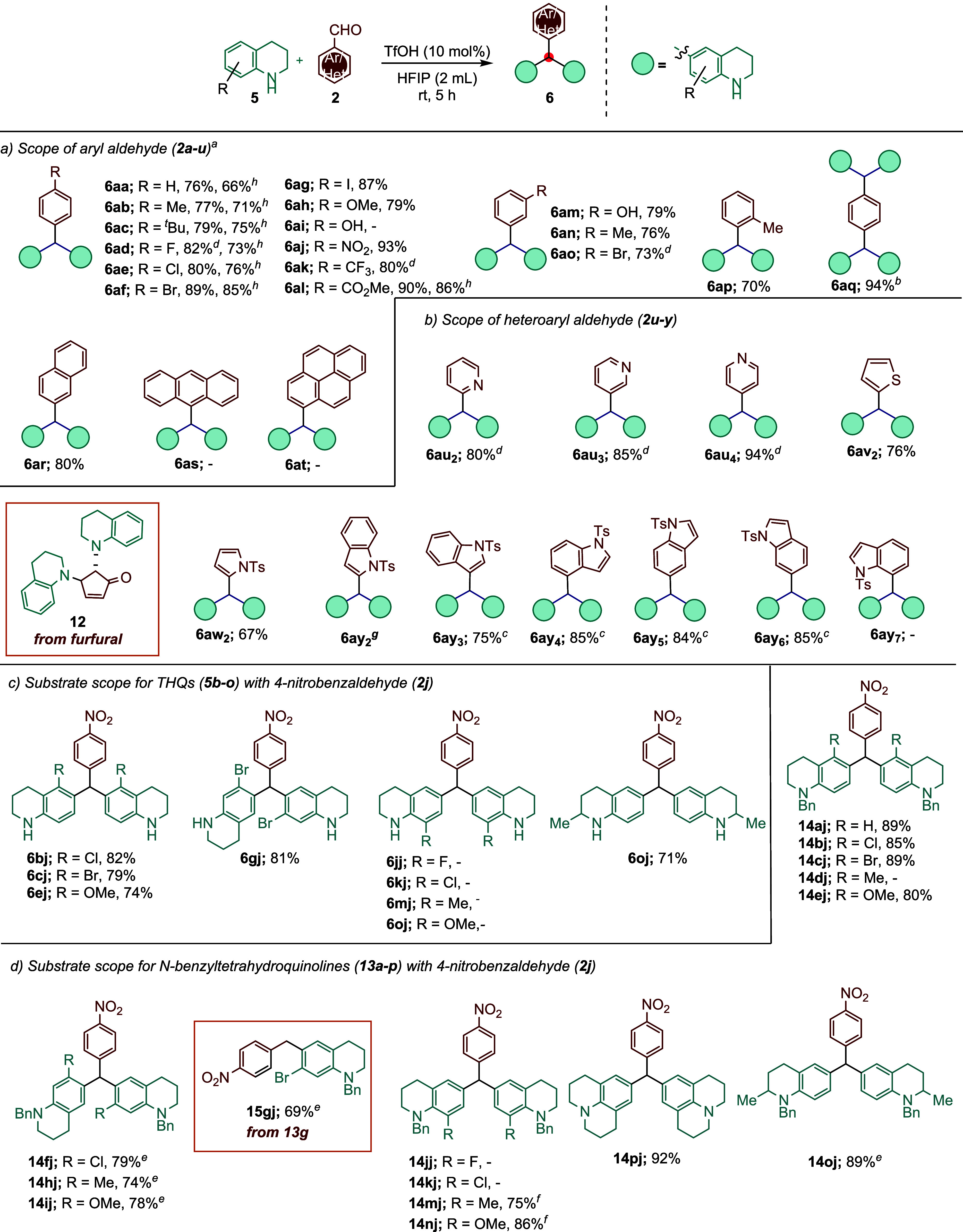
Substrate Scope of THQs (**5a–p** and **13a–p**) and Aryl/Heteroaryl Aldehydes (**2a–y**)

a Reaction Conditions: **5a** (1 mmol, 2.5 equiv), aldehyde (0.40 mmol, 1 equiv), 5 h.
b
**5a** (3 mmol, 5 equiv), **2q** (0.25 mmol, 1 equiv), 24 h.
c
**5a** (1.5 mmol, 6 equiv), aldehyde (0.25 mmol, 1 equiv), 56 h.
d
**5a** (1 mmol, 2.5 equiv), aldehyde (0.40 mmol, 1 equiv), 7 h.
e In a sealed tube at 90 °C.
f In a sealed tube at 120 °C.

g
Could not be purified.
h THQ (1 mmol, 2.5 equiv), aldehyde (0.40 mmol, 1 equiv), 5 h in DCE.

**Table 2:**
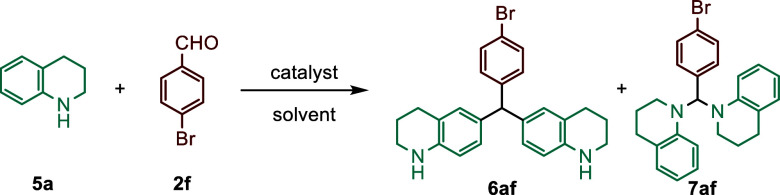
Optimization of the Reaction Conditions^a^

entry	catalyst (10 mol %)	solvent	**5a** (equiv)	time (h)	**6af** (yield %)^b^	**7af** (yield %)^c^
1	-	HFIP	2.1	5	20	-
2	-	HFIP	2.5	5	23	-
3	-	HFIP	4	24	58	-
4	-	HFIP	6	24	68	-
5	-	MeOH	2.5	24	-	-
6	-	EtOH	2.5	24	-	-
7	Sc(OTf)_3_	HFIP	2.5	5	63	-
8	Sn(OTf)_2_	HFIP	2.5	5	65	-
9	Zn(OTf)_2_	HFIP	2.5	5	65	-
10	Yb(OTf)_3_	HFIP	2.5	5	64	-
11	In(OTf)_3_	HFIP	2.5	5	68	-
12	BF_3_·OEt_2_	HFIP	2.5	5	70	-
13	FeCl_3_	HFIP	2.5	5	66	-
14	B(C_6_F_5_)_3_	HFIP	2.5	5	70	-
15	TFA	HFIP	2.5	5	78	-
16	TfOH	HFIP	2.5	5	89	-
17	TfOH	DCE	2.5	5	85	-
18	TfOH	DCM	2.5	5	72	-
19	TfOH	dioxane	2.5	5	79	-
20	TfOH	toluene	2.5	5	71	-
21	TfOH	MeCN	2.5	5	82	-
22	TfOH	THF	2.5	5	80	-
23	-	DCE	2.1	24	-	16

a Reaction conditions: **5a** (0.5 mmol), catalyst (10 mol %), **2f** (0.2 mmol), solvent (1 mL), room temperature.

b Isolated yield.

c NMR yield.

When the reaction between 2.1 equiv of **5a** and 1 equiv of **2f** was carried out in dichloroethane (DCE) without a catalyst, the formation of the aminal **7af** was determined by ^1^H NMR spectroscopy (Table 2, entry 21). Hence, the reactions were performed using 2.5 equiv of 1,2,3,4-tetrahydroquinoline in HFIP with 10 mol % TfOH catalyst (Table 2, entry 14). After the reaction conditions for tetrahydroquinoline (**5a**) were optimized, reactions with other aldehyde derivatives were also performed (Scheme 3a,b). Since the reactions between **5a** and all other aldehydes (**2a–y**) exhibited trends similar to those observed with indoline, they are not discussed in detail here (see Scheme 3a,b). As seen in Scheme 3a,b, higher yields were achieved in reactions with certain aldehydes by adjusting the tetrahydroquinoline equivalent and reaction time. Distinctly, the reaction between *N*-tosylpyrrole-2-carbaldehyde (**2w**_**2**_) and tetrahydroquinoline (**5a**) afforded the corresponding product **6aw**_**2**_ with a yield of 67% (Scheme 3a). Although product formation was observed in the reaction between indole-2-carbaldehyde (**2y**_**2**_) and tetrahydroquinoline (**5a**), the product could not be purified (Scheme 3b). The corresponding products **6aa-6af** and **6al** were obtained using DCE as solvent, albeit with slightly lower yields compared to reactions conducted in HFIP (Scheme 3a).

Additionally, furfural (**2x**_**2**_) exhibited a behavior different from other aldehydes and, similar to indoline, provided the Piancatelli rearrangement product **12** (Scheme 3b).^17^ To investigate the effect of substituents on the reaction, the reactions of tetrahydroquinoline derivatives containing substituents at the 5-, 7-, and 8-positions with *para*-nitrobenzaldehyde (**2j**) were studied (Scheme 3c). Similar to the behavior observed in indoline derivatives, no product formation was detected for 8-substituted tetrahydroquinoline derivatives with the substituent in the ortho-position relative to the nitrogen atom. In contrast, the expected products (**6bj–6ej**, **6gj**, **6oj**) were obtained from tetrahydroquinoline derivatives containing 5-chloro, 5-bromo, 5-methoxy, and 7-bromo substituents, as well as, 2-methyl-tetrahydroquinoline compounds (Scheme 3c). However, reactions with other substituents at the 5-position (Me) and 7-position (Cl, Me, OMe) did not yield positive results. Specifically, the presence of substituents at the 8-position of tetrahydroquinoline also leads to the failure of the reaction. This failure is attributed to the steric effects of these groups, which hinder the formation of possible products or the addition of a second tetrahydroquinoline to these intermediates (Scheme 3c). The presence of electron-donating groups at the 5-, 7-, and 8-positions of the benzene ring in *N*-benzyltetrahydroquinoline derivatives has been shown to greatly tolerate the reaction and allow it to proceed smoothly (Scheme 3d). Similar results were observed with *N*-benzylindolines and were also obtained for *N*-benzyltetrahydroquinoline derivatives (Scheme 3d). However, for 2-methyl-*N*-benzyl-tetrahydroquinoline (**13o**) and some *N*-benzyl-tetrahydroquinoline derivatives (**13f–i**, **13m–n**) containing substituents at the 7- and 8-positions, the reactions were carried out in sealed tubes at 90 and 120 °C, because of incomplete reactions (Scheme 3d).

To demonstrate the synthetic potential of the developed protocol and evaluate its applicability on a gram scale, it is necessary to investigate the recycling and reuse potential of the high-cost HFIP reagent has arisen (Scheme 4a). For this purpose, a reaction was carried out using 1.0 g (5.40 mmol) of 4-bromobenzaldehyde (**2f**) and 32.4 mmol of indoline (**1a**), and the final product **3af** was obtained with a 74% yield. During the reaction, 34 mL of the 40 mL HFIP used was recovered. Similarly, in the gram-scale reaction between 4-bromobenzaldehyde (**2f**) and tetrahydroquinoline (5a), the product 6af was obtained with a 79% yield. In addition, 32 mL of the 40 mL HFIP used was recovered. A decrease of 10–11% in chemical yields was observed during the gram-scale studies, compared to 1.0 mmol-scale reactions. This decrease is thought to be due to difficulties in reaction kinetics, mixture homogeneity, and heat transfer associated with scale-up. Nevertheless, the substantial recovery of HFIP and the good yields of the products are satisfactory. Additionally, the potential for the conversion of indoline and tetrahydroquinoline derivatives **3af** and 6af to the indole and quinoline derivatives was investigated (Scheme 4a). While **3af** was converted to the indole derivative **16af** with a 90% yield in the presence of MnO_2_ at room temperature in methylene chloride, the compound 6af was also converted under the same conditions to the quinoline derivative **17af**, with an 80% yield. The promising results outlined above demonstrate the broad applicability and high efficiency of the methodology for the synthesis of TRAMs. These attributes render the protocol a valuable tool for the precise late-stage modification of complex biologically relevant scaffolds (Scheme 4b). Notably, *DL*-tryptophan, cholesterol, and *L*-menthol were efficiently converted into late-stage modified products (**18–20**; 71–74%) through the developed methodology using tetrahydroquinoline. This finding underscores the synthetic advantages of the present approach.

**Scheme 4:**
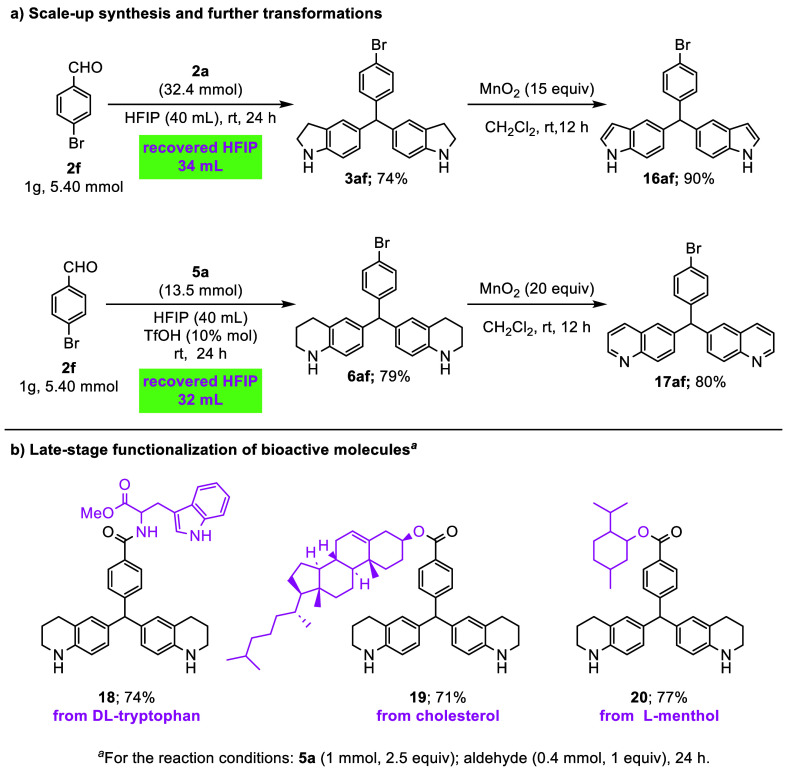
Applications: Scale-Up, Transformation, Late-Stage Functionalization

In the next step, we set out to establish a plausible mechanism for the developed strategy, which involved synthesizing TRAMs in a single step and conducting several control experiments. During optimization studies, it was determined that when 2.1 equiv of indoline were used, the condensation reaction between the nitrogen atom of indoline and 4-bromobenzaldehyde (**2f**) led to the formation of the aminal compound **4af** (Table 1, entry 1). This transformation suggests that the reaction may proceed via the formation of an iminium intermediate between the amine and aldehyde groups, followed by the nucleophilic addition of indoline to form the corresponding aminal compound. However, it was observed that the aminal structure was unstable when subjected to elution on a silica gel column, reverting to the starting materials. This instability may represent an intermediate step facilitating the formation of C-alkylation products in the reaction medium. Therefore, it was observed that, upon adding 4 equiv of indoline and stirring the reaction mixture under standard conditions for 24 h, the aminal structure completely converted at the C5 position of indoline to the Friedel–Crafts type condensation product **3af** (Scheme 5a). This transformation supports the idea that the reaction proceeds through aminal or iminium intermediates. To confirm this pathway, we aimed to trap the iminium intermediate via an intramolecular nucleophilic addition reaction.

**Scheme 5:**
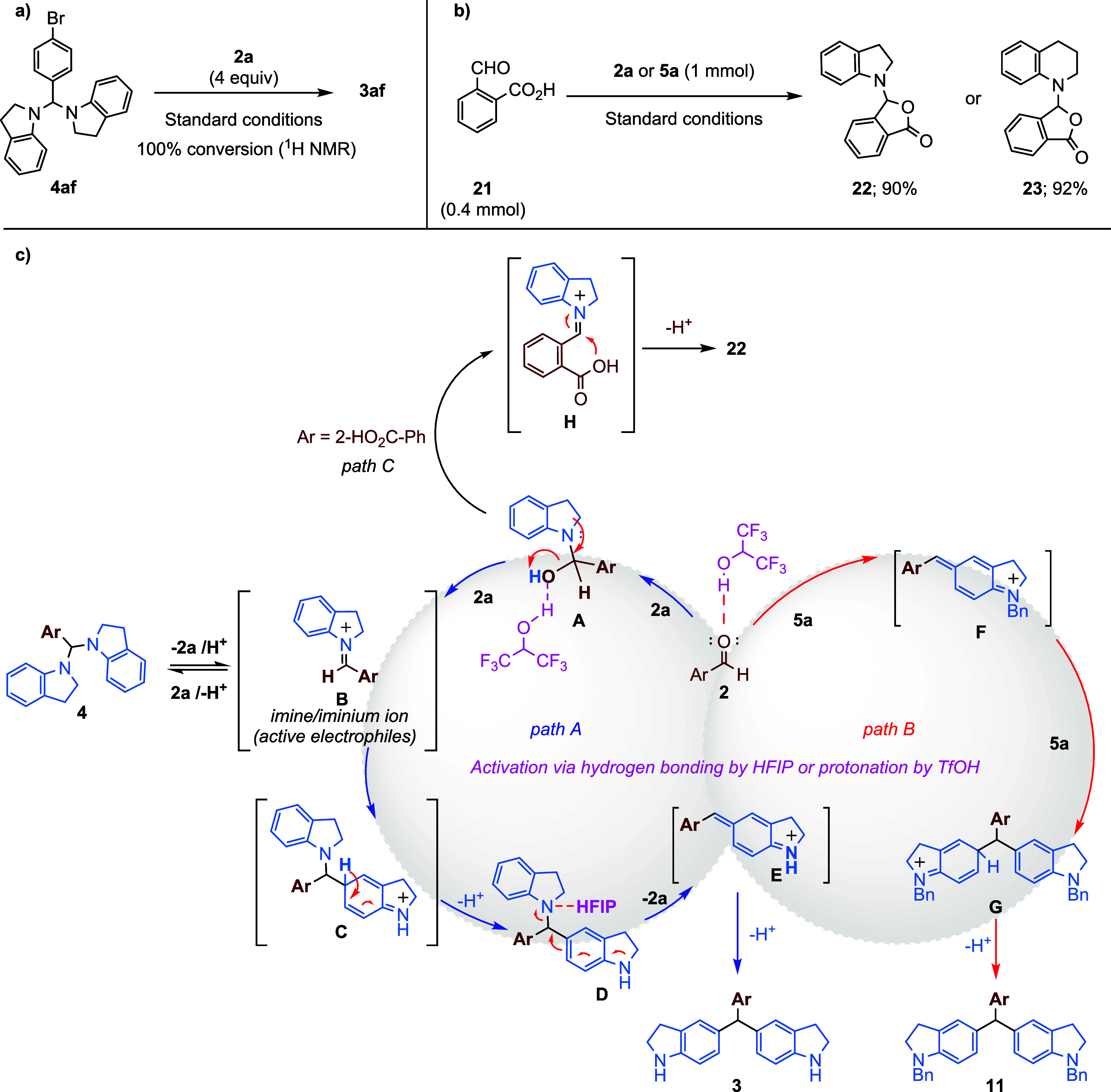
Control Experiments and Proposed Mechanistic Pathways^a^

a For the reaction conditions: **5a** (1 mmol, 2.5 equiv); aldehyde (0.4 mmol, 1 equiv), 24 h.

For this purpose, 2-formylbenzoic acid (**21**) was selected as an aldehyde derivative. To test whether the carboxylic acid group at the *ortho*-position could trap the iminium intermediate, a control experiment was performed using 2-formylbenzoic acid (**21**) and indoline (**2a**) under standard conditions. In this experiment, the hemiaminal-acetal type product **22** was obtained, instead of forming the expected C-alkylation product TRAM. A similar transformation occurred when 2-formylbenzoic acid (**21**) was reacted with tetrahydroquinoline (**5a**), yielding compound **23**.

Control experiments and literature findings confirm that the reaction of indoline (**2a**) and tetrahydroquinoline (**5a**) with aryl aldehydes follows a pathway involving iminium or aminal intermediates, as depicted in the proposed mechanism (Scheme 5c).^18^ The mechanism is represented using indoline as an example. HFIP, known for its unique solvent properties, also exhibits catalytic behavior by forming hydrogen bonds.^10^ Its ability to create hydrogen bond clusters enhances catalytic activation, particularly in combination with Lewis or Brønsted acids.10 In the presence of HFIP (or TfOH), the aldehyde carbonyl is activated either through hydrogen bonding (HFIP) or protonation (TfOH), enhancing nucleophilic attack by indoline (**2a**) or tetrahydroquinoline (**5a**). In the proposed mechanism, the first step is the activation of the aldehyde substrate’s (**2**) carbonyl group through hydrogen bonding with HFIP, allowing the nucleophile of indoline (or tetrahydroquinoline) to react more easily. This is followed by the gradual addition of the nucleophile, leading to the formation of hemiaminal structure **A**. This structure then converts into the more reactive iminium ion intermediate **B**. The nucleophile can add to this intermediate **B** in two ways: first, by adding through the nitrogen atom to form aminal structure **4**, or second, by adding through the carbon atom to form intermediate **C**. Chromatographic studies and control experiments confirm that aminal structure **4** is unstable and readily reverts to iminium intermediate **B** in the presence of HFIP (Scheme 5c). The aromatization of intermediate **C** leads to the formation of compound **D**. In this compound, the nitrogen atom in the tertiary amine structure interacts with HFIP, causing the C–N bond to break and leading to the formation of the aza-*para*-quinone methide intermediate E. The final step involves the formation of the more stable triarylmethane structure **3**, which is achieved by 1,6-conjugate addition of indoline to the aza-*para*-quinone methide structure and followed by deprotonation to restore aromaticity. This mechanism can also be modified under acid catalysis.

Notably, the presence of substituents at the C7 position of indoline and the C8 position of tetrahydroquinoline significantly hinders the reaction. The evidence suggests that bulky groups at these positions sterically or electronically interfere with the formation of the iminium intermediate, preventing the reaction from proceeding efficiently. To validate the critical role played by the hydrogen on the nitrogen atom in this mechanism, the reactions of *N*-benzylindoline and *N*-benzyl tetrahydroquinoline derivatives, in which the nitrogen atom is protected with a benzyl group, with *para*-nitrobenzaldehyde (as a model substrate) were investigated in detail (Schemes 2 and 3 ). These studies revealed that the protected indoline and tetrahydroquinoline derivatives react directly with the activating aldehyde (**2**) through Carbon-nucleophilic addition at the *para*-position (Scheme 5, path B). According to the proposed mechanism, a Friedel–Crafts-type alkylation reaction catalyzed by HFIP, or TfOH in the first step occurs between the indoline derivatives and nitrobenzaldehyde. Upon the elimination of water, a conjugated imine/iminium ion intermediate **F** is formed at the 5-position. Then, the indoline derivative adds to this intermediate, resulting in a second Friedel–Crafts alkylation **G**. The final step involves deprotonation, completing the mechanism. The formation of imine/iminium ion intermediate **B** is supported by another control experiment involving its capture by an intramolecular nucleophile (Scheme 5b). All these experimental results, including the formation of hemiaminal-acetal products with 2-formylbenzoic acid, provide strong evidence that the reaction proceeds via an iminium intermediate **H** (Scheme 5c, Path C).

To clarify the mechanistic basis of the contrasting reactivity between indoline and tetrahydroquinoline derivatives, DFT computations were performed. Mulliken charge analysis revealed that the nitrogen atom in indoline carries a more negative charge (−0.122) while THQ nitrogen shows a less negative charge (−0.054). The more electron-rich nitrogen in indoline exhibits high reactivity without catalyst, while the more electron-deficient nitrogen in THQ requires acid catalysis.

## Conclusion

We have developed a powerful and operationally simple method for the regioselective synthesis of symmetrical triarylmethanes through C–H alkylation of indolines and tetrahydroquinolines. This approach offers several notable advantages over existing methods: (1) metal-free conditions using HFIP as both solvent and promoter, (2) high regioselectivity without directing groups, (3) broad substrate scope encompassing various aryl aldehydes and heterocyclic compounds, (4) compatibility with both N–H free and *N*-protected substrates, and (5) mild reaction conditions. The practical utility of this methodology is evidenced by successful gram-scale synthesis with high HFIP recovery rates (>80%) and effective late-stage functionalization of complex bioactive molecules. Mechanistic studies reveal a novel pathway involving iminium intermediates, providing valuable insights for future reaction development. The products can be readily oxidized to their corresponding indole and quinoline derivatives, further expanding the synthetic utility. This methodology represents a significant contribution to sustainable chemistry, offering an environmentally friendly route to important triarylmethane scaffolds for applications in medicinal chemistry, materials science, and chemical biology. The combination of operational simplicity, broad scope, and practical advantages makes this approach particularly attractive for both academic and industrial applications.

## Supplementary Material



## Data Availability

The data underlying this study are available in the published article and its online Supporting Information.

## Notes

Synthetic procedures, characterization data (^1^H, ^13^C­{^1^H} NMR, HRMS), and NMR and HRMS spectra for compounds (PDF)
