# Supplementary material for: Synthesis of Indoline- and 1,2,3,4-Tetrahydroquinoline-Based Symmetrical Triarylmethanes
Source: J Org Chem. 2025 Jul 7;90(28):9954–64. doi: 10.1021/acs.joc.5c00960 (PMC12281573; doi:10.1021/acs.joc.5c00960)

Supporting Information

Synthesis of Indoline- and 1,2,3,4-Tetrahydroquinoline-based Symmetrical Triarylmethanes

Yunus Taskesenligil,[a] Murat Aslan,[a] Rabia Ardahanli,[a]

Nurullah Saracoglu[a,b]*

[a]Department of Chemistry, Faculty of Sciences, Atatürk University, Erzurum, 25240, Türkiye; [b]Biotechnology Institute, Ankara University, Ankara, 06135, Türkiye

E-mail: saracoglu@ankara.edu.tr

Table of Contents

1. General Information **S2**

2. General Procedures **S2-S4**

3. Characterization Data of Starting Materials and Products **S4-S41**

4. The details of DFT computations **S41**

5. References **S42-S45**

6. NMR Spectra of Starting Materials and Products **S46-S207**

7. HRMS Spectra of Unknown Compounds **S208-S244**

1. General Information

^{1}H NMR spectra were recorded on a Varian or Bruker 400 MHz spectrometers, with chemical shifts reported in parts per million (δ) relative to the internal solvent signal (7.26 ppm in $CDCl_3$). The peak patterns are described as follows: s = singlet, d = doublet, dd = doublet of doublets, t = triplet, q = quartet, m = multiplet. The coupling constants (J) are given in Hertz (Hz). The $^{13}C\{^1H\}$ NMR spectra were obtained using Varian or Bruker instruments at 101 MHz, referenced to the internal solvent signals (central peak at 77.16 ppm in $CDCl_3$). $CDCl_3$ was used as the NMR solvent. HRMS and APCI-MS were performed using Agilent 6530 Accurate-Mass QTOF LC/MS.

Unless otherwise noted, all reagents were purchased from commercial suppliers and used without further purification. Thin-layer chromatography (TLC) was performed on pre-coated silica gel plates (silica gel, 60 F254). Column chromatography was performed using silica gel with a pore size of 60 Å and a mesh size of 70-230. Melting points were determined using a Buchi 539 capillary melting point apparatus (uncorrected).

2. General Procedures

General procedure A: Synthesis of indolines and 1,2,3,4-tehrahydoquinolines

Indolines and 1,2,3,4-tetrahydroquinolines **1b-1p** and **5b-5n** were prepared according to the reported literature method.[1] To a solution of indole (3 mmol, 1 eq) or quinoline (3 mmol, 1 equiv) in 15 mL acetic acid at 0°C, $NaBH_3CN$ (12 mmol, 4 eq) was added slowly. The reaction mixture was warmed to room temperature and stirred for 4 hours; then 40% NaOH solution was slowly added at 0 °C until pH 9 was reached. The mixture was then extracted with ethyl acetate (3×30 mL). The organic phase was dried over Na_2SO_4 and the solvent was removed under vacuum. The crude product was purified by silica gel column chromatography using EtOAc/hexane as eluent.

General Procedure B: Synthesis of *N*-Benzylindolines and *N*-Benzyltetrahydroquinolines

N-Benzylindolines and *N*-benzyltetrahydroquinolines **10b-10p** and **13a-13o** were prepared according to the reported literature method.[1] To a solution of indoline **1** (2.5 mmol, 1 equiv) or tetrahydroquinoline **5** (2.5 mmol, 1 equiv) in 10 mL DMF at room temperature, K_2CO_3 (3 mmol, 1.2 equiv) was added, and stirred for 10 minutes, followed by slow addition of BnBr (2.75 mmol, 1.1 equiv). The reaction mixture was stirred at room temperature for 16 hours. The mixture was then extracted with ethyl acetate (3×30 mL). The organic phase was dried over Na_2SO_4 and the solvent was removed under vacuum. The crude product was purified by silica gel column chromatography using EtOAc/hexane as eluent.

General Procedure C: Synthesis of *N*-Tosylpyrrole Aldehyde and *N*-Tosylindole Aldehydes

N-Tosylpyrrole aldehyde and *N*-tosylindole aldehydes **$2w_2$** and **$2y_2$-$2y_7$** were prepared according to the reported literature method.[2] To a solution of pyrrole aldehyde (2 mmol, 1 equiv) or indole aldehyde derivatives (2 mmol, 1 equiv) in 10 mL acetonitrile (MeCN) at 0 °C, 60% NaH (3 mmol, 1.5 eq) was added slowly and stirred at the same temperature for 15 minutes. TsCl (2.2 mmol, 1.1 eq) was then added, and the reaction was stirred at room temperature for 16 hours. The reaction was quenched by adding a 15 mL solution of saturated NH_4Cl. The mixture was then extracted with dichloromethane (2×30 mL). The organic phase was dried over Na_2SO_4 and the solvent was removed under vacuum. The crude product was purified by silica gel column chromatography using EtOAc/hexane as eluent.

General Procedure D: Reaction of Indolines with Aldehydes

A solution of indoline (**1a**; 1.5 mmol, 6 equiv.) and aldehyde (**2a−y**; 0.25 mmol, 1 equiv., 32 examples) in 2 mL HFIP was stirred at room temperature for 24 hours. After the reaction time, the solvent was removed under vacuum. The crude product was purified by column chromatography using silica gel with EtOAc/hexane as eluent to give the desired product **3** (25 examples).

General Procedure E: Reaction of Tetrahydroquinolines with Aldehydes

To a solution of tetrahydroquinoline (**5a**; 1 mmol, 2.5 equiv) and aldehyde (**2a−y**; 0.4 mmol, 1 equiv., 32 examples) in 2 mL HFIP, TfOH (0.04 mmol, 0.1 eq, 10 mol%) was added. The reaction was stirred at room temperature for 5 hours. The solvent was then removed under vacuum. The crude product was purified by column chromatography on silica gel with EtOAc/hexane as eluent to give the desired products **6** (26 examples).

General Procedure F: Reaction of Indolines (or Tetrahydroquinolines) with *p*-Nitrobenzaldehyde (2j)

To a solution of (**1b−p**) (or tetrahydroquinoline (**5b−o**)) (1.0 mmol, 2.5 equiv.) and *p*-nitrobenzaldehyde (**2j**; 60.4 mg, 0.4 mmol, 1 equiv) in 2 mL HFIP, TfOH (7.06 μL, 80 μmol, 20 mol%) was added. The resulting mixture was stirred at room temperature for 24 hours. The solvent was then removed under vacuum. The crude product was purified by column chromatography on silica gel with EtOAc/hexane (20:80) as eluent to give the desired products (**3cj, 3jj, 3gj**, and **3nj**) (**6bj−gj** and **6oj**).

General Procedure G: Reaction of *N*-Benzylindolines (or *N*-Benzyltetrahydroquinolines with *p*-Nitrobenzaldehyde (2j)

To a solution of *N*-benzylindoles (**10a−p**) (or *N*-benzyltetrahydroquinolines (**14a−o**)) (1.0 mmol, 2.5 equiv.) and p-nitrobenzaldehyde (**2j**) (60.4 mg, 0.4 mmol, 1 equiv.) in 2 mL of HFIP in a thermolysis tube, TfOH (7.06 μL, 80 μmol, 20 mol%) was added. The resulting solution was heated in an oil bath (80−120 °C) and stirred for 24 hours. The solvent was removed under vacuum. The crude product was

purified by column chromatography on silica gel with EtOAc/hexane as eluent to give the desired products (**11bj−ej**, **11gj−kj** and **11nj−oj**) (**14aj−cj**, **14eh−ij** and **14mj−oj**).

3. Characterization Data of Starting Materials and Products

4-Bromoindoline (1b) General Procedure A, purified by silica gel chromatography with EtOAc/hexane (1:99). Colorless oil (480 mg, 95%).**^{1}H NMR (400 MHz, CDCl$_3$)** δ 6.87 (t, *J* = 7.6 Hz, 1H), 6.84 – 6.80 (m, 1H), 6.52 (d, *J* = 7.6 Hz, 1H), 3.83 (bs, 1H), 3.59 (t, *J* = 8.5 Hz, 2H), 3.06 (t, *J* = 8.5 Hz, 2H). **^{13}C{^{1}H} NMR (101 MHz, CDCl$_3$)** δ 152.8, 129.9, 129.00, 121.3, 119.9, 107.9, 46.4, 31.3. Spectroscopic data for the title compound were consistent with those reported in the literature.[3]

4-Chloroindoline (1c). General Procedure A, purified by silica gel chromatography with EtOAc/hexane (1:99). Colorless oil (490 mg, 96%).**^{1}H NMR (400 MHz, CDCl$_3$)** δ 6.96 (t, *J* = 7.9 Hz, 1H), 6.69 (d, *J* = 7.9 Hz, 1H), 6.49 (d, *J* = 7.9 Hz, 1H), 3.84 (bs, 1H), 3.59 (t, *J* = 8.5 Hz, 2H), 3.09 (t, *J* = 8.5 Hz, 2H). **^{13}C{^{1}H} NMR (101 MHz, CDCl$_3$)** δ 153.0, 130.6, 128.6, 127.4, 118.2, 107.2, 46.6, 29.0. Spectroscopic data for the title compound were consistent with those reported in the literature.[4]

4-Methylindoline (1d). General Procedure A, purified by silica gel chromatography with EtOAc/hexane (1:99). Colorless oil (420 mg, 83%). **^{1}H NMR (400 MHz, CDCl$_3$)** δ 6.94 (t, *J* = 7.6 Hz, 1H), 6.55 (d, *J* = 7.6 Hz, 1H), 6.50 (d, *J* = 7.6 Hz, 1H), 3.57 (t, *J* = 8.4 Hz, 2H), 2.97 (t, *J* = 8.4 Hz, 2H), 2.23 (s, 3H), (NH, 1H, not observed). **^{13}C{^{1}H} NMR (101 MHz, CDCl$_3$)** δ 151.3, 134.0, 127.9, 127.1, 119.6, 106.8, 46.8, 28.5, 18.7. Spectroscopic data for the title compound were consistent with those reported in the literature.[5]

4-Methoxyindoline (1e). General Procedure A, purified by silica gel chromatography with EtOAc/hexane (1:99). Colorless oil (470 mg, 92%). **^{1}H NMR (400 MHz, CDCl$_3$)** δ 7.02 (t, *J* = 8.0 Hz, 1H), 6.36 – 6.30 (m, 2H), 3.84 (s, 3H), 3.69 (bs, 1H), 3.58 (t, *J* = 8.5 Hz, 2H), 3.02 (t, *J* = 8.5 Hz, 2H). **^{13}C{^{1}H} NMR (101 MHz, CDCl$_3$)** δ 156.6, 153.4, 128.7, 115.9, 103.3, 101.7, 55.3, 47.5, 27.0. Spectroscopic data for the title compound were consistent with those reported in the literature.[4]

4-Nitroindoline (1f). General Procedure A, purified by silica gel chromatography with EtOAc/hexane (5:95). Red solid (405 mg, 80%; mp 185–186°C). **^{1}H NMR (400 MHz, CDCl$_3$)** δ 7.46 (d, *J* = 8.1 Hz, 1H), 7.13 (t, *J* = 8.1 Hz, 1H), 6.81 (d, *J* = 8.1 Hz, 1H), 4.05 (bs, 1H), 3.71 – 3.65 (m, 2H), 3.53 (t, *J* = 8.4 Hz, 2H). **^{13}C{^{1}H} NMR (101 MHz, CDCl$_3$)** δ 154.4, 145.9, 128.5, 126.1, 113.6, 113.2, 47.0, 31.0. Spectroscopic data for the title compound were consistent with those reported in the literature.[6]

6-Bromoindoline (1g). General Procedure A, purified by silica gel chromatography with EtOAc/hexane (1:99). Colorless oil (480 mg, 95%). **^{1}H NMR (400 MHz, CDCl$_3$)** δ 6.74 (d, *J* = 7.8 Hz, A part of AB system, 1H), 6.60 (dd, *J* = 7.8, 1.7 Hz, B part of AB system, 1H), 6.48 (d, *J* = 1.7

Hz, 1H), 3.58 (bs, 1H), 3.31 (t, J = 8.4 Hz, 2H), 2.74 (t, J = 8.4 Hz, 2H). **$^{13}C\{^1H\}$ NMR (101 MHz, $CDCl_3$)** δ 153.1, 128.1, 125.5, 120.6, 120.3, 111.7, 47.4, 29.0. Spectroscopic data for the title compound were consistent with those reported in the literature.[5]

6-Iodoindoline (1h). General Procedure A, purified by silica gel chromatography with EtOAc/hexane (1:99). Gray solid (470 mg, 93%; mp 83–90°C). **^1H NMR (400 MHz, $CDCl_3$)** δ 7.00 (dd, J = 7.6, 1.1 Hz, A part of AB system, 1H), 6.93 (d, J = 1.1 Hz, 1H), 6.83 (d, J = 7.6 Hz, B part of AB system, 1H), 3.68 (bs, 1H), 3.54 (t, J = 8.4 Hz, 2H), 2.97 (t, J = 8.4 Hz, 2H). **$^{13}C\{^1H\}$ NMR (101 MHz, $CDCl_3$)** δ 153.4, 129.2, 127.4, 126.3, 118.0, 92.0, 47.5, 29.4. Spectroscopic data for the title compound were consistent with those reported in the literature.[7]

6-Methylindoline (1i). General Procedure A, purified by silica gel chromatography with EtOAc/hexane (1:99). Colorless oil (455 mg, %90). **^1H NMR (400 MHz, $CDCl_3$)** δ 7.04 (d, J = 7.4 Hz, A part of AB system, 1H), 6.57 (d, J = 7.4 Hz, B part of AB system, 1H), 6.52 (s, 1H), 3.62 (bs, 1H), 3.57 (t, J = 8.3 Hz, 2H), 3.02 (t, J = 8.3 Hz, 2H), 2.31 (s, 3H). **$^{13}C\{^1H\}$ NMR (101 MHz, $CDCl_3$)** δ 151.9, 137.1, 126.5, 124.3, 119.5, 110.5, 47.6, 29.6, 21.5. Spectroscopic data for the title compound were consistent with those reported in the literature.[4]

6-Methoxyindoline (1j). General Procedure A, purified by silica gel chromatography with EtOAc/hexane (1:99). Colorless oil (445 mg, 88%). **^1H NMR (400 MHz, $CDCl_3$)** δ 6.99 (d, J = 8.7 Hz, A part of AB system, 1H), 6.36 – 6.15 (m, 2H), 3.75 (s, 3H), 3.56 (t, J = 8.3 Hz, 2H), 2.96 (t, J = 8.3 Hz, 2H), (NH, 1H, not observed). **$^{13}C\{^1H\}$ NMR (101 MHz, $CDCl_3$)** δ 159.8, 153.0, 124.6, 121.5, 103.2, 96.3, 55.3, 47.9, 29.0. Spectroscopic data for the title compound were consistent with those reported in the literature.[5]

Indoline-6-ol (1k). General Procedure A, purified by silica gel chromatography with EtOAc/hexane (5:95). Colorless oil (410 mg, 81%). **^1H NMR (400 MHz, $CDCl_3$)** δ 6.92 (d, J = 7.8 Hz, A part of AB system, 1H), 6.18 (dd, J = 7.8, 2.3 Hz, B part of AB system, 1H), 6.15 (d, J = 2.3 Hz, 1H), 4.60 (bs, 2H), 3.52 (t, J = 8.3 Hz, 2H), 2.93 (t, J = 8.3 Hz, 2H). **$^{13}C\{^1H\}$ NMR (101 MHz, $CDCl_3$)** δ 155.8, 151.7, 125.2, 121.9, 107.0, 99.2, 47.8, 29.0. Spectroscopic data for the title compound were consistent with those reported in the literature.[8]

7-Bromoindoline (1l). General Procedure A, purified by silica gel chromatography with EtOAc/hexane (1:99). Colorless oil (475 mg, 94%). **^1H NMR (400 MHz, $CDCl_3$)** δ 7.15 (d, J = 7.5 Hz, 1H), 7.02 (dd, J = 7.5, 0.9 Hz, 1H), 6.56 (t, J = 7.5 Hz, 1H), 3.98 (bs, 1H), 3.62 (t, J = 8.5 Hz, 2H), 3.15 (t, J = 8.5 Hz, 2H). **$^{13}C\{^1H\}$ NMR (101 MHz, $CDCl_3$)** δ 150.2, 130.6, 129.9, 123.5, 119.7, 103.3, 46.8, 30.9. Spectroscopic data for the title compound were consistent with those reported in the literature.[4]

Indoline-7-carbonitrile (1m). General Procedure A, purified by silica gel chromatography with EtOAc/hexane (5:95). Brown solid (425 mg, 84%; mp 64–65°C). **^{1}H NMR (400 MHz, $CDCl_3$)** δ 7.19 (d, *J* = 7.5 Hz, 1H), 7.13 (d, *J* = 7.5 Hz, 1H), 6.61 (t, *J* = 7.5 Hz, 1H), 4.46 (bs, 1H), 3.71 (td, *J* = 8.6, 1.4 Hz, 2H), 3.09 (t, *J* = 8.6 Hz, 2H). **$^{13}C\{^1H\}$ NMR (101 MHz, $CDCl_3$)** δ 155.2, 130.3, 129.4, 128.6, 118.0, 117.8, 90.2, 47.0, 29.2. Spectroscopic data for the title compound were consistent with those reported in the literature.[9]

7-Methylindoline (1n). General Procedure A, purified by silica gel chromatography with EtOAc/hexane (1:99). Colorless oil (470 mg, 93%). **^{1}H NMR (400 MHz, $CDCl_3$)** δ 7.01 (d, *J* = 7.4 Hz, 1H), 6.88 (d, *J* = 7.4 Hz, 1H), 6.68 (t, *J* = 7.4 Hz, 1H), 3.58 (t, *J* = 8.4 Hz, 2H), 3.07 (t, *J* = 8.4 Hz, 2H), 2.16 (s, 3H). **$^{13}C\{^1H\}$ NMR (101 MHz, $CDCl_3$)** δ 150.1, 128.7, 128.2, 122.1, 118.9, 118.8, 47.2, 30.2, 16.9. Spectroscopic data for the title compound were consistent with those reported in the literature.[7]

7-Metoksiindolin (1o). General Procedure A, purified by silica gel chromatography with EtOAc/hexane (1:99). Colorless oil (470 mg, 93%). **^{1}H NMR (400 MHz, $CDCl_3$)** δ 6.81 (d, *J* = 7.5 Hz, 1H), 6.72 (t, *J* = 7.5 Hz, 1H), 6.67 (d, *J* = 7.5 Hz, 1H), 3.83 (s, 3H), 3.59 (t, *J* = 8.4 Hz, 2H), 3.07 (t, *J* = 8.4 Hz, 2H), (NH, 1H, not observed). **$^{13}C\{^1H\}$ NMR (101 MHz, $CDCl_3$)** δ 145.5, 140.5, 130.4, 119.2, 117.2, 109.2, 55.3, 47.7, 30.4. Spectroscopic data for the title compound were consistent with those reported in the literature.[8]

7-Nitroindoline (1p). General Procedure A, purified by silica gel chromatography with EtOAc/hexane (5:95). Orange solid (415 mg, 82%; mp 85–86°C). **^{1}H NMR (400 MHz, $CDCl_3$)** δ 7.76 (dd, *J* = 8.1, 0.7 Hz, 1H), 7.19 (d, *J* = 8.1 Hz, 1H), 6.74 (bs, 1H), 6.58 – 6.50 (m, 1H), 3.90 – 3.80 (m, 2H), 3.16 (t, *J* = 8.5 Hz, 2H). **$^{13}C\{^1H\}$ NMR (101 MHz, $CDCl_3$)** δ 149.2, 133.8, 129.7, 129.2, 122.4, 116.4, 46.9, 28.3. **HRMS** (ESI-TOF) *m/z*: $[M + H]^+$ for $C_8H_9N_2O_2$ calculated 165.0659; found 165.0660.

5-Chloro-1,2,3,4-tetrahydroquinoline (5b). General Procedure A, purified by silica gel chromatography with EtOAc/hexane (1:99). Brown oil (426 mg, 80%). **^{1}H NMR (400 MHz, $CDCl_3$)** δ 6.90 (t, *J* = 7.9 Hz, 1H), 6.70 (d, *J* = 7.9 Hz, 1H), 6.39 (d, *J* = 7.9 Hz, 1H), 3.77 (bs, 1H), 3.28 (t, *J* = 6.6 Hz, 2H), 2.80 (t, *J* = 6.6 Hz, 2H), 1.98 (p, *J* = 6.6 Hz, 2H). $^{13}C\{^1H\}$ **NMR (101 MHz, $CDCl_3$)** δ 146.4, 134.8, 127.1, 119.1, 117.3, 112.5, 41.4, 24.7, 21.9. Spectroscopic data for the title compound were consistent with those reported in the literature.[10]

5-Bromo-1,2,3,4-tetrahydroquinoline (5c). General Procedure A, purified by silica gel chromatography with EtOAc/hexane (1:99). Yellow oil (290 mg, 55%). **^{1}H NMR (400 MHz, $CDCl_3$)** δ 6.87 (dd, *J* = 7.8, 1.2 Hz, 1H), 6.81 (t, *J* = 7.8 Hz, 1H), 6.41 (dd, *J* = 7.8, 1.2 Hz, 1H), 3.91 (bs, 1H),

3.26 (t, J = 6.6 Hz, 2H), 2.77 (t, J = 6.6 Hz, 2H), 1.96 (p, J = 6.6 Hz, 2H). **$^{13}C\{^1H\}$ NMR (101 MHz, $CDCl_3$)** δ 146.6, 127.6, 126.0, 120.7, 120.6, 113.2, 41.5, 27.7, 22.2. Spectroscopic data for the title compound were consistent with those reported in the literature.[3]

5-Methyl-1,2,3,4-tetrahydroquinoline (5d). General Procedure A, purified by silica gel chromatography with EtOAc/hexane (1:99). Yellow oil (320 mg, 60%). **^1H NMR (400 MHz, $CDCl_3$)** δ 6.92 (t, J = 7.5 Hz, CH, 1H), 6.55 (d, J = 7.5 Hz, 1H), 6.40 (d, J =7.5 Hz, 1H), 3.59 (bs, 1H), 3.28 (t, J = 6.6 Hz, 2H), 2.67 (t, J = 6.6 Hz, 2H), 2.21 (s, 3H), 2.01 (p, J = 6.6 Hz, 2H). **$^{13}C\{^1H\}$ NMR (101 MHz, $CDCl_3$)** δ 145.0, 137.1, 126.2, 120.1, 118.9, 112.5, 41.6, 24.1, 22.6, 19.4. Spectroscopic data for the title compound were consistent with those reported in the literature.[3]

5-Methoxy-1,2,3,4-tetrahydroquinoline (5e). General Procedure A, purified by silica gel chromatography with EtOAc/hexane (1:99). Yellow oil (453 mg, 85%). **^1H NMR (400 MHz, $CDCl_3$)** δ 6.93 (t, J = 8.1 Hz, 1H), 6.22 (d, J = 8.1 Hz, 1H), 6.16 (d, J = 8.1 Hz, 1H), 3.79 (s, 3H), 3.25 (t, J = 6.6 Hz, 2H), 2.66 (t, J = 6.6 Hz, 2H), 1.96 – 1.89 (m, J = 6.6 Hz, 2H), (NH, 1H, not observed). **$^{13}C\{^1H\}$ NMR (101 MHz, $CDCl_3$)** δ 155.2, 143.2, 123.9, 106.8, 105.0, 96.2, 52.4, 38.8, 19.2, 17.9. Spectroscopic data for the title compound were consistent with those reported in the literature.[10]

7-Chloro-1,2,3,4-tetrahydroquinoline (5f). General Procedure A, purified by silica gel chromatography with EtOAc/hexane (1:99). Colorless solid (442 mg, 83%; mp 64–65°C). **^1H NMR (400 MHz, $CDCl_3$)** δ 6.84 (d, J = 8.0 Hz, A part of AB system, 1H), 6.56 (dd, J = 8.0, 2.0 Hz, B part of AB system, 1H), 6.46 (d, J = 2,0 Hz, 1H), 3.89 (bs, 1H), 3.29 (t, J = 6.4 Hz, 2H), 2.71 (t, J = 6.4 Hz, 2H), 1.92 (p, J = 6.4 Hz, 2H). **$^{13}C\{^1H\}$ NMR (101 MHz, $CDCl_3$)** δ 145.5, 132.1, 130.6, 120.0, 117.0, 113.8, 41.9, 26.7, 21.9. Spectroscopic data for the title compound were consistent with those reported in the literature.[11]

7-Bromo-1,2,3,4-tetrahidrokinolin (5g). General Procedure A, purified by silica gel chromatography with EtOAc/hexane (1:99). Yellow solid (450 mg, 85%, mp 72–73 °C). **^1H NMR (400 MHz, $CDCl_3$)** δ 6.78 (d, J = 8.0 Hz, A part of AB system, 1H), 6.68 (d, J = 8.0 Hz, B part of AB system, 1H), 6.59 (s, 1H), 3.88 (bs, 1H), 3.28 (t, J = 6.3 Hz, 2H), 2.69 (t, J = 6.3 Hz, 2H), 1.91 (p, J = 6.3 Hz, 2H). **$^{13}C\{^1H\}$ NMR (101 MHz, $CDCl_3$)** δ 146.1, 130.8, 120.2, 120.0, 119.5, 116.3, 41.8, 26.7, 21.8. Spectroscopic data for the title compound were consistent with those reported in the literature.[12]

7-Methyl-1,2,3,4-tetrahydroquinoline (5h). General Procedure A, purified by silica gel chromatography with EtOAc/hexane (1:99). Yellow oil (340 mg, 70%). **^1H NMR (400 MHz, $CDCl_3$)** δ 6.91 (d, J = 7.6 Hz, A part of AB system, 1H), 6.52 (d, J = 7.6 Hz, B part of AB system, 1H), 6.36 (s, 1H), 3.74 (bs, 1H), 3.33 (t, J = 6.4 Hz, 2H), 2.80 (t, J = 6.4 Hz, 2H), 2.30 (s, 3H), 1.99 (p, J = 6.4 Hz, 2H). **$^{13}C\{^1H\}$ NMR (101 MHz, $CDCl_3$)** δ 144.7, 136.3, 129.4, 118.5, 117.9, 114.8, 42.1, 26.7,

22.5, 21.2. Spectroscopic data for the title compound were consistent with those reported in the literature.[11]

7-Methoxy-1,2,3,4-tetrahydroquinoline (5i). General Procedure A, purified by silica gel chromatography with EtOAc/hexane (1:99). Brown oil (522 mg, 98%). **^{1}H NMR (400 MHz, $CDCl_3$)** δ 6.90 (d, *J* = 8.2 Hz, A part of AB system, 1H), 6.25 (dd, *J* = 8.2, 2.5 Hz, B part of AB system, 1H), 6.09 (d, *J* = 2.5 Hz, 1H), 3.78 (s, 3H), 3.33 (t, *J* = 6.4 Hz, 2H), 2.75 (t, *J* = 6.4 Hz, 2H), 1.97 (p, *J* = 6.4 Hz, 2H), (NH, 1H, not observed). **^{13}C{^{1}H} NMR (101 MHz, $CDCl_3$)** δ 158.9, 145.6, 130.1, 114.1, 102.9, 99.5, 55.2, 42.0, 26.4, 22.5. Spectroscopic data for the title compound were consistent with those reported in the literature.[13]

8-Fluoro-1,2,3,4-tetrahydroquinoline (5j). General Procedure A, purified by silica gel chromatography with EtOAc/hexane (1:99). Colorless oil (317 mg, 70%). **^{1}H NMR (400 MHz, $CDCl_3$)** δ 6.84 – 6.66 (m, 2H), 6.54 – 6.43 (m, 1H), 3.99 (bs, 1H), 3.34 (t, *J* = 6.4 Hz, 2H), 2.78 (t, *J* = 6.4 Hz, 2H), 1.95 (p, *J* = 6.4 Hz, 1H). **^{13}C{^{1}H} NMR (101 MHz, $CDCl_3$)** δ 151.0 (d, *J* = 237.5 Hz), 133.3 (d, *J* = 12.1 Hz), 124.6 (d, *J* = 2.8 Hz), 123.7 (d, *J* = 3.7 Hz), 115.6 (d, *J* = 7.4 Hz), 112.2 (d, *J* = 18.2 Hz), 41.4, 26.6 (d, *J* = 3.0 Hz), 21.9. Spectroscopic data for the title compound were consistent with those reported in the literature.[10]

8-Chloro-1,2,3,4-tetrahydroquinoline (5k). General Procedure A, purified by silica gel chromatography with EtOAc/hexane (1:99). Colorless oil (300 mg, 55%). **^{1}H NMR (400 MHz, $CDCl_3$)** δ 7.07 (dd, *J* = 7.7, 1.4 Hz, 1H), 6.86 (dd, *J* = 7.7, 1.4 Hz, 1H), 6.51 (t, *J* = 7.7 Hz, 1H), 4.42 (bs, 1H), 3.40 (d, *J* = 6.4 Hz, 2H), 2.78 (t, *J* = 6.4 Hz, 2H), 1.94 (p, *J* = 6.4 Hz, 2H). **^{13}C{^{1}H} NMR (101 MHz, $CDCl_3$)** δ 140.8, 127.8, 126.8, 122.7, 118.1, 116.3, 41.9, 27.3, 21.8. Spectroscopic data for the title compound were consistent with those reported in the literature.[10]

8-Bromo-1,2,3,4-tetrahydroquinoline (5l). General Procedure A, purified by silica gel chromatography with EtOAc/hexane (1:99). Yellow oil (424 mg, 80%). **^{1}H NMR (400 MHz, $CDCl_3$)** δ 7.24 (d, *J* = 7.7 Hz, 1H), 6.90 (dd, *J* = 7.7, 1.0 Hz, 1H), 6.46 (t, *J* = 7.7 Hz, 1H), 4.44 (bs, 1H), 3.40 (t, *J* = 6.4 Hz, 2H), 2.79 (t, *J* = 6.4 Hz, 2H), 1.94 (p, *J* = 6.4 Hz, 2H). **^{13}C{^{1}H} NMR (101 MHz, $CDCl_3$)** δ 141.8, 130.1, 128.5, 122.9, 117.0, 108.8, 42.1, 27.5, 21.8. Spectroscopic data for the title compound were consistent with those reported in the literature.[14]

8-Methyl-1,2,3,4-tetrahydroquinoline (5m). General Procedure A, purified by silica gel chromatography with EtOAc/hexane (1:99). Colorless oil (296 mg, 55%). **^{1}H NMR (400 MHz, $CDCl_3$)** δ 6.91 (d, *J* = 7.6 Hz, 1H), 6.88 (d, *J* = 7.6 Hz, 1H), 6.59 (t, *J* = 7.6 Hz, 1H), 3.41 (t, *J* = 6.4 Hz, 2H), 2.82 (t, *J* = 6.4 Hz, 2H), 2.11 (s, 3H), 1.98 (p, *J* = 6.4 Hz, 2H), (NH, 1H, not observed).

$^{13}C\{^1H\}$ NMR (101 MHz, $CDCl_3$) δ 141.8, 126.9, 126.4, 120.2, 119.9, 115.5, 41.4, 26.4, 21.3, 16.2. Spectroscopic data for the title compound were consistent with those reported in the literature.[3,]

8-Methoxy-1,2,3,4-tetrahydroquinoline (5n). General Procedure A, purified by silica gel chromatography with EtOAc/hexane (1:99). Yellow oil (373 mg, 70%). **^1H NMR (400 MHz, $CDCl_3$)** δ 6.64 – 6.56 (m, 3H), 3.83 (s, 3H), 3.38 – 3.30 (t, *J* = 6.4 Hz, 2H), 2.78 (t, *J* = 6.4 Hz, 2H), 1.97 (p, *J* = 6.4 Hz, 2H), (NH, 1H, not observed). **$^{13}C\{^1H\}$ NMR (101 MHz, $CDCl_3$)** δ 146.4, 134.4, 121.8, 121.6, 115.9, 107.5, 55.5, 41.6, 26.7, 22.2. Spectroscopic data for the title compound were consistent with those reported in the literature.[14]

1-Benzylindoline (10a). General Procedure B, purified by silica gel chromatography with EtOAc/hexane (5:95). Colorless liquid (481 mg, 92%). **^1H NMR (400 MHz, $CDCl_3$)** δ 7.47 – 7.29 (m, 5H), 7.18 – 7.09 (m, 2H), 6.73 (t, *J* = 7.6 Hz, 1H), 6.57 (d, *J* = 7.6 Hz, 1H), 4.31 (s, 2H), 3.36 (t, *J* = 8.3 Hz, 2H), 3.02 (t, *J* = 8.3 Hz, 2H). **$^{13}C\{^1H\}$ NMR (101 MHz, $CDCl_3$)** δ 152.6, 138.6, 130.1, 128.6, 128.0, 127.4, 127.2, 124.6, 117.8, 107.2, 53.8, 53.7, 28.6. Spectroscopic data for the title compound were consistent with those reported in the literature.[1]

1-Benzyl-4-bromoindoline (10b). General Procedure B, purified by silica gel chromatography with EtOAc/hexane (5:95). Brown solid (554 mg, 77%; mp 54–56°C). **^1H NMR (400 MHz, $CDCl_3$)** δ 7.43 – 7.30 (m, 5H), 6.95 (t, *J* = 7.9 Hz, 1H), 6.83 (d, *J* = 7.9 Hz, 1H), 6.43 (d, *J* = 7.9 Hz, 1H), 4.29 (s, 2H), 3.42 (t, *J* = 8.5 Hz, 2H), 3.04 (t, *J* = 8.5 Hz, 2H). **$^{13}C\{^1H\}$ NMR (101 MHz, $CDCl_3$)** δ 153.6, 137.9, 130.2, 129.2, 128.7, 127.9, 127.4, 120.3, 119.8, 105.4, 53.3, 52.4, 29.9. **HRMS** (ESI-TOF) *m/z*: $[M + H]^+$ for $C_{15}H_{15}BrN$ calculated 288.0382; found 288.0380.

1-Benzyl-4-chloroindoline (10c). General Procedure B, purified by silica gel chromatography with EtOAc/hexane (5:95). Dirty white solid (505 mg, 83%; mp 55–56 °C). **^1H NMR (400 MHz, $CDCl_3$)** δ 7.42 – 7.28 (m, 5H), 7.00 (t, *J* = 7.9 Hz, 1H), 6.66 (d, *J* = 7.9 Hz, 1H), 6.38 (d, *J* = 7.9 Hz, 1H), 4.29 (s, 2H), 3.42 (t, *J* = 8.5 Hz, 2H), 3.05 (t, *J* = 8.5 Hz, 2H). **$^{13}C\{^1H\}$ NMR (101 MHz, $CDCl_3$)** δ 153.9, 138.0, 130.7, 129.0, 128.7, 128.0, 127.9, 127.4, 117.6, 105.0, 53.3, 52.9, 27.9. **HRMS** (ESI-TOF) *m/z*: $[M + H]^+$ for $C_{15}H_{15}ClN$ calculated 244.0888; found 244.0889.

1-Benzyl-4-methylindoline (10d). General Procedure B, purified by silica gel chromatography with EtOAc/hexane (5:95). Colorless oil (435 mg, 78%). **^1H NMR (400 MHz, $CDCl_3$)** δ 7.58 – 7.39 (m, 5H), 7.15 (t, *J* = 7.1 Hz, 1H), 6.68 (d, *J* = 7.2 Hz, 1H), 6.53 (d, *J* = 7.2 Hz, 1H), 4.39 (s, 2H), 3.47 (t, *J* = 8.3 Hz, 2H), 3.04 (t, *J* = 8.3 Hz, 2H), 2.37 (s, 3H). **$^{13}C\{^1H\}$ NMR (101 MHz, $CDCl_3$)** δ 152.4, 138.7, 134.0, 128.5 (2C) , 128.0, 127.5, 127.1, 119.2, 104.7, 53.8, 53.4, 27.3, 18.7. Spectroscopic data for the title compound were consistent with those reported in the literature.[1]

1-Benzyl-4-methoxyindoline (10e). General Procedure B, purified by silica gel chromatography with EtOAc/hexane (5:95). Off-white solid (484 mg, 81%; mp 51–52°C). **^{1}H NMR (400 MHz, $CDCl_3$)** δ 7.43 – 7.26 (m, 5H), 7.07 (t, *J* = 8.0 Hz, 1H), 6.32 (d, *J* = 8.0 Hz, 1H), 6.25 (d, *J* = 8.0 Hz, 1H), 4.28 (s, 2H), 3.85 (s, 3H), 3.36 (t, *J* = 8.4 Hz, 2H), 2.96 (t, *J* = 8.4 Hz, 2H). **^{13}C{^{1}H} NMR (101 MHz, $CDCl_3$)** δ 156.3, 154.3, 138.6, 128.8, 128.5, 128.0, 127.1, 115.9, 101.3, 101.1, 55.3, 53.7 (2C), 25.6. **HRMS** (ESI-TOF) *m/z*: $[M + H]^+$ for $C_{16}H_{18}NO$ calculated 240.1383; found 240.1388.

1-Benzyl-4-nitroindoline (10f). General Procedure B, purified by silica gel chromatography with EtOAc/hexane (5:95). Brown oil (476 mg, 75%). **^{1}H NMR (400 MHz, $CDCl_3$)** δ 7.41 (d, *J* = 8.1 Hz, 1H), 7.38 – 7.29 (m, 5H), 7.15 (t, *J* = 8.1 Hz, 1H), 6.65 (d, *J* = 8.1 Hz, 1H), 4.34 (s, 2H), 3.58 – 3.43 (m, 4H). **^{13}C{^{1}H} NMR (101 MHz, $CDCl_3$)** δ 154.6, 145.6, 137.3, 128.8 (2C), 127.7, 127.6, 127.0, 112.0, 110.8, 52.9, 52.7, 29.7. **HRMS** (ESI-TOF) *m/z*: $[M + H]^+$ for $C_{15}H_{15}N_2O_2$ calculated 255.1128; found 255.1128.

1-Benzyl-6-bromoindoline (10g). General Procedure B, purified by silica gel chromatography with EtOAc/hexane (5:9). Yellow oil (516 mg, 71%). **^{1}H NMR (400 MHz, $CDCl_3$)** δ 7.50 – 7.26 (m, 5H), 6.93 (d, *J* = 7.7 Hz, A part of AB system, 1H), 6.78 (dd, *J* = 7.7, 1.7 Hz, B part of AB system, 1H), 6.62 (d, *J* = 1.7 Hz, 1H), 4.25 (s, 2H), 3.37 (t, *J* = 8.4 Hz, 2H), 2.93 (t, *J* = 8.4 Hz, 2H). **^{13}C{^{1}H} NMR (101 MHz, $CDCl_3$)** δ 153.9, 137.7, 129.0, 128.6, 127.8, 127.3, 125.5, 121.1, 120.0, 109.7, 53.4, 52.9, 28.0. Spectroscopic data for the title compound were consistent with those reported in the literature.[15]

1-Benzyl-6-iodoindoline (10h). General Procedure B, purified by silica gel chromatography with EtOAc/hexane (5:95). Dirty white solid (719 mg, 86%; mp 60–61°C). **^{1}H NMR (400 MHz, $CDCl_3$)** δ 7.48 – 7.34 (m, 5H), 7.05 (dd, *J* = 7.5, 1.3 Hz, A part of AB system, 1H), 6.91 – 6.84 (m, 2H), 4.27 (s, 2H), 3.38 (t, *J* = 8.4 Hz, 2H), 2.97 (t, *J* = 8.4 Hz, 2H). **^{13}C{^{1}H} NMR (101 MHz, $CDCl_3$)** δ 153.9, 137.7, 129.9, 128.6, 127.9, 127.4, 126.4, 126.1, 115.5, 92.5, 53.3, 52.9, 28.1. **HRMS** (ESI-TOF) *m/z*: $[M + H]^+$ for $C_{15}H_{15}IN$ calculated 336.0244; found 336.0247.

1-Benzyl-6-methylindoline (10i). General Procedure B, purified by silica gel chromatography with EtOAc/hexane (5:95). Brown oil (424 mg, 76%). **^{1}H NMR (400 MHz, $CDCl_3$)** δ 7.67 – 7.40 (m, 5H), 7.18 (d, *J* = 7.3 Hz, A part of AB system, 1H), 6.70 (d, *J* = 7.3 Hz, B part of AB system, 1H), 6.56 (s, 1H), 4.41 (s, 2H), 3.47 (t, *J* = 8.2 Hz, 2H), 3.10 (t, *J* = 8.2 Hz, 2H), 2.48 (s, 3H). **^{13}C{^{1}H} NMR (101 MHz, $CDCl_3$)** δ 152.8, 138.7, 137.1, 128.5, 127.9, 127.1 (2C) , 124.2, 118.4, 108.0, 53.9, 53.7, 28.2, 21.8. Spectroscopic data for the title compound were consistent with those reported in the literature.[16]

1-Benzyl-6-methoxyindoline (10j). General Procedure B, purified by silica gel chromatography with EtOAc/hexane (5:95). Green oil (472 mg, 79%). **^{1}H NMR (400 MHz, $CDCl_3$)** δ 7.60 – 7.35 (m, 5H), 7.12 (d, *J* = 8.0 Hz, A part of AB system, 1H), 6.36 (dd, *J* = 8.0, 2.3 Hz, B part of AB system, 1H),

6.29 (d, J = 2.2 Hz, 1H), 4.37 (s, 2H), 3.87 (s, 3H), 3.46 (t, J = 8.3 Hz, 2H), 3.04 (t, J = 8.2 Hz, 2H). **$^{13}C\{^1H\}$ NMR (101 MHz, $CDCl_3$)** δ 160.1, 153.8, 138.3, 128.5, 127.9, 127.1, 124.5, 122.3, 101.5, 94.8, 55.3, 54.1, 53.3, 27.7. Spectroscopic data for the title compound were consistent with those reported in the literature.[15]

1-Benzylindoline-6-ol (10k). General Procedure B, purified by silica gel chromatography with EtOAc/hexane (5:95). Colorless oil (400 mg, 71%). **^1H NMR (400 MHz, $CDCl_3$)** δ 7.50 – 7.23 (m, 5H), 6.90 (d, J = 7.7 Hz, A part of AB system, 1H), 6.13 (dd, J = 7.7, 2.2 Hz, B part of AB system, 1H), 6.05 (d, J = 2.2 Hz, 1H), 4.22 (s, 2H), 3.34 (t, J = 8.3 Hz, 2H), 2.89 (t, J = 8.3 Hz, 2H). **$^{13}C\{^1H\}$ NMR (101 MHz, $CDCl_3$)** δ 155.7, 153.9, 138.1, 128.5, 128.2, 127.2, 124.8, 122.1, 104.3, 96.0, 53.9, 53.4, 27.7. **HRMS** (ESI-TOF) *m/z*: $[M + H]^+$ for $C_{15}H_{16}NO$ calculated 226.1226; found 226.1228.

1-Benzyl-7-bromoindoline (10l). General Procedure B, purified by silica gel chromatography with EtOAc/hexane (5:95). Colorless oil (619 mg, 86%). **^1H NMR (400 MHz, $CDCl_3$)** δ 7.50 – 7.29 (m, 6H), 7.09 (d, J = 7.4 Hz, 1H), 6.66 (t, J = 7.4 Hz, 1H), 4.87 (s, 2H), 3.46 (t, J = 8.8 Hz, 2H), 3.03 (t, J = 8.8 Hz, 2H). **$^{13}C\{^1H\}$ NMR (101 MHz, $CDCl_3$)** δ 148.9, 139.1, 134.0, 132.8, 128.5, 128.0, 127.1, 123.8, 120.1, 103.6, 54.7, 53.6, 28.7. **HRMS** (ESI-TOF) *m/z*: $[M + H]^+$ for $C_{15}H_{15}BrN$ calculated 288.0382; found 226.0384.

1-Benzylindoline-7-carbonitrile (10m). General Procedure B, purified by silica gel chromatography with EtOAc/hexane (5:95). Brown oil (428 mg, 73%). **^1H NMR (400 MHz, $CDCl_3$)** δ 7.63 – 7.48 (m, 1H), 7.27 – 7.15 (m, 5H), 7.10 (dd, J = 7.0, 1.1 Hz, 1H), 6.54 (dd, J = 8.4, 7.0 Hz, 1H), 4.37 (s, 2H), 3.60 (t, J = 8.8 Hz, 1H), 3.00 (t, J = 8.8 Hz, 1H). **$^{13}C\{^1H\}$ NMR (101 MHz, $CDCl_3$)** δ 146.1, 137.0, 135.7, 133.7, 128.74, 128.70, 128.0, 127.5, 124.5 (2C), 117.3, 54.8, 27.6. **HRMS** (ESI-TOF) *m/z*: $[M + H]^+$ for $C_{16}H_{15}N_2$ calculated 235.1230; found 235.1231.

1-Benzyl-7-methylindoline (10n). General Procedure B, purified by silica gel chromatography with EtOAc/hexane (5:95). Colorless oil (418 mg, 75%). **^1H NMR (400 MHz, $CDCl_3$)** δ 7.64 – 7.41 (m, 5H), 7.18 (d, J = 7.1 Hz, 1H), 7.08 (d, J = 7.2 Hz, 1H), 6.90 (t, J = 7.2 Hz, 1H), 4.64 (s, 2H), 3.52 (t, J = 8.6 Hz, 2H), 3.13 (t, J = 8.6 Hz, 2H), 2.56 (s, 3H). **$^{13}C\{^1H\}$ NMR (101 MHz, $CDCl_3$)** δ 150.7, 139.8, 131.2, 130.7, 128.5, 127.6, 127.0, 122.6, 120.1, 119.4, 56.2, 53.9, 28.9, 19.4. **HRMS** (ESI-TOF) *m/z*: $[M + H]^+$ for $C_{16}H_{18}N$ calculated 224.1434; found 224.1439.

1-Benzyl-7-methoxyindoline (10o). General Procedure B, purified by silica gel chromatography with EtOAc/hexane (5:95). Colorless oil (472 mg, 79%). **^1H NMR (400 MHz, $CDCl_3$)** δ 7.67 – 7.08 (m, 5H), 7.08 – 6.57 (m, 3H), 4.69 (s, 2H), 3.86 (s, 3H), 3.28 (t, J = 8.7 Hz, 2H), 2.97 (t, J = 8.7 Hz, 2H). **$^{13}C\{^1H\}$ NMR (101 MHz, $CDCl_3$)** δ 146.3, 140.4, 139.6, 132.0, 128.4, 128.3, 126.8, 119.4, 117.8,

111.4, 55.7, 55.5, 53.6, 29.2. Spectroscopic data for the title compound were consistent with those reported in the literature.[1]

1-Benzyl-7-nitroindoline (10p). General Procedure B, purified by silica gel chromatography with EtOAc/hexane (5:95). Brown oil (431 mg, 68%). **^{1}H NMR (400 MHz, $CDCl_3$)** δ 7.59 (d, *J* = 7.9 Hz, 1H), 7.36 – 7.21 (m, 5H), 7.16 (dd, *J* = 7.0, 1.1 Hz, 1H), 6.69 – 6.51 (m, 1H), 4.43 (s, 2H), 3.66 (t, *J* = 8.9 Hz, 2H), 3.06 (t, *J* = 8.9 Hz, 2H). **^{13}C{^{1}H} NMR (101 MHz, $CDCl_3$)** δ 146.2, 137.0, 135.7, 133.7, 128.7 (2C) , 128.0, 127.5, 124.5, 117.3, 54.8 (2C) , 27.6. **HRMS** (ESI-TOF) *m/z*: $[M + H]^+$ for $C_{15}H_{15}N_2O_2$ calculated 255.1128; found 255.1129.

1-Benzyl-1,2,3,4-tetrahydroquinoline (13a). General Procedure B, purified by silica gel chromatography with EtOAc/hexane (5:95). Light yellow liquid (502 mg, 90%). **^{1}H NMR (400 MHz, $CDCl_3$)** δ 7.36 – 7.22 (m, 5H), 7.03 – 6.95 (m, 2H), 6.60 (t, *J* = 7.5 Hz, 1H), 6.53 (d, *J* = 7.5 Hz, 1H), 4.50 (s, 2H), 3.38 (t, *J* = 6.3 Hz, 2H), 2.84 (t, *J* = 6.3 Hz, 2H), 2.03 (p, *J* = 6.3 Hz, 2H). **^{13}C{^{1}H} NMR (101 MHz, $CDCl_3$)** δ 145.7, 139.0, 129.1, 128.7, 127.3, 126.8, 126.7, 122.3, 115.9, 111.1, 55.3, 50.0, 28.3, 22.5. Spectroscopic data for the title compound were consistent with those reported in the literature.[17]

1-Benzyl-5-chloro-1,2,3,4-tetrahydroquinoline (13b). General Procedure B, purified by silica gel chromatography with EtOAc/hexane (5:95). Yellow oil (560 mg, 87%). **^{1}H NMR (400 MHz, $CDCl_3$)** δ 7.42 – 7.36 (m, 2H), 7.33 – 7.27 (m, 3H), 6.92 (t, *J* = 8.1 Hz, 1H), 6.72 (dd, *J* = 8.1, 0.9 Hz, 1H) 6.45 (d, *J* = 8.1 Hz, 1H), 4.54 (s, 2H), 3.42 (t, *J* = 6.5 Hz, 2H), 2.94 (t, *J* = 6.5 Hz, 2H), 2.08 (p, *J* = 6.5 Hz, 2H). **^{13}C{^{1}H} NMR (101 MHz, $CDCl_3$)** δ 147.1, 138.5, 134.5, 128.7, 127.4, 127.0, 126.5, 119.9, 116.7, 109.6, 55.7, 49.7, 25.6, 22.0. Spectroscopic data for the title compound were consistent with those reported in the literature.[18]

1-Benzyl-5-bromo-1,2,3,4-tetrahydroquinoline (13c). General Procedure B, purified by silica gel chromatography with EtOAc/hexane (5:95). Yellow oil (642 mg, 85%). **^{1}H NMR (400 MHz, $CDCl_3$)** δ 7.53 – 7.45 (m, 2H), 7.44 – 7.38 (m, 3H), 7.03 (dd, *J* = 8.1, 1.0 Hz, 1H), 6.95 (t, *J* = 8.1 Hz, 1H), 6.61 (d, *J* = 8.1 Hz, 1H), 4.63 (s, 2H), 3.50 (t, *J* = 6.5 Hz, 2H), 3.05 (t, *J* = 6.5 Hz, 2H), 2.17 (p, *J* = 6.5 Hz, 2H). **^{13}C{^{1}H} NMR (101 MHz, $CDCl_3$)** δ 147.1, 138.3, 128.7, 127.8, 126.9, 126.4, 125.6, 121.3, 119.8, 110.2, 55.5, 49.7, 28.7, 22.2. Spectroscopic data for the title compound were consistent with those reported in the literature.[19]

1-Benzyl-5-methyl-1,2,3,4-tetrahydroquinoline (13d). General Procedure B, purified by silica gel chromatography with EtOAc/hexane (5:95). Yellow oil (568 mg, 89%). **^{1}H NMR (400 MHz, $CDCl_3$)** δ 7.51 – 7.32 (m, 5H), 7.03 (t, *J* = 7.7 Hz, 1H), 6.64 (d, *J* = 7.7 Hz, 1H), 6.55 (d, *J* = 7.7 Hz, CH, 1H), 4.60 (s, 2H), 3.83 – 3.28 (m, 2H), 2.85 (t, *J* = 6.5 Hz, 2H), 2.36 (s, 3H), 2.23 – 2.12 (m, 2H). **^{13}C{^{1}H}**

NMR (101 MHz, $CDCl_3$) δ 145.9, 139.3, 136.5, 128.6, 126.8, 126.6, 126.5, 120.8, 118.2, 109.5, 55.9, 49.7, 25.0, 22.4, 20.1. Spectroscopic data for the title compound were consistent with those reported in the literature.[18]

1-Benzyl-5-methoxy-1,2,3,4-tetrahydroquinoline (13e). General Procedure B, purified by silica gel chromatography with EtOAc/hexane (5:95). Yellow oil (525 mg, 83%). **^{1}H NMR (400 MHz, $CDCl_3$)** δ 7.40 – 7.23 (m, 5H), 6.98 (t, *J* = 8.2 Hz, 1H), 6.28 (d, *J* = 8.2 Hz, 1H), 6.27 (d, *J* = 8.2 Hz, 1H), 4.51 (s, 2H), 3.84 (s, 3H), 3.47 – 3.18 (t, *J* = 6.5 Hz, 2H), 2.78 (t, *J* = 6.5 Hz, 2H), 2.16 – 1.74 (p, *J* = 6.5 Hz, 2H). **^{13}C{^{1}H} NMR (101 MHz, $CDCl_3$)** δ 157.5, 146.7, 139.3, 128.7, 126.9, 126.8, 126.7, 110.2, 104.9, 98.8, 55.8, 55.5, 49.6, 22.0, 21.3. **HRMS** (ESI-TOF) *m/z*: $[M + H]^+$ for $C_{17}H_{20}NO$ calculated 254.1539; found 254.1544.

1-Benzyl-7-chloro-1,2,3,4-tetrahydroquinoline (13f). General Procedure B, purified by silica gel chromatography with EtOAc/hexane (5:95). Colorless oil (604 mg, 80%). **^{1}H NMR (400 MHz, $CDCl_3$)** δ 7.45 – 7.38 (m, 2H), 7.38 – 7.30 (m, 3H), 6.96 (d, *J* = 7.9 Hz, 1H), 6.72 – 6.52 (m, 2H), 4.53 (s, 2H), 3.42 (t, *J* = 6.1 Hz, 2H), 2.84 (t, *J* = 6.1 Hz, 2H), 2.06 (p, *J* = 6.1 Hz, 2H). **^{13}C{^{1}H} NMR (101 MHz, $CDCl_3$)** δ 146.6, 138.1, 132.6, 129.8, 128.8, 127.0, 126.6, 120.6, 115.5, 110.4, 54.9, 49.5, 27.8, 22.1. Spectroscopic data for the title compound were consistent with those reported in the literature.[20]

1-Benzyl-7-bromo-1,2,3,4-tetrahydroquinoline (13g). General Procedure B, purified by silica gel chromatography with EtOAc/hexane (5:95). Colorless oil (642 mg, 85%). **^{1}H NMR (400 MHz, $CDCl_3$)** δ 7.41 – 7.33 (m, 2H), 7.31 – 7.24 (m, 3H), 6.85 (d, *J* = 7.9 Hz, 1H), 6.71 (dd, *J* = 7.9, 1.8 Hz, 1H), 6.67 (d, *J* = 1.8 Hz, 1H), 4.48 (s, 2H), 3.36 (t, *J* = 6.2 Hz, 2H), 2.77 (t, *J* = 6.2 Hz, 2H), 2.00 (p, *J* = 6.2 Hz, 2H). **^{13}C{^{1}H} NMR (101 MHz, $CDCl_3$)** δ 146.8, 138.0, 130.2, 128.8, 127.1, 126.6, 121.1, 120.8, 118.5, 113.3, 54.9, 49.5, 27.9, 22.1. Spectroscopic data for the title compound were consistent with those reported in the literature.[21]

1-Benzyl-7-methyl-1,2,3,4-tetrahydroquinoline (13h). General Procedure B, purified by silica gel chromatography with EtOAc/hexane (5:95). Colorless oil (516 mg, 87%). **^{1}H NMR (400 MHz, $CDCl_3$)** δ 7.44 – 7.29 (m, 5H), 6.95 (d, *J* = 7.4 Hz, A part of AB system, 1H), 6.49 (d, *J* = 7.4 Hz, B part of AB system, 1H), 6.44 (s, 1H), 4.55 (s, 2H), 3.39 (t, *J* = 6.2 Hz, 1H), 2.85 (t, *J* = 6.2 Hz, 1H), 2.26 (s, 3H), 2.06 (p, *J* = 6.2 Hz, 1H). **^{13}C{^{1}H} NMR (101 MHz, $CDCl_3$)** δ 145.6, 139.2, 136.9, 129.0, 128.7, 126.8, 126.7, 119.5, 116.8, 111.6, 55.2, 49.8, 28.0, 22.6, 21.7. Spectroscopic data for the title compound were consistent with those reported in the literature.[19]

1-Benzyl-7-methoxy-1,2,3,4-tetrahydroquinoline (13i). General Procedure B, purified by silica gel chromatography with EtOAc/hexane (5:95). Colorless oil (513 mg, 81%). **^{1}H NMR (400 MHz,**

$CDCl_3$) δ 7.39 – 7.25 (m, 5H), 6.93 (d, *J* = 8.1 Hz, 1H), 6.20 (dd, *J* = 8.1, 2.2 Hz, 1H), 6.14 (d, *J* = 2.2 Hz, 1H), 4.50 (s, 2H), 3.70 (s, 3H), 3.39 (t, *J* = 6.3 Hz, 2H), 2.80 (t, *J* = 6.3 Hz, 2H), 2.03 (p, *J* = 6.3 Hz, 2H). **$^{13}C\{^1H\}$ NMR (101 MHz, $CDCl_3$)** δ 159.3, 146.5, 138.8, 129.4, 128.7, 126.9, 126.7, 115.2, 100.3, 97.8, 55.3, 55.2, 49.9, 27.6, 22.7. **HRMS** (ESI-TOF) *m/z*: $[M + H]^+$ for $C_{17}H_{20}NO$ calculated 254.1539; found 254.1543.

1-Benzyl-8-fluoro-1,2,3,4-tetrahydroquinoline (13j). General Procedure B, purified by silica gel chromatography with EtOAc/hexane (5:95). Brown oil (500 mg, 83%). **^1H NMR (400 MHz, $CDCl_3$)** δ 7.5 (d, *J* = 7.4 Hz, 2H), 7.4 (t, *J* = 7.4 Hz, 2H), 7.36 (t, *J* = 7.4 Hz, 1H), 6.99 – 6.88 (m, 2H), 6.83 – 6.76 (m, 1H), 4.45 (s, 2H), 3.15 (t, *J* = 6.3 Hz, 2H), 2.85 (t, *J* = 6.3 Hz, 2H), 1.91, (p, *J* = 6.3 Hz, 2H). **$^{13}C\{^1H\}$ NMR (101 MHz, $CDCl_3$)** δ 154.3 (d, *J* = 242.7 Hz), 139.7, 135.0 (d, *J* = 7.7 Hz), 129.4 (d, *J* = 3.1 Hz), 128.4, 128.0 (d, *J* = 0.8 Hz), 127.0, 124.8 (d, *J* = 2.8 Hz), 119.0 (d, *J* = 8.4 Hz), 114.1 (d, *J* = 21.4 Hz), 58.6 (d, *J* = 8.7 Hz), 48.6, 28.2 (d, *J* = 2.8 Hz), 19.8. Spectroscopic data for the title compound were consistent with those reported in the literature.[21]

1-Benzyl-8-chloro-1,2,3,4-tetrahydroquinoline (13k). General Procedure B, purified by silica gel chromatography with EtOAc/hexane (5:95). Colorless oil (515 mg, 80%). **^1H NMR (400 MHz, $CDCl_3$)** δ 7.67 (d, *J* = 7.4 Hz, 1H), 7.45 (t, *J* = 7.4 Hz, 1H), 7.37 (t, *J* = 7.4 Hz, 1H), 7.30 (d, *J* = 7.6 Hz, 1H), 7.07 (d, *J* = 7.6 Hz, 1H), 6.93 (t, *J* = 7.6 Hz, 1H), 4.33 (s, 2H), 3.16 – 2.96 (m, 2H), 2.89 (t, *J* = 6.6 Hz, 2H), 1.92 – 1.83 (m, 2H). **$^{13}C\{^1H\}$ NMR (101 MHz, $CDCl_3$)** δ 145.5, 139.4, 131.54, 128.35 (2C), 128.35 (2C), 128.2, 127.1, 122.1, 57.7, 46.7, 27.9, 17.0. **HRMS** (ESI-TOF) *m/z*: $[M + H]^+$ for $C_{16}H_{17}ClN$ calculated 258.1044; found 258.1048.

1-Benzyl-8-methyl-1,2,3,4-tetrahydroquinoline (13m). General Procedure B, purified by silica gel chromatography with EtOAc/hexane (5:95). Yellow oil (462 mg, 78%). **^1H NMR (400 MHz, $CDCl_3$)** δ 8.07 (d, *J* = 7.6 Hz, 2H), 7.89 (t, *J* = 7.6 Hz, 2H), 7.80 (t, *J* = 7.6 Hz, 1H), 7.57 (d, *J* = 7.1 Hz, 1H), 7.51 – 7.40 (m, 2H), 4.58 (s, 2H), 3.57 – 3.47 (m, 2H), 3.34 (t, *J* = 6.7 Hz, 2H), 2.89 (s, 3H), 2.36 – 2.25 (m, 2H). **$^{13}C\{^1H\}$ NMR (101 MHz, $CDCl_3$)** δ 147.7, 139.6, 131.3, 128.9, 128.9, 128.4, 127.4 (2C), 126.8, 121.6, 57.7, 46.8, 27.7, 18.6, 16.7. Spectroscopic data for the title compound were consistent with those reported in the literature.[17]

1-Benzyl-8-methoxy-1,2,3,4-tetrahydroquinoline (13n). General Procedure B, purified by silica gel chromatography with EtOAc/hexane (5:95). Colorless oil (532 mg, 84%). **^1H NMR (400 MHz, $CDCl_3$)** δ 7.63 (d, *J* = 7.3 Hz, 2H), 7.46 (t, *J* = 7.3 Hz, 2H), 7.38 (t, *J* = 7.3 Hz, 1H), 6.98 (t, *J* = 7.8 Hz, 1H), 6.87 – 6.78 (m, 2H), 4.31 (s, 2H), 3.95 (s, 3H), 3.18 – 3.02 (m, 2H), 2.88 (t, *J* = 6.5 Hz, 2H), 1.96 – 1.83 (m, 2H). **$^{13}C\{^1H\}$ NMR (101 MHz, $CDCl_3$)** δ 152.1, 140.4, 137.5, 129.4, 128.8, 128.2, 126.9, 121.9, 120.9, 108.6, 57.7, 55.4, 47.3, 27.9, 17.7. Spectroscopic data for the title compound were consistent with those reported in the literature.[22]

1-Benzyl-2-methyl-1,2,3,4-tetrahydroquinoline (13o). General Procedure B, purified by silica gel chromatography with EtOAc/hexane (5:95). Colorless oil (522 mg, 88%). **^{1}H NMR (400 MHz, $CDCl_3$)** δ 7.40 – 7.24 (m, 5H), 7.07 (d, *J* = 7.6 Hz, 1H), 7.00 (t, *J* = 7.6 Hz, 1H), 6.63 (t, *J* = 7.6 Hz, 1H), 6.45 (d, *J* = 7.6 Hz, 1H), 4.64 – 4.49 (m, 2H), 3.69 – 3.59 (m, 1H), 3.06 – 2.91 (m, 1H), 2.87 – 2.77 (m, 1H), 2.16 – 2.04 (m, 1H), 1.94 – 1.84 (m, 1H), 1.24 (d, *J* = 6.4 Hz, 3H). **$^{13}C\{^1H\}$ NMR (101 MHz, $CDCl_3$)** δ 144.8, 139.5, 128.9, 128.6, 127.2, 126.7, 126.4, 121.8, 115.5, 111.4, 53.4, 53.1, 28.2, 24.1, 19.1. Spectroscopic data for the title compound were consistent with those reported in the literature.[19]

1-Tosyl-1*H*-pyrrole-2-carbaldehyde (2w$_2$). General Procedure C, purified by silica gel chromatography with EtOAc/hexane (1:99). White solid (428 mg, 86%; mp 93–94°C). **^{1}H NMR (400 MHz, $CDCl_3$)** δ 9.96 (s, 1H), 7.82 – 7.76 (m, AA' part of AA'BB' system, 2H), 7.61 (dd, *J* = 3.4, 1.7 Hz, 1H), 7.35 – 7.28 (m, BB' part of AA'BB' system, 2H), 7.15 (dd, *J* = 3.4, 1.7 Hz, CH, 1H), 6.39 (t, *J* = 3.4 Hz, 1H), 2.40 (s, 3H). **$^{13}C\{^1H\}$ NMR (101 MHz, $CDCl_3$)** δ 179.1, 146.1, 135.3, 133.6, 130.3, 129.6, 127.6, 124.6, 112.5, 21.8. Spectroscopic data for the title compound were consistent with those reported in the literature.[23]

1-Tosyl-1*H*-indole-2-carbaldehyde (2y$_2$). General Procedure C, purified by silica gel chromatography with EtOAc/hexane (1:99). White solid (453 mg, 91%; mp 121–122°C). **^{1}H NMR (400 MHz, $CDCl_3$)** δ 10.54 (s, 1H), 8.24 (d, *J* = 8.6 Hz, 1H), 7.69 – 7.64 (m, AA' part of AA'BB' system, 2H), 7.62 (d, *J* = 7.8 Hz, 1H), 7.52 (t, *J* = 7.7Hz, 1H), 7.47 (s, 1H), 7.31 (t, *J* = 7.7 Hz, 1H), 7.26 (s, 1H), 7.12 – 7.16 (m, BB' part of AA'BB' system, 2H), 2.33 (s, 3H). **$^{13}C\{^1H\}$ NMR (101 MHz, $CDCl_3$)** δ 183.5, 145.7, 138.6, 137.9, 134.7, 130.1, 128.9, 128.3, 126.8, 124.9, 123.7, 119.0, 115.5, 21.7. Spectroscopic data for the title compound were consistent with those reported in the literature.[24]

1-Tosyl-1*H*-indole-3-carbaldehyde (2y$_3$). General Procedure C, purified by silica gel chromatography with EtOAc/hexane (1:99). Purple solid (478 mg, 96%; mp 139–140°C). **^{1}H NMR (400 MHz, $CDCl_3$)** δ 10.01 (s, 1H), 8.20 – 8.13 (m, 2H), 7.87 (d, *J* = 8.2 Hz, 1H), 7.81 – 7.73 (m, AA' part of AA'BB' system, 2H), 7.35 – 7.23 (m, 2H), 7.221 – 7.15 (m, BB' part of AA'BB' system, 2H), 2.25 (s, 3H). **$^{13}C\{^1H\}$ NMR (101 MHz, $CDCl_3$)** δ 185.5, 146.2 (2C), 136.4, 135.2, 134.3, 130.4, 127.3, 126.3, 125.1, 122.6, 122.4, 113.3, 21.6. Spectroscopic data for the title compound were consistent with those reported in the literature.[2]

1-Tosyl-1*H*-indole-4-carbaldehyde (2y$_4$). General Procedure C, purified by silica gel chromatography with EtOAc/hexane (1:99). Off-white solid (398 mg, 80%; mp 143–144°C). **^{1}H NMR (400 MHz, $CDCl_3$)** δ 10.16 (s, 1H), 8.27 (d, *J* = 8.4 Hz, 1H), 7.78 – 7.74 (m, 3H), 7.71 (d, *J* = 7.1 Hz, 1H), 7.51 – 7.43 (m, 2H), 7.25 – 7.19 (m, BB' part of AA'BB' system, 2H), 2.33 (s, 3H).

$^{13}C\{^1H\}$ NMR (101 MHz, $CDCl_3$) δ 192.3, 145.6, 135.5, 135.1, 130.2, 129.6, 129.5, 129.1, 129.0, 126.9, 124.4, 119.3, 108.6, 21.7. Spectroscopic data for the title compound were consistent with those reported in the literature.[25]

1-Tosyl-1*H*-indole-5-carbaldehyde ($2y_5$). General Procedure C, purified by silica gel chromatography with EtOAc/hexane (1:99). Pink solid (443 mg, 89%; mp 133–134°C). **^1H NMR (400 MHz, $CDCl_3$)** δ 10.04 (s, 1H), 8.13 (d, *J* = 8.6 Hz, A part of AB system, 1H), 8.08 (d, *J* = 8.6, 1.3 Hz, 1H), 7.87 (dd, *J* = 8.6, 1.3 Hz, B part of AB system, 1H), 7.83 – 7.77 (m, AA' part of AA'BB' system, 2H), 7.69 (d, *J* = 3.7 Hz, 1H), 7.31 – 7.24 (m, BB' part of AA'BB' system, 2H), 6.79 (d, *J* = 3.7 Hz, 1H), 2.37 (s, 3H). **$^{13}C\{^1H\}$ NMR (101 MHz, $CDCl_3$)** δ 192.9, 146.7, 139.2, 136.0, 133.3, 131.9, 131.2, 129.1, 128.0, 126.4, 125.9, 115.0, 110.5, 22.7. Spectroscopic data for the title compound were consistent with those reported in the literature.[25]

1-Tosyl-1*H*-indole-6-carbaldehyde ($2y_6$). General Procedure C, purified by silica gel chromatography with EtOAc/hexane (1:99). Green solid (453 mg, 91%; mp 154–155°C). **^1H NMR (400 MHz, $CDCl_3$)** δ 10.08 (s, 1H), 8.49 (s, 1H), 7.83 – 7.76 (m, 4H), 7.65 (d, *J* = 8.2 Hz, B part of AB system, 1H), 7.27 – 7.21 (m, BB' part of AA'BB' system, 2H), 6.73 (d, *J* = 3.6 Hz, 1H), 2.34 (s, 3H). **$^{13}C\{^1H\}$ NMR (101 MHz, $CDCl_3$)** δ 192.0, 145.7, 135.8, 135.0, 134.6, 133.3, 130.2, 130.2, 127.0, 123.9, 122.1, 116.4, 109.0, 21.7. Spectroscopic data for the title compound were consistent with those reported in the literature.[25]

1-Tosyl-1*H*-indole-7-carbaldehyde ($2y_7$). General Procedure C, purified by silica gel chromatography with EtOAc/hexane (1:99). Red solid (496 mg, 83%; mp 107–108°C). **^1H NMR (400 MHz, $CDCl_3$)** δ 10.70 (s, 1H), 7.79 (dd, *J* = 7.7, 0.9 Hz, 1H), 7.70 (dd, *J* = 7.7, 1.1 Hz, 1H), 7.66 (d, *J* = 3.7 Hz, 1H), 7.49 – 7.41 (m, AA' part of AA'BB' system, 2H), 7.34 (t, *J* = 7.7 Hz, 1H), 7.18 – 7.10 (m, BB' part of AA'BB' system, 2H), 6.77 (d, *J* = 3.7 Hz, 1H), 2.30 (s, 3H). **$^{13}C\{^1H\}$ NMR (101 MHz, $CDCl_3$)** δ 190.8, 145.5, 134.2, 134.0, 133.6, 130.4, 129.9, 126.9, 126.8, 126.2, 125.6, 124.4, 111.6, 21.7. **HRMS** (ESI-TOF) *m/z*: $[M + H]^+$ for $C_{16}H_{14}NO_3S$ calculated 300.0689; found 300.0692.

5,5'-(Phenylmethylene)diindoline (3aa). General Procedure D, purified by silica gel chromatography with EtOAc/hexane (20:80). Purple oil (62 mg, 71%). **^1H NMR (400 MHz, $CDCl_3$)** δ 7.25 – 7.22 (m, 2H), 7.16 (t, *J* = 7.2 Hz, 1H), 7.11 (d, *J* = 7.2 Hz, 2H), 6.86 (s, 2H), 6.73 (d, *J* = 8.0 Hz, A part of AB system, 2H), 6.53 (d, *J* = 8.0 Hz, B part of AB system, 2H), 5.33 (s, 1H), 3.51 (t, *J* = 8.3 Hz, 4H), 3.39 (bs, 2H), 2.94 (t, *J* = 8.3 Hz, 4H). **$^{13}C\{^1H\}$ NMR (101 MHz, $CDCl_3$)** δ 149.8, 145.6, 135.3, 129.5, 129.4, 128.2, 128.1, 125.8, 125.7, 109.1, 55.9, 47.5, 29.9. **HRMS** (ESI-TOF) *m/z*: $[M + H]^+$ for $C_{23}H_{23}N_2$ calculated 327.1856; found 327.1856.

5,5'-(*p*-Tolylmethylene)diindoline (3ab). General Procedure D, purified by silica gel chromatography with EtOAc/hexane (20:80). Off-white solid (62 mg, 73%; mp 87–88°C). **^{1}H NMR (400 MHz, $CDCl_3$)** δ 7.13 – 7.05 (m, AA' part of AA'BB' system, 2H), 7.05 – 6.99 (m, BB' part of AA'BB' system, 2H), 6.87 (s, 2H), 6.75 (d, *J* = 8.0 Hz, A part of AB system, 2H), 6.55 (d, *J* = 8.0 Hz, B part of AB system, 2H), 5.31 (s, 1H), 3.53 (t, *J* = 8.3 Hz, 4H), 2.96 (t, *J* = 8.3 Hz, 4H), 2.31 (s, 3H), (2H, not observed). **^{13}C{^{1}H} NMR (101 MHz, $CDCl_3$)** δ 149.3, 142.6, 136.0, 135.4, 129.8, 129.4, 129.0, 128.4, 125.8, 109.6, 55.6, 47.6, 30.0, 21.2. **HRMS** (ESI-TOF) *m/z*: $[M + H]^+$ for $C_{24}H_{25}N_2$ calculated 341.2012; found 341.2011.

5,5'-((4-(*tert*-Butyl)phenyl)methylene)diindoline (3ac). General Procedure D, purified by silica gel chromatography with EtOAc/hexane (20:80). Off-white solid (72 mg, 75%; mp 81–82°C). **^{1}H NMR (400 MHz, $CDCl_3$)** δ 7.38 – 7.31 (m, AA' part of AA'BB' system, 2H), 7.17 – 7.10 (m, BB' part of AA'BB' system, 2H), 6.96 (s, 2H), 6.83 (d, *J* = 8.0 Hz, A part of AB system, 2H), 6.59 (d, *J* = 8.0 Hz, B part of AB system, 2H), 5.38 (s, 1H), 3.67 (bs, 2H), 3.55 (t, *J* = 8.3 Hz, 4H), 3.00 (t, *J* = 8.3 Hz, 4H), 1.37 (s, 9H). **^{13}C{^{1}H} NMR (101 MHz, $CDCl_3$)** δ 149.8, 148.5, 142.4, 135.7, 129.5, 129.0, 128.3, 125.8, 125.0, 109.2, 55.6, 47.6, 34.4, 31.5, 30.0. **HRMS** (ESI-TOF) *m/z*: $[M + H]^+$ for $C_{27}H_{31}N_2$ calculated 383.2482; found 383.2482.

5,5'-((4-Fluorophenyl)methylene)diindoline (3ad). General Procedure D, purified by silica gel chromatography with EtOAc/hexane (20:80). Off-white solid (74 mg, 86%; mp 82–83 °C). **^{1}H NMR (400 MHz, $CDCl_3$)** δ 7.12 – 7.06 (m, AA' part of AA'BB' system, 2H), 6.99 – 6.92 (m, BB' part of AA'BB' system, 2H), 6.86 (s, 2H), 6.74 (d, *J* = 7.9 Hz, A part of AB system, 2H), 6.57 (d, *J* = 7.9 Hz, B part of AB system, 2H), 5.34 (s, 1H), 3.61 (bs, 2H), 3.54 (t, *J* = 8.4 Hz, 4H), 2.97 (t, *J* = 8.3 Hz, 4H). **^{13}C{^{1}H} NMR (101 MHz, $CDCl_3$)** δ 161.1 (d, *J* = 244.0 Hz), 149.8, 141.3 (d, *J* = 3.0 Hz), 135.1, 130.7 (d, *J* = 7.7 Hz), 129.6, 128.1, 125.5, 114.7 (d, *J* = 21.1 Hz), 109.1, 55.1, 47.4, 29.8. **HRMS** (ESI-TOF) *m/z*: $[M + H]^+$ for $C_{23}H_{22}FN_2$ calculated 345.1762; found 345.1761.

5,5'-((4-Chlorophenyl)methylene)diindoline (3ae). General Procedure D, purified by silica gel chromatography with EtOAc/hexane (20:80). Off-white solid (71 mg, 78%; mp > 300 °C). **^{1}H NMR (400 MHz, $CDCl_3$)** δ 7.25 – 7.18 (m, AA' part of AA'BB' system, 2H), 7.11 – 7.02 (m, BB' part of AA'BB' system, 2H), 6.84 (s, 2H), 6.72 (d, *J* = 8.0 Hz, A part of AB system, 2H), 6.55 (d, *J* = 8.0 Hz, B part of AB system, 2H), 5.31 (s, 1H), 3.54 (t, *J* = 8.3 Hz, 4H), 2.96 (t, *J* = 8.3 Hz, 4H), (2H, not observed). **^{13}C{^{1}H} NMR (101 MHz, $CDCl_3$)** δ 150.0, 144.3, 134.9, 131.7, 130.9, 129.8, 128.3 (2C), 125.7, 109.3, 55.4, 47.6, 30.0. **HRMS** (ESI-TOF) *m/z*: $[M + H]^+$ for $C_{23}H_{22}ClN_2$ calculated 361.1466; found 361.1445.

5,5'-((4-Bromophenyl)methylene)diindoline (3af). General Procedure D, purified by silica gel chromatography with EtOAc/hexane (20:80). Off-white solid (86 mg, 85%; mp 88–89 °C). **^{1}H NMR**

(400 MHz, $CDCl_3$) δ 7.47 – 7.36 (m, AA' part of AA'BB' system, 2H), 7.10 – 7.01 (m, BB' part of AA'BB' system, 2H), 6.88 (s, 2H), 6.76 (d, *J* = 7.5 Hz, A part of AB system, 2H), 6.57 (d, *J* = 7.5 Hz, B part of AB system, 2H), 5.33 (s, 1H), 3.76 (bs, 2H), 3.54 (t, *J* = 8.3 Hz, 4H), 2.98 (t, *J* = 8.3 Hz, 4H). **$^{13}C\{^1H\}$ NMR (101 MHz, $CDCl_3$)** δ 150.0, 144.8, 134.6, 131.2, 131.2, 129.6, 128.2, 125.6, 119.7, 109.1, 55.3, 47.6, 29.9. **HRMS** (ESI-TOF) *m/z*: $[M + H]^+$ for $C_{23}H_{22}BrN_2$ calculated 405.0961; found 405.0942.

5,5'-((4-Iodophenyl)methylene)diindoline (3ag). General Procedure D, purified by silica gel chromatography with EtOAc/hexane (20:80). Off-white solid (99 mg, 87%; mp 83–84 °C). **^1H NMR (400 MHz, $CDCl_3$)** δ 7.62 – 7.53 (m, AA' part of AA'BB' system, 2H), 6.92 – 6.86 (m, BB' part of AA'BB' system, 2H), 6.83 (s, 2H), 6.71 (d, *J* = 8.0 Hz, A part of AB system, 2H), 6.55 (d, *J* = 8.0 Hz, B part of AB system, 2H), 5.28 (s, 1H), 3.53 (t, *J* = 8.4 Hz, 4H), 2.96 (t, *J* = 8.3 Hz, 4H), (2H, not observed). **$^{13}C\{^1H\}$ NMR (101 MHz, $CDCl_3$)** δ 150.0, 145.5, 137.2, 134.7, 131.6, 129.7, 128.3, 125.7, 109.2, 91.3, 55.5, 47.6, 30.0. **HRMS** (ESI-TOF) *m/z*: $[M + H]^+$ for $C_{23}H_{22}IN_2$ calculated 453.0822; found 453.0822.

5,5'-((4-Methoxyphenyl)methylene)diindoline (3ah). General Procedure D, purified by silica gel chromatography with EtOAc/hexane (20:80). Off-white solid (68 mg, 76%; mp 103–104 °C). **^1H NMR (400 MHz, $CDCl_3$)** δ 7.08 – 6.98 (m, AA' part of AA'BB' system, 2H), 6.87 (s, 2H), 6.83 – 6.78 (m, BB' part of AA'BB' system, 2H), 6.74 (d, *J* = 8.1 Hz, A part of AB system, 2H), 6.56 (d, *J* = 8.1 Hz, B part of AB system, 2H), 5.31 (s, 1H), 3.78 (s, 3H), 3.53 (t, *J* = 8.3 Hz, 4H), 2.96 (t, *J* = 8.3 Hz, 4H), (2H, not observed). **$^{13}C\{^1H\}$ NMR (101 MHz, $CDCl_3$)** δ 157.8, 149.8, 137.9, 135.8, 130.4, 129.6, 128.3, 125.8, 113.5, 109.2, 55.3, 55.1, 47.7, 30.0. **HRMS** (ESI-TOF) m/z: $[M + H]^+$ for $C_{24}H_{25}N_2O$ calculated 357.1961; found 357.1952.

5,5'-((4-Nitrophenyl)methylene)diindoline (3aj). General Procedure D, purified by silica gel chromatography with EtOAc/hexane (20:80). Indoline (**2a**; 1.5 mmol, 2.5 equiv) and aldehyde **11j** (0.6 mmol, 1 equiv). Yellow solid (196 mg, 88%; mp 185–186 °C). **^1H NMR (400 MHz, $CDCl_3$)** δ 8.15 – 8.09 (m, AA' part of AA'BB' system, 2H), 7.32 – 7.27 (m, BB' part of AA'BB' system, 2H), 6.82 (s, 2H), 6.71 (dd, *J* = 8.0, 1.1 Hz, A part of AB system, 2H), 6.56 (d, *J* = 8.0 Hz, B part of AB system, 2H), 5.42 (s, 1H), 3.55 (t, *J* = 8.4 Hz, 4H), 2.97 (t, *J* = 8.4 Hz, 4H), (2H, not observed). **$^{13}C\{^1H\}$ NMR (101 MHz, $CDCl_3$)** δ 153.7, 150.4, 146.3, 133.6, 130.3, 130.0, 128.3, 125.7, 123.5, 109.3, 55.9, 47.6, 29.9. **HRMS** (ESI-TOF) *m/z*: $[M + H]^+$ for $C_{23}H_{22}N_3O_2$ calculated 372.1707; found 372.1706.

5,5'-((4-(Trifluoromethyl)phenyl)methylene)diindoline (3ak). General Procedure D, purified by silica gel chromatography with EtOAc/hexane (20:80). Indoline (**2a**; 1.5 mmol, 2.5 equiv) and aldehyde **11k** (0.6 mmol, 1 equiv). Brown solid (206 mg, 87%; mp 84–85 °C). **^1H NMR (400 MHz,**

$CDCl_3$) δ 7.49 – 7.35 (m, AA' part of AA'BB' system, 2H), 7.24 – 7.10 (m, BB' part of AA'BB' system, 2H), 6.76 (s, 2H), 6.64 (d, *J* = 7.9 Hz, A part of AB system, 2H), 6.47 (d, *J* = 7.9 Hz, B part of AB system, 2H), 5.31 (s, 1H), 3.52 (bs, 2H), 3.45 (t, *J* = 8.4 Hz, 4H), 2.88 (t, *J* = 8.3 Hz, 4H). **$^{13}C\{^1H\}$ NMR (101 MHz, $CDCl_3$)** δ 149.9, 149.8, 134.5, 129.8, 129.7, 128.3, 128.1 (q, *J* = 32.2 Hz), 125.7, 125.1 (q, *J* = 3.6 Hz), 124.5 (q, *J* = 270.0 Hz) 109.3, 55.8, 47.5, 29.9. **HRMS** (ESI-TOF) *m/z*: $[M + H]^+$ for $C_{24}H_{22}F_3N_2$ calculated 395.1730; found 395.1722.

Methyl 4-(di(indolin-5-yl)methyl)benzoate (3al). General Procedure D, purified by silica gel chromatography with EtOAc/hexane (20:80). Indoline (**2a**; 1.5 mmol, 2.5 equiv) and aldehyde **11l** (0.6 mmol, 1 equiv). Gray solid (187 mg, 81%; mp 82–83 °C). **^1H NMR (400 MHz, $CDCl_3$)** δ 8.02 – 7.85 (m, AA' part of AA'BB' system, 2H), 7.24 – 7.13 (m, BB' part of AA'BB' system, 2H), 6.85 (s, 2H), 6.73 (d, *J* = 8.0 Hz, A part of AB system, 2H), 6.56 (d, *J* = 8.0 Hz, B part of AB system, 2H), 5.40 (s, 1H), 3.89 (s, 3H), 3.54 (t, *J* = 8.3 Hz, 4H), 2.97 (d, *J* = 8.3 Hz, 4H), (2H, not observed). **$^{13}C\{^1H\}$ NMR (101 MHz, $CDCl_3$)** δ 167.3, 151.2, 150.0, 134.5, 129.8, 129.6, 129.5, 128.3, 127.9, 125.7, 109.3, 56.0, 52.1, 47.6, 30.0. **HRMS** (ESI-TOF) *m/z*: $[M + H]^+$ for $C_{25}H_{25}N_2O_2$ calculated 385.1911; found 385.1904.

3-(Di(indolin-5-yl)methyl)phenol (3am). General Procedure D, purified by silica gel chromatography with EtOAc/hexane (20:80). White solid (59 mg, 69%; mp 121–122 °C). **^1H NMR (400 MHz, $CDCl_3$)** δ 7.07 (t, *J* = 7.8 Hz, 1H), 6.86 (s, 2H), 6.72 (d, *J* = 7.9 Hz, A part of AB system, 2H), 6.66 (d, *J* = 7.7 Hz, 1H), 6.57 (d, *J* = 8.0 Hz, 1H), 6.54 – 6.48 (m, 3H), 5.24 (s, 1H), 3.42 (t, *J* = 8.2 Hz, 4H), 2.87 (t, *J* = 8.1 Hz, 4H), (OH, NH, 3H, not observed). **$^{13}C\{^1H\}$ NMR (101 MHz, $CDCl_3$)** δ 156.1, 149.0, 147.2, 136.0, 130.1, 129.3, 128.4, 125.8, 121.4, 116.6, 113.2, 110.1, 55.8, 47.5, 30.0. **HRMS** (ESI-TOF) *m/z*: $[M + H]^+$ for $C_{23}H_{23}N_2O$ calculated 343.1805; found 343.1800.

5,5'-(*m*-Tolylmethylene)diindoline (3an). General Procedure D, purified by silica gel chromatography with EtOAc/hexane (20:80). Off-white solid (62 mg, 73%; mp 89–90 °C). **^1H NMR (400 MHz, $CDCl_3$)** δ 7.15 (t, *J* = 7.7 Hz, 1H), 6.99 (d, *J* = 7.7 Hz, 1H), 6.96 (s, 1H), 6.92 (d, *J* = 7.7 Hz, 1H), 6.88 (s, 2H), 6.75 (d, *J* = 7.8 Hz, A part of AB system, 2H), 6.56 (d, *J* = 7.8 Hz, B part of AB system, 2H), 5.32 (s, 1H), 3.53 (t, *J* = 8.2 Hz, 4H), 2.97 (t, *J* = 8.2 Hz, 4H), 2.29 (s, 3H), (2H, not observed). **$^{13}C\{^1H\}$ NMR (101 MHz, $CDCl_3$)** δ 149.7, 145.5, 137.6, 135.5, 130.2, 129.6, 128.3, 128.0, 126.7, 126.6, 125.8, 109.2, 55.9, 47.6, 30.0, 21.6. **HRMS** (ESI-TOF) *m/z*: $[M + H]^+$ for $C_{24}H_{25}N_2$ calculated 341.2012; found 341.2012.

5,5'-((3-Bromophenyl)methylene)diindoline (3ao). General Procedure D, purified by silica gel chromatography with EtOAc/hexane (20:80). Off-white solid (84 mg, 83%; mp 86–87 °C). **^1H NMR (400 MHz, $CDCl_3$)** δ 7.31 (d, *J* = 7.7 Hz, 1H), 7.27 (s, 1H), 7.13 (t, *J* = 7.7 Hz, 1H), 7.05 (d, *J* = 7.7 Hz, 1H), 6.85 (s, 2H), 6.74 (d, *J* = 7.8 Hz, A part of AB system, 2H), 6.59 (d, *J* = 7.8 Hz, B part of AB

system, 2H), 5.32 (s, 1H), 3.57 (t, *J* = 7.2 Hz, 4H), 2.98 (t, *J* = 7.9 Hz, 4H), (2H, not observed). **^{13}C{^{1}H} NMR (101 MHz, CDCl$_3$)** δ 149.2, 148.0, 134.9, 132.3, 130.0, 129.7, 129.1, 128.3, 128.1, 125.6, 122.4, 109.7, 55.6, 47.4, 29.9. **HRMS** (ESI-TOF) *m/z*: $[M + H]^+$ for $C_{23}H_{22}BrN_2$ calculated 405.0961; found 405.0948.

5,5'-(*o*-Tolylmethylene)diindoline (3ap). General Procedure D, purified by silica gel chromatography with EtOAc/hexane (20:80). Off-white solid (62 mg, 73%; mp 87–88 °C). **^{1}H NMR (400 MHz, CDCl$_3$)** δ 7.15 (t, *J* = 7.5 Hz, 1H), 6.99 (d, *J* = 7.7 Hz, 1H), 6.96 (s, 1H), 6.92 (d, *J* = 7.7 Hz, 1H), 6.88 (s, 2H), 6.75 (d, *J* = 7.8 Hz, A part of AB system, 2H), 6.56 (d, *J* = 7.8 Hz, B part of AB system, 2H), 5.32 (s, 1H), 3.53 (t, *J* = 8.2 Hz, 4H), 2.97 (t, *J* = 8.2 Hz, 4H), 2.29 (s, 3H), (2H, not observed). **^{13}C{^{1}H} NMR (101 MHz, CDCl$_3$)** δ 149.6, 143.9, 136.6, 134.9, 130.2, 129.6, 129.4, 128.4, 125.9 (2C), 125.6, 109.3, 52.5, 47.5, 29.9, 20.0. **HRMS** (ESI-TOF) *m/z*: $[M + H]^+$ for $C_{24}H_{25}N_2$ calculated 341.2012; found 341.2011.

1,4-Bis(di(indolin-5-yl)methyl)benzene (3aq). General Procedure D, purified by silica gel chromatography with EtOAc/hexane (30:70). Indoline (**2a**; 3 mmol, 12 equiv). Brown solid (115 mg, 80%; mp 167–168 °C). **^{1}H NMR (400 MHz, CDCl$_3$)** δ 7.04 (s, 4H), 6.91 (s, 4H), 6.79 (d, *J* = 8.0 Hz, A part of AB system, 4H), 6.58 (d, *J* = 8.0 Hz, B part of AB system, 4H), 5.32 (s, 2H), 3.55 (t, *J* = 8.3 Hz, 8H), 3.35 (bs, 4H), 2.98 (t, *J* = 8.3 Hz, 8H). **^{13}C{^{1}H} NMR (101 MHz, CDCl$_3$)** δ 149.3, 142.7, 135.8, 129.6, 129.0, 128.3, 125.7, 109.3, 55.6, 47.5, 29.9. **HRMS** (ESI-TOF) *m/z*: $[M + H]^+$ for $C_{40}H_{39}N_4$ calculated 575.3169; found 575.3167.

5,5'-(Naphthalen-2-ylmethylene)diindoline (3ar). General Procedure D, purified by silica gel chromatography with EtOAc/hexane (20:80). TfOH (2.2 μL, 25 μmol, 10 mol%) was used. Off-white solid (75 mg, 79%; mp 102–103 °C). **^{1}H NMR (400 MHz, CDCl$_3$)** δ 7.85 – 7.80 (m, 1H), 7.79 – 7.72 (m, *J* = 7.8 Hz, 2H), 7.52 (s, 1H), 7.47 – 7.42 (m, *J* = 7.5 Hz, 2H), 7.40 – 7.34 (m, 1H), 6.95 (s, 2H), 6.83 (d, *J* = 7.9 Hz, A part of AB system, 2H), 6.59 (d, *J* = 7.9 Hz, B part of AB system, 2H), 5.55 (s, 1H), 3.54 (t, *J* = 8.3 Hz, 4H), 2.98 (t, *J* = 8.3 Hz, 4H), (2H, not observed). **^{13}C{^{1}H} NMR (101 MHz, CDCl$_3$)** δ 149.9, 143.3, 135.2, 133.5, 132.1, 129.7, 128.5 (2C), 128.0, 127.7, 127.6, 127.5, 125.9, 125.9, 125.4, 109.3, 56.1, 47.6, 30.0. **HRMS** (ESI-TOF) *m/z*: $[M + H]^+$ for $C_{27}H_{25}N_2$ calculated 377.2012; found 377.2010.

5,5'-(Pyridin-2-ylmethylene)diindoline (3au$_2$). General Procedure D, purified by silica gel chromatography with EtOAc/hexane (20:80). Indoline (**2a**; 1.5 mmol, 2.5 equiv) and aldehyde (**2u$_2$**; 0.6 mmol, 1 equiv). Brown solid (159 mg, 81%; mp 126–127 °C). **^{1}H NMR (400 MHz, CDCl$_3$)** δ 8.59 – 8.56 (m, 1H), 7.62 – 7.55 (m, 1H), 7.13 – 7.05 (m, 2H), 6.92 (s, 2H), 6.79 (dd, *J* = 8.0, 1.3 Hz, A part of AB system, 2H), 6.56 (d, *J* = 8.0 Hz, B part of AB system, 2H), 5.50 (s, 1H), 3.52 (t, *J* = 8.3 Hz, 4H), 3.14 (bs, 2H), 2.96 (t, *J* = 8.3 Hz, 4H). **^{13}C{^{1}H} NMR (101 MHz, CDCl$_3$)** δ 164.8, 149.9,

149.3, 136.3, 134.0, 129.7, 128.2, 125.6, 123.7, 121.1, 109.3, 58.5, 47.5, 29.9. **HRMS** (ESI-TOF) *m/z*: $[M + H]^+$ for $C_{22}H_{22}N_3$ calculated 328.1808; found 328.1808.

5,5'-(Pyridin-3-ylmethylene)diindoline (3au$_3$). General Procedure D, purified by silica gel chromatography with EtOAc/hexane (30:70). Indoline (**2a**; 1.5 mmol, 2.5 equiv) and aldehyde (**2u$_3$**; 0.6 mmol, 1 equiv). Purple solid (165 mg, 84%; mp 116–117 °C). **^{1}H NMR (400 MHz, CDCl$_3$)** δ 8.47 – 8.41 (m, 2H), 7.42 (d, *J* = 7.9 Hz, 1H), 7.19 (dd, *J* = 7.9, 4.8 Hz, 1H), 6.85 (s, 2H), 6.73 (d, *J* = 8.0 Hz, A part of AB system, 2H), 6.55 (d, *J* = 8.0 Hz, B part of AB system, 2H), 5.34 (s, CH, 1H), 3.54 (t, *J* = 8.4 Hz, 4H), 3.30 (bs, 2H), 2.96 (t, *J* = 8.3 Hz, 4H). **^{13}C{^{1}H} NMR (101 MHz, CDCl$_3$)** δ 150.9, 150.2, 147.3, 141.1, 136.9, 134.1, 129.8, 128.3, 125.6, 123.2, 109.2, 53.5, 47.6, 29.9. **HRMS** (ESI-TOF) *m/z*: $[M + H]^+$ for $C_{22}H_{22}N_3$ calculated 328.1808; found 328.1808.

5,5'-(Pyridin-4-ylmethylene)diindoline (3au$_4$). General Procedure D, purified by silica gel chromatography with EtOAc/hexane (30:70). Indoline (**2a**; 1.5 mmol, 2.5 equiv) and aldehyde (**2u$_4$**; 0.6 mmol, 1 equiv). Yellow solid (177 mg, 90%; mp 106–107 °C). **^{1}H NMR (400 MHz, CDCl$_3$)** δ 8.59 – 8.36 (m, AA' part of AA'BB' system, 2H), 7.14 – 6.99 (m, BB' part of AA'BB' system, 2H), 6.83 (s, 2H), 6.72 (d, *J* = 8.0 Hz, A part of AB system, 2H), 6.56 (d, *J* = 8.0 Hz, B part of AB system, 2H), 5.29 (s, 1H), 3.54 (t, *J* = 8.4 Hz, 4H), 2.97 (t, *J* = 8.4 Hz, 4H), (2H, not observed). **^{13}C{^{1}H} NMR (101 MHz, CDCl$_3$)** δ 154.8, 150.3, 149.6, 133.4, 129.8, 128.3, 125.6, 124.8, 109.2, 55.4, 47.6, 29.9. **HRMS** (ESI-TOF) *m/z*: $[M + H]^+$ for $C_{22}H_{22}N_3$calculated 328.1808; found 328.1809.

5,5'-(Thien-2-ylmethylene)diindoline (3av$_2$). General Procedure D, purified by silica gel chromatography with EtOAc/hexane (20:80). Purple solid (63 mg, 76%; mp 102–103°C). **^{1}H NMR (400 MHz, CDCl$_3$)** δ 7.17 (dd, *J* = 5.1, 1.0 Hz, 1H), 6.97 (s, 2H), 6.92 (dd, *J* = 5.1, 3.5 Hz, 1H), 6.86 (d, *J* = 8.0 Hz, A part of AB system, 2H), 6.69 (d, *J* = 3.5 Hz, 1H), 6.57 (d, *J* = 8.0 Hz, B part of AB system, 2H), 5.48 (s, 1H), 3.54 (t, *J* = 8.3 Hz, 4H), 3.34 (bs, 2H), 2.98 (t, *J* = 8.3 Hz, 4H). **^{13}C{^{1}H} NMR (101 MHz, CDCl$_3$)** δ 150.3, 150.1, 135.3, 129.6, 127.7, 126.5, 125.8, 125.1, 124.1, 109.2, 51.3, 47.6, 30.0. **HRMS** (ESI-TOF) *m/z*: $[M + H]^+$ for $C_{21}H_{21}N_2S$ calculated 333.1420; found 333.1411.

5,5'-((1-Tosyl-1*H*-indol-3-yl)methylene)diindoline (3ay$_3$). General Procedure D, purified by silica gel chromatography with EtOAc/hexane (20:80). TfOH (2.2 µL, 25 µmol, 10 mol%) was used. Yellow solid (96 mg, 74%; mp 138–139 °C). **^{1}H NMR (400 MHz, CDCl$_3$)** δ 7.98 (d, *J* = 8.1 Hz, 1H), 7.71 (d, *J* = 8.1 Hz, 2H), 7.30 – 7.17 (m, 4H), 7.11 (d, *J* = 7.3 Hz, 1H), 7.00 (s, 1H), 6.91 (s, 2H), 6.79 (d, *J* = 7.9 Hz, A part of AB system, 2H), 6.56 (d, *J* = 7.9 Hz, B part of AB system, 2H), 5.34 (s, 1H), 3.56 (t, *J* = 8.4 Hz, 4H), 2.98 (t, *J* = 8.3 Hz, 4H), 2.37 (s, 3H), (2H, not observed). **^{13}C{^{1}H} NMR (101 MHz, CDCl$_3$)** δ 150.1, 144.6, 135.9, 135.2, 133.4, 130.9, 129.7, 128.6, 127.6, 126.8, 125.6, 125.1, 124.5, 123.1, 120.8, 113.8, 109.3, 47.5 (2C), 29.9, 21.6, (1 signal overlapped). **HRMS** (ESI-TOF) *m/z*: $[M + H]^+$ for $C_{32}H_{30}N_3O_2S$ calculated 520.2053; found 520.2050.

5,5'-((1-Tosyl-1*H*-indol-4-yl)methylene)diindoline (3ay$_4$). General Procedure D, purified by silica gel chromatography with EtOAc/hexane (20:80). TfOH (2.2 µL, 25 µmol, 10 mol%) was used. Yellow solid (109 mg, 84%; mp 128–129 °C). **^{1}H NMR (400 MHz, CDCl$_3$)** δ 7.85 (d, *J* = 8.3 Hz, 1H), 7.82 – 7.73 (m, AA' part of AA'BB' system, 2H), 7.46 (d, *J* = 3.8 Hz, 1H), 7.25 – 7.15 (m, *J* = 7.2 Hz, 3H), 6.91 – 6.80 (m, *J* = 6.8 Hz, 3H), 6.73 (dd, *J* = 8.0, 1.2 Hz, 2H), 6.62 – 6.46 (m, 3H), 5.63 (s, 1H), 3.59 (bs, 2H), 3.50 (t, *J* = 8.3 Hz, 4H), 2.92 (t, *J* = 8.3 Hz, 4H), 2.32 (s, 3H). **^{13}C{^{1}H} NMR (101 MHz, CDCl$_3$)** δ 149.9, 144.8, 138.7, 135.5, 134.8, 134.4, 130.3, 129.9, 129.6, 128.2, 126.9, 125.7, 125.6, 124.4, 123.6, 111.4, 109.2, 108.1, 53.3, 47.5, 29.9, 21.6. **HRMS** (ESI-TOF) *m/z*: [M + H]$^+$ for $C_{32}H_{30}N_3O_2S$ calculated 520.2053; found 520.2051.

5,5'-((1-Tosyl-1*H*-indol-5-yl)methylene)diindoline (3ay$_5$). General Procedure D, purified by silica gel chromatography with EtOAc/hexane (20:80). TfOH (2.2 µL, 25 µmol, 10 mol%) was used. Yellow solid (107 mg, 83%; mp 126–127 °C). **^{1}H NMR (400 MHz, CDCl$_3$)** δ 7.87 (d, *J* = 8.6 Hz, A part of AB system, 1H), 7.81 – 7.75 (m, AA' part of AA'BB' system, 2H), 7.51 (d, *J* = 3.7 Hz, 1H), 7.24 – 7.18 (m, 3H), 7.15 (dd, *J* = 8.6, 1.3 Hz, B part of AB system, 1H), 6.86 (s, 2H), 6.74 (d, *J* = 7.7 Hz, A part of AB system, 2H), 6.59 – 6.48 (m, 3H), 5.42 (s, 1H), 3.52 (t, *J* = 8.3 Hz, 4H), 2.94 (t, *J* = 8.3 Hz, 4H), 2.34 (s, 3H), (2H, not observed). **^{13}C{^{1}H} NMR (101 MHz, CDCl$_3$)** δ 149.8, 144.8, 140.8, 135.5 (2C), 133.2, 130.7, 129.9, 129.6, 128.3, 126.9, 126.6, 126.2, 125.7, 121.7, 113.0, 109.1 (2C), 55.7, 47.5, 29.9, 21.6. **HRMS** (ESI-TOF) *m/z*: [M + H]$^+$ for $C_{32}H_{30}N_3O_2S$ calculated 520.2053; found 520.2051.

5,5'-((1-Tosyl-1*H*-indol-6-yl)methylene)diindoline (3ay$_6$). General Procedure D, purified by silica gel chromatography with EtOAc/hexane (20:80). TfOH (2.2 µL, 25 µmol, 10 mol%) was used. Off-white solid (110 mg, 85%; mp 110–111°C). **^{1}H NMR (400 MHz, CDCl$_3$)** δ 7.74 (s, 1H), 7.62 – 7.55 (m, AA' part of AA'BB' system, 2H), 7.55 – 7.51 (m, 1H), 7.40 (d, *J* = 8.1 Hz, A part of AB system, 1H), 7.18 – 7.12 (m, BB' part of AA'BB' system, 2H), 7.08 (d, *J* = 8.1 Hz, B part of AB system, 1H), 6.91 (s, 2H), 6.78 (d, *J* = 7.9 Hz, A part of AB system, 2H), 6.63 – 6.55 (m, 3H), 5.49 (s, 1H), 3.81 – 3.68 (m, *J* = 3.8 Hz, 2H), 3.55 (t, *J* = 8.3 Hz, 4H), 2.98 (t, *J* = 8.3 Hz, 4H), 2.34 (s, 3H). **^{13}C{^{1}H} NMR (101 MHz, CDCl$_3$)** δ 149.8, 144.7, 142.6, 135.4, 134.9, 134.8, 129.8, 129.6, 128.9, 128.3, 127.1, 126.1, 125.8, 125.2, 120.8, 114.4, 109.2, 108.8, 56.1, 47.6, 30.0, 21.6. **HRMS** (ESI-TOF) *m/z*: [M + H]$^+$ for $C_{32}H_{30}N_3O_2S$ calculated 520.2053; found 520.2053.

5,5'-((4-Nitrophenyl)methylene)bis(4-chloroindoline) (3cj). General Procedure F, purified by silica gel chromatography with EtOAc/hexane (20:80). Yellow solid (138 mg, 79%; mp 117–118°C). **^{1}H NMR (400 MHz, CDCl$_3$)** δ 8.20 – 8.07 (m, AA' part of AA'BB' system, 2H), 7.31 – 7.16 (m, BB' part of AA'BB' system, 2H), 6.41 (d, *J* = 2.3 Hz, 4H), 6.10 (s, 1H), 3.62 (t, *J* = 8.5 Hz, 4H), 3.08 (t, *J* = 8.5 Hz, 4H). **^{13}C{^{1}H} NMR (101 MHz, CDCl$_3$)** δ 151.8, 151.5, 146.6, 131.1, 130.4, 129.8, 129.4,

128.9, 123.6, 107.0, 49.3, 47.1, 29.9; **HRMS** (APCI-TOF) *m/z*: $[M + H]^+$ for $C_{23}H_{20}Cl_2N_3O_2$ calculated 440.0927; found 440.0927.

5,5'-((4-Nitrophenyl)methylene)bis(6-bromoindoline) (3gj). General Procedure F, purified by silica gel chromatography with EtOAc/hexane (20:80). Yellow solid (157 mg, 75%; mp 103–104°C). **^{1}H NMR (400 MHz, CDCl$_3$)** δ 8.23 – 8.03 (m, AA' part of AA'BB' system, 2H), 7.25 – 7.14 (m, BB' part of AA'BB' system, 2H), 6.84 (s, 2H), 6.47 (s, 2H), 6.07 (s, 1H), 3.84 (bs, 2H), 3.56 (t, *J* = 8.5 Hz, 4H), 2.90 (t, *J* = 8.5 Hz, 4H). **^{13}C{^{1}H} NMR (101 MHz, CDCl$_3$)** δ 151.9, 151.5, 146.6, 130.7, 130.5, 129.1, 126.5, 123.9, 123.6, 113.5, 54.9, 47.8, 29.5; **HRMS** (APCI-TOF) *m/z*: $[M + H]^+$ for $C_{23}H_{20}Br_2N_3O_2$ calculated 527.9917; found 527.9918.

5,5'-((4-Nitrophenyl)methylene)bis(6-methoxyindoline) (3jj). General Procedure F, purified by silica gel chromatography with EtOAc/hexane (20:80). Yellow solid (131 mg, 76%; mp 113–114°C). **^{1}H NMR (400 MHz, CDCl$_3$)** δ 8.20 – 7.93 (m, AA' part of AA'BB' system, 2H), 7.25 – 7.16 (m, BB' part of AA'BB' system, 2H), 6.51 (s, 2H), 6.28 (s, 2H), 6.03 (s, 1H), 3.62 (s, 6H), 3.53 (t, *J* = 8.4 Hz, 4H), 2.89 (t, *J* = 8.4 Hz, 4H). **^{13}C{^{1}H} NMR (101 MHz, CDCl$_3$)** δ 157.0, 154.9, 151.4, 145.9, 129.8, 125.9, 123.1, 121.6, 120.5, 94.4, 56.0, 48.0, 43.0, 29.5; **HRMS** (APCI-TOF) *m/z*: $[M + H]^+$ for $C_{25}H_{26}N_3O_4$ calculated 432.1918; found 432.1917.

5,5'-((4-Nitrophenyl)methylene)bis(7-methylindoline) (3nj). General Procedure F, purified by silica gel chromatography with EtOAc/hexane (20:80). Yellow solid (115 mg, 72%; mp 91–92°C). **^{1}H NMR (400 MHz, CDCl$_3$)** δ 8.17 – 8.07 (m, AA' part of AA'BB' system, 2H), 7.42 – 7.25 (m, BB' part of AA'BB' system, 2H), 6.68 (s, 2H), 6.58 (s, 2H), 5.40 (s, 1H), 3.57 (t, *J* = 8.4 Hz, 4H), 3.00 (t, *J* = 8.4 Hz, 4H), 2.08 (s, 6H). **^{13}C{^{1}H} NMR (101 MHz, CDCl$_3$)** δ 153.9, 148.9, 146.2, 134.0, 130.3, 129.3, 129.2, 123.5, 123.2, 118.9, 55.9, 47.5, 30.3, 17.1; **HRMS** (APCI-TOF) *m/z*: $[M + H]^+$ for $C_{25}H_{26}N_3O_2$ calculated 400.2020; found 400.2017.

1,1'-((4-Bromophenyl)methylene)diindoline (4af). Indoline (**2a**; 120 mg, 113 µL, 1 mmol, 2.1 equiv) and 4-bromobenzaldehyde (**11f**; 93 mg, 0.50 mmol, 1 equiv) were stirred in 1 mL HFIP at room temperature for 5 hours. The solvent was then removed under vacuum, and NMR was recorded. **^{1}H NMR (400 MHz, CDCl$_3$)** δ 7.55 – 7.45 (m, AA' part of AA'BB' system, 2H), 7.42 – 7.34 (m, BB' part of AA'BB' system, 2H), 7.08 (d, *J* = 7.5 Hz, A part of AB system, 2H), 6.97 (t, *J* = 7.4 Hz, 2H), 6.66 (t, *J* = 7.4 Hz, 2H), 6.31 (d, *J* = 7.4 Hz, B part of AB system, 2H), 5.80 (s, 1H), 3.46 – 3.38 (m, 4H), 2.99 (t, *J* = 8.4 Hz, 4H). **^{13}C{^{1}H} NMR (101 MHz, CDCl$_3$)** δ 150.6, 137.3, 131.9, 130.0, 129.6, 127.4, 124.8, 122.0, 118.1, 107.9, 72.4, 49.7, 28.3. **HRMS** (ESI-TOF) *m/z*: $[M - H]^+$ for $C_{23}H_{20}BrN_2$ calculated 403.0815; found 403.0803.

4,5-Di(indolin-1-yl)cyclopent-2-en-1-one (8). General Procedure D, purified by silica gel chromatography with EtOAc/hexane (20:80). Indoline (**2a**; 1 mmol, 2.5 equiv), aldehyde (**11x$_2$**; 0.4 mmol, 1 equiv), and TfOH (0.1 mmol, 3.56 µL, 40 µmol). Yellow oil (67 mg, 85%). **^{1}H NMR (400 MHz, $CDCl_3$)** δ 7.71 (dd, *J* = 6.2, 1.9 Hz, A part of AB system, 1H), 7.10 (d, *J* = 7.6 Hz, 1H), 7.07 (d, *J* = 7.6 Hz, 1H), 6.97 (t, *J* = 7.6 Hz, 1H), 6.92 (t, *J* = 7.6 Hz, 1H), 6.74 – 6.60 (m, 2H), 6.52 (dd, *J* = 6.2, 1.9 Hz, B part of AB system, 1H), 6.36 (d, *J* = 7.6 Hz, 1H), 6.08 (d, *J* = 7.6 Hz, 1H), 5.04 – 4.98 (m, 1H), 4.41 (d, *J* = 3.6 Hz, 1H), 3.57 – 3.48 (m, 2H), 3.45 – 3.31 (m, 2H), 3.00 (t, *J* = 8.4 Hz, 4H). **$^{13}C\{^1H\}$ NMR (101 MHz, $CDCl_3$)** δ 202.2, 161.6, 149.7, 149.5, 134.9, 130.0, 129.7, 127.2, 127.0, 124.7, 124.6, 118.3, 117.9, 107.2, 106.4, 63.0, 58.3, 51.0, 49.4, 28.3, 28.2. Spectroscopic data for the title compound were consistent with those reported in the literature.[26]

4-Methoxy-1-(4-nitrobenzyl)indoline (9ej). General Procedure F, purified by silica gel chromatography with EtOAc/hexane (20:80). Brown solid (58 mg, 51%; mp 132–133°C). **^{1}H NMR (400 MHz, $CDCl_3$)** δ 8.27 – 8.11 (m, AA' part of AA'BB' system, 2H), 7.68 – 7.50 (m, BB' part of AA'BB' system, 2H), 7.04 (t, *J* = 8.0 Hz, 1H), 6.33 (d, *J* = 8.0 Hz, A part of AB system, 1H), 6.12 (d, *J* = 8.0 Hz, B part of AB system, 1H). 4.33 (s, 2H), 3.83 (s, 3H), 3.38 (t, *J* = 8.4 Hz, 2H), 2.98 (t, *J* = 8.4 Hz, 2H). **$^{13}C\{^1H\}$ NMR (101 MHz, $CDCl_3$)** δ 156.4, 153.8, 147.3, 146.7, 129.0, 128.4, 123.9, 115.9, 101.9, 101.0, 55.4, 54.3, 53.5, 25.7; **HRMS** (APCI-TOF) *m/z*: $[M + H]^+$ for $C_{16}H_{17}N_2O_3$ calculated 285.1234; found 285.1233.

6-Iodo-1-(4-nitrobenzyl)indoline (9hj). General Procedure F, purified by silica gel chromatography with EtOAc/hexane (20:80). Brown oil (69 mg, 46%). **^{1}H NMR (400 MHz, $CDCl_3$)** δ 8.28 – 8.10 (m, AA' part of AA'BB' system, 2H), 7.57 – 7.46 (m, BB' part of AA'BB' system, 2H), 7.01 (dd, *J* = 7.6, 1.3 Hz, A part of AB system, 1H), 6.84 (d, *J* = 7.6 Hz, B part of AB system, 1H). 6.71 (d, *J* = 1.3 Hz, 1H), 4.32 (s, 2H), 3.37 (t, *J* = 8.4 Hz, 2H), 2.98 (t, *J* = 8.4 Hz, 2H). **$^{13}C\{^1H\}$ NMR (101 MHz, $CDCl_3$)** δ 153.5, 147.4, 145.8, 129.8, 128.3, 127.2, 126.4, 124.0, 115.7, 92.4, 53.9, 52.9, 28.3; **HRMS** (APCI-TOF) *m/z*: $[M + H]^+$ for $C_{15}H_{14}IN_2O_2$ calculated 381.0094; found 381.0095.

6-Methyl-1-(4-nitrobenzyl)indoline (9ij). General Procedure F, purified by silica gel chromatography with EtOAc/hexane (20:80). Brown solid (60 mg, 56%; mp 103–104°C). **^{1}H NMR (400 MHz, $CDCl_3$)** δ 8.33 – 8.13 (m, AA' part of AA'BB' system, 2H), 7.67 – 7.50 (m, BB' part of AA'BB' system, 2H), 7.06 (d, *J* = 7.4 Hz, A part of AB system, 1H), 6.59 (d, *J* = 7.4 Hz, B part of AB system, 1H). 6.31 (s, 1H), 4.37 (s, 2H), 3.40 (t, *J* = 8.2 Hz, 2H), 3.03 (t, *J* = 8.2 Hz, 2H), 2.31 (s, 3H). **$^{13}C\{^1H\}$ NMR (101 MHz, $CDCl_3$)** δ 152.3, 147.2, 146.8, 137.3, 128.3, 127.0, 124.4, 123.8, 119.0, 108.0, 54.4, 53.4, 28.3, 21.8; **HRMS** (APCI-TOF) *m/z*: $[M + H]^+$ for $C_{16}H_{17}N_2O_2$ calculated 269.1285; found 269.1295.

1-(4-Nitrobenzyl)indoline-7-carbonitrile (9mj). General Procedure F, purified by silica gel chromatography with EtOAc/hexane (20:80). Yellow solid (64 mg, 58%; mp 136–137°C). **^{1}H NMR (400 MHz, CDCl$_3$)** δ 8.34 – 8.14 (m, AA' part of AA'BB' system, 2H), 7.62 – 7.41 (m, BB' part of AA'BB' system, 2H), 7.20 (d, *J* = 7.6 Hz, 2H), 6.65 (t, *J* = 7.6 Hz, 1H), 4.94 (s, 2H), 3.55 (t, *J* = 8.8 Hz, 2H), 3.06 (t, *J* = 8.8 Hz, 2H). **^{13}C{^{1}H} NMR (101 MHz, CDCl$_3$)** δ 152.6, 147.5, 145.4, 132.0, 131.9, 128.9, 128.4, 124.2, 119.0, 117.9, 88.9, 53.4, 51.8, 27.4; **HRMS** (APCI-TOF) *m/z*: $[M + H]^+$ for $C_{16}H_{14}N_3O_2$ calculated 280.1081; found 280.1082.

5,5'-((4-Nitrophenyl)methylene)bis(1-benzyl-4-bromoindoline) (11bj). General Procedure G, purified by silica gel chromatography with EtOAc/hexane (10:90). Yellow solid (212 mg, 80%; mp 185–186°C). **^{1}H NMR (400 MHz, CDCl$_3$)** δ 8.19 – 8.08 (m, AA' part of AA'BB' system, 2H), 7.42 – 7.26 (m, 10H), 7.25 – 7.21 (m, BB' part of AA'BB' system, 2H), 6.44 (d, *J* = 8.2 Hz, A part of AB system, 2H), 6.30 (d, *J* = 8.2 Hz, B part of AB system, 2H), 6.09 (s, 1H), 4.24 (s, 4H), 3.42 (t, *J* = 8.4 Hz, 4H), 3.02 (t, *J* = 8.4 Hz, 4H). **^{13}C{^{1}H} NMR (101 MHz, CDCl$_3$)** δ 152.3, 151.8, 146.5, 137.9, 131.7, 130.5, 130.1, 130.0, 128.7, 127.9, 127.5, 123.6, 122.8, 105.1, 54.1, 53.5, 52.7, 30.8; **HRMS** (APCI-TOF) *m/z*: $[M + H]^+$ for $C_{37}H_{32}Br_2N_3O_2$ calculated 708.0856; found 708.0851.

5,5'-((4-Nitrophenyl)methylene)bis(1-benzyl-4-chloroindoline) (11cj). General Procedure G, purified by silica gel chromatography with EtOAc/hexane (10:90). Yellow solid (202 mg, 88%; mp 104–105°C). **^{1}H NMR (400 MHz, CDCl$_3$)** δ 8.18 – 8.08 (m, AA' part of AA'BB' system, 2H), 7.38 – 7.26 (m, 10H), 7.26 – 7.22 (m, BB' part of AA'BB' system, 2H), 6.46 (d, *J* = 8.1 Hz, A part of AB system, 2H), 6.26 (d, *J* = 8.1 Hz, B part of AB system, 2H), 6.12 (s, 1H), 4.23 (s, 4H), 3.42 (t, *J* = 8.4 Hz, 4H), 3.03 (t, *J* = 8.4 Hz, 4H). **^{13}C{^{1}H} NMR (101 MHz, CDCl$_3$)** δ 152.7, 151.7, 146.5, 138.0, 130.9, 130.4, 130.0, 129.2, 128.7, 128.5, 128.0, 127.5, 123.6, 104.6, 53.5, 53.2, 49.1, 28.5; **HRMS** (APCI-TOF) *m/z*: $[M + H]^+$ for $C_{37}H_{32}Cl_2N_3O_2$ calculated 620.1866; found 620.1867.

5,5'-((4-Nitrophenyl)methylene)bis(1-benzyl-4-methylindoline) (11dj). General Procedure G, purified by silica gel chromatography with EtOAc/hexane (10:90). Yellow solid (167 mg, 78%; mp 106–107°C). **^{1}H NMR (400 MHz, CDCl$_3$)** δ 8.07 – 7.92 (m, AA' part of AA'BB' system, 2H), 7.31 – 7.20 (m, 8H), 7.20 – 7.10 (m, 4H), 6.29 (d, *J* = 8.1 Hz, A part of AB system, 2H), 6.16 (d, *J* = 8.1 Hz, B part of AB system, 2H), 5.54 (s, 1H), 4.14 (d, *J* = 14.7 Hz, A part of AB system, 2H), 4.10 (d, *J* = 14.7 Hz, B part of AB system, 2H), 3.30 – 3.19 (m, 4H), 2.82 (t, *J* = 8.2 Hz, 4H), 1.92 (s, 6H). **^{13}C{^{1}H} NMR (101 MHz, CDCl$_3$)** δ 153.4, 151.2, 146.3, 138.7, 132.5, 131.2, 130.6, 129.7, 128.7, 128.6, 128.1, 127.2, 123.5, 104.3, 54.0, 53.6, 49.5, 27.9, 15.6; **HRMS** (APCI-TOF) *m/z*: $[M + H]^+$ for $C_{39}H_{38}N_3O_2$ calculated 580.2959; found 580.2958.

5,5'-((4-Nitrophenyl)methylene)bis(1-benzyl-4-methoxyindoline) (11ej). General Procedure G, purified by silica gel chromatography with EtOAc/hexane (10:90). Yellow solid (183 mg, 81%; mp

149–150°C). **^{1}H NMR (400 MHz, CDCl$_3$)** δ 8.17 – 8.07 (m, AA' part of AA'BB' system, 2H), 7.46 – 7.17 (m, 12H), 6.54 (d, *J* = 8.1 Hz, A part of AB system, 2H), 6.21 (d, *J* = 8.1 Hz, B part of AB system, 2H), 6.11 (s, 1H), 4.25 (d, *J* = 14.7 Hz, A part of AB system, 2H), 4.20 (d, *J* = 14.7 Hz, B part of AB system, 2H),3.61 (s, 6H), 3.43 – 3.29 (m, 4H), 3.06 (t, *J* = 8.2 Hz, 4H). **^{13}C{^{1}H} NMR (101 MHz, CDCl$_3$)** δ 154.5, 154.3, 153.9, 146.1, 138.4, 130.0, 129.6, 128.6, 128.1, 127.3, 125.5, 123.3, 120.2, 102.5, 59.6, 54.1, 54.0, 43.2, 26.7; **HRMS** (APCI-TOF) *m/z*: $[M + H]^+$ for $C_{39}H_{38}N_3O_4$calculated 612.2857; found 612.2857.

5,5'-((4-Nitrophenyl)methylene)bis(1-benzyl-6-bromoindoline) (11gj). General Procedure G, purified by silica gel chromatography with EtOAc/hexane (10:90). Yellow solid (223 mg, 84%; mp 109–110°C). **^{1}H NMR (400 MHz, CDCl$_3$)** δ 8.13 – 7.92 (m, AA' part of AA'BB' system, 2H), 7.33 – 7.17 (m, 10H), 7.15 – 7.02 (m, BB' part of AA'BB' system, 2H), 6.63 (s, 2H), 6.39 (s, 2H), 6.00 (s, 1H), 4.13 (s, 4H), 3.24 (t, *J* = 8.5 Hz, 4H), 2.76 (t, *J* = 8.4 Hz, 4H). **^{13}C{^{1}H} NMR (101 MHz, CDCl$_3$)** δ 152.6, 151.7, 146.5, 137.8, 130.5, 129.7, 129.6, 128.7, 128.0, 127.5, 126.3, 124.2, 123.6, 111.1, 54.8, 53.7, 53.4, 28.3; **HRMS** (APCI-TOF) *m/z*: $[M + H]^+$ for $C_{37}H_{32}Br_2N_3O_2$ calculated 708.0856; found 708.0852.

5,5'-((4-Nitrophenyl)methylene)bis(1-benzyl-6-iodoindoline) (11hj). General Procedure G, purified by silica gel chromatography with EtOAc/hexane (10:90). Brown solid (248 mg, 82%; mp 110–111°C). **^{1}H NMR (400 MHz, CDCl$_3$)** δ 8.28 – 8.08 (m, AA' part of AA'BB' system, 2H), 7.51 – 7.29 (m, 10H), 7.29 – 7.18 (m, BB' part of AA'BB' system, 2H), 7.05 (s, 2H), 6.49 (s, 2H), 5.85 (s, 1H), 4.24 (s, 4H), 3.33 (t, *J* = 8.7 Hz, 4H), 2.87 (t, *J* = 8.7 Hz, 4H). **^{13}C{^{1}H} NMR (101 MHz, CDCl$_3$)** δ 152.6, 151.8, 146.4, 137.8, 133.0, 130.8, 130.8, 128.7, 128.0, 127.4, 126.2, 123.6, 117.6, 101.1, 63.9, 53.6, 53.3, 28.3; **HRMS** (APCI-TOF) *m/z*: $[M + H]^+$ for $C_{37}H_{32}I_2N_3O_2$ calculated 804.0578; found 804.0578.

5,5'-((4-Nitrophenyl)methylene)bis(1-benzyl-6-methylindoline) (11ij). General Procedure G, purified by silica gel chromatography with EtOAc/hexane (10:90). Orange solid (184 mg, 86%; mp 108–109°C). **^{1}H NMR (400 MHz, CDCl$_3$)** δ 8.11 – 7.93 (m, AA' part of AA'BB' system, 2H), 7.35 – 7.08 (m, 12H), 6.36 (s, 2H), 6.31 (s, 2H), 5.52 (s, 1H), 4.18 (d, *J* = 14.7 Hz, A part of AB system, 2H), 4.11 (d, *J* = 14.7 Hz, B part of AB system, 2H), 3.27 – 3.07 (m, 4H), 2.75 (t, *J* = 8.2 Hz, 4H), 2.01 (s, 6H). **^{13}C{^{1}H} NMR (101 MHz, CDCl$_3$)** δ 153.3, 151.4, 146.3, 138.7, 135.4, 130.6, 130.5, 128.6, 128.1, 127.6, 127.3, 125.4, 123.5, 109.5, 54.2, 54.1, 49.5, 28.5, 20.3. **HRMS** (APCI-TOF) *m/z*: $[M + H]^+$ for $C_{39}H_{38}N_3O_2$ calculated 580.2958; found 580.2959.

5,5'-((4-Nitrophenyl)methylene)bis(1-benzyl-6-methoxyindoline) (11jj). General Procedure G, purified by silica gel chromatography with EtOAc/hexane (10:90). Yellow solid (192 mg, 85%; mp 106–107°C). **^{1}H NMR (400 MHz, CDCl$_3$)** δ 8.17 – 7.97 (m, AA' part of AA'BB' system, 2H), 7.42 –

7.32 (m, 8H), 7.32 – 7.21 (m, 4H), 6.53 (s, 2H), 6.16 (s, 2H), 6.04 (s, 1H), 4.28 (d, *J* = 14.7 Hz, A part of AB system, 2H), 4.23 (d, *J* = 14.7 Hz, B part of AB system, 2H), 3.62 (s, 6H), 3.43 – 3.18 (m, 4H), 2.84 (t, *J* = 8.2 Hz, 4H). **$^{13}C\{^1H\}$ NMR (101 MHz, $CDCl_3$)** δ 157.2, 155.2, 152.5, 145.9, 138.6, 129.8, 128.6, 128.1, 127.3, 126.0, 123.2, 121.2, 120.4, 92.6, 56.1, 54.4, 54.2, 42.9, 28.2. **HRMS** (APCI-TOF) *m/z*: $[M + H]^+$ for $C_{39}H_{38}N_3O_4$ calculated 612.2857; found 612.2856.

5,5'-((4-Nitrophenyl)methylene)bis(1-benzylindoline-6-ol) (11kj). General Procedure G, purified by silica gel chromatography with EtOAc/hexane (10:90). Yellow oily substance (78 mg, 83%). **^1H NMR (400 MHz, $CDCl_3$)** δ 8.15 – 8.08 (m, AA' part of AA'BB' system, 2H), 7.36 – 7.25 (m, 12H), 6.54 (s, 2H), 6.01 (s, 2H), 5.77 (s, 1H), 5.44 (bs, 2H), 4.15 (s, 4H), 3.28 (t, *J* = 7.9 Hz, 4H), 2.80 (t, *J* = 7.9 Hz, 4H). **$^{13}C\{^1H\}$ NMR (101 MHz, $CDCl_3$)** δ 153.0, 152.7, 152.2, 146.2, 138.1, 130.1, 128.6, 128.1, 127.3, 125.3, 123.3, 122.6, 117.3, 96.5, 53.9, 53.7, 43.5, 27.9. **HRMS** (APCI-TOF) *m/z*: $[M + H]^+$ for $C_{37}H_{34}N_3O_4$ calculated 584.2544; found 584.2542.

5,5'-((4-Nitrophenyl)methylene)bis(1-benzyl-7-methylindoline) (11nj). General Procedure G (at 80°C), purified by silica gel chromatography with EtOAc/hexane (10:90). Brown oily substance (154 mg, 72%). **^1H NMR (400 MHz, $CDCl_3$)** δ 8.16 – 7.97 (m, AA' part of AA'BB' system, 2H), 7.37 – 7.02 (m, 12H), 6.59 (s, 2H), 6.52 (s, 2H), 5.30 (s, 1H), 4.35 (s, 4H), 3.24 (t, *J* = 8.6 Hz, 4H), 2.80 (t, *J* = 8.6 Hz, 4H), 2.22 (s, 6H). **$^{13}C\{^1H\}$ NMR (101 MHz, $CDCl_3$)** δ 153.5, 149.4, 146.2, 139.7, 134.1, 131.7, 130.2 (2C), 128.5, 127.7, 127.1, 123.4, 123.4, 119.9, 56.4, 55.5, 54.2, 28.9, 19.5; **HRMS** (APCI-TOF) *m/z*: $[M + H]^+$ for $C_{39}H_{38}N_3O_2$ calculated 580.2958; found 580.2959.

5,5'-((4-Nitrophenyl)methylene)bis(1-benzyl-7-methoxyindoline) (11oj). General Procedure G (at 80°C), purified by silica gel chromatography with EtOAc/hexane (10:90). Yellow oily substance (151 mg, 67%). **^1H NMR (400 MHz, $CDCl_3$)** δ 8.20 – 7.96 (m, AA' part of AA'BB' system, 2H), 7.37 – 7.12 (m, 12H), 6.39 (s, 2H), 6.34 (s, 2H), 5.36 (s, 1H), 4.54 (s, 4H), 3.64 (s, 6H), 3.15 (t, *J* = 8.6 Hz, 4H), 2.78 (t, *J* = 8.6 Hz, 4H**). $^{13}C\{^1H\}$ NMR (101 MHz, $CDCl_3$)** δ 153.4, 146.4, 146.3, 139.5, 132.4, 130.3, 128.5 (2C), 128.4, 127.0, 126.5, 123.5, 118.7, 112.7, 55.9, 55.8, 54.0, 29.8, 29.3; **HRMS** (APCI-TOF) *m/z*: $[M + H]^+$ for $C_{39}H_{38}N_3O_4$ calculated 612.2857; found 612.2856.

6,6'-(Phenylmethylene)bis(1,2,3,4-tetrahydroquinoline) (6aa). General Procedure E, purified by silica gel chromatography with EtOAc/hexane (10:90). Green solid (108 mg, 76%; 66%, using DCE as solvent; mp 115–116 °C). **^1H NMR (400 MHz, $CDCl_3$)** δ 7.37 – 7.29 (m, 3H), 7.25 – 7.19 (m, 2H), 6.79 (s, 2H), 6.77 (d, *J* = 8.2 Hz, B part of AB system, 2H), 6.47 (d, *J* = 8.2 Hz, B part of AB system, 2H), 5.32 (s, 1H), 3.82 (bs, 2H), 3.34 (t, *J* = 6.4 Hz, 4H), 2.76 (t, *J* = 6.4 Hz, 4H), 1.99 (p, *J* = 6.4 Hz, 4H). **$^{13}C\{^1H\}$ NMR (101 MHz, $CDCl_3$)** δ 145.7, 143.0, 133.5, 130.5, 129.5, 128.1, 127.8, 125.8, 121.4, 114.1, 55.5, 42.2, 27.1, 22.4. **HRMS** (ESI-TOF) *m/z*: $[M + H]^+$ for $C_{25}H_{27}N_2$ calculated 355.2169; found 355.2169.

6,6'-(p-Tolylmethylene)bis(1,2,3,4-tetrahydroquinoline) (6ab). General Procedure E, purified by silica gel chromatography with EtOAc/hexane (10:90). Green solid (114 mg, 77%; 71%, using DCE as solvent; mp 94–95 °C). **^{1}H NMR (400 MHz, $CDCl_3$)** δ 7.10 – 7.05 (m, AA' part of AA'BB' system, 2H), 7.05 – 7.01 (m, BB' part of AA'BB' system, 2H), 6.72 (s, 2H), 6.69 (d, *J* = 8.2 Hz, A part of AB system, 2H), 6.39 (d, *J* = 8.1 Hz, B part of AB system, 2H), 5.22 (s, 1H), 3.65 (bs, 2H), 3.27 (t, *J* = 6.4 Hz, 4H), 2.69 (t, *J* = 6.4 Hz, 4H), 2.32 (s, 3H), 1.92 (p, *J* = 6.4 Hz, 4H). **^{13}C{^{1}H} NMR (101 MHz, $CDCl_3$)** δ 142.9, 142.7, 135.2, 133.7, 130.5, 129.3, 128.8, 127.7, 121.3, 114.2, 55.1, 42.2, 27.1, 22.4, 21.2. **HRMS** (ESI-TOF) *m/z*: $[M + H]^+$ for $C_{26}H_{29}N_2$ calculated 369.2325; found 369.2326.

6,6'-((4-(*tert*-Butyl)phenyl)methylene)bis(1,2,3,4-tetrahydroquinoline) (6ac). General Procedure E, purified by silica gel chromatography with EtOAc/hexane (10:90). Green solid (130 mg, 79%; 75%, using DCE as solvent; mp 96–97 °C). **^{1}H NMR (400 MHz, $CDCl_3$)** δ 7.33 – 7.26 (m, AA' part of AA'BB' system, 2H), 7.12 – 7.06 (m, BB' part of AA'BB' system, 2H), 6.76 (s, 2H), 6.73 (d, *J* = 8.2 Hz, A part of AB system, 2H), 6.41 (d, *J* = 8.1 Hz, B part of AB system, 2H), 5.23 (s, 1H), 3.67 (bs, 2H), 3.28 (t, *J* = 6.4 Hz, 4H), 2.71 (t, *J* = 6.4 Hz, 4H), 1.93 (p, *J* = 6.4 Hz, 4H), 1.32 (s, 9H). **^{13}C{^{1}H} NMR (101 MHz, $CDCl_3$)** δ 148.3, 142.8, 142.4, 133.7, 130.4, 128.9, 127.6, 124.9, 121.2, 114.1, 55.0, 42.1, 34.4, 31.5, 27.0, 22.4. **HRMS** (ESI-TOF) *m/z*: $[M + H]^+$ for $C_{29}H_{35}N_2$ calculated 411.2795; found 411.2795.

6,6'-((4-Fluorophenyl)methylene)bis(1,2,3,4-tetrahydroquinoline) (6ad). General Procedure E (for 7 hours), purified by silica gel chromatography with EtOAc/hexane (10:90). Green solid (122 mg, 82%; 73%, using DCE as solvent; mp 87–88°C). **^{1}H NMR (400 MHz, $CDCl_3$)** δ 7.14 – 7.06 (m, AA' part of AA'BB' system, 2H), 6.99 – 6.91 (m, BB' part of AA'BB' system, 2H), 6.76 – 6.63 (m, 4H), 6.40 (d, *J* = 7.9 Hz, A part of AB system, 2H), 5.24 (s, 1H), 3.64 (bs, 2H), 3.28 (t, *J* = 6.4 Hz, 4H), 2.70 (t, *J* = 6.4 Hz, 4H), 1.93 (p, *J* = 6.4 Hz, 4H). **^{13}C{^{1}H} NMR (101 MHz, $CDCl_3$)** δ 161.2 (d, *J* = 243.6 Hz), 143.0, 141.4 (d, *J* = 3.1 Hz), 133.2, 130.8 (d, *J* = 7.7 Hz), 130.4, 127.6, 121.4, 114.8 (d, *J* = 20.9 Hz), 114.2, 54.7, 42.2, 27.1, 22.4. **HRMS** (ESI-TOF) *m/z*: $[M + H]^+$ for $C_{25}H_{26}FN_2$ calculated 373.2075; found 373.2075.

6,6'-((4-Chlorophenyl)methylene)bis(1,2,3,4-tetrahydroquinoline) (6ae). General Procedure E, purified by silica gel chromatography with EtOAc/hexane (10:90). Green solid (125 mg, 80%; 76%, using DCE as solvent; mp 94–95 °C). **^{1}H NMR (400 MHz, $CDCl_3$)** δ 7.25 – 7.20 (m, AA' part of AA'BB' system, 2H), 7.10 – 7.05 (m, BB' part of AA'BB' system, 2H), 6.72 – 6.64 (m, 4H), 6.40 (d, *J* = 7.9 Hz, B part of AB system, 2H), 5.22 (s, 1H), 3.69 (bs, 2H), 3.28 (t, *J* = 6.3 Hz, 4H), 2.69 (t, *J* = 6.3 Hz, 4H), 1.92 (t, *J* = 6.3 Hz, 4H). **^{13}C{^{1}H} NMR (101 MHz, $CDCl_3$)** δ 144.3, 143.1, 132.8, 131.5, 130.8, 130.4, 128.2, 127.6, 121.4, 114.2, 54.8, 42.14, 27.1, 22.3. **HRMS** (ESI-TOF) *m/z*: $[M + H]^+$ for $C_{25}H_{26}ClN_2$ calculated 389.1779; found 389.1780.

6,6'-((4-Bromophenyl)methylene)bis(1,2,3,4-tetrahydroquinoline) (6af). General Procedure E, purified by silica gel chromatography with EtOAc/hexane (10:90). Green solid (155 mg, 89%; 85%, using DCE as solvent; mp 100–101°C). **^{1}H NMR (400 MHz, CDCl$_3$)** δ 7.41 – 7.33 (m, AA' part of AA'BB' system, 2H), 7.04 – 6.98 (m, BB' part of AA'BB' system, 2H), 6.70 – 6.62 (m, 4H), 6.39 (d, *J* = 7.9 Hz, B part of AB system, 2H), 5.18 (s, 1H), 3.73 (bs, 2H), 3.27 (t, *J* = 6.4 Hz, 4H), 2.68 (t, *J* = 6.4 Hz, 4H), 1.91 (p, *J* = 6.4 Hz, 4H). **^{13}C{^{1}H} NMR (101 MHz, CDCl$_3$)** δ 144.8, 143.0, 132.9, 131.3, 131.2, 130.4, 127.7, 121.5, 119.7, 114.3, 54.9, 42.1, 27.1, 22.3. **HRMS** (ESI-TOF) *m/z*: $[M + H]^+$ for $C_{25}H_{26}BrN_2$ calculated 433.1274; found 433.1273.

6,6'-((4-Iodophenyl)methylene)bis(1,2,3,4-tetrahydroquinoline) (6ag). General Procedure E, purified by silica gel chromatography with EtOAc/hexane (10:90). Green solid (168 mg, 87%; mp 88–89°C). **^{1}H NMR (400 MHz, CDCl$_3$)** δ 7.59 – 7.52 (m, AA' part of AA'BB' system, 2H), 6.92 – 6.87 (m, BB' part of AA'BB' system, 2H), 6.71 – 6.60 (m, 4H), 6.39 (d, *J* = 8.0 Hz, B part of AB system, 2H), 5.18 (s, 1H), 3.70 (bs, 2H), 3.27 (t, *J* = 6.4 Hz, 4H), 2.69 (t, *J* = 6.4 Hz, 4H), 1.92 (t, *J* = 6.4 Hz, 4H). **^{13}C{^{1}H} NMR (101 MHz, CDCl$_3$)** δ 145.6, 143.1, 137.1, 132.7, 131.6, 130.4, 127.7, 121.4, 114.2, 91.2, 55.0, 42.2, 27.1, 22.3. **HRMS** (ESI-TOF) *m/z*: $[M + H]^+$ for $C_{25}H_{26}IN_2$ calculated 481.1135; found 481.1136.

6,6'-((4-Methoxyphenyl)methylene)bis(1,2,3,4-tetrahydroquinoline) (6ah). General Procedure E, purified by silica gel chromatography with EtOAc/hexane (10:90). Green solid (122 mg, 79%; mp 98–99°C). **^{1}H NMR (400 MHz, CDCl$_3$)** δ 7.09 – 7.01 (m, AA' part of AA'BB' system, 2H), 6.84 – 6.78 (m, BB' part of AA'BB' system, 2H), 6.73 – 6.65 (m, 4H), 6.39 (d, *J* = 8.0 Hz, B part of AB system, 2H), 5.20 (s, 1H), 3.78 (s, 3H), 3.27 (t, *J* = 6.4 Hz, 4H), 2.69 (t, *J* = 6.4 Hz, 4H), 1.92 (p, *J* = 6.4 Hz, 4H), (2H, not observed). **^{13}C{^{1}H} NMR (101 MHz, CDCl$_3$)** δ 157.7, 142.9, 137.9, 133.8, 130.4, 130.3, 127.7, 121.3, 114.2, 113.5, 55.3, 54.6, 42.2, 27.1, 22.4. **HRMS** (ESI-TOF) *m/z*: $[M + H]^+$ for $C_{26}H_{29}N_2O$ calculated 385.2274; found 385.2274.

6,6'-((4-Nitrophenyl)methylene)bis(1,2,3,4-tetrahydroquinoline) (6aj). General Procedure E, purified by silica gel chromatography with EtOAc/hexane (20:80). Orange solid (149 mg, 93%; mp 90–91°C). **^{1}H NMR (400 MHz, CDCl$_3$)** δ 8.15 – 8.05 (m, AA' part of AA'BB' system, 2H), 7.33 – 7.26 (m, AA' part of AA'BB' system, 2H), 6.71 – 6.61 (m, 4H), 6.40 (d, *J* = 7.8 Hz, B part of AB system, 2H), 5.31 (s, 1H), 3.79 (bs, 2H), 3.28 (t, *J* = 6.3 Hz, 4H), 2.69 (t, *J* = 6.3 Hz, 3H), 1.92 (p, *J* = 6.3 Hz, 4H), (2H, not observed). **^{13}C{^{1}H} NMR (101 MHz, CDCl$_3$)** δ 153.8, 146.2, 143.4, 131.6, 130.3, 130.2, 127.6, 123.5, 121.5, 114.3, 55.4, 42.1, 27.1, 22.2. **HRMS** (ESI-TOF) m/z: $C_{25}H_{26}N_3O_2$ for $[M + H]^+$ calculated 400.2020; found 400.2020.

6,6'-((4-(Trifluoromethyl)phenyl)methylene)bis(1,2,3,4-tetrahydroquinoline) (6ak). General Procedure E (for 7 hours), purified by silica gel chromatography with EtOAc/hexane (20:80). Yellow

solid (136 mg, 80%; mp 85–86°C). **^{1}H NMR (400 MHz, CDCl$_3$)** δ 7.51 – 7.45 (m, AA' part of AA'BB' system, 2H), 7.26 – 7.21 (m, BB' part of AA'BB' system, 2H), 6.67 (s, 2H), 6.65 (d, *J* = 8.1 Hz, A part of AB system, 2H), 6.37 (d, *J* = 8.1 Hz, B part of AB system, 2H), 5.26 (s, 1H), 3.73 (bs, 2H), 3.25 (t, *J* = 6.4 Hz, 4H), 2.67 (t, *J* = 6.4 Hz, 4H), 1.90 (p, *J* = 6.4 Hz, 4H). **^{13}C{^{1}H} NMR (101 MHz, CDCl$_3$)** δ 150.0, 143.3, 132.4, 130.4, 129.7, 128.0 (q, *J* = 32.2 Hz), 127.7, 125.1 (q, *J* = 3.7 Hz), 124.6 (q, *J* = 271.9 Hz), 121.4, 114.2, 55.4, 42.1, 27.1, 22.3. **HRMS** (ESI-TOF) *m/z*: $[M + H]^+$ for $C_{26}H_{26}F_3N_2$ calculated 423.2043; found 423.2043.

Methyl 4-(bis(1,2,3,4-tetrahydroquinolin-6-yl)methyl)benzoate (6al). General Procedure E, purified by silica gel chromatography with EtOAc/hexane (20:80). Green solid (149 mg, 90%; 86%, using DCE as solvent; mp 76–77°C). **^{1}H NMR (400 MHz, CDCl$_3$)** δ 7.96 – 7.89 (m, AA' part of AA'BB' system, 2H), 7.25 – 7.18 (m, AA' part of AA'BB' system, 2H), 6.72 – 6.63 (m, 4H), 6.39 (d, *J* = 7.9 Hz, B part of AB system, 2H), 5.28 (s, 1H), 3.89 (s, 3H), 3.27 (t, *J* = 6.4 Hz, 4H), 2.68 (t, *J* = 6.4 Hz, 4H), 1.91 (p, *J* = 6.4 Hz, 4H), (2H, not observed). **^{13}C{^{1}H} NMR (101 MHz, CDCl$_3$)** δ 167.4, 151.3, 143.2, 132.5, 130.4, 129.5 (2C), 127.74, 127.7, 121.4, 114.5, 55.5, 52.1, 42.1, 27.1, 22.3. **HRMS** (ESI-TOF) *m/z*: $[M + H]^+$ for $C_{27}H_{29}N_2O_2$ calculated 413.2224; found 413.2224.

3-(Bis(1,2,3,4-tetrahydroquinolin-6-yl)methyl)phenol (6am). General Procedure E, purified by silica gel chromatography with EtOAc/hexane (20:80). Green solid (117 mg, 79%; mp 87–88°C). **^{1}H NMR (400 MHz, CDCl$_3$)** δ 7.10 (t, *J* = 7.7 Hz, 1H), 6.74 – 6.65 (m, 5H), 6.62 (d, *J* = 7.7 Hz, 1H), 6.54 (s, 1H), 6.38 (d, *J* = 8.1 Hz, B part of AB system, 2H), 5.17 (s, 1H), 4.13 (bs, 3H), 3.24 (t, *J* = 6.4 Hz, 4H), 2.67 (t, *J* = 6.4 Hz, 4H), 1.90 (p, *J* = 6.4 Hz, 4H). **^{13}C{^{1}H} NMR (101 MHz, CDCl$_3$)** δ 155.7, 147.5, 142.8, 133.6, 130.5, 129.3, 127.7, 121.9, 121.7, 116.5, 114.5, 112.9, 55.3, 42.2, 27.0, 22.3. **HRMS** (ESI-TOF) *m/z*: $[M + H]^+$ for $C_{25}H_{27}N_2O$ calculated 371.2118; found 371.2118.

6,6'-(*m*-Tolylmethylene)bis(1,2,3,4-tetrahydroquinoline) (6an). General Procedure E, purified by silica gel chromatography with EtOAc/hexane (10:90). Green solid (112 mg, 76%; mp 85–86 °C). **^{1}H NMR (400 MHz, CDCl$_3$)** δ 7.16 (t, *J* = 7.7 Hz, 1H), 7.03 – 6.97 (m, 2H), 6.94 (d, *J* = 7.7 Hz, 1H), 6.73 (s, 2H), 6.70 (d, *J* = 8.2 Hz, A part of AB system, 2H), 6.40 (d, *J* = 8.2 Hz, B part of AB system, 2H), 5.22 (s, 1H), 3.65 (bs, 2H), 3.28 (t, *J* = 6.2 Hz, 4H), 2.70 (t, *J* = 6.2 Hz, 4H), 2.30 (s, 3H), 1.93 (p, *J* = 6.2 Hz, 4H). **^{13}C{^{1}H} NMR (101 MHz, CDCl$_3$)** δ 145.9, 143.05, 137.9, 133.7, 130.6, 130.3, 128.1, 128.0, 126.74, 126.7, 121.5, 114.4, 55.6, 42.4, 27.2, 22.6, 21.8. **HRMS** (ESI-TOF) *m/z*: $[M + H]^+$ for $C_{26}H_{29}N_2$ calculated 369.2325; found 369.2325.

6,6'-((3-Bromophenyl)methylene)bis(1,2,3,4-tetrahydroquinoline) (6ao). General Procedure E (for 7 hours), purified by silica gel chromatography with EtOAc/hexane (10:90). Green solid (127 mg, 73%; mp 87–88°C). **^{1}H NMR (400 MHz, CDCl$_3$)** δ 7.33 – 7.27 (m, 2H), 7.11 (t, *J* = 7.7 Hz, 1H), 7.06 (d, *J* = 7.7 Hz, 1H), 6.70 – 6.63 (m, 4H), 6.39 (d, *J* = 7.9 Hz, B part of AB system, 2H), 5.19 (s, 1H),

3.78 (bs, 2H), 3.28 (t, *J* = 6.4 Hz, 4H), 2.69 (t, *J* = 6.4 Hz, 4H), 1.92 (p, *J* = 6.4 Hz, 4H). **^{13}C{^{1}H} NMR (101 MHz, CDCl$_3$)** δ 148.2, 143.2, 132.5, 132.4, 130.4, 129.7, 129.0, 128.2, 127.7, 122.4, 121.4, 114.2, 55.3, 42.1, 27.1, 22.3. **HRMS** (ESI-TOF) *m/z*: [M + H]$^+$ for $C_{25}H_{26}BrN_2$ calculated 433.1274; found 433.1273.

6,6'-(*o*-Tolylmethylene)bis(1,2,3,4-tetrahydroquinoline) (6ap). General Procedure E, purified by silica gel chromatography with EtOAc/hexane (10:90). Green solid (104 mg, 70%; mp 85–86°C). **^{1}H NMR (400 MHz, CDCl$_3$)** δ 7.15 – 7.08 (m, 3H), 6.93 – 6.89 (m, 1H), 6.67 (s, 2H), 6.64 (d, *J* = 8.2 Hz, A part of AB system, 2H), 6.39 (d, *J* = 8.2 Hz, B part of AB system, 2H), 5.37 (s, 1H), 3.54 (bs, 2H), 3.28 (t, *J* = 6.4 Hz, 4H), 2.68 (t, *J* = 6.4 Hz, 4H), 2.24 (s, 3H), 1.92 (p, *J* = 6.4 Hz, 4H). **^{13}C{^{1}H} NMR (101 MHz, CDCl$_3$)** δ 144.0, 142.8, 136.6, 132.9, 130.6, 130.2, 129.5, 127.9, 125.8, 125.6, 121.4, 114.2, 52.08, 42.2, 27.1, 22.4, 20.1. **HRMS** (ESI-TOF) *m/z*: [M + H]$^+$ for $C_{26}H_{29}N_2$ calculated 369.2325; found 369.2325.

1,4-Bis(bis(1,2,3,4-tetrahydroquinolin-6-yl)methyl)benzene (6aq). General Procedure E, purified by silica gel chromatography with EtOAc/hexane (20:80). Tetrahydroquinoline (**5a**; 3 mmol, 5 equivalent). Green solid (238 mg, 94%; mp 111–112°C). **^{1}H NMR (400 MHz, CDCl$_3$)** δ 7.01 (s, 4H), 6.73 – 6.67 (m, 8H), 6.38 (d, *J* = 7.9 Hz, B part of AB system, 4H), 5.19 (s, 2H), 3.62 (bs, 4H), 3.26 (t, *J* = 6.4 Hz, 8H), 2.68 (t, *J* = 6.4 Hz, 8H), 1.91 (p, *J* = 6.4 Hz, 8H). **^{13}C{^{1}H} NMR (101 MHz, CDCl$_3$)** δ 142.9, 142.8, 133.9, 130.5, 129.0, 127.7, 121.3, 114.1, 55.2, 42.2, 27.1, 22.4. **HRMS** (ESI-TOF) *m/z*: [M + H]$^+$ for $C_{44}H_{47}N_4$ calculated 631.3795; found 631.3793.

6,6'-(Naphthalene-2-ylmethylene)bis(1,2,3,4-tetrahydroquinoline) (6ar). General Procedure E, purified by silica gel chromatography with EtOAc/hexane (10:90). White solid (130 mg, 80%; mp 96–97°C). **^{1}H NMR (400 MHz, CDCl$_3$)** δ 7.84 – 7.79 (m, 1H), 7.78 – 7.72 (m, 2H), 7.53 (s, 1H), 7.47 – 7.40 (m, 2H), 7.36 (dd, *J* = 8.5, 1.5 Hz, 1H), 6.88 – 6.68 (m, 4H), 6.42 (d, *J* = 7.9 Hz, B part of AB system, 2H), 5.43 (s, 1H), 3.67 (bs, 2H), 3.29 (t, *J* = 6.4 Hz, 4H), 2.70 (t, *J* = 6.4 Hz, 4H), 1.93 (p, *J* = 6.4 Hz, 4H). **^{13}C{^{1}H} NMR (101 MHz, CDCl$_3$)** δ 143.4, 143.0, 133.5, 133.2, 132.1, 130.6, 128.6, 128.0, 127.9, 127.6, 127.5, 125.8, 125.3, 121.4, 114.3, 55.6, 42.2, 27.1, 22.4, (1 signal overlapped). **HRMS** (ESI-TOF) *m/z*: [M + H]$^+$ for $C_{29}H_{29}N_2$ calculated 405.2325; found 405.2326.

6,6'-(Pyridin-2-ylmethylene)bis(1,2,3,4-tetrahydroquinoline) (6au$_2$). General Procedure E (for 7 hours), purified by silica gel chromatography with EtOAc/hexane (20:80). White solid (113 mg, 80%; mp 84–85°C). **^{1}H NMR (400 MHz, CDCl$_3$)** δ 8.56 (d, *J* = 4.0 Hz, 1H), 7.56 (td, *J* = 7.8, 1.8 Hz, 1H), 7.11 (d, *J* = 7.8 Hz, 1H), 7.09 – 7.05 (m, 1H), 6.78 – 6.71 (m, 4H), 6.39 (d, *J* = 7.9 Hz, B part of AB system, 2H), 5.39 (s, 1H), 3.72 (bs, 2H), 3.26 (t, *J* = 6.4 Hz, 4H), 2.68 (t, *J* = 6.4 Hz, 4H), 1.90 (p, *J* = 6.4 Hz, 4H). **^{13}C{^{1}H} NMR (101 MHz, CDCl$_3$)** δ 165.1, 149.4, 143.2, 136.2, 132.1, 130.4, 127.7,

123.6, 121.4, 121.0, 114.3, 58.2, 42.2, 27.1, 22.4. **HRMS** (ESI-TOF) *m/z*: $[M + H]^+$ for $C_{24}H_{26}N_3$ calculated 356.2121; found 356.2121.

6,6'-(Pyridin-3-ylmethylene)bis(1,2,3,4-tetrahydroquinoline) (6au$_3$). General Procedure E (for 7 hours), purified by silica gel chromatography with EtOAc/hexane (20:80). White solid (120 mg, 85%; mp 84–85°C). **^{1}H NMR (400 MHz, CDCl$_3$)** δ 8.45 – 8.40 (m, 2H), 7.43 (dt, J = 7.9, 1.7 Hz, 1H), 7.20 – 7.15 (m, 1H), 6.70 – 6.64 (m, 4H), 6.39 (d, J = 8.3 Hz, B part of AB system, 2H), 5.24 (s, 1H), 3.78 (bs, 2H), 3.27 (t, J = 6.4 Hz, 4H), 2.68 (t, J = 6.4 Hz, 4H), 1.91 (p, J = 6.4 Hz, 4H). **^{13}C{^{1}H} NMR (101 MHz, CDCl$_3$)** δ 150.9, 147.2, 143.3, 136.8, 132.1, 130.3, 127.6, 123.1, 121.4, 114.2, 53.1, 42.1, 27.1, 22.3. **HRMS** (ESI-TOF) *m/z*: $[M + H]^+$ for $C_{24}H_{26}N_3$ calculated 356.2121; found 356.2122.

6,6'-(Pyridin-4-ylmethylene)bis(1,2,3,4-tetrahydroquinoline) (6au$_4$). General Procedure E (for 7 hours), purified by silica gel chromatography with EtOAc/hexane (20:80). Green solid (133 mg, 94%; mp 82–83°C). **^{1}H NMR (400 MHz, CDCl$_3$)** δ 8.48 – 8.44 (m, AA' part of AA'BB' system, 2H), 7.09 – 7.05 (m, BB' part of AA'BB' system, 2H), 6.69 – 6.64 (m, 4H), 6.39 (d, J = 8.8 Hz, B part of AB system, 2H), 5.18 (s, 1H), 3.77 (bs, 2H), 3.27 (t, J = 6.4 Hz, 4H), 2.68 (t, J = 6.4 Hz, 4H), 1.91 (p, J = 6.4 Hz, 4H). **^{13}C{^{1}H} NMR (101 MHz, CDCl$_3$)** δ 154.8, 149.6, 143.4, 131.5, 130.4, 127.7, 124.8, 121.5, 114.2, 55.0, 42.1, 27.1, 22.3. **HRMS** (ESI-TOF) *m/z*: $[M + H]^+$ for $C_{24}H_{26}N_3$ calculated 356.2121; found 356.2122.

6,6'-(Thien-2-ylmethylene)bis(1,2,3,4-tetrahydroquinoline) (6av$_2$). General Procedure E, purified by silica gel chromatography with EtOAc/hexane (10:90). Green solid (109 mg, 76%; mp 81–82°C). **^{1}H NMR (400 MHz, CDCl$_3$)** δ 7.17 (dd, J = 5.2, 1.1 Hz, 1H), 6.94 – 6.90 (m, 1H), 6.84 – 6.79 (m, 4H), 6.73 – 6.68 (m, 1H), 6.41 (d, J = 8.8 Hz, B part of AB system, 2H), 5.38 (s, 1H), 3.75 (bs, 2H), 3.28 (t, J = 6.4 Hz, 4H), 2.72 (t, J = 6.4 Hz, 4H), 1.93 (p, J = 6.4 Hz, 4H). **^{13}C{^{1}H} NMR (101 MHz, CDCl$_3$)** δ 150.4, 143.3, 133.4, 129.9, 127.1, 126.4, 125.7, 123.9, 121.3, 114.2, 50.9, 42.2, 27.1, 22.4. **HRMS** (ESI-TOF) *m/z*: $[M + H]^+$ for $C_{23}H_{25}N_2S$ calculated 361.1733; found 361.1733.

6,6'-((1-Tosil-1H-pirol-2-il)metilen)bis(1,2,3,4-tetrahidrokinolin) (6aw$_2$). General Procedure G, purified by silica gel chromatography with EtOAc/hexane (10:90). Off-white (113 mg, 67%; mp 89-90 °C). **^{1}H NMR (400 MHz, CDCl$_3$)** δ 7.33 (dd, J = 3.2, 1.5 Hz, 1H), 7.32 – 7.28 (m, AA' part of AA'BB' system, 2H), 7.08 – 7.03 (m, BB' part of AA'BB' system, 2H), 6.53 (dd, J = 8.2, 1.7 Hz, 2H), 6.46 (s, 2H), 6.25 (d, J = 8.2 Hz, 2H), 6.18 (t, J = 3.2 Hz, 1H), 5.77 – 5.72 (m, 2H), 3.64 (bs, 2H), 3.44 – 3.08 (m, 4H), 2.65 – 2.56 (m, 2H), 2.55 – 2.46 (m, 2H), 2.36 (s, 3H), 1.91 – 1.84 (m, 4H). **^{13}C{^{1}H} NMR (101 MHz, CDCl$_3$)** δ 143.7, 143.1, 139.5, 136.2, 131.8, 130.0 129.3, 127.4, 127.0, 122.5, 121.0, 115.4, 114.1, 110.9, 47.0, 42.1, 27.0, 22.4, 21.7. **HRMS** (ESI-TOF) *m/z*: $[M + H]^+$ for $C_{30}H_{32}N_3O_2S$ calculated 498.2210; found 498.2209.

6,6'-((1-Tosyl-1*H*-indol-3-yl)methylene)bis(1,2,3,4-tetrahydroquinoline) (6ay$_3$). General Procedure E (for 56 hours), purified by silica gel chromatography with EtOAc/hexane (20:80). Green solid (164 mg, 75%; mp 90–91 °C). **^{1}H NMR (400 MHz, CDCl$_3$)** δ 7.95 (d, *J* = 8.3 Hz, 1H), 7.71 – 7.65 (m, AA' part of AA'BB' system, 2H), 7.28 – 7.15 (m, 4H), 7.08 (t, *J* = 7.5 Hz, 1H), 6.98 (s, 1H), 6.70 (s, 2H), 6.68 (dd, *J* = 8.2 Hz, *J* = 1.8 Hz, A part of AB system, 2H), 6.37 (d, *J* = 8.2 Hz, B part of AB system, 2H), 5.21 (s, 1H), 3.46 – 3.04 (m, 4H), 2.72 – 2.63 (m, 4H), 2.35 (s, 3H), 2.11 – 1.79 (m, 4H), (2H, not observed). **^{13}C{^{1}H} NMR (101 MHz, CDCl$_3$)** δ 144.7, 143.2, 135.1, 135.3, 135.3, 134.7, 129.9, 129.8, 128.8, 127.1, 126.9, 125.7, 124.5, 123.2, 121.5, 120.9, 114.3, 113.9, 47.0, 42.2, 27.1, 22.4, 21.7. **HRMS** (ESI-TOF) *m/z*: $[M + H]^+$ for $C_{34}H_{34}N_3O_2S$ calculated 548.2366; found 548.2366.

6,6'-((1-Tosyl-1*H*-indol-4-yl)methylene)bis(1,2,3,4-tetrahydroquinoline) (6ay$_4$). General Procedure E (for 56 hours), purified by silica gel chromatography with EtOAc/hexane (20:80). Green solid (186 mg, 85%; mp 89–90°C). **^{1}H NMR (400 MHz, CDCl$_3$)** δ 7.82 (d, *J* = 8.3 Hz, 1H), 7.78 – 7.74 (m, AA' part of AA'BB' system, 2H), 7.45 (d, *J* = 3.7 Hz, 1H), 7.23 – 7.15 (m, 3H), 6.82 (d, *J* = 7.4 Hz, 1H), 6.68 – 6.62 (m, 4H), 6.52 (d, *J* = 3.7 Hz, 1H), 6.35 (d, *J* = 8.0 Hz, A part of AB system, 2H), 5.50 (s, 1H), 3.50 (bs, 2H), 3.25 (t, *J* = 6.3 Hz, 4H), 2.64 (t, *J* = 6.3 Hz, 4H), 2.34 (s, 3H), 1.89 (p, *J* = 6.3 Hz, 4H). **^{13}C{^{1}H} NMR (101 MHz, CDCl$_3$)** δ 146.6, 144.7, 140.5, 137.3, 136.5, 134.2, 132.1, 132.1, 131.6, 129.4, 128.7, 127.3, 126.1, 125.4, 123.1, 115.9, 113.1, 110.0, 54.5, 43.9, 28.8, 24.1, 23.4. **HRMS** (ESI-TOF) *m/z*: $[M + H]^+$ for $C_{34}H_{34}N_3O_2S$ calculated 548.2366; found 548.2366.

6,6'-((1-Tosyl-1*H*-indol-5-yl)methylene)bis(1,2,3,4-tetrahydroquinoline) (6ay$_5$). General Procedure E (for 56 hours), purified by silica gel chromatography with EtOAc/hexane (20:80). Green solid (184 mg, 84%; mp 94–95°C). **^{1}H NMR (400 MHz, CDCl$_3$)** δ 7.84 (d, *J* = 8.6 Hz, A part of AB system, 1H), 7.78 – 7.74 (m, AA' part of AA'BB' system, 2H), 7.50 (d, *J* = 3.6 Hz, 1H), 7.23 – 7.18 (m, 3H), 7.14 (dd, *J* = 8.6, 1.2 Hz, B part of AB system, 1H), 6.70 – 6.64 (m, 4H), 6.54 (d, *J* = 3.6 Hz, 1H), 6.37 (d, *J* = 7.9 Hz, B part of AB system, 2H), 5.30 (s, CH, 1H), 3.26 (t, *J* = 6.4 Hz, 4H), 2.66 (t, *J* = 6.4 Hz, 4H), 2.34 (s, 3H), 1.90 (t, *J* = 6.4 Hz, 4H), (2H, not observed). **^{13}C{^{1}H} NMR (101 MHz, CDCl$_3$)** δ 144.9, 143.0, 141.0, 135.5, 133.5, 133.3, 130.8, 130.5, 129.9, 127.8, 127.0, 126.7, 126.2, 121.7, 121.3, 114.1, 113.1, 109.3, 55.3, 42.2, 27.1, 22.4, 21.7. **HRMS** (ESI-TOF) *m/z*: $[M + H]^+$ for $C_{34}H_{34}N_3O_2S$ calculated 548.2366; found 548.2366.

6,6'-((1-Tosyl-1*H*-indol-6-yl)methylene)bis(1,2,3,4-tetrahydroquinoline) (6ay$_6$). General Procedure E (for 56 hours), purified by silica gel chromatography with EtOAc/hexane (20:80). Green solid (186 mg, 85%; mp 93–94°C). **^{1}H NMR (400 MHz, CDCl$_3$)** δ 7.74 (bs, CH, 1H), 7.61 – 7.56 (m, AA' part of AA'BB' system, 2H), 7.50 (d, *J* = 3.6 Hz, 1H), 7.38 (d, *J* = 8.1 Hz, A part of AB system, 1H), 7.17 – 7.12 (m, BB' part of AA'BB' system, 2H), 7.07 (dd, *J* = 8.1, 1.2 Hz, B part of AB system,

1H), 6.73 (s, 2H), 6.71 (d, *J* = 8.1 Hz, A part of AB system, 2H), 6.58 (d, *J* = 3.6 Hz, 1H), 6.42 (d, *J* = 8.1 Hz, B part of AB system, 2H), 5.36 (s, 1H), 3.55 (bs, 2H), 3.30 (t, *J* = 6.4 Hz, 4H), 2.70 (t, *J* = 6.4 Hz, 4H), 2.33 (s, 3H), 1.94 (p, *J* = 6.4 Hz, 4H). **$^{13}C\{^1H\}$ NMR (101 MHz, $CDCl_3$)** δ 144.7, 142.95, 142.7, 134.9, 134.9, 133.5, 130.5, 129.8, 128.8, 127.7, 127.1, 126.1, 125.3, 121.3, 120.8, 114.5, 114.2, 108.9, 55.7, 42.2, 27.1, 22.4, 21.7. **HRMS** (ESI-TOF) *m/z*: $[M + H]^+$ for $C_{34}H_{34}N_3O_2S$ calculated 548.2366; found 548.2366.

4,5-Bis(3,4-dihydroquinolin-1(2*H*)-yl)cyclopent-2-en-1-one (12). General Procedure E, purified by silica gel chromatography with EtOAc/hexane (10:90). Yellow oil (120 mg, 87%). **^1H NMR (400 MHz, $CDCl_3$)** δ 7.66 (dd, *J* = 6.3, 2.0 Hz, 1H), 7.00 – 6.84 (m, 4H), 6.65 – 6.48 (m, 4H), 6.07 (d, *J* = 8.1 Hz, 1H), 5.37 (bs, 1H), 4.30 (bs, 1H), 3.35 – 3.11 (m, 4H), 2.82 – 2.61 (m, 4H), 2.03 – 1.87 (m, 4H). **$^{13}C\{^1H\}$ NMR (101 MHz, $CDCl_3$)** δ 202.0, 161.8, 144.4, 143.5, 134.4, 129.6 (2C), 127.1, 126.9, 123.7, 123.5, 117.3, 117.13, 111.5, 110.8, 68.5, 59.9, 45.3, 41.9, 28.1, 28.0, 22.5, 22.3. Spectroscopic data for the title compound were consistent with those reported in the literature.[26]

6,6'-((4-Nitrophenyl)methylene)bis(5-chloro-1,2,3,4-tetrahydroquinoline) (6bj). General Procedure F, purified by silica gel chromatography with EtOAc/hexane (20:80). Yellow solid (138 mg, 82%; mp 134–135°C). **^1H NMR (400 MHz, $CDCl_3$)** δ 8.13 – 8.08 (m, AA' part of AA'BB' system, 2H), 7.23 – 7.18 (m, BB' part of AA'BB' system, 2H), 6.38 (d, *J* = 8.4 Hz, A part of AB system, 2H), 6.30 (d, *J* = 8.4 Hz, B part of AB system, 2H), 6.18 (s, 1H), 3.74 (bs, 2H), 3.27 – 3.21 (m, 4H), 2.78 (t, *J* = 6.5 Hz, 4H), 1.99 – 1.92 (m, 4H). **$^{13}C\{^1H\}$ NMR (101 MHz, $CDCl_3$)** δ 151.8, 146.4, 145.1, 135.2, 130.3, 128.2, 128.1, 123.5, 120.1, 112.3, 50.3, 41.3, 25.4, 22.0. **HRMS** (ESI-TOF) *m/z*: $C_{25}H_{24}Cl_2N_3O_2$ for $[M + H]^+$ calculated 468.1240; found 468.1241.

6,6'-((4-Nitrophenyl)methylene)bis(5-bromo-1,2,3,4-tetrahydroquinoline) (6cj). General Procedure E, purified by silica gel chromatography with EtOAc/hexane (20:80). Yellow solid (161 mg, 79%; mp 141–142°C). **^1H NMR (400 MHz, $CDCl_3$)** δ 8.12 – 8.08 (m, AA' part of AA'BB' system, 2H), 7.22 – 7.17 (m, BB' part of AA'BB' system, 2H), 6.37 (d, *J* = 8.4 Hz, A part of AB system, 2H), 6.33 (d, *J* = 8.4 Hz, B part of AB system, 2H), 6.18 (s, 1H), 3.75 (bs, 2H), 3.27 – 3.20 (m, 4H), 2.77 (t, *J* = 6.5 Hz, 4H), 2.00 – 1.91 (m, 4H). **$^{13}C\{^1H\}$ NMR (101 MHz, $CDCl_3$)** δ 152.0, 146.4, 145.2, 130.5, 130.0, 129.3, 128.6, 123.5, 121.8, 113.0, 55.8, 41.3, 28.7, 22.4. **HRMS** (ESI-TOF) *m/z*: $[M + H]^+$ for $C_{25}H_{24}Br_2N_3O_2$ calculated 556.0230; found 556.0229.

6,6'-((4-Nitrophenyl)methylene)bis(5-methoxy-1,2,3,4-tetrahydroquinoline) (6ej). General Procedure E, purified by silica gel chromatography with EtOAc/hexane (20:80). Yellow solid (122 mg, 74%; mp 114–115°C). **^1H NMR (400 MHz, $CDCl_3$)** δ 8.16 – 8.12 (m, AA' part of AA'BB' system, 2H), 7.31 – 7.27 (m, BB' part of AA'BB' system, 2H), 6.36 (d, *J* = 8.5 Hz, A part of AB system, 2H), 6.16 (d, *J* = 8.5 Hz, B part of AB system, 2H), 5.17 (s, 1H), 3.78 (s, 6H), 3.16 – 3.11 (m,

4H), 2.68 (t, *J* = 6.5 Hz, 4H), 1.87 – 1.78 (m, 4H), (NH, 1H, not observed). **^{13}C{^{1}H} NMR (101 MHz, $CDCl_3$)** δ 157.0, 151.1, 147.0, 143.3, 130.5, 127.2, 123.8, 118.7, 110.8, 99.0, 55.3, 45.6, 42.0, 21.6, 21.2. **HRMS** (ESI-TOF) *m/z*: [M + H]$^+$ for $C_{27}H_{30}N_3O_4$ calculated 460.2231; found 460.2220.

6,6'-((4-Nitrophenyl)methylene)bis(7-bromo-1,2,3,4-tetrahydroquinoline) (6gj). General Procedure E, purified by silica gel chromatography with EtOAc/hexane (20:80). Yellow solid (166 mg, 81%; mp 128–129°C). **^{1}H NMR (400 MHz, $CDCl_3$)** δ 8.13 – 8.09 (m, AA' part of AA'BB' system, 2H), 7.22 – 7.17 (m, BB' part of AA'BB' system, 2H), 6.70 (s, 2H), 6.29 (s, 2H), 5.96 (s, 1H), 3.93 (bs, 2H), 3.29 – 3.24 (m, 4H), 2.57 (td, *J* = 5.9, 1.8 Hz, 4H), 1.92 – 1.84 (m, 4H). **^{13}C{^{1}H} NMR (101 MHz, $CDCl_3$)** δ 151.6, 146.4, 144.6, 131.3, 130.5, 129.0, 123.5, 123.0, 120.7, 117.9, 54.2, 41.7, 26.8, 21.7. **HRMS** (ESI-TOF) *m/z*: [M + H]$^+$ for $C_{25}H_{24}Br_2N_3O_2$ calculated 556.0230; found 556.0228.

6,6'-((4-Nitrophenyl)methylene)bis(2-methyl-1,2,3,4-tetrahydroquinoline) (6oj). General Procedure E, purified by silica gel chromatography with EtOAc/hexane (20:80). Green oil (108 mg, 71%). **^{1}H NMR (400 MHz, $CDCl_3$)** δ 8.12 – 8.06 (m, AA' part of AA'BB' system, 2H), 7.29 – 7.26 (m, BB' part of AA'BB' system 2H), 6.69 – 6.61 (m, 4H), 6.43 (d, *J* = 7.8 Hz, B part of AB system, 2H), 5.31 (s, 1H), 3.46 – 3.29 (m, 2H), 2.83 – 2.58 (m, 4H), 1.95 – 1.85 (m, 2H), 1.63 – 1.52 (m, 2H), 1.21 (d, *J* = 6.2 Hz, 6H), (NH, 1H, not observed). **^{13}C{^{1}H} NMR (101 MHz, $CDCl_3$)** δ 153.7, 146.2, 143.2, 131.8, 130.2, 130.1, 127.6, 123.4, 121.3, 114.2, 55.4, 47.4, 30.1, 26.7, 22.6. **HRMS** (ESI-TOF) *m/z*: [M + H]$^+$ for $C_{27}H_{30}N_3O_2$ calculated 428.2333; found 428.2314.

6,6'-((4-Nitrophenyl)methylene)bis(1-benzyl-1,2,3,4-tetrahydroquinoline) (14aj). General Procedure G, purified by silica gel chromatography with EtOAc/hexane (10:90). Yellow solid (206 mg, 89%; mp 86–87°C). **^{1}H NMR (400 MHz, $CDCl_3$)** δ 8.12 – 8.07 (m, AA' part of AA'BB' system, 2H), 7.34 – 7.29 (m, 5H), 7.29 – 7.22 (m, 7H), 6.70 (d, *J* = 2.0 Hz, 2H), 6.64 (dd, *J* = 8.5, 2.0 Hz, A part of AB system, 2H), 6.41 (d, *J* = 8.5 Hz, B part of AB system, 2H), 5.29 (s, 1H), 4.44 (s, 4H), 3.38 – 3.32 (m, 4H), 2.74 (t, *J* = 6.2 Hz, 4H), 2.04 – 1.95 (m, 4H). **^{13}C{^{1}H} NMR (101 MHz, $CDCl_3$)** δ 153.9, 146.1, 144.4, 139.1, 130.3, 130.2, 129.9, 128.7, 127.9, 126.9, 126.7, 123.4, 122.3, 110.9, 55.5, 55.2, 50.0, 28.3, 22.5; **HRMS** (ESI-TOF) *m/z*: [M + H]$^+$ for $C_{39}H_{38}N_3O_2$ calculated 580.2959; found 580.2945.

6,6'-((4-Nitrophenyl)methylene)bis(1-benzyl-5-chloro-1,2,3,4-tetrahydroquinoline) (14bj). General Procedure G, purified by silica gel chromatography with EtOAc/hexane (10:90). Yellow oil (220 mg, 85%). **^{1}H NMR (400 MHz, $CDCl_3$)** δ 8.10 – 8.05 (m, AA' part of AA'BB' system, 2H), 7.33 – 7.28 (m, 5H), 7.25 – 7.21 (m, 5H), 7.19 – 7.16 (m, BB' part of AA'BB' system, 2H), 6.34 (d, *J* = 8.7 Hz, A part of AB system, 2H), 6.28 (d, *J* = 8.7 Hz, B part of AB system, 2H), 6.16 (s, 1H), 4.43 (s, 4H), 3.50 – 3.13 (m, 4H), 2.87 (t, *J* = 6.5 Hz, 4H), 2.20 – 1.87 (m, 4H). **^{13}C{^{1}H} NMR (101 MHz, $CDCl_3$)** δ 152.1, 146.4, 145.9, 138.7, 134.7, 130.4, 128.8, 128.5, 127.1, 127.0, 126.6, 123.5, 120.7,

109.2, 55.9, 50.4, 49.7, 26.4, 22.1; **HRMS** (ESI-TOF) *m/z*: $[M + H]^+$ for $C_{39}H_{36}Cl_2N_3O_2$ calculated 648.2179; found 648.2177.

6,6'-((4-Nitrophenyl)methylene)bis(1-benzyl-5-bromo-1,2,3,4-tetrahydroquinoline) (14cj). General Procedure G, purified by silica gel chromatography with EtOAc/hexane (10:90). Yellow solid (262 mg, 89%; mp 97–98°C). **^{1}H NMR (400 MHz, $CDCl_3$)** δ 8.12 – 8.03 (m, AA' part of AA'BB' system, 2H), 7.34 – 7.28 (m, 4H), 7.25 – 7.21 (m, 6H), 7.19 – 7.14 (m, BB' part of AA'BB' system, 2H), 6.35 – 6.27 (m, 4H), 6.18 (s, 1H), 4.43 (s, 4H), 3.41 – 3.29 (m, 4H), 2.88 (t, *J* = 6.5 Hz, 4H), 2.08 – 1.99 (m, 4H). **$^{13}C\{^1H\}$ NMR (101 MHz, $CDCl_3$)** δ 152.2, 146.3, 146.0, 138.6, 130.6, 128.91, 128.89, 128.77 (2C), 127.0, 126.5, 123.5, 122.4, 109.9, 55.9, 55.8, 49.7, 29.8, 22.5; **HRMS** (ESI-TOF) *m/z*: $[M + H]^+$ for $C_{39}H_{36}Br_2N_3O_2$ calculated 736.1169; found 736.1174.

6,6'-((4-Nitrophenyl)methylene)bis(1-benzyl-5-methoxy-1,2,3,4-tetrahydroquinoline) (14ej). General Procedure G, purified by silica gel chromatography with EtOAc/hexane (10:90). Yellow solid (204 mg, 80%; mp 85–86°C). **^{1}H NMR (400 MHz, $CDCl_3$)** δ 8.13 – 8.04 (m, AA' part of AA'BB' system, 2H), 7.38 – 7.21 (m, 12H), 6.48 (d, *J* = 8.6 Hz, A part of AB system, 2H), 6.24 (d, *J* = 8.6 Hz, B part of AB system, 2H), 6.12 (s, 1H), 4.46 (s, 4H), 3.60 (s, 6H), 3.42 – 3.34 (m, 4H), 2.94 – 2.78 (m, 4H), 2.06 – 1.98 (m, 4H). **$^{13}C\{^1H\}$ NMR (101 MHz, $CDCl_3$)** δ 155.8, 154.4, 146.0, 145.8, 139.0, 129.9, 128.6, 128.4, 126.9, 126.6, 123.3, 123.2, 115.6, 106.9, 60.2, 55.7, 49.7, 42.8, 22.2, 22.0; **HRMS** (ESI-TOF) *m/z*: $[M + H]^+$ for $C_{41}H_{42}N_3O_4$ calculated 640.3170; found 640.3142.

6,6'-((4-Nitrophenyl)methylene)bis(1-benzyl-7-chloro-1,2,3,4-tetrahydroquinoline) (14fj). General Procedure G (at 90°C), purified by silica gel chromatography with EtOAc/hexane (10:90). Yellow solid (204 mg, 79%; mp 93–94°C). **^{1}H NMR (400 MHz, $CDCl_3$)** δ 8.16 – 8.11 (m, AA' part of AA'BB' system, 2H), 7.38 – 7.33 (m, 4H), 7.30 – 7.21 (m, 8H), 6.57 (s, 2H), 6.39 (s, 2H), 6.05 (s, 1H), 4.45 (s, 4H), 3.34 (t, *J* = 5.6 Hz, 4H), 2.71 – 2.62 (m, 4H), 2.01 – 1.92 (m, 2H). **$^{13}C\{^1H\}$ NMR (101 MHz, $CDCl_3$)** δ 151.9, 146.5, 145.6, 138.4, 132.9, 130.7, 130.4, 128.9, 127.3, 126.9, 126.2, 123.7, 121.0, 111.7, 55.3, 49.6, 49.1, 28.1, 22.3; **HRMS** (ESI-TOF) *m/z*: $[M + H]^+$ for $C_{39}H_{36}Cl_2N_3O_2$ calculated 648.2179; found 648.2166.

6,6'-((4-Nitrophenyl)methylene)bis(1-benzyl-7-methyl-1,2,3,4-tetrahydroquinoline) (14hj). General Procedure G (at 90°C), purified by silica gel chromatography with EtOAc/hexane (10:90). Brown oil (179 mg, 74%). **^{1}H NMR (400 MHz, $CDCl_3$)** δ 8.14 – 8.08 (m, AA' part of AA'BB' system, 2H), 7.37 – 7.21 (m, 12H), 6.38 (s, 2H), 6.29 (s, 2H), 5.50 (s, 1H), 4.46 (s, 4H), 3.40 – 3.22 (m, 4H), 2.66 (t, *J* = 6.0 Hz, 4H), 1.99 (s, 6H), 1.97 – 1.92 (m, 4H). **$^{13}C\{^1H\}$ NMR (101 MHz, $CDCl_3$)** δ 153.4, 146.2, 144.3, 139.4, 134.9, 130.6, 129.8, 128.6, 126.9 (3C), 123.4, 119.6, 113.2, 55.4, 49.7, 48.9, 28.0, 22.6, 20.0; **HRMS** (ESI-TOF) *m/z*: $[M + H]^+$ for $C_{41}H_{42}N_3O_2$ calculated 608.3272; found 608.3246.

6,6'-((4-Nitrophenyl)methylene)bis(1-benzyl-7-methoxy-1,2,3,4-tetrahydroquinoline) (14ij). General Procedure G (at 90°C), purified by silica gel chromatography with EtOAc/hexane (10:90). Yellow solid (199 mg, 78%; mp 92–93 °C). **^{1}H NMR (400 MHz, CDCl$_3$)** δ 8.09 – 8.03 (m, AA' part of AA'BB' system, 2H), 7.39 – 7.30 (m, 7H), 7.28 – 7.19 (m, 5H), 6.43 (s, 2H), 6.11 (s, 2H), 5.94 (s, 1H), 4.48 (s, 4H), 3.48 (s, 6H), 3.42 – 3.23 (m, 4H), 2.73 – 2.63 (m, 4H), 2.03 – 1.94 (m, 4H). **^{13}C{^{1}H} NMR (101 MHz, CDCl$_3$)** δ 156.4, 155.2, 145.7, 145.3, 139.4, 130.6, 129.7, 128.7, 127.0, 126.8, 123.1, 118.8, 114.0, 95.7, 56.0, 55.8, 50.1, 42.1, 27.6, 22.7; **HRMS** (ESI-TOF) *m/z*: $[M + H]^+$ for $C_{41}H_{42}N_3O_4$ calculated 640.3170; found 640.3151.

6,6'-((4-Nitrophenyl)methylene)bis(1-benzyl-8-methyl-1,2,3,4-tetrahydroquinoline) (14mj). General Procedure G (at 120°C), purified by silica gel chromatography with EtOAc/hexane (10:90). Green oil (182 mg, 75%). **^{1}H NMR (400 MHz, CDCl$_3$)** δ 8.18 – 8.13 (m, AA' part of AA'BB' system, 2H), 7.55 – 7.49 (m, 4H), 7.44 – 7.27 (m, 8H), 6.77 (s, 2H), 6.67 (s, 2H), 5.40 (s, 1H), 4.08 (s, 4H), 3.04 – 2.96 (m, 4H), 2.77 (t, *J* = 6.7 Hz, 4H), 2.28 (s, 6H), 1.84 – 1.76 (m, 4H). **^{13}C{^{1}H} NMR (101 MHz, CDCl$_3$)** δ 153.0, 146.5, 146.4, 139.8, 135.7, 131.6, 130.3, 130.0, 129.2, 128.6, 128.2, 127.6, 127.0, 123.6, 57.9, 55.8, 47.2, 28.0, 19.0, 16.9; **HRMS** (ESI-TOF) *m/z*: $[M + H]^+$ for $C_{41}H_{42}N_3O_2$ calculated 608.3272; found 608.3246.

6,6'-((4-Nitrophenyl)methylene)bis(1-benzyl-8-methoxy-1,2,3,4-tetrahydroquinoline) (14nj). General Procedure G (at 120°C), purified by silica gel chromatography with EtOAc/hexane (10:90). Yellow oil (220 mg, 86%). **^{1}H NMR (400 MHz, CDCl$_3$)** δ 8.20 – 8.14 (m, AA' part of AA'BB' system, 2H), 7.54 – 7.48 (m, 4H), 7.40 – 7.32 (m, 6H), 7.32 – 7.27 (m, 2H), 6.50 (d, *J* = 1.4 Hz, 2H), 6.40 (d, *J* = 1.4 Hz, 2H), 5.47 (s, 1H), 4.21 (s, 4H), 3.77 (s, 6H), 3.04 – 2.97 (m, 4H), 2.71 (t, *J* = 6.4 Hz, 4H), 1.85 – 1.74 (m, 4H). **^{13}C{^{1}H} NMR (101 MHz, CDCl$_3$)** δ 152.8, 152.1, 146.4, 140.4, 136.2, 135.1, 130.3, 129.2, 128.8, 128.3, 127.0, 123.5, 122.7, 110.0, 57.8, 56.2, 55.5, 47.5, 28.1, 17.9; **HRMS** (ESI-TOF) *m/z*: $[M + H]^+$ for $C_{41}H_{42}N_3O_4$ calculated 640.3170; found 640.3177.

6,6'-((4-Nitrophenyl)methylene)bis(1-benzyl-2-methyl-1,2,3,4-tetrahydroquinoline) (14oj). General Procedure G (at 90°C), purified by silica gel chromatography with EtOAc/hexane (10:90). Brown oil (216 mg, 89%). **^{1}H NMR (400 MHz, CDCl$_3$)** δ 8.16 – 8.10 (m, AA' part of AA'BB' system, 2H), 7.38 – 7.23 (m, 12H), 6.79 (s, 2H), 6.70 – 6.63 (m, A part of AB system, 2H), 6.35 (dd, *J* = 8.5, 1.7 Hz, B part of AB system, 2H), 5.34 (s, 1H), 4.62 – 4.41 (m, 4H), 3.69 – 3.53 (m, 2H), 2.96 – 2.82 (m, 2H), 2.78 – 2.65 (m, 2H), 2.13 – 2.02 (m, 2H), 1.93 – 1.81 (m, 2H), 1.22 (d, *J* = 6.2 Hz, 6H). **^{13}C{^{1}H} NMR (101 MHz, CDCl$_3$)** δ 154.0, 146.0, 143.5, 139.6, 130.2, 129.9, 129.6, 128.6, 127.8, 126.7, 126.4, 123.4, 121.7, 111.3, 55.2, 53.6, 53.5, 53.12, 53.09, 28.3, 24.2, 19.1; **HRMS** (ESI-TOF) *m/z*: $[M + H]^+$ for $C_{41}H_{42}N_3O_2$ calculated 608.3272; found 608.3264.

9,9'-((4-Nitrophenyl)methylene)bis(2,3,6,7-tetrahydro-1*H*,5*H*-pyrido[3,2,1-*ij*]quinoline) (14pj). General Procedure G, purified by silica gel chromatography with EtOAc/hexane (10:90). Yellow solid (176 mg, 92%; mp 218–219°C). **^{1}H NMR (400 MHz, $CDCl_3$)** δ 8.11 – 8.07 (m, AA' part of AA'BB' system, 2H), 7.31 – 7.28 (m, BB' part of AA'BB' system, 2H), 6.47 (s, 4H), 5.20 (s, 1H), 3.10 (t, J = 6.5 Hz, 8H), 2.67 (t, J = 6.5 Hz, 8H), 1.94 (p, J = 6.5 Hz, 8H). **$^{13}C\{^1H\}$ NMR (101 MHz, $CDCl_3$)** δ 154.0, 146.1, 141.6, 130.4, 130.3, 127.8, 123.4, 121.6, 55.5, 50.1, 27.8, 22.2; **HRMS** (ESI-TOF) *m/z*: $[M + H]^+$ for $C_{31}H_{34}N_3O_2$ calculated 480.2646; found 480.2646.

1-Benzyl-7-bromo-6-(4-nitrobenzyl)-1,2,3,4-tetrahydroquinoline (15gj). General Procedure G (at 90°C), purified by silica gel chromatography with EtOAc/hexane (10:90). Yellow oil (120 mg, 69%). **^{1}H NMR (400 MHz, $CDCl_3$)** δ 8.15 – 8.10 (m, AA' part of AA'BB' system, 2H), 7.36 – 7.31 (m, 4H), 7.29 – 7.22 (m, 3H), 6.76 (s, 1H), 6.71 (s, 1H), 4.45 (s, 2H), 4.04 (s, 2H), 3.36 – 3.30 (m, 2H), 2.71 (t, J = 6.2 Hz, 2H), 2.01 – 1.94 (m, 1H). **$^{13}C\{^1H\}$ NMR (101 MHz, $CDCl_3$)** δ 149.0, 146.5, 145.8, 138.1, 131.2, 129.6, 128.9, 127.2, 126.7, 125.0, 123.7, 123.0, 122.2, 114.5, 55.1, 49.5, 40.7, 27.9, 22.1. **HRMS** (ESI-TOF) *m/z*: $[M + H]^+$ for $C_{23}H_{22}BrN_2O_2$ calculated 437.0859; found 437.0858.

Aplications: Scale-up, transformation, late-stage functionalization

Scale-up Synthesis of 3af

A solution of indoline (**1a**; 3.64 mL, 32.4 mmol, 6 equiv.) and 4-bromobenzaldehyde (**2f**; 1g, 5.4 mmol, 1 equiv.) in 40 mL HFIP was stirred at room temperature for 24 hours. After 34 mL of HFIP was recovered by the simple distillation method the remaining solvent was removed under vacuum. The crude product was purified by column chromatography on silica gel with EtOAc/hexane (20:80) as the eluent to give the desired products **3af** (1.62g 74%).

Scale-up Synthesis of 6af

To a solution of tetrahydroquinoline (**5a**; 1.70 mL, 13.5 mmol, 2.5 equiv) and 4-bromobenzaldehyde (**2f**; 1g, 5.4 mmol, 1 equiv.) in 40 mL HFIP, TfOH (47 µL, 0.54 mmol, 0.1 eq, 10 mol%) was added. The reaction was stirred at room temperature for 24 hours. After 32 mL of HFIP was recovered by simple distillation method the remaining solvent was removed under vacuum. The solvent was then removed under vacuum. The crude product was purified by column chromatography on silica gel with EtOAc/hexane (20:80) as the eluent to give the desired products **6af** (1.85 g 79%).

5,5'-((4-Bromophenyl)methylene)bis(1*H*-indole) (16af). 5,5'-((4-Bromophenyl)methylene) diindoline (**3af**, 81 mg, 0.2 mmol) was dissolved in 4 mL of DCM, and MnO_2 (261 mg, 3 mmol, 15 equiv) was added. The mixture was stirred at room temperature for 12 hours. After completion, the reaction mixture was filtered through filter paper, and the solvent was removed under vacuum. The crude product was purified by silica gel column chromatography using EtOAc/hexane (1:9). The

compound was obtained as a white solid (72 mg, 90%; mp 103–104°C). **^{1}H NMR (400 MHz, DMSO-*d_6*)** δ 11.03 (bs, 2H), 7.50 – 7.41 (m, AA' part of AA'BB' system, 2H), 7.32 (d, *J* = 8.4 Hz, A part of AB system, 2H), 7.29 (t, *J* = 2.6 Hz, 2H), 7.20 (s, 2H), 7.13 – 7.04 (m, BB' part of AA'BB' system, 2H), 6.90 (d, *J* = 8.4 Hz, B part of AB system, 2H), 6.33 (bs, 2H), 5.71 (s, 1H). **^{13}C{^{1}H} NMR (101 MHz, DMSO-*d_6*)** δ 145.4, 134.8, 134.4, 131.4, 130.9, 127.6, 125.5, 122.8, 120.1, 118.8, 111.2, 101.0, 55.6. **HRMS** (ESI-TOF) *m/z*: $[M + H]^+$ for $C_{23}H_{18}BrN_2$ calculated 401.0648; found 401.0644.

6,6'-((4-Bromophenyl)methylene)diquinoline (17af). 6,6'-((4-Bromophenyl)methylene) bis(1,2,3,4-tetrahydroquinoline) (**16af**, 87 mg, 0.2 mmol) was dissolved in 4 mL of DCM, and MnO_2 (349 mg, 4 mmol, 20 equiv) was added. The mixture was stirred at room temperature for 12 hours. After completion, the reaction mixture was filtered through filter paper, and the solvent was removed under vacuum. The crude product was purified by silica gel column chromatography using EtOAc/hexane (1:1). The compound was obtained as a yellow solid (68 mg, 80%; mp 100–101°C). **^{1}H NMR (400 MHz, $CDCl_3$)** δ 8.91 – 8.84 (m, 2H), 8.06 (d, *J* = 8.8 Hz, 2H), 8.01 (d, *J* = 8.2 Hz, 2H), 7.54 (dd, *J* = 8.7, 1.9 Hz, 2H), 7.47 – 7.40 (m, 4H), 7.35 (dd, *J* = 8.3, 4.2 Hz, 2H), 7.05 (d, *J* = 8.5 Hz, 2H), 5.86 (s, 1H). **^{13}C{^{1}H} NMR (101 MHz, $CDCl_3$)** δ 150.4, 147.2, 141.7, 141.2, 136.2, 131.8, 131.5, 131.3, 129.7, 128.2, 127.7, 121.5, 121.0, 56.0. **HRMS** (ESI-TOF) *m/z*: $[M + H]^+$ for $C_{25}H_{18}BrN_2$ calculated 425.0648; found 425.0643.

Late-stage functionalization

Methyl (4-(bis(1,2,3,4-tetrahydroquinolin-6-yl)methyl)benzoyl)tryptophanate (18). The compound was synthesized to General Procedure F. light yellow solid (177 mg, 74%; mp 152–153 °C). **^{1}H NMR (400 MHz, Acetone-*d_6*)** δ 10.07 (bs, 1H), 7.77 – 7.66 (m, 3H), 7.62 (d, *J* = 7.6 Hz, 1H), 7.36 (d, *J* = 7.6 Hz, 1H), 7.26 (s, 1H), 7.21 – 7.13 (m, BB' part of AA'BB' system, 2H), 7.09 (t, *J* = 7.6 Hz, 1H), 7.02 (t, *J* = 7.6 Hz, 1H), 6.68 – 6.55 (m, 4H), 6.38 (d, *J* = 7.9 Hz, B part of AB system, 2H), 5.21 (s, 1H), 4.97 (bs, 1H), 4.37 (bs, 2H), 3.66 (s, 3H), 3.50 – 3.28 (m, 2H), 3.28 – 3.12 (m, 4H), 2.60 (t, *J* = 5.5 Hz, 4H), 1.89 – 1.74 (m, 4H). **^{13}C{^{1}H} NMR (101 MHz, Acetone-*d_6*)** δ 173.3, 150.9, 144.4, 137.5, 132.5, 130.7, 129.9, 128.5, 128.4, 128.2, 128.1, 127.8, 124.4, 122.2, 121.1, 119.6, 119.1, 114.5, 112.3, 111.0, 56.1, 54.5, 52.3, 42.3, 28.1, 27.8, 22.9. **HRMS** (ESI-TOF) *m/z*: $[M + H]^+$ for $C_{38}H_{39}N_4O_3$ calculated 599.3017; found 599.3013.

10,13-Dimethyl-17-(6-methylheptan-2-yl)-2,3,4,7,8,9,10,11,12,13,14,15,16,17-tetradecahydro-1*H*-cyclopenta[*a*]phenanthren-3-yl 4-(bis(1,2,3,4-tetrahydroquinolin-6-yl)methyl)benzoate (19). General Procedure F, purified by silica gel chromatography with EtOAc/hexane (20:80). Light yellow solid (218 mg, 71%; mp 157–158 °C).**^{1}H NMR (400 MHz, $CDCl_3$)** δ 7.97 – 7.88 (m, AA' part of AA'BB' system, 2H), 7.24 – 7.16 (m, BB' part of AA'BB' system, 2H), 6.71 – 6.63 (m, 4H), 6.42 (d, *J* = 7.9 Hz, B part of AB system, 2H), 5.42 (d, *J* = 3.8 Hz, 1H), 5.28 (s, 1H), 4.91 – 4.77 (m, 1H),

3.64 (bs, 2H), 3.34 – 3.19 (m, 4H), 2.68 (t, *J* = 6.3 Hz, 4H), 2.45 (d, *J* = 7.8 Hz, 2H), 2.07 – 1.80 (m, 10H), 1.78 – 1.66 (m, 1H), 1.64 – 1.44 (m, 7H), 1.42 – 1.09 (m, 14H), 1.07 (s, 3H), 1.04 – 0.96 (m, 3H), 0.93 (d, *J* = 6.5 Hz, 3H), 0.88 (dd, *J* = 6.6, 1.6 Hz, 6H), 0.70 (s, 3H). **$^{13}C\{^1H\}$ NMR (101 MHz, $CDCl_3$)** δ 166.2, 151.0, 142.7, 139.8, 132.9, 130.4, 129.5, 129.4, 128.5, 127.7, 122.8, 121.7, 114.5, 74.4, 56.8, 56.2, 55.6, 50.2, 42.4, 42.2, 39.9, 39.6, 38.4, 37.2, 36.8, 36.3, 35.9, 32.1, 32.0, 28.4, 28.1, 28.0, 27.0, 24.4, 24.0, 23.0, 22.7, 22.3, 21.2, 19.5, 18.8, 12.0. **HRMS** (ESI-TOF) *m/z*: $[M + H]^+$ for $C_{53}H_{71}N_2O_2$ calculated 767.5510; found 767.5492.

2-Isopropyl-5-methylcyclohexyl 4-(bis(1,2,3,4-tetrahydroquinolin-6-yl)methyl)benzoate (20). General Procedure F, purified by silica gel chromatography with EtOAc/hexane (20:80). Light yellow solid (165 mg, 77%; mp 101–102 °C). **^1H NMR (400 MHz, $CDCl_3$)** δ 8.37 – 7.77 (m, AA' part of AA'BB' system, 2H), 7.24 – 7.17 (m, BB' part of AA'BB' system, 2H), 6.73 – 6.63 (m, 4H), 6.40 (d, *J* = 8.0 Hz, B part of AB system, 2H), 5.29 (s, 1H), 4.92 (td, *J* = 10.8, 4.3 Hz, 1H), 3.64 (bs, 2H), 3.33 – 3.22 (m, 4H), 2.69 (t, *J* = 6.3 Hz, 4H), 2.12 (d, *J* = 11.8 Hz, CH, 1H), 2.01 – 1.86 (m, 5H), 1.73 (d, *J* = 11.1 Hz, 2H), 1.63 – 1.48 (m, 2H), 1.19 – 1.02 (m, 2H), 0.93 (d, *J* = 6.4 Hz, 3H), 0.92 (d, *J* = 6.4 Hz, 3H), 0.79 (d, *J* = 6.9 Hz, 3H). **$^{13}C\{^1H\}$ NMR (101 MHz, $CDCl_3$)** δ 166.3, 151.0, 143.0, 132.7, 130.4, 129.5, 129.4, 128.4, 127.7, 121.5, 114.3, 74.6, 55.6, 47.4, 42.2, 41.1, 34.4, 31.6, 27.1, 26.5, 23.7, 22.3, 22.2, 20.9, 16.6. **HRMS** (ESI-TOF) *m/z*: $[M + H]^+$ for $C_{36}H_{45}N_2O_2$ calculated 537.3476; found 537.3471.

Control experiments

3-(Indolin-1-yl)isobenzofuran-1(3*H*)-one (22). General Procedure D, purified by silica gel chromatography with EtOAc/hexane (20:80): indoline (**2a**; 1 mmol, 2.5 equiv) and aldehyde (**21**; 0.4 mmol, 1 equiv). White solid (57 mg, 90%; mp 187–188 °C). **^1H NMR (400 MHz, $CDCl_3$)** δ 7.96 (d, *J* = 7.9 Hz, 1H), 7.79 – 7.73 (m, 1H), 7.64 (t, *J* = 6.8 Hz, 2H), 7.18 – 7.10 (m, 2H), 7.07 (s, 1H), 6.84 (t, *J* = 7.3 Hz, 1H), 6.79 (d, *J* = 8.0 Hz, 1H), 3.26 – 3.09 (m, 2H), 3.06 – 2.91 (m, 2H). **$^{13}C\{^1H\}$ NMR (101 MHz, $CDCl_3$)** δ 169.4, 148.1, 144.7 (2C), 134.4, 130.6, 128.1, 127.4, 125.7, 125.2, 123.6, 120.7, 108.8, 89.2, 46.3, 28.0. **HRMS** (ESI-TOF) *m/z*: $[M + H]^+$ for $C_{16}H_{14}NO_2$ calculated 252.1019; found 252.1020.

3-(3,4-Dihydroquinolin-1(2*H*)-yl)isobenzofuran-1(3*H*)-one (23). General Procedure E, purified by silica gel chromatography with EtOAc/hexane (20:80). Yellow solid (98 mg, 92%; mp 108–109°C). **^1H NMR (400 MHz, $CDCl_3$)** δ 7.97 (d, *J* = 7.5 Hz, 1H), 7.74 (td, *J* = 7.5, 1.0 Hz, 1H), 7.63 (t, *J* = 7.5 Hz, 1H), 7.52 (d, *J* = 7.5 Hz, 1H), 7.22 – 7.16 (m, 3H), 7.09 (d, *J* = 7.4 Hz, 1H), 6.92 – 6.85 (m, 1H), 2.87 (t, *J* = 6.0 Hz, 2H), 2.84 – 2.71 (m, 2H), 2.00 – 1.86 (m, A part of AB system, 1H), 1.84 – 1.73 (m, B part of AB system, 1H). **$^{13}C\{^1H\}$ NMR (101 MHz, $CDCl_3$)** δ 169.6, 144.7, 143.6, 134.4, 130.5,

129.5, 128.7, 127.3, 126.5, 125.9, 123.4, 120.4, 114.0, 92.2, 42.4, 27.6, 22.8. **HRMS** (ESI-TOF) *m/z*: $[M + H]^+$ for $C_{17}H_{16}NO_2$ calculated 266.1176; found 266.1175.

4. The details of DFT computations

All density functional theory (DFT) computations were carried out using the unrestricted method with the Gaussian 16 package.[27] Geometry optimizations were performed at the UM06-2X[28] level of theory, including Grimme's D3 dispersion correction[29] and the 6-31++G(d,p) basis set.[30,31] Solvent effects were accounted for using the SMD solvation model.[32,33]

Since HFIP is not available as a predefined solvent in Gaussian 16, its parameters were manually defined based on literature values.[34] The dielectric constant (ε) at 298 K was set to 16.7; the square of the refractive index (EpsInf, n^2) to 1.625625; hydrogen bond acidity (α) and basicity (β) to 0.77 and 0.10, respectively; surface tension at the liquid–air interface (γ) to 23.23 cal·mol^{-1}·Å^{-2}; aromatic carbon fraction (ϕ) to 0.00, as HFIP contains no aromatic carbon atoms; and the fraction of electronegative halogenic atoms (ψ) to 0.60, based on 6 fluorine atoms out of 10 non-hydrogenic atoms.

Mulliken atomic charges were computed ulated for the optimized structures of representative indoline (on the left) and tetrahydroquinoline (THQ, on the right) derivatives at the same level of theory.

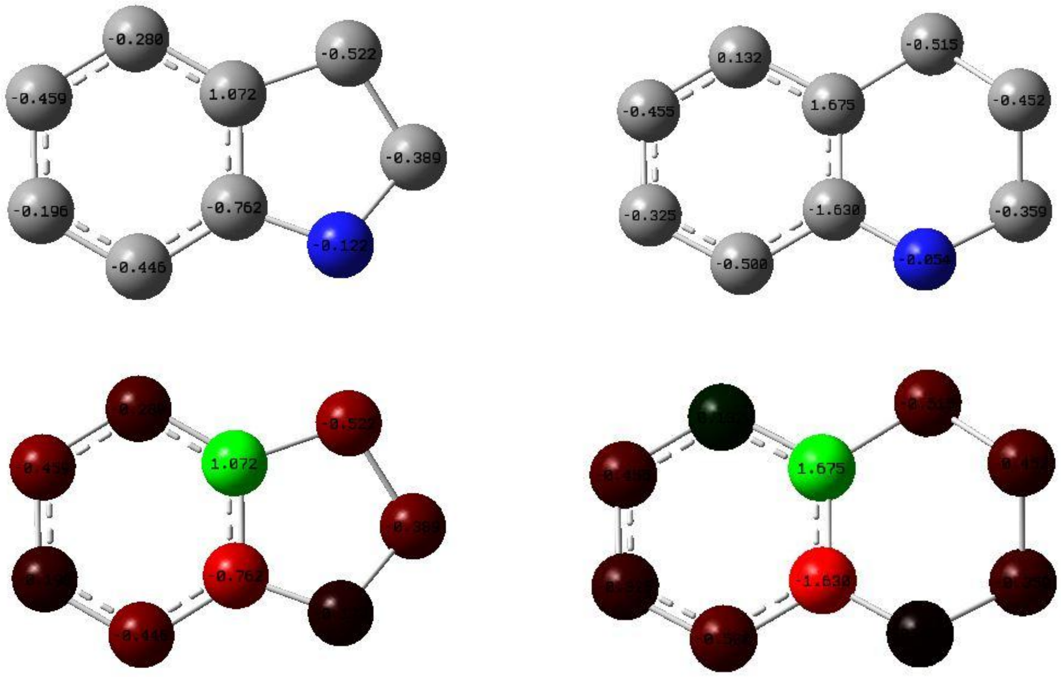

5. References

(1) Zhang, W.; Xu, G.; Qiu, L.; Sun, J. Gold-Catalyzed C5-Alkylation of Indolines and Sequential Oxidative Aromatization: Access to C5-Functionalized Indoles. *Org. Biomol. Chem.* **2018**, *16*, 3889–3892.

(2) Taskesenligil, Y.; Saracoglu, N. Dehydrogenative Photocyclization of 3-Styryl Indoles to Fused Indole Systems. *J. Org. Chem.* **2024**, *89*, 17447–17452.

(3) Bhatt, T.; Suman, V.; Choudhary, M.; Singh, S. K.; Natte, K. Dearomative Selective Reduction of Structurally Diverse N-Heteroarenes Enabled by a Homogeneous Titanium Catalyst. *J. Catal.* **2025**, *443*, 115937.

(4) Zhou, B.; Chandrashekhar, V. G.; Ma, Z.; Kreyenschulte, C.; Bartling, S.; Lund, H.; Beller, M.; Jagadeesh, R. V. Development of a General and Selective Nanostructured Cobalt Catalyst for the Hydrogenation of Benzofurans, Indoles, and Benzothiophenes. *Angew. Chem. Int. Ed.* **2023**, *62*, e202215699.

(5) Zhang, J.; Chen, Z.; Chen, M.; Zhou, Q.; Zhou, R.; Wang, W.; Zhang, F. Lanthanide/B(C_6F_5)$_3$-Promoted Hydroboration Reduction of Indoles and Quinolines with Pinacolborane. *J. Org. Chem.* **2024**, *89*, 887–897.

(6) Zhou, H. S.; Hu, L. B.; Zhang, H.; Shan, W. X.; Wang, Y.; Li, X.; Jiang, Z. Y. Design, Synthesis, and Structure–Activity Relationships of Indoline-Based Kelch-like ECH-Associated Protein 1-Nuclear Factor (Erythroid-Derived 2)-Like 2 (Keap1-Nrf2) Protein–Protein Interaction Inhibitors. *J. Med. Chem.* **2020**, *63*, 11149–11168.

(7) Nad, P.; Mukherjee, A. A Lewis Acid-Base Pair Catalyzed Dearomative Transformation of Unprotected Indoles via B−H Bond Activation. *Chem. Asian J.* **2023**, *18*, e202300714.

(8) Zeng, Y. F.; Li, Y. N.; Zhou, M. X.; Han, S.; Guo, Y.; Wang, Z. Metal-Free Hydrogenation of N-Heterocycles with Trimethylamine Borane and TFA in Aqueous Solution. *Adv. Synth. Catal.* **2022**, *364*, 3664–3669.

(9) Calogero, F.; Allegrini, P.; Attolino, E.; Passarella, D. Synthesis of Silodosin by Copper-Catalysed C–C Arylation. *Eur. J. Org. Chem.* **2015**, *2015*, 6011–6016.

(10) Timelthaler, D.; Topf, C. Heterogeneous Hydrogenation of Quinoline Derivatives Effected by a Granular Cobalt Catalyst. *Synthesis* **2022**, *54*, 629–642.

(11) Cui, X.; Huang, W.; Wu, L. Zirconium-Hydride-Catalyzed Transfer Hydrogenation of Quinolines and Indoles with Ammonia Borane. *Org. Chem. Front.* **2021**, *8*, 5002–5007.

(12) Zhu, M.; Tian, H.; Chen, S.; Xue, W.; Wang, Y.; Lu, H.; Li, T.; Chen, F.; Tang, C. Homogeneous Cobalt Catalyzed Reductive Formylation of N-Heteroarenes with Formic Acid. *J. Catal.* **2022**, *416*, 170–175.

(13) Ouyang, L.; Xia, Y.; Liao, J.; Miao, R.; Yang, X.; Luo, R. Iridium Complex-Catalyzed Transfer Hydrogenation of N-Heteroarenes and Tentative Asymmetric Synthesis. *ACS Omega* **2021**, *6*, 10415–10427.

(14) Mandal, A.; Pradhan, M.; Maji, A.; Debnath, R.; Kundu, S. Nanoparticles of Cobalt for the Reversible (De)Hydrogenation and Oxidative Dehydrogenation of *N*-Heterocycles under Mild Conditions. *ACS Appl. Nano Mater.* **2024**, *7*, 10182–10193.

(15) Maji, M.; Borthakur, I.; Srivastava, S.; Kundu, S. Regio-Selective C3- and N-Alkylation of Indolines in Water under Air Using Alcohols. *J. Org. Chem.* **2022**, *87*, 5603–5616.

(16) Lu, J.; Xu, R.; Zeng, H.; Zhong, G.; Wang, M.; Ni, Z.; Zeng, X. Synthesis of C5-Allylindoles through an Iridium-Catalyzed Asymmetric Allylic Substitution/Oxidation Reaction Sequence of N-Alkyl Indolines. *Org. Lett.* **2021**, *23*, 3426–3431.

(17) Kaga, A.; Hayashi, H.; Hakamata, H.; Oi, M.; Uchiyama, M.; Takita, R.; Chiba, S. Nucleophilic Amination of Methoxy Arenes Promoted by a Sodium Hydride/Iodide Composite. *Angew. Chem. Int. Ed.* **2017**, *56*, 11807–11811.

(18) Sukowski, V.; van Borselen, M.; Mathew, S.; de Bruin, B.; Fernández-Ibáñez, M. Á. meta-C−H Arylation of Aniline Derivatives via Palladium/S, O-Ligand/Norbornene Cooperative Catalysis. *Angew. Chem.* **2024**, *136*, e202317741.

(19) Yadav, S.; Chaudhary, D.; Maurya, N. K.; Kumar, D.; Ishu, K.; Kuram, M. R. Transfer Hydrogenation of Pyridinium and Quinolinium Species Using Ethanol as a Hydrogen Source to Access Saturated N-Heterocycles. *Chem. Commun.* **2022**, *58*, 4255–4258.

(20) Adhikari, P.; Bhattacharyya, D.; Nandi, S.; Kancharla, P. K.; Das, A. Reductive Alkylation of Quinolines to N-Alkyl Tetrahydroquinolines Catalyzed by Arylboronic Acid. *Org. Lett.* **2021**, *23*, 2437–2442.

(21) Li, F. Y.; Xiao, Y.; Huang, D. W.; Xu, H.; Wang, B.; Wang, J. Y. Direct Dearomatization of Quinoline/Isoquinoline Ammonium Halides to Construct N-Substituted Tetrahydroquinolines and Tetrahydroisoquinolines. *Chem. Select* **2024**, *9*, e202304934.

[22] Konwar, M.; Das, T.; Das, A. Cyclometalated Ruthenium Catalyst Enables Selective Oxidation of N-Substituted Tetrahydroquinolines to Lactams. *Org. Lett.* **2024**, *26*, 1184–1189.

(23) Bosset, C.; Angibaud, P.; Stanfield, I.; Meerpoel, L.; Berthelot, D.; Guerinot, A.; Cossy, J. Iron-Catalyzed Synthesis of C2 Aryl- and N-Heteroaryl-Substituted Tetrahydropyrans. *J. Org. Chem.* **2015**, *80*, 12509–12525.

(24) Li, Z. Z.; Jiang, S. J.; He, S. Y.; Gao, Y. N.; Bian, M.; Chen, H. Y.; Liu, Z. J. Synthesis of 2-Acyl Benzofurans and Indoles Based on Nucleophile-Intercepted Meyer–Schuster Rearrangement of o-Hydroxyphenyl and o-Aminophenyl Propargylic Alcohols. *Org. Chem. Front.* **2024**, *11*, 809–815.

(25) Sakata, Y.; Yasui, E.; Takatori, K.; Suzuki, Y.; Mizukami, M.; Nagumo, S. Syntheses of Polycyclic Tetrahydrofurans by Cascade Reactions Consisting of Five-Membered Ring Selective Prins Cyclization and Friedel–Crafts Cyclization. *J. Org. Chem.* **2018**, *83*, 9103–9118.

(26) Cavaca, L. A.; Coelho, J. A.; Lucas, S. D.; Loureiro, R. M.; Gomes, R. F.; Afonso, C. A. Upgrading Furanic Platforms to α-Enaminones: Tunable Continuous Flow Hydrogenation of Bio-Based Cyclopentenones. *React. Chem. Eng.* **2023**, *8*, 482–489.

(27) Frisch, M. J.; Trucks, G. W.; Schlegel, H. B.; Scuseria, G. E.; Robb, M. A.; Cheeseman, J. R.; Scalmani, G.; Barone, V.; Petersson, G. A.; Nakatsuji, H.; Li, X.; Caricato, M.; Marenich, A. V.; Bloino, J.; Janesko, B. G.; Gomperts, R.; Mennucci, B.; Hratchian, H. P.; Ortiz, J. V.; Izmaylov, A. F.; Sonnenberg, J. L.; Williams-Young, D.; Ding, F.; Lipparini, F.; Egidi, F.; Goings, J.; Peng, B.; Petrone, A.; Henderson, T.; Ranasinghe, D.; Zakrzewski, V. G.; Gao, J.; Rega, N.; Zheng, G.; Liang, W.; Hada, M.; Ehara, M.; Toyota, K.; Fukuda, R.; Hasegawa, J.; Ishida, M.; Nakajima, T.; Honda, Y.; Kitao, O.; Nakai, H.; Vreven, T.; Throssell, K.; Montgomery, J. A., Jr.; Peralta, J. E.; Ogliaro, F.; Bearpark, M. J.; Heyd, J. J.; Brothers, E. N.; Kudin, K. N.; Staroverov, V. N.; Keith, T. A.; Kobayashi, R.; Normand, J.; Raghavachari, K.; Rendell, A. P.; Burant, J. C.; Iyengar, S. S.; Tomasi, J.; Cossi, M.; Millam, J. M.; Klene, M.; Adamo, C.; Cammi, R.; Ochterski, J. W.; Martin, R. L.; Morokuma, K.; Farkas, O.; Foresman, J. B.; Fox, D. J. Gaussian 16, Revision C.01; Gaussian, Inc.: Wallingford, CT, **2016**.

(28) Zhao, Y.; Truhlar, D. G. The M06 Suite of Density Functionals for Main Group Thermochemistry, Thermochemical Kinetics, Noncovalent Interactions, Excited States, and Transition Elements: Two New Functionals and Systematic Testing of Four M06-Class Functionals and 12 Other Functionals. *Theor. Chem. Acc.* **2008**, *120*, 215–241.

(29) Hariharan, P. C.; Pople, J. A. The Influence of Polarization Functions on Molecular Orbital Hydrogenation Energies. *Theor. Chim. Acta* **1973**, *28*, 213–222.

(30) Krishnan, R. B. J. S.; Binkley, J. S.; Seeger, R.; Pople, J. A. Self-Consistent Molecular Orbital Methods. XX. A Basis Set for Correlated Wave Functions. *J. Chem. Phys.* **1980**, *72*, 650–654.

(31) McLean, A. D.; Chandler, G. S. Contracted Gaussian Basis Sets for Molecular Calculations. I. Second Row Atoms, Z = 11–18. *J. Chem. Phys.* **1980**, *72*, 5639–5648.

(32) Zhao, Y.; Truhlar, D. G. The M06 Suite of Density Functionals for Main Group Thermochemistry, Thermochemical Kinetics, Noncovalent Interactions, Excited States, and Transition Elements: Two New Functionals and Systematic Testing of Four M06-Class Functionals and 12 Other Functionals. *Theor. Chem. Acc.* **2008**, *120*, 215–241.

(33) Marenich, A. V.; Cramer, C. J.; Truhlar, D. G. Universal Solvation Model Based on Solute Electron Density and on a Continuum Model of the Solvent Defined by the Bulk Dielectric Constant and Atomic Surface Tensions. *J. Phys. Chem. B* **2009**, *113*, 6378–6396.

(34) To, T. A.; Phan, N. T.; Mai, B. K.; Nguyen, T. V. Controlling the Regioselectivity of the Bromolactonization Reaction in HFIP. *Chem. Sci.* **2024**, *15*, 7187–7197.

6. NMR Spectra of Starting Materials and Products

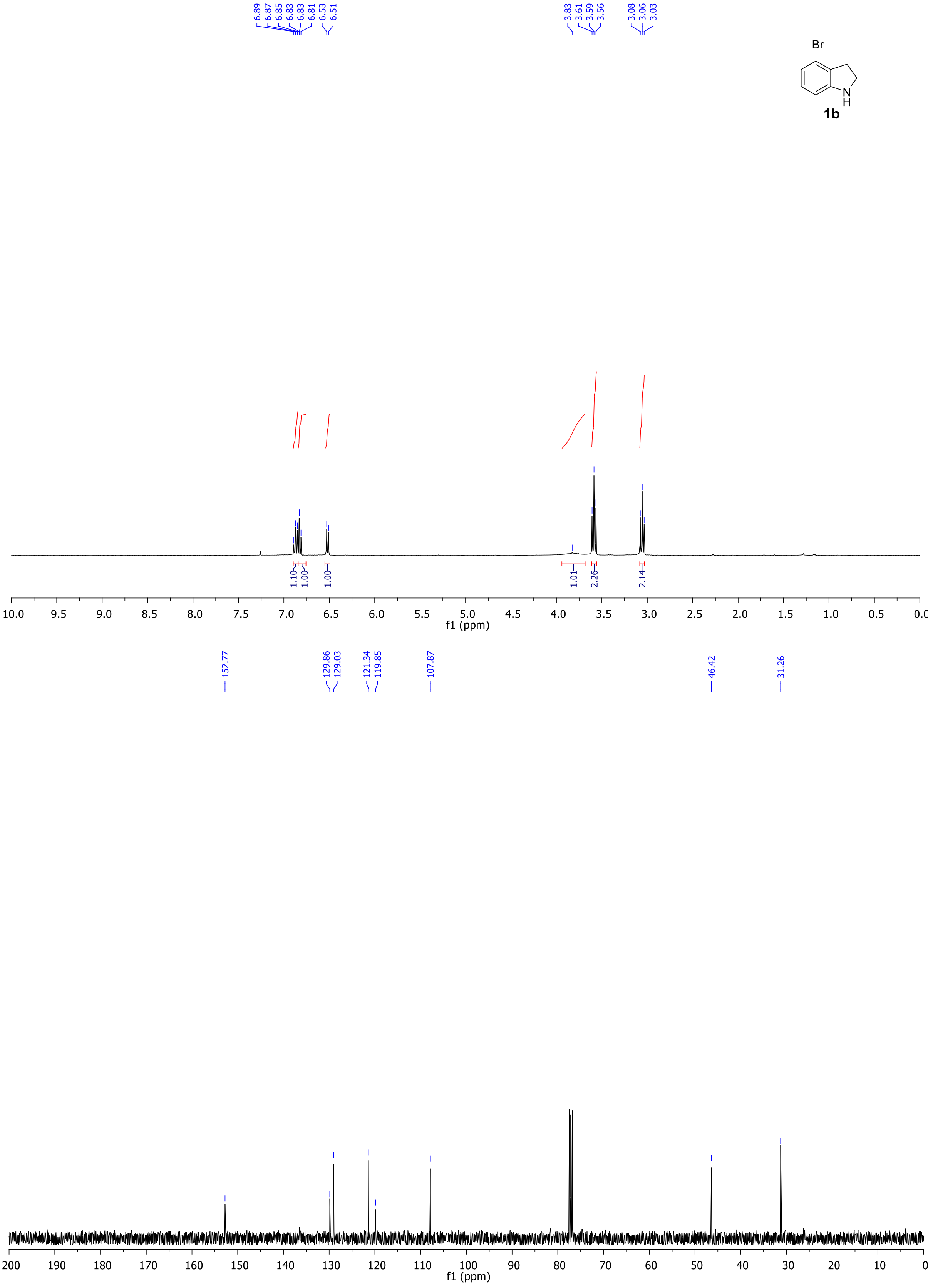

^{1}H NMR (400 MHz) and ^{13}C{^{1}H} NMR (100 MHz) spectra of **1b** ($CDCl_3$)

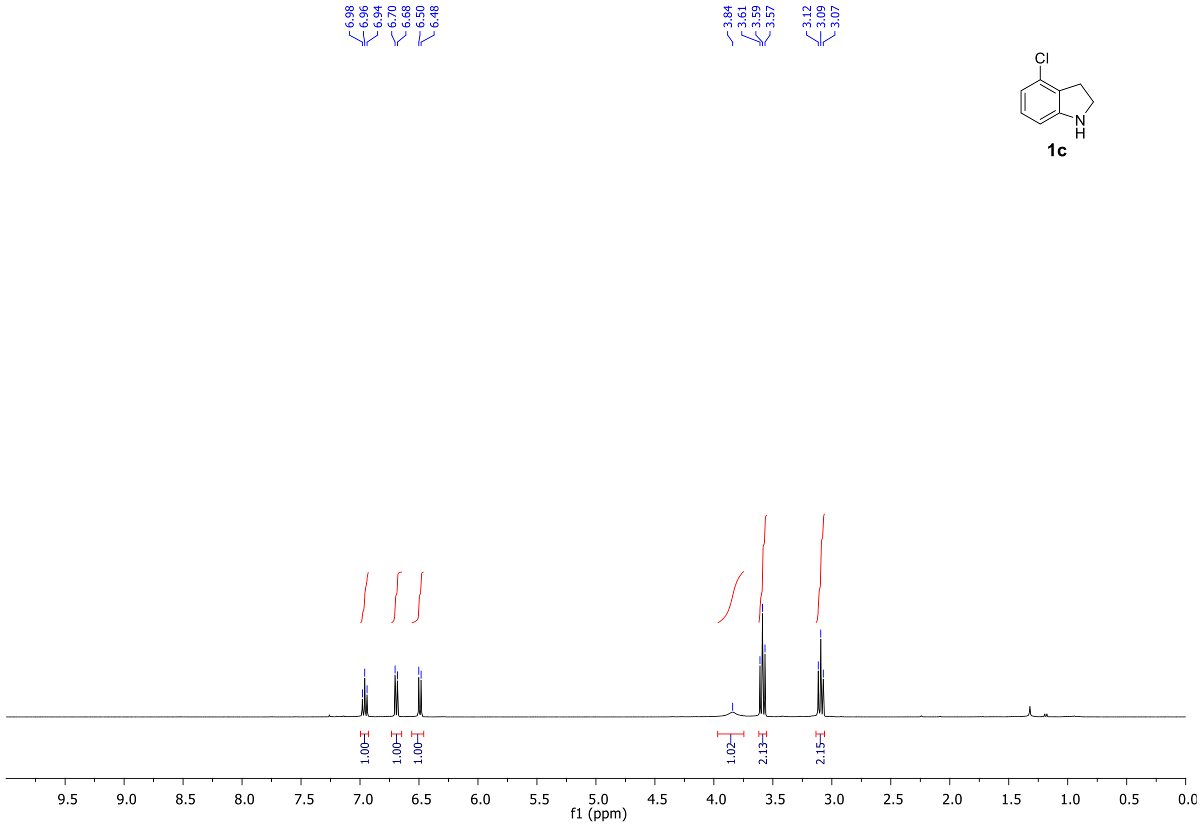

1c

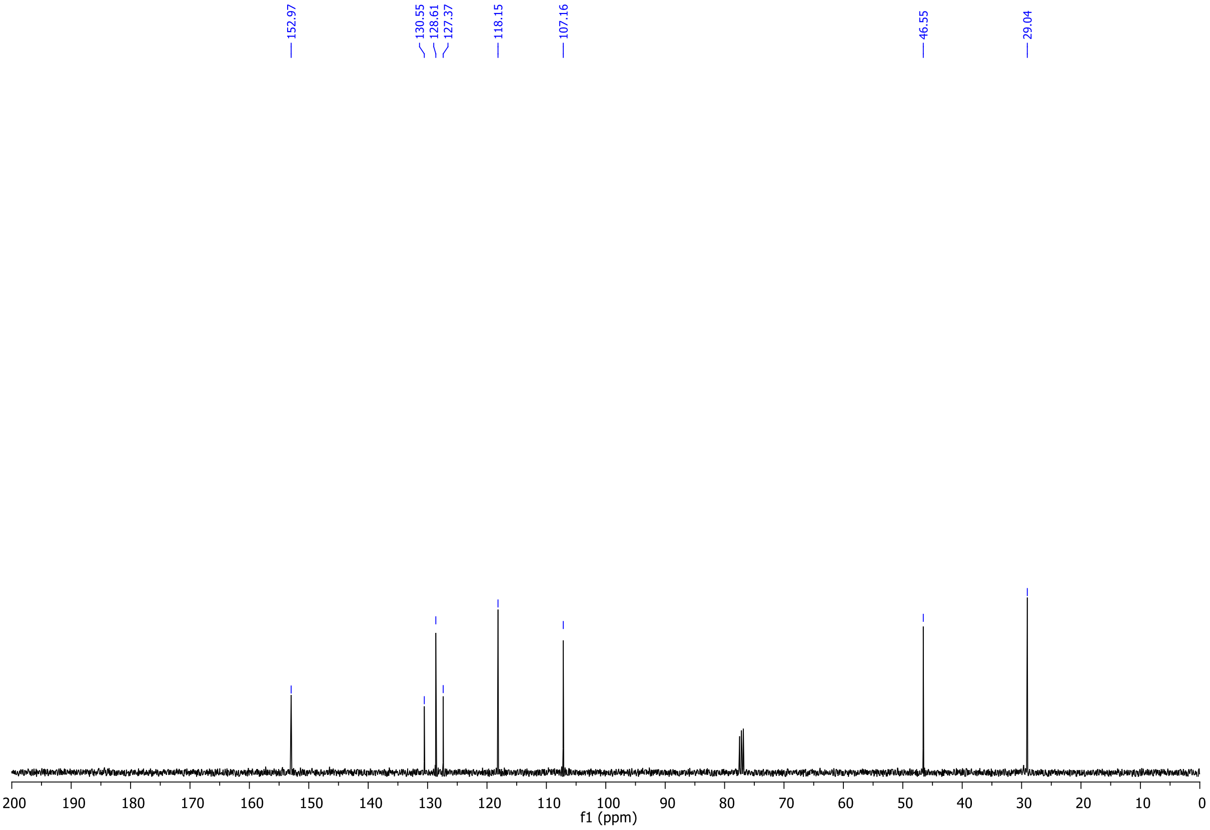

^{1}H NMR (400 MHz) and ^{13}C{^{1}H} NMR (100 MHz) spectra of **1c** ($CDCl_3$)

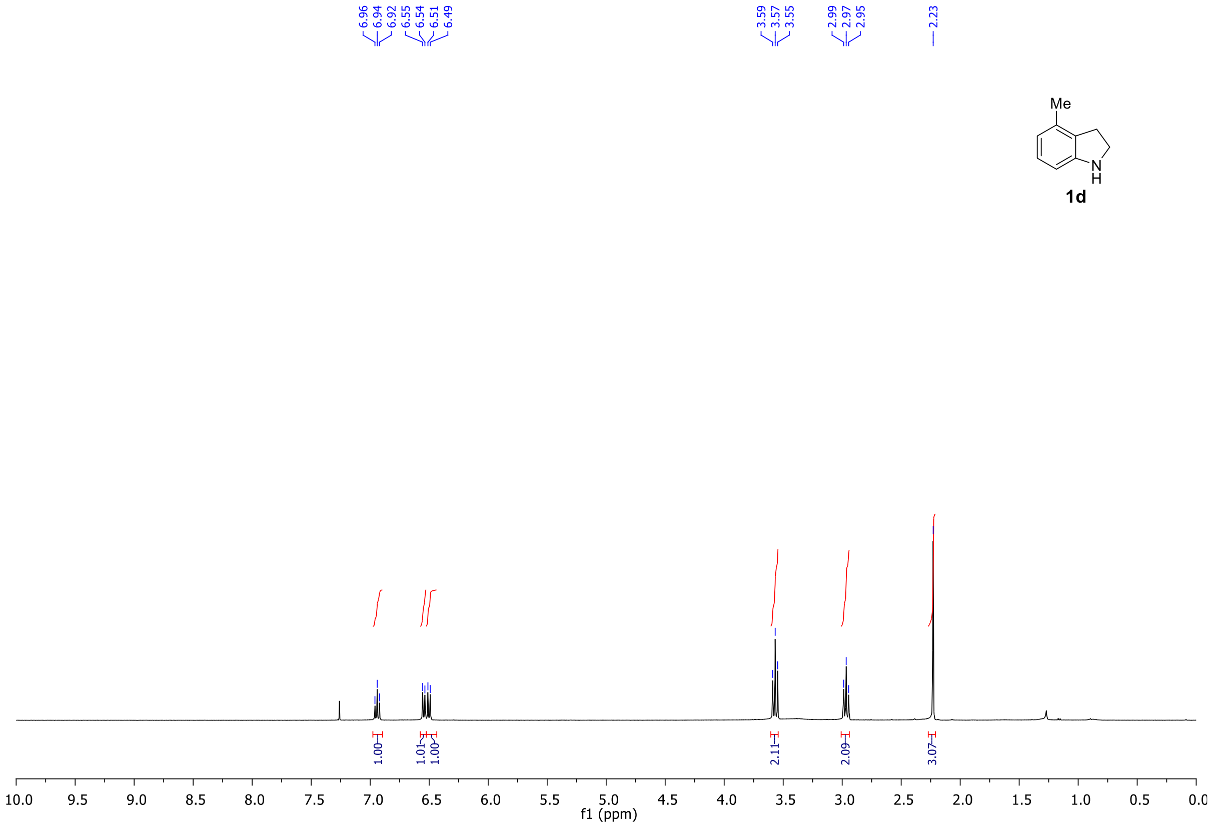

1d

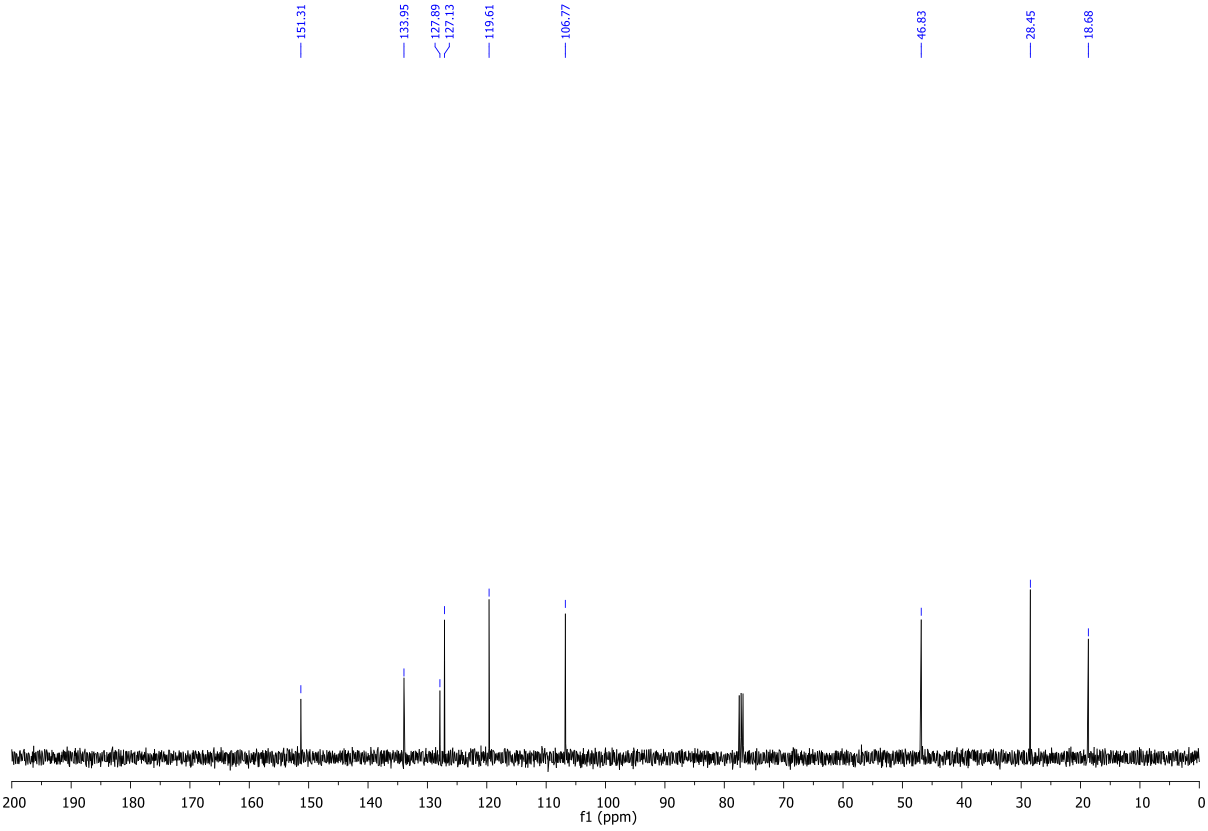

^{1}H NMR (400 MHz) and $^{13}C\{^{1}H\}$ NMR (100 MHz) spectra of **1d** ($CDCl_3$)

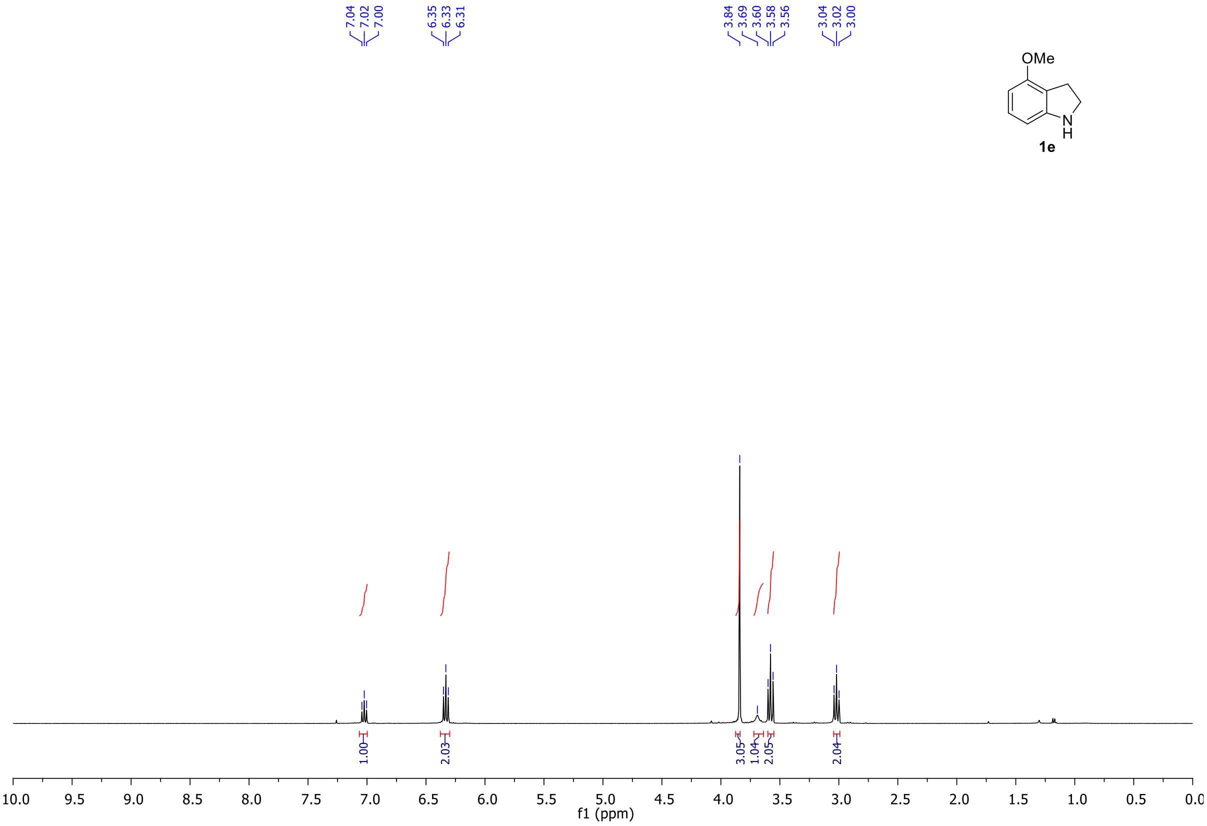

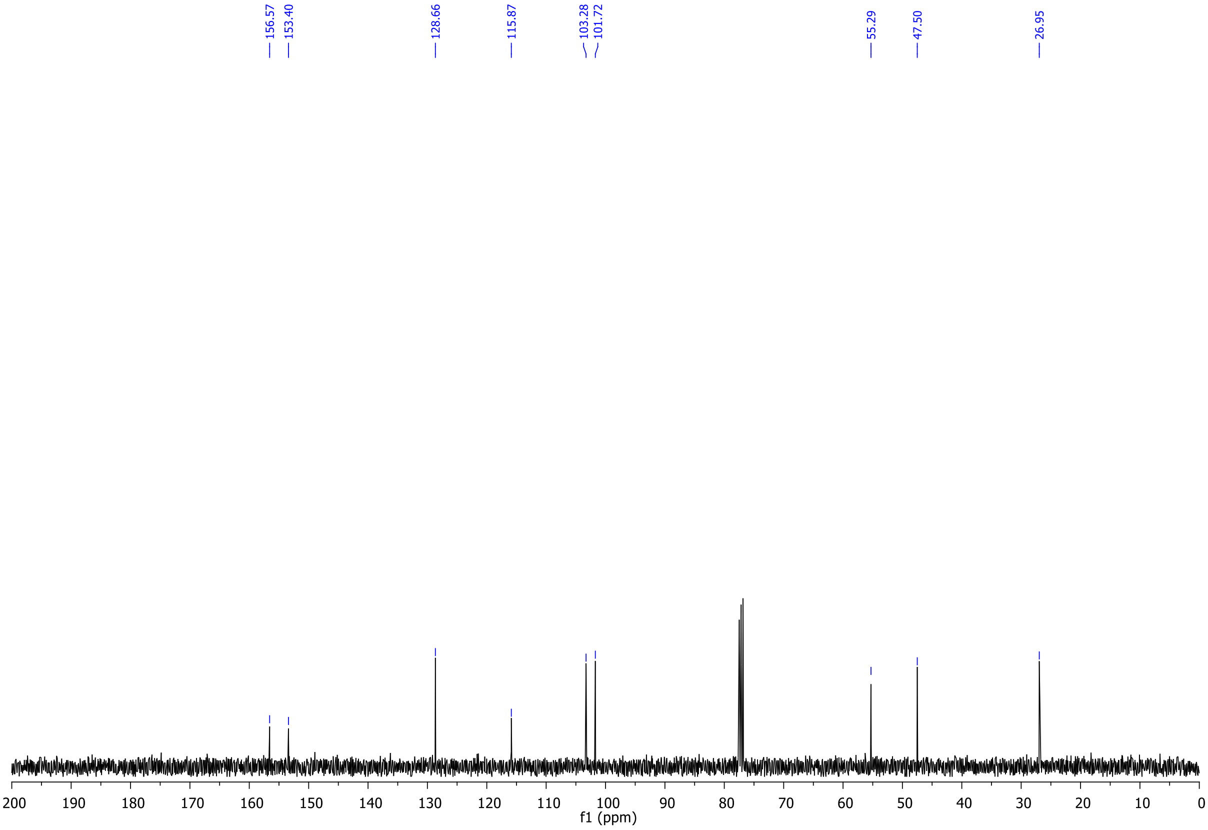

^{1}H NMR (400 MHz) and ^{13}C{^{1}H} NMR (100 MHz) spectra of **1e** ($CDCl_3$)

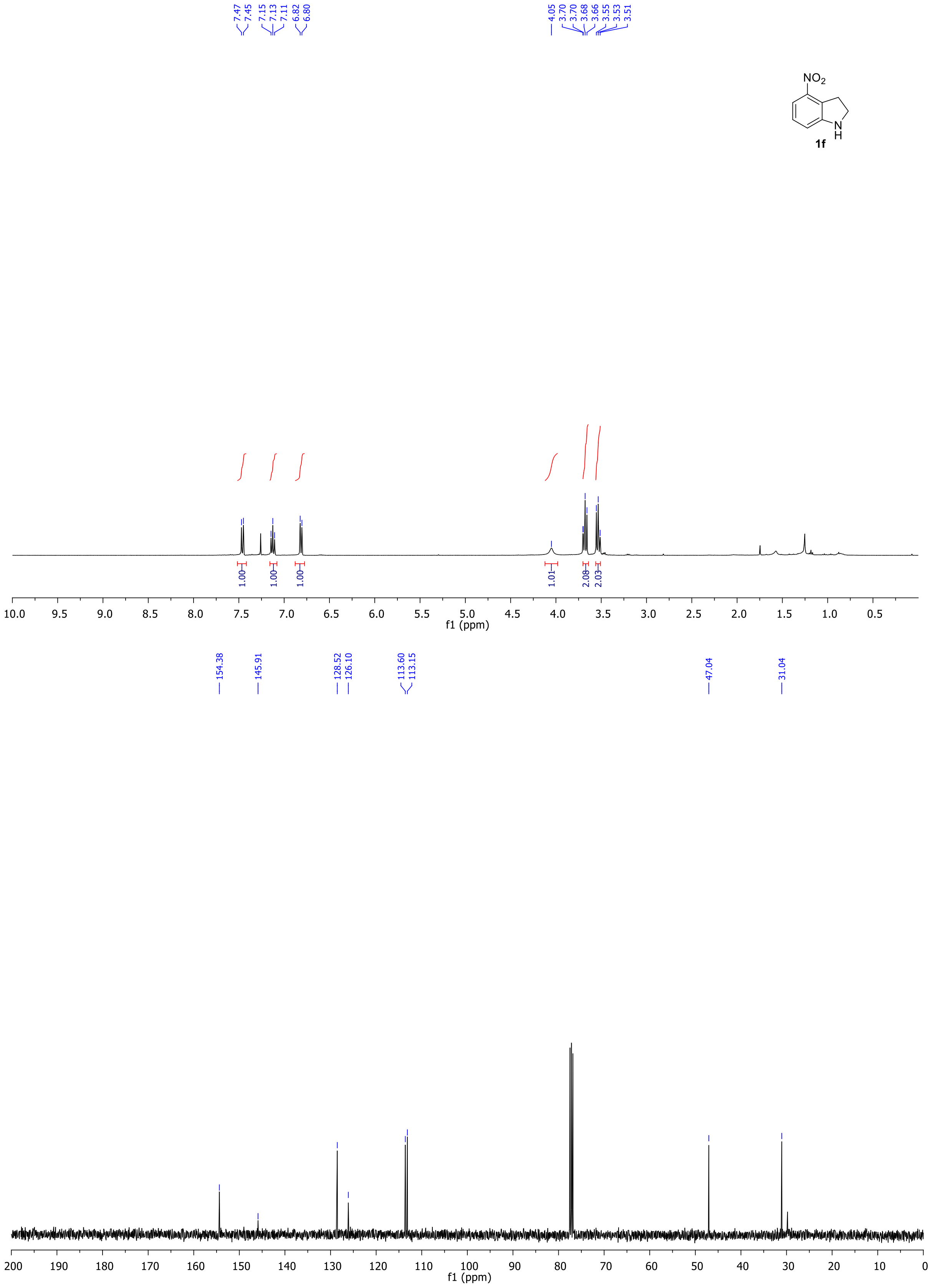

^{1}H NMR (400 MHz) and $^{13}C\{^{1}H\}$ NMR (100 MHz) spectra of **1f** ($CDCl_3$)

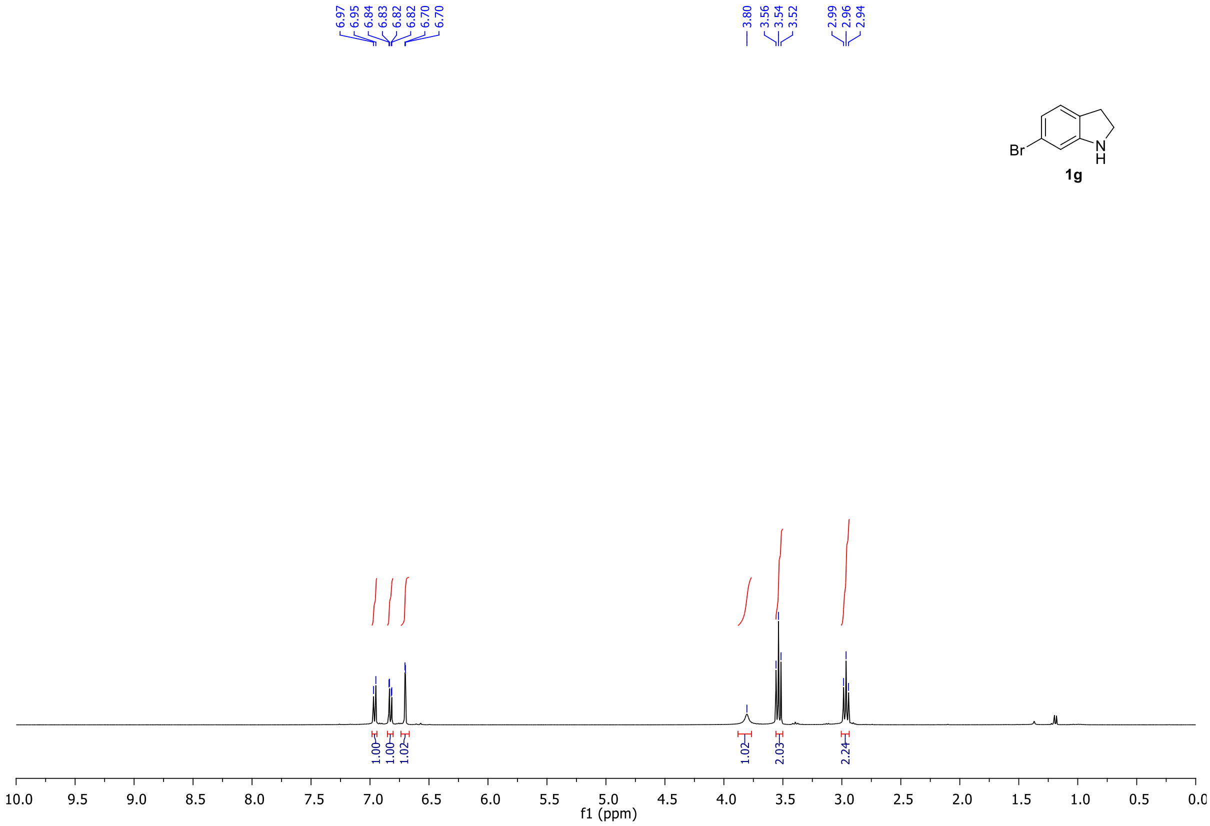

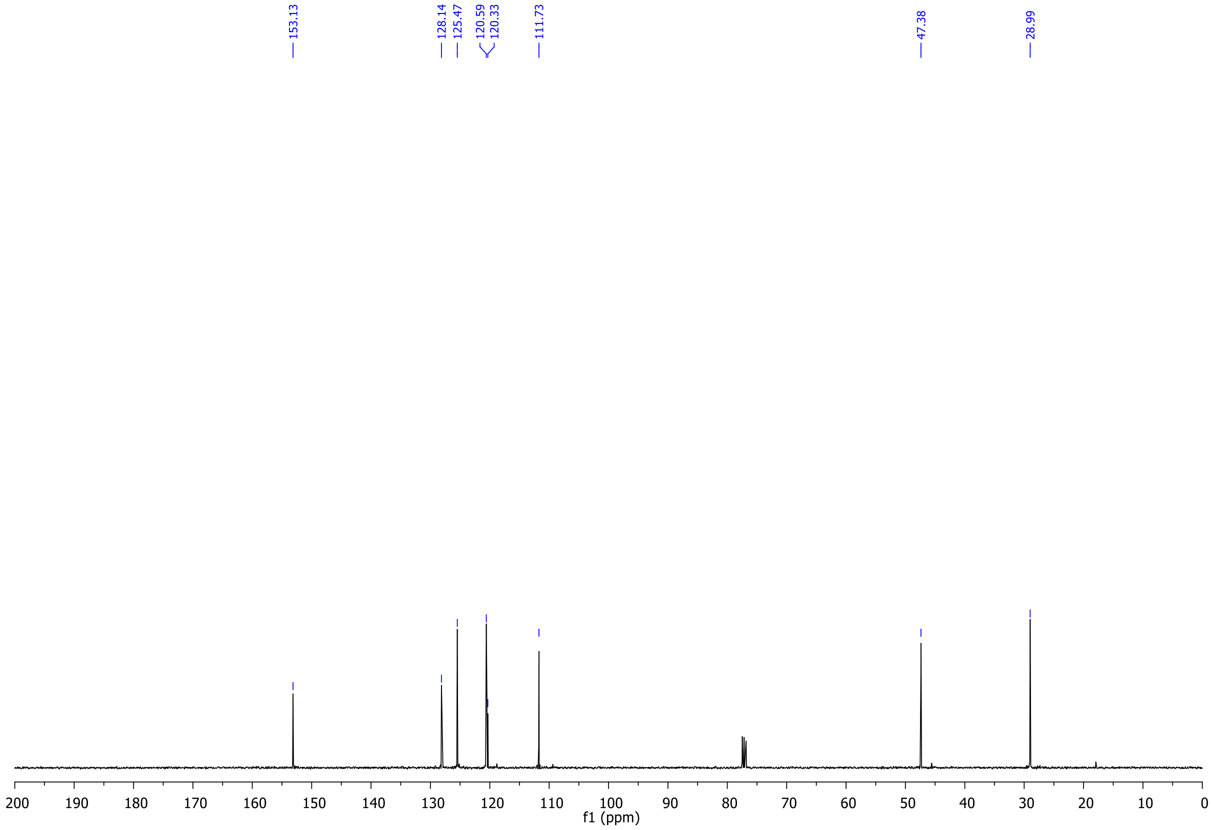

^1H NMR (400 MHz) and $^{13}C\{^1H\}$ NMR (100 MHz) spectra of **1g** ($CDCl_3$)

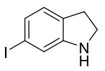

1h

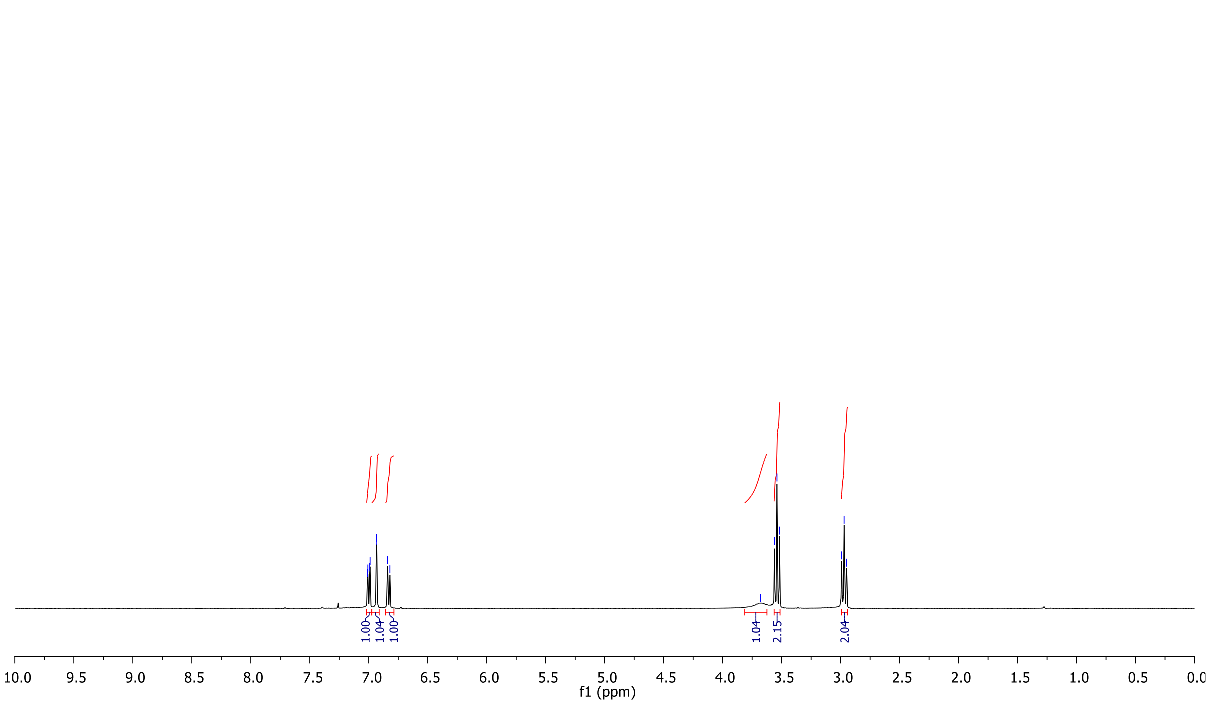

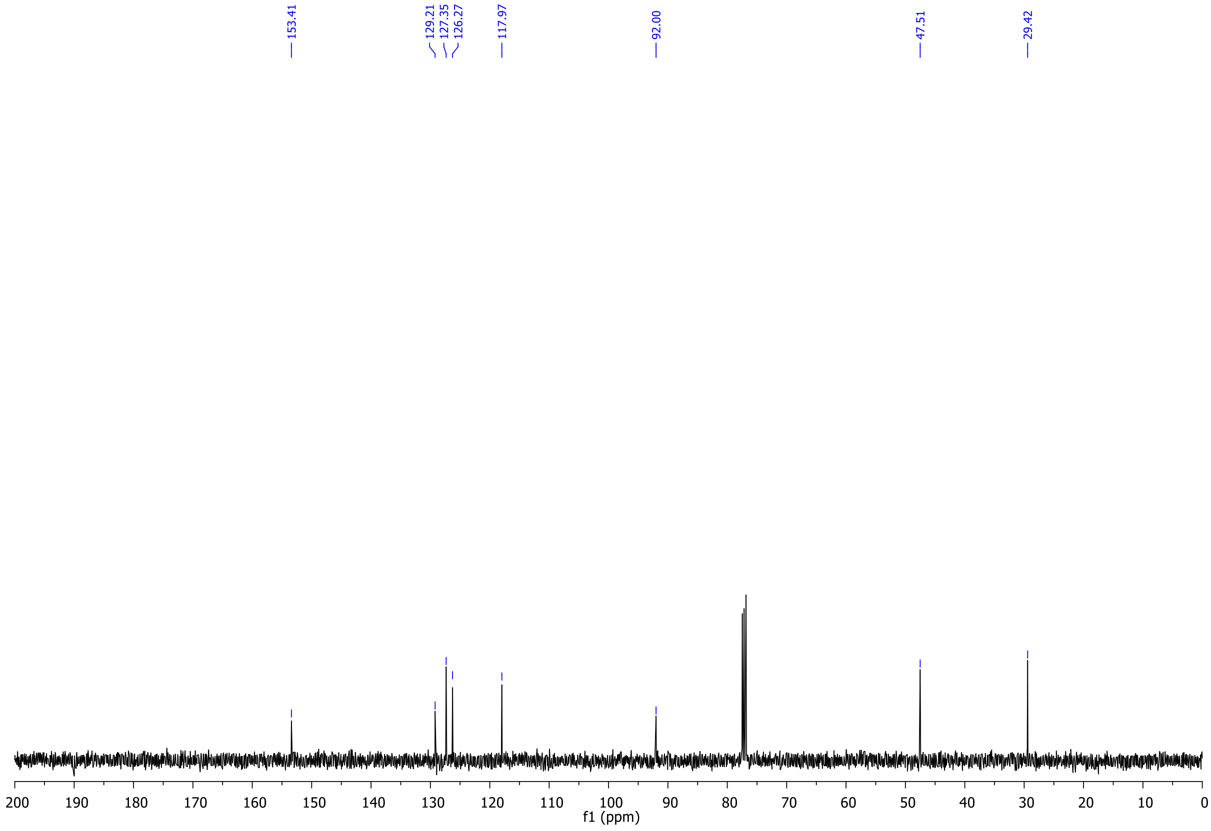

^{1}H NMR (400 MHz) and ^{13}C{^{1}H} NMR (100 MHz) spectra of **1h** ($CDCl_3$)

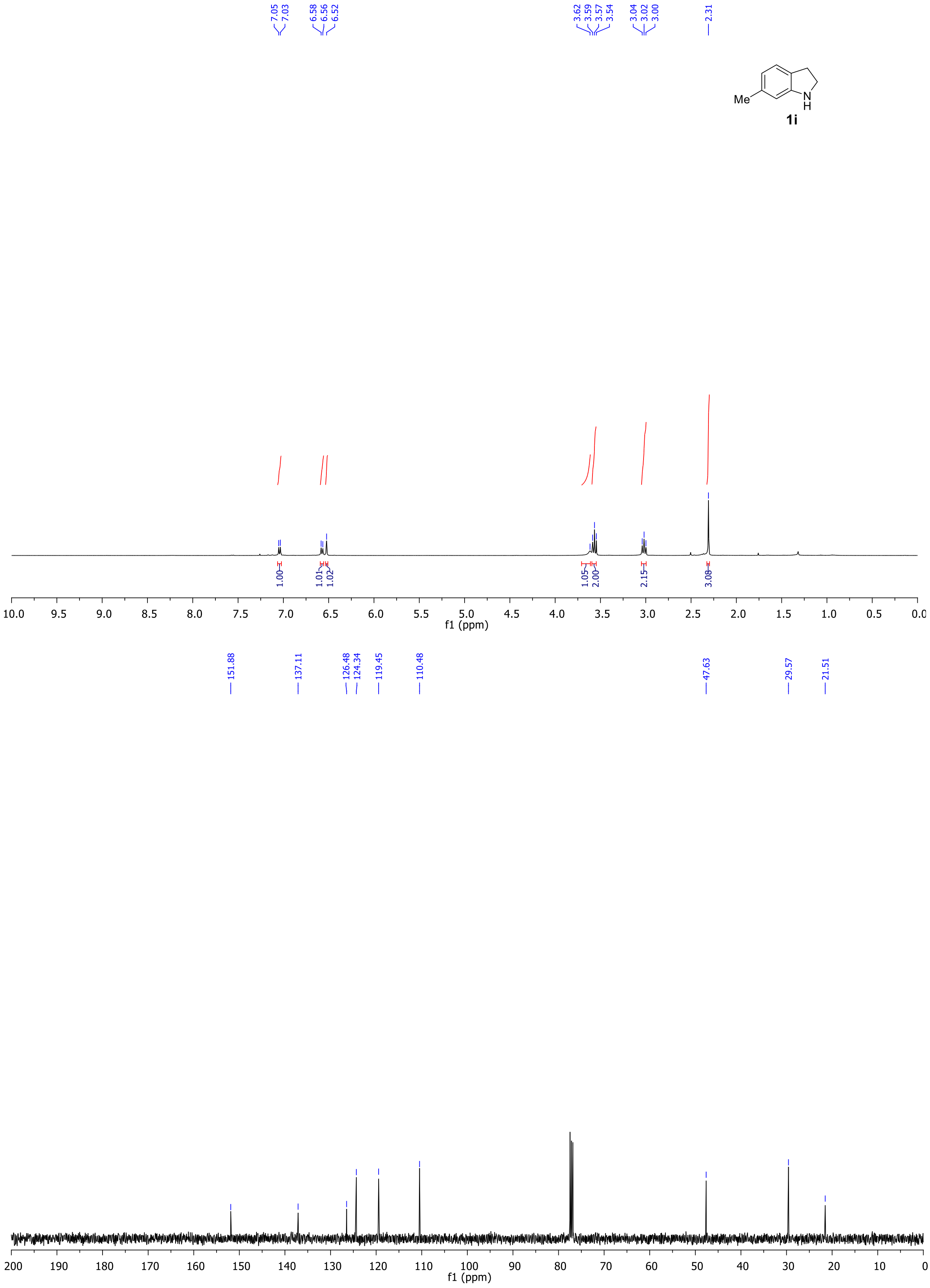

^{1}H NMR (400 MHz) and ^{13}C{^{1}H} NMR (100 MHz) spectra of **1i** ($CDCl_3$)

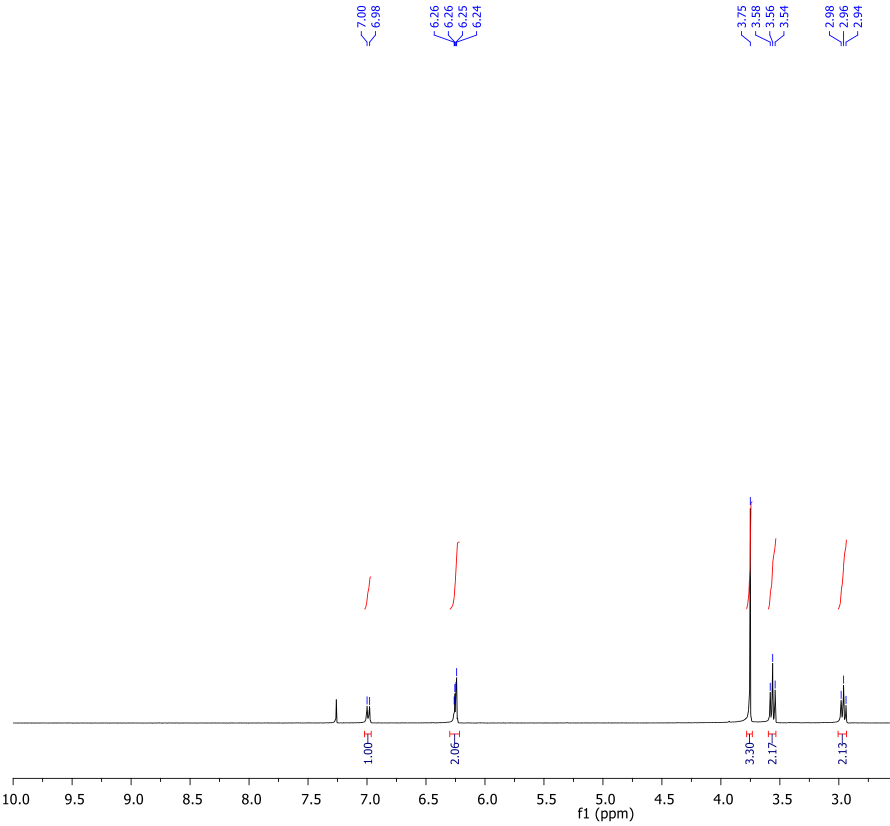

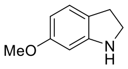

1j

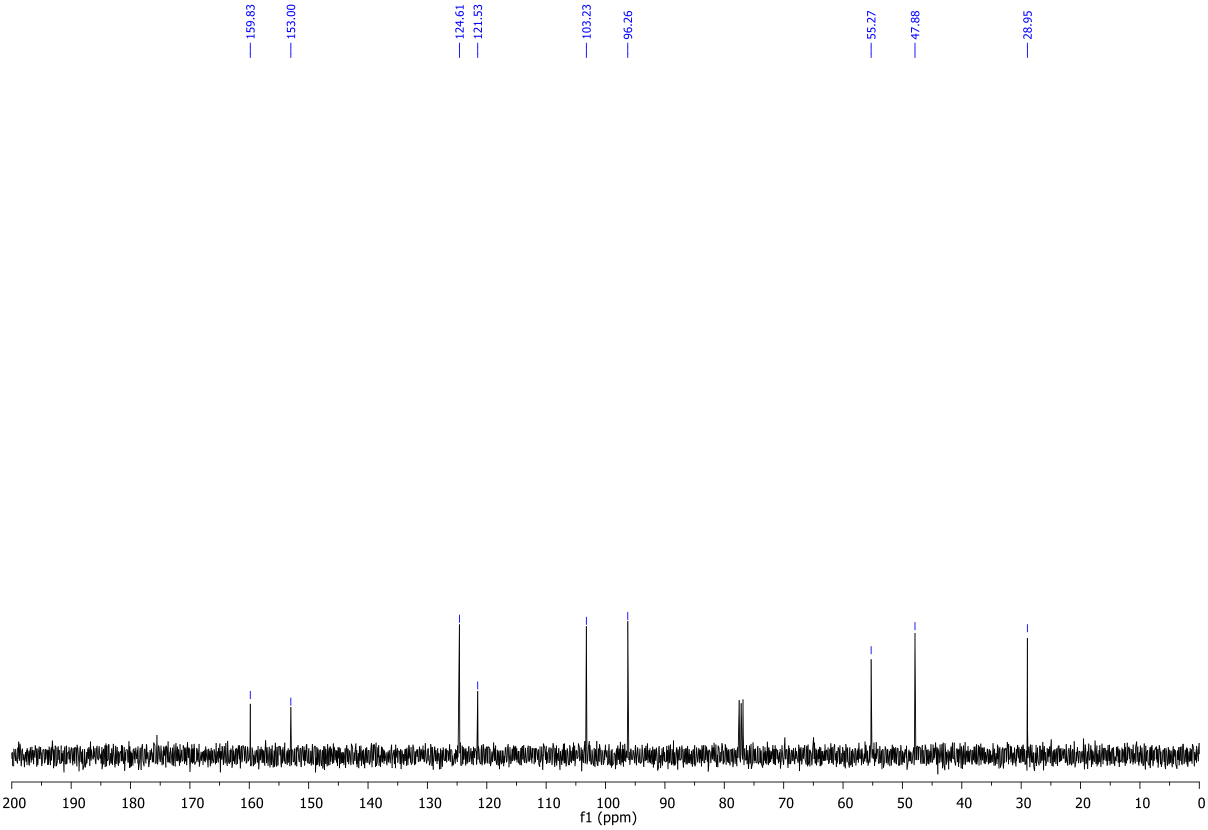

^{1}H NMR (400 MHz) and ^{13}C{^{1}H} NMR (100 MHz) spectra of **1j** ($CDCl_3$)

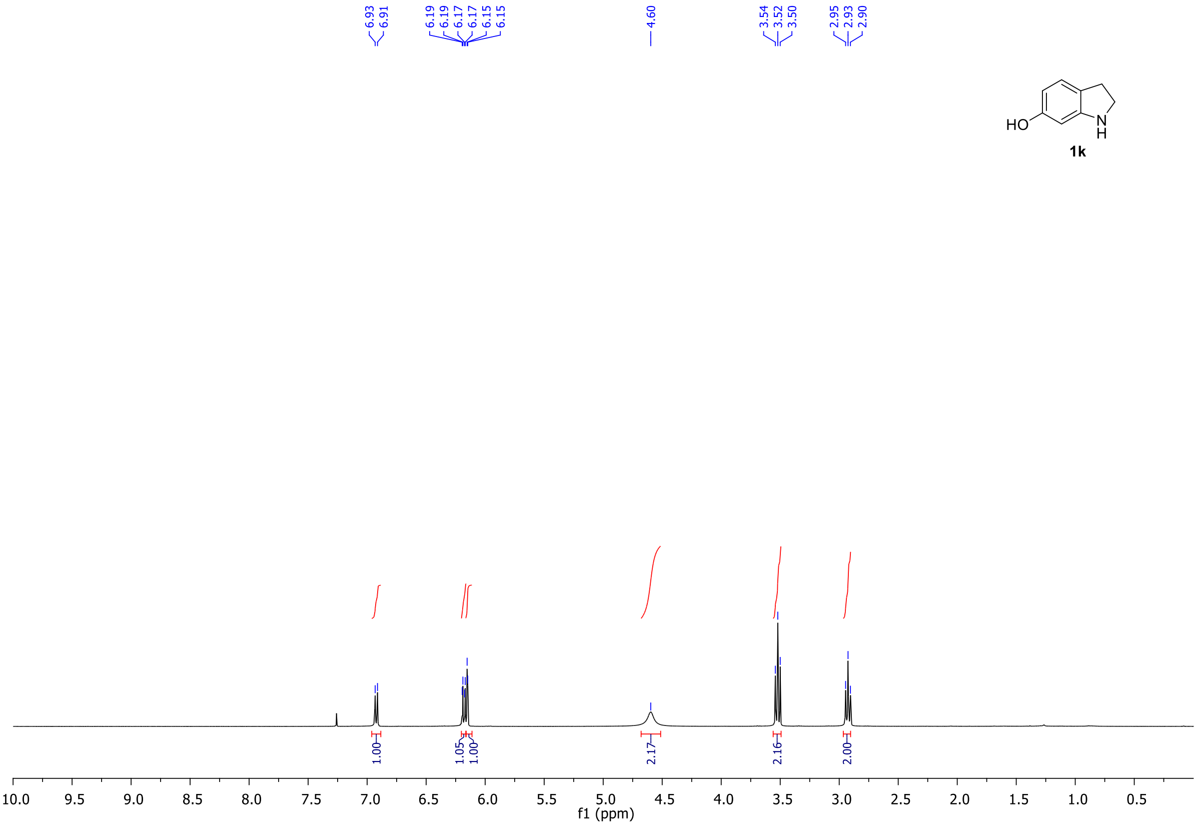

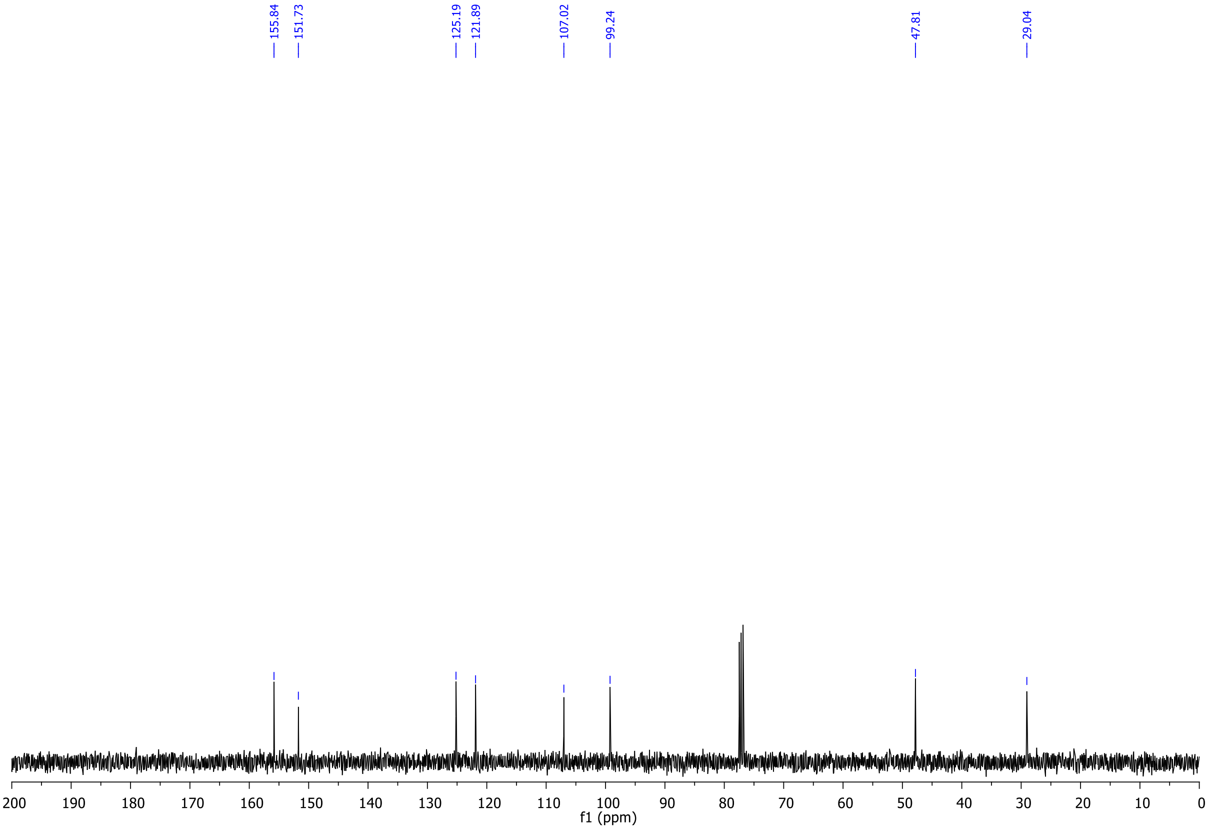

^{1}H NMR (400 MHz) and ^{13}C{^{1}H} NMR (100 MHz) spectra of **1k** ($CDCl_3$)

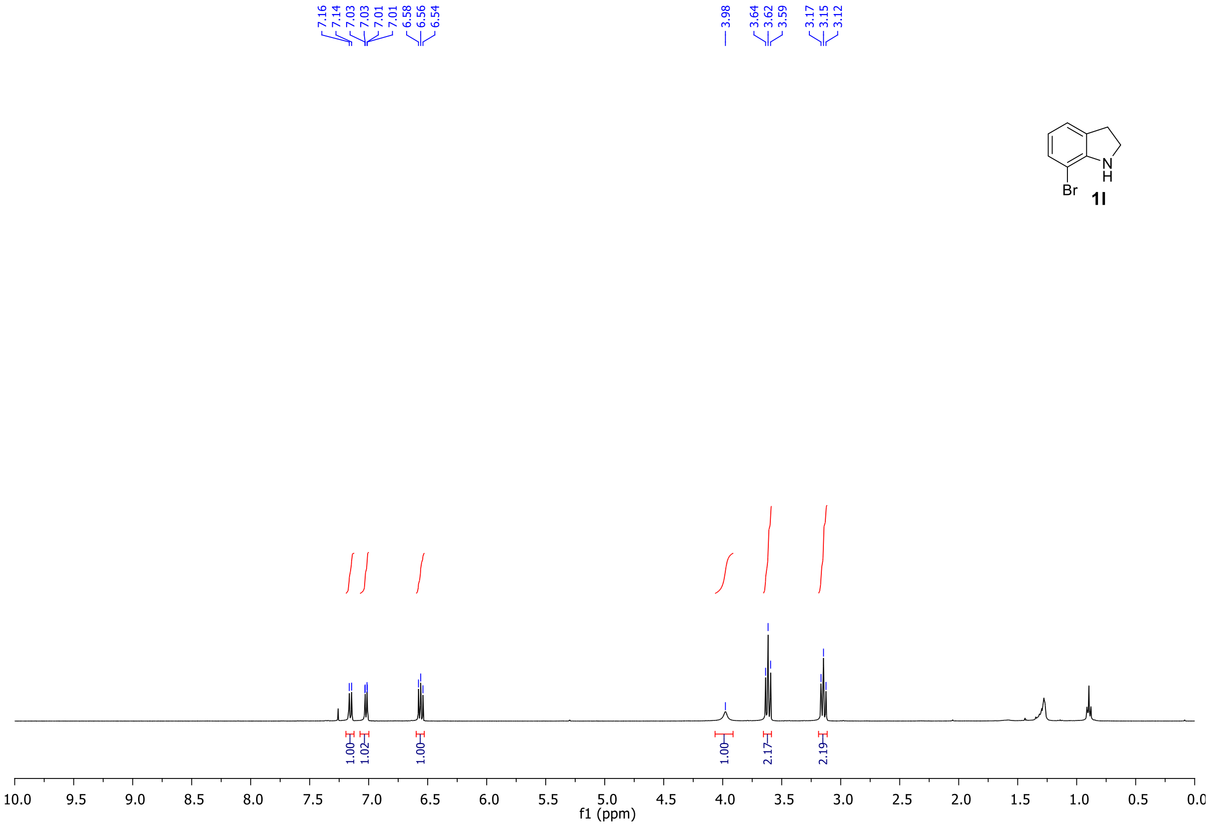

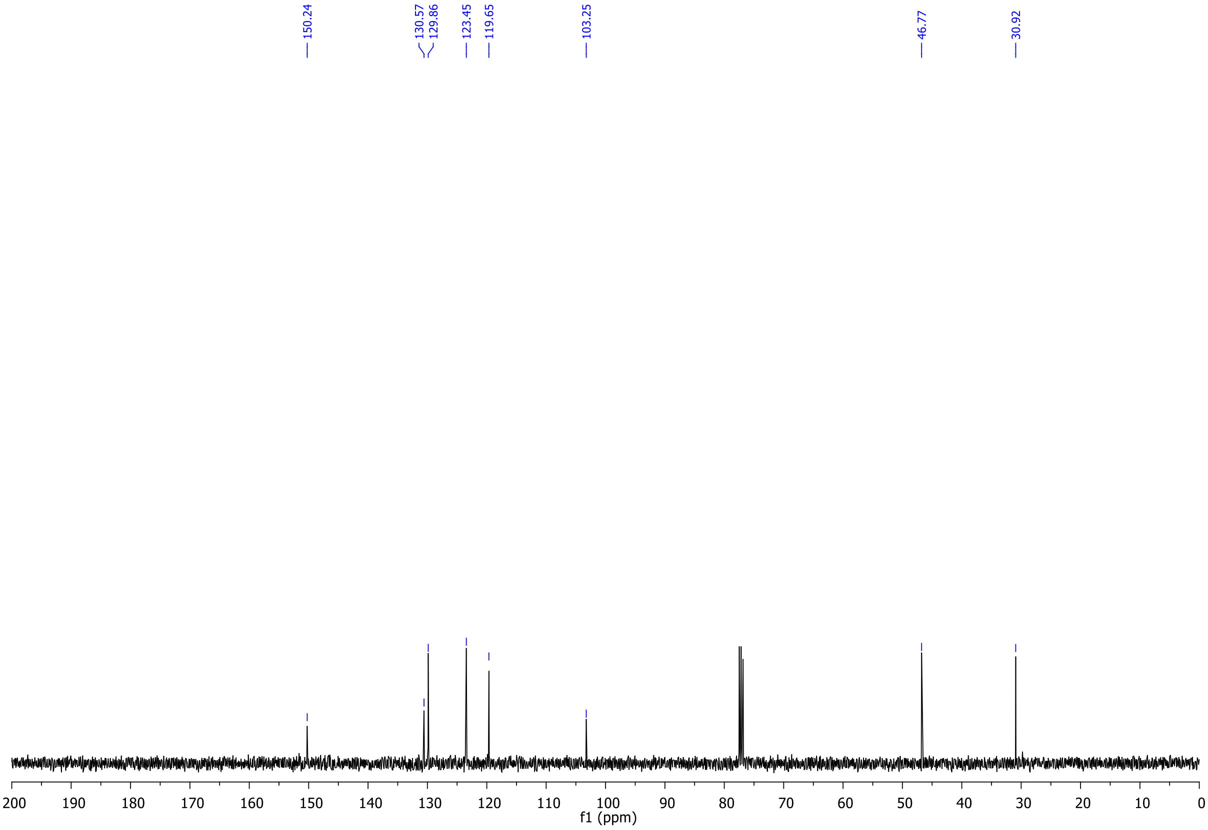

^{1}H NMR (400 MHz) and ^{13}C{^{1}H} NMR (100 MHz) spectra of **1l** ($CDCl_3$)

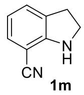

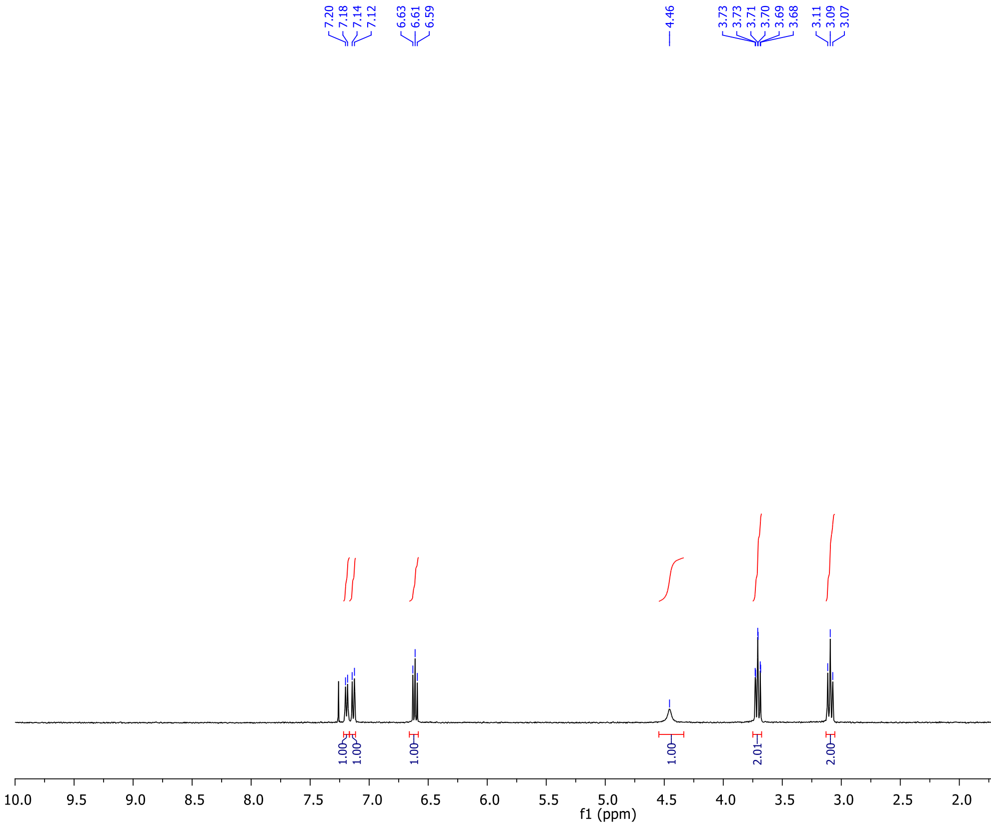

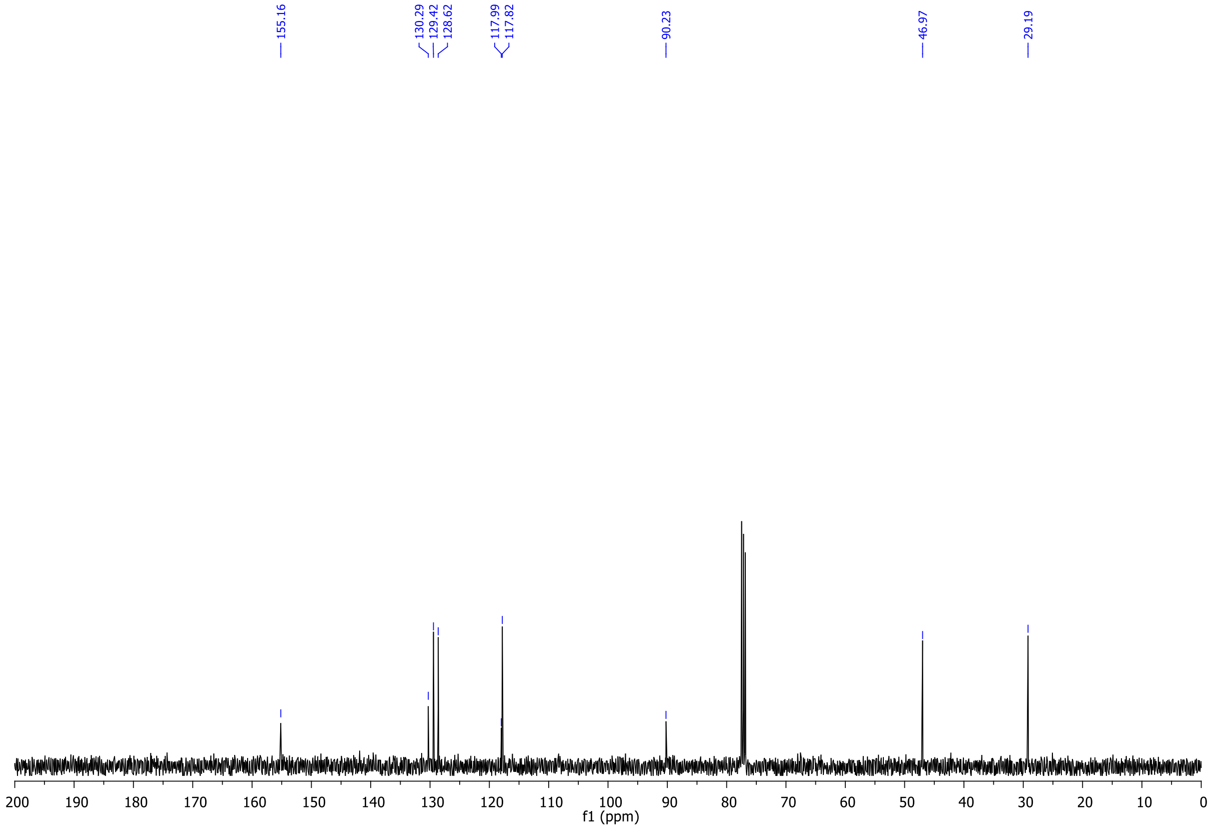

^{1}H NMR (400 MHz) and $^{13}C\{^{1}H\}$ NMR (100 MHz) spectra of **1m** ($CDCl_3$)

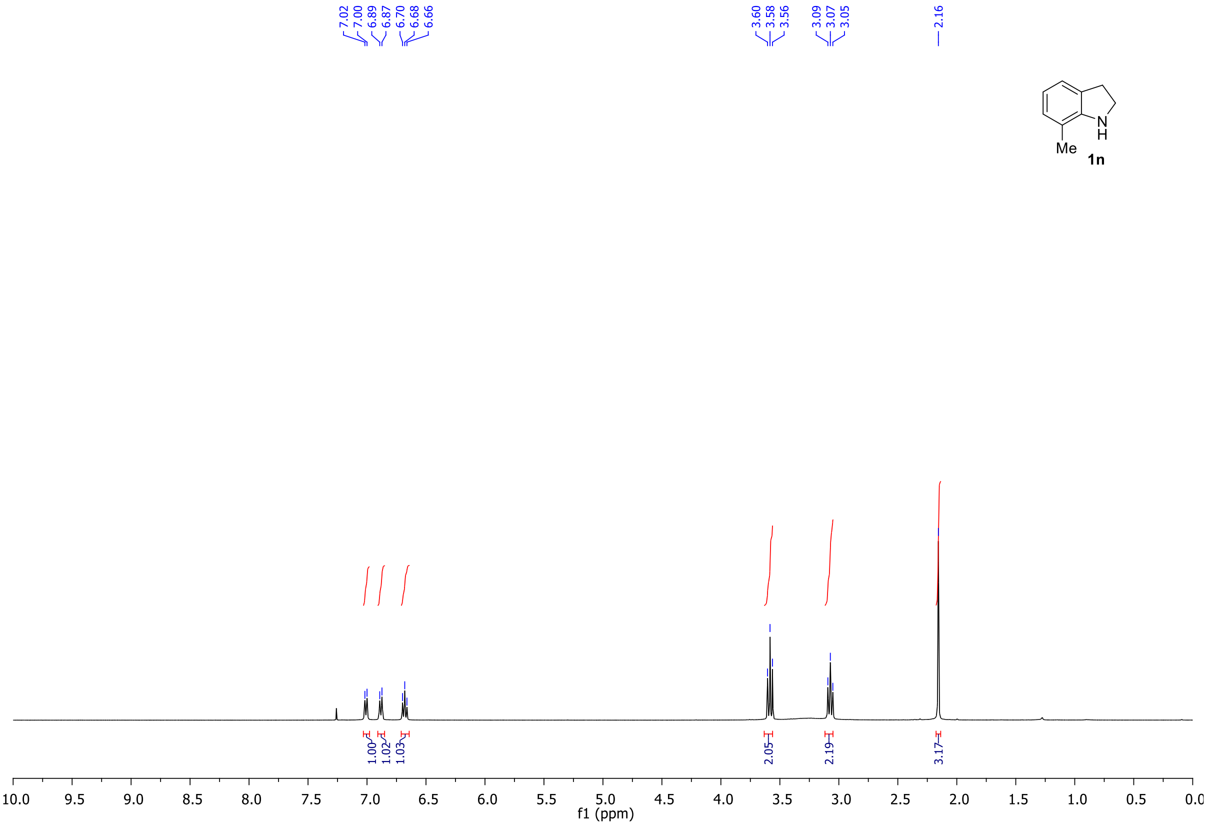

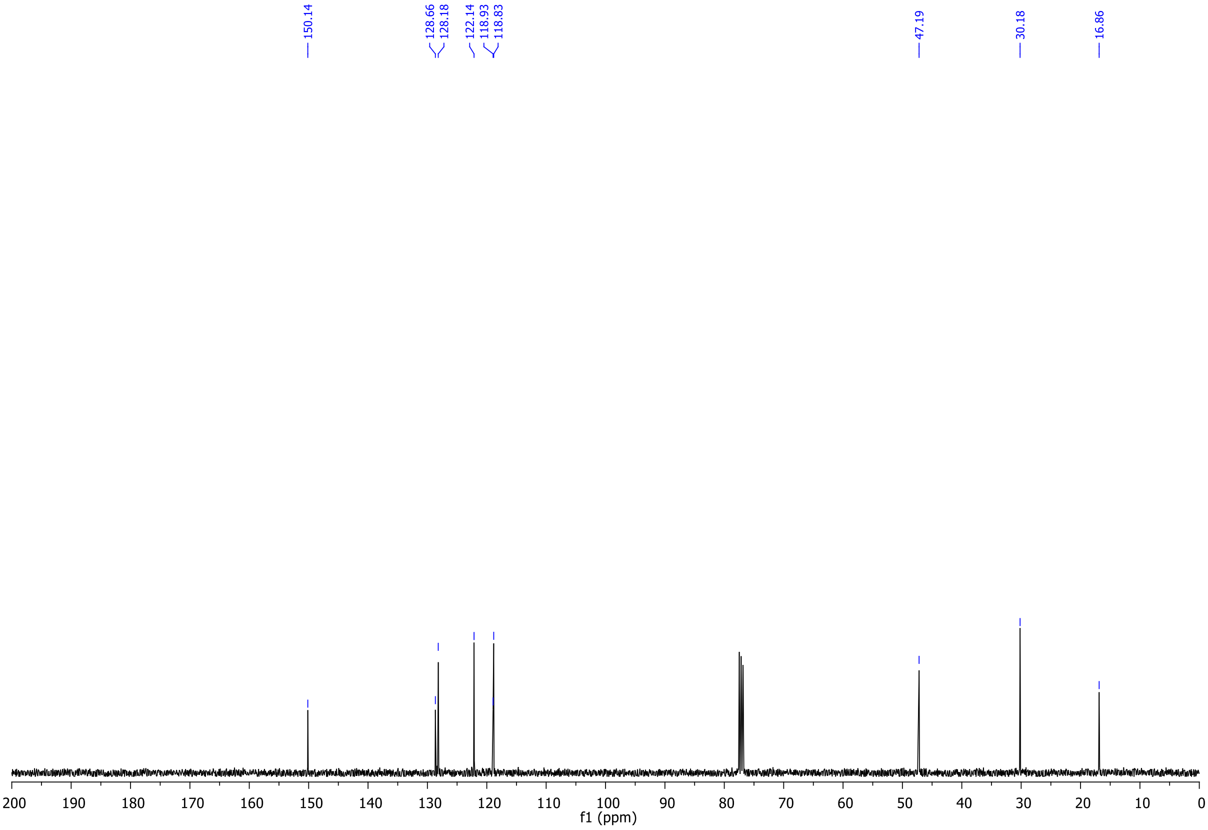

^{1}H NMR (400 MHz) and ^{13}C{^{1}H} NMR (100 MHz) spectra of **1n** ($CDCl_3$)

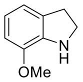

1o

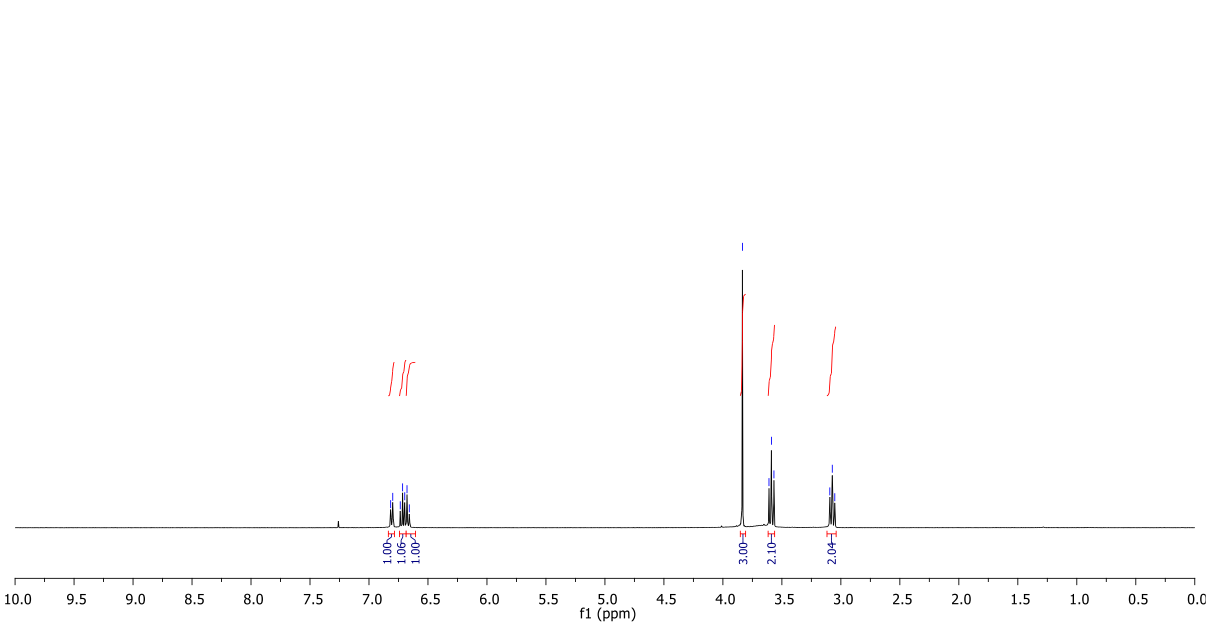

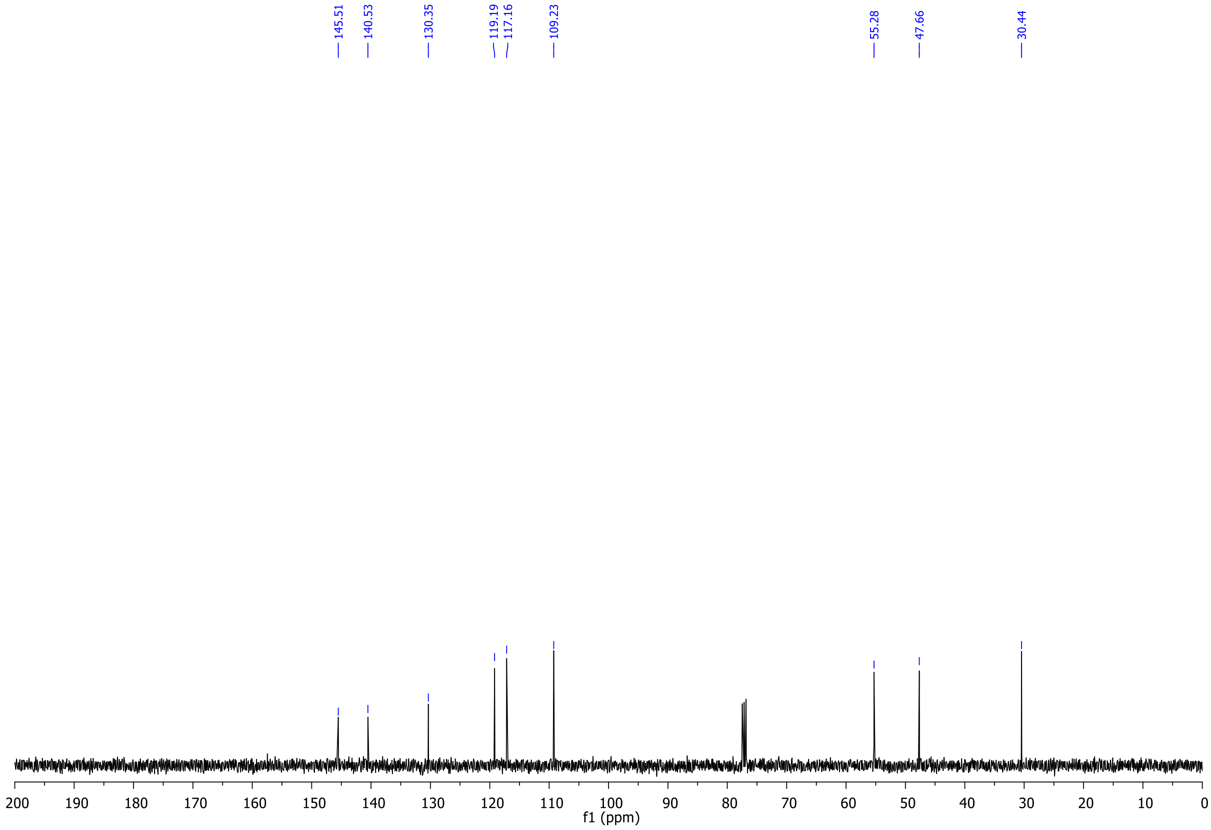

^{1}H NMR (400 MHz) and ^{13}C{^{1}H} NMR (100 MHz) spectra of **1o** ($CDCl_3$)

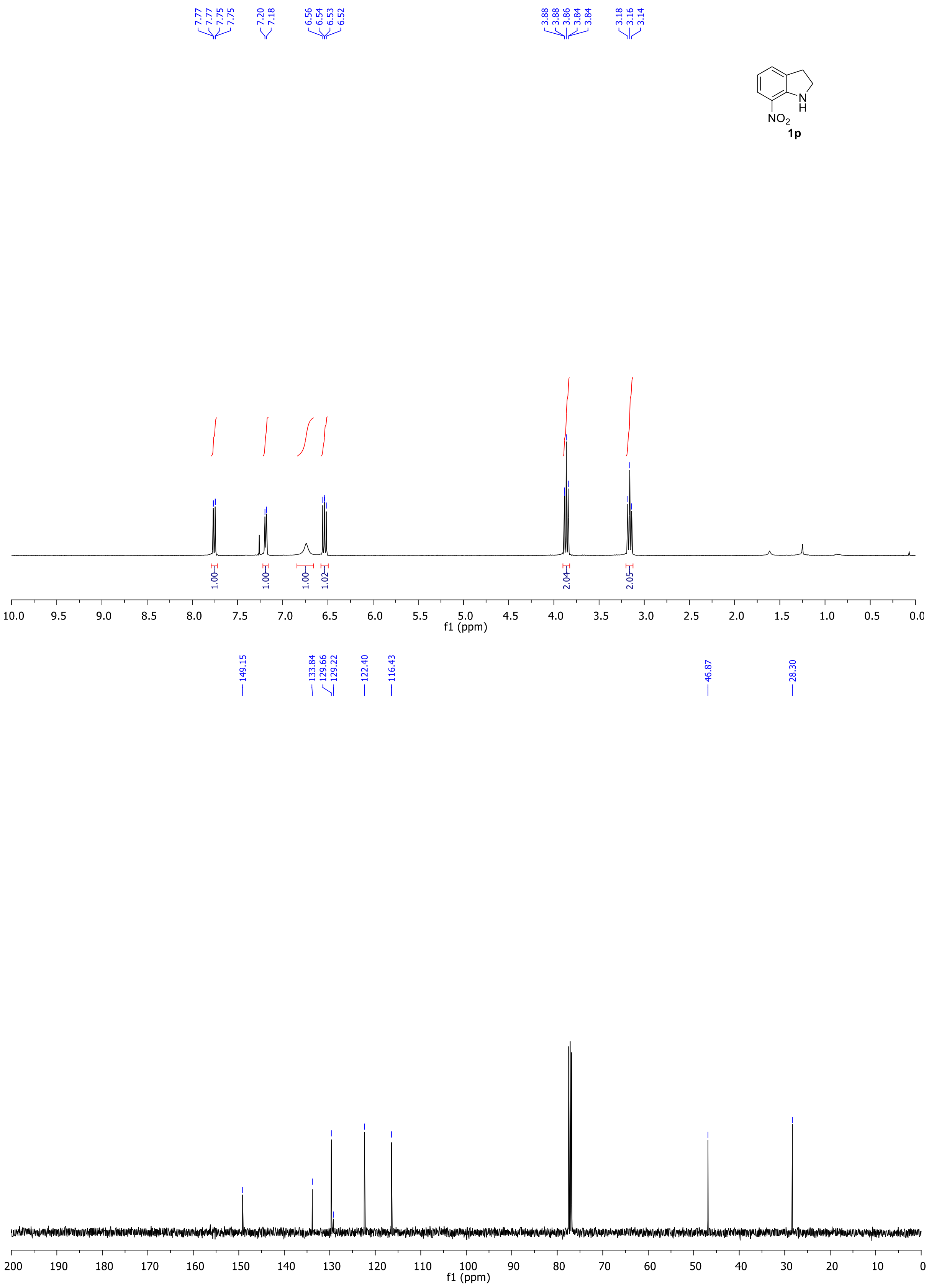

^{1}H NMR (400 MHz) and $^{13}C\{^{1}H\}$ NMR (100 MHz) spectra of **1p** ($CDCl_3$)

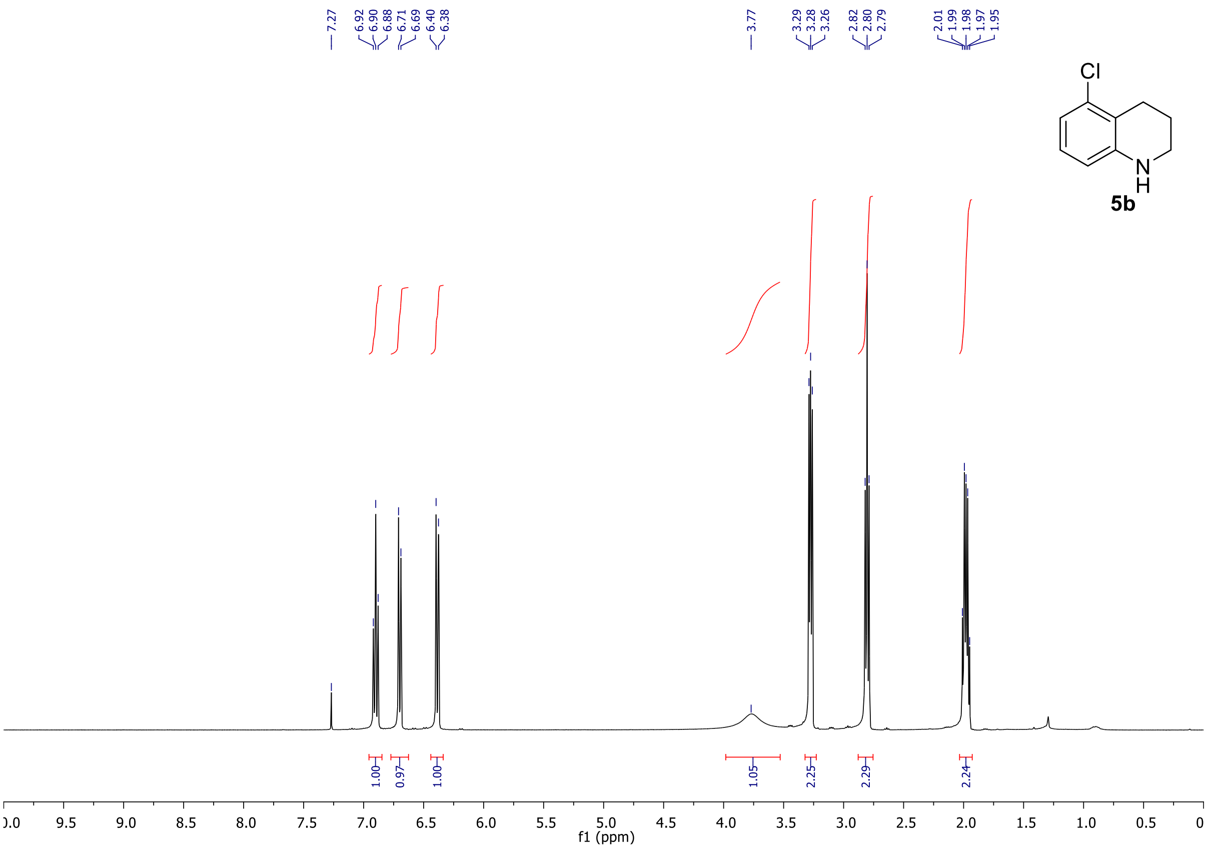

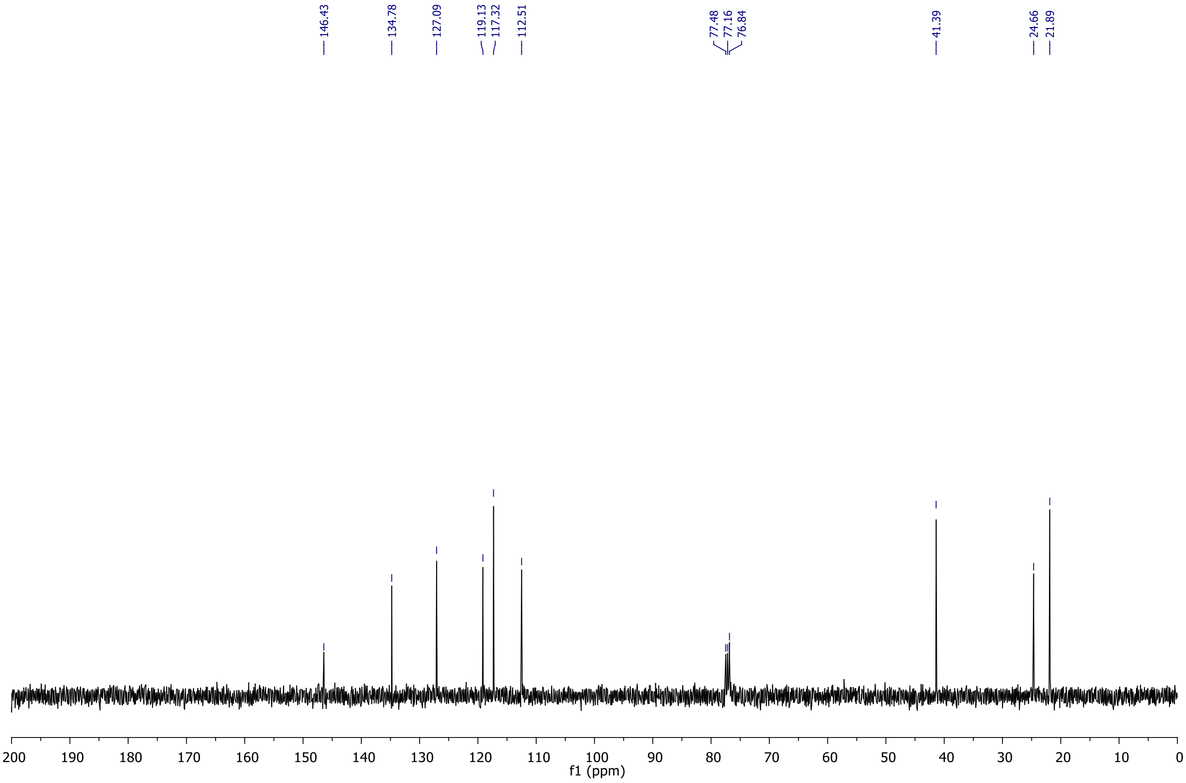

^{1}H NMR (400 MHz) and ^{13}C{^{1}H} NMR (100 MHz) spectra of **5b** ($CDCl_3$)

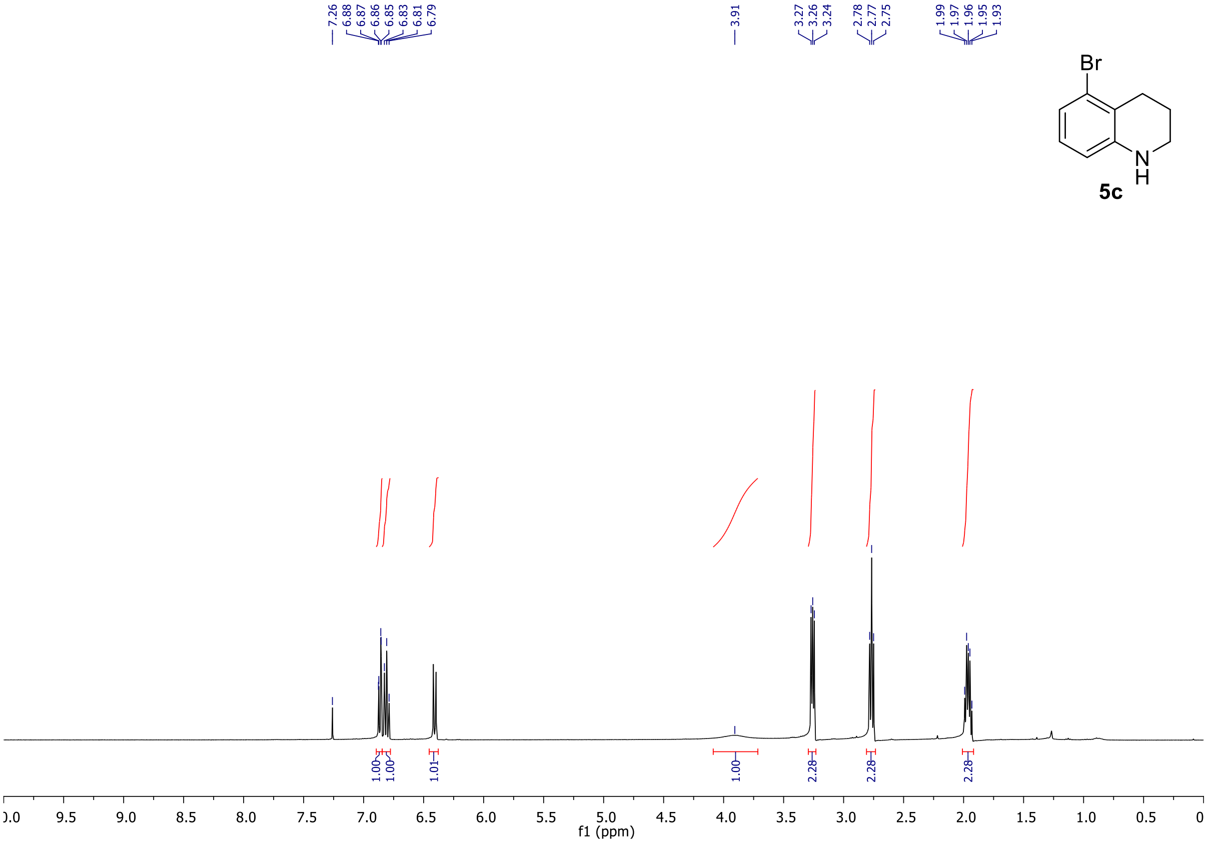

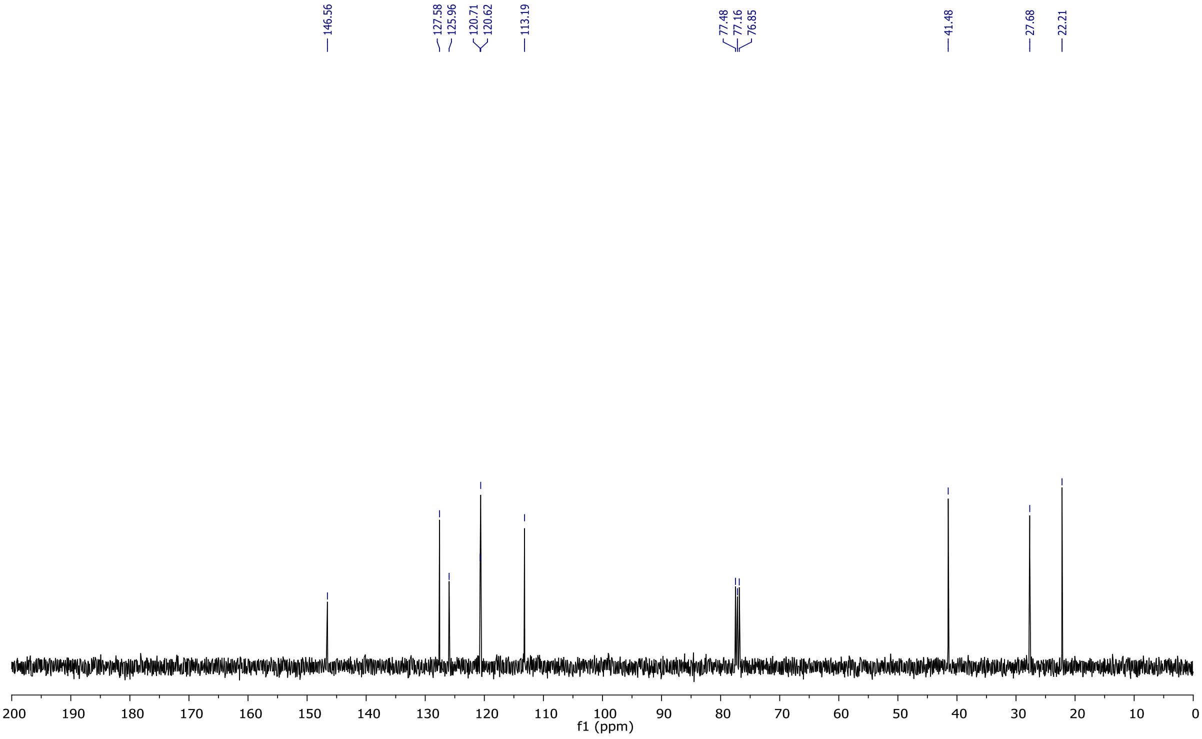

^{1}H NMR (400 MHz) and ^{13}C{^{1}H} NMR (100 MHz) spectra of **5c** ($CDCl_3$)

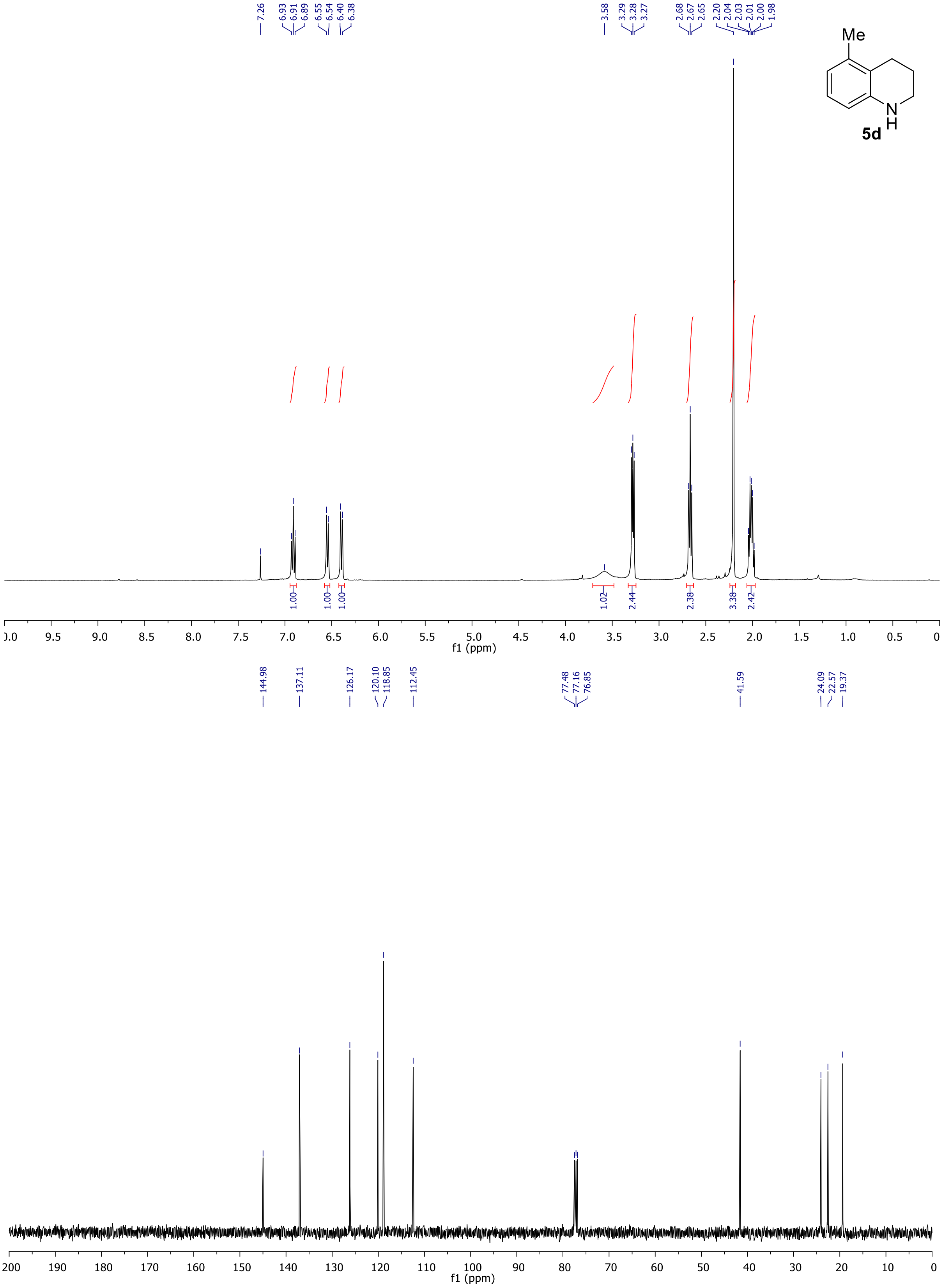

^{1}H NMR (400 MHz) and $^{13}C\{^{1}H\}$ NMR (100 MHz) spectra of **5d** ($CDCl_3$)

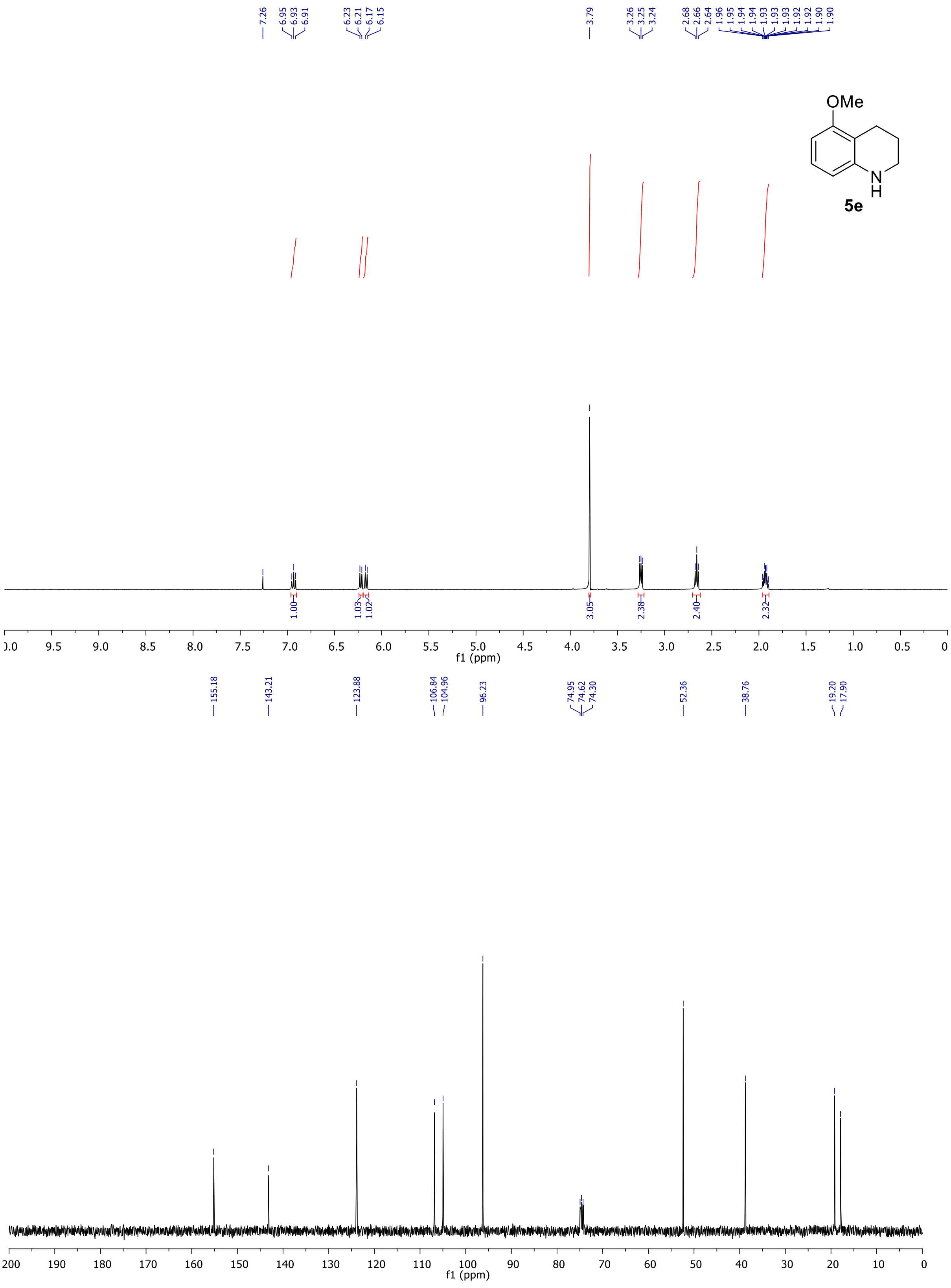

^{1}H NMR (400 MHz) and $^{13}C\{^{1}H\}$ NMR (100 MHz) spectra of **5e** ($CDCl_3$)

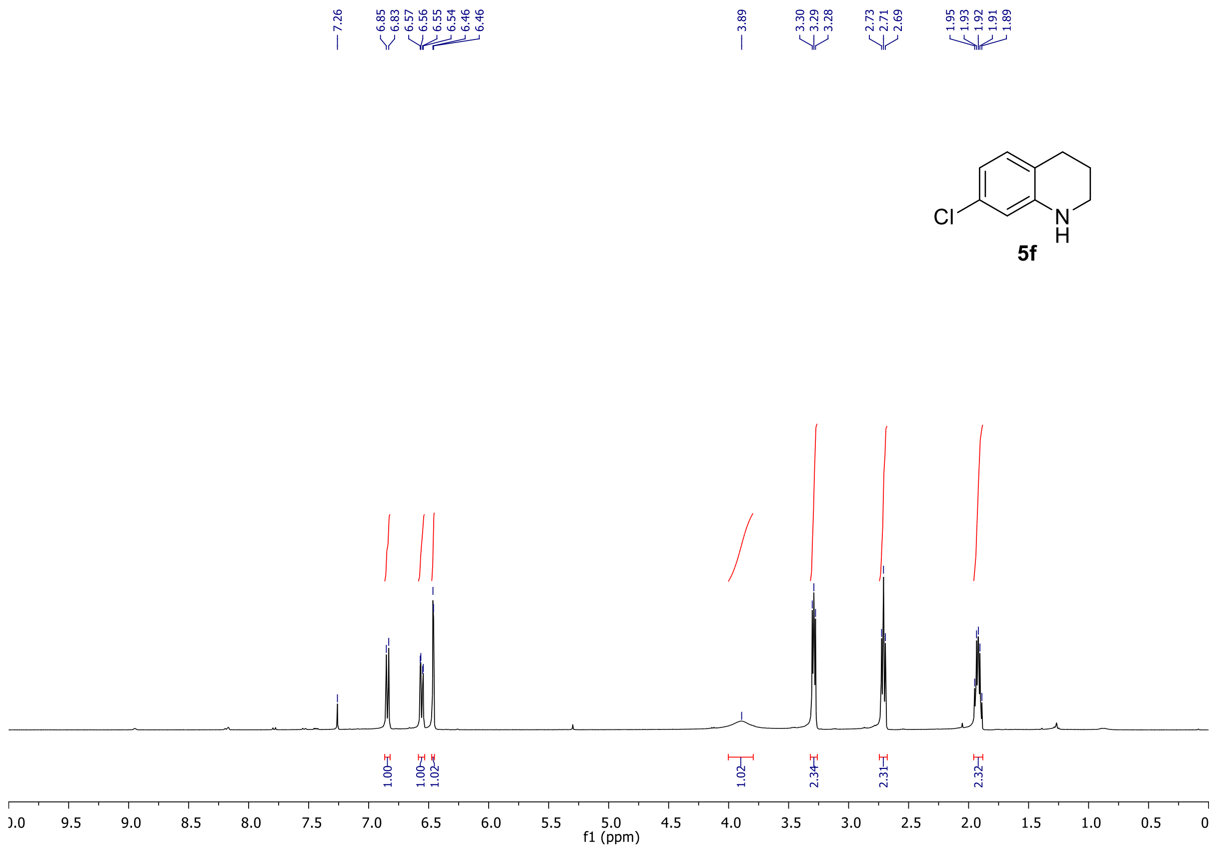

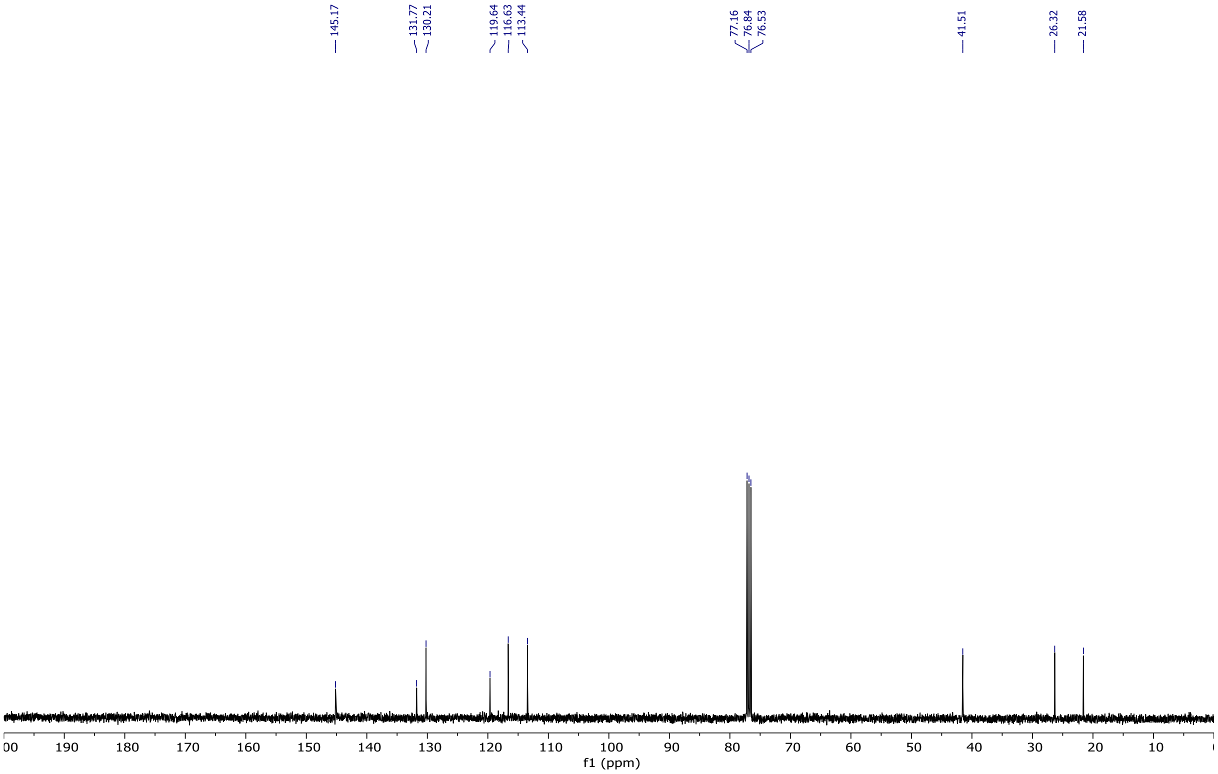

^{1}H NMR (400 MHz) and $^{13}C\{^{1}H\}$ NMR (100 MHz) spectra of **5f** ($CDCl_3$)

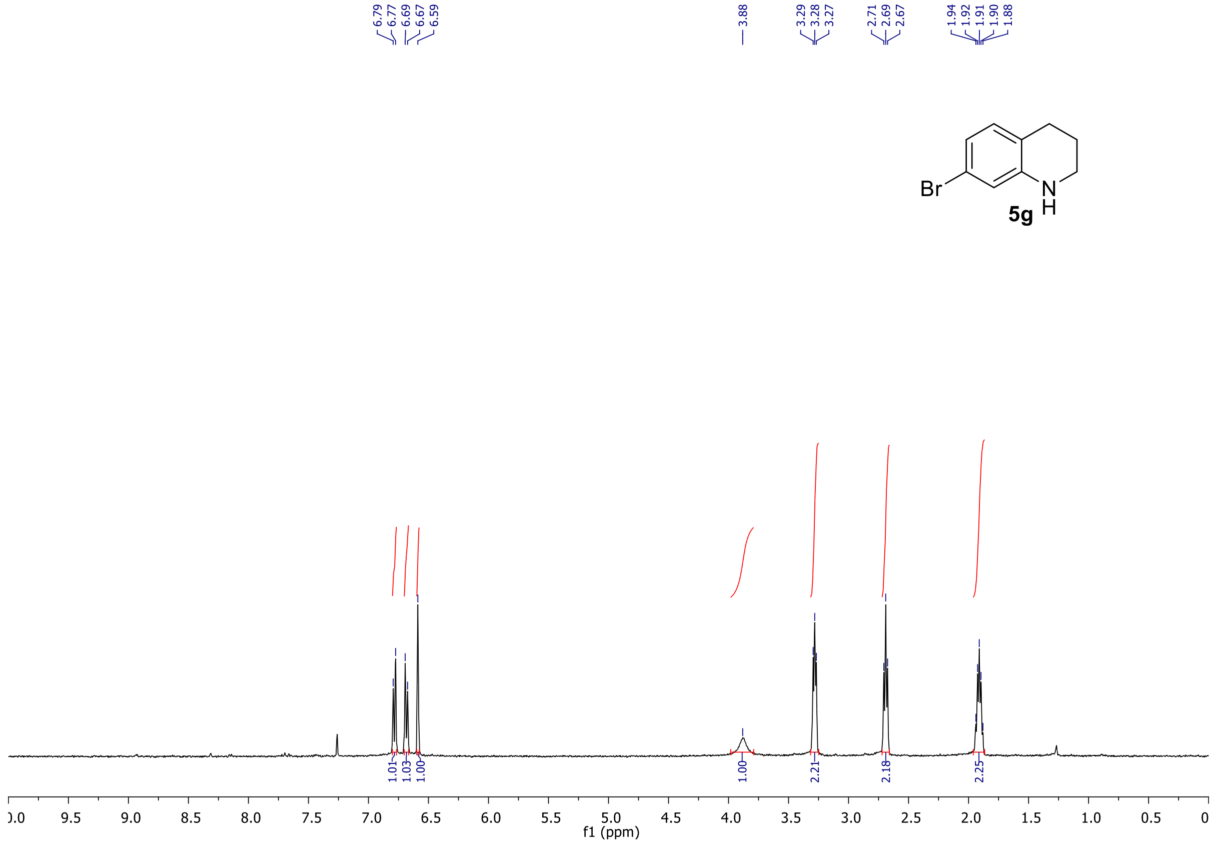

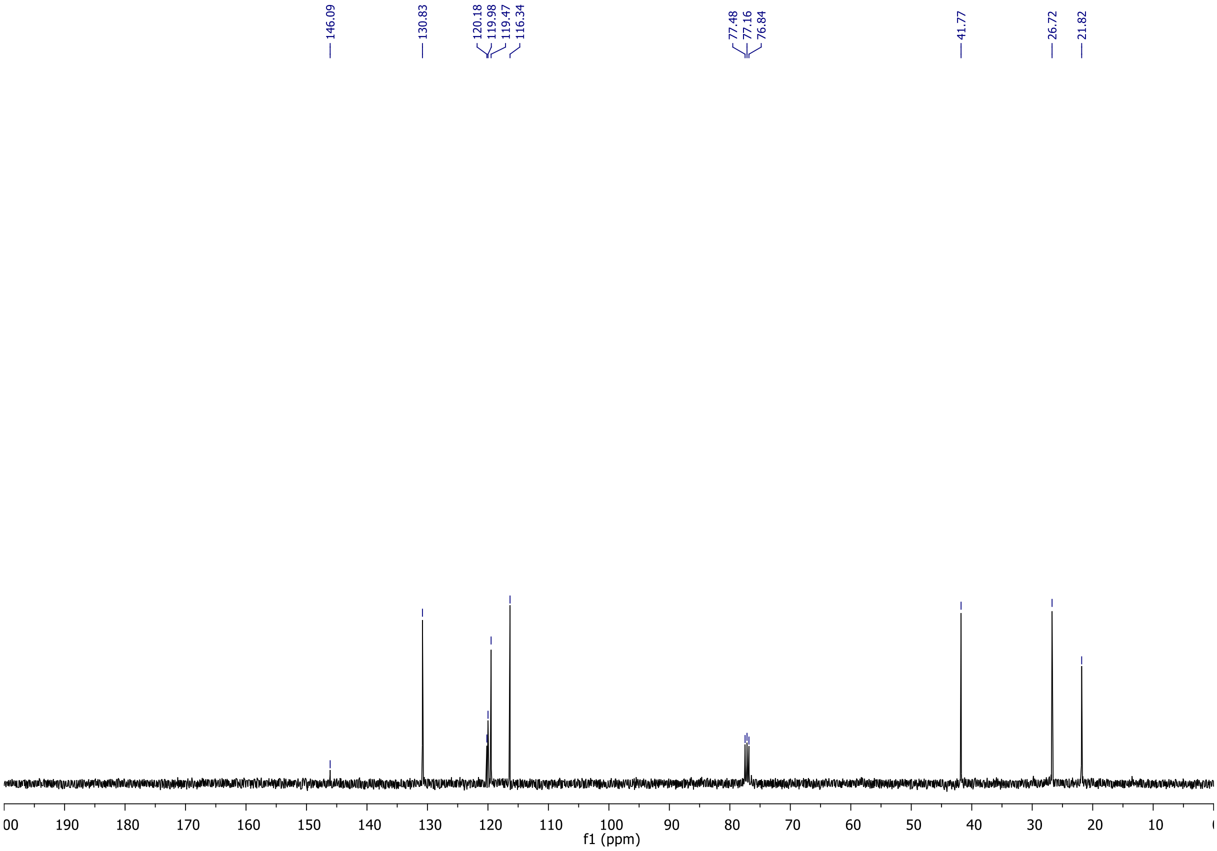

^{1}H NMR (400 MHz) and ^{13}C{^{1}H} NMR (100 MHz) spectra of **5g** ($CDCl_3$)

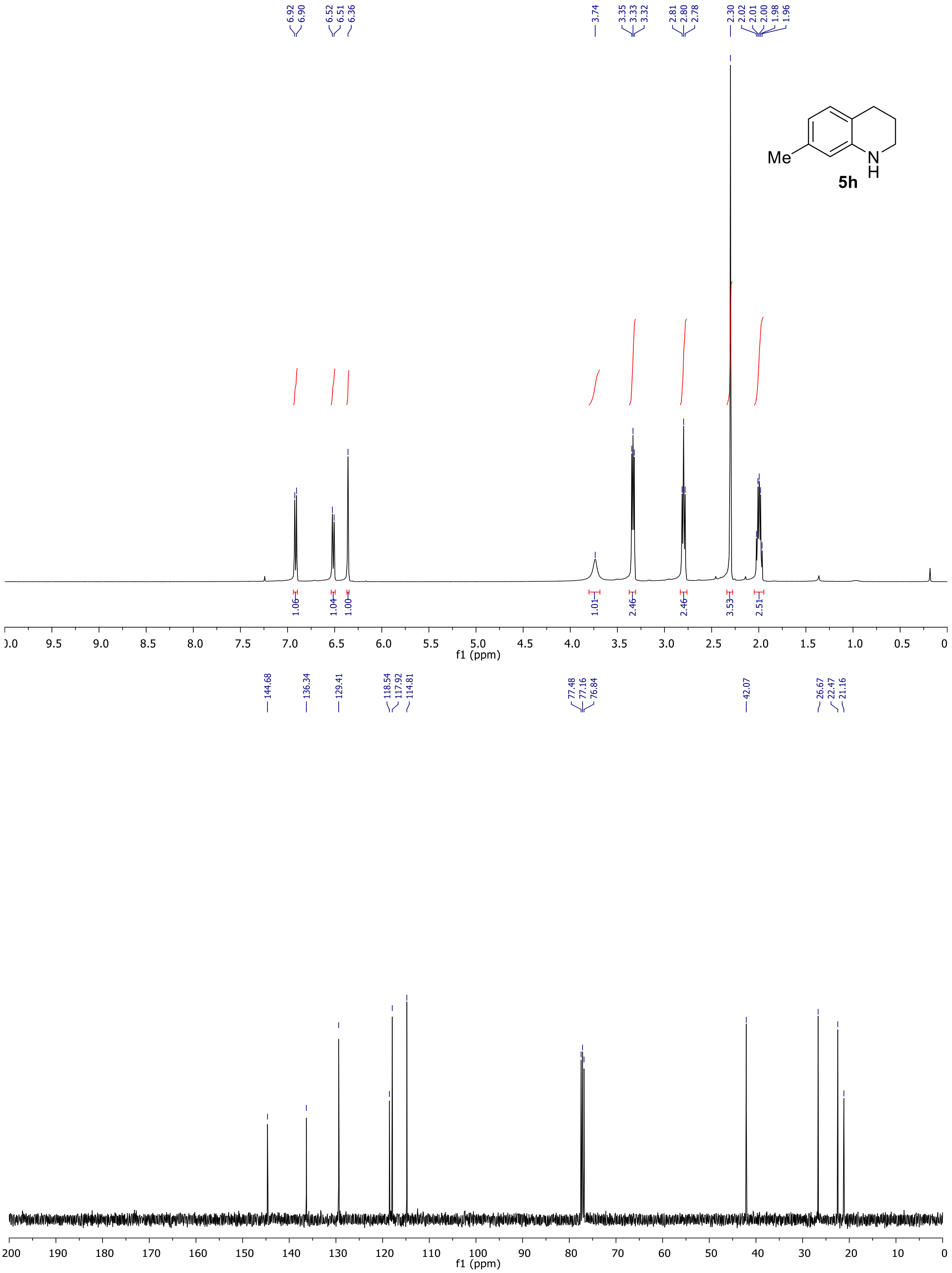

^{1}H NMR (400 MHz) and $^{13}C\{^{1}H\}$ NMR (100 MHz) spectra of **5h** ($CDCl_3$)

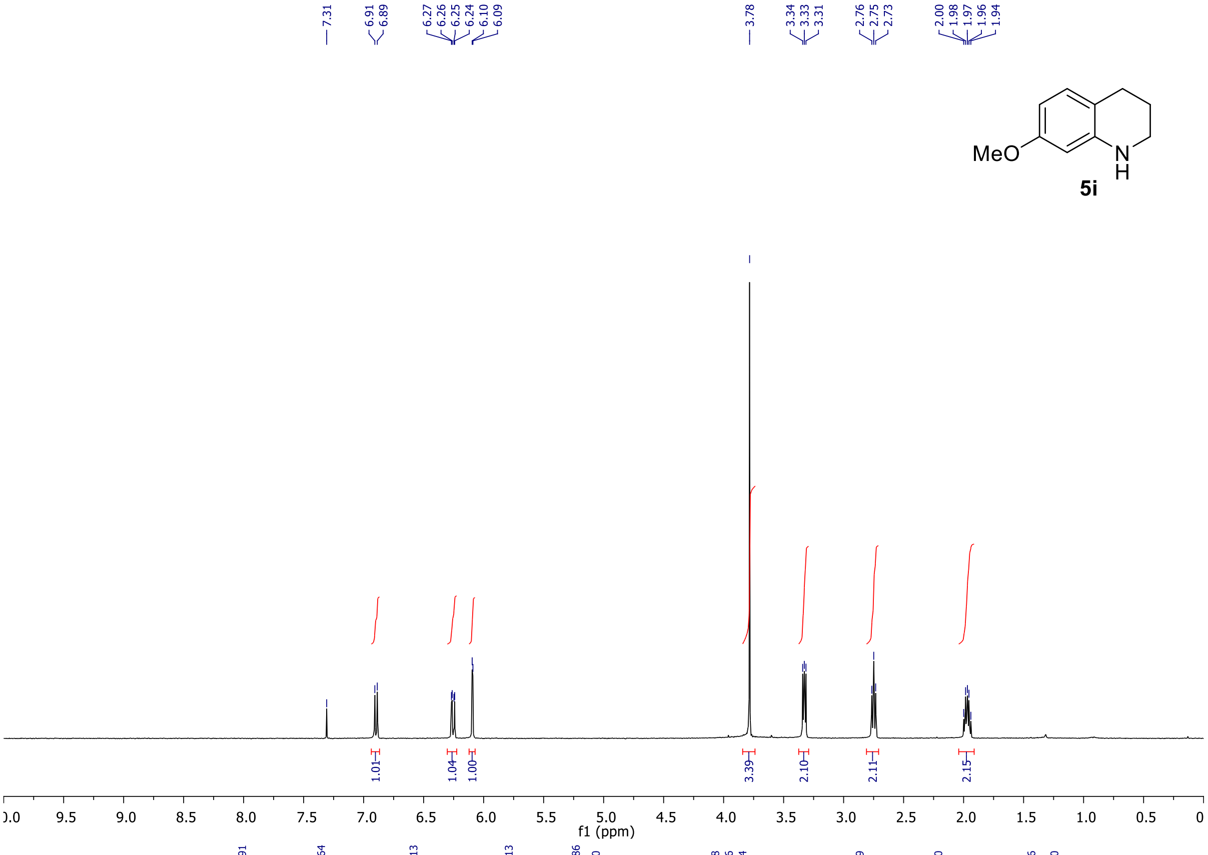

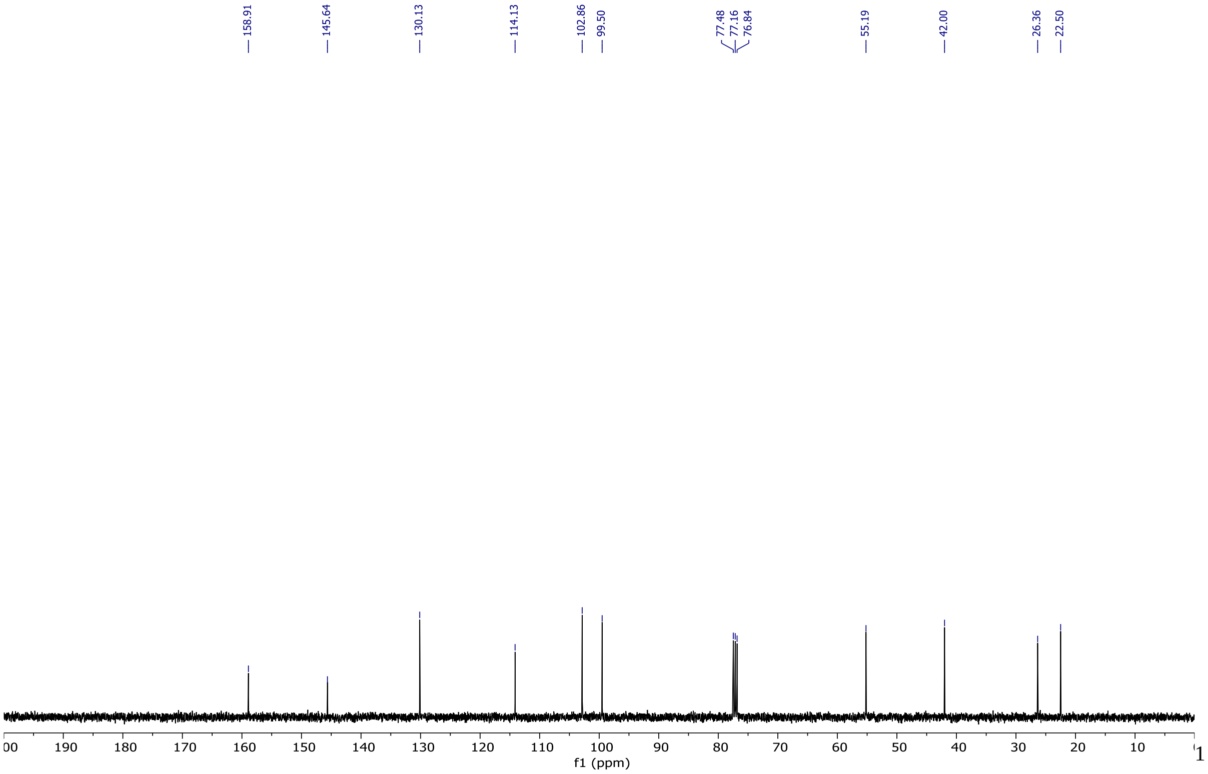

H NMR (400 MHz) and ^{13}C{^{1}H} NMR (100 MHz) spectra of **5i** ($CDCl_3$)

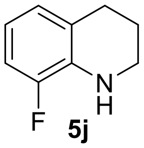

5j

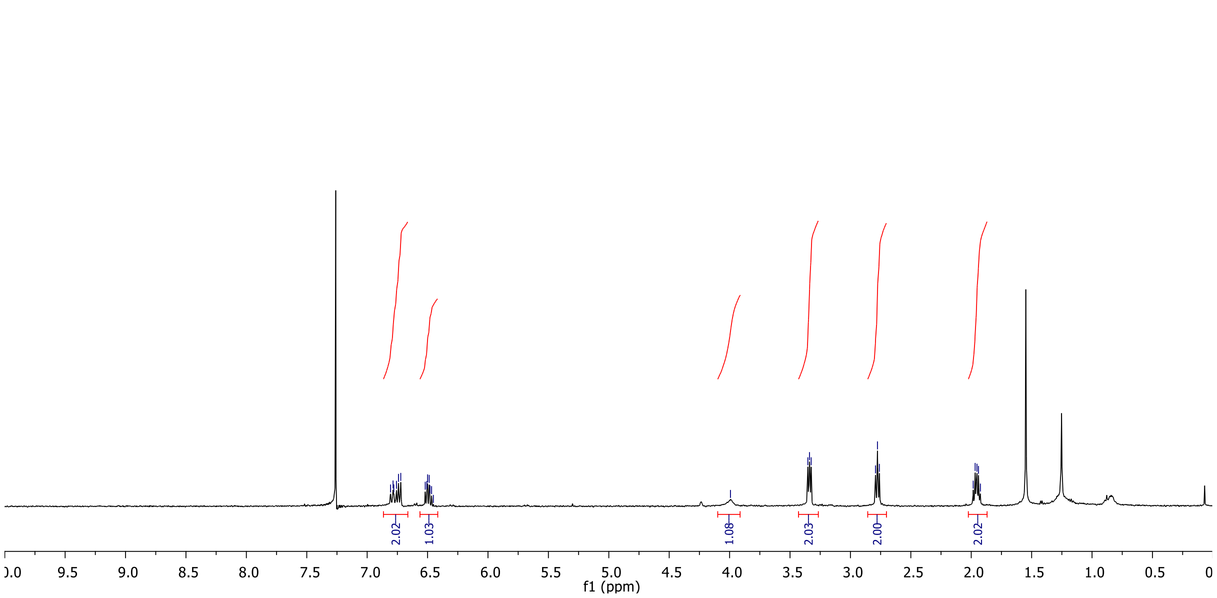

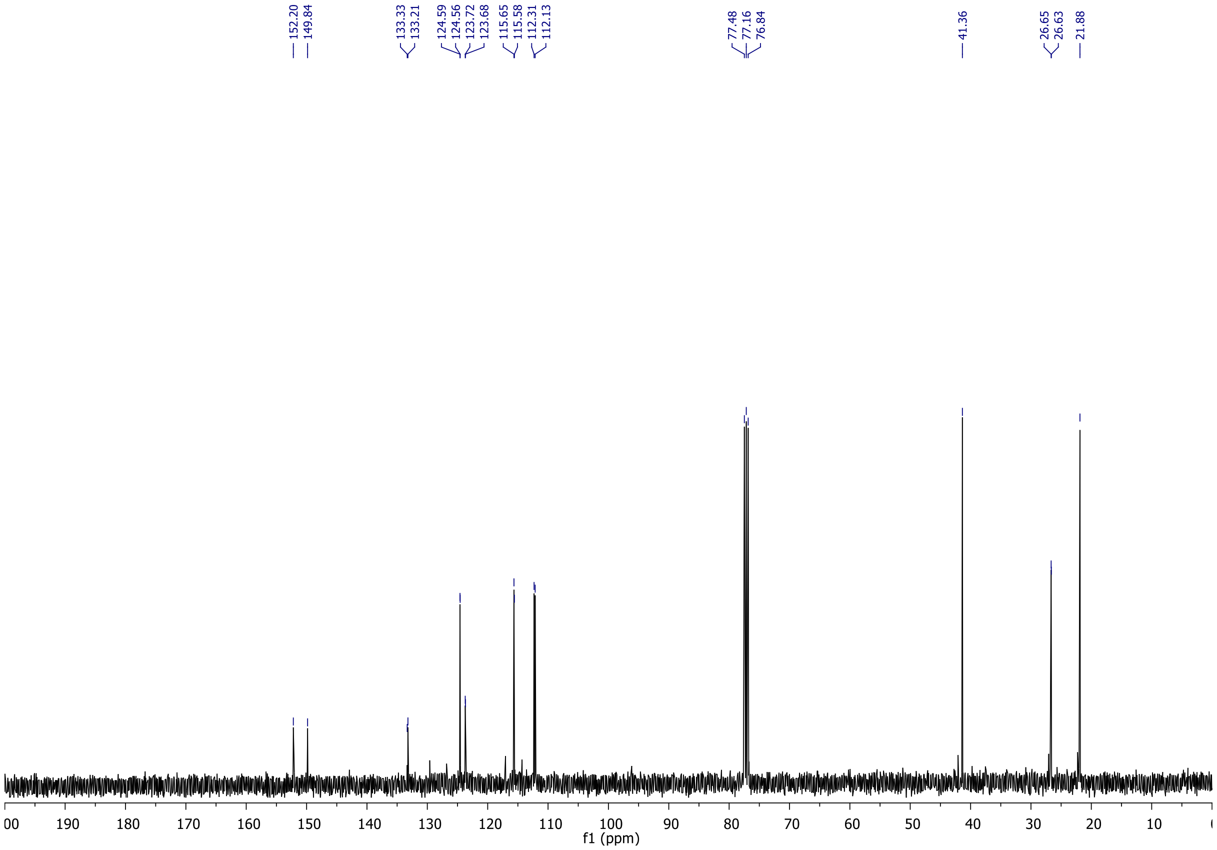

^{1}H NMR (400 MHz) and ^{13}C{^{1}H} NMR (100 MHz) spectra of **5j** ($CDCl_3$)

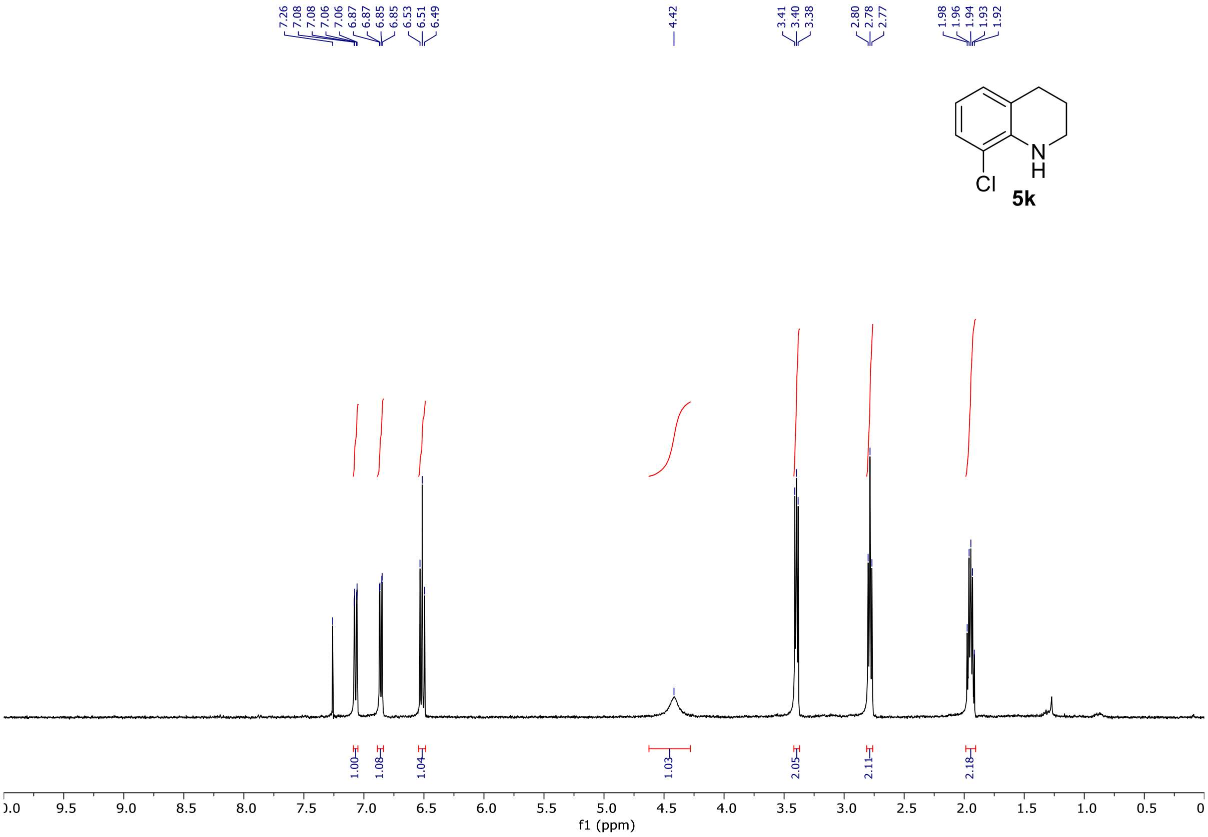

5k

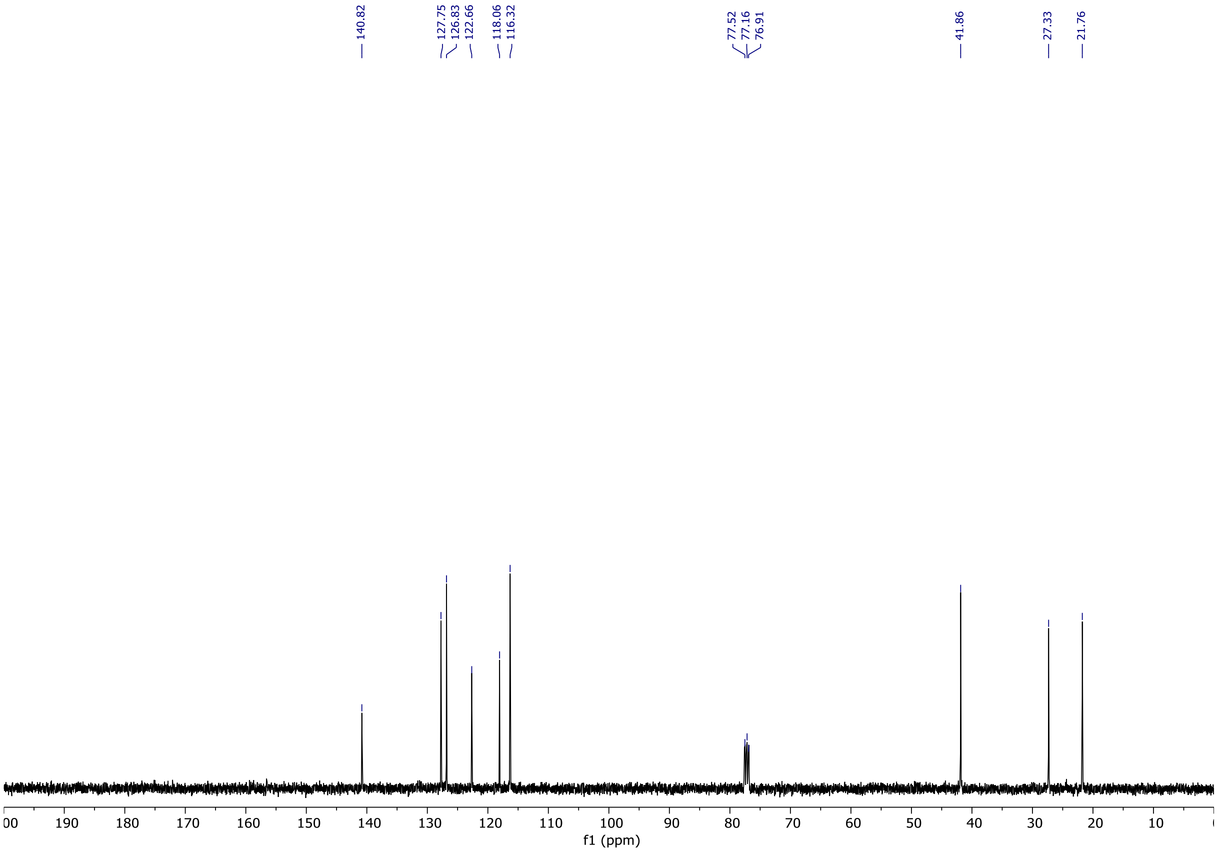

^{1}H NMR (400 MHz) and ^{13}C{^{1}H} NMR (100 MHz) spectra of **5k** ($CDCl_3$)

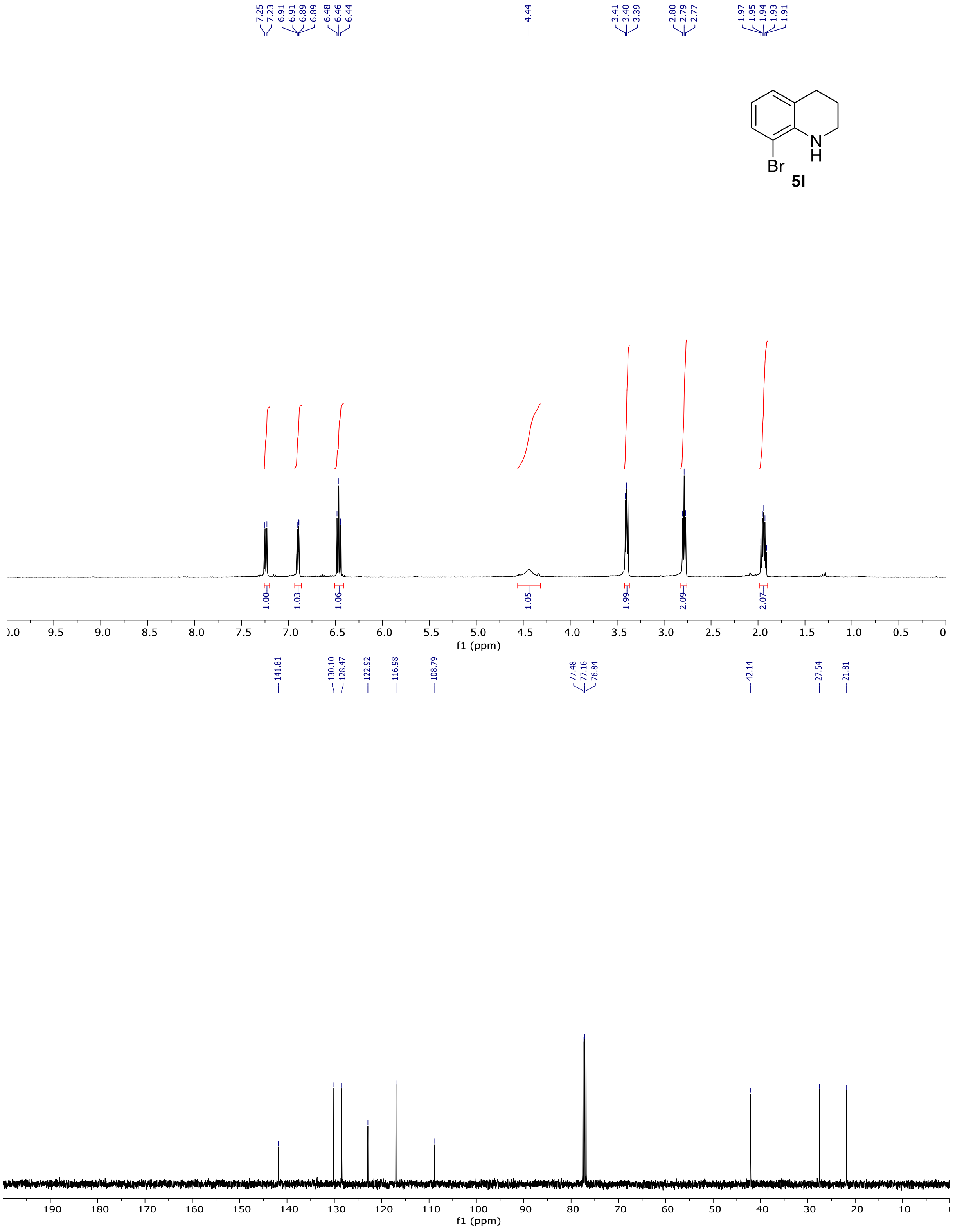

^{1}H NMR (400 MHz) and ^{13}C{^{1}H} NMR (100 MHz) spectra of **5l** ($CDCl_3$)

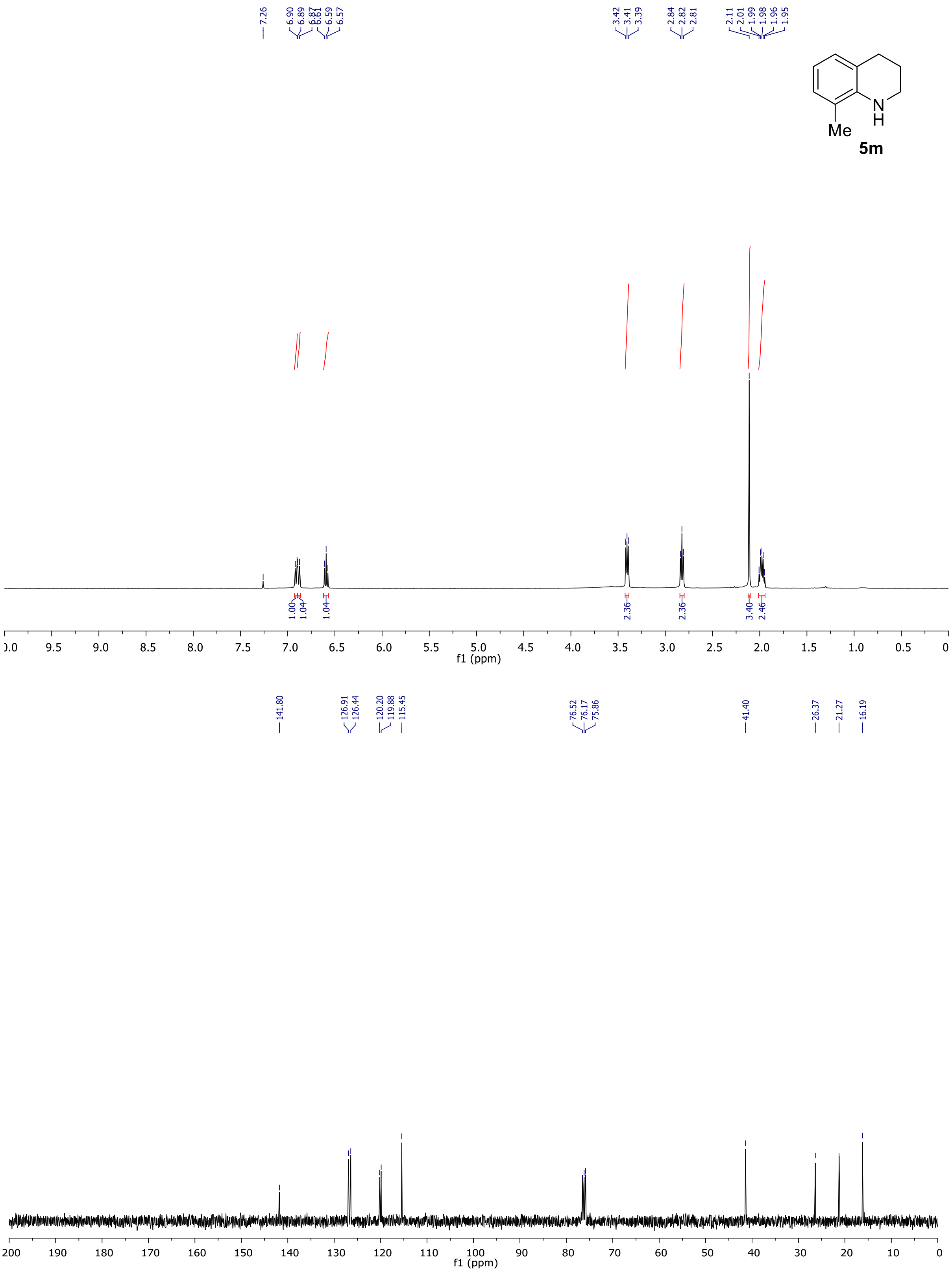

^{1}H NMR (400 MHz) and ^{13}C{^{1}H} NMR (100 MHz) spectra of **5m** ($CDCl_3$)

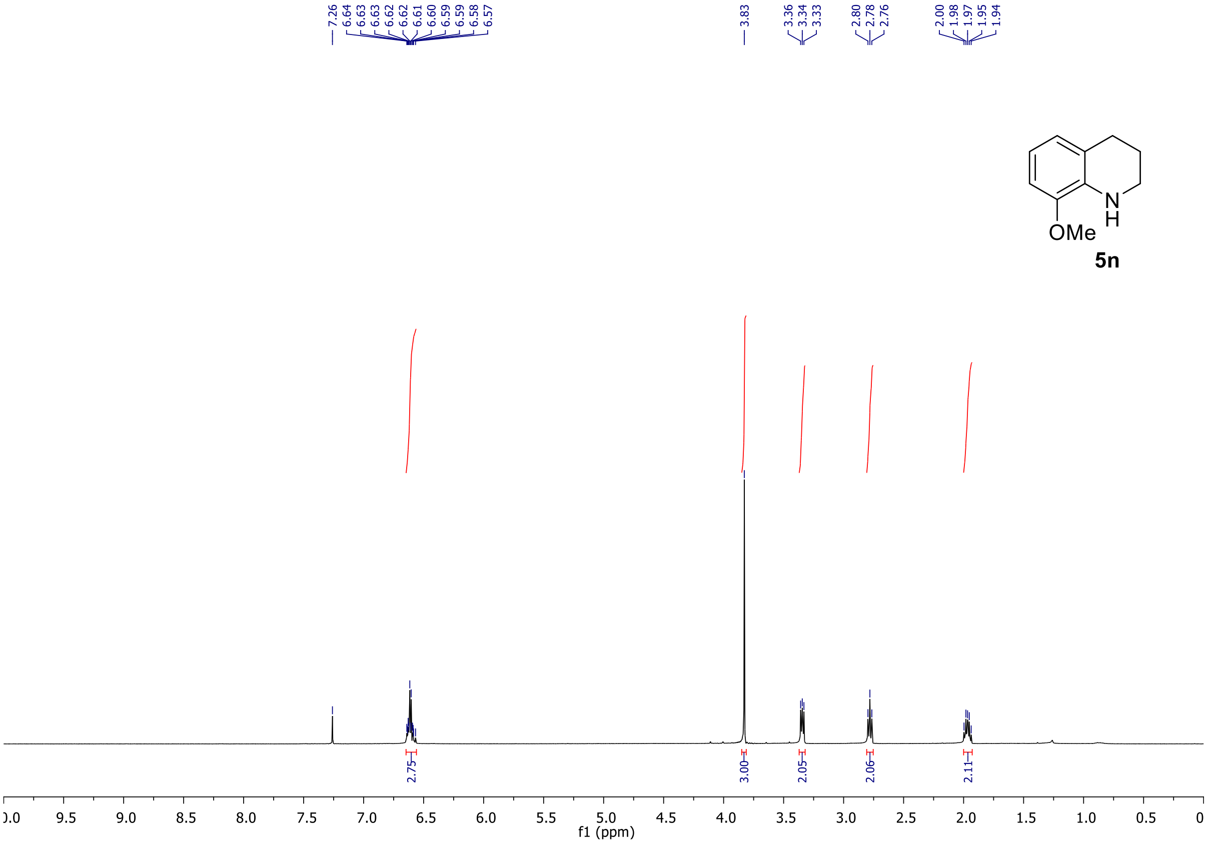

5n

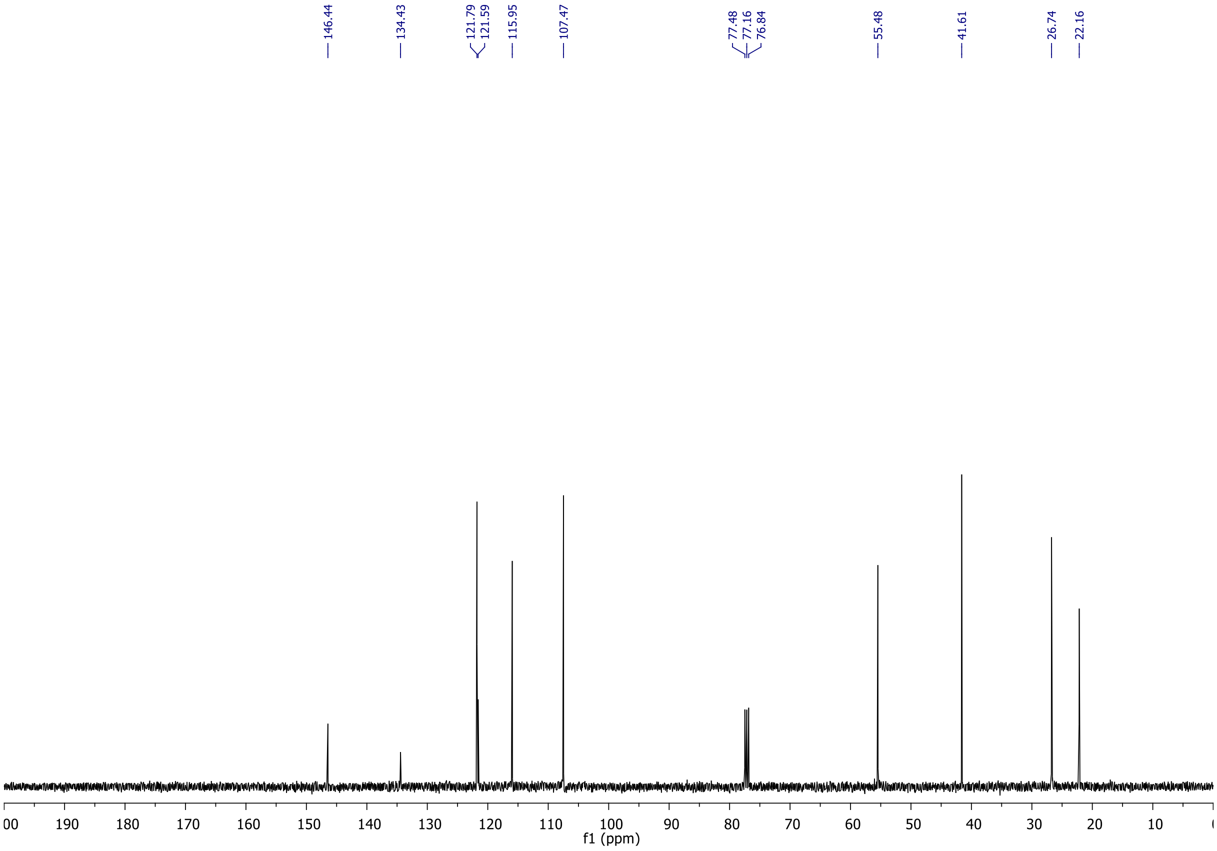

^{1}H NMR (400 MHz) and $^{13}C\{^{1}H\}$ NMR (100 MHz) spectra of **5n** ($CDCl_3$)

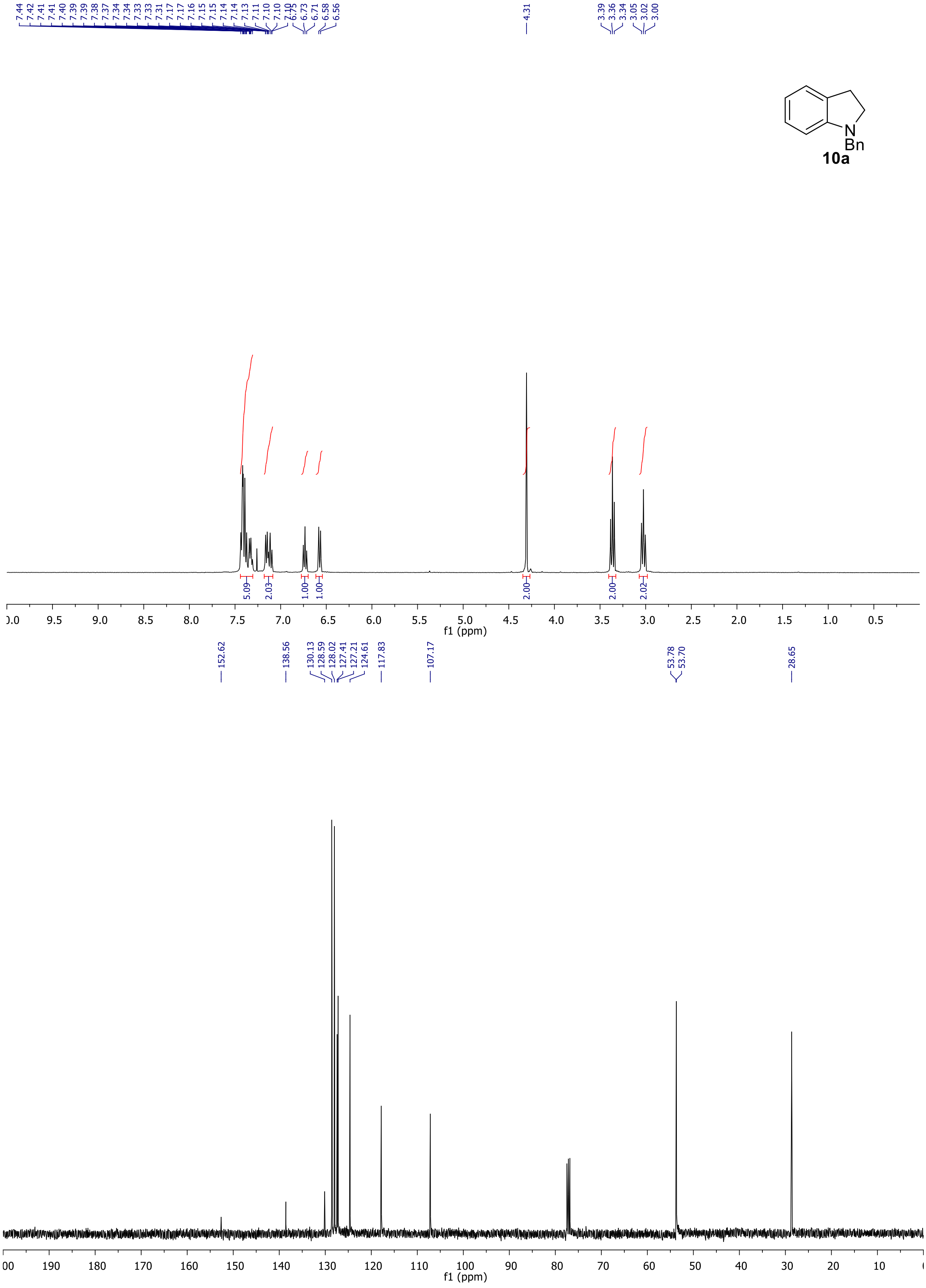

^{1}H NMR (400 MHz) and $^{13}C\{^{1}H\}$ NMR (100 MHz) spectra of **10a** ($CDCl_3$)

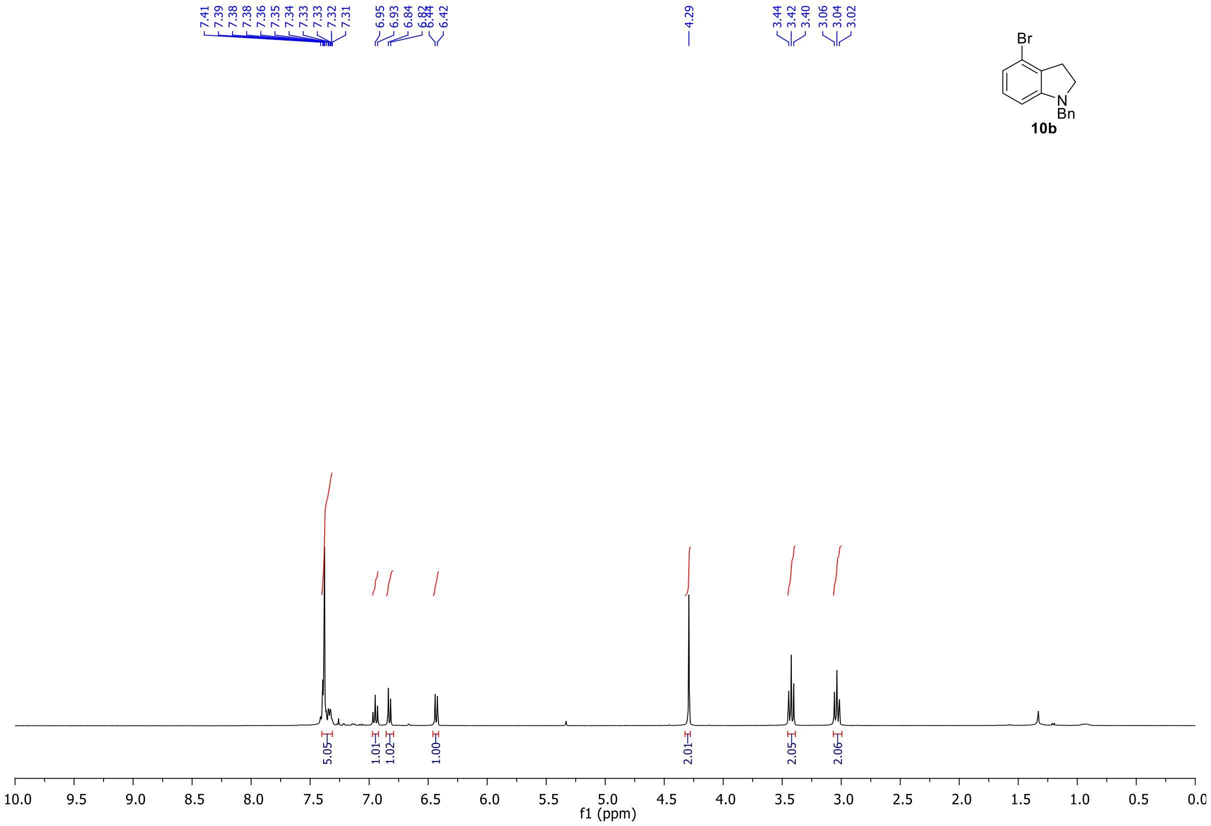

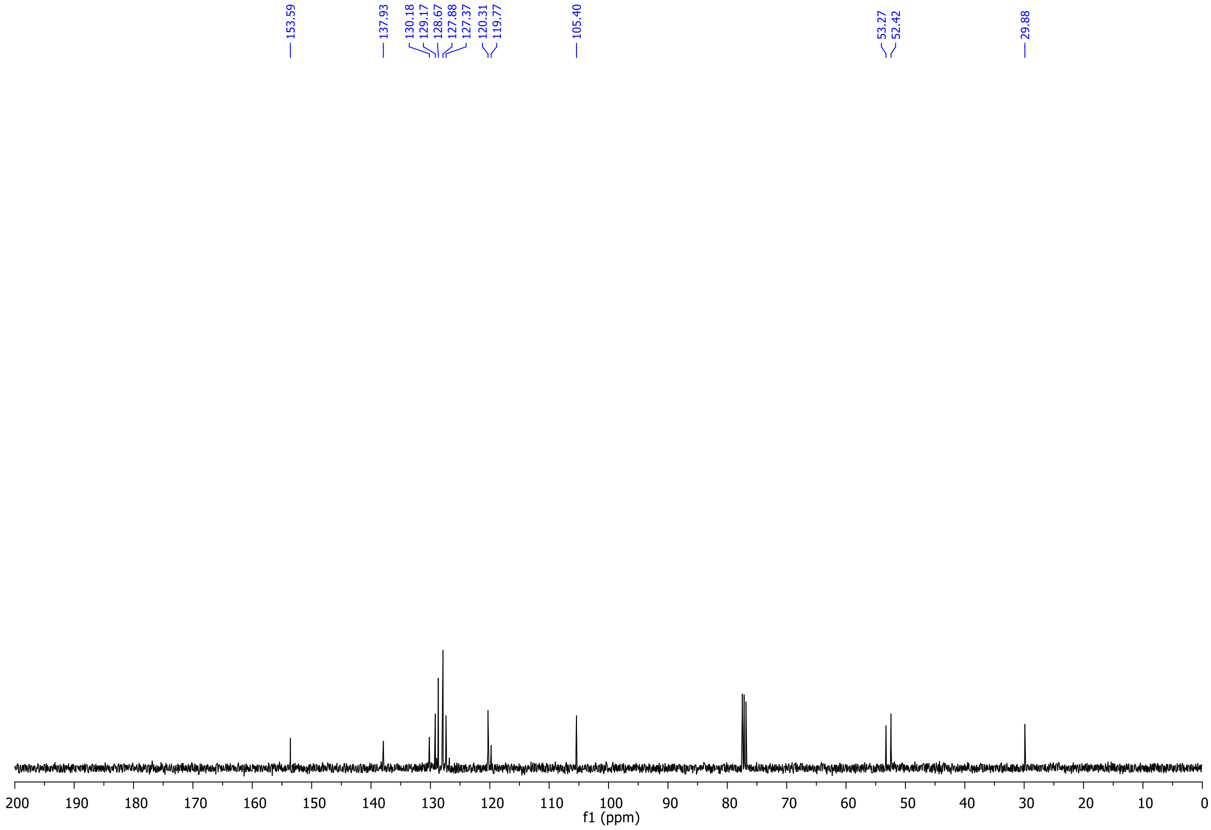

^{1}H NMR (400 MHz) and $^{13}C\{^{1}H\}$ NMR (100 MHz) spectra of **10b** ($CDCl_3$)

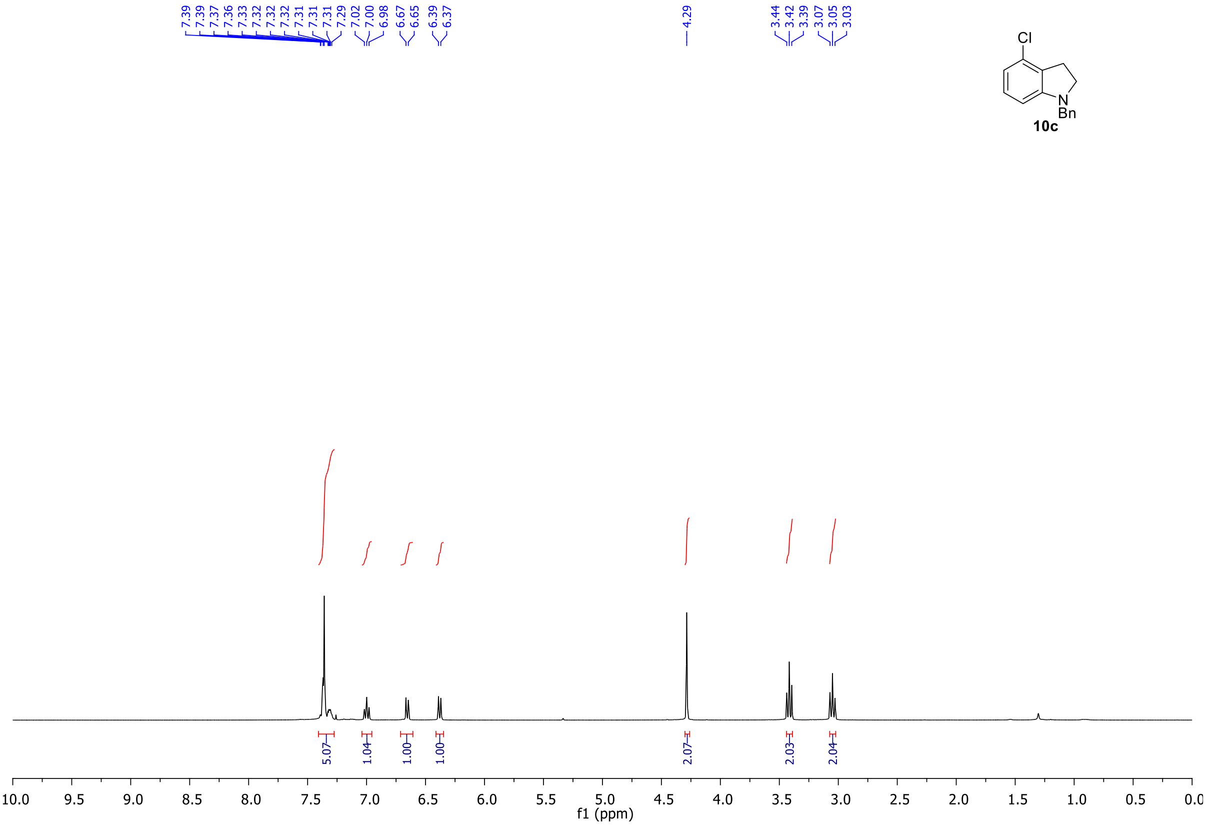

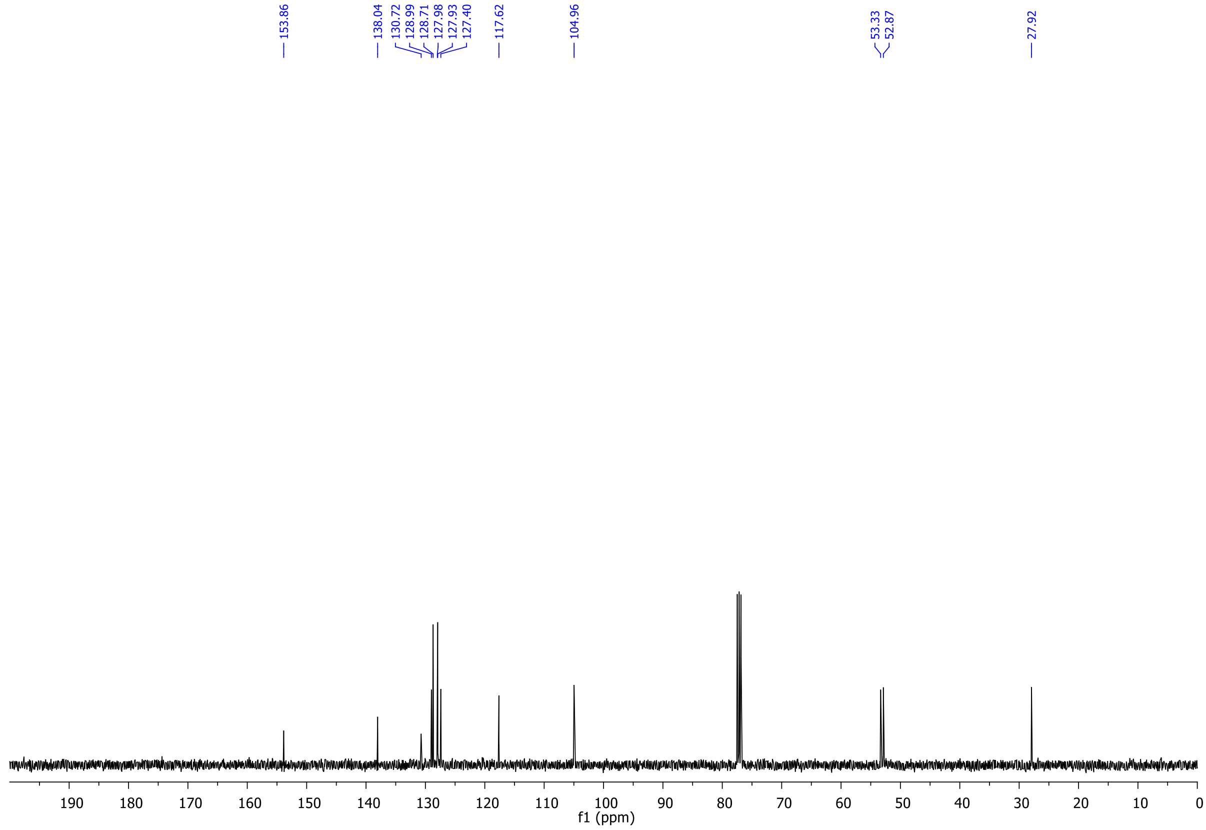

^{1}H NMR (400 MHz) and ^{13}C{^{1}H} NMR (100 MHz) spectra of **10c** ($CDCl_3$)

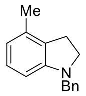

10d

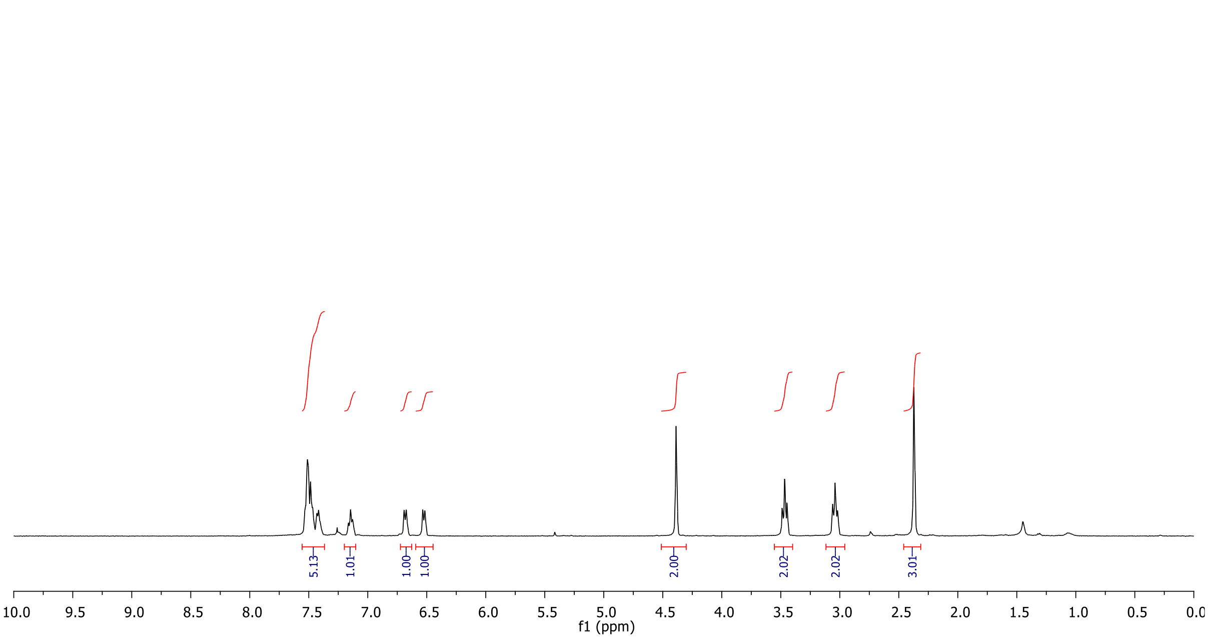

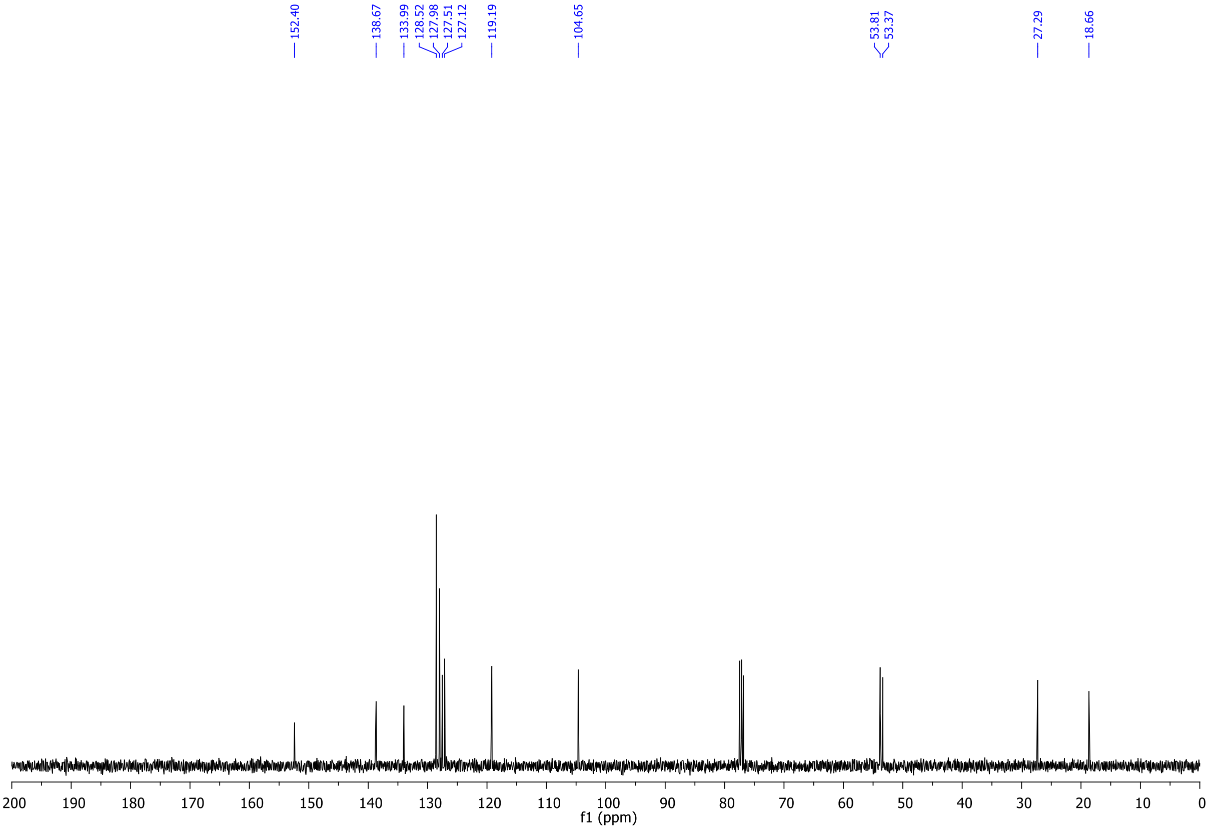

^{1}H NMR (400 MHz) and ^{13}C{^{1}H} NMR (100 MHz) spectra of **10d** ($CDCl_3$)

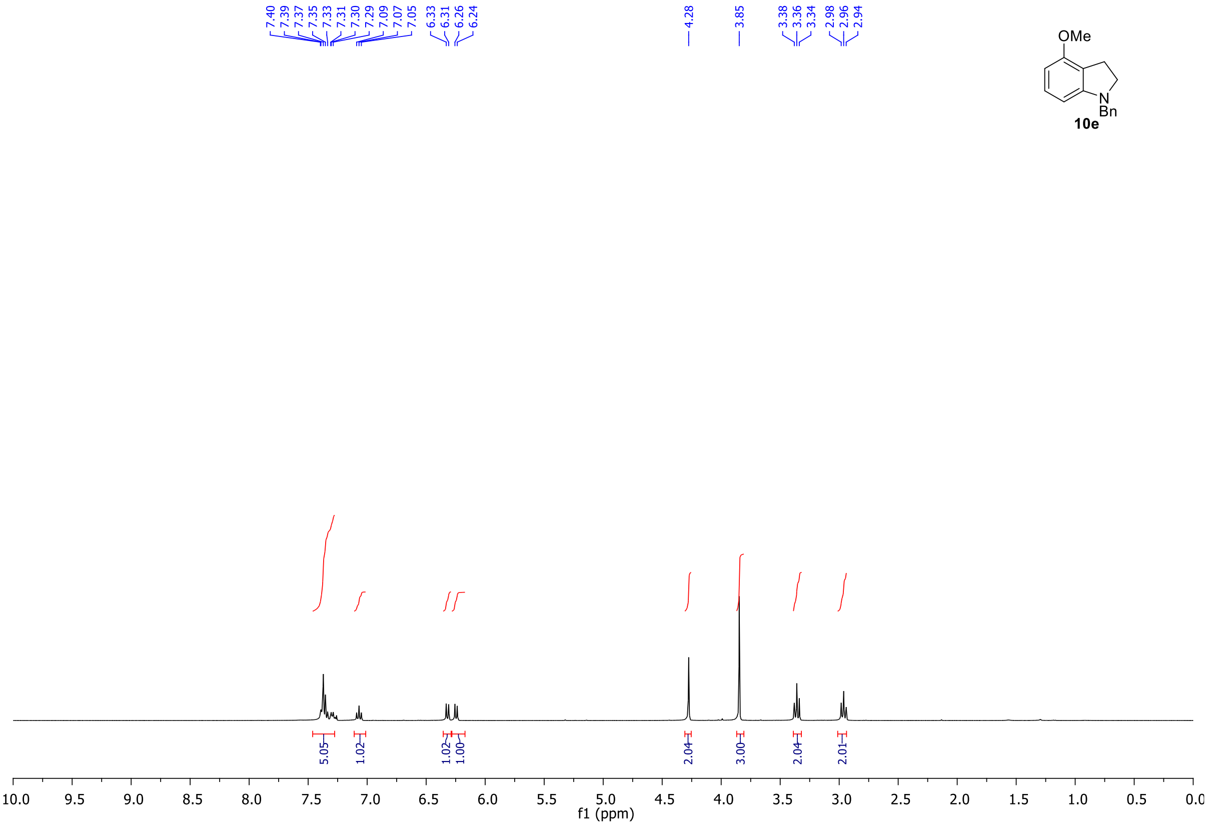

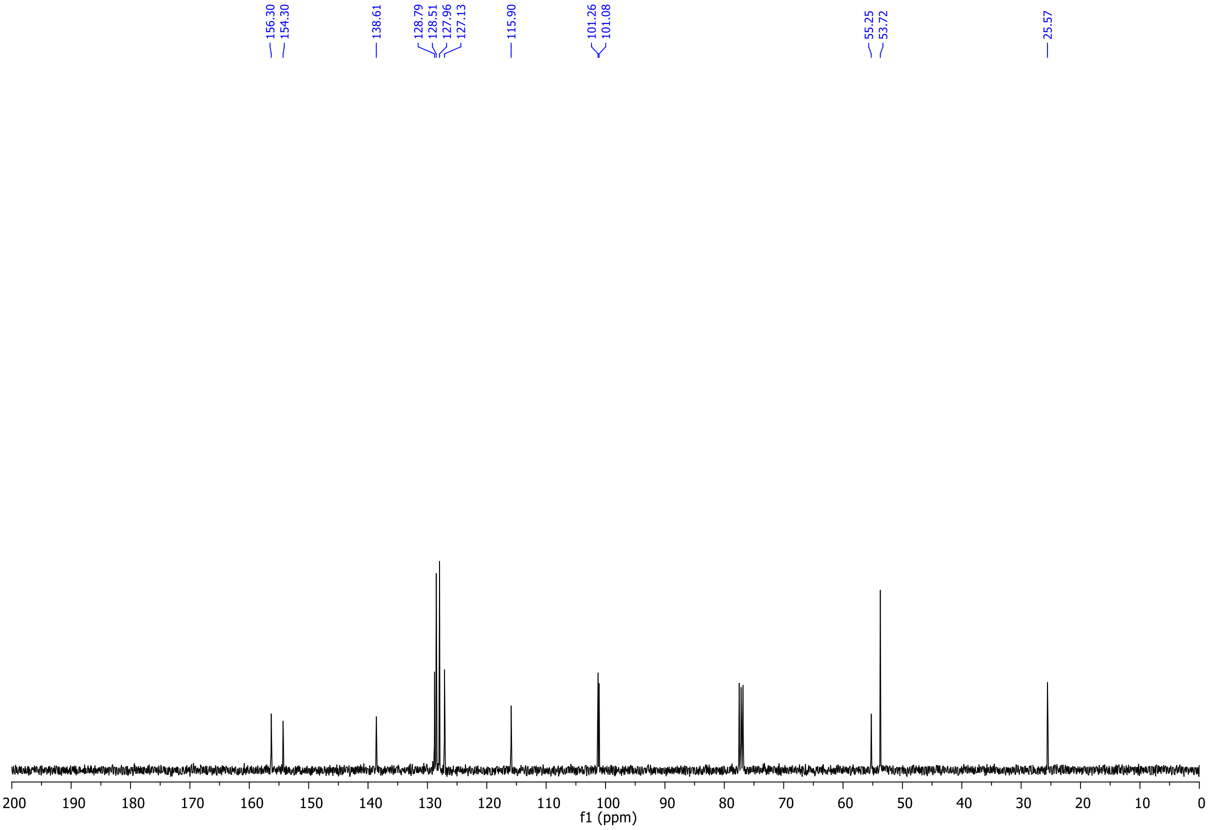

^{1}H NMR (400 MHz) and ^{13}C{^{1}H} NMR (100 MHz) spectra of **10e** ($CDCl_3$)

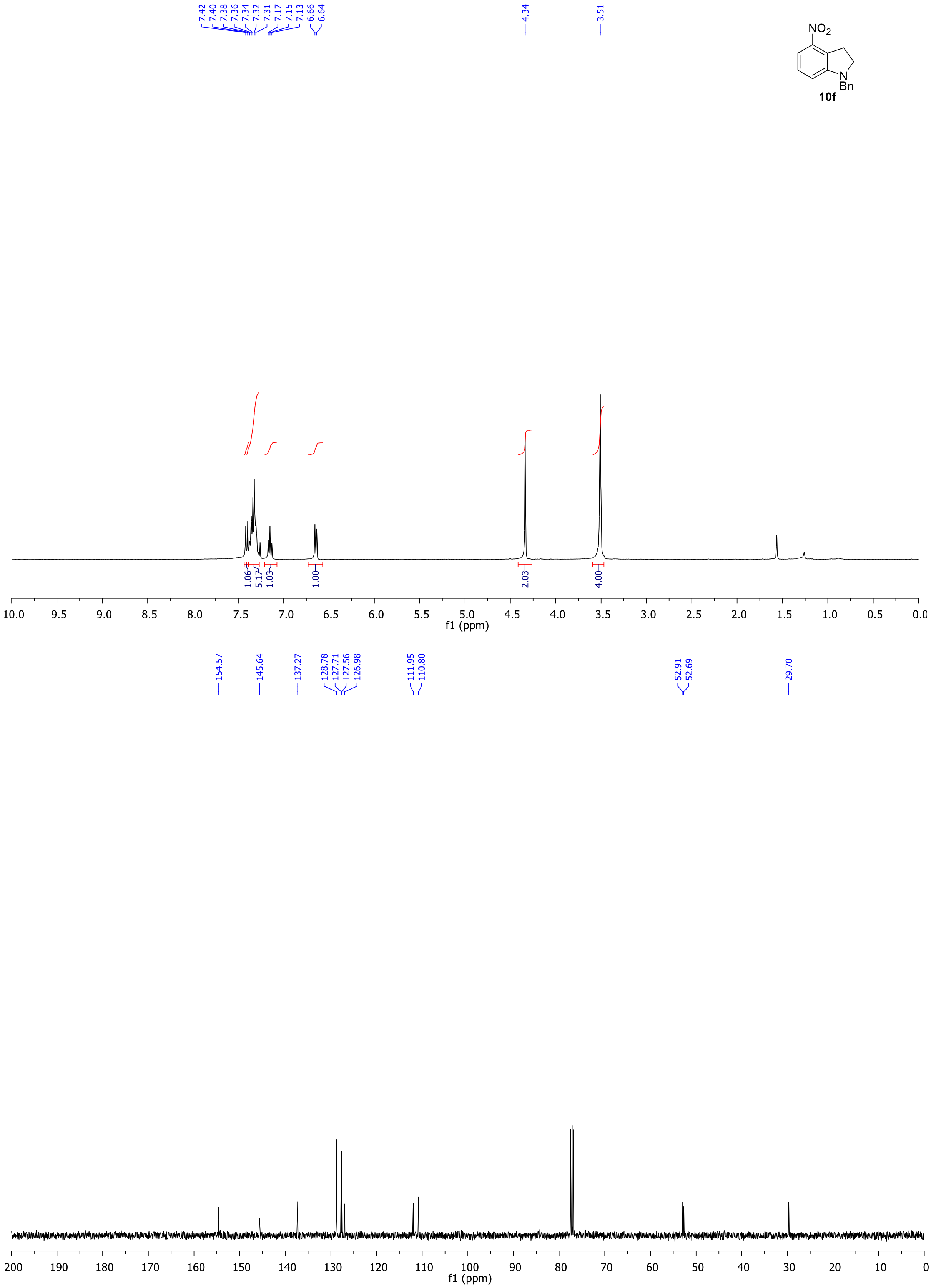

^{1}H NMR (400 MHz) and ^{13}C{^{1}H} NMR (100 MHz) spectra of **10f** ($CDCl_3$)

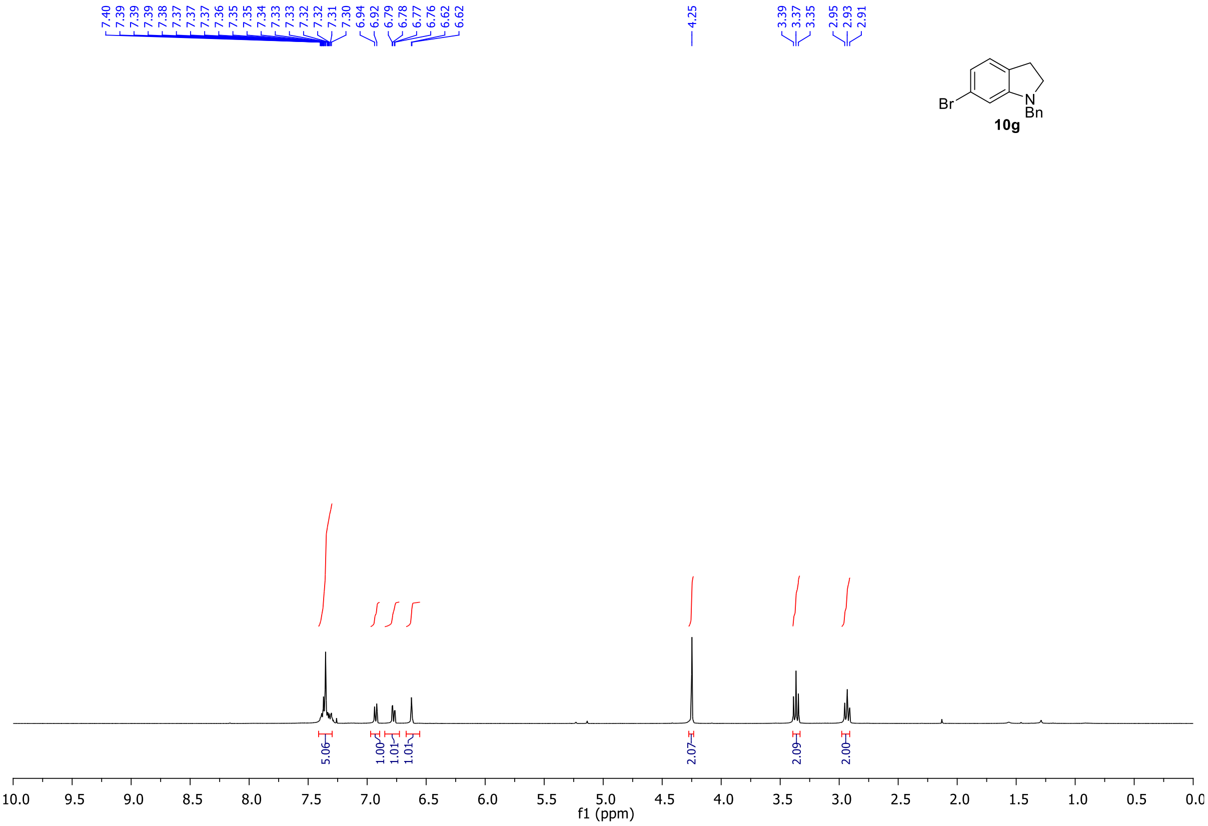

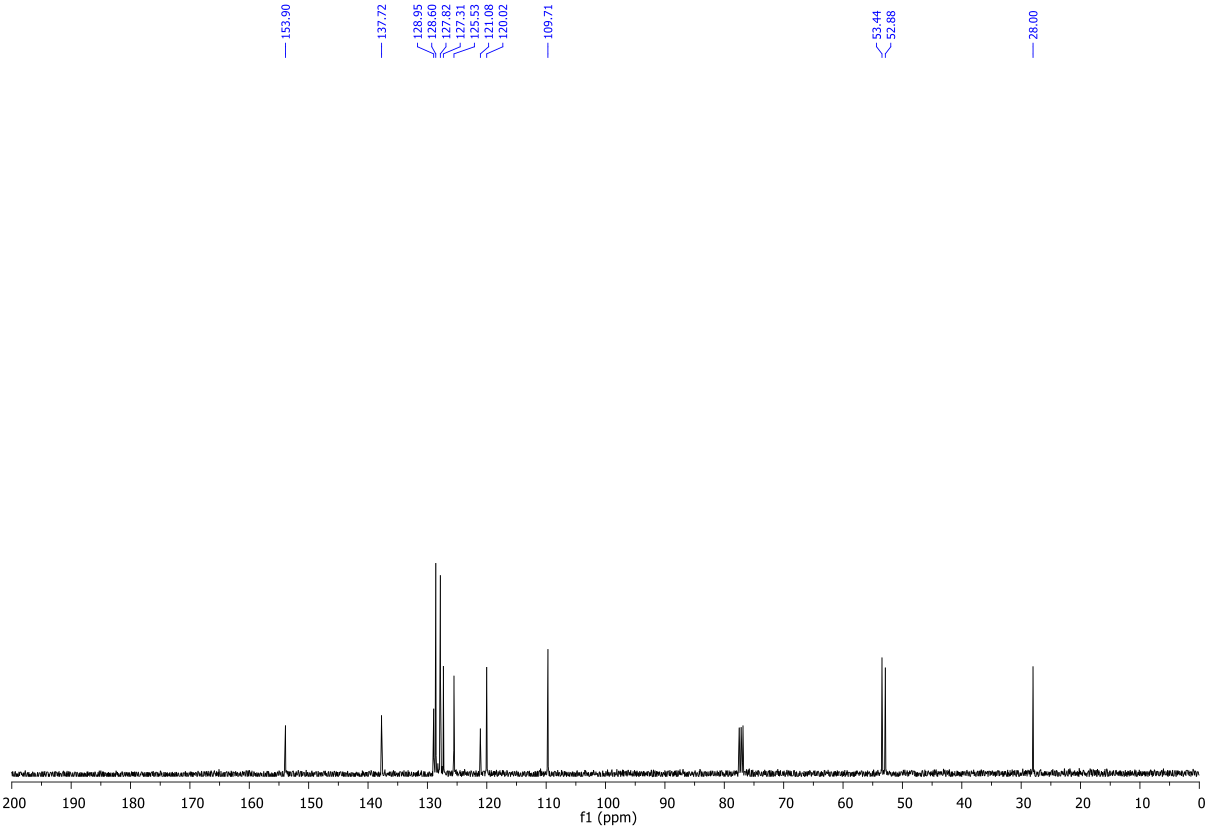

^{1}H NMR (400 MHz) and $^{13}C\{^{1}H\}$ NMR (100 MHz) spectra of **10g** ($CDCl_3$)

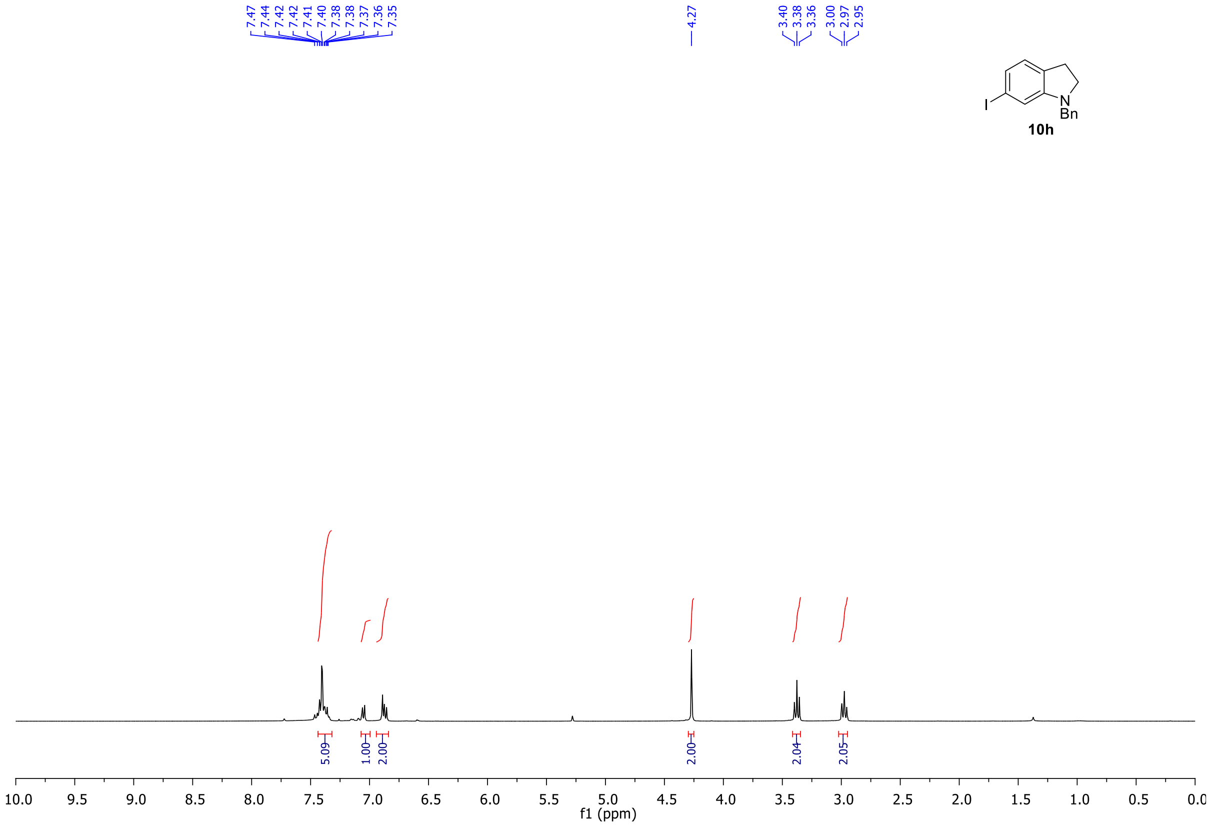

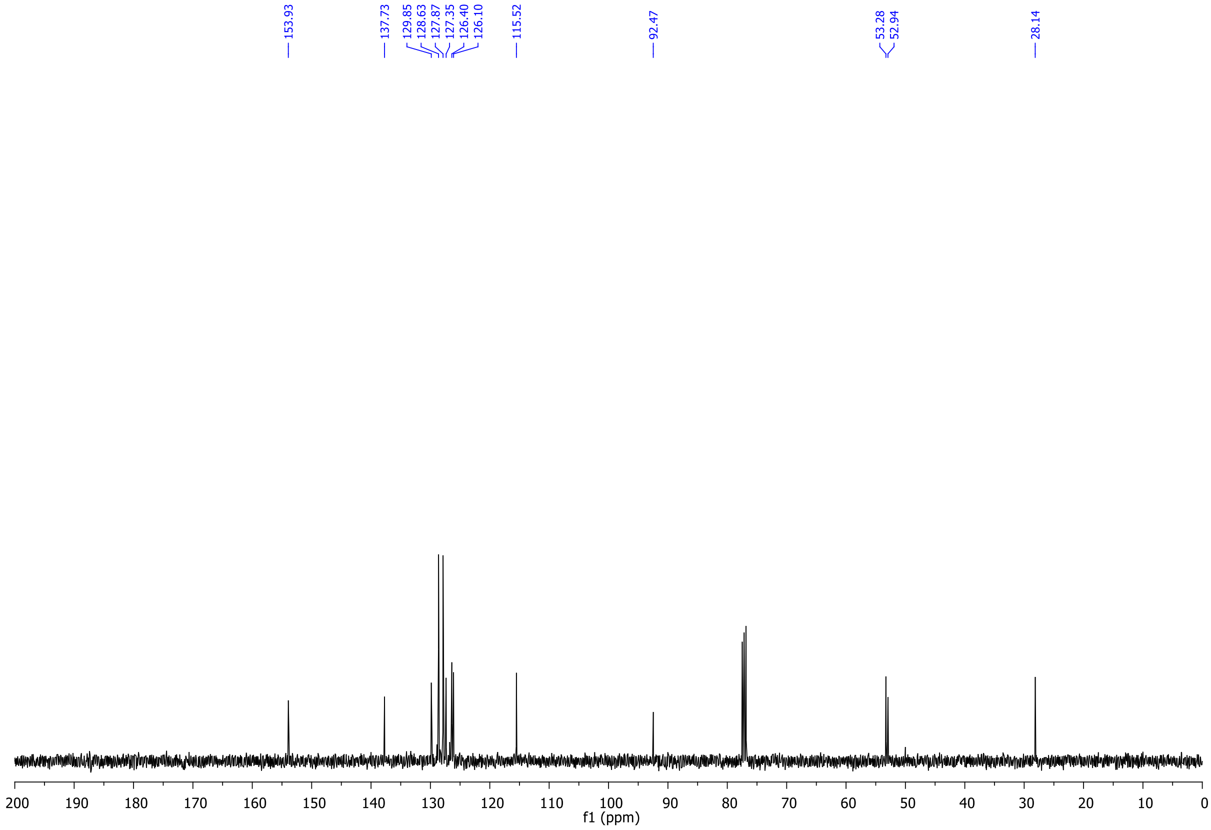

^{1}H NMR (400 MHz) and ^{13}C{^{1}H} NMR (100 MHz) spectra of **10h** ($CDCl_3$)

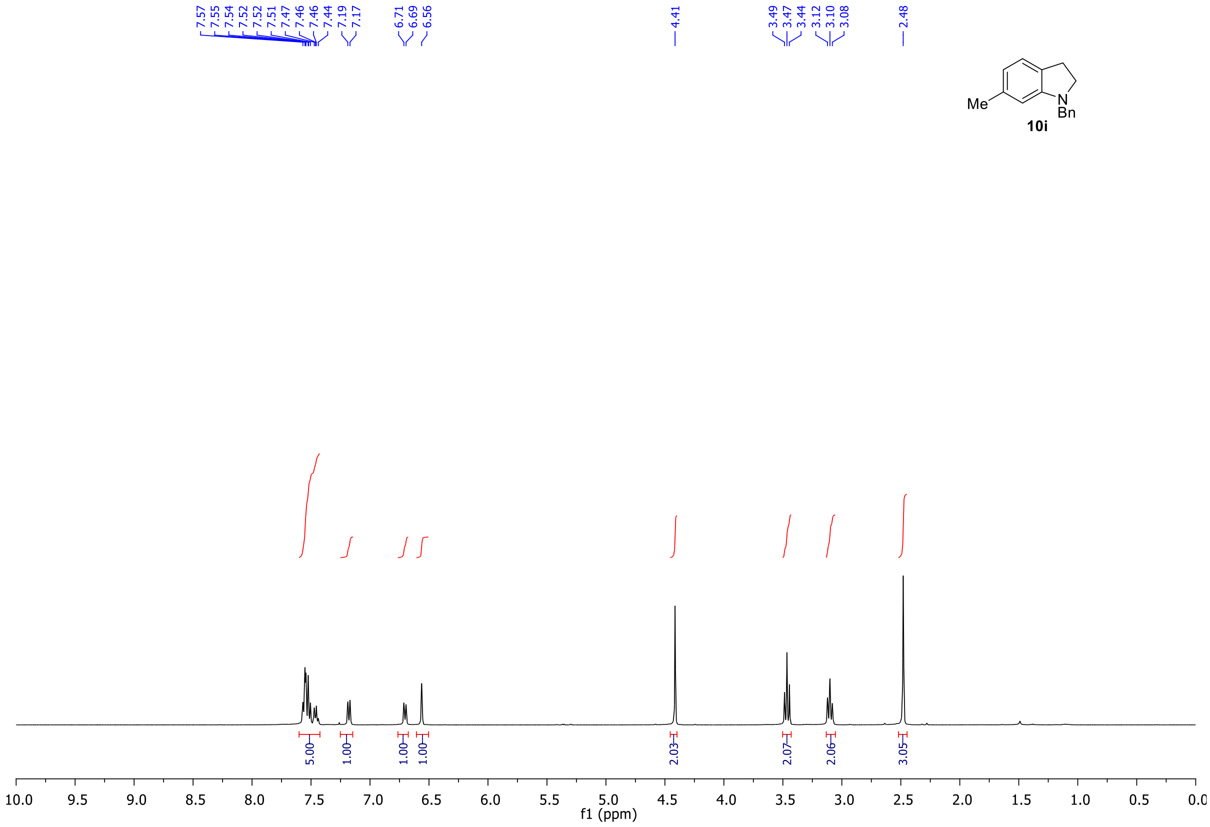

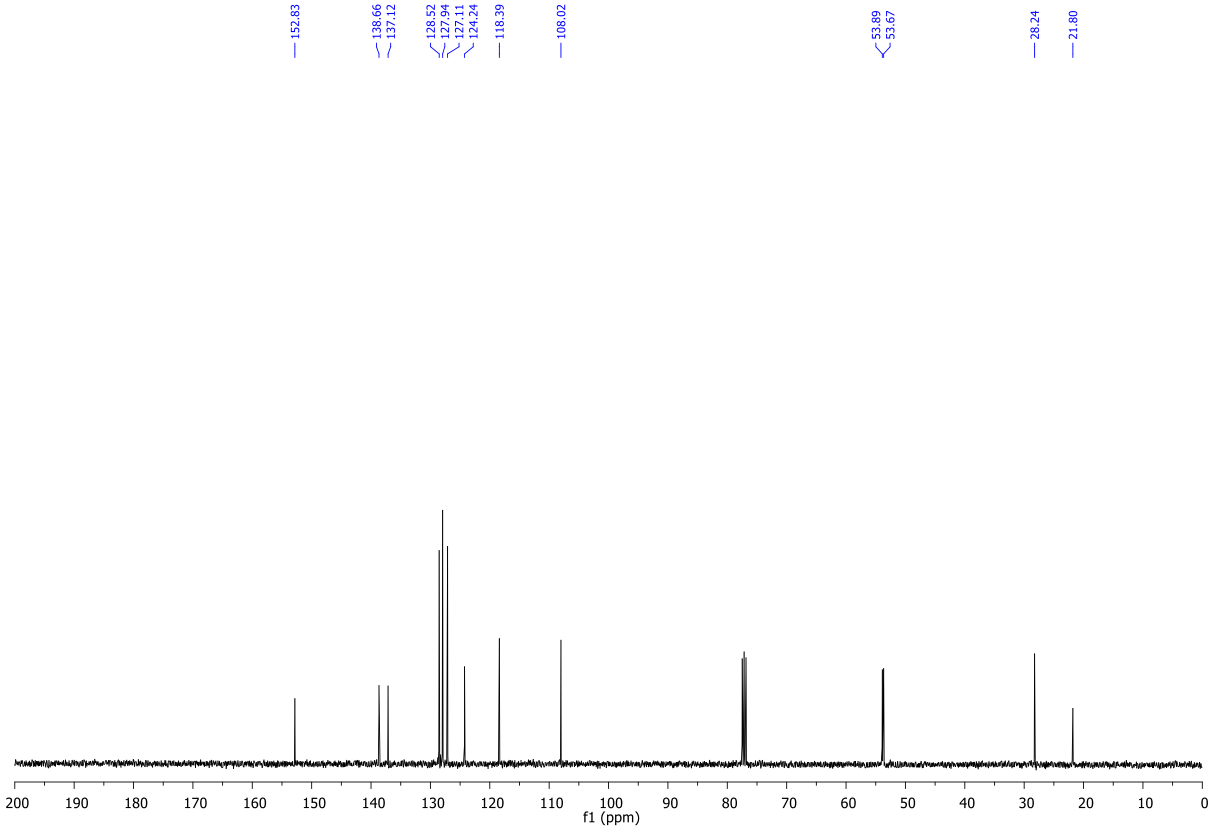

^{1}H NMR (400 MHz) and ^{13}C{^{1}H} NMR (100 MHz) spectra of **10i** ($CDCl_3$)

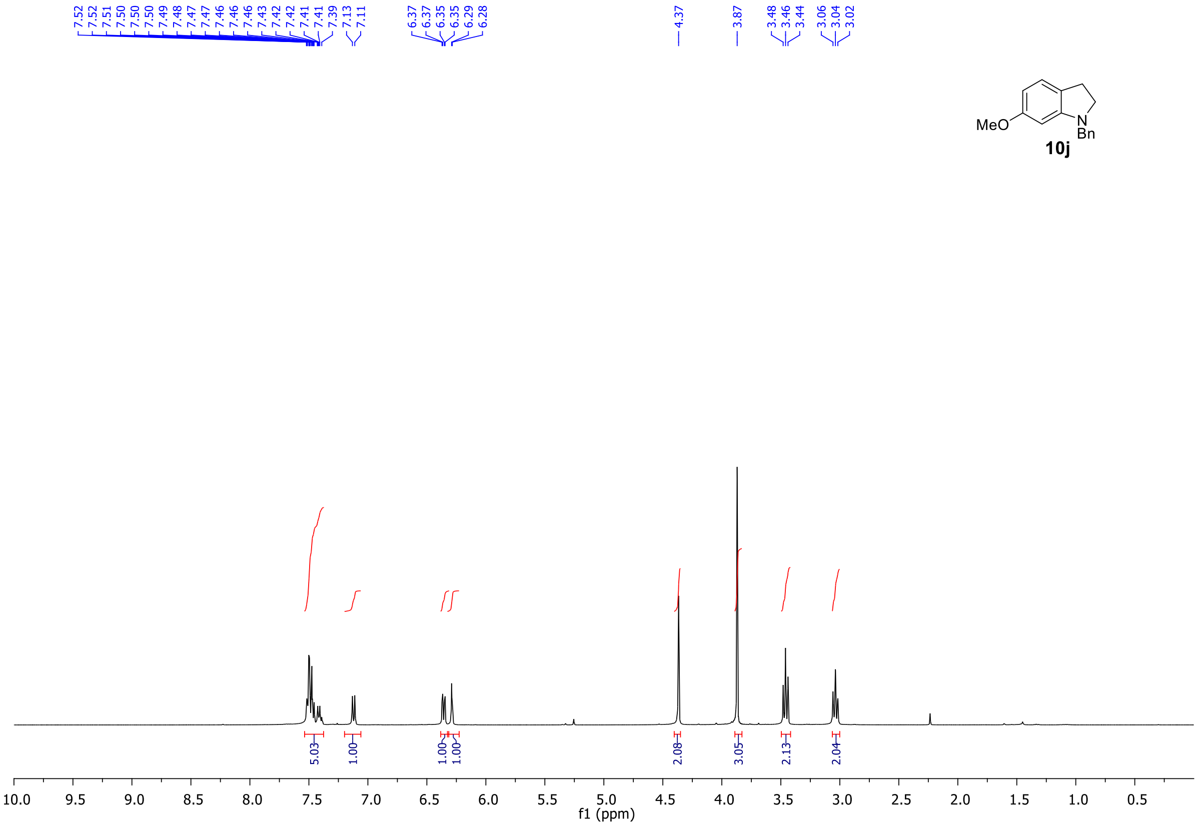

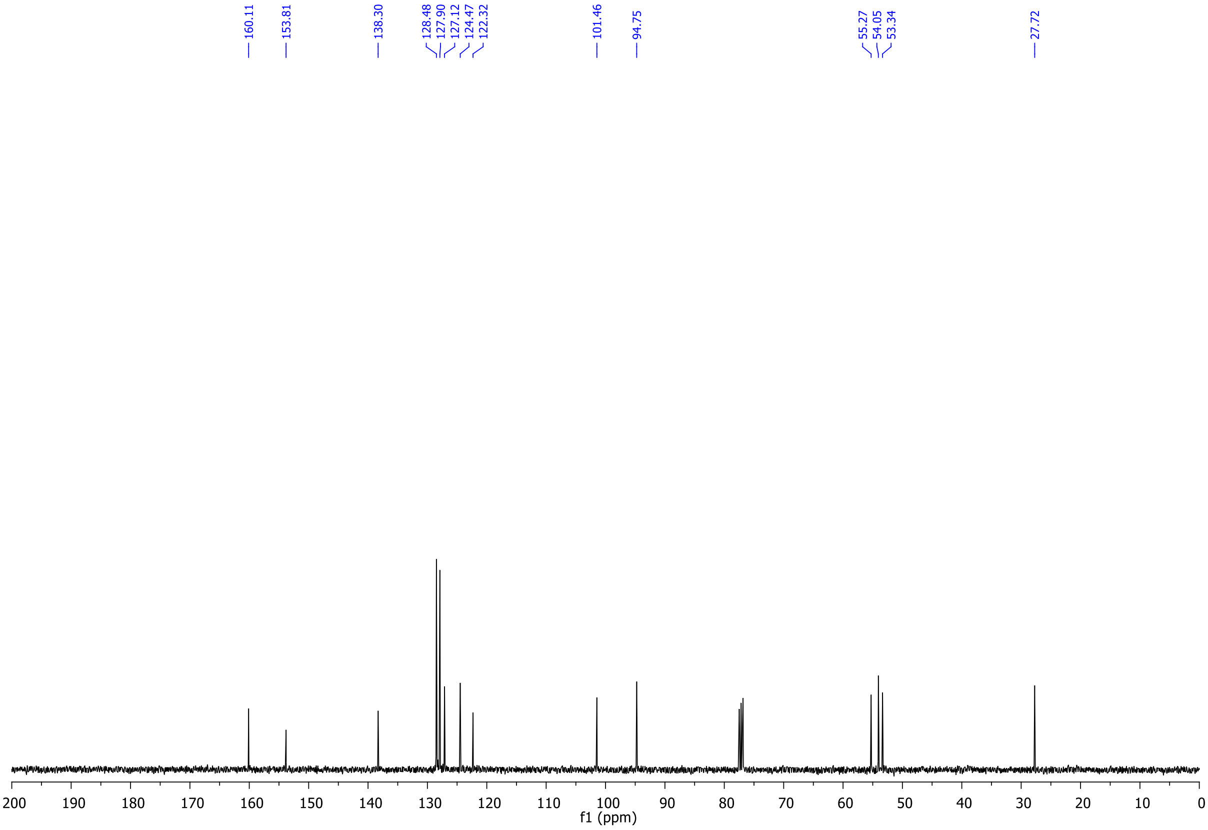

^{1}H NMR (400 MHz) and $^{13}C\{^{1}H\}$ NMR (100 MHz) spectra of **10j** ($CDCl_3$)

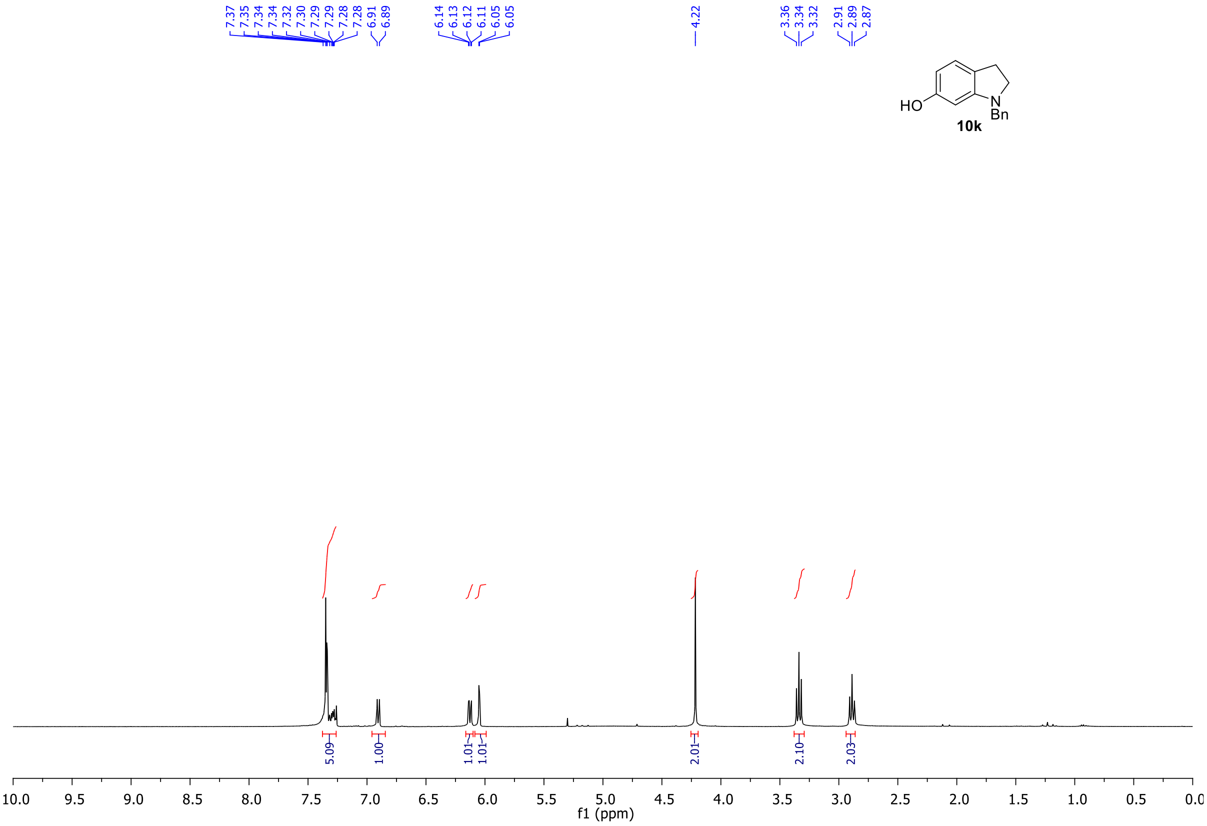

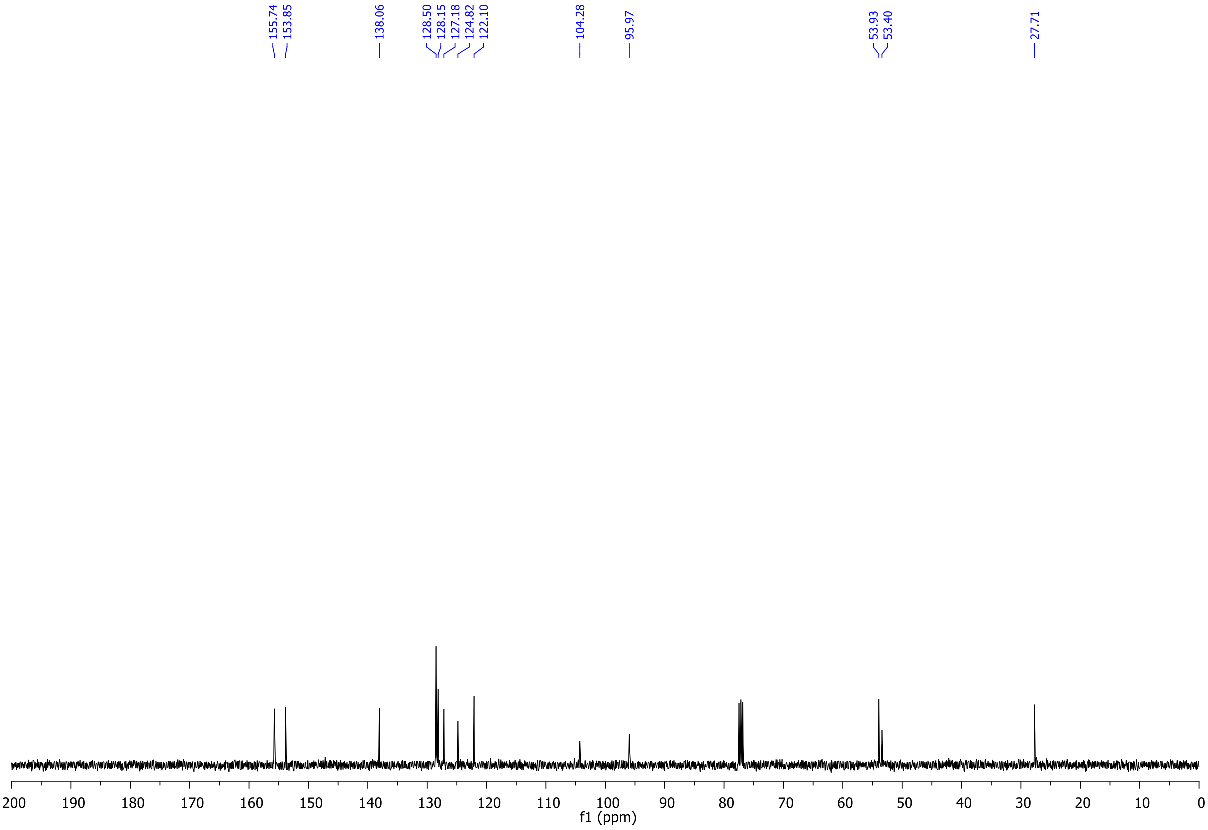

^{1}H NMR (400 MHz) and ^{13}C{^{1}H} NMR (100 MHz) spectra of **10k** ($CDCl_3$)

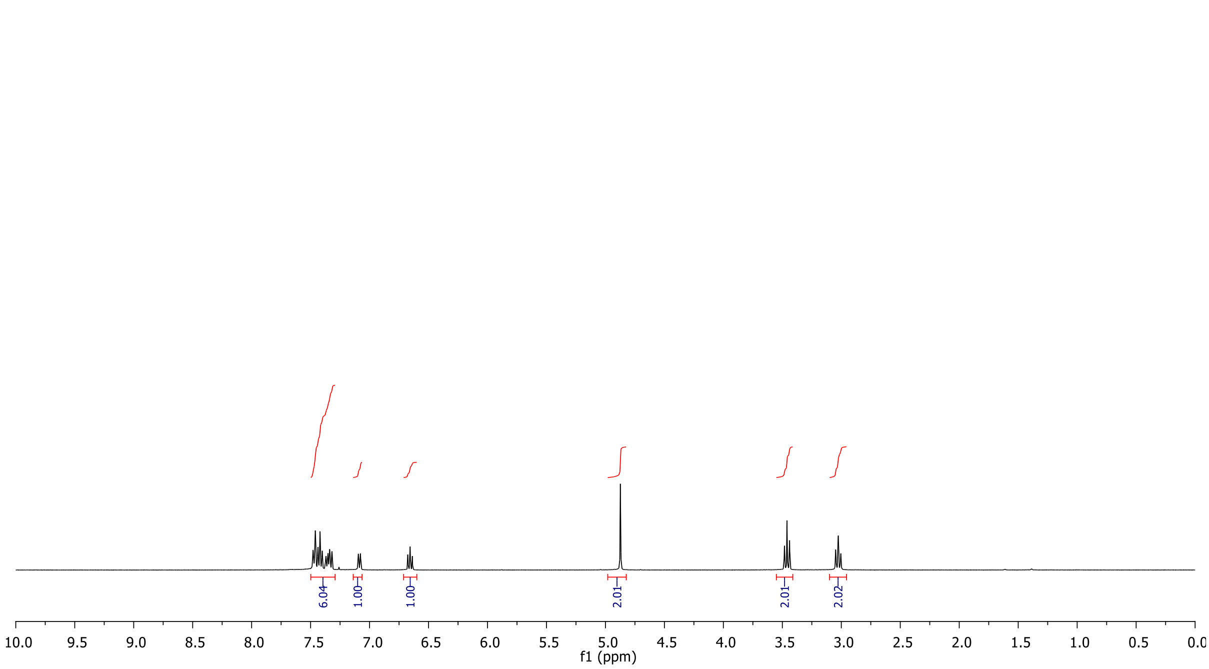

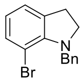

10l

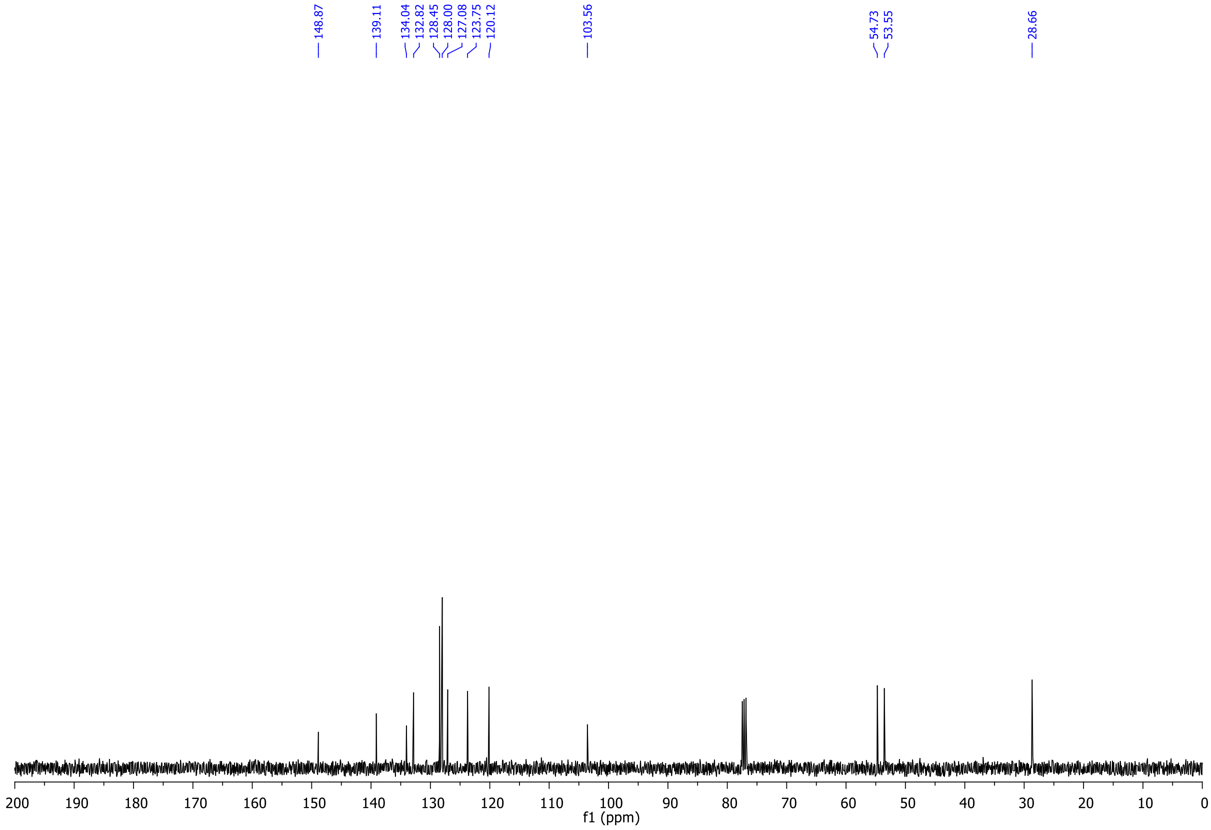

^{1}H NMR (400 MHz) and $^{13}C\{^{1}H\}$ NMR (100 MHz) spectra of **10l** ($CDCl_3$)

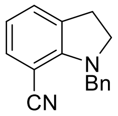

10m

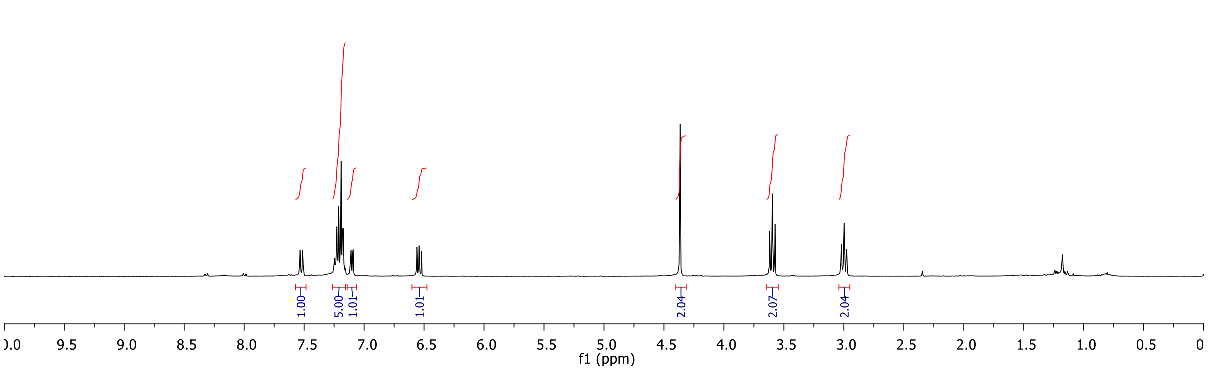

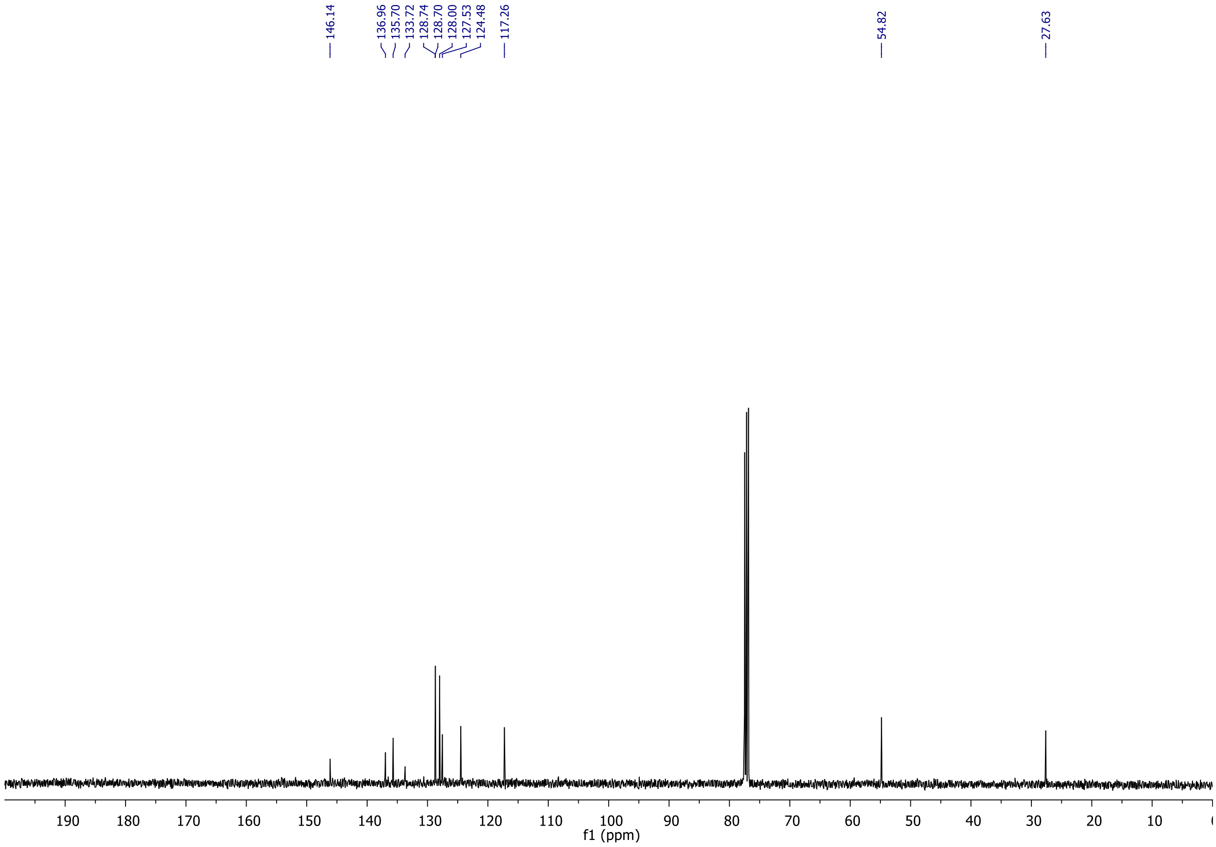

^{1}H NMR (400 MHz) and ^{13}C{^{1}H} NMR (100 MHz) spectra of **10m** ($CDCl_3$)

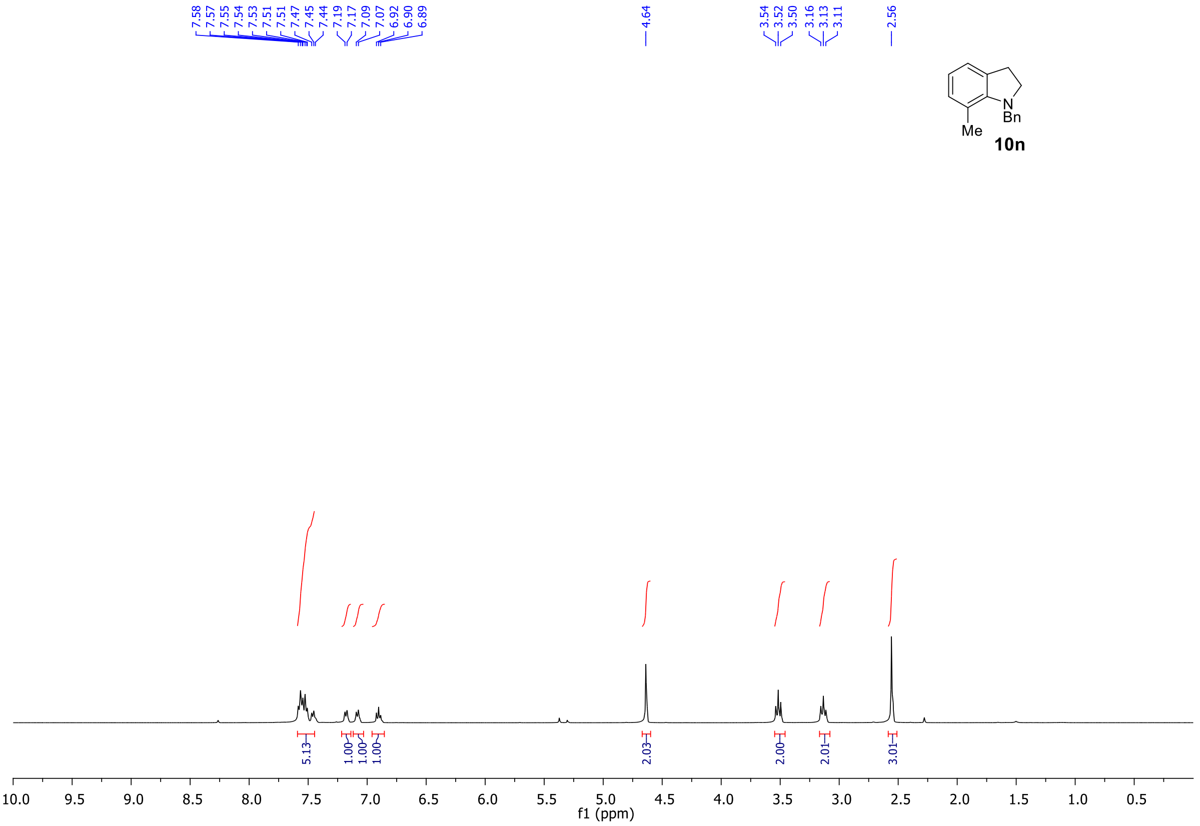

10n

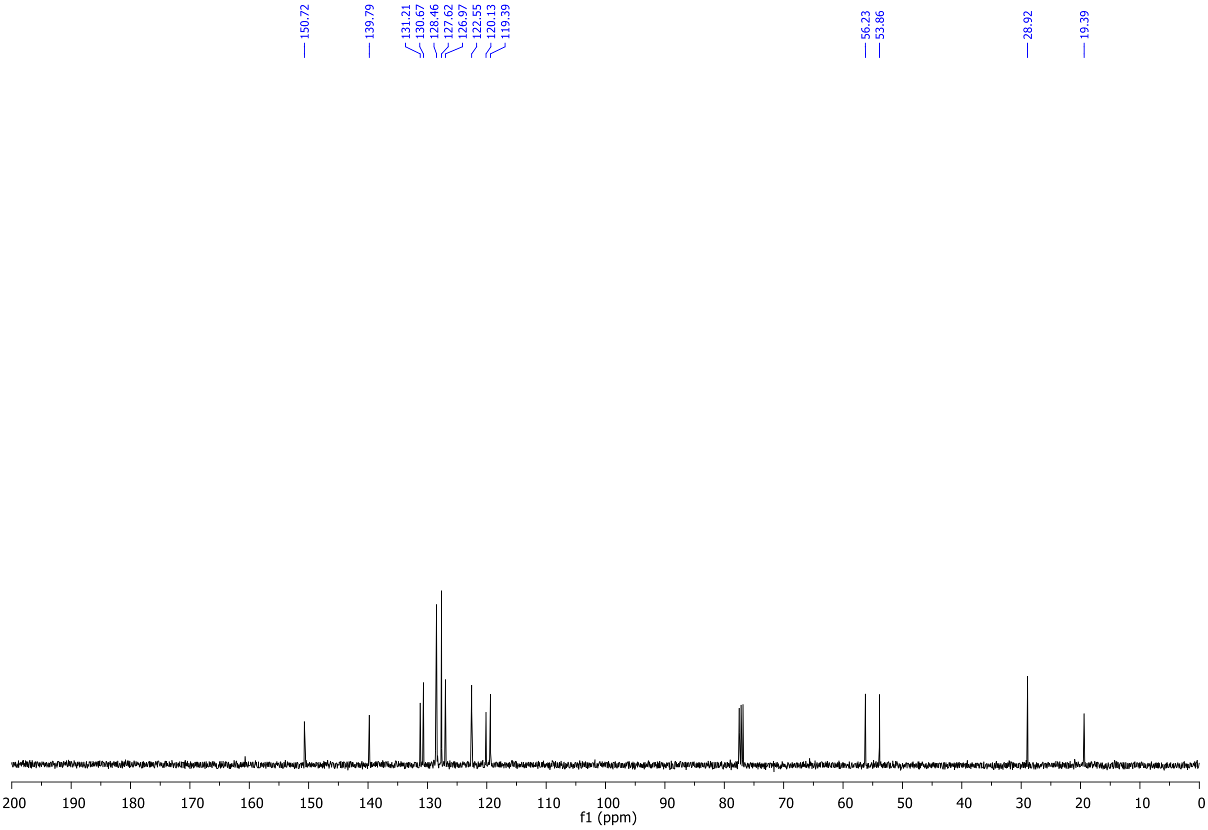

^{1}H NMR (400 MHz) and ^{13}C{^{1}H} NMR (100 MHz) spectra of **10n** ($CDCl_3$)

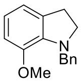

10o

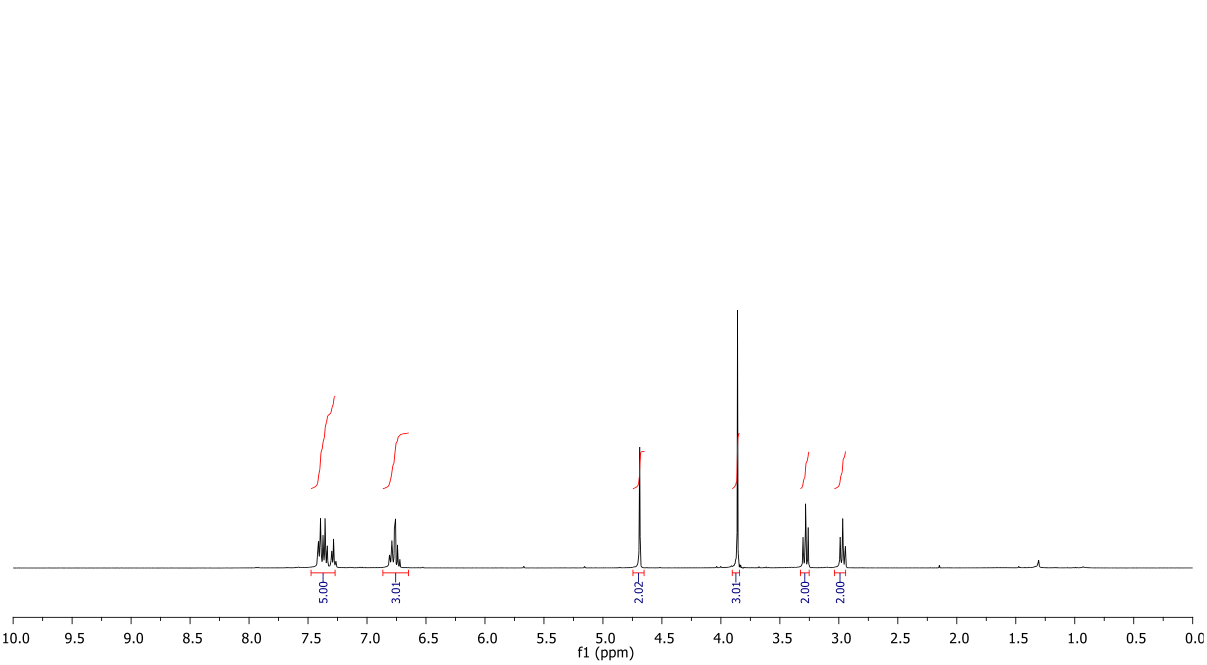

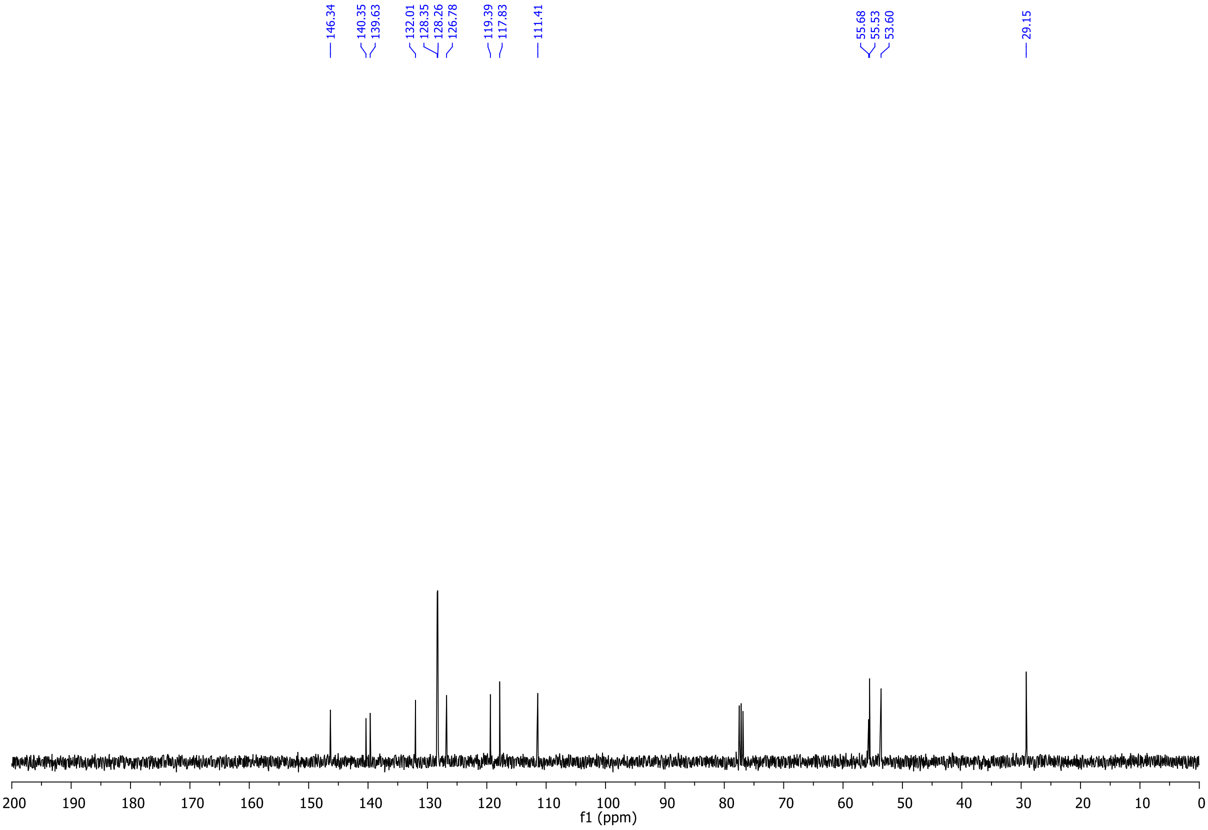

^{1}H NMR (400 MHz) and $^{13}C\{^{1}H\}$ NMR (100 MHz) spectra of **10o** ($CDCl_3$)

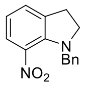

10p

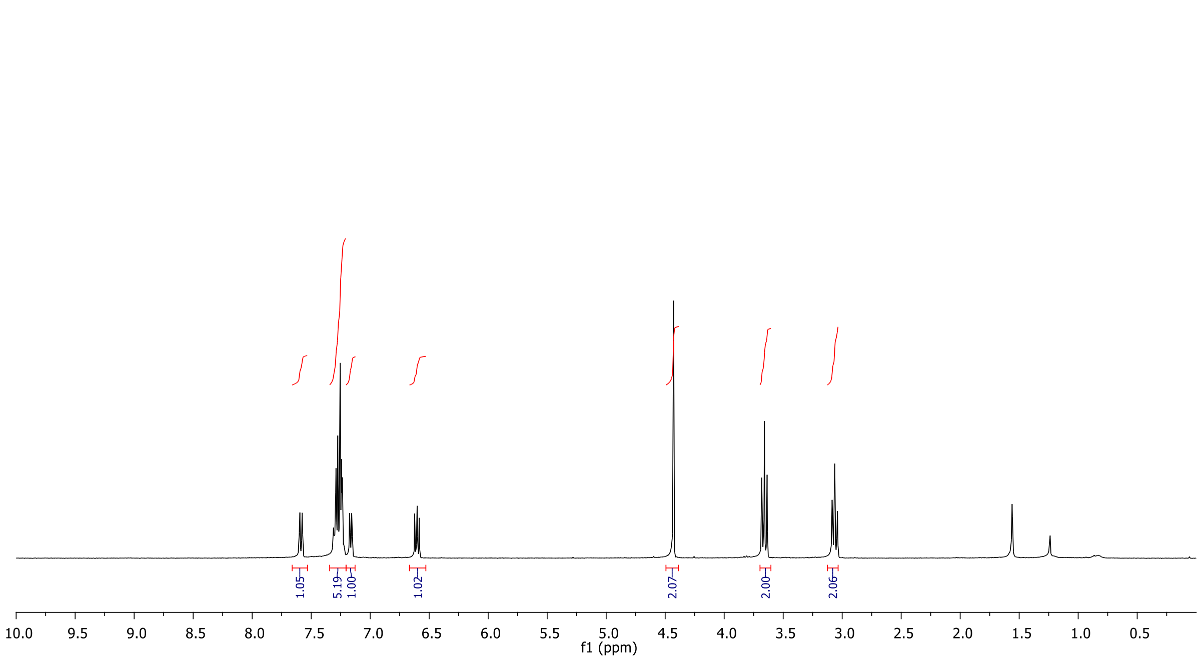

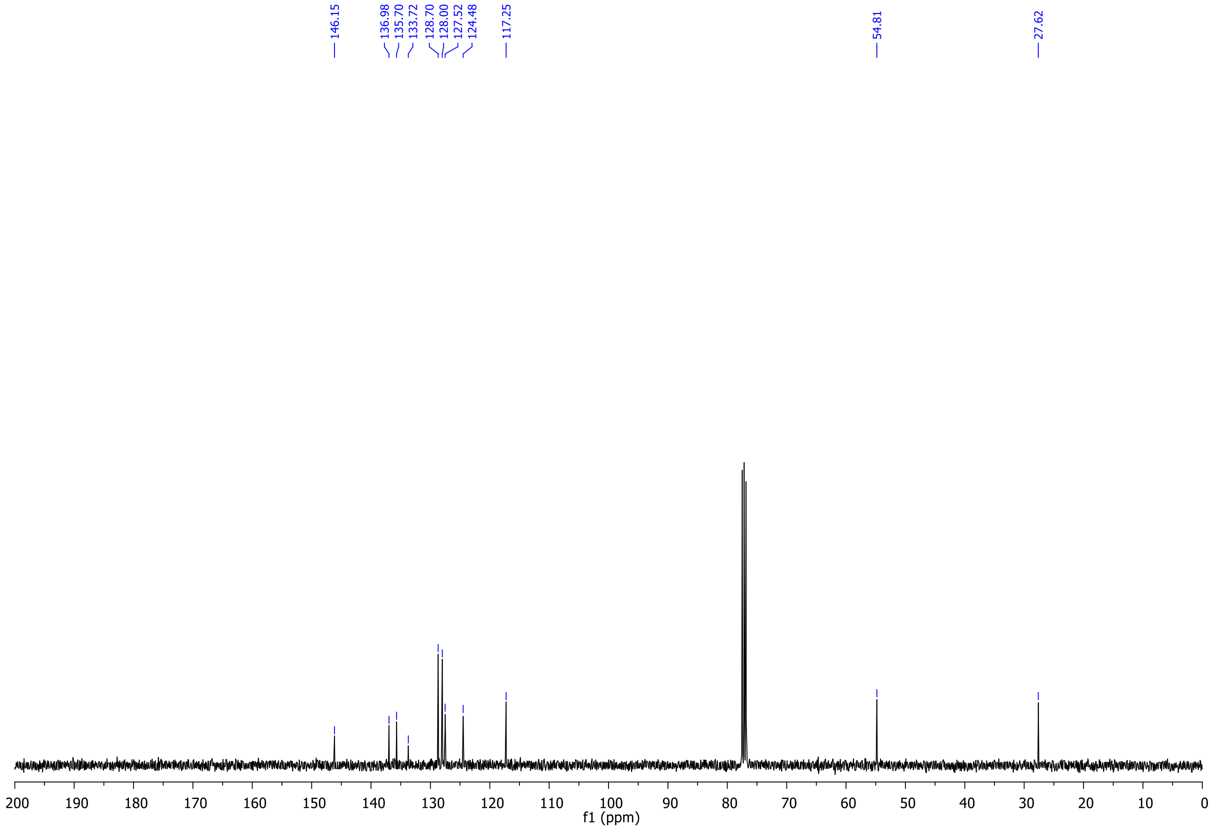

^{1}H NMR (400 MHz) and $^{13}C\{^{1}H\}$ NMR (100 MHz) spectra of **10p** ($CDCl_3$)

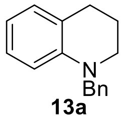

13a

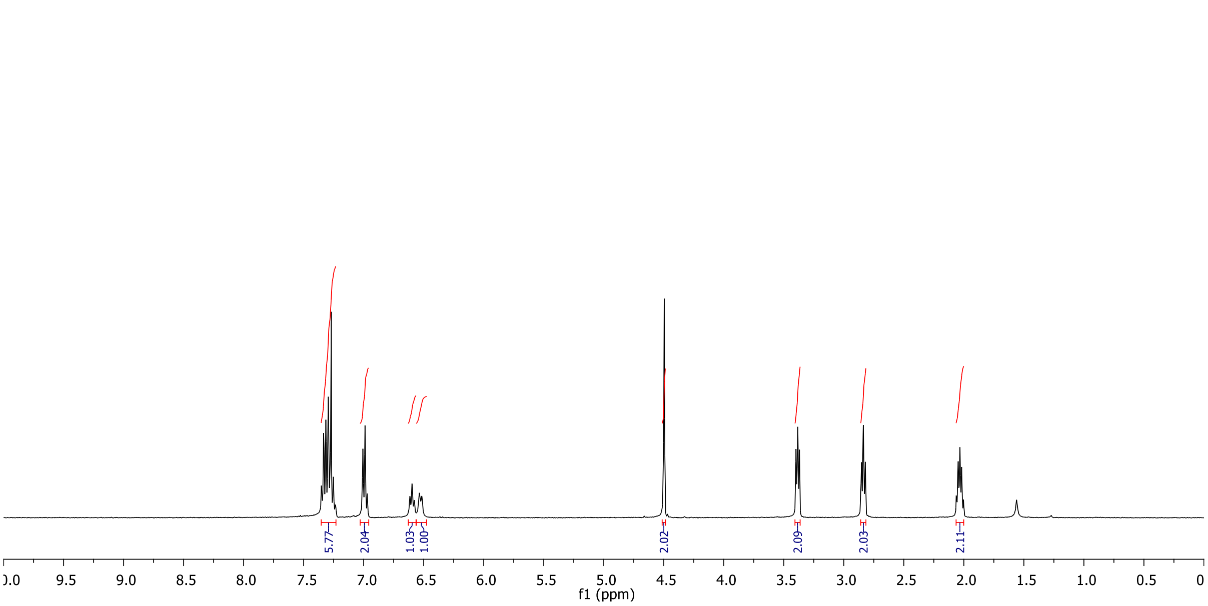

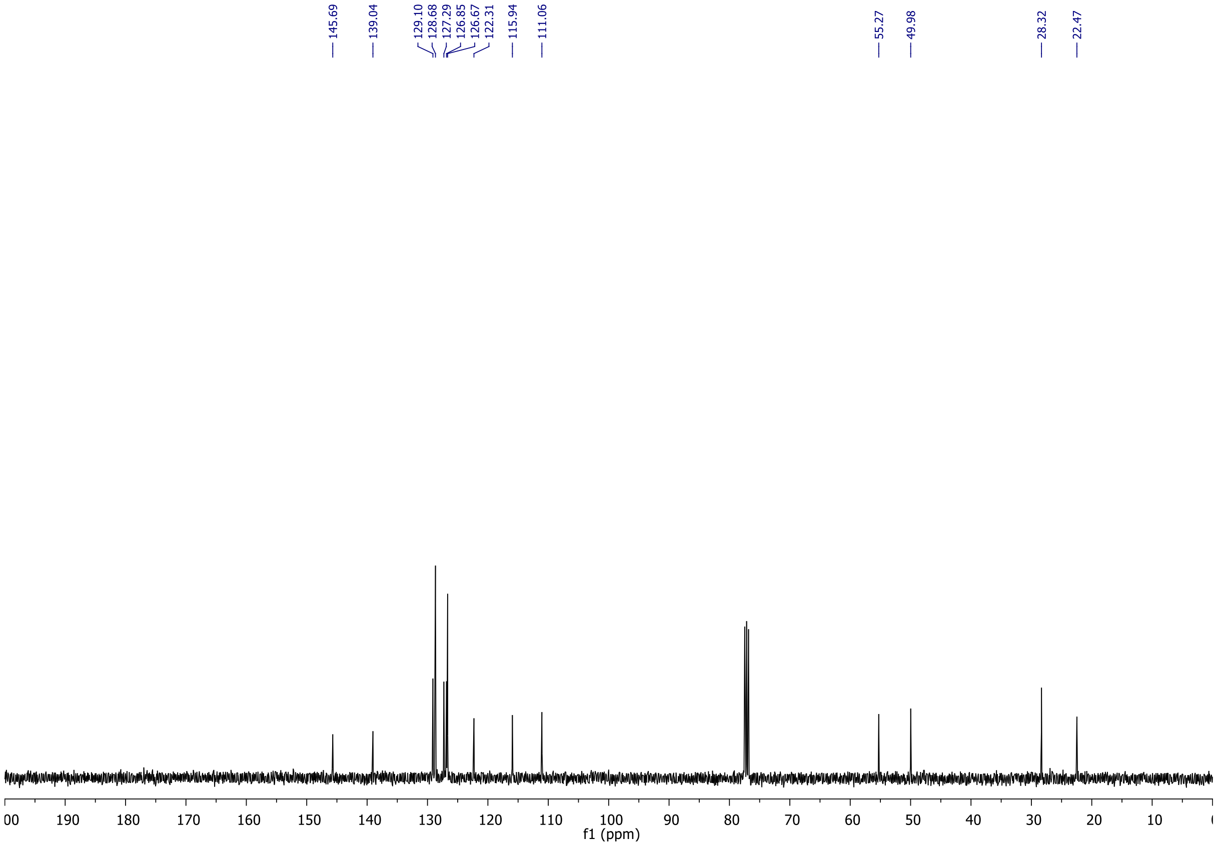

^{1}H NMR (400 MHz) and $^{13}C\{^{1}H\}$ NMR (100 MHz) spectra of **13a** ($CDCl_3$)

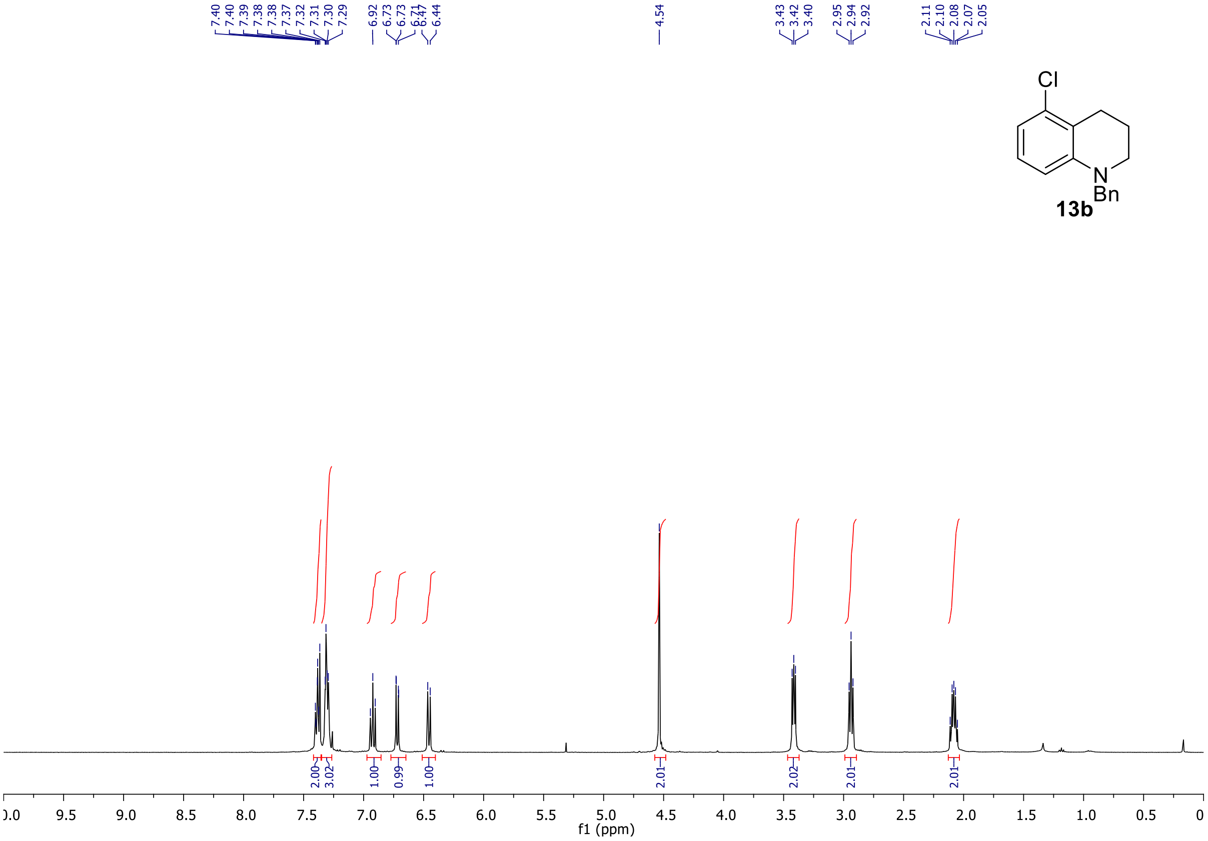

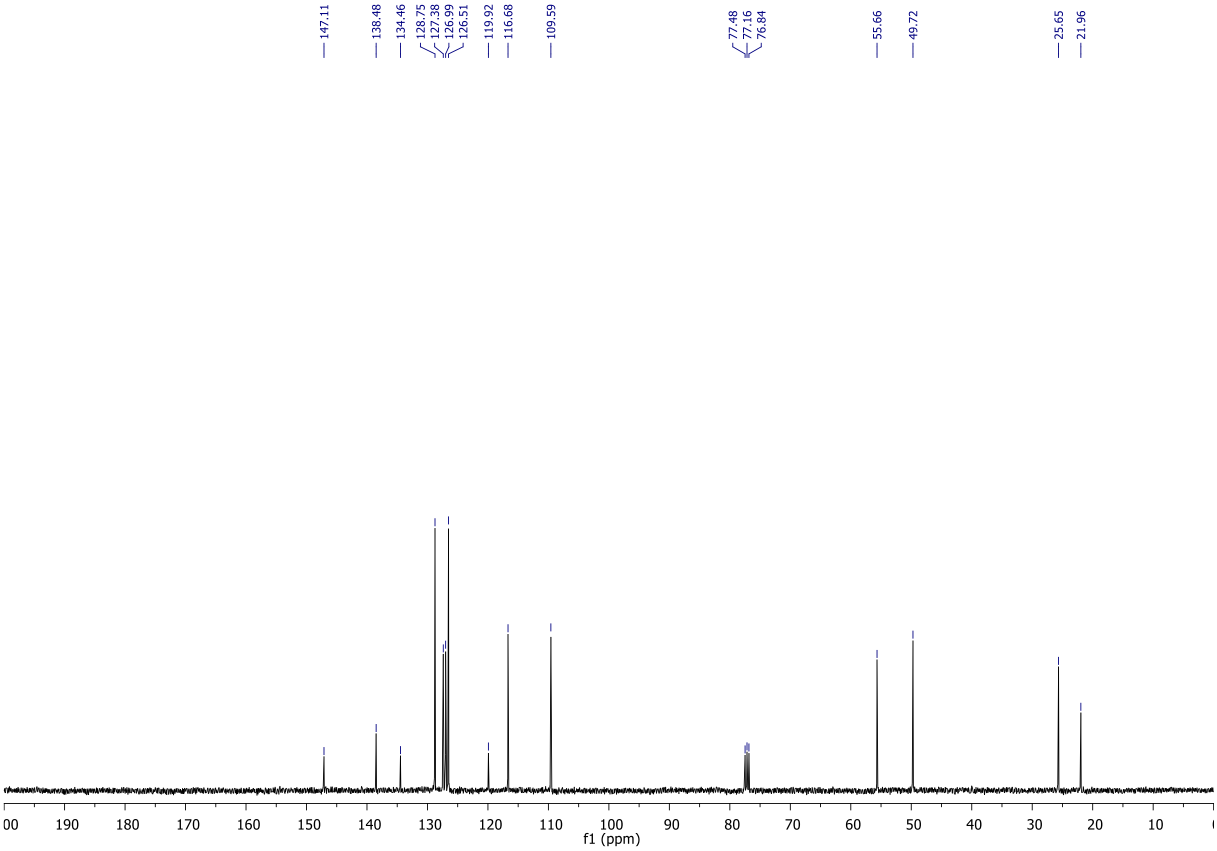

^{1}H NMR (400 MHz) and $^{13}C\{^{1}H\}$ NMR (100 MHz) spectra of **13b** ($CDCl_3$)

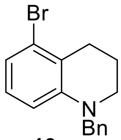

13c

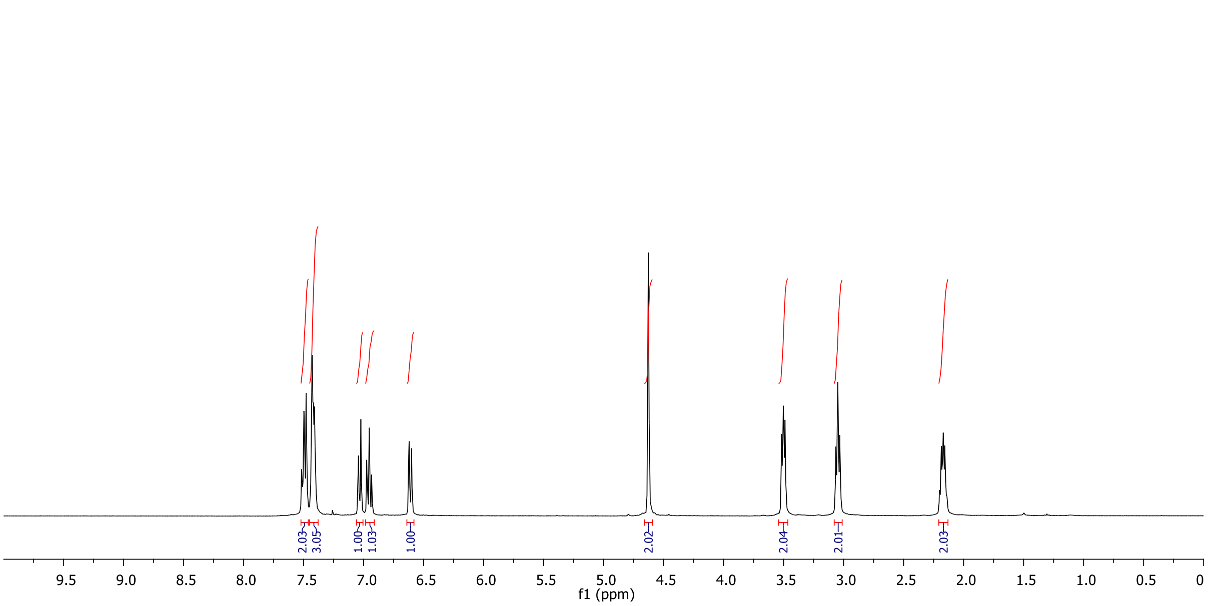

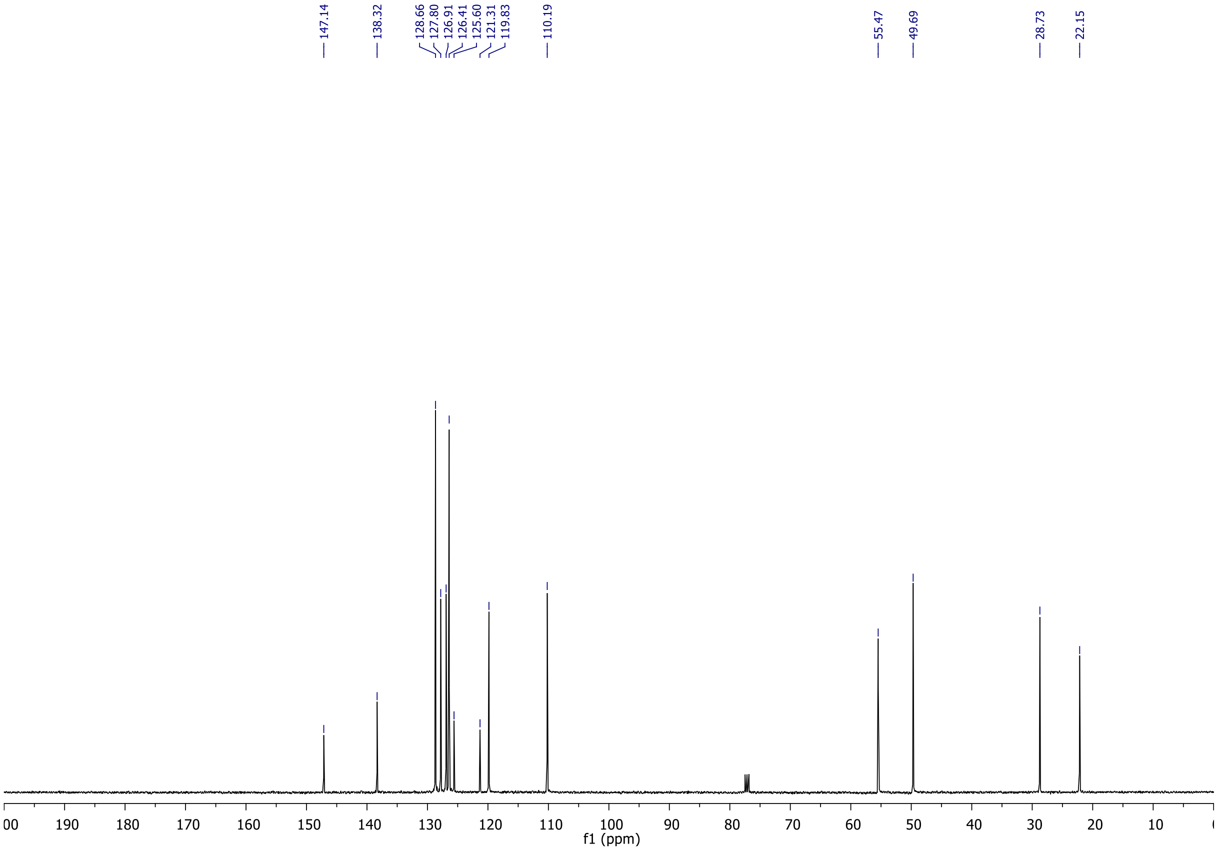

^{1}H NMR (400 MHz) and $^{13}C\{^{1}H\}$ NMR (100 MHz) spectra of **13c** ($CDCl_3$)

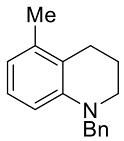

13d

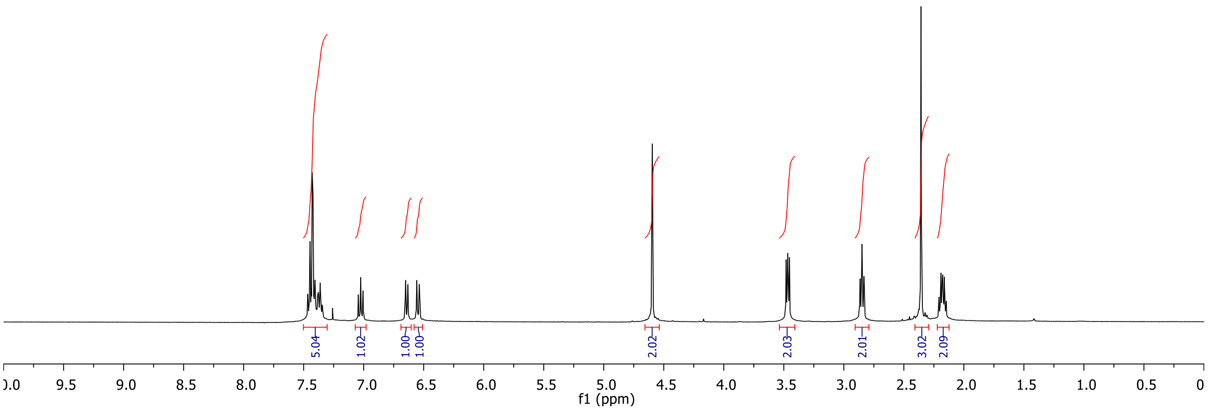

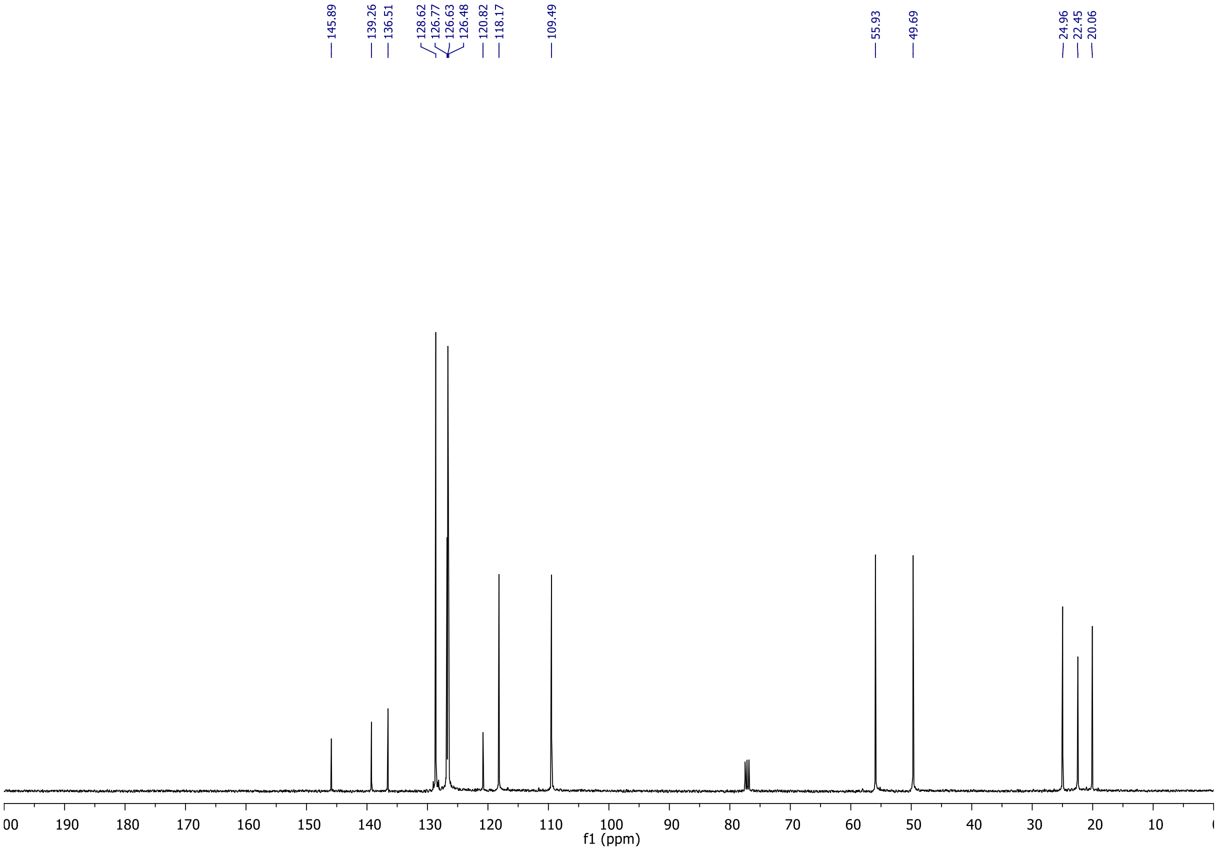

^{1}H NMR (400 MHz) and ^{13}C{^{1}H} NMR (100 MHz) spectra of **13d** ($CDCl_3$)

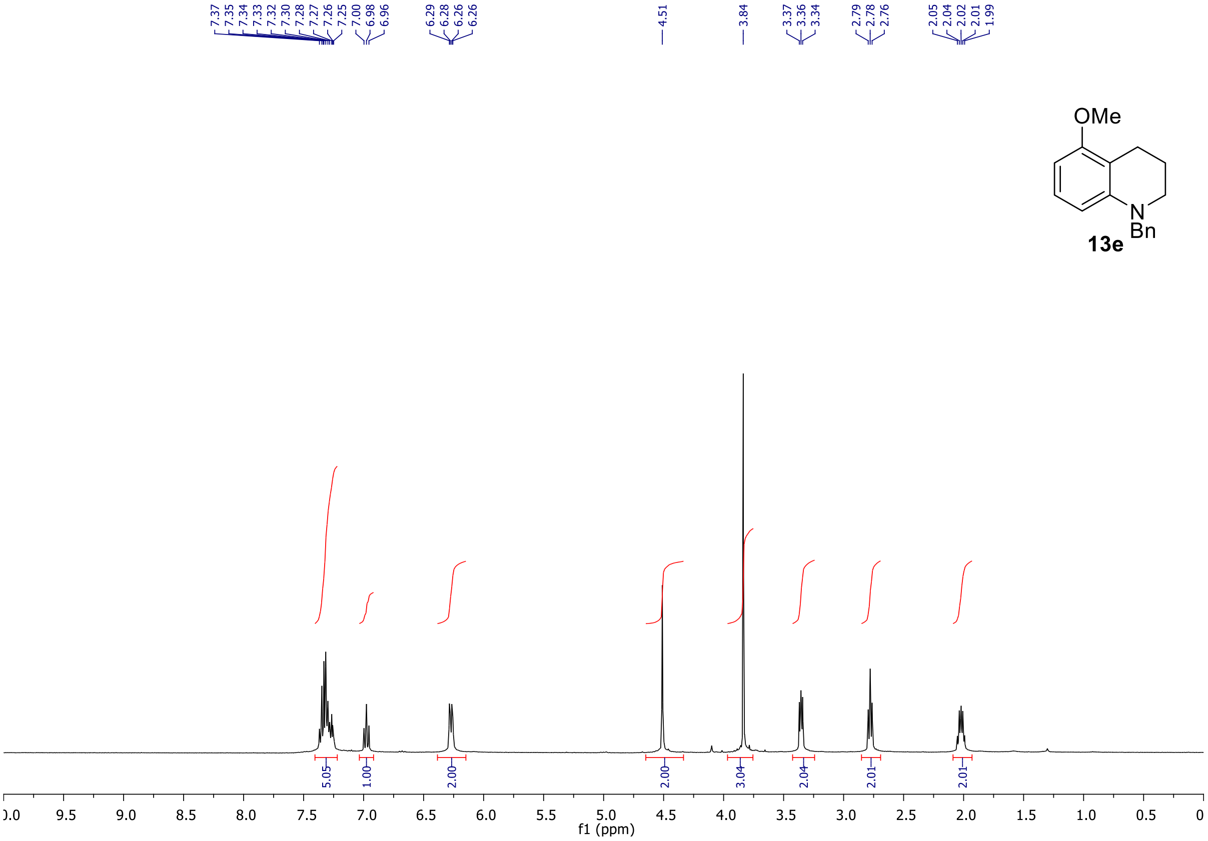

13e

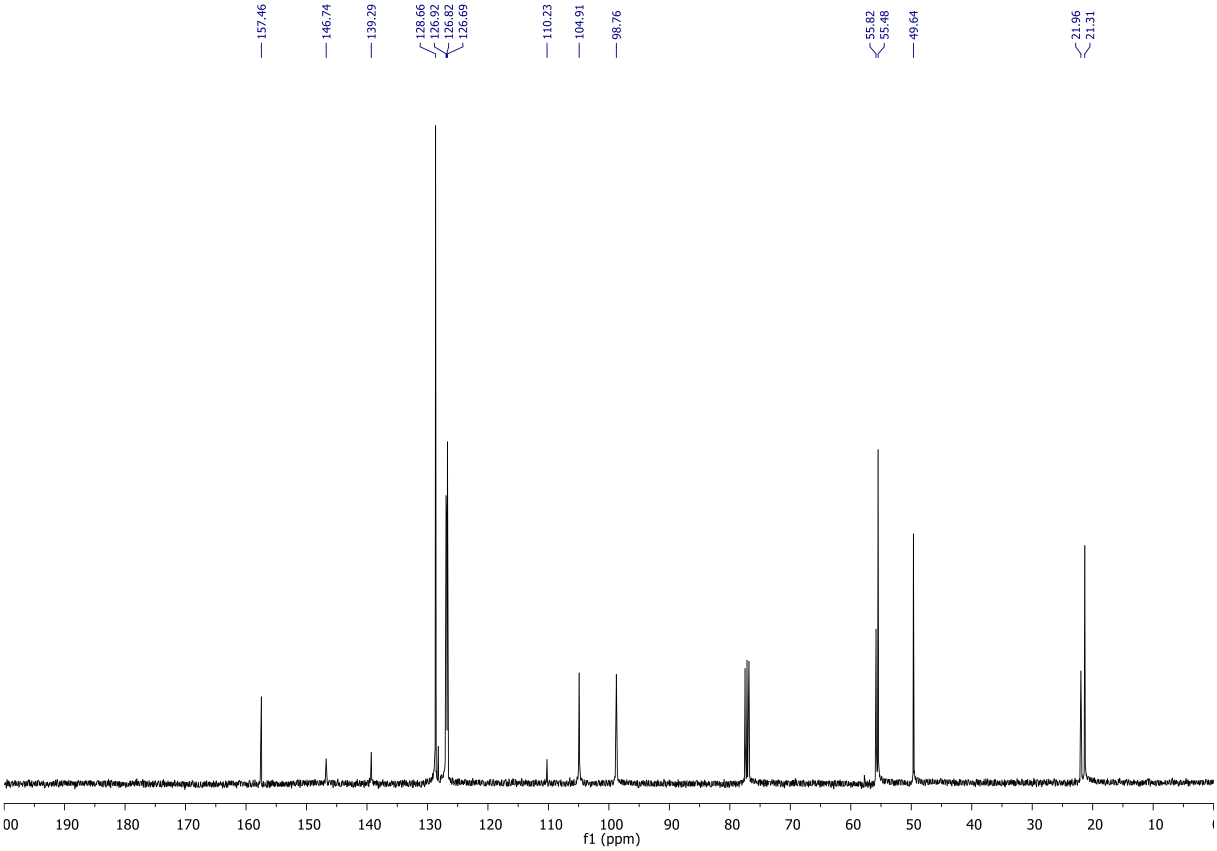

^{1}H NMR (400 MHz) and ^{13}C{^{1}H} NMR (100 MHz) spectra of **13e** ($CDCl_3$)

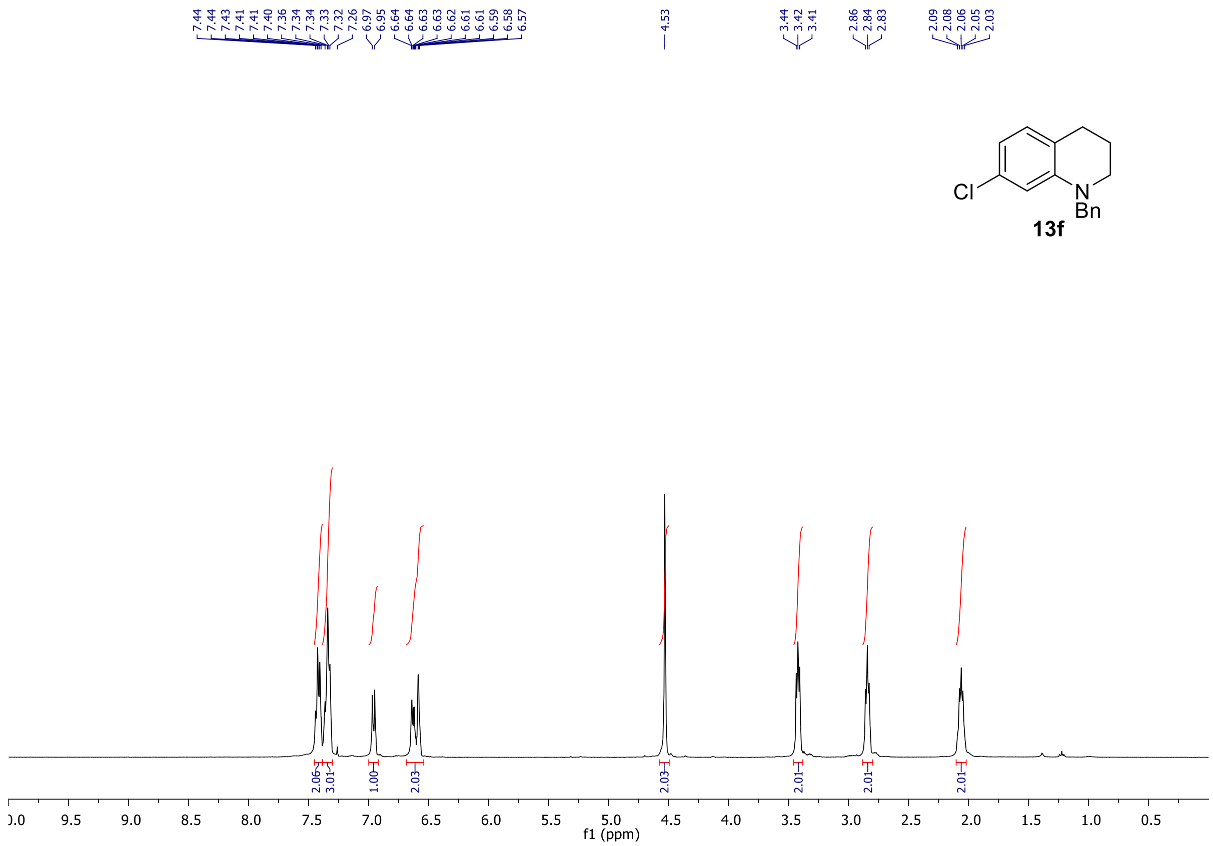

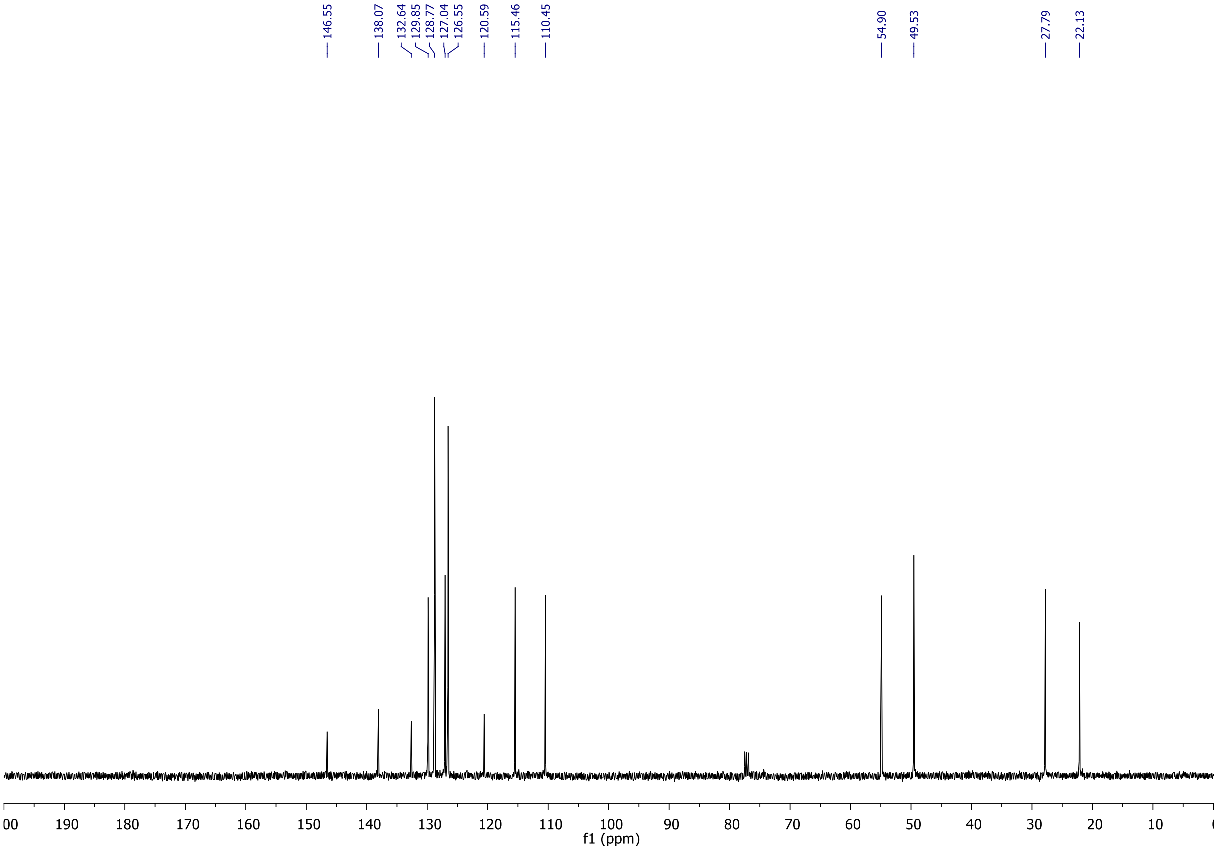

^{1}H NMR (400 MHz) and $^{13}C\{^{1}H\}$ NMR (100 MHz) spectra of **13f** ($CDCl_3$)

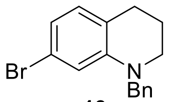

13g

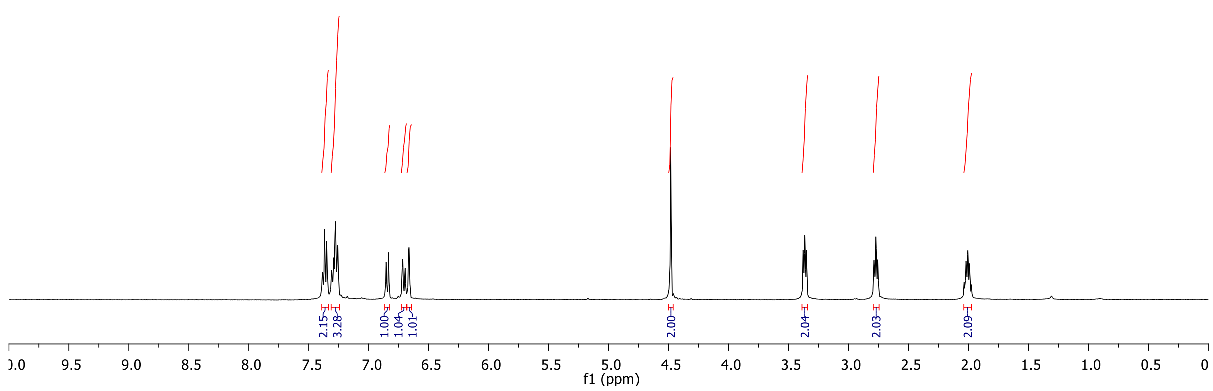

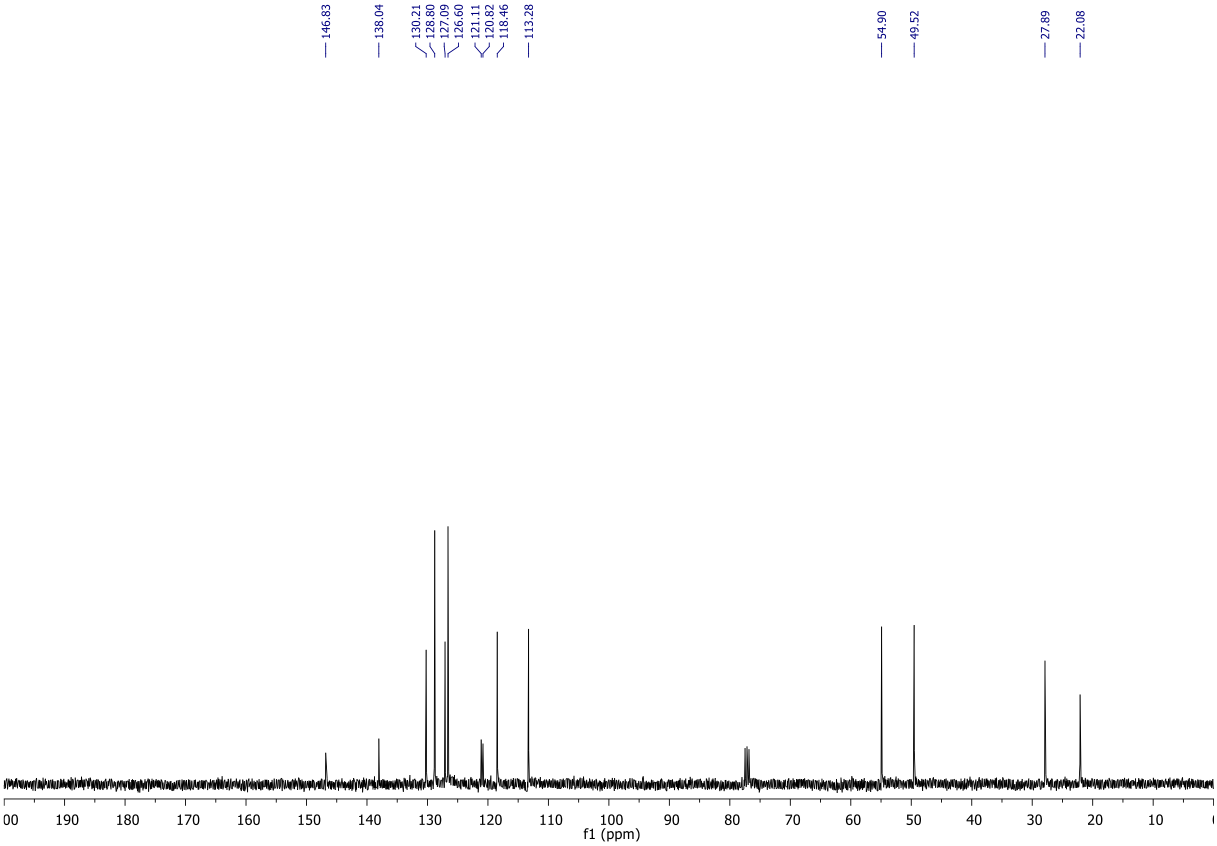

^{1}H NMR (400 MHz) and ^{13}C{^{1}H} NMR (100 MHz) spectra of **13g** ($CDCl_3$)

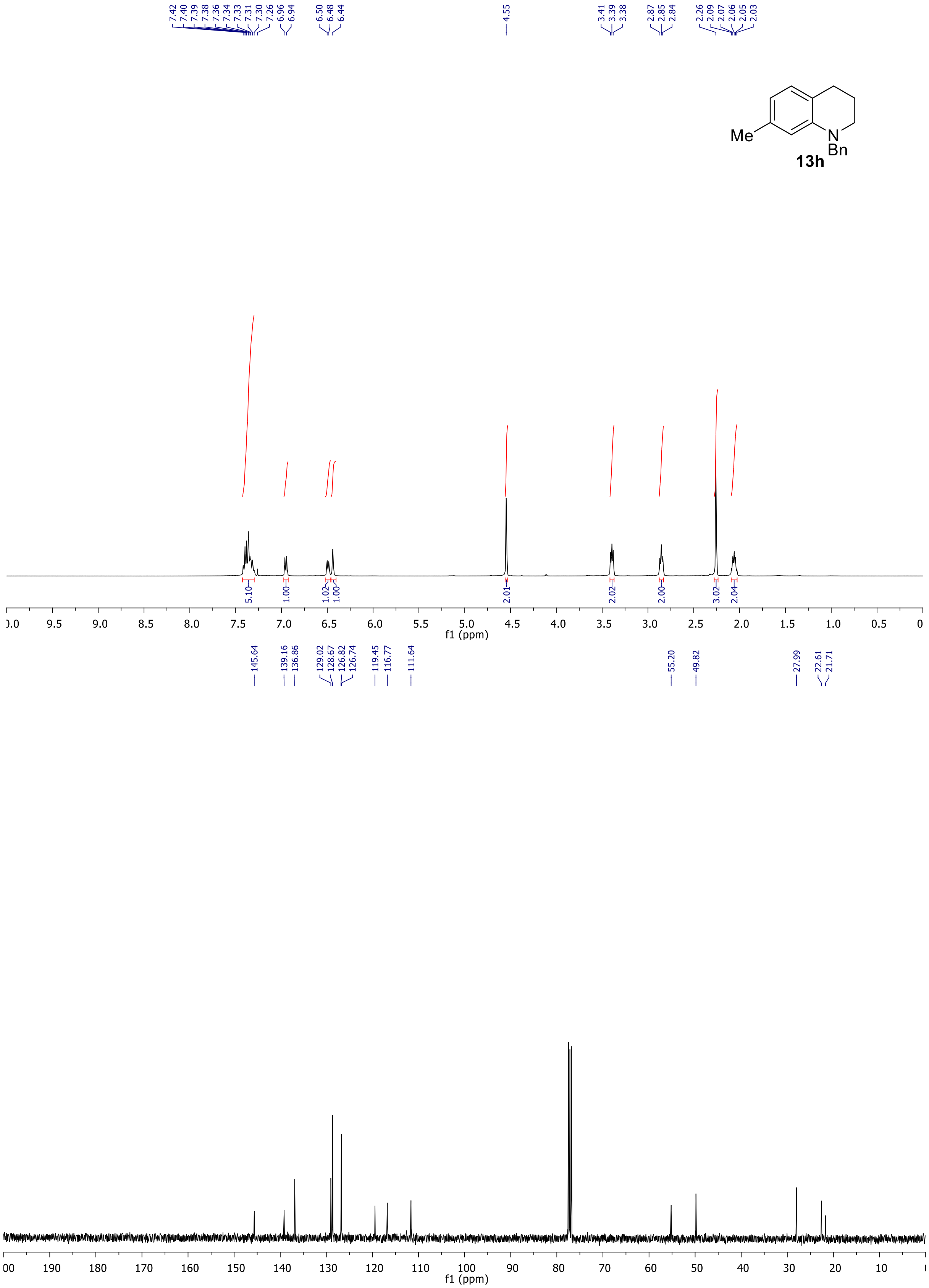

^{1}H NMR (400 MHz) and ^{13}C{^{1}H} NMR (100 MHz) spectra of **13h** ($CDCl_3$)

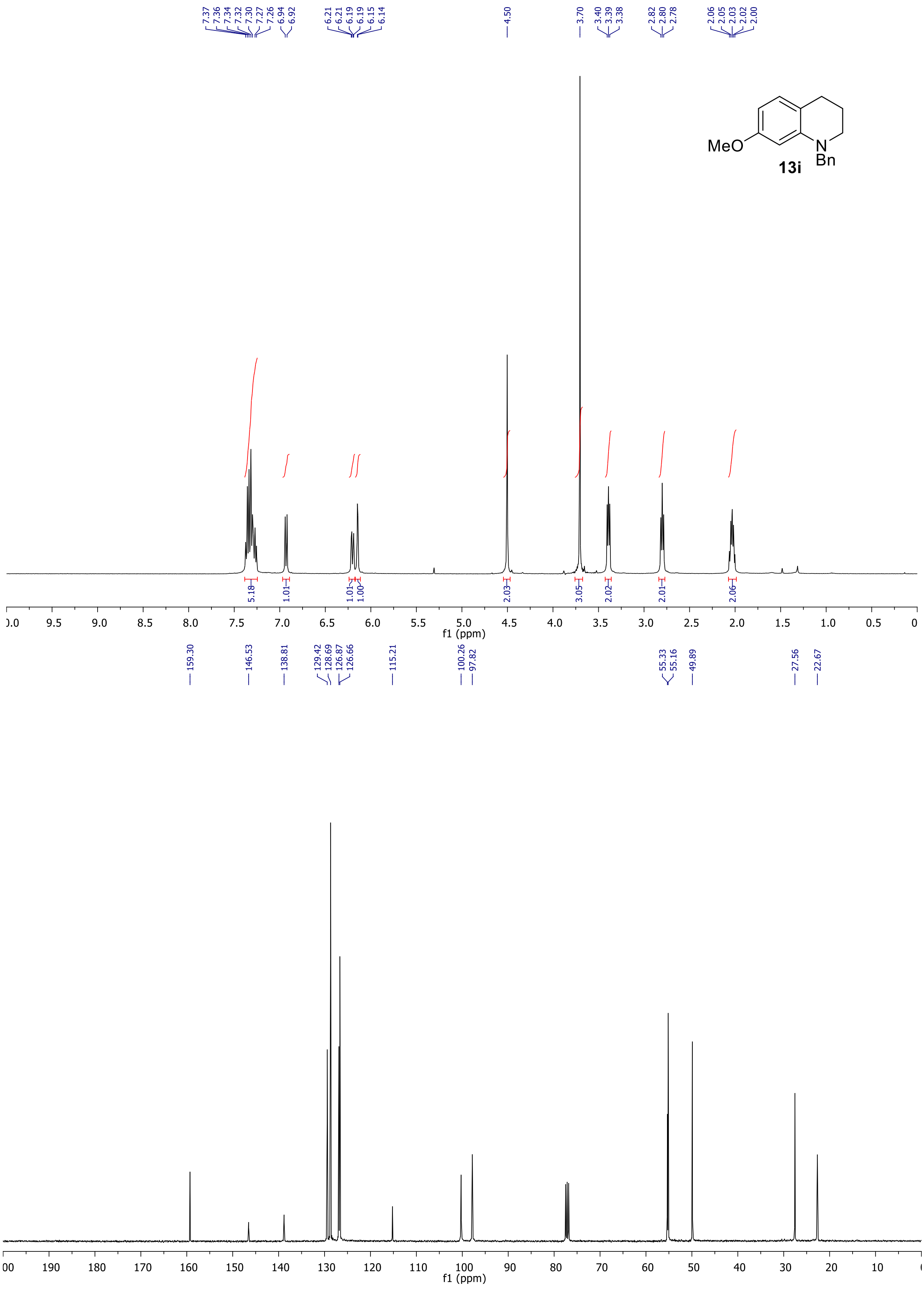

^{1}H NMR (400 MHz) and $^{13}C\{^{1}H\}$ NMR (100 MHz) spectra of **13i** ($CDCl_3$)

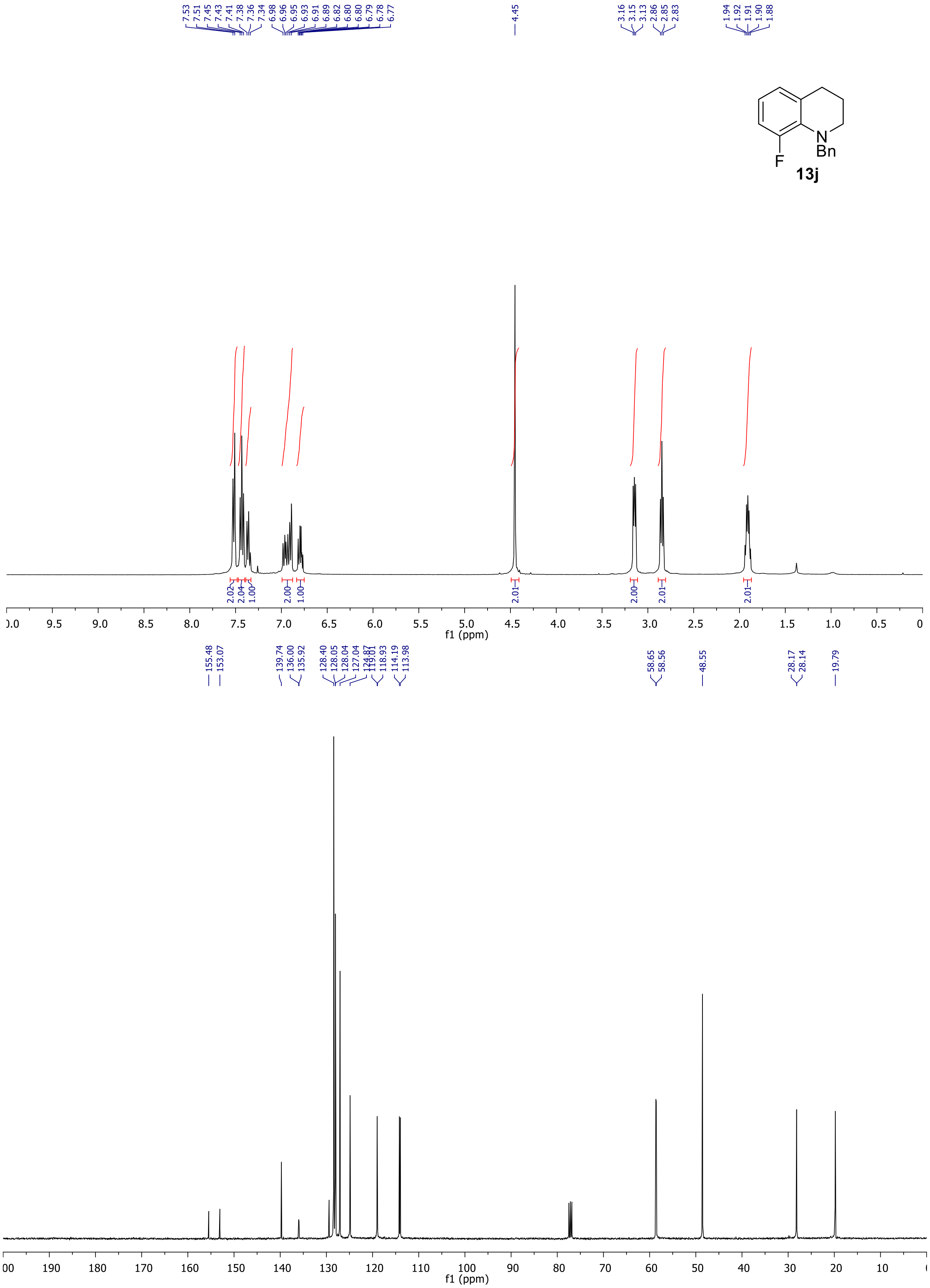

^{1}H NMR (400 MHz) and ^{13}C{^{1}H} NMR (100 MHz) spectra of **13j** (CDCl$_3$)

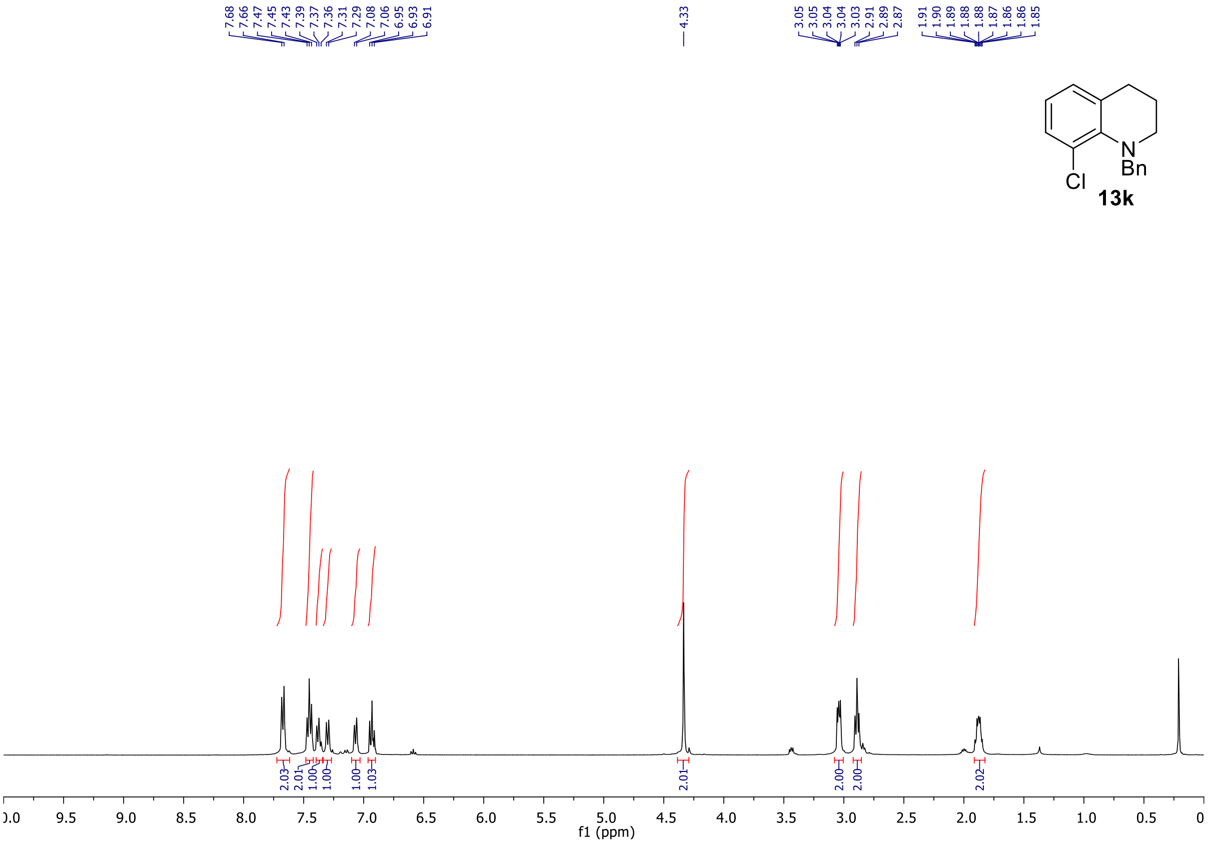

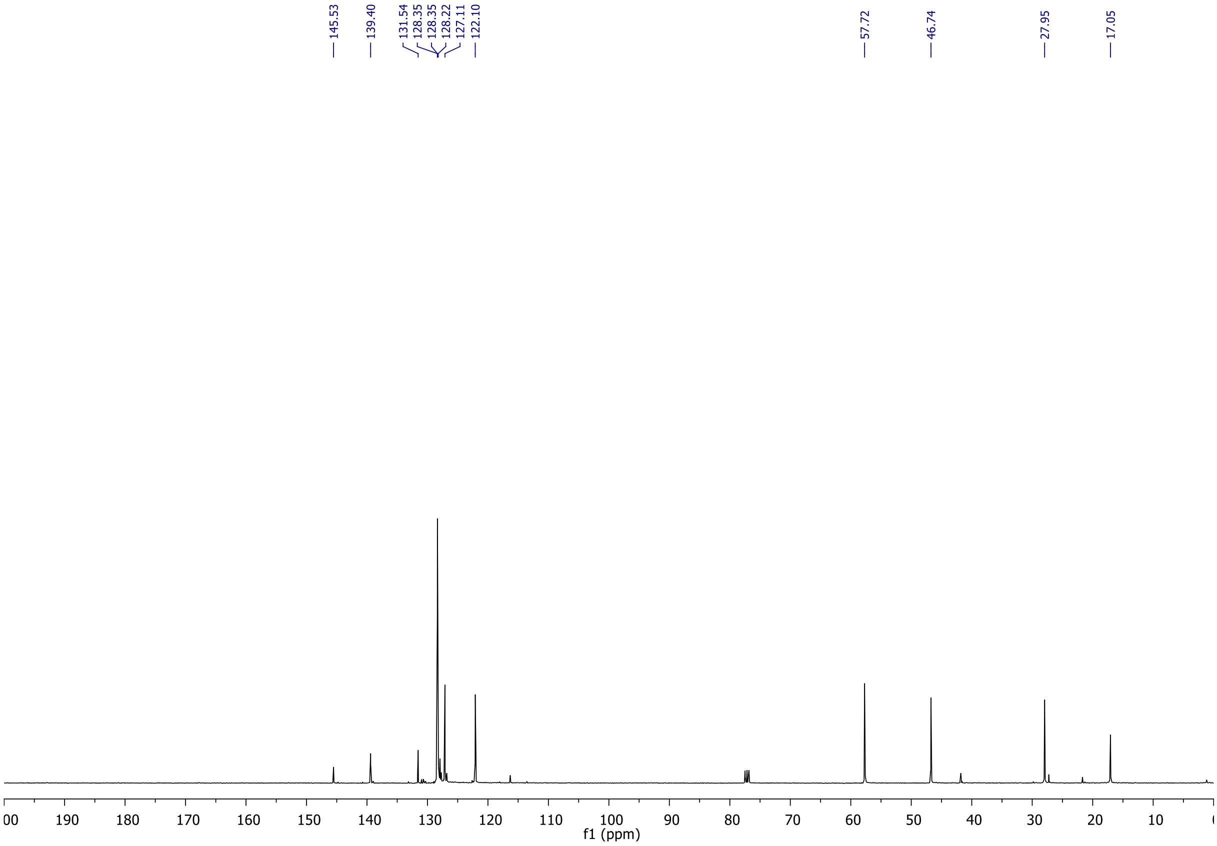

^{1}H NMR (400 MHz) and ^{13}C{^{1}H} NMR (100 MHz) spectra of **13k** ($CDCl_3$)

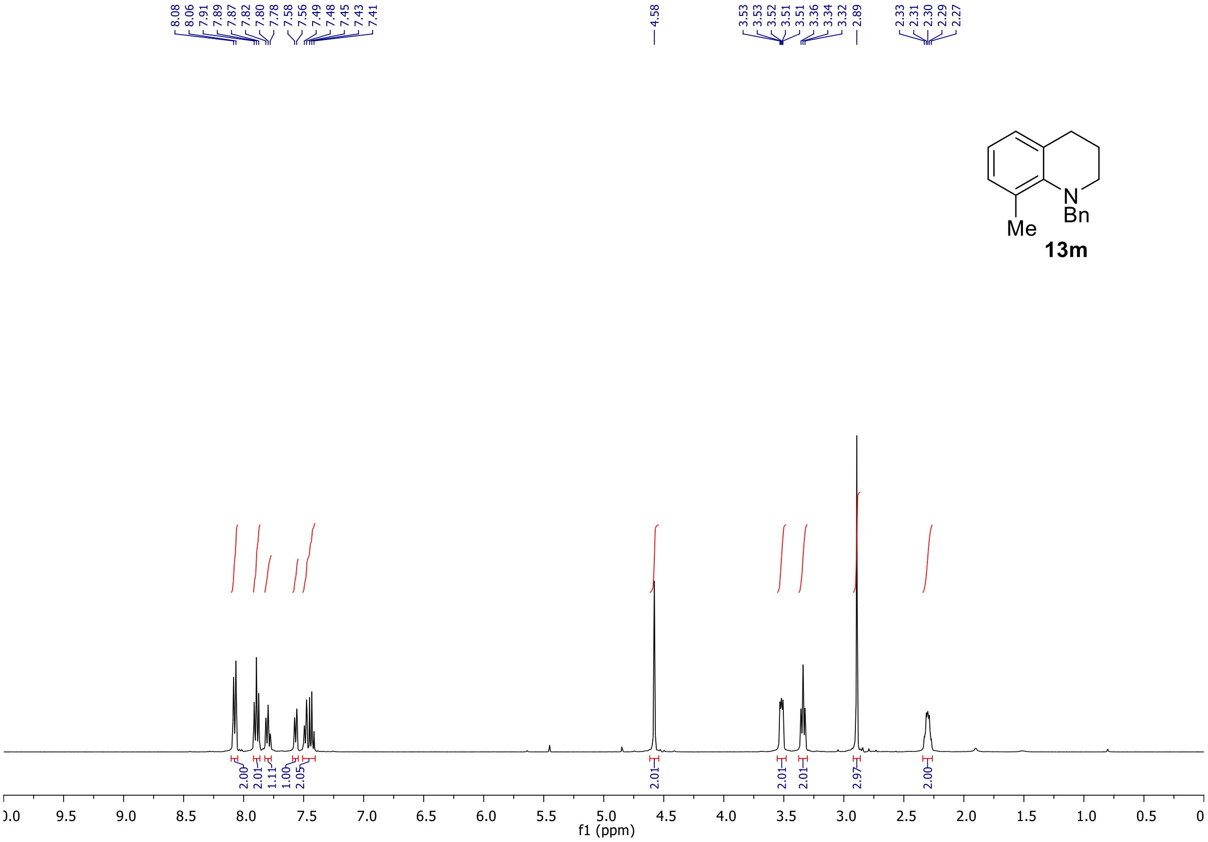

13m

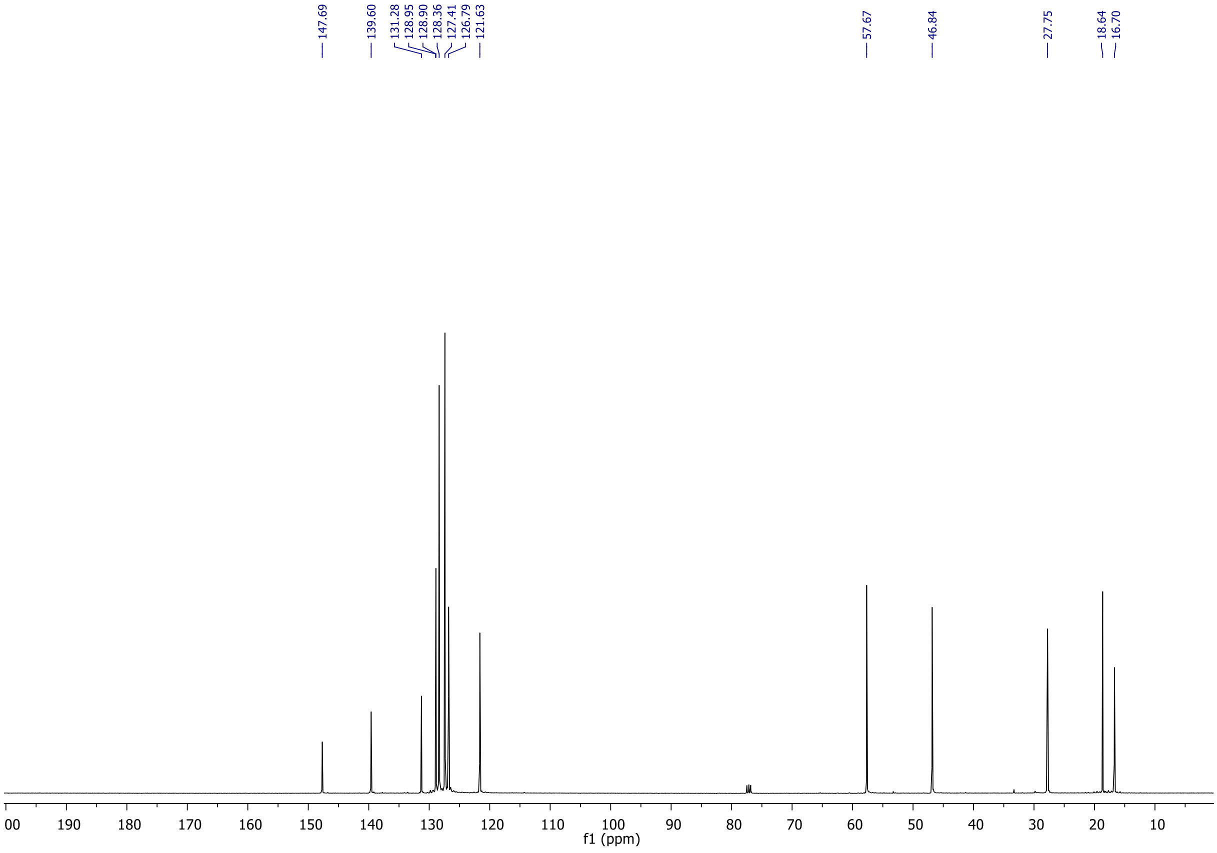

^{1}H NMR (400 MHz) and $^{13}C\{^{1}H\}$ NMR (100 MHz) spectra of **13m** ($CDCl_3$)

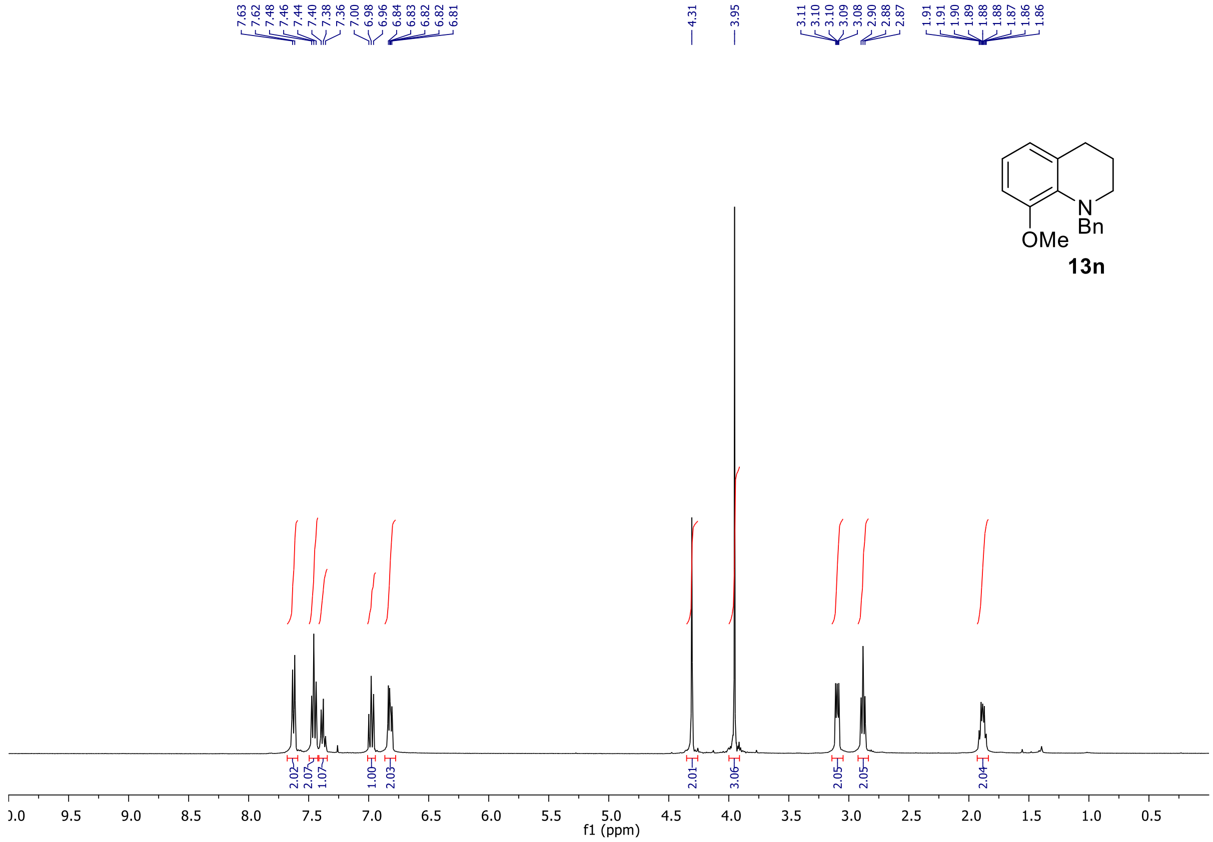

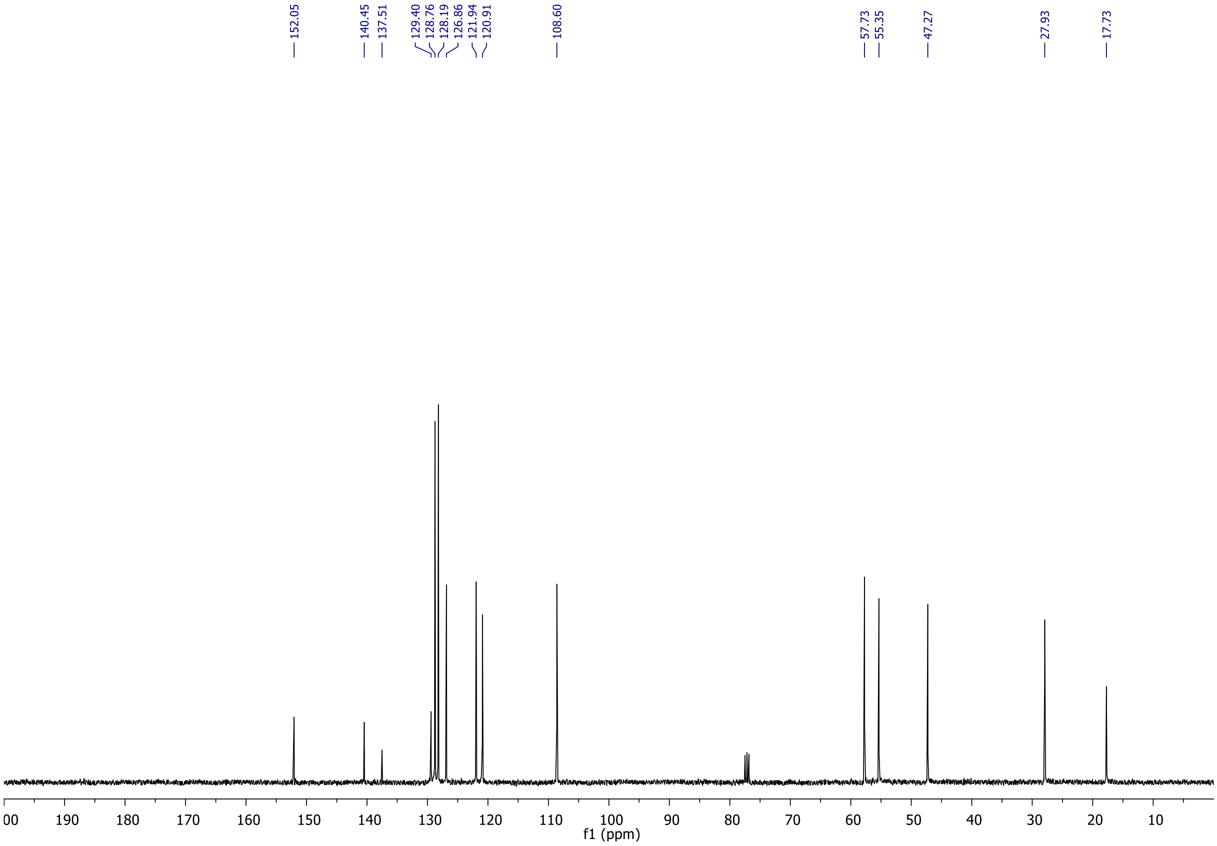

^{1}H NMR (400 MHz) and ^{13}C{^{1}H} NMR (100 MHz) spectra of **13n** ($CDCl_3$)

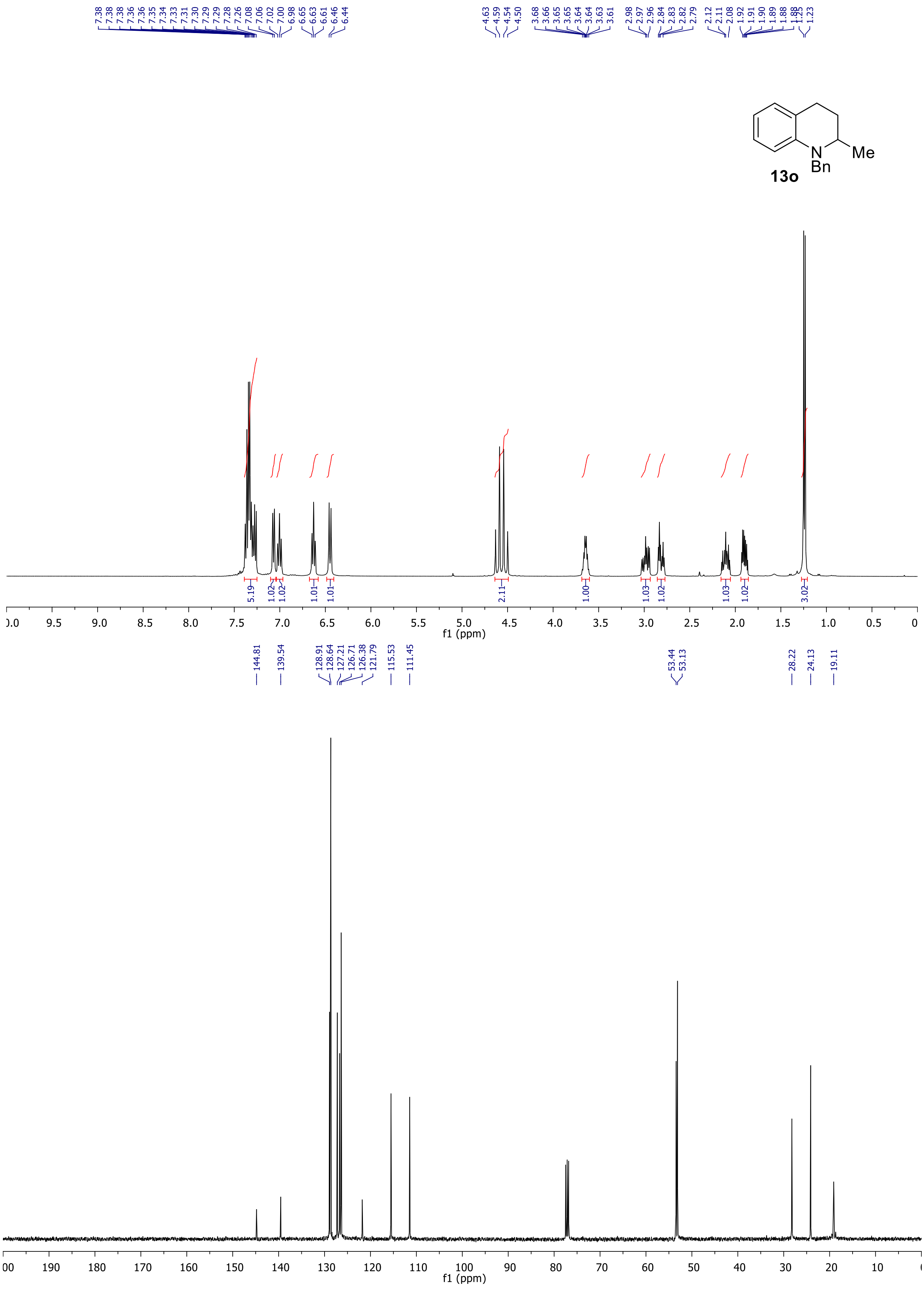

13o

^{1}H NMR (400 MHz) and $^{13}C\{^{1}H\}$ NMR (100 MHz) spectra of **13o** ($CDCl_3$)

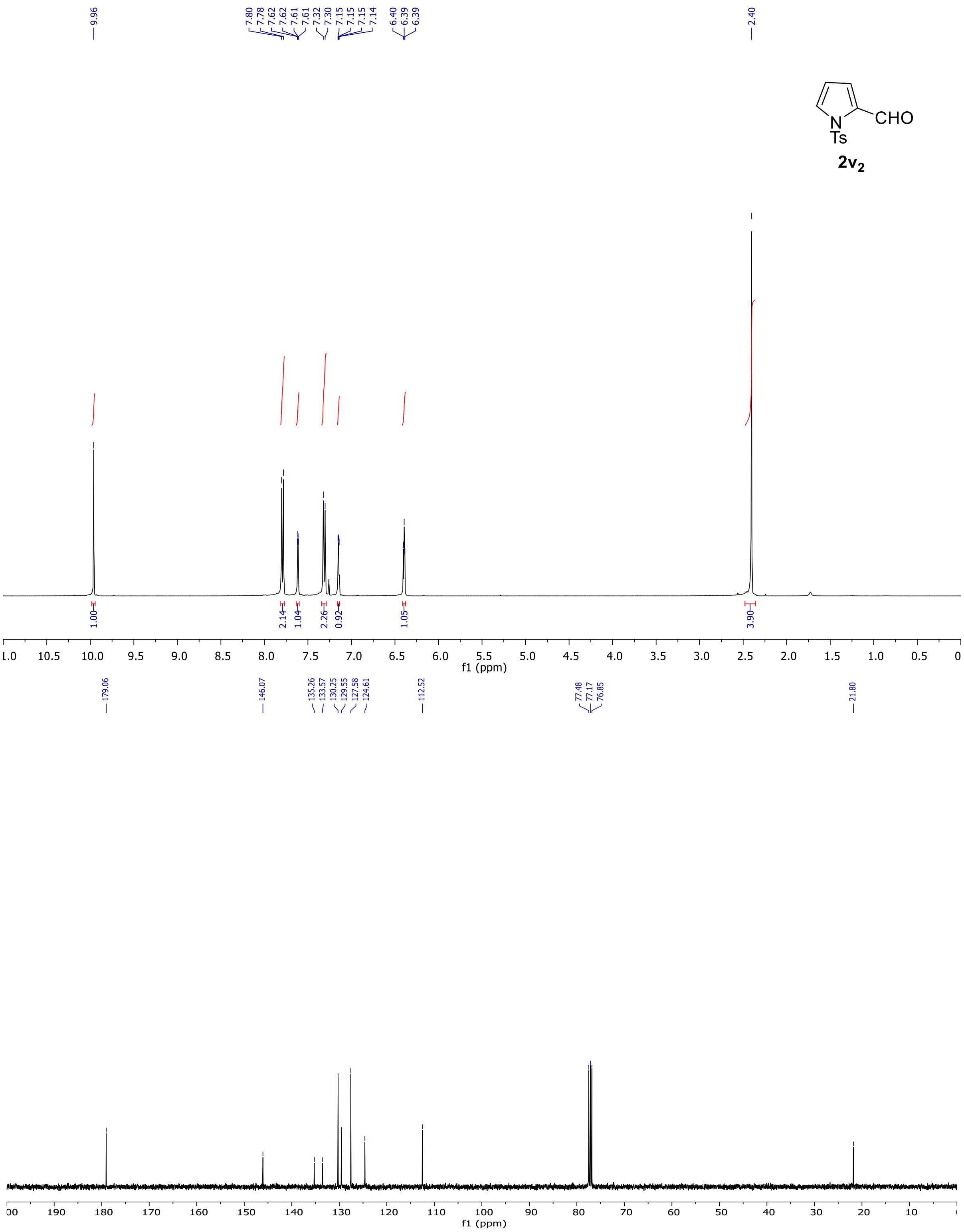

^1H NMR (400 MHz) and $^{13}C\{^1H\}$ NMR (100 MHz) spectra of **2v₂** ($CDCl_3$)

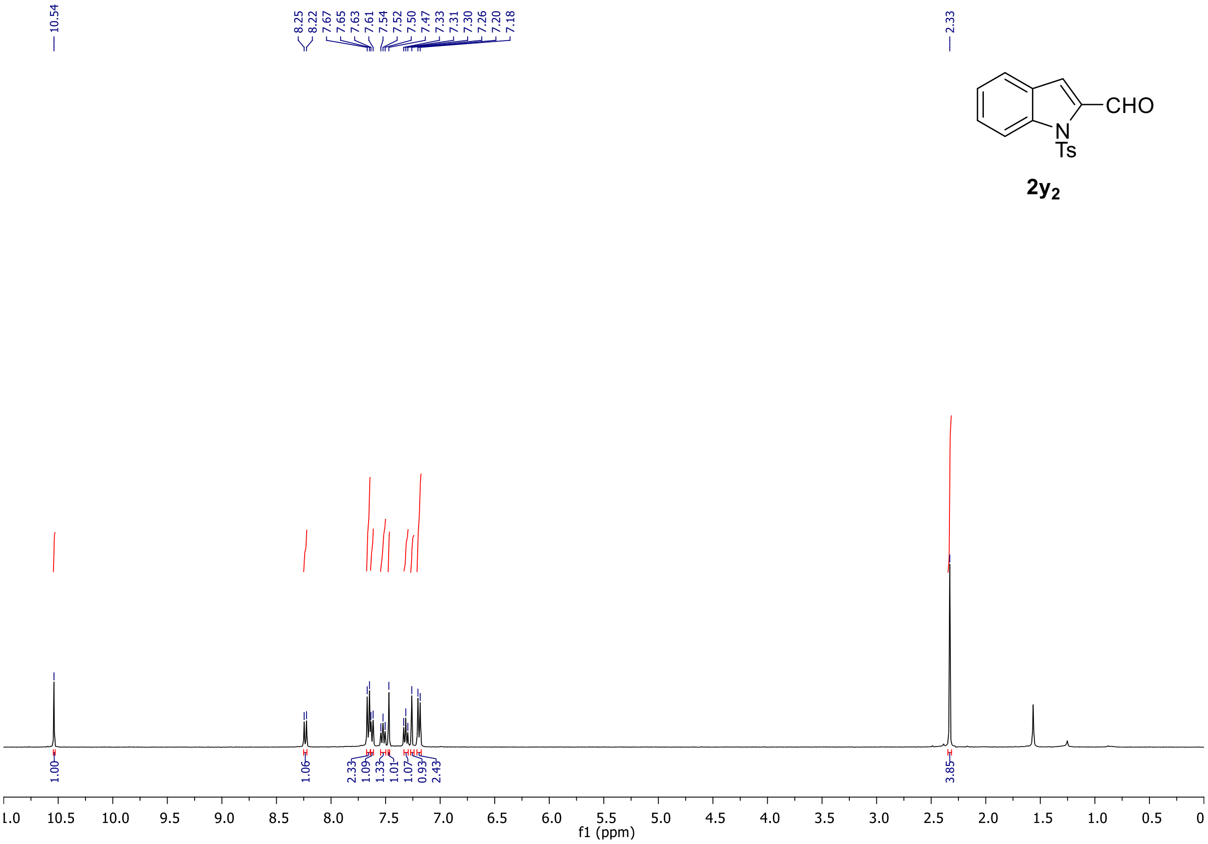

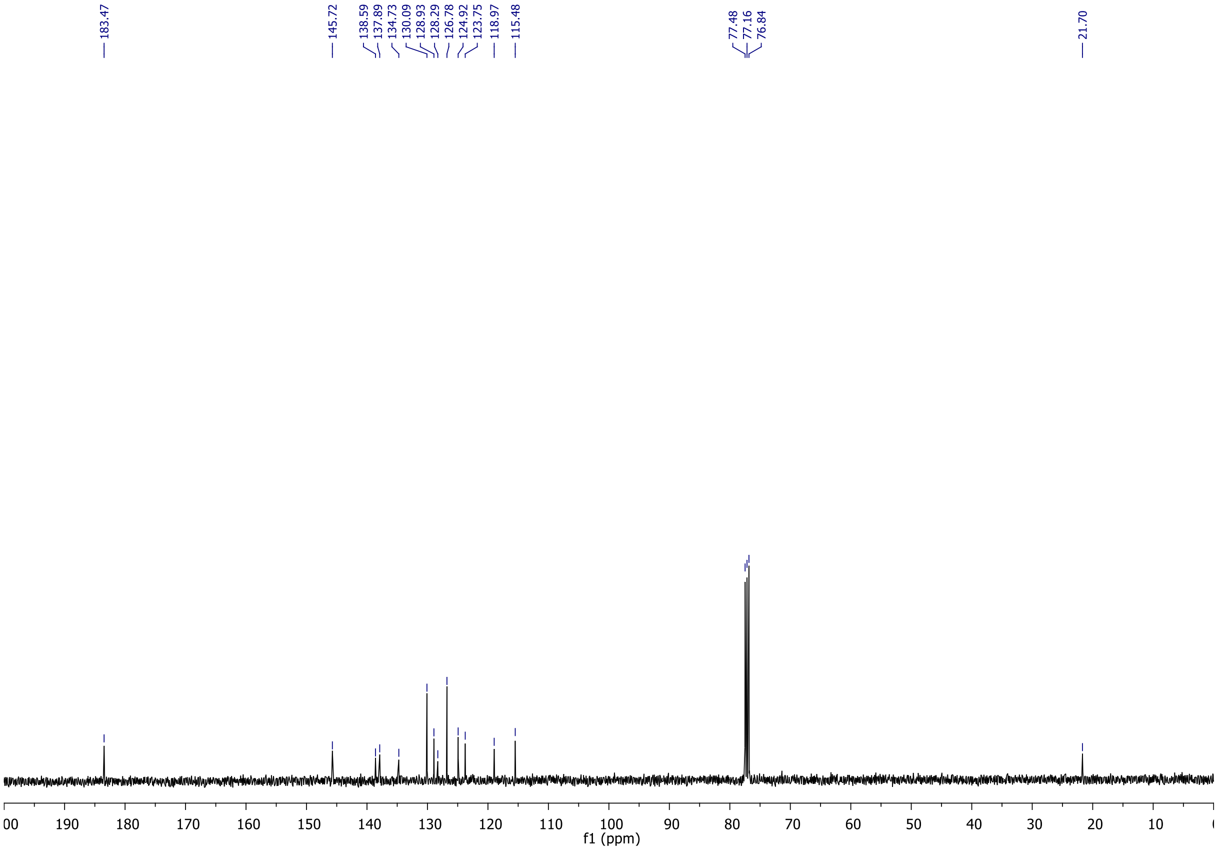

^{1}H NMR (400 MHz) and $^{13}C\{^{1}H\}$ NMR (100 MHz) spectra of **2y$_2$** ($CDCl_3$)

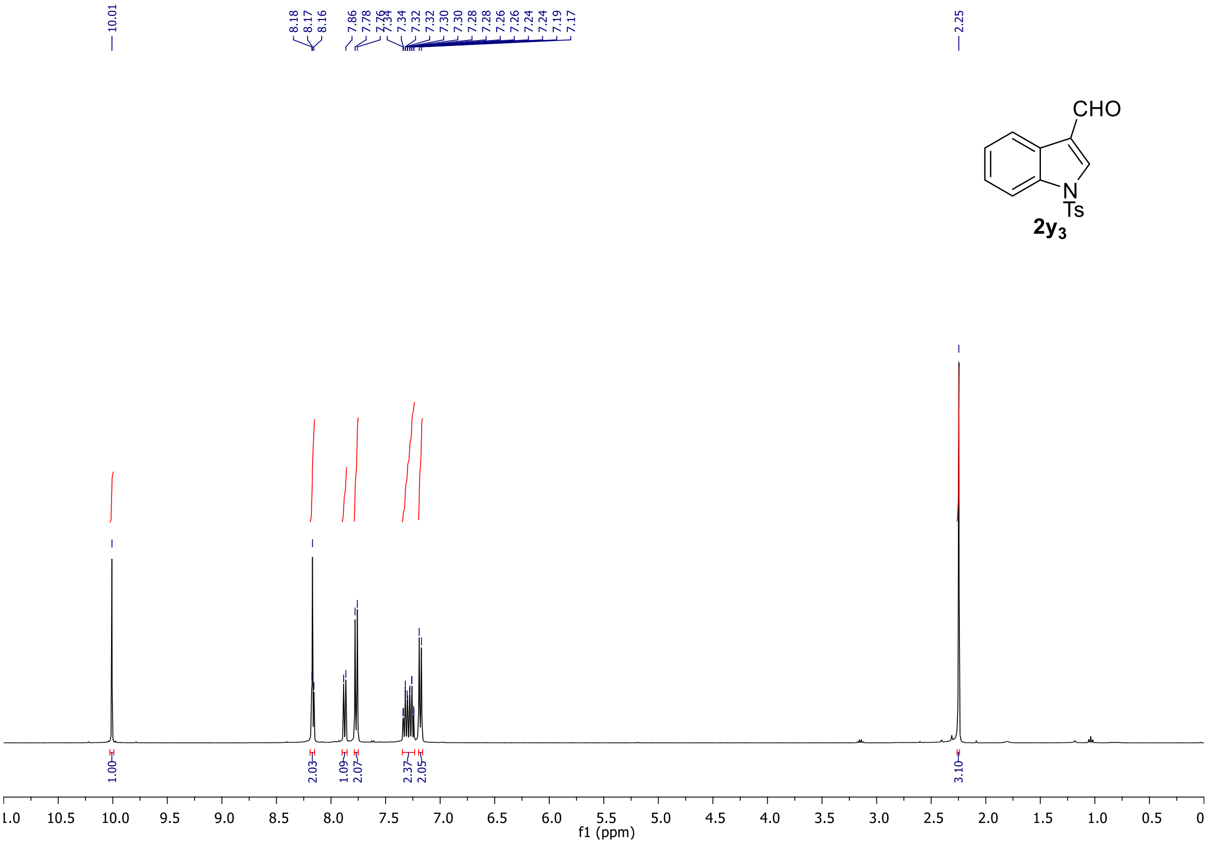

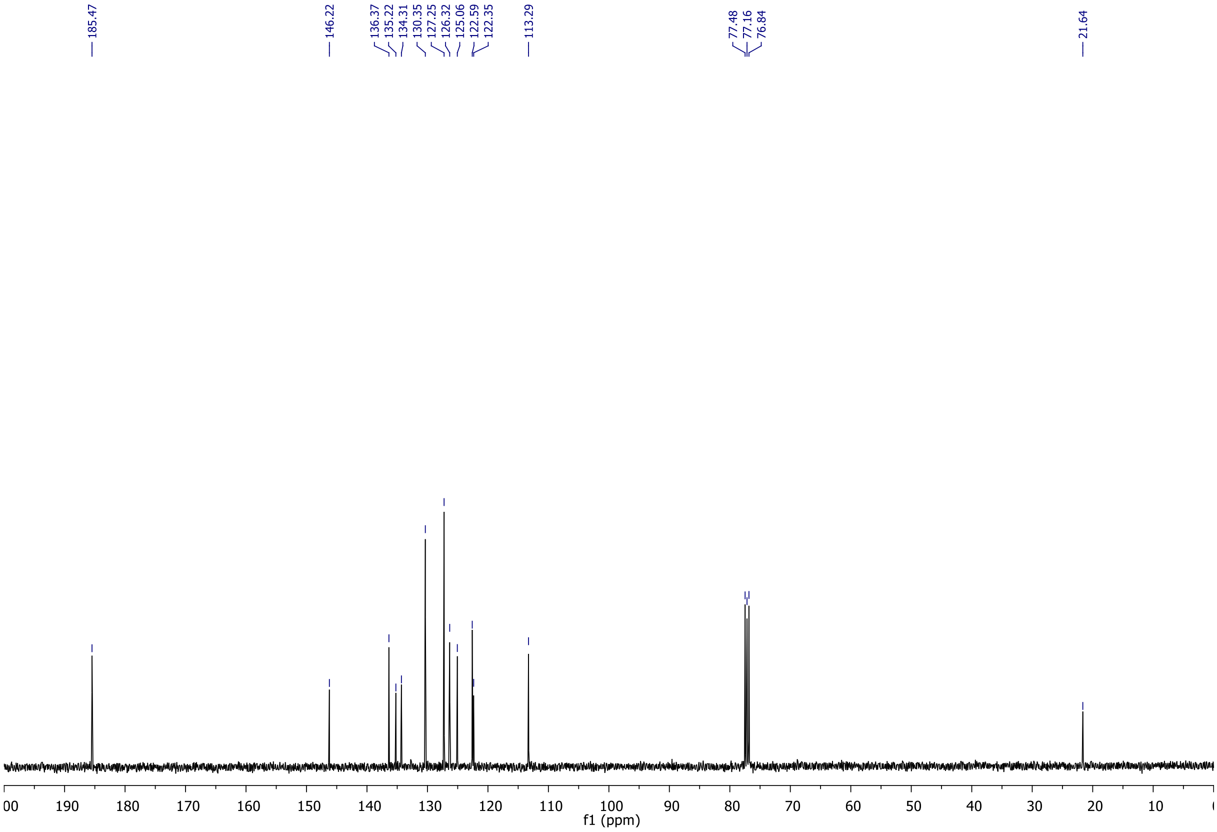

^{1}H NMR (400 MHz) and ^{13}C{^{1}H} NMR (100 MHz) spectra of **2y3** ($CDCl_3$)

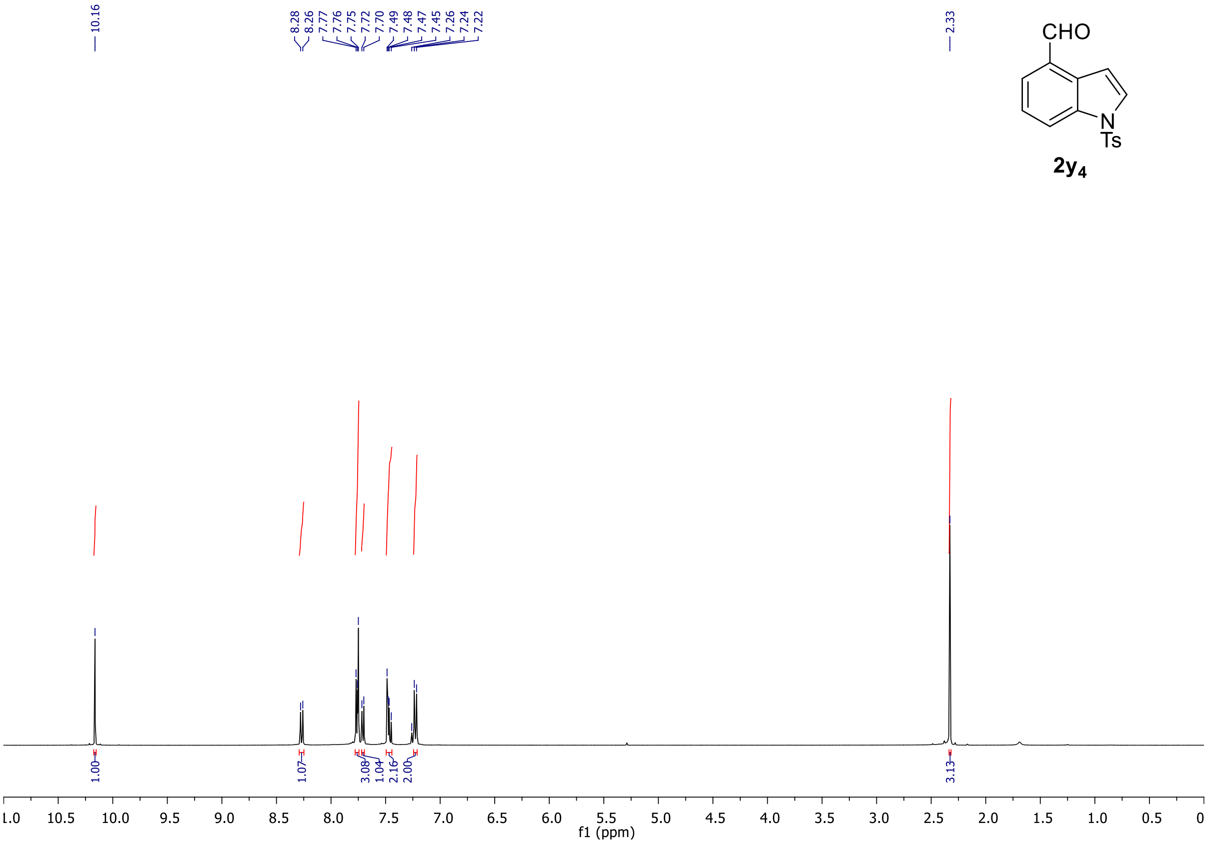

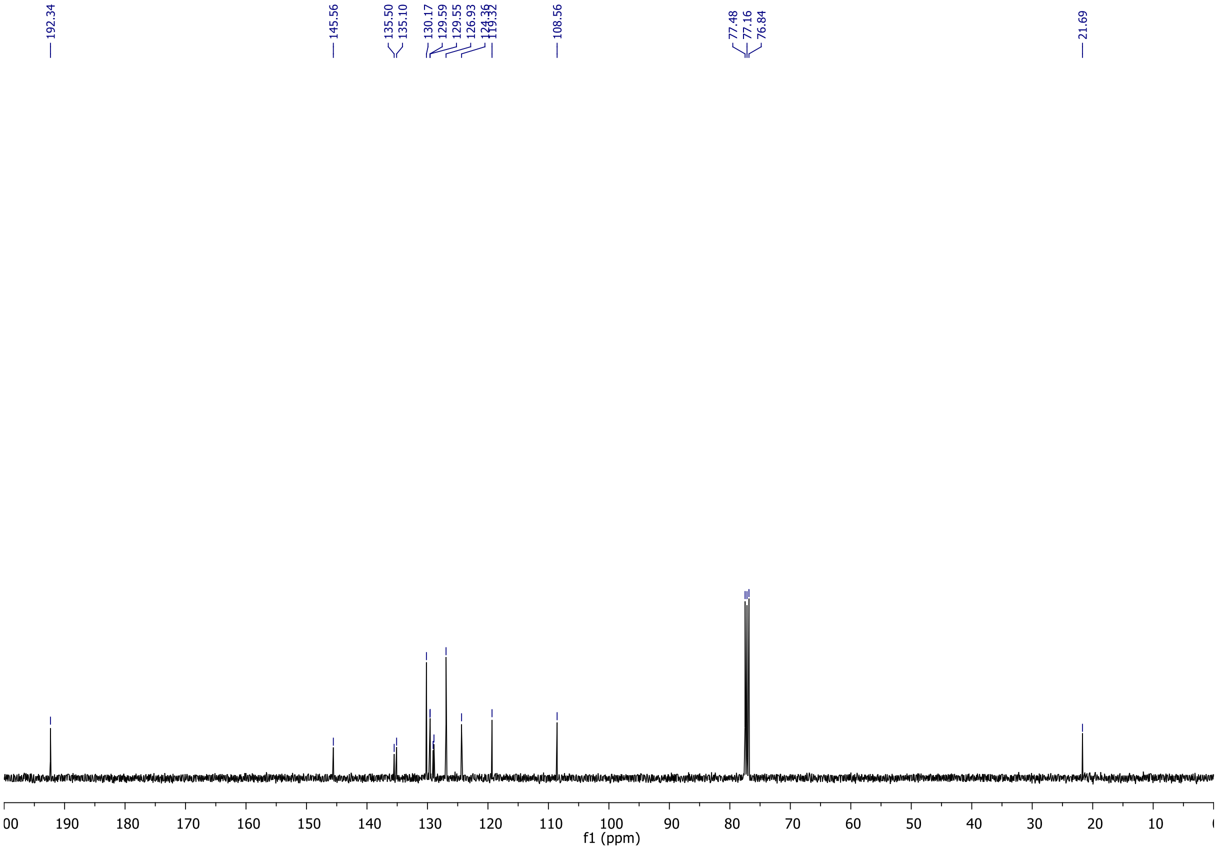

^{1}H NMR (400 MHz) and $^{13}C\{^{1}H\}$ NMR (100 MHz) spectra of **2y$_4$** ($CDCl_3$)

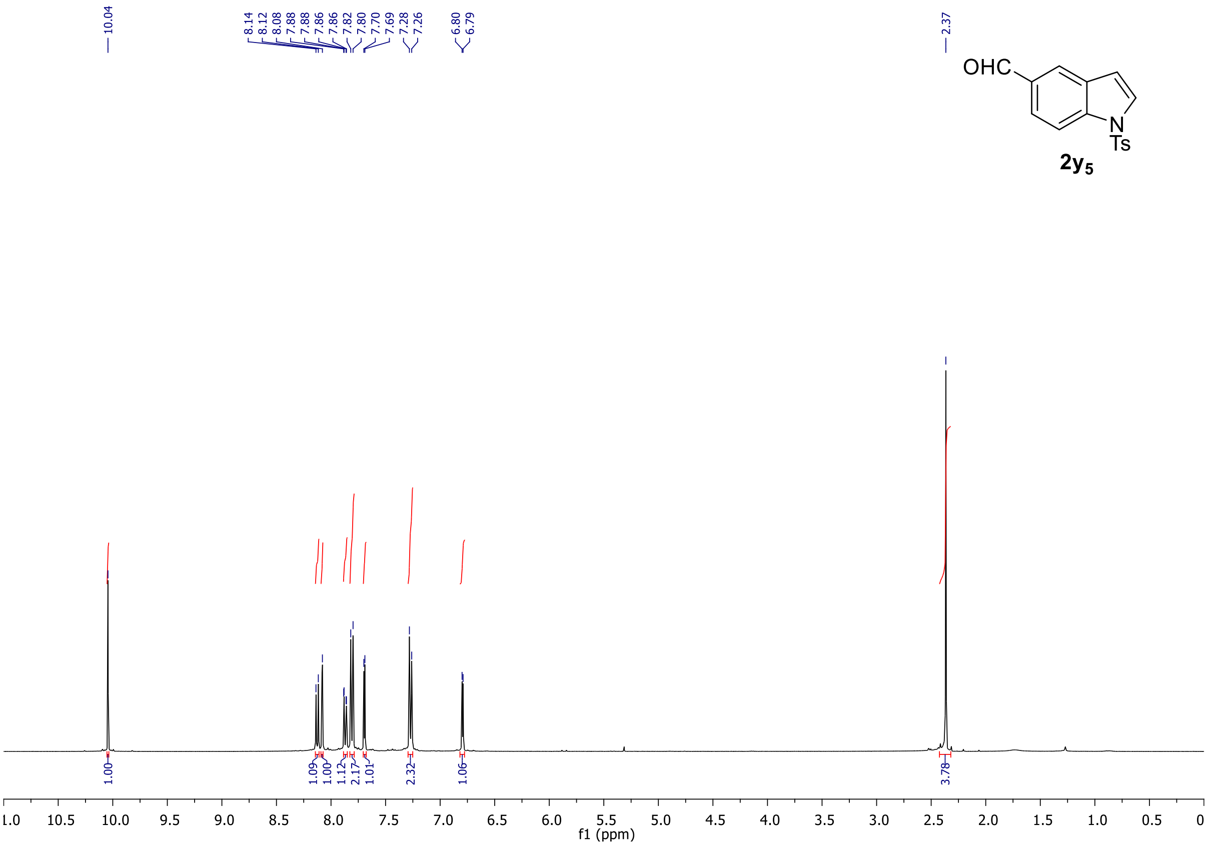

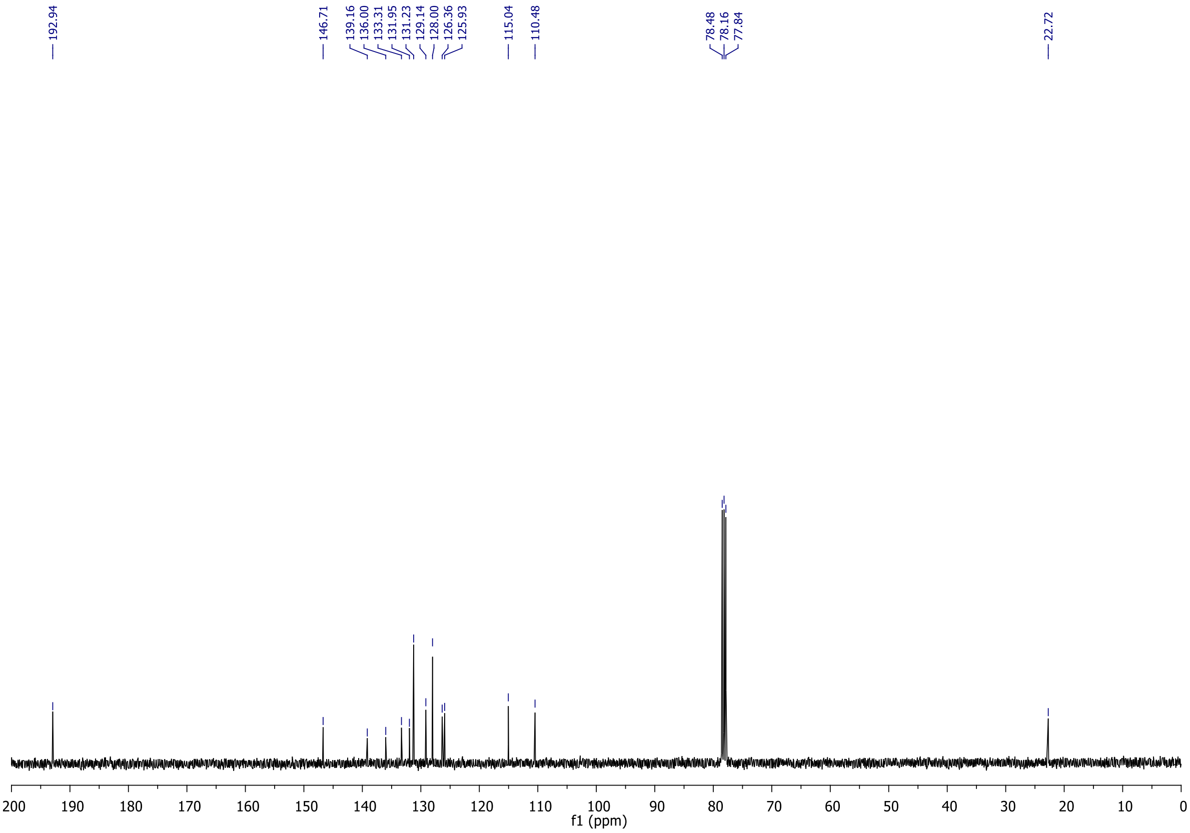

^{1}H NMR (400 MHz) and ^{13}C{^{1}H} NMR (100 MHz) spectra of **2y$_5$** ($CDCl_3$)

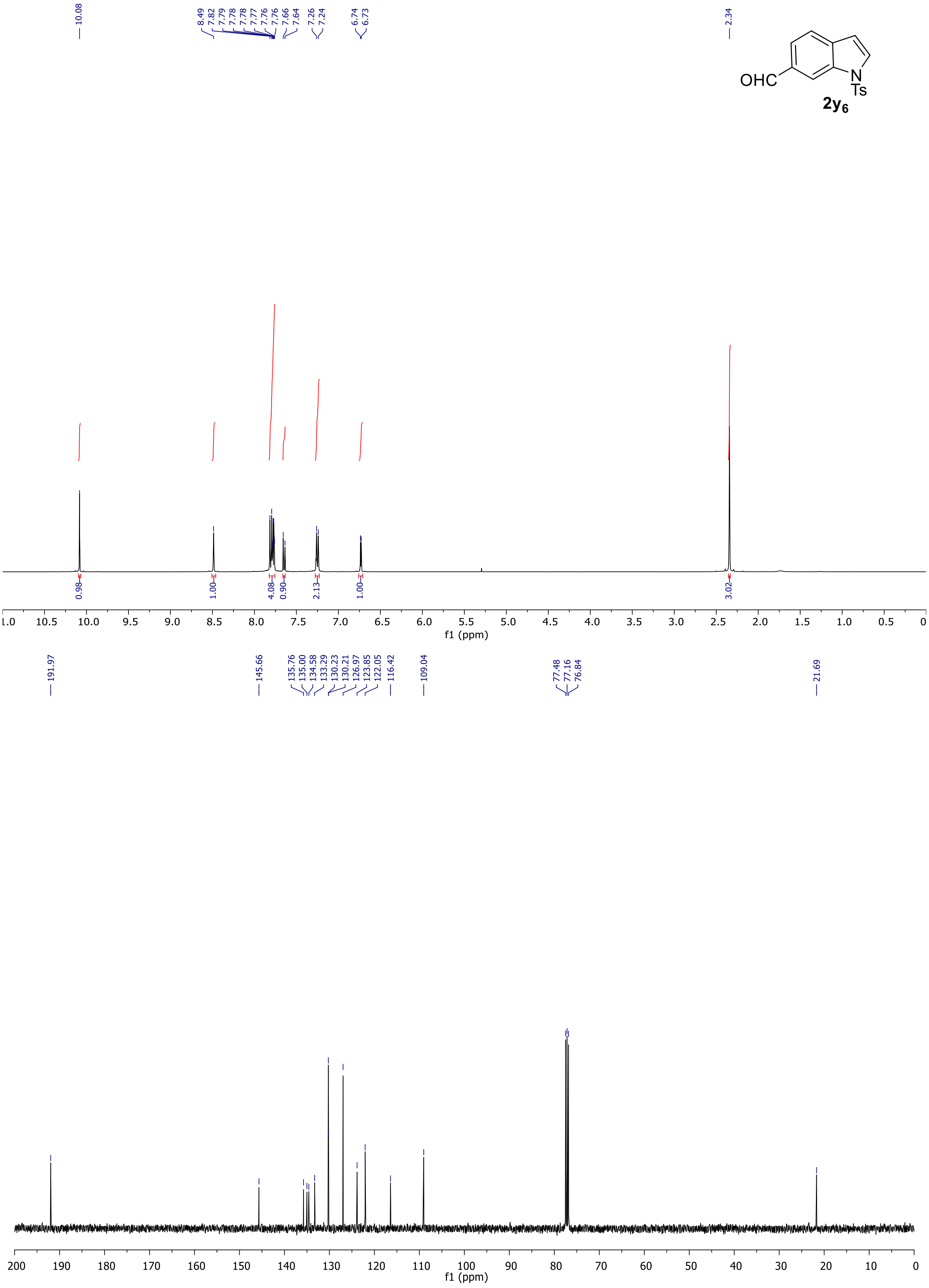

^{1}H NMR (400 MHz) and $^{13}C\{^{1}H\}$ NMR (100 MHz) spectra of **2y6** ($CDCl_3$)

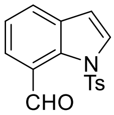

2y$_7$

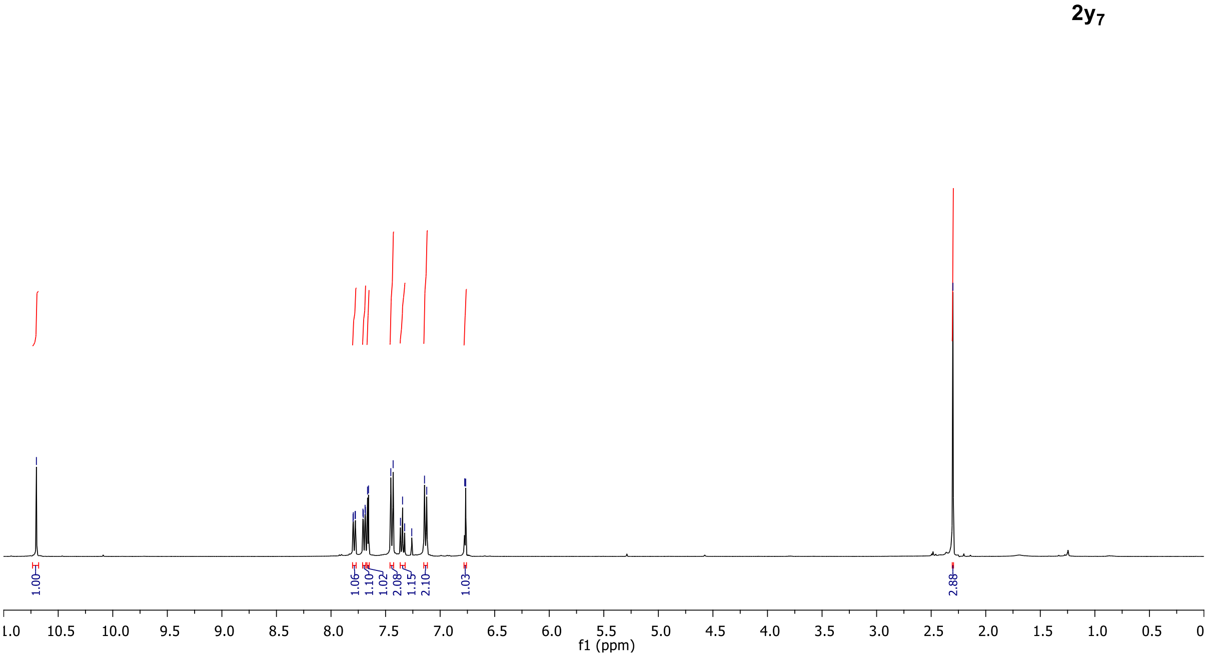

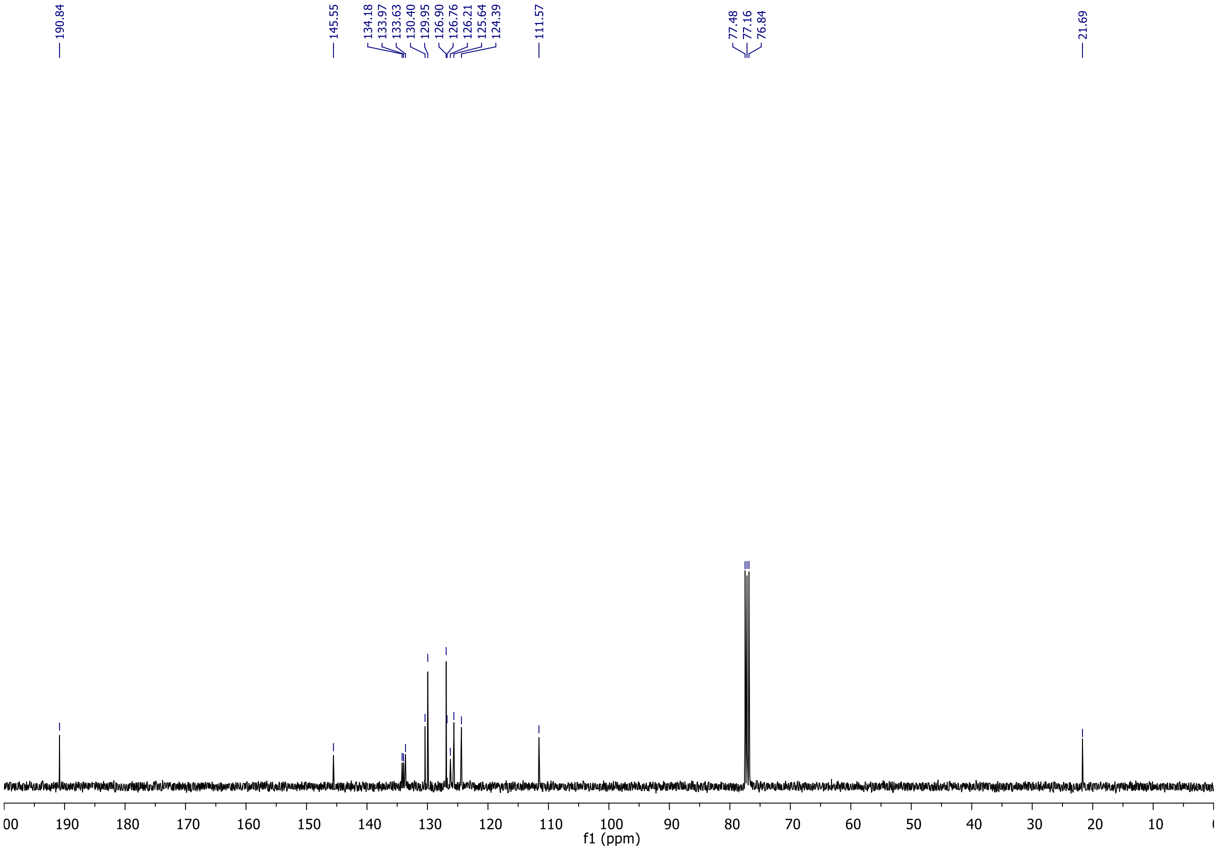

^{1}H NMR (400 MHz) and ^{13}C{^{1}H} NMR (100 MHz) spectra of **2y$_7$** ($CDCl_3$)

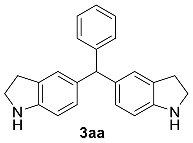

3aa

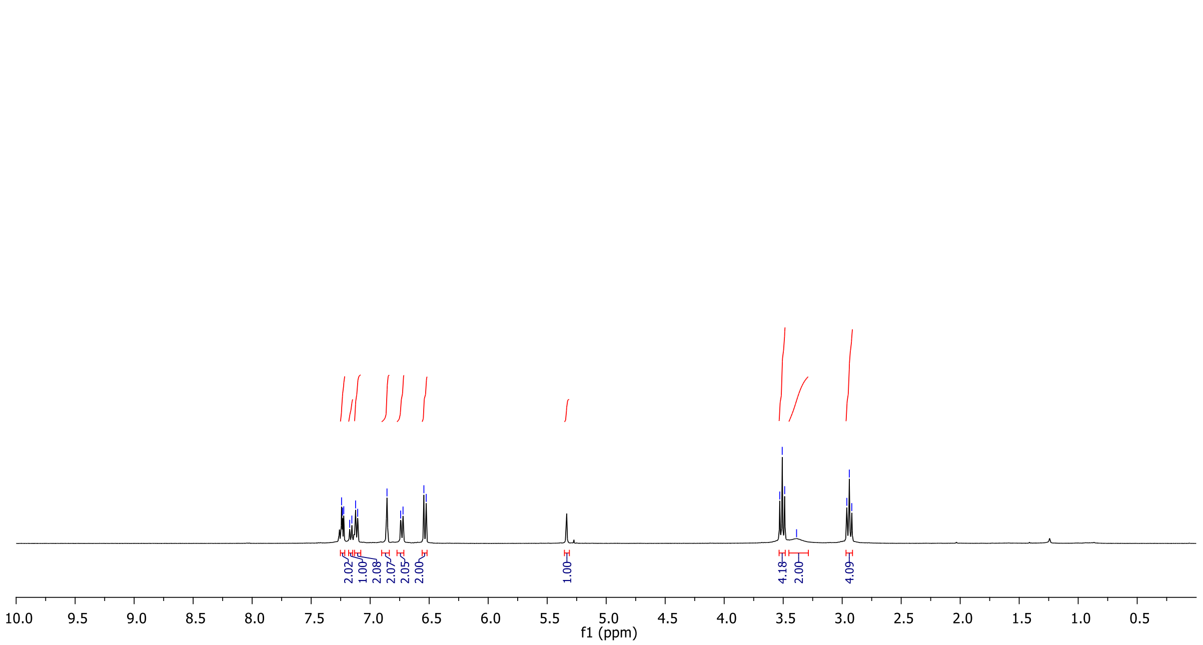

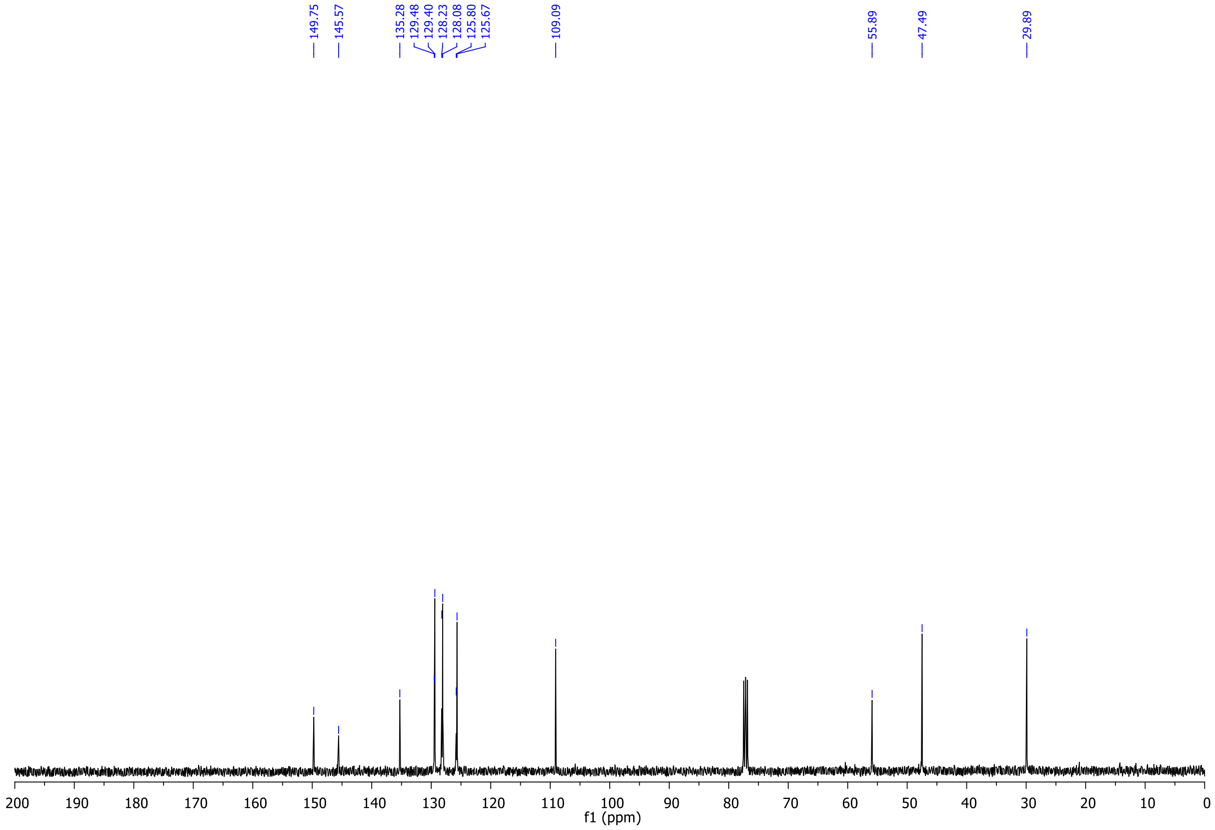

^{1}H NMR (400 MHz) and $^{13}C\{^{1}H\}$ NMR (100 MHz) spectra of **3aa** ($CDCl_3$)

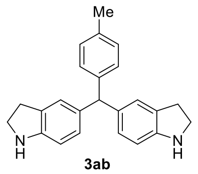

3ab

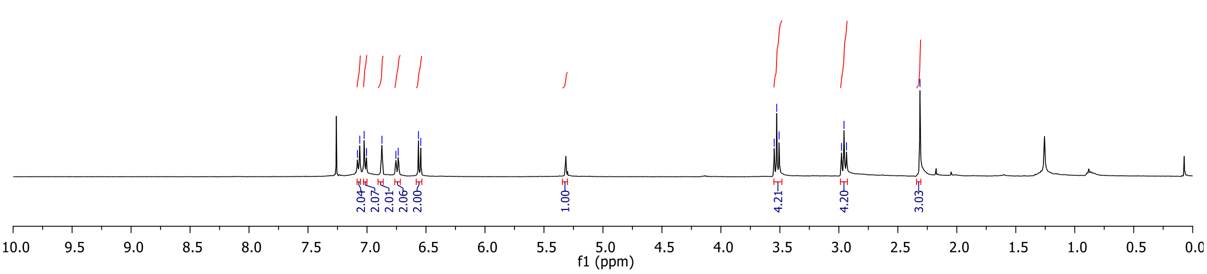

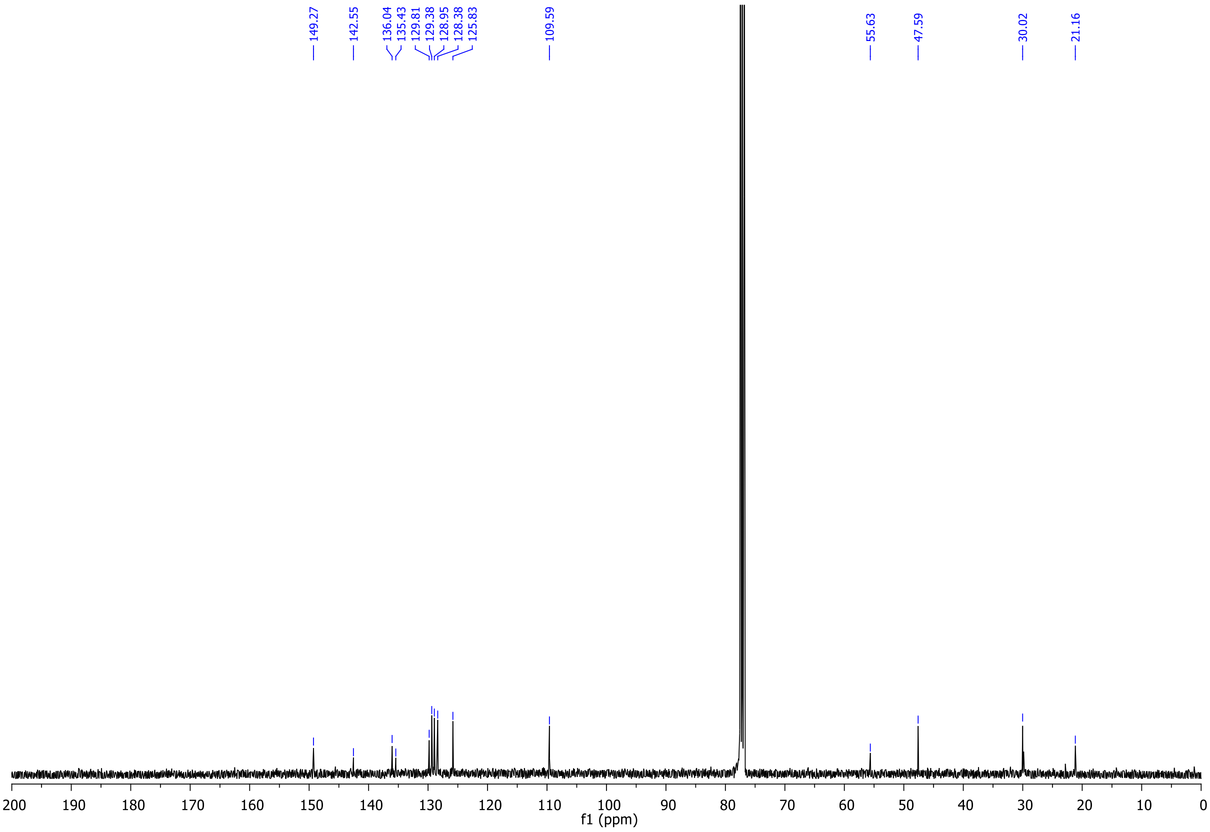

^{1}H NMR (400 MHz) and ^{13}C{^{1}H} NMR (100 MHz) spectra of **3ab** ($CDCl_3$)

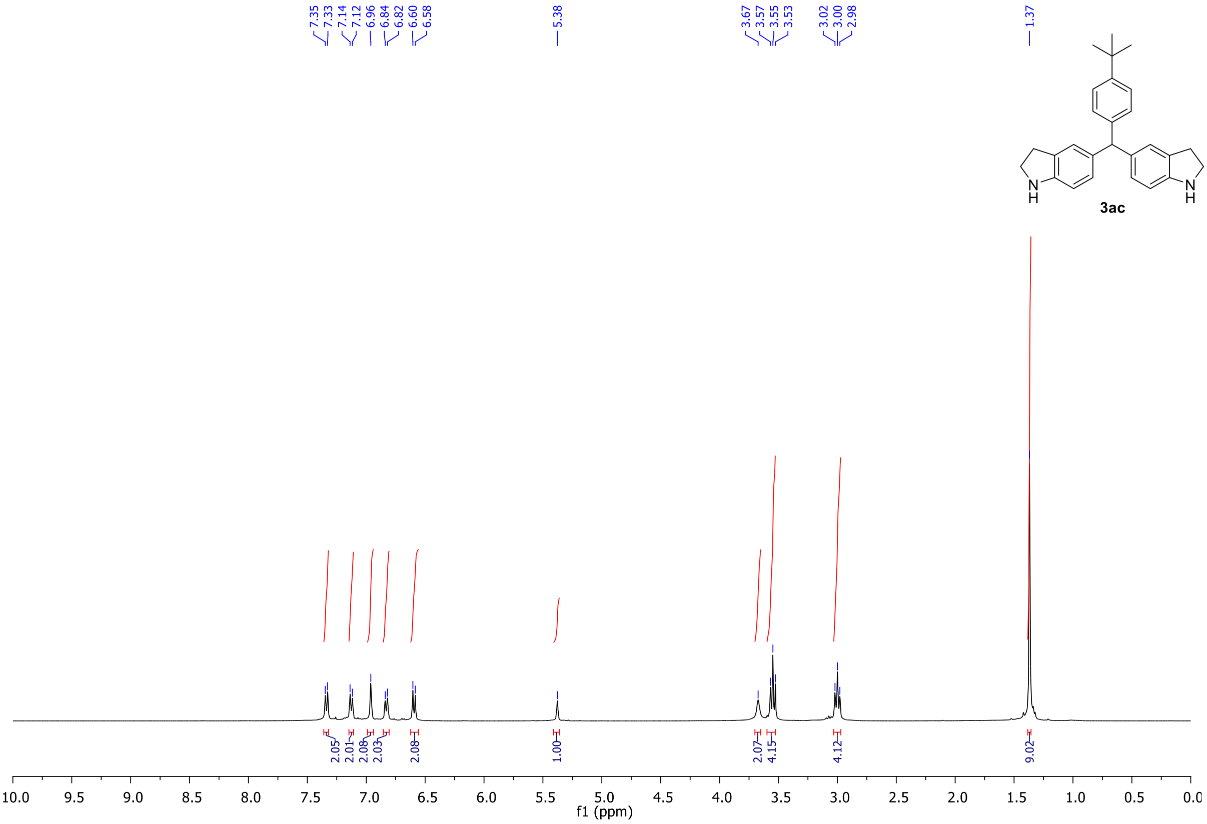

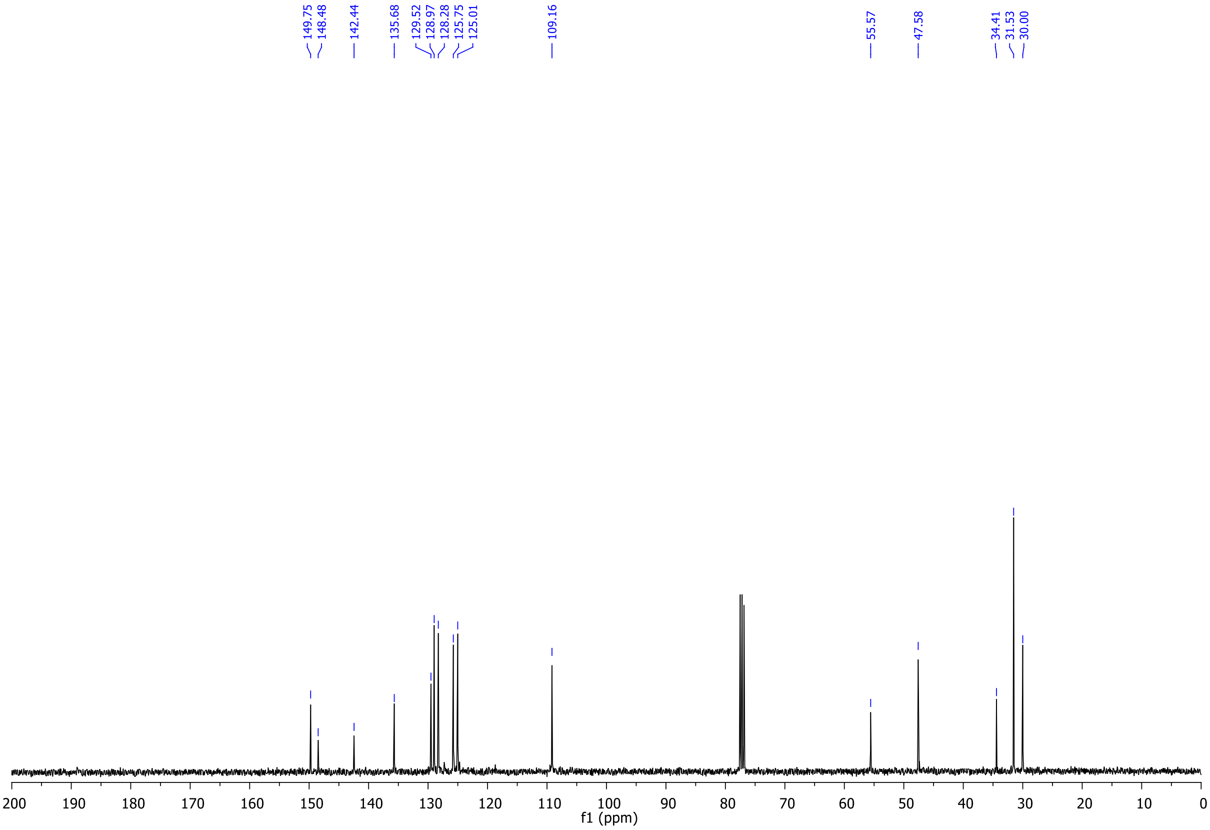

^{1}H NMR (400 MHz) and ^{13}C{^{1}H} NMR (100 MHz) spectra of **3ac** ($CDCl_3$)

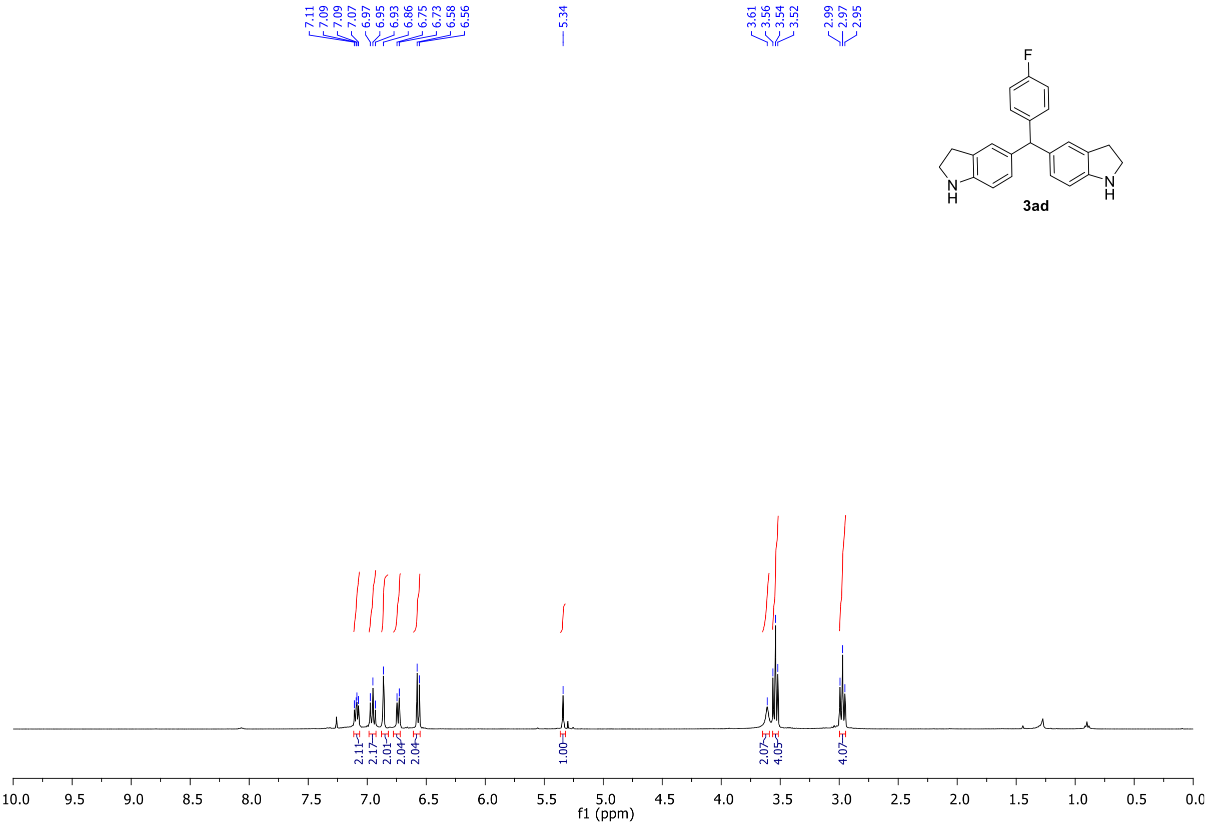

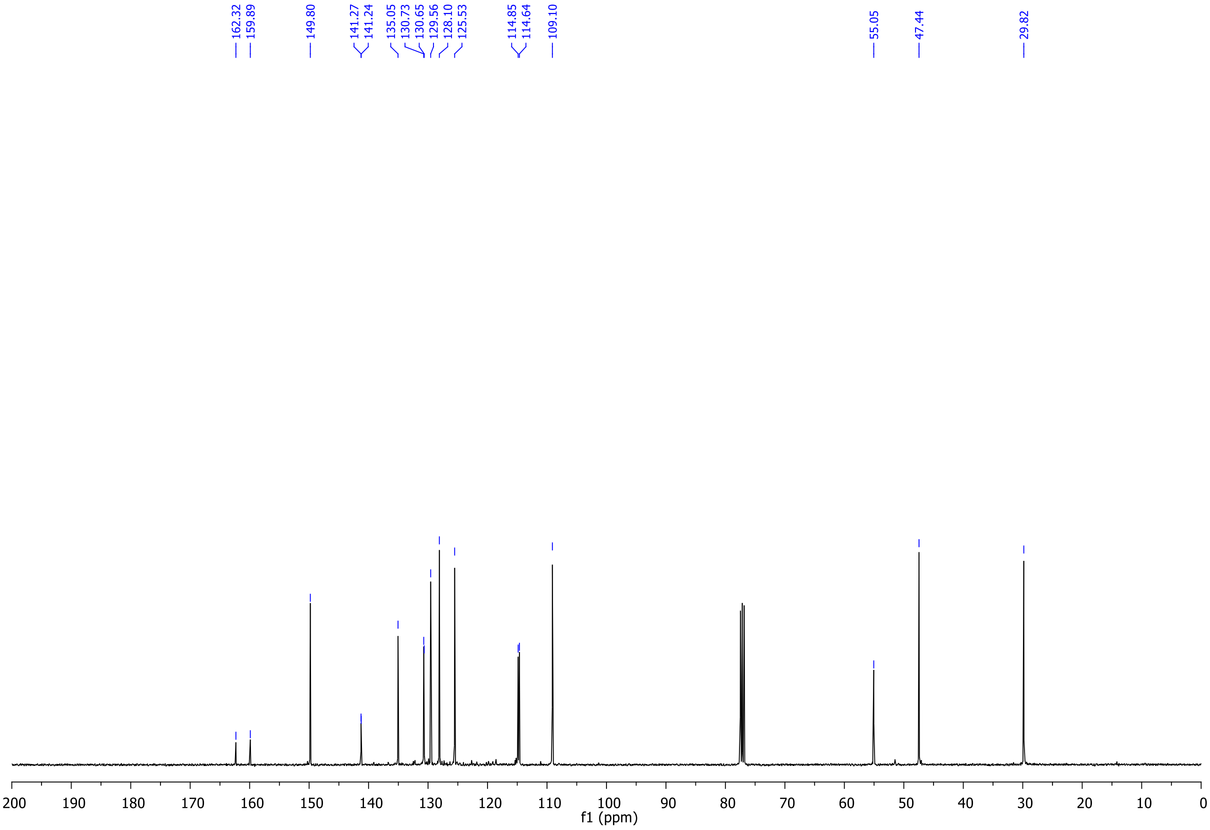

^{1}H NMR (400 MHz) and ^{13}C{^{1}H} NMR (100 MHz) spectra of **3ad** ($CDCl_3$)

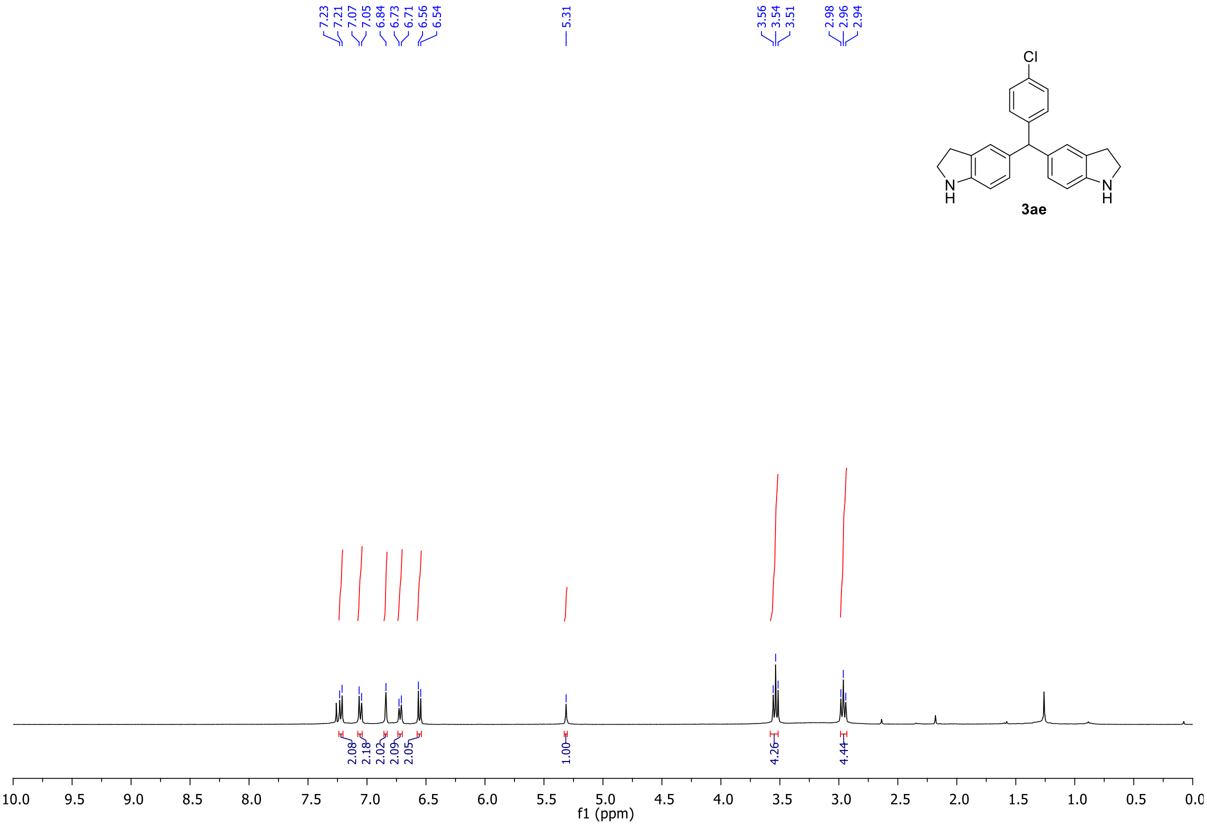

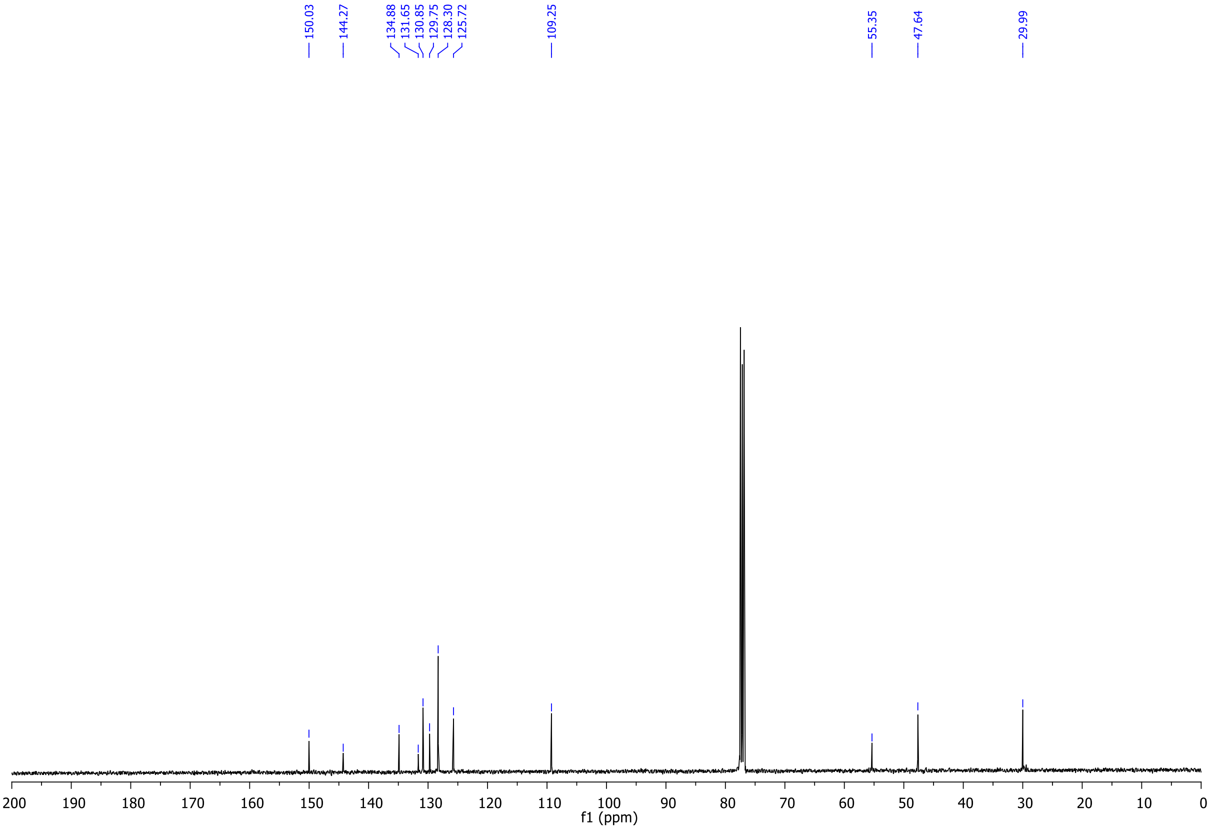

^{1}H NMR (400 MHz) and $^{13}C\{^{1}H\}$ NMR (100 MHz) spectra of **3ae** ($CDCl_3$)

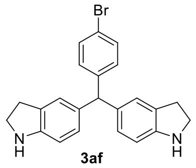

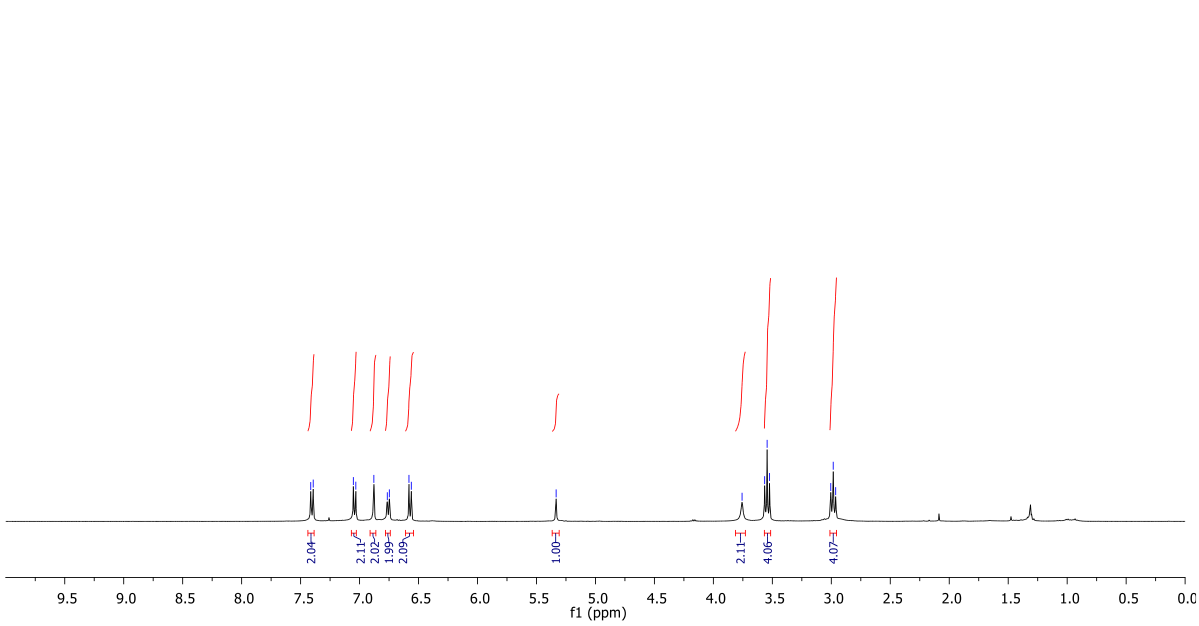

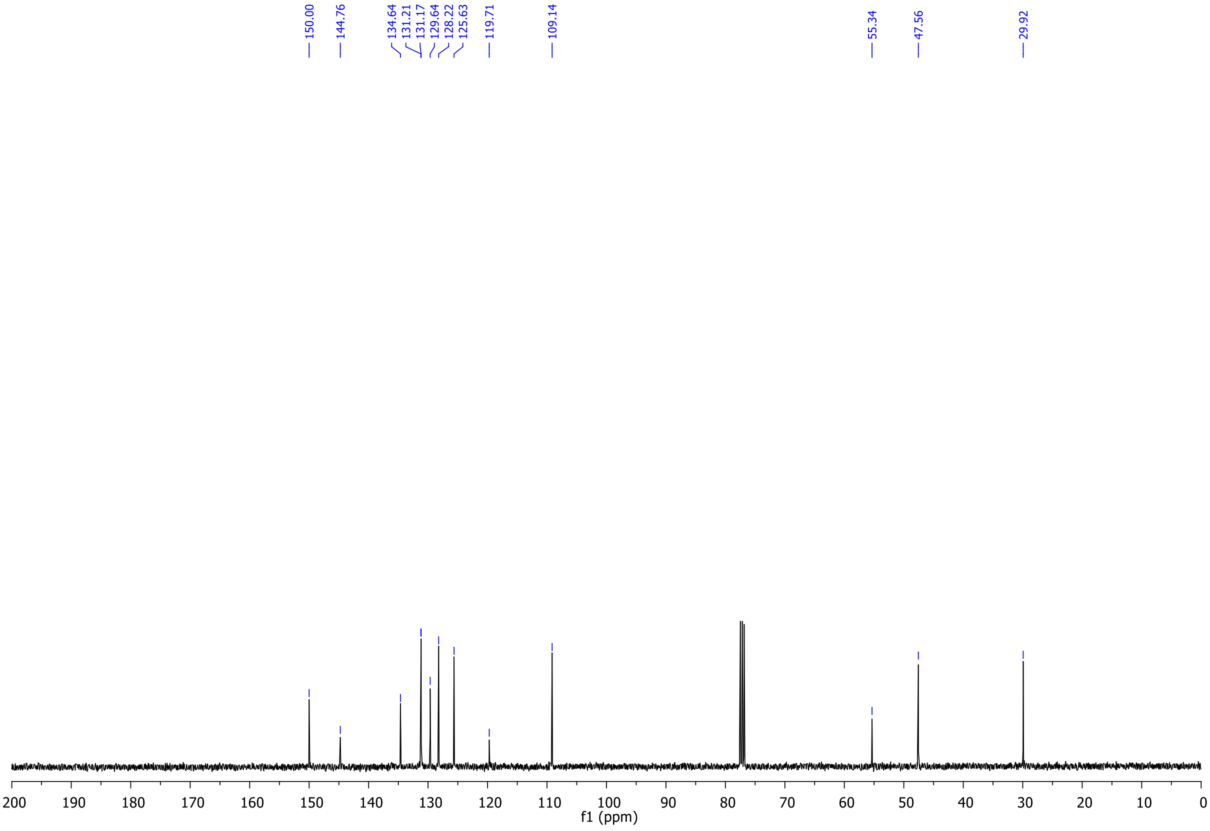

^{1}H NMR (400 MHz) and $^{13}C\{^{1}H\}$ NMR (100 MHz) spectra of **3af** ($CDCl_3$)

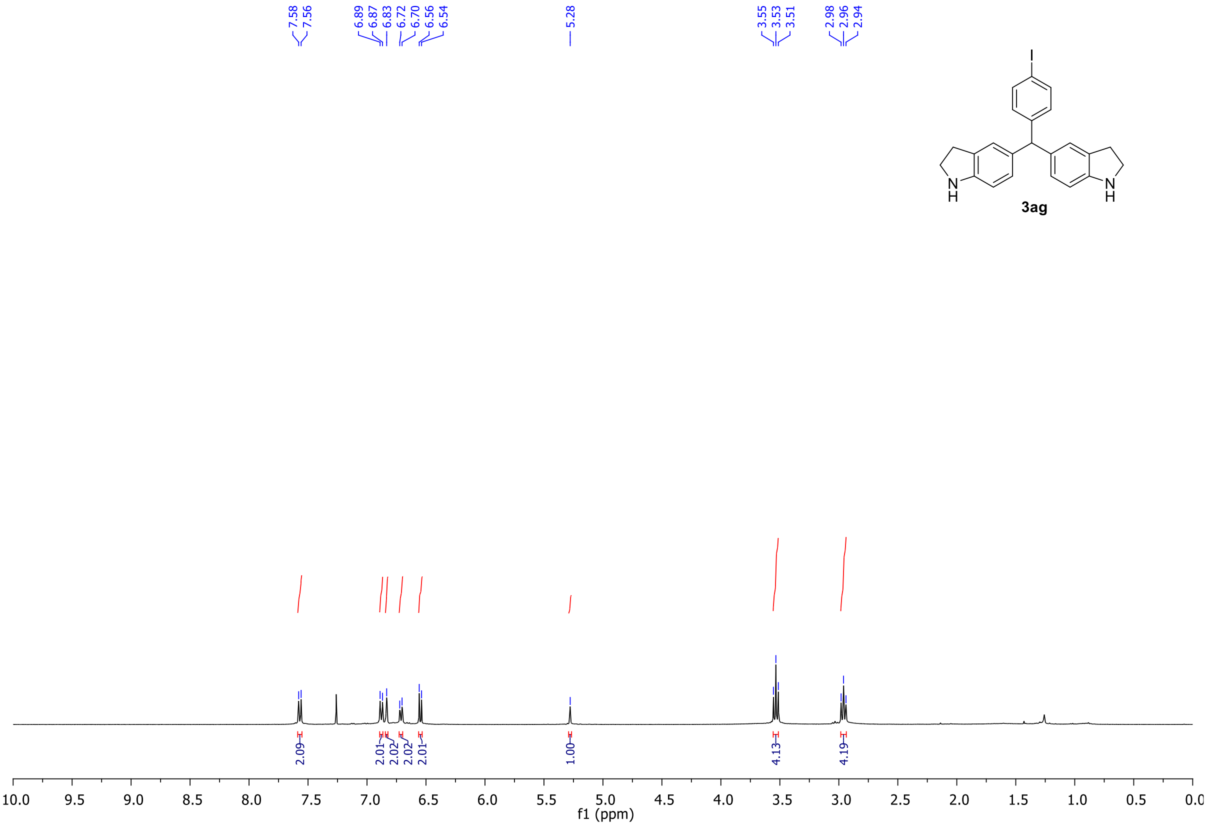

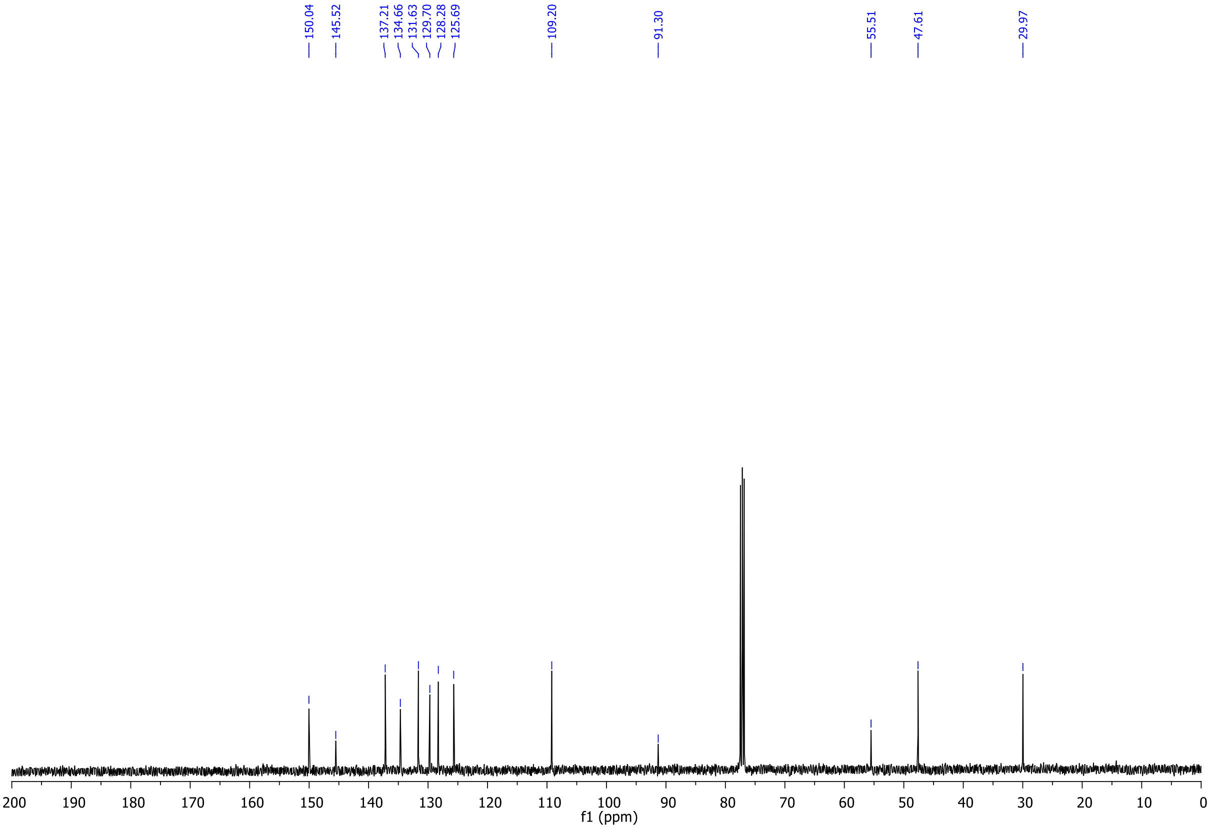

^{1}H NMR (400 MHz) and ^{13}C{^{1}H} NMR (100 MHz) spectra of **3ag** ($CDCl_3$)

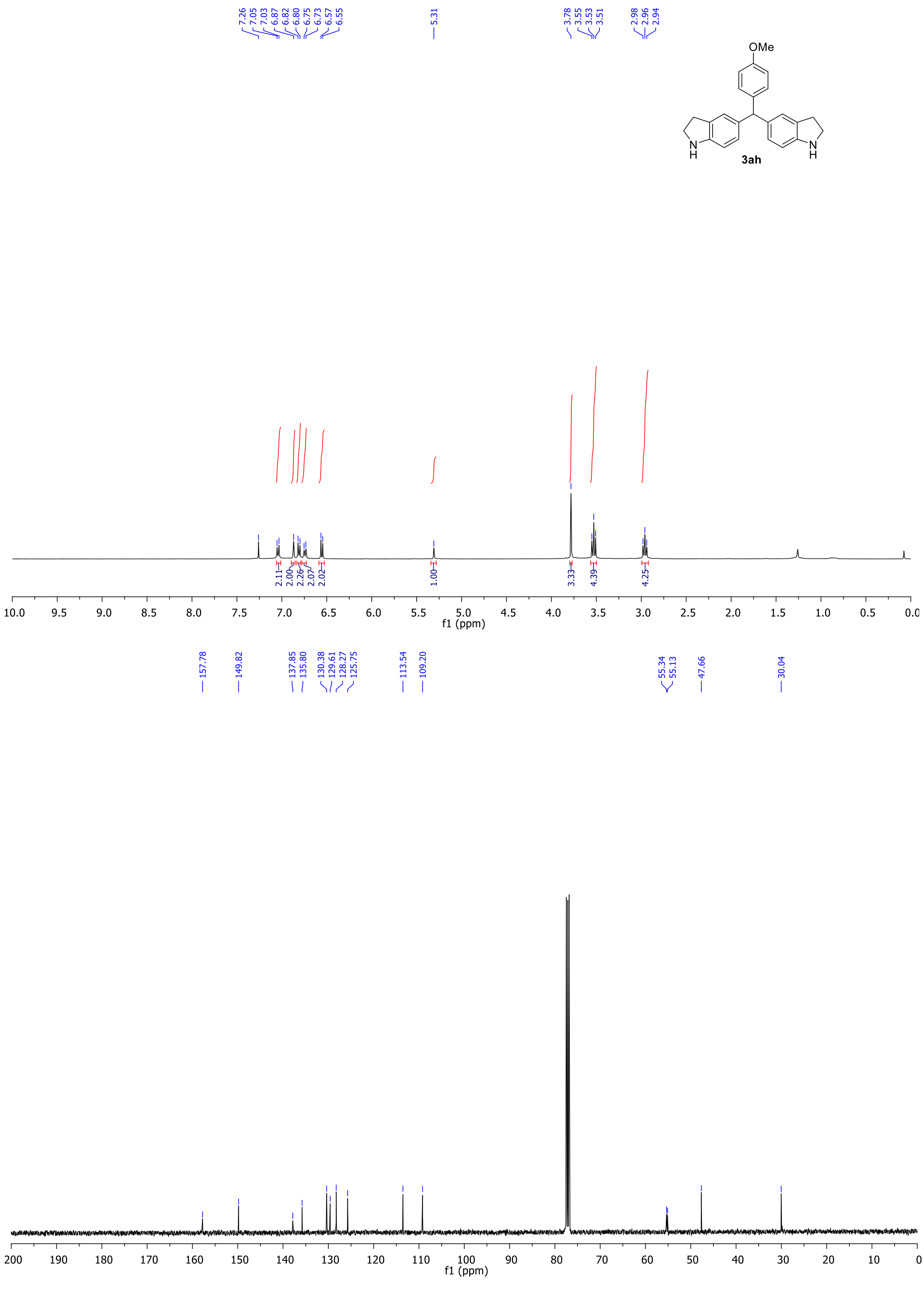

^{1}H NMR (400 MHz) and ^{13}C{^{1}H} NMR (100 MHz) spectra of **3ah** ($CDCl_3$)

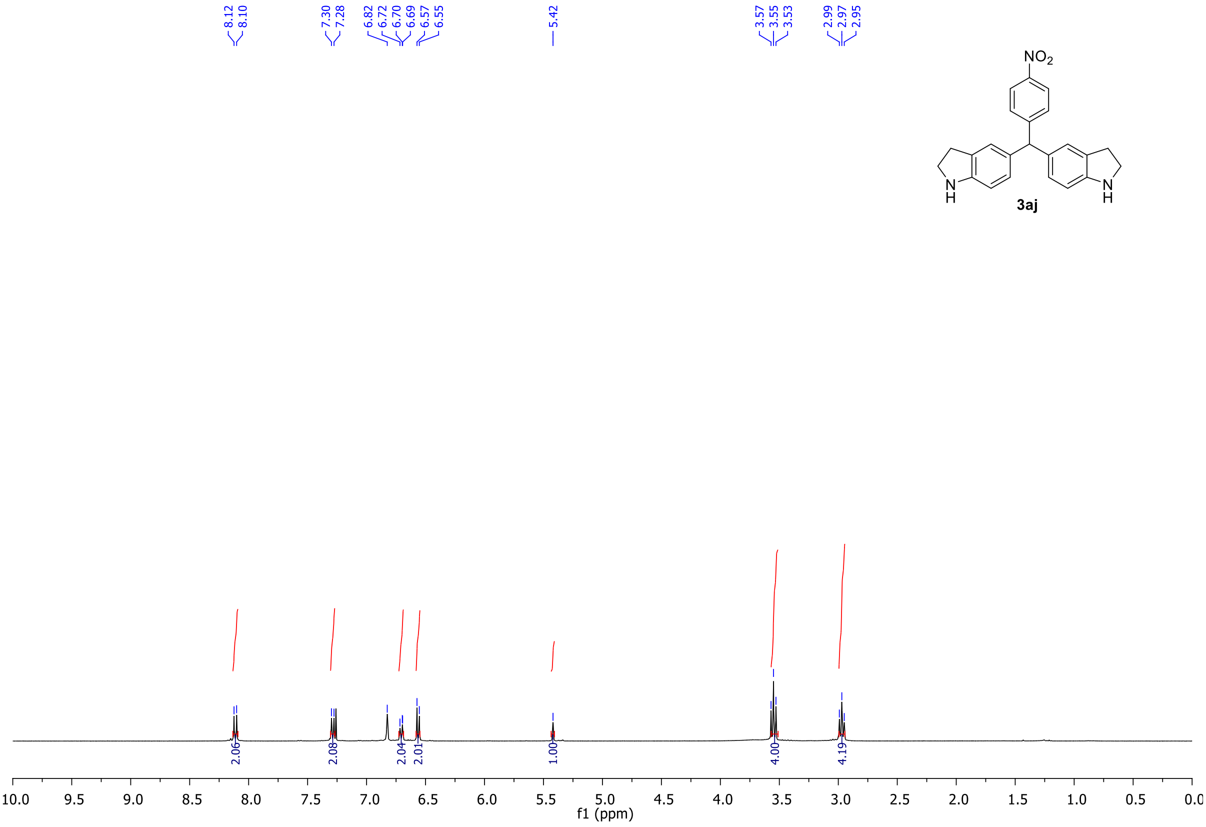

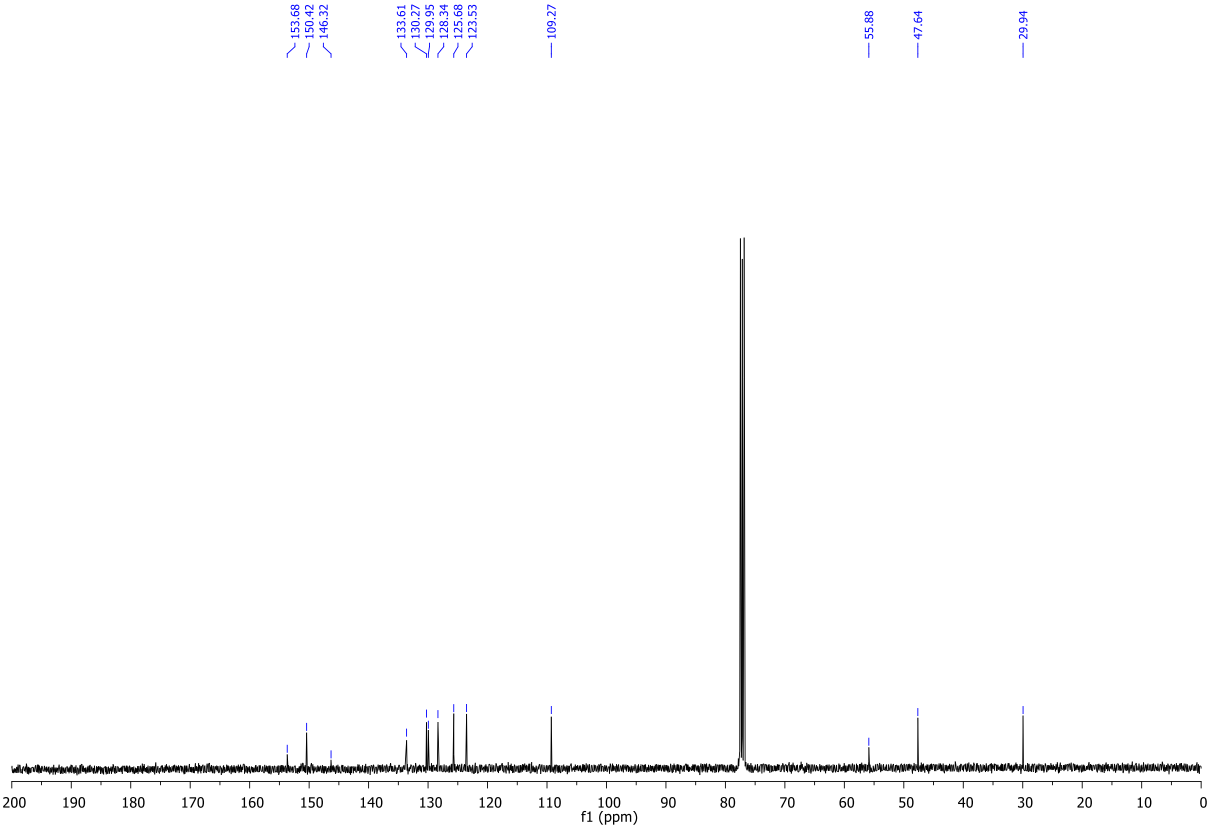

^{1}H NMR (400 MHz) and ^{13}C{^{1}H} NMR (100 MHz) spectra of **3aj** ($CDCl_3$)

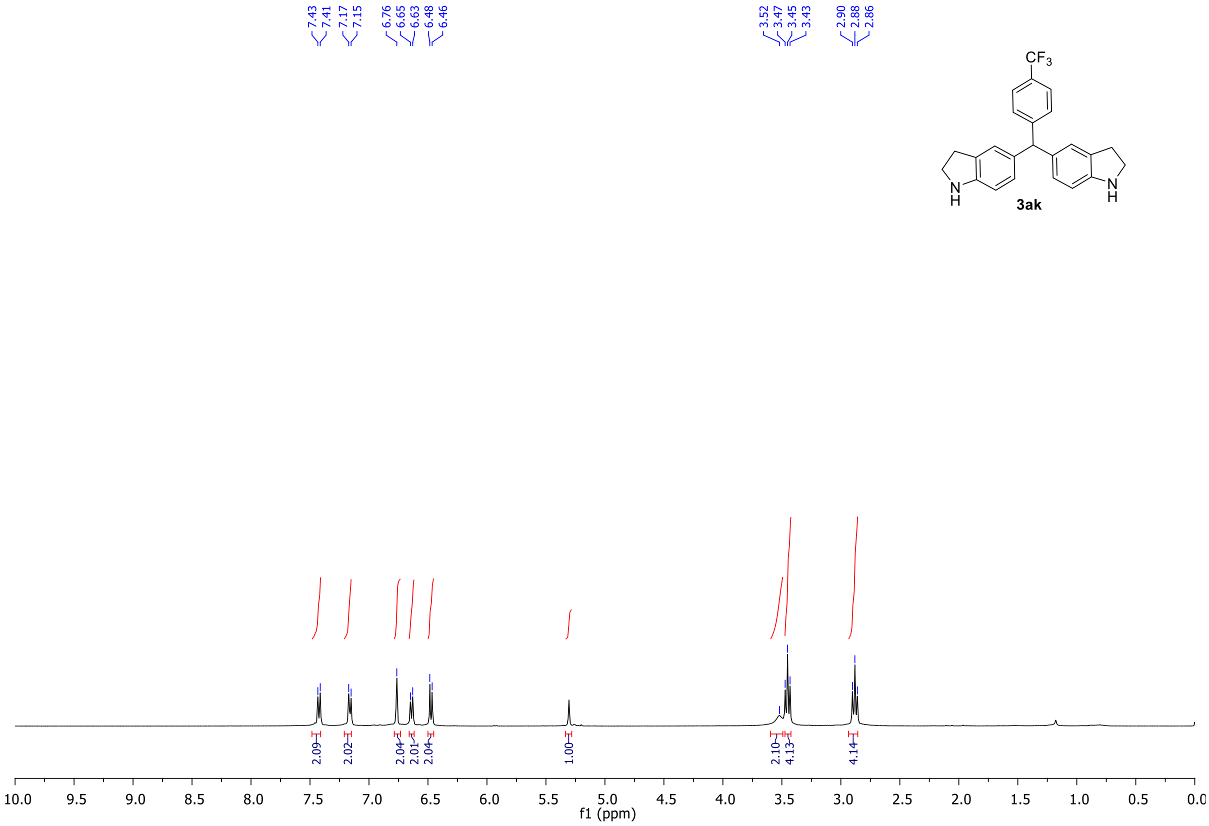

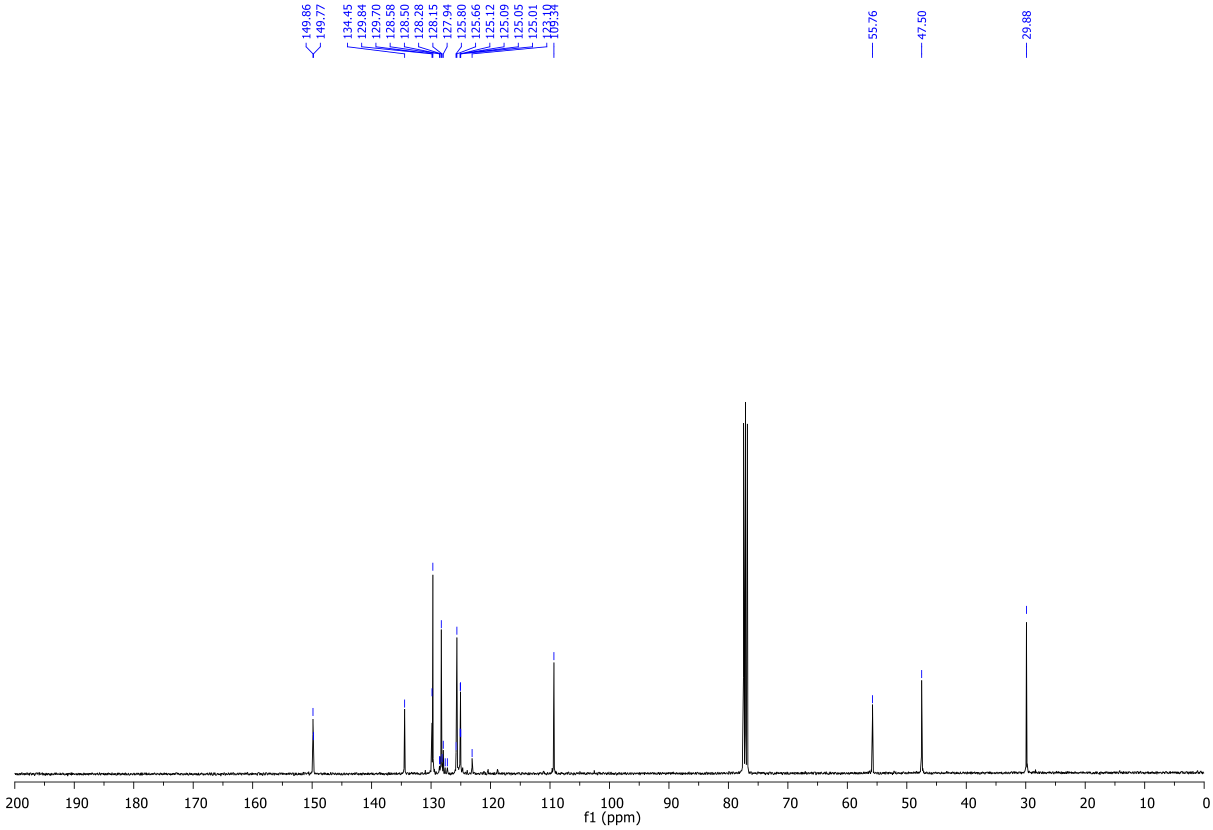

^{1}H NMR (400 MHz) and ^{13}C{^{1}H} NMR (100 MHz) spectra of **3ak** ($CDCl_3$)

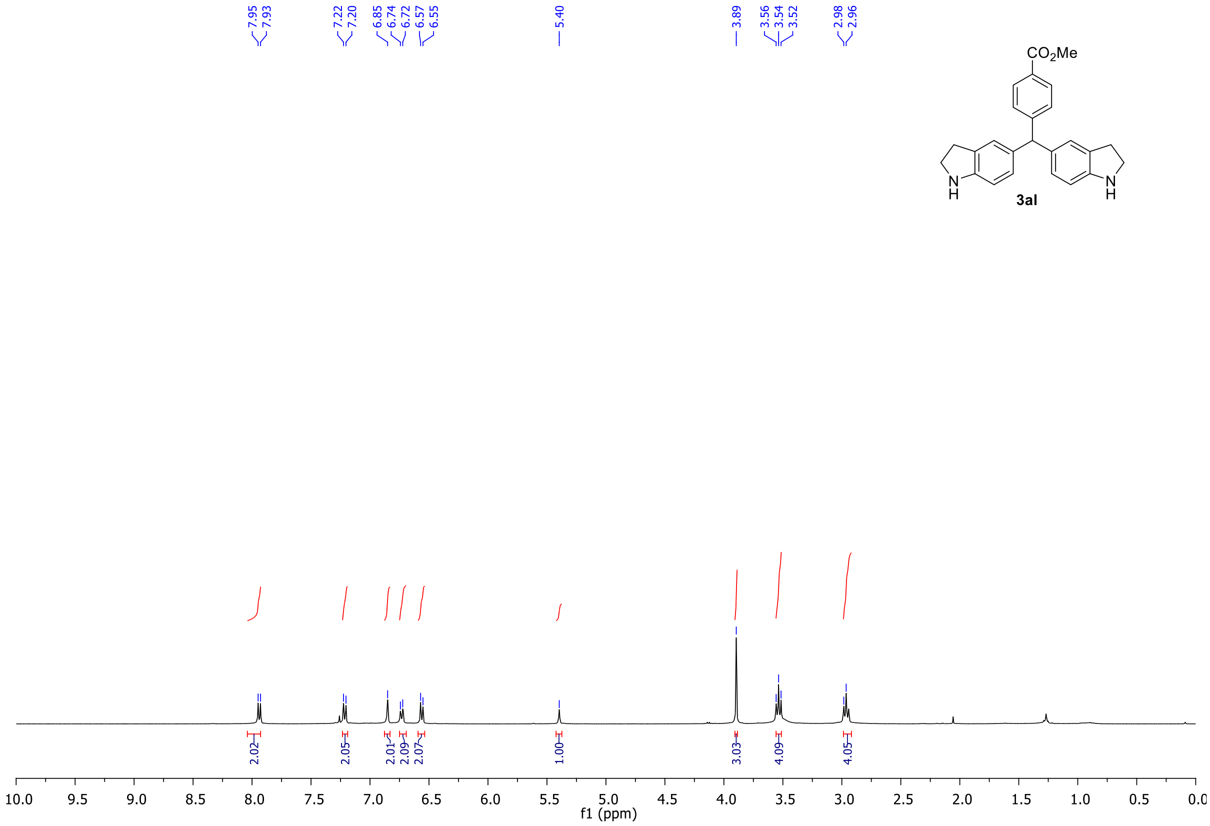

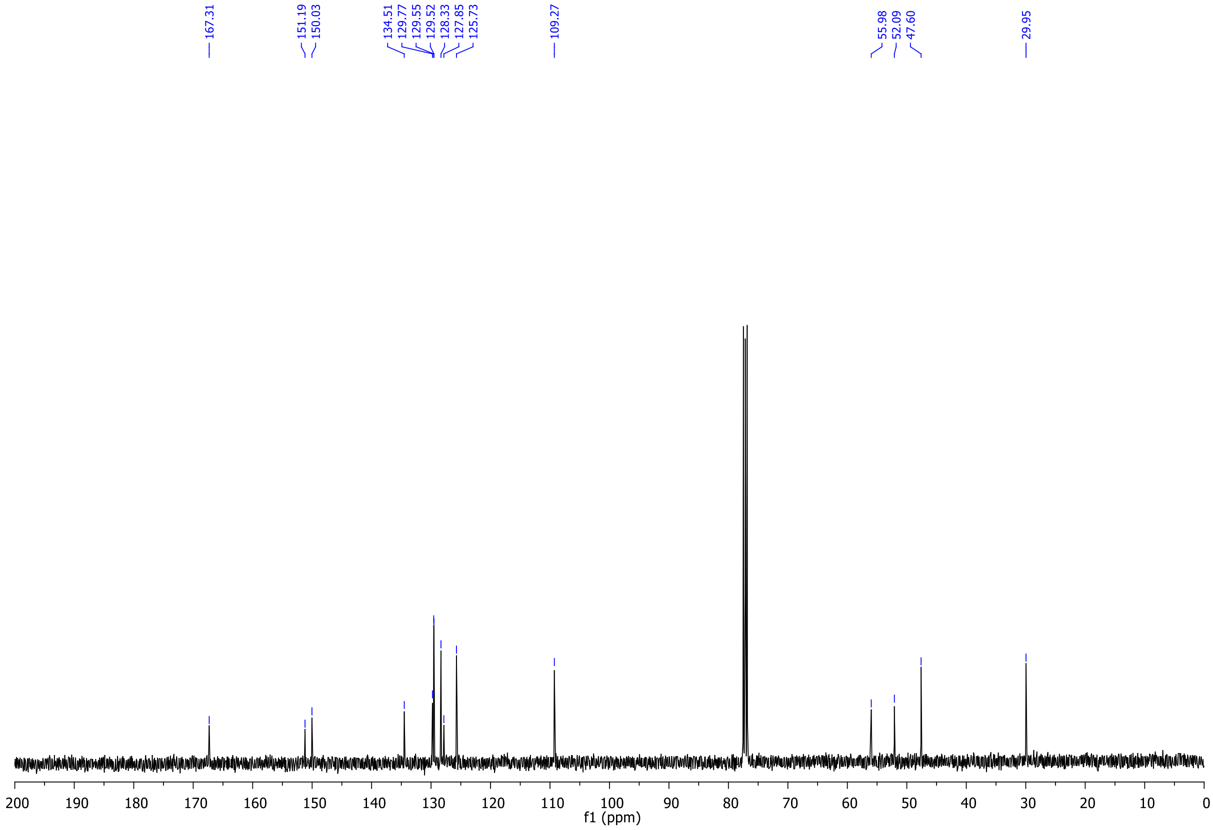

^{1}H NMR (400 MHz) and ^{13}C{^{1}H} NMR (100 MHz) spectra of **3al** ($CDCl_3$)

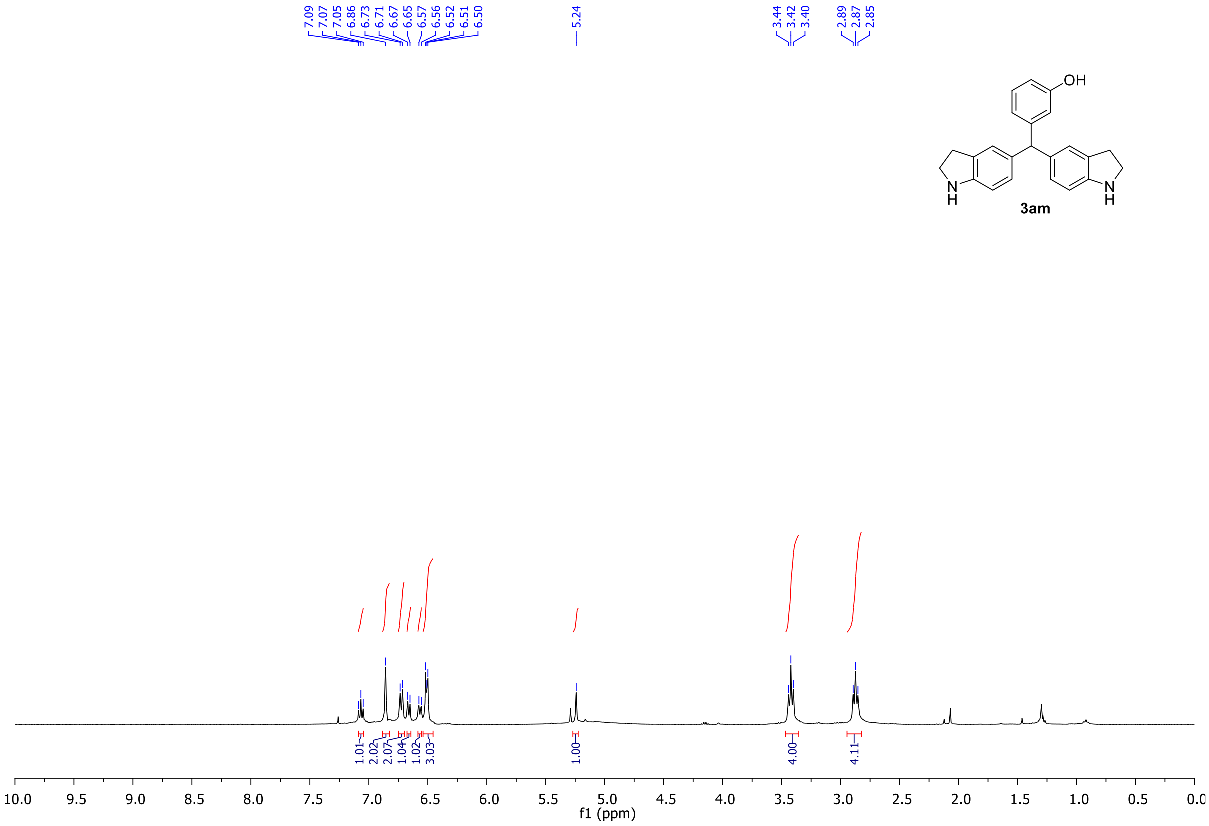

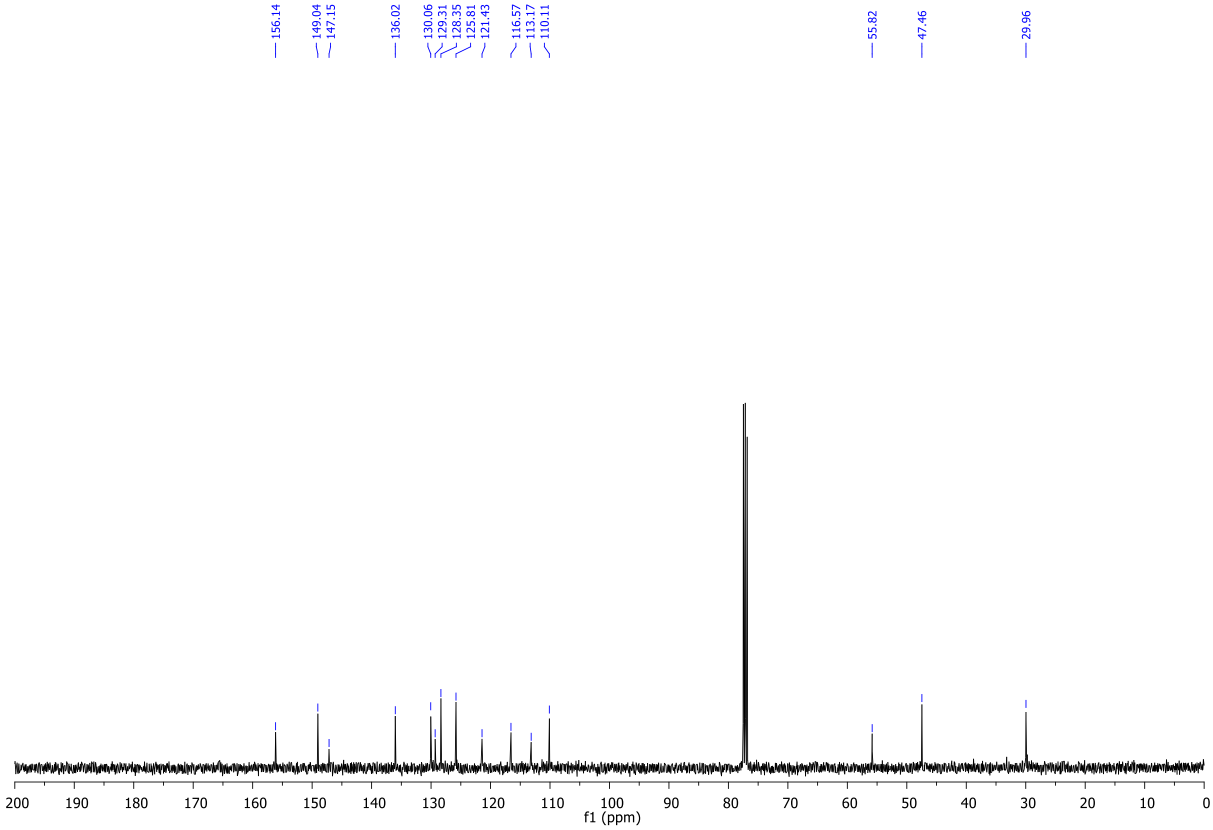

^{1}H NMR (400 MHz) and ^{13}C{^{1}H} NMR (100 MHz) spectra of **3am** ($CDCl_3$)

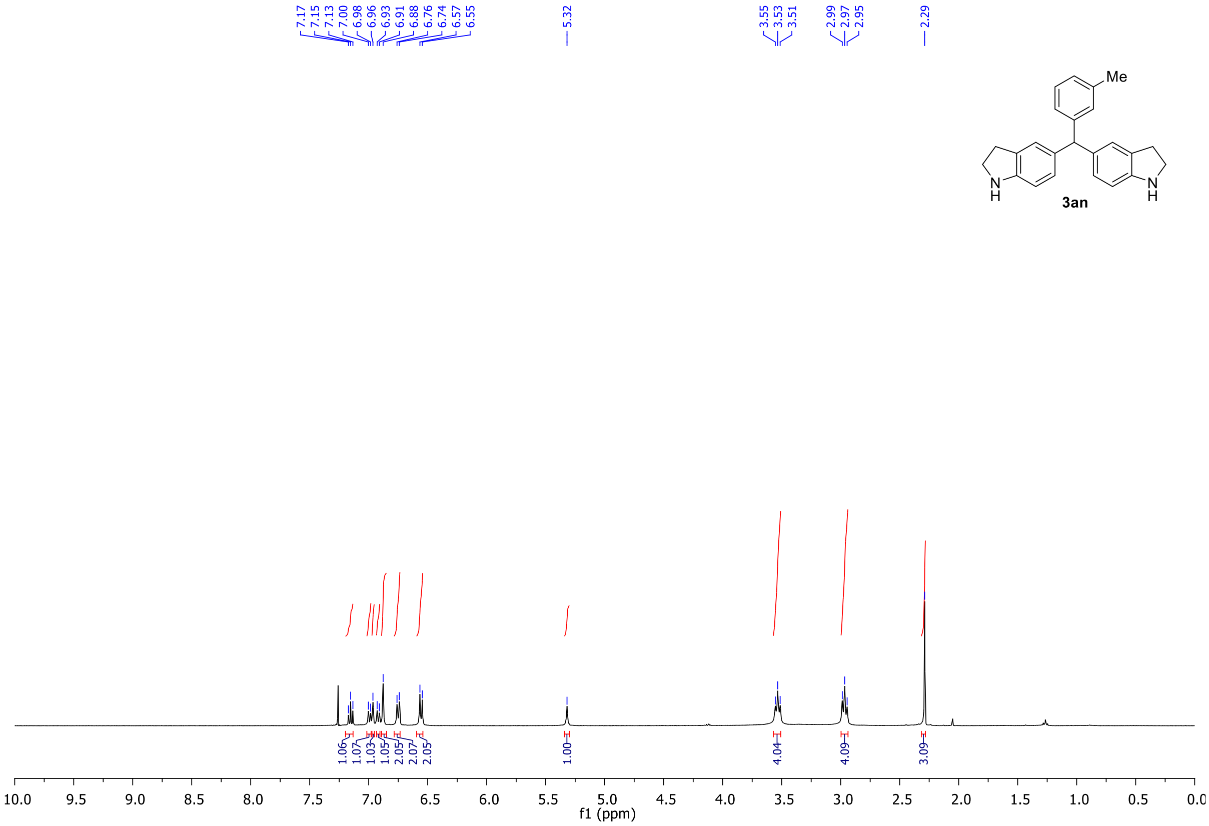

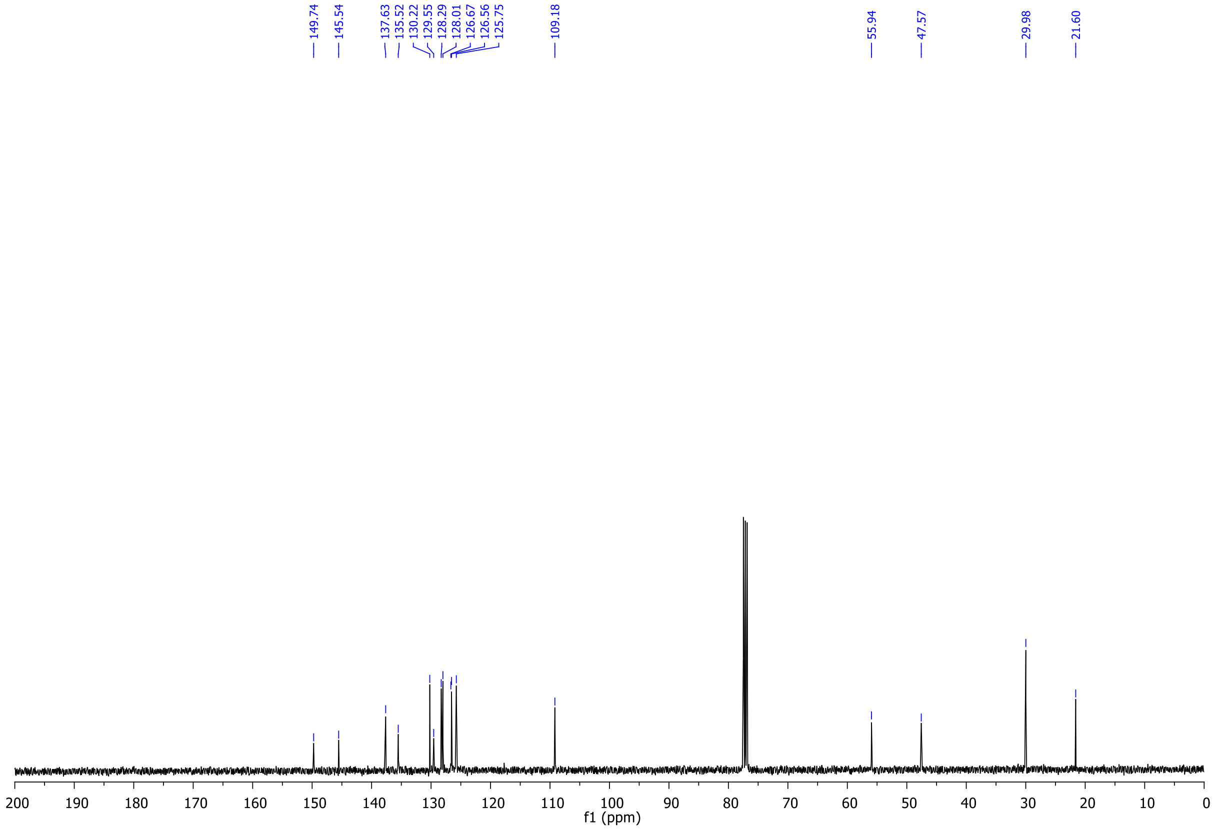

^{1}H NMR (400 MHz) and ^{13}C{^{1}H} NMR (100 MHz) spectra of **3an** ($CDCl_3$)

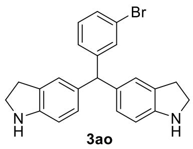

3ao

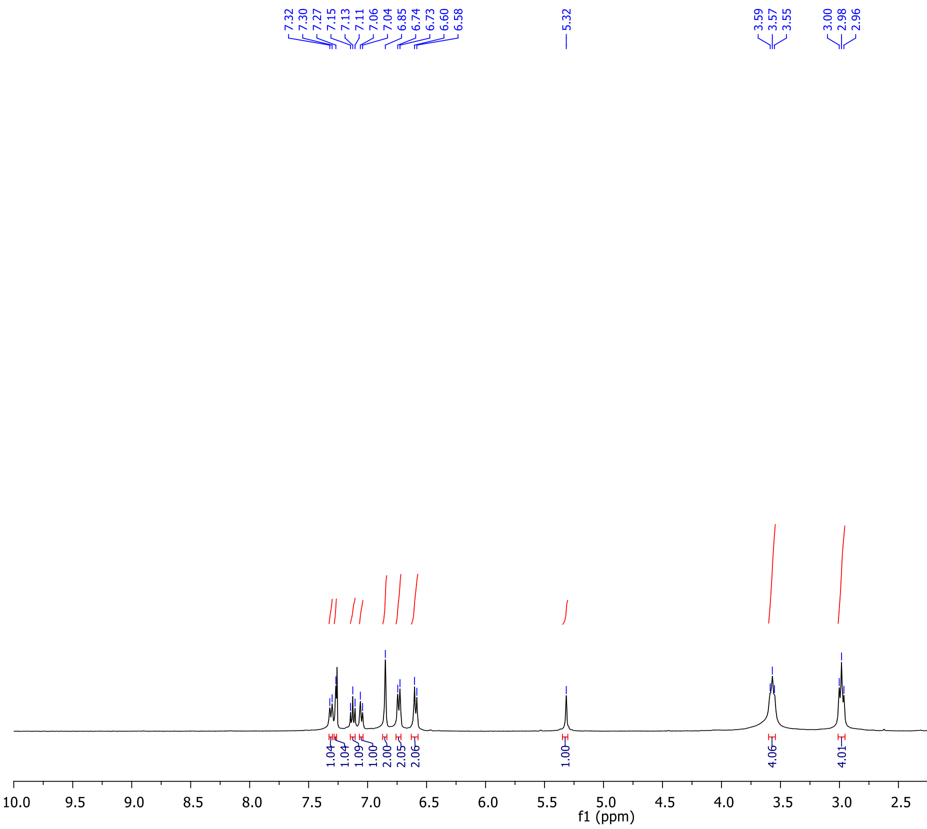

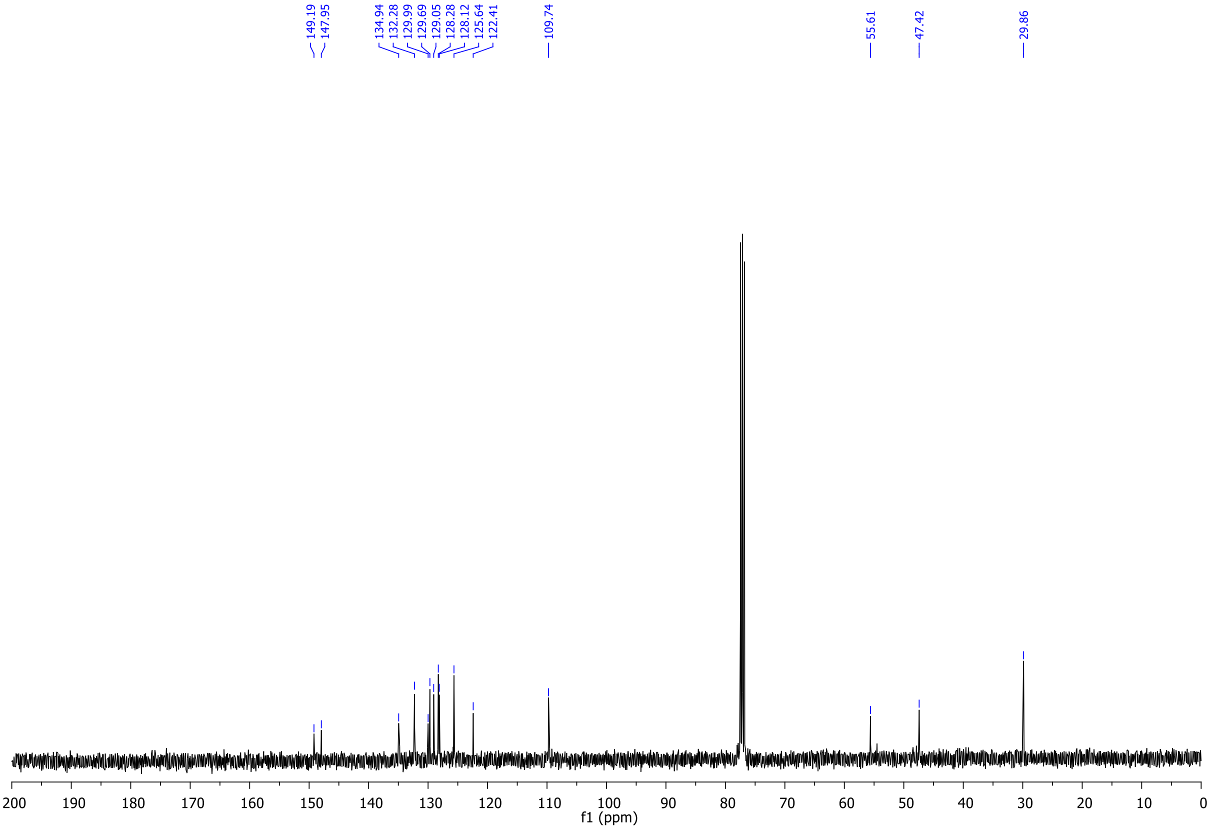

^{1}H NMR (400 MHz) and ^{13}C{^{1}H} NMR (100 MHz) spectra of **3ao** ($CDCl_3$)

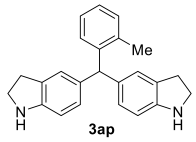

3ap

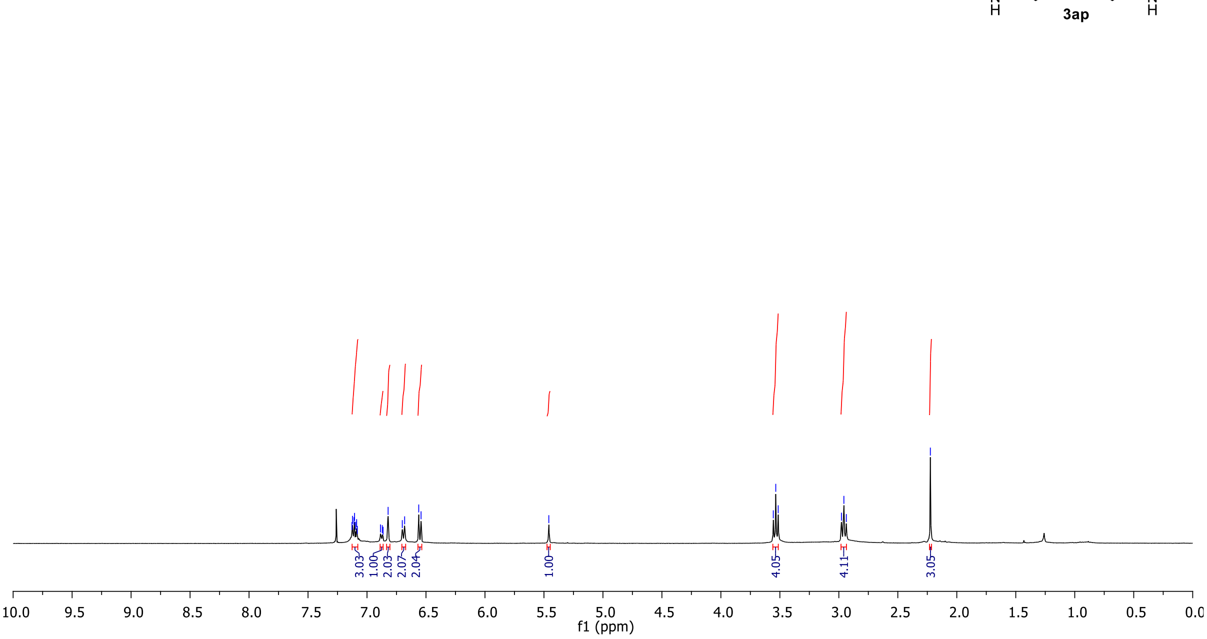

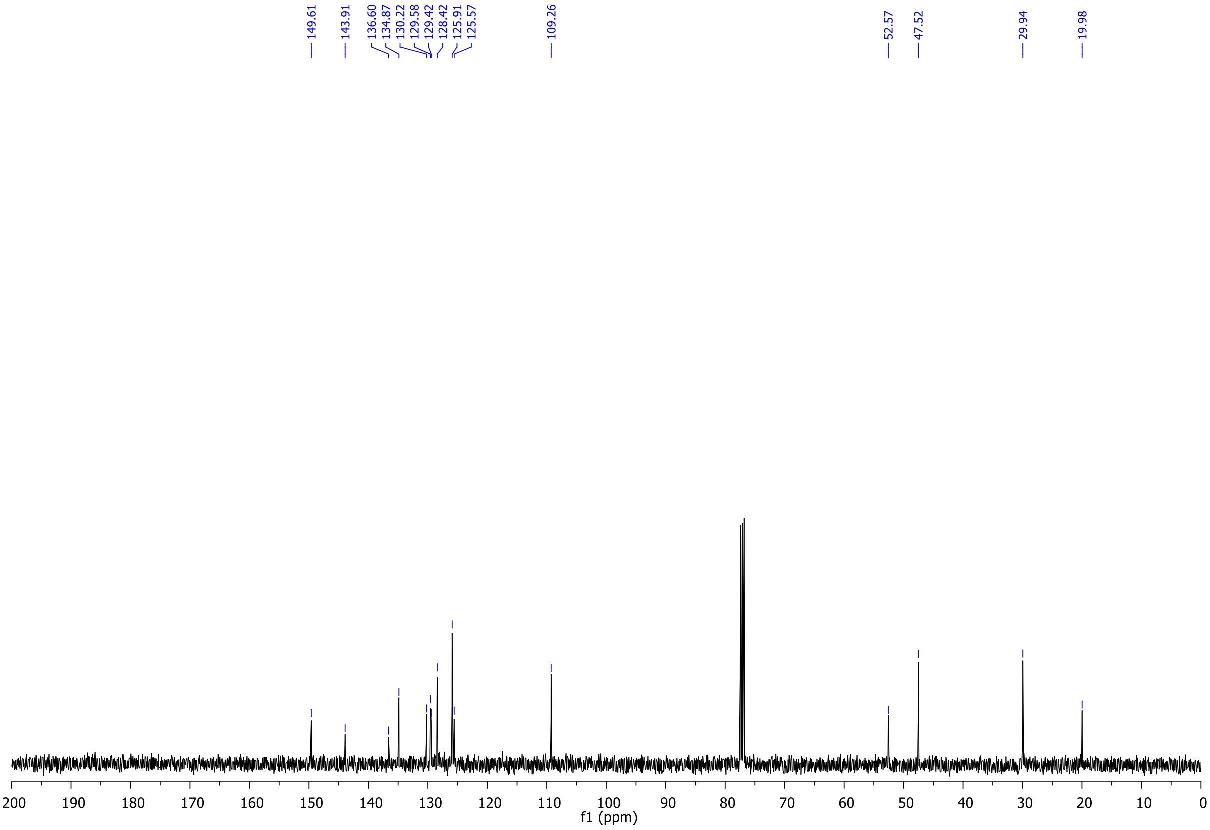

^{1}H NMR (400 MHz) and $^{13}C\{^{1}H\}$ NMR (100 MHz) spectra of **3ap** ($CDCl_3$)

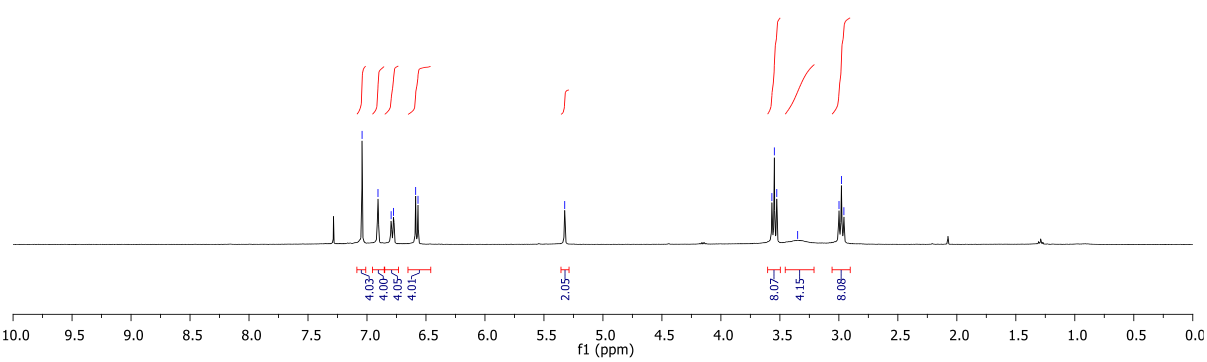

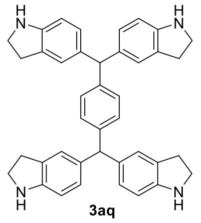

3aq

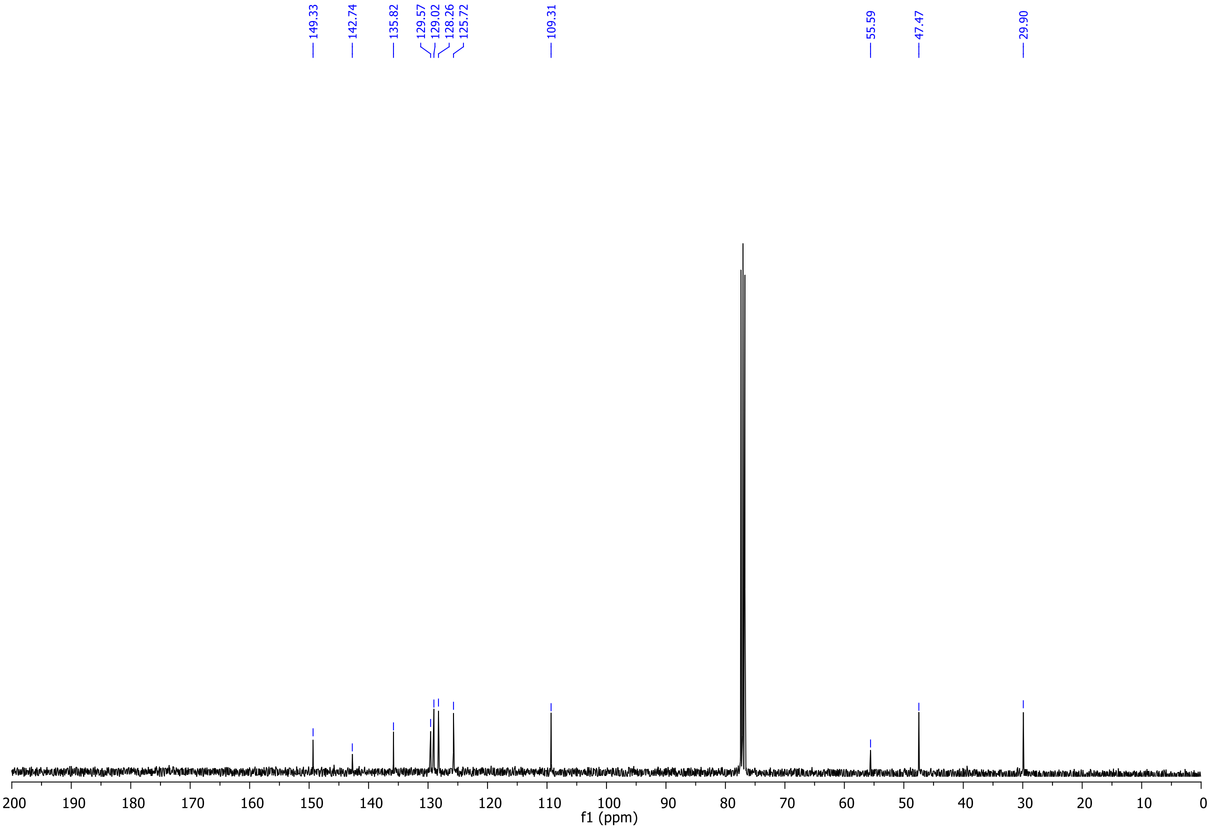

^{1}H NMR (400 MHz) and ^{13}C{^{1}H} NMR (100 MHz) spectra of **3aq** ($CDCl_3$)

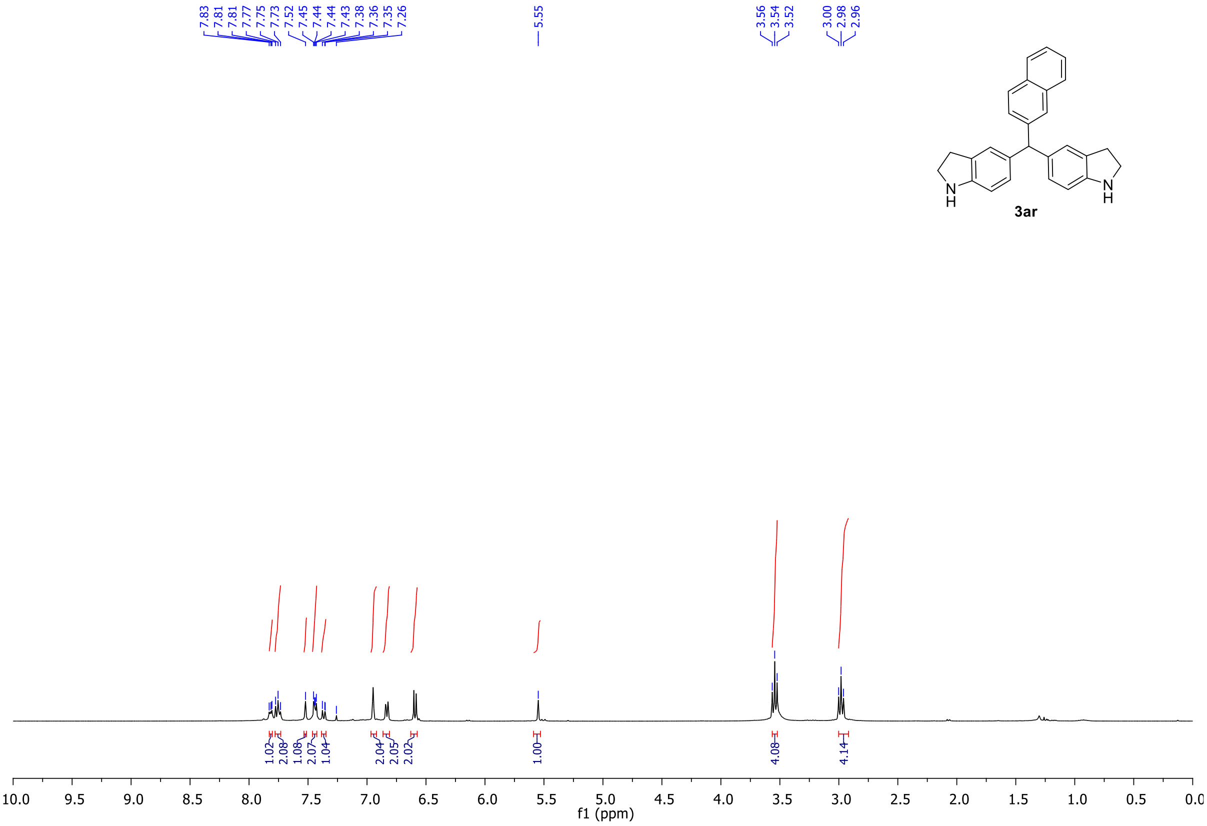

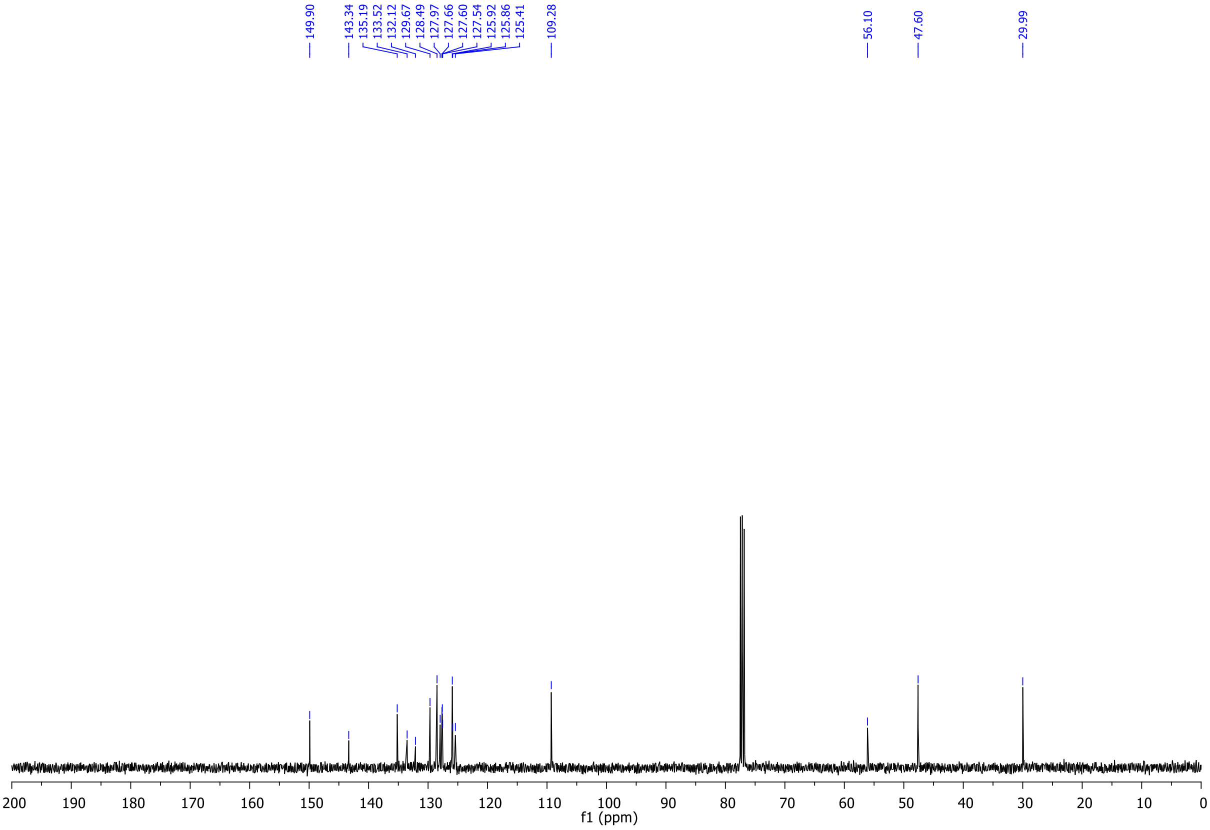

^{1}H NMR (400 MHz) and ^{13}C{^{1}H} NMR (100 MHz) spectra of **3ar** ($CDCl_3$)

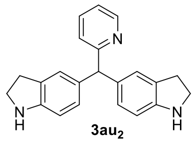

$\mathbf{3au_2}$

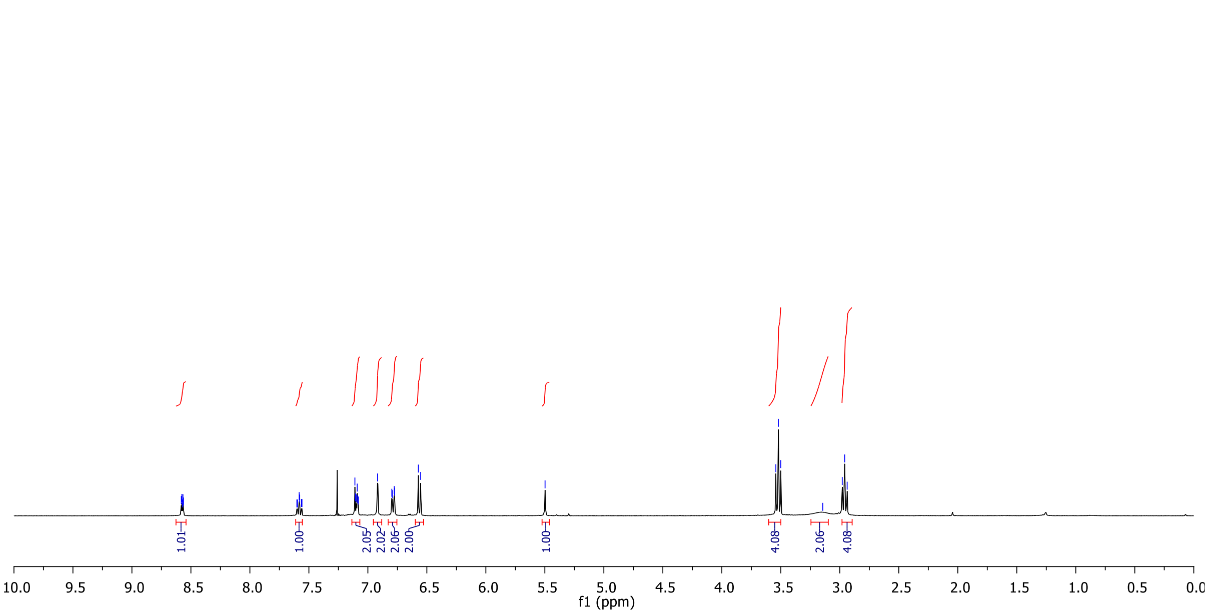

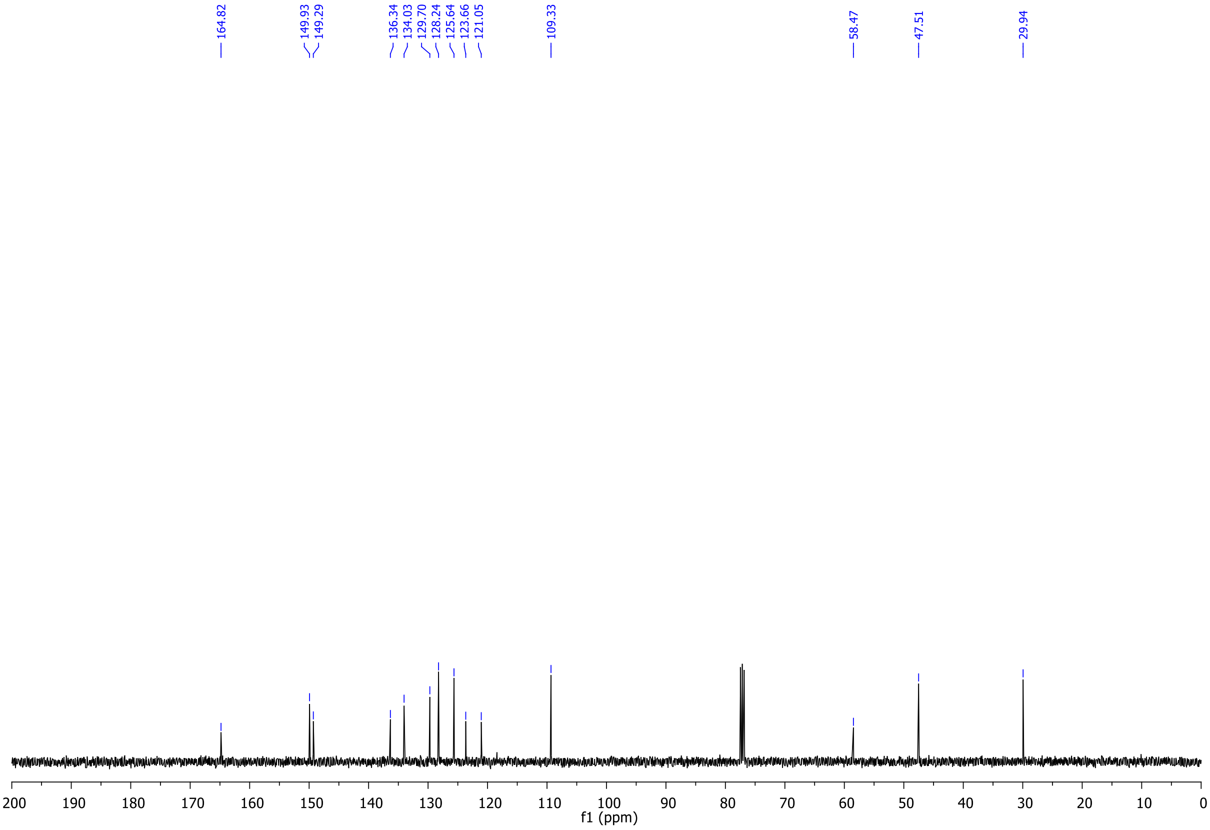

^{1}H NMR (400 MHz) and ^{13}C{^{1}H} NMR (100 MHz) spectra of **$3au_2$** ($CDCl_3$)

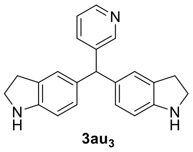

3au₃

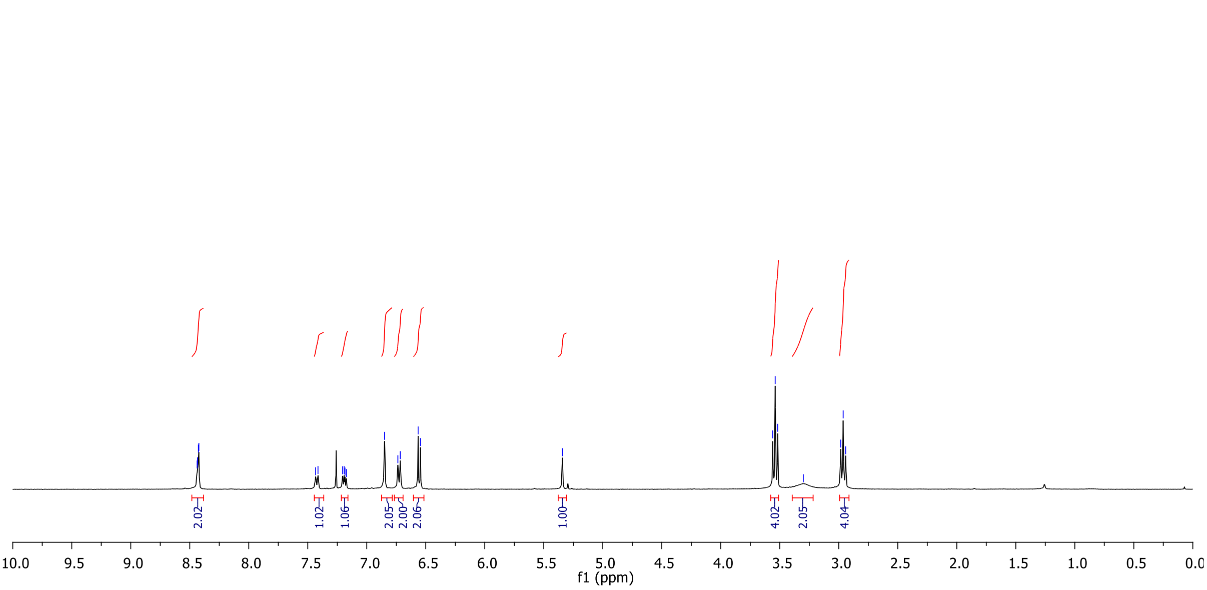

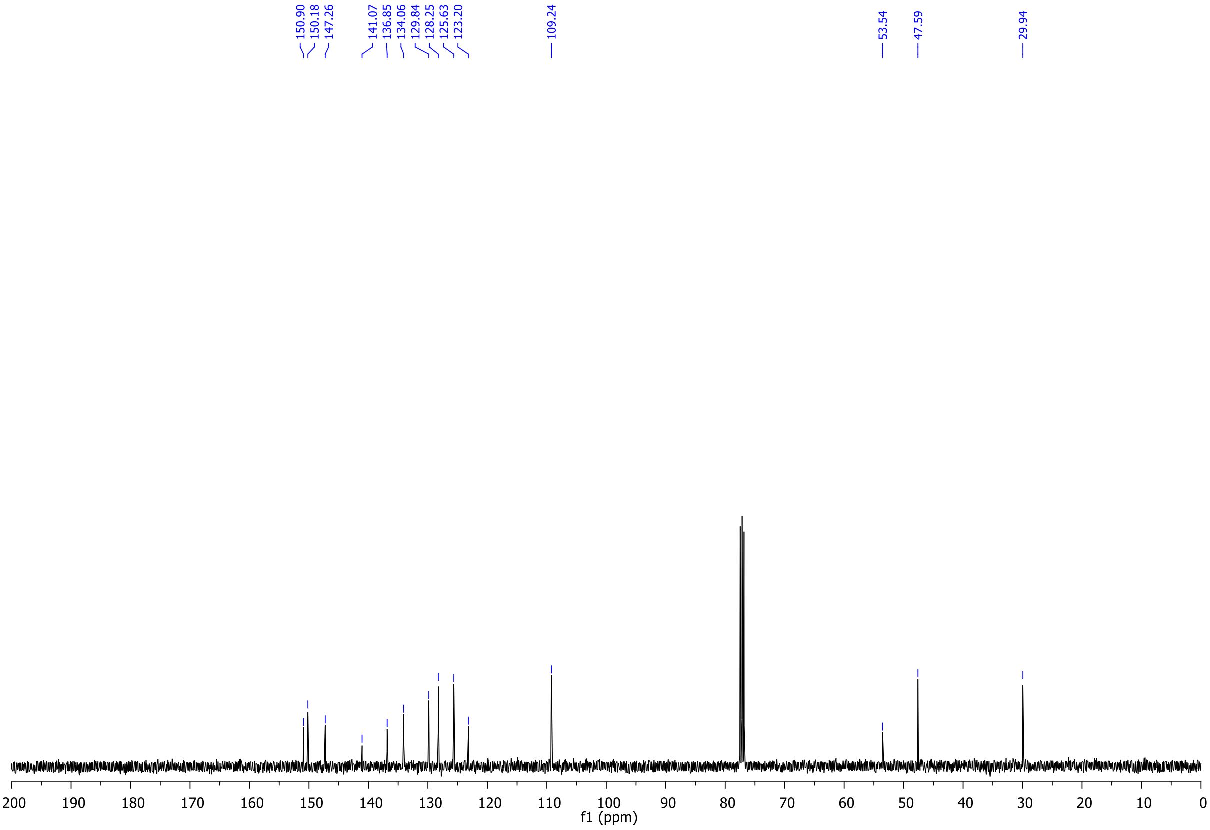

^{1}H NMR (400 MHz) and ^{13}C{^{1}H} NMR (100 MHz) spectra of **3au$_3$** ($CDCl_3$)

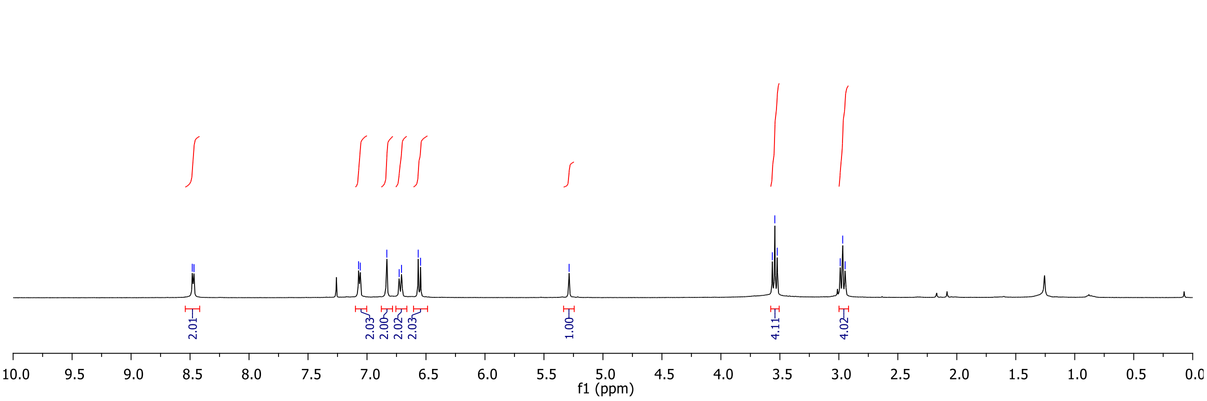

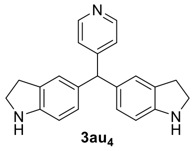

3au$_4$

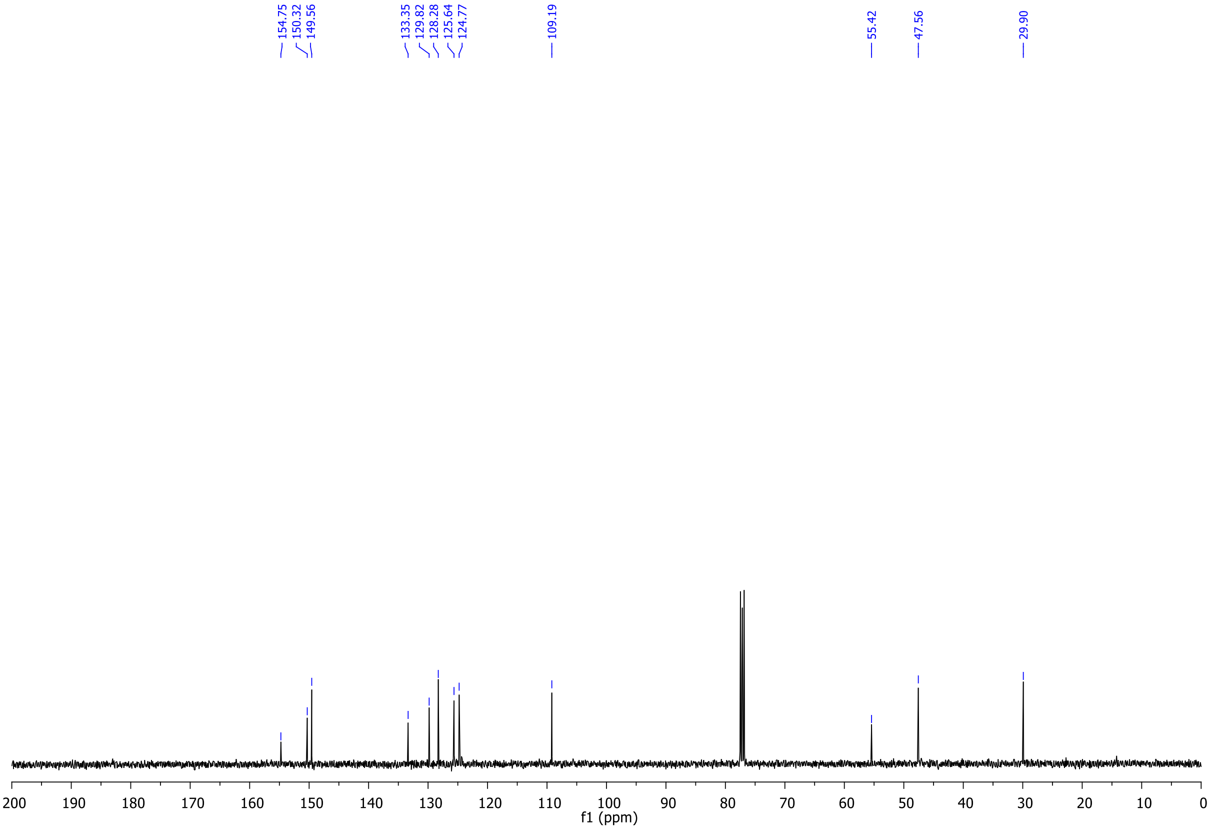

^{1}H NMR (400 MHz) and ^{13}C{^{1}H} NMR (100 MHz) spectra of **3au$_4$** ($CDCl_3$)

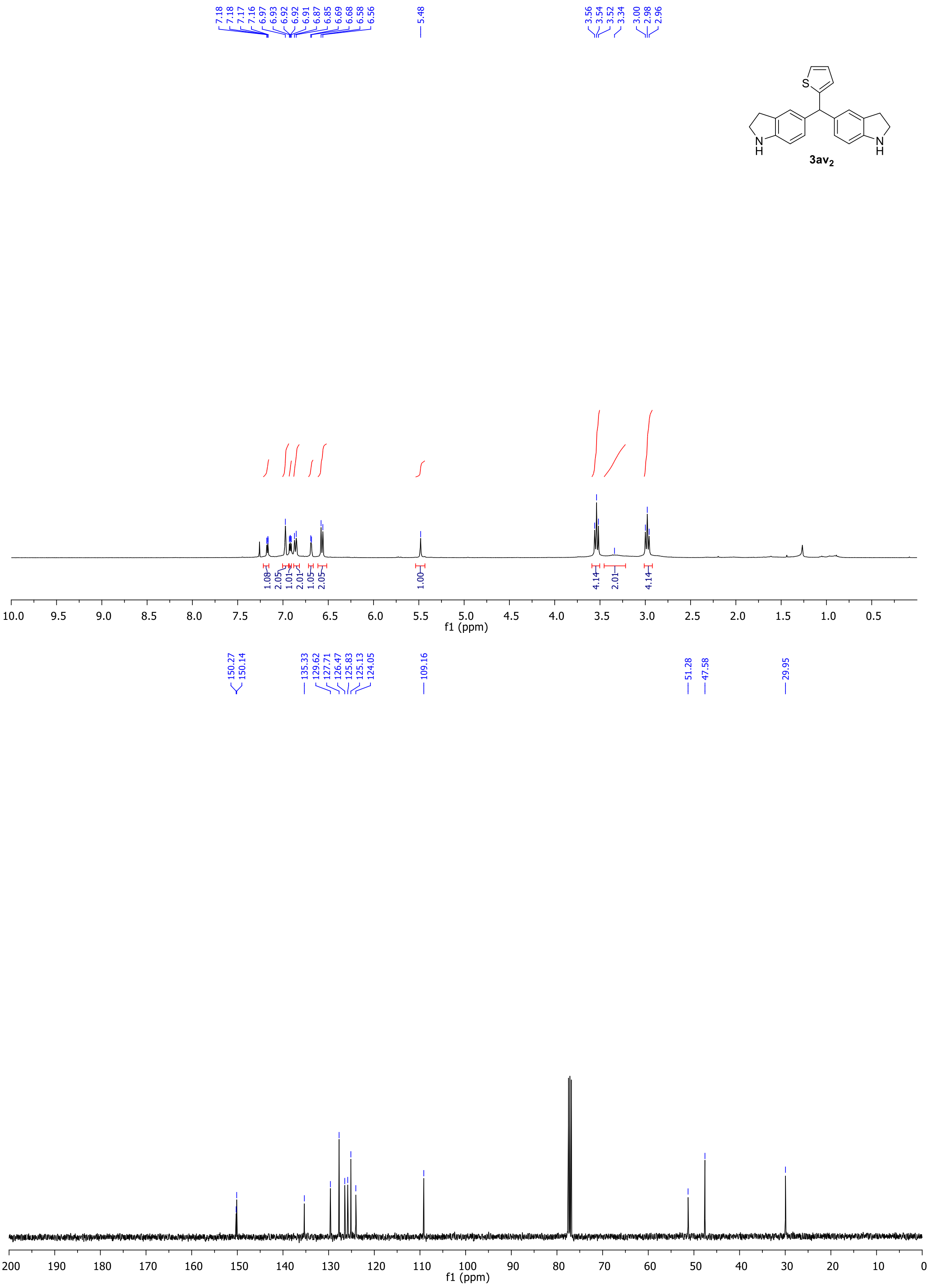

^{1}H NMR (400 MHz) and ^{13}C{^{1}H} NMR (100 MHz) spectra of **3av$_2$** ($CDCl_3$)

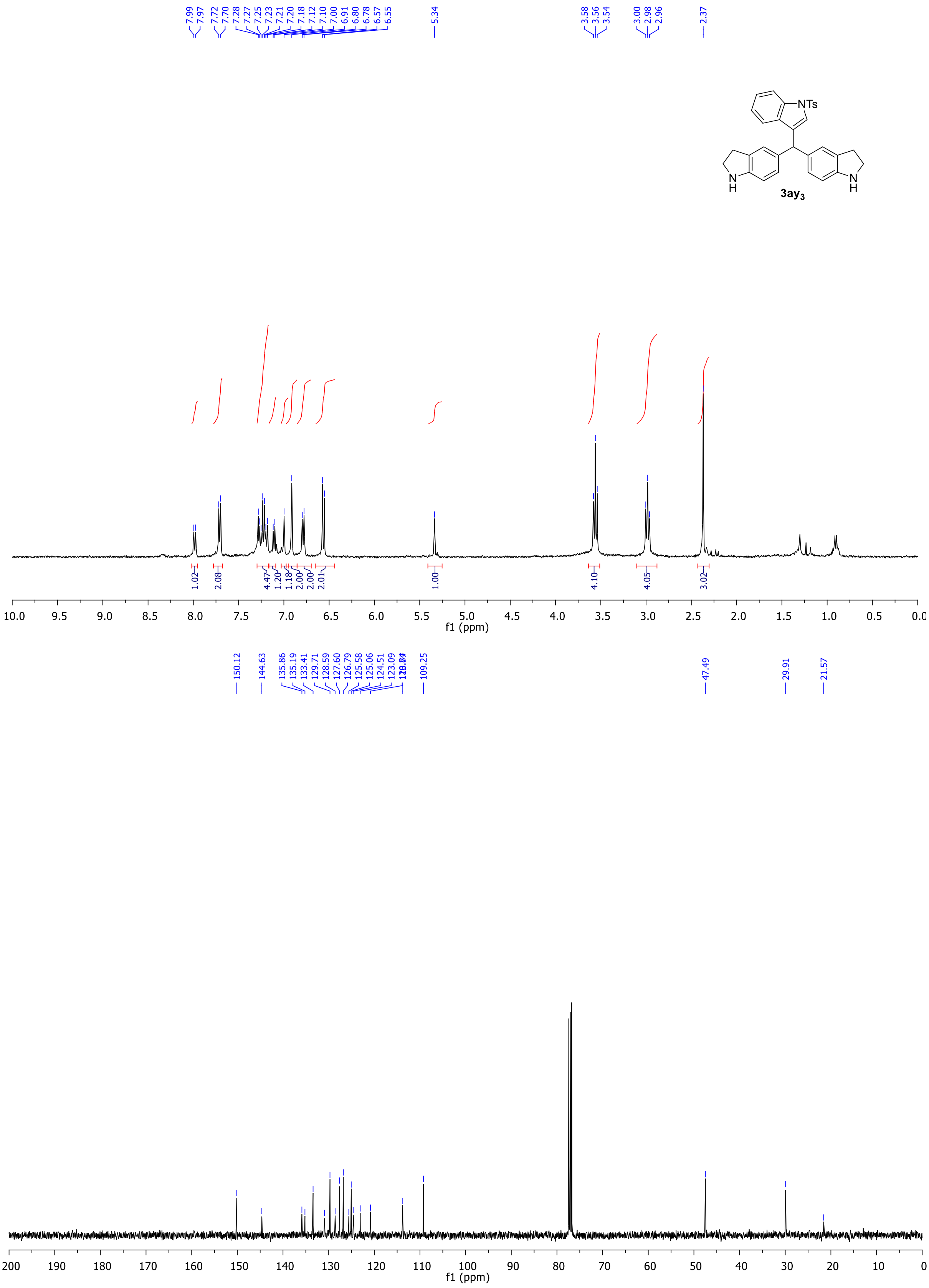

^{1}H NMR (400 MHz) and ^{13}C{^{1}H} NMR (100 MHz) spectra of **3ay$_3$** ($CDCl_3$)

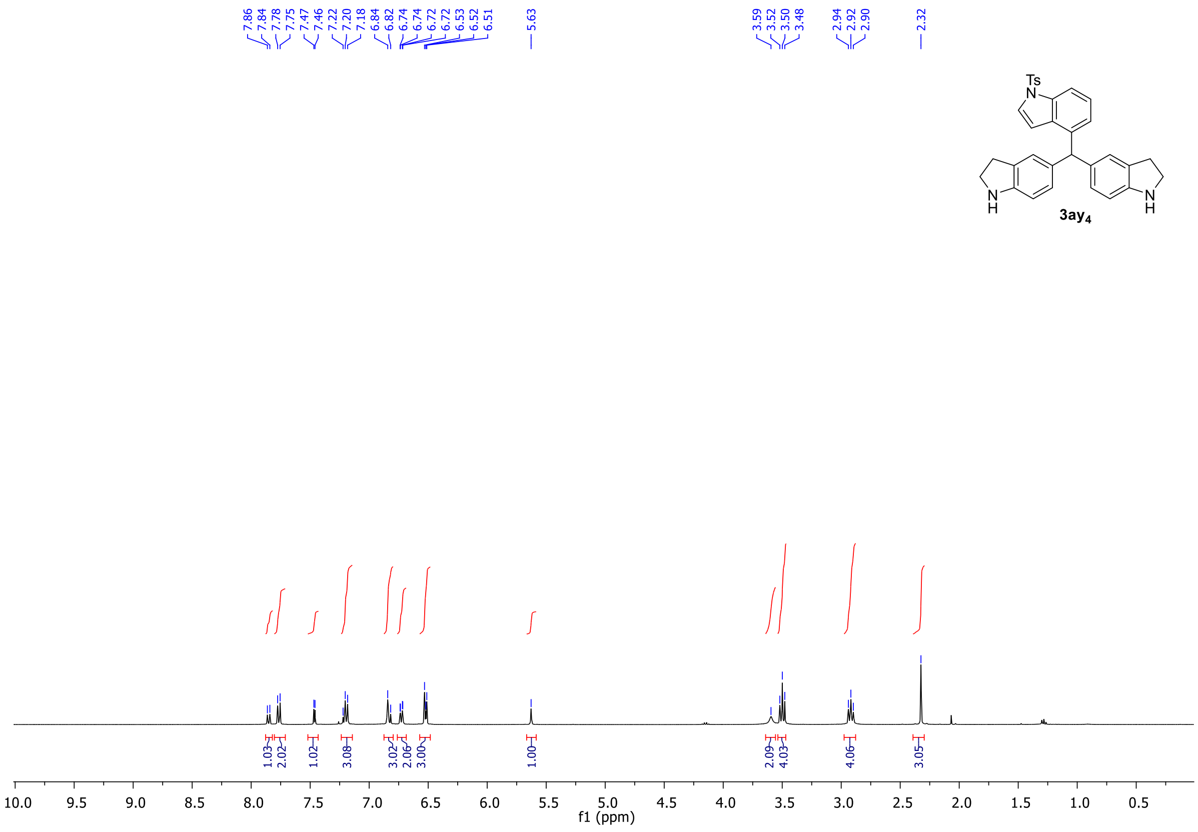

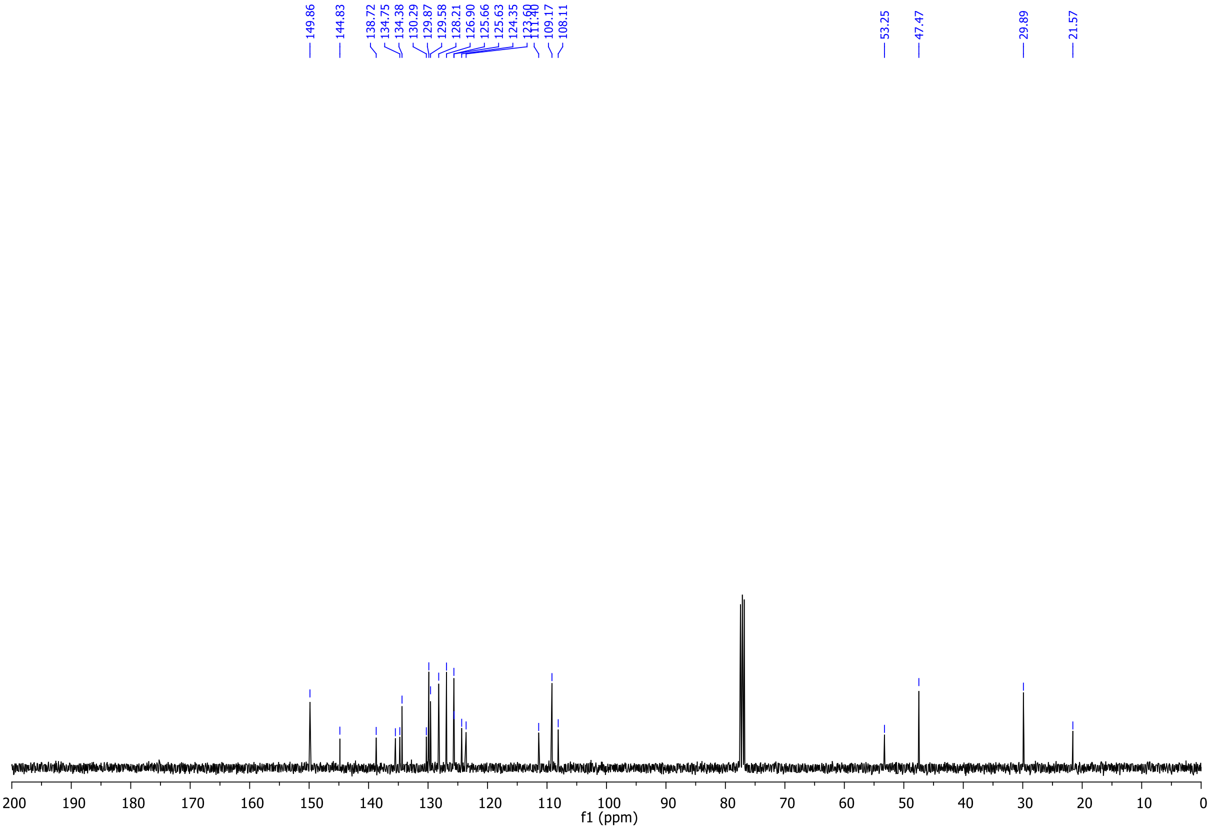

^{1}H NMR (400 MHz) and $^{13}C\{^{1}H\}$ NMR (100 MHz) spectra of **3ay$_4$** ($CDCl_3$)

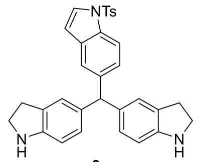

3ay$_5$

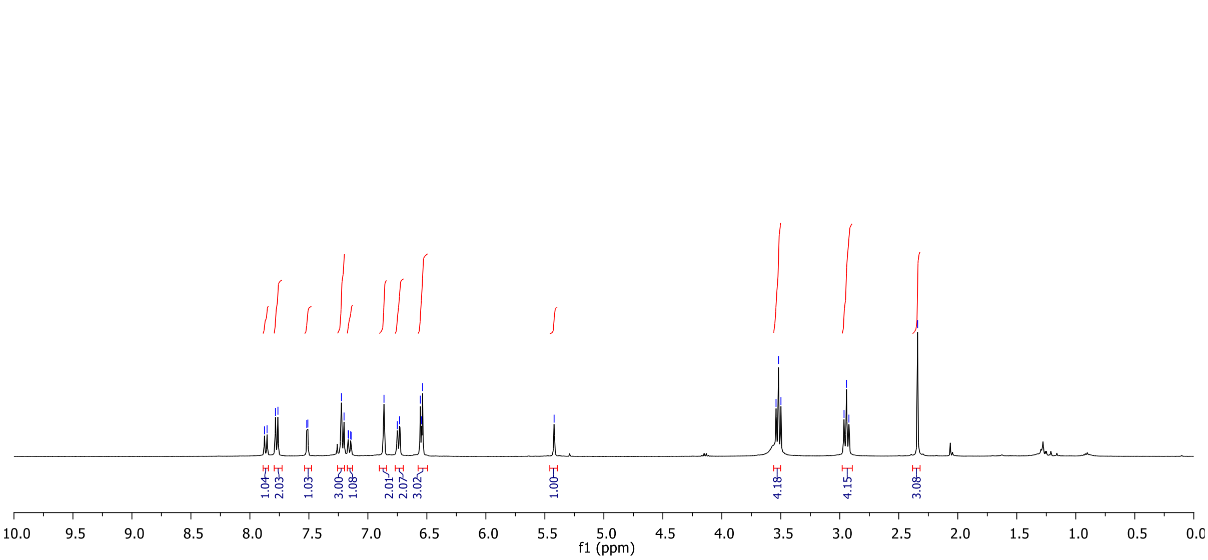

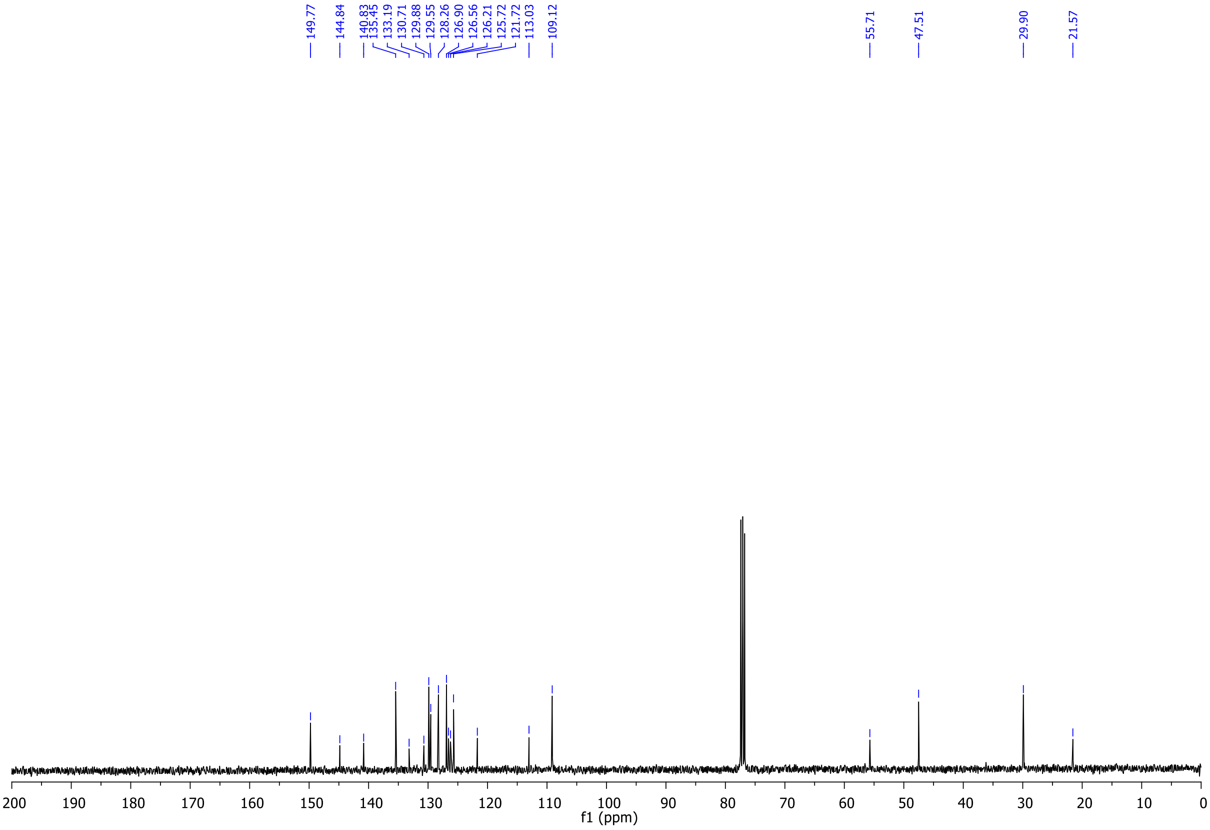

^{1}H NMR (400 MHz) and ^{13}C{^{1}H} NMR (100 MHz) spectra of **3ay$_5$** ($CDCl_3$)

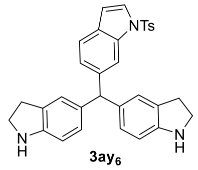

3ay$_6$

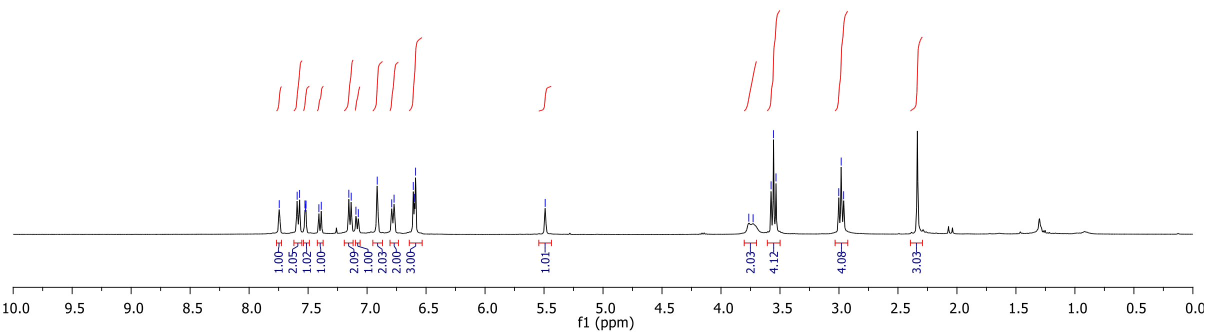

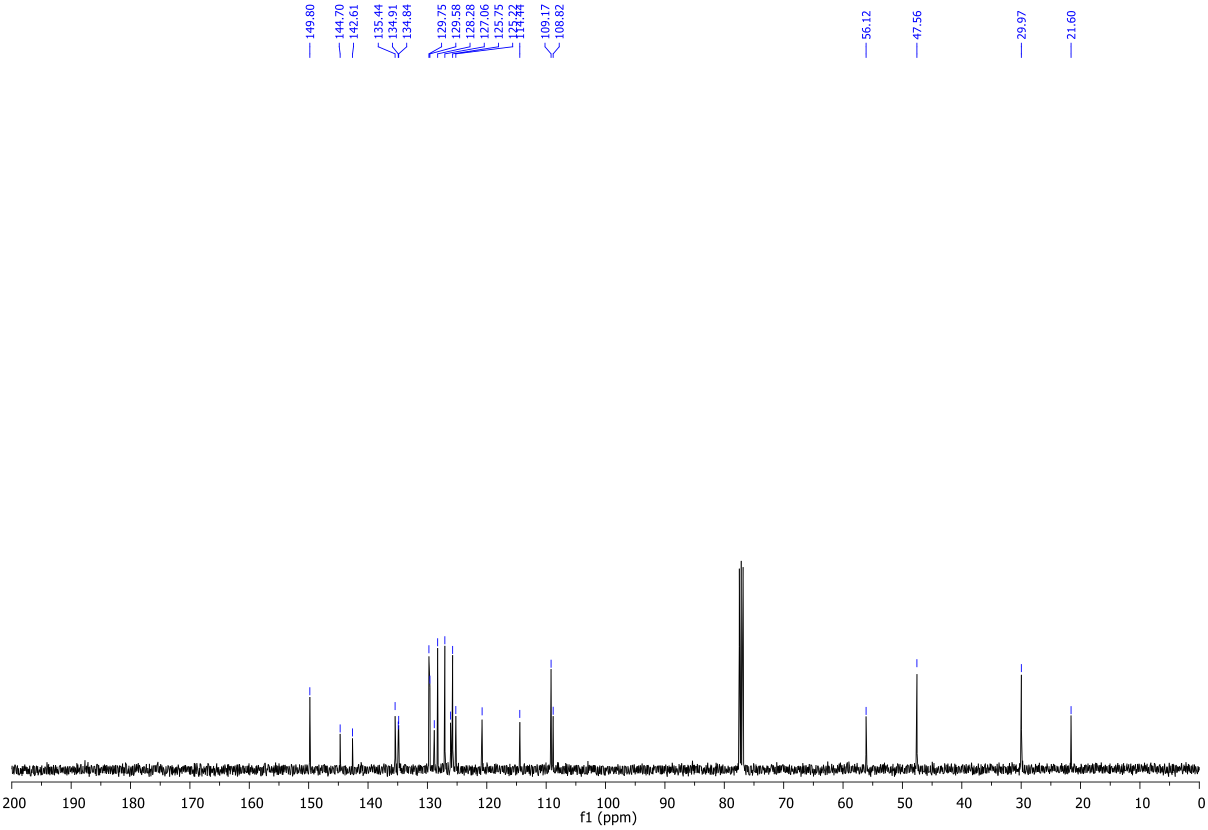

^{1}H NMR (400 MHz) and $^{13}C\{^{1}H\}$ NMR (100 MHz) spectra of **3ay$_6$** ($CDCl_3$)

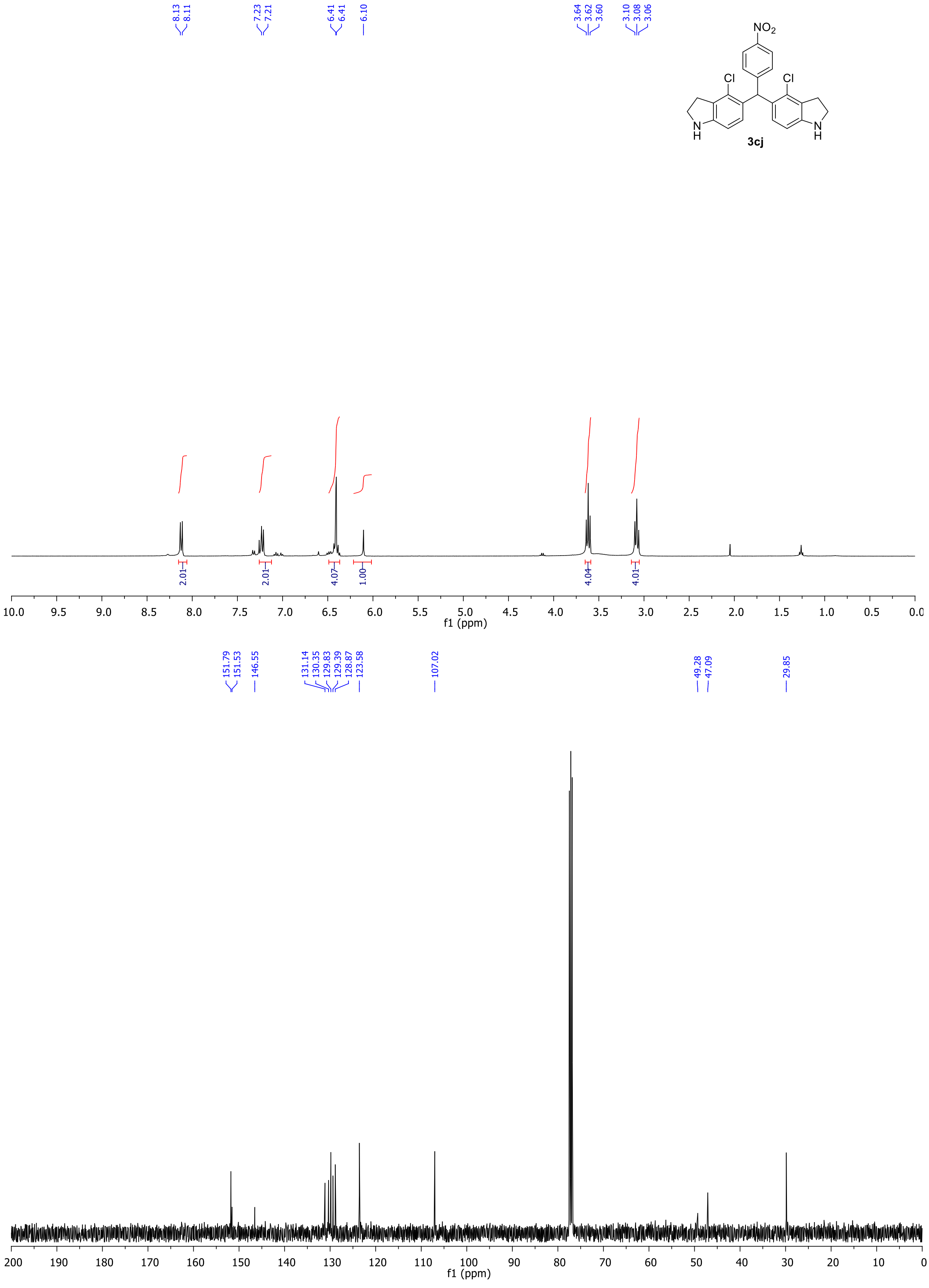

^{1}H NMR (400 MHz) and ^{13}C{^{1}H} NMR (100 MHz) spectra of **3cj** ($CDCl_3$)

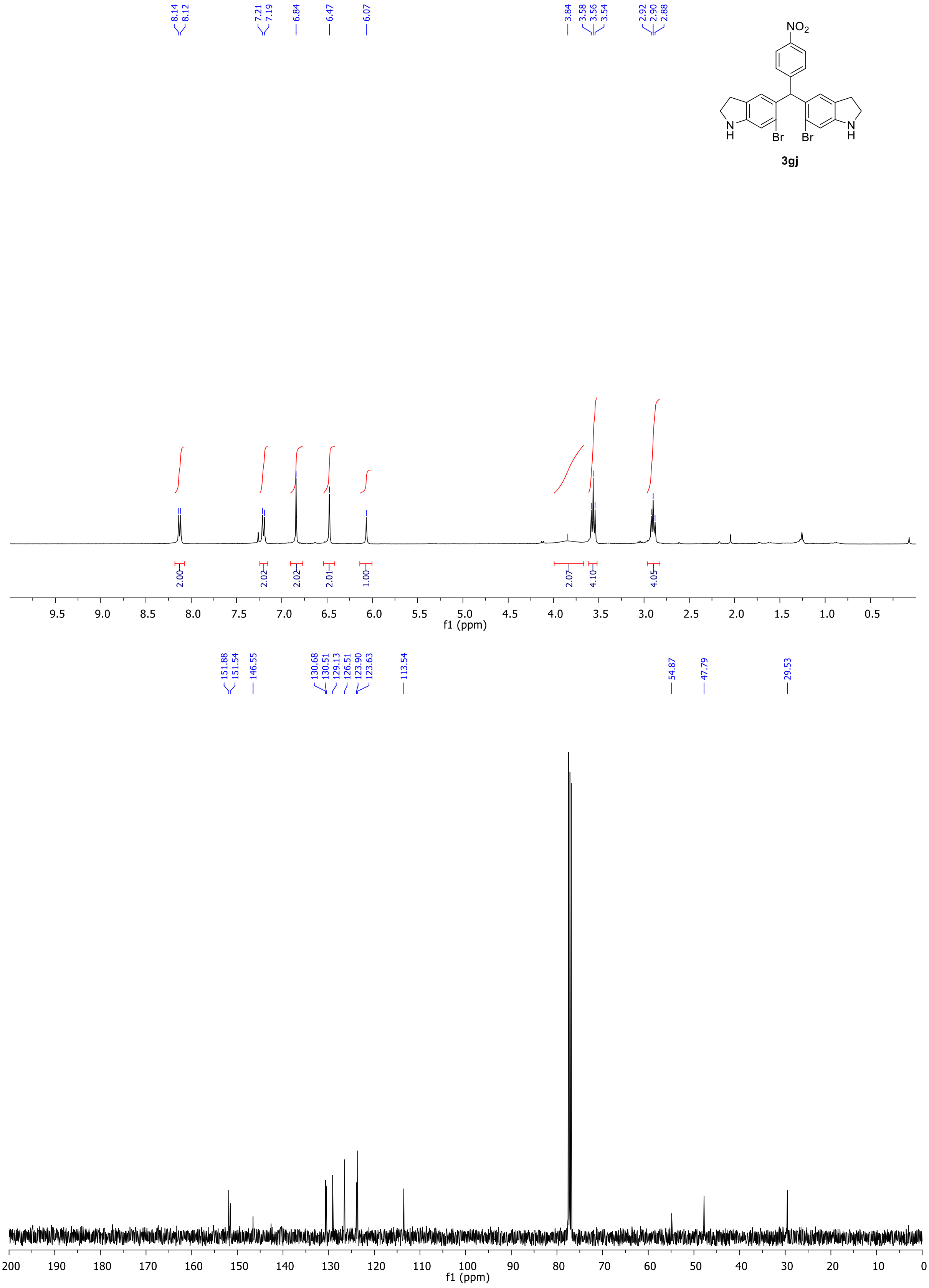

^{1}H NMR (400 MHz) and $^{13}C\{^{1}H\}$ NMR (100 MHz) spectra of **3gj** ($CDCl_3$)

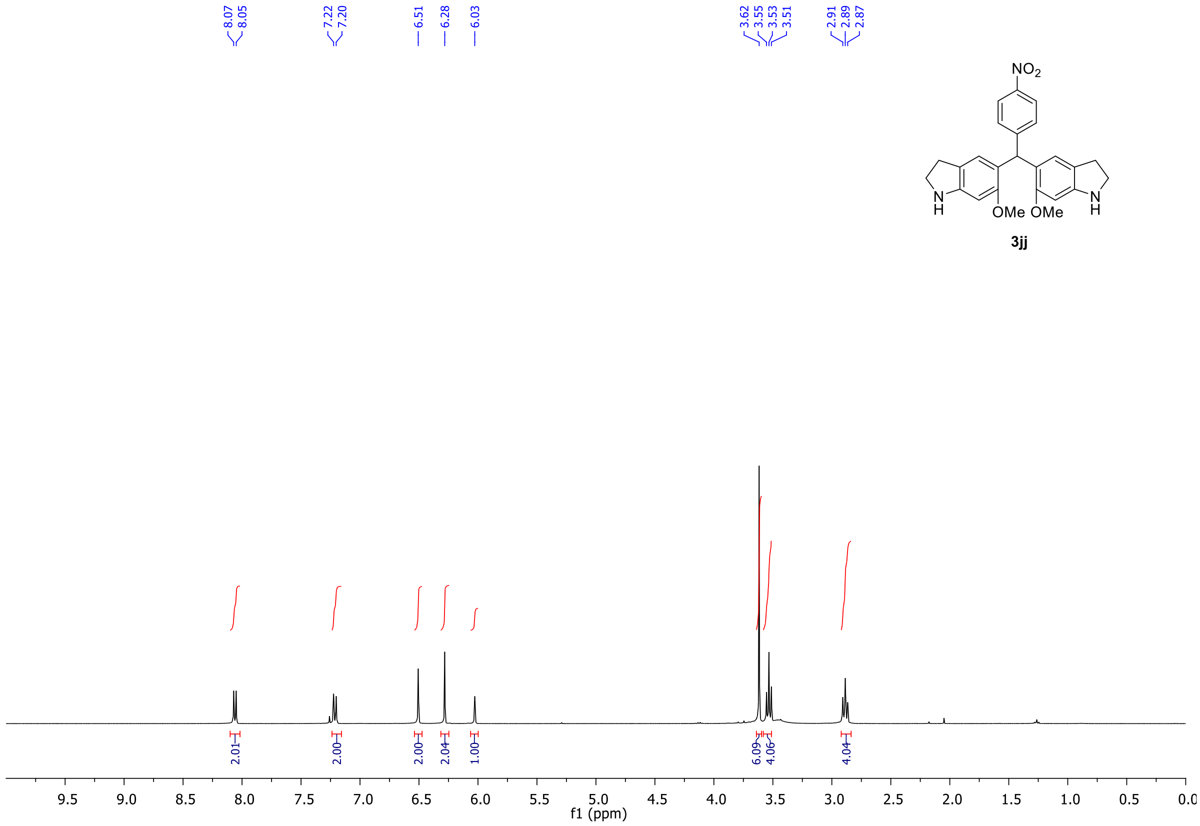

3jj

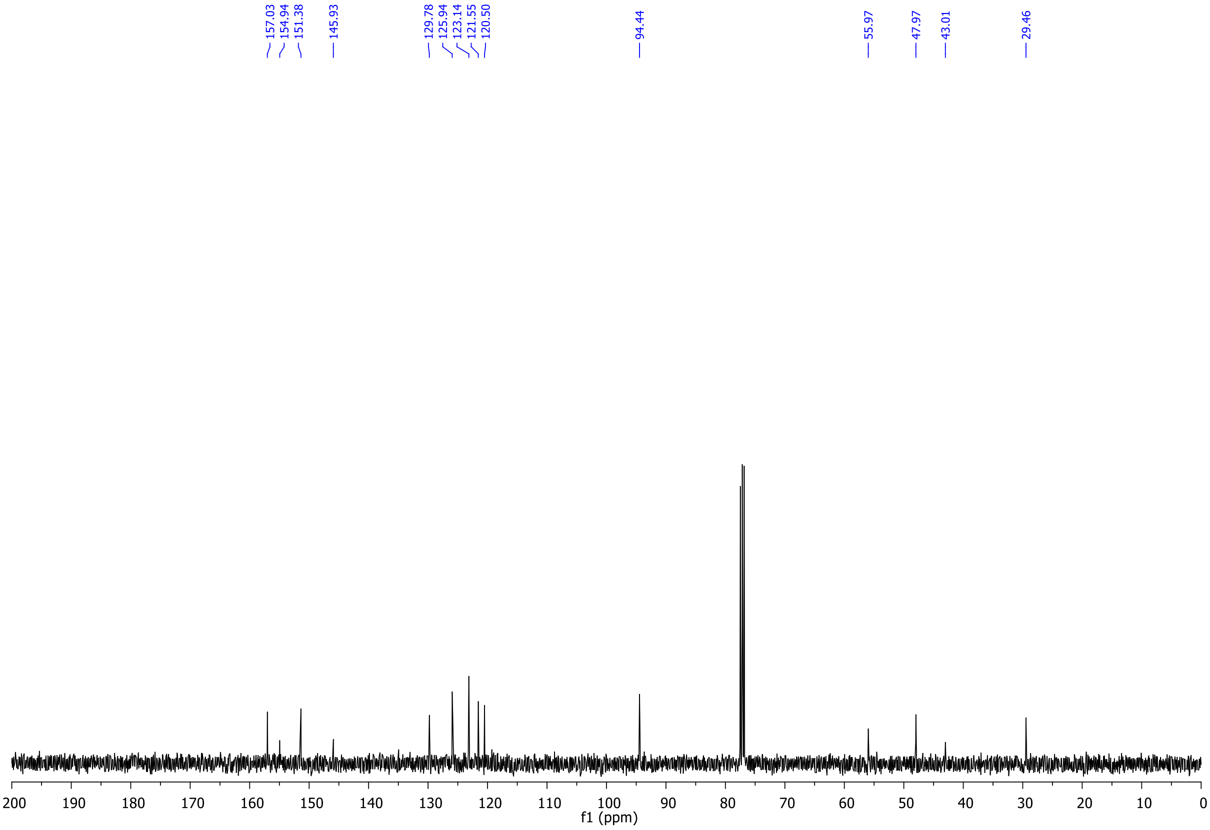

^{1}H NMR (400 MHz) and ^{13}C{^{1}H} NMR (100 MHz) spectra of **3jj** ($CDCl_3$)

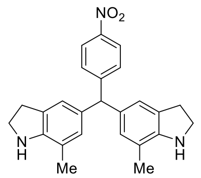

3nj

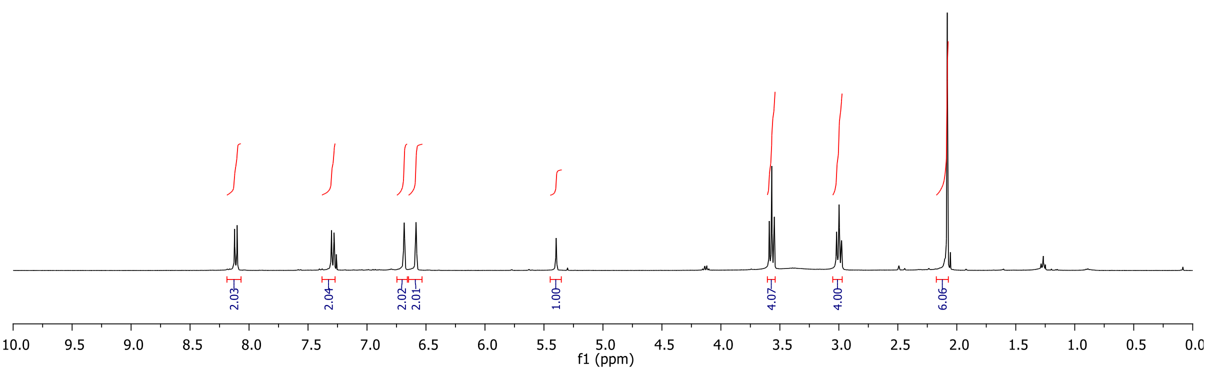

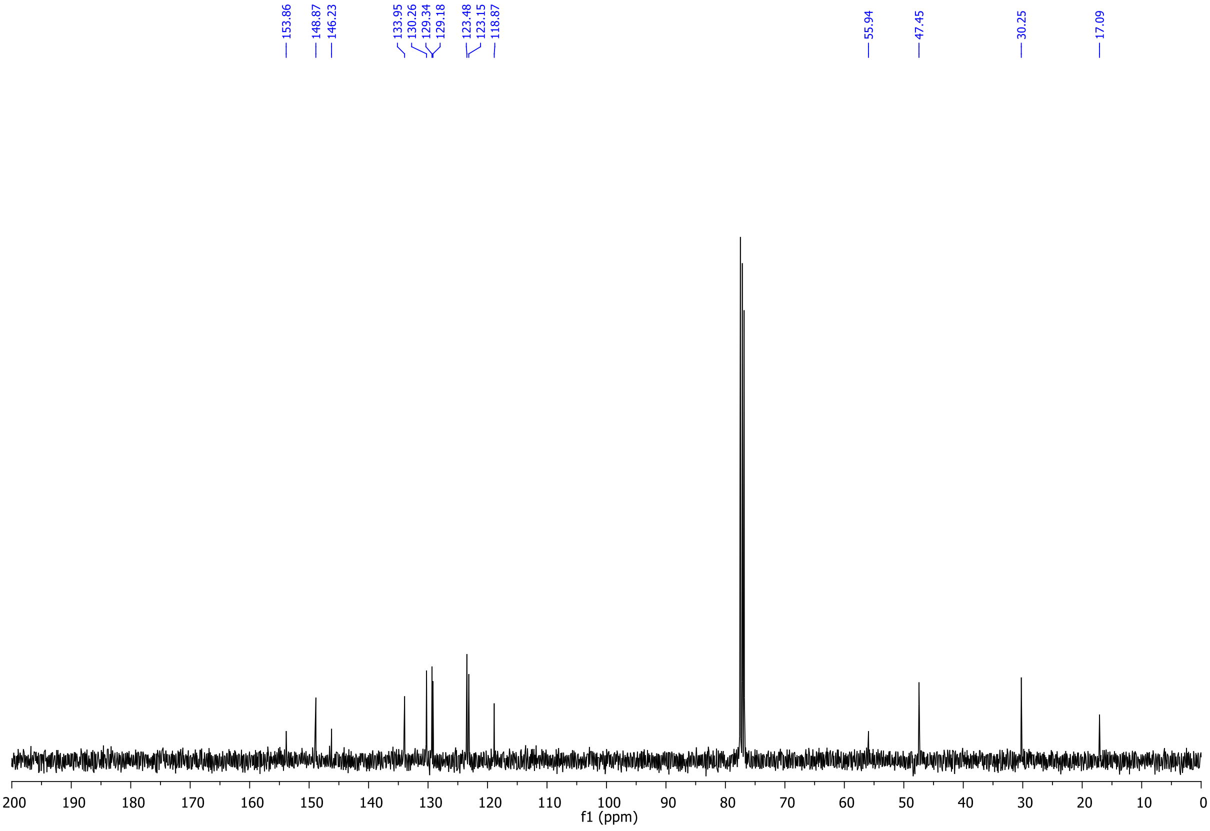

^{1}H NMR (400 MHz) and ^{13}C{^{1}H} NMR (100 MHz) spectra of **3nj** ($CDCl_3$)

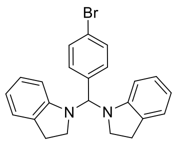

4af

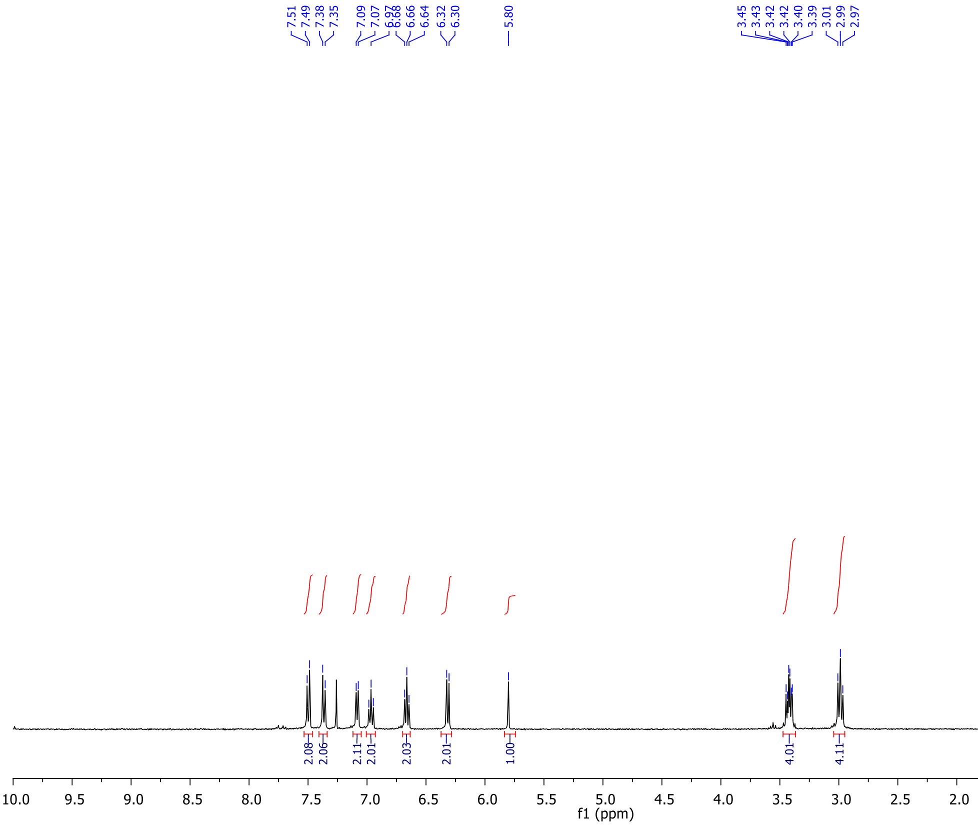

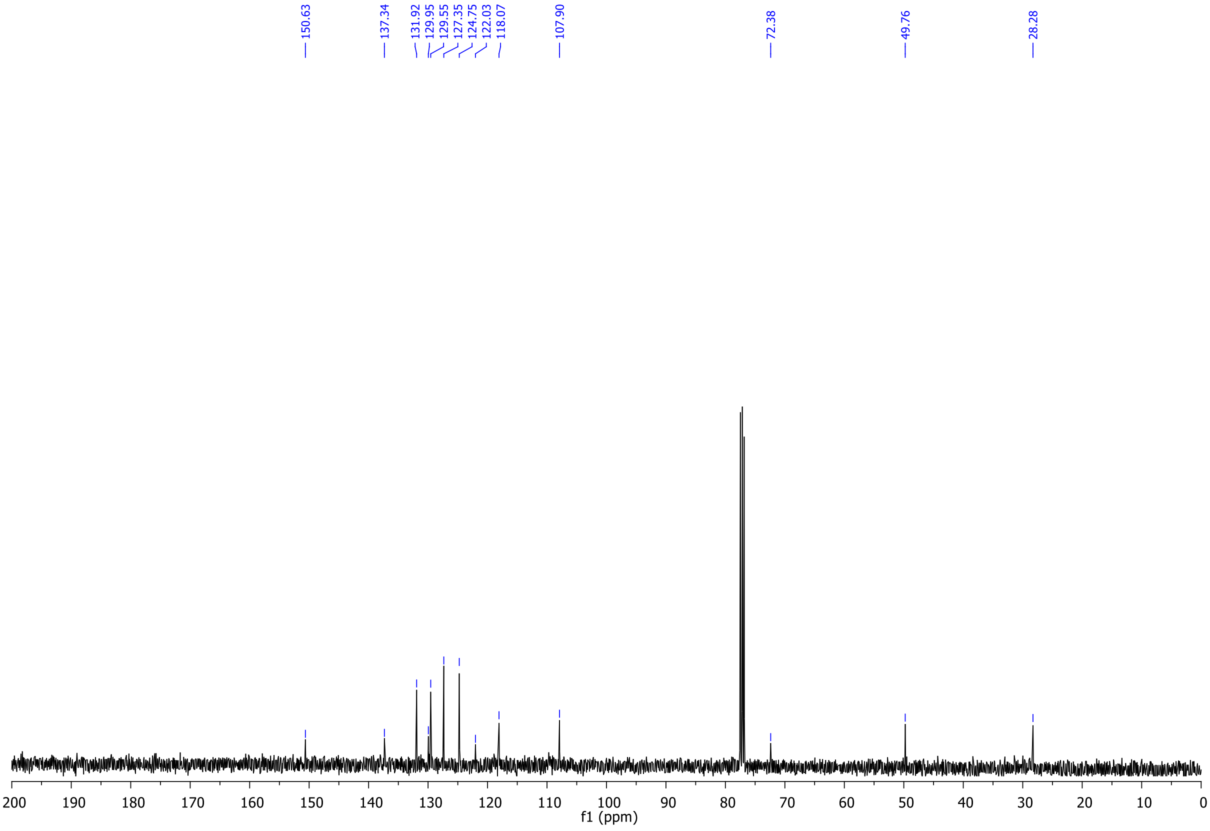

^{1}H NMR (400 MHz) and ^{13}C{^{1}H} NMR (100 MHz) spectra of **4af** ($CDCl_3$)

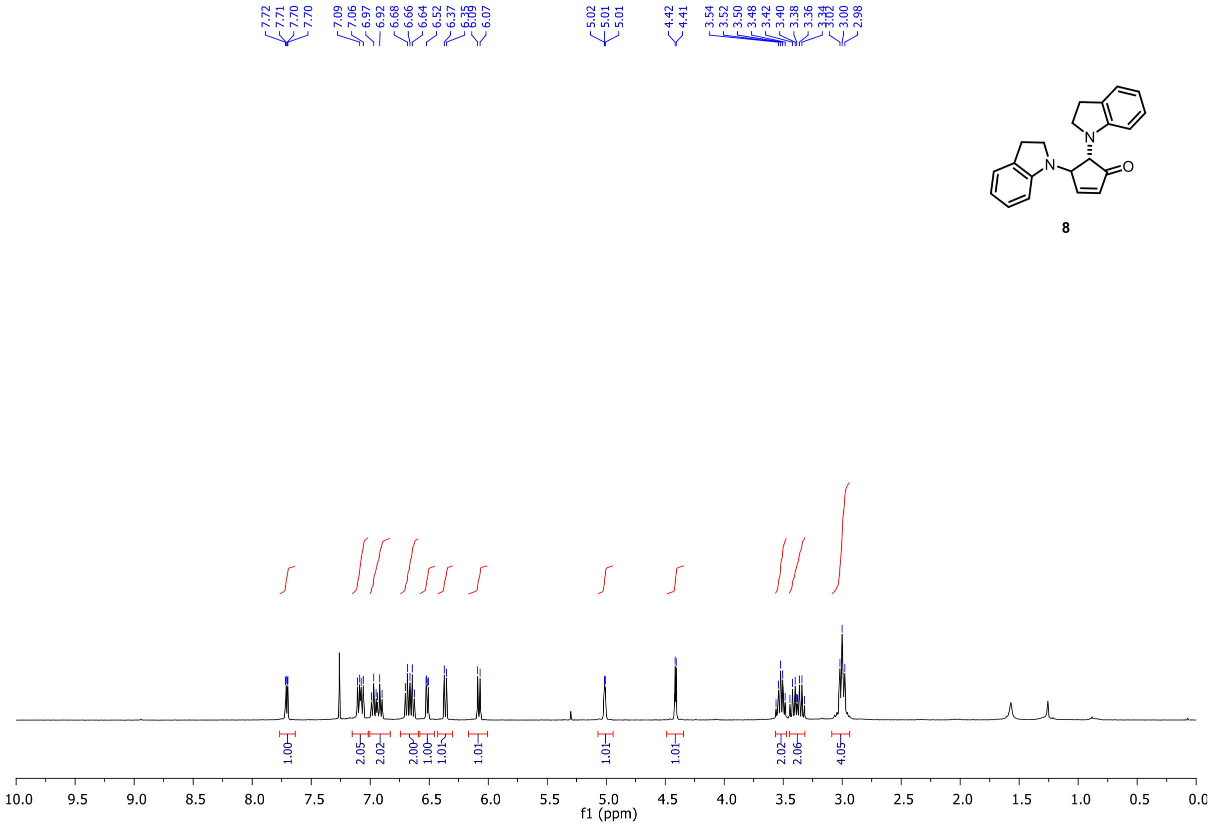

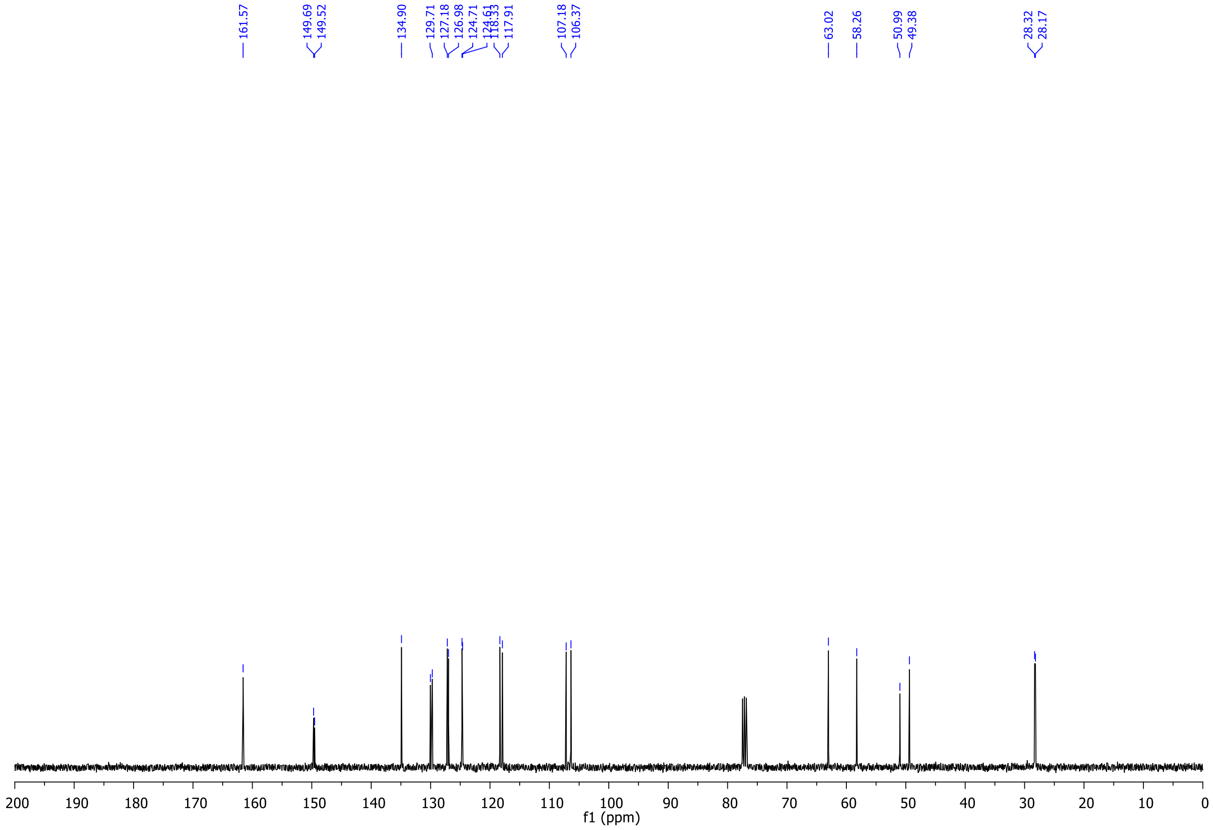

^{1}H NMR (400 MHz) and $^{13}C\{^{1}H\}$ NMR (100 MHz) spectra of **8** ($CDCl_3$)

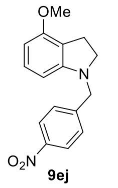

9ej

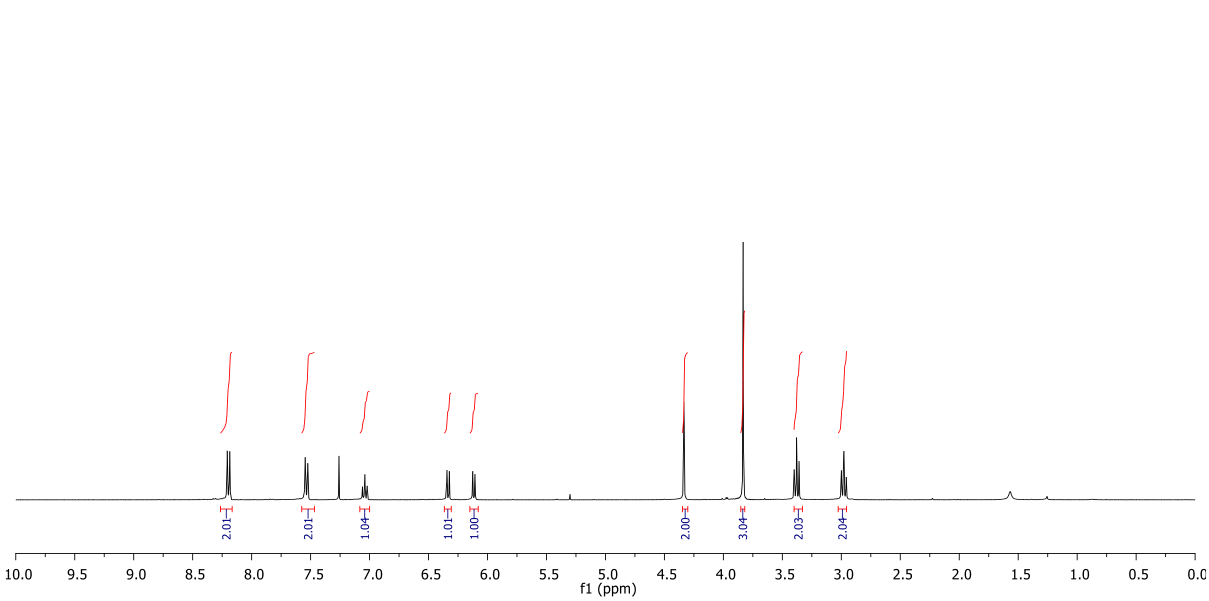

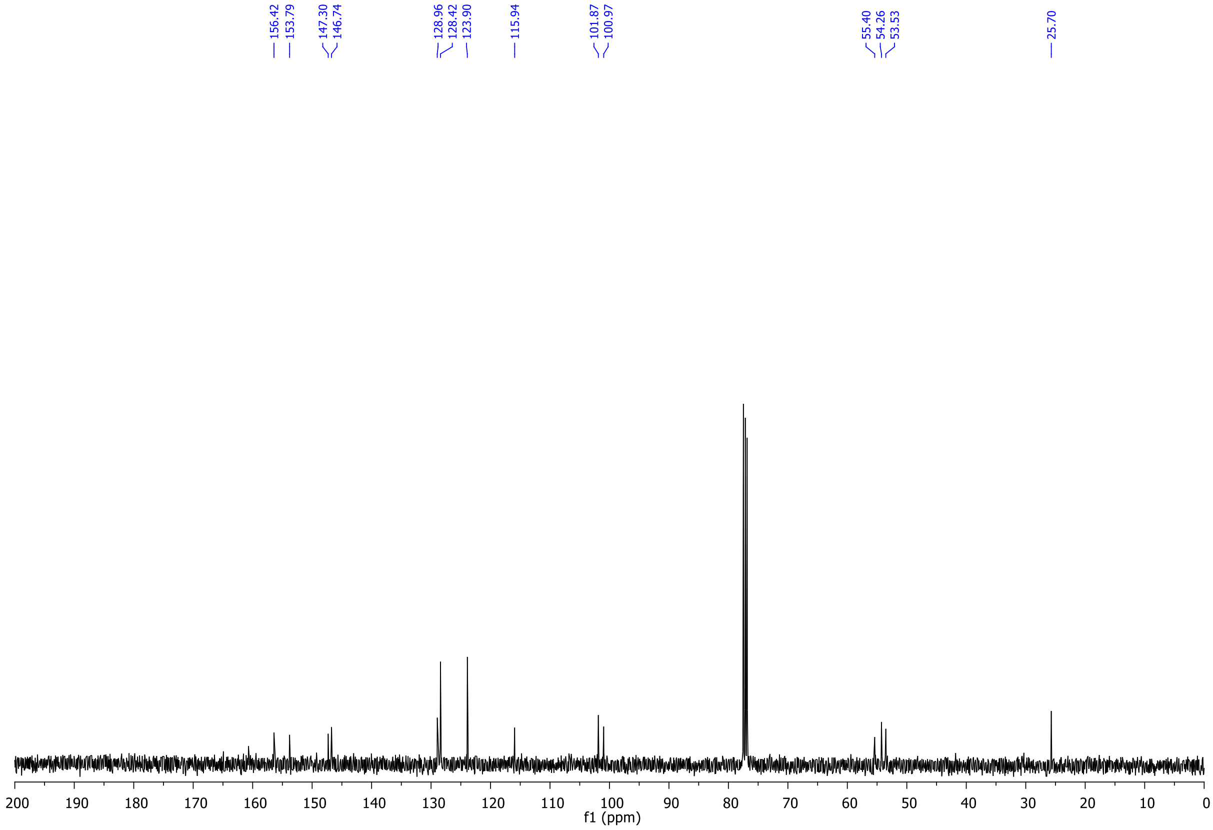

^{1}H NMR (400 MHz) and $^{13}C\{^{1}H\}$ NMR (100 MHz) spectra of **9ej** ($CDCl_3$)

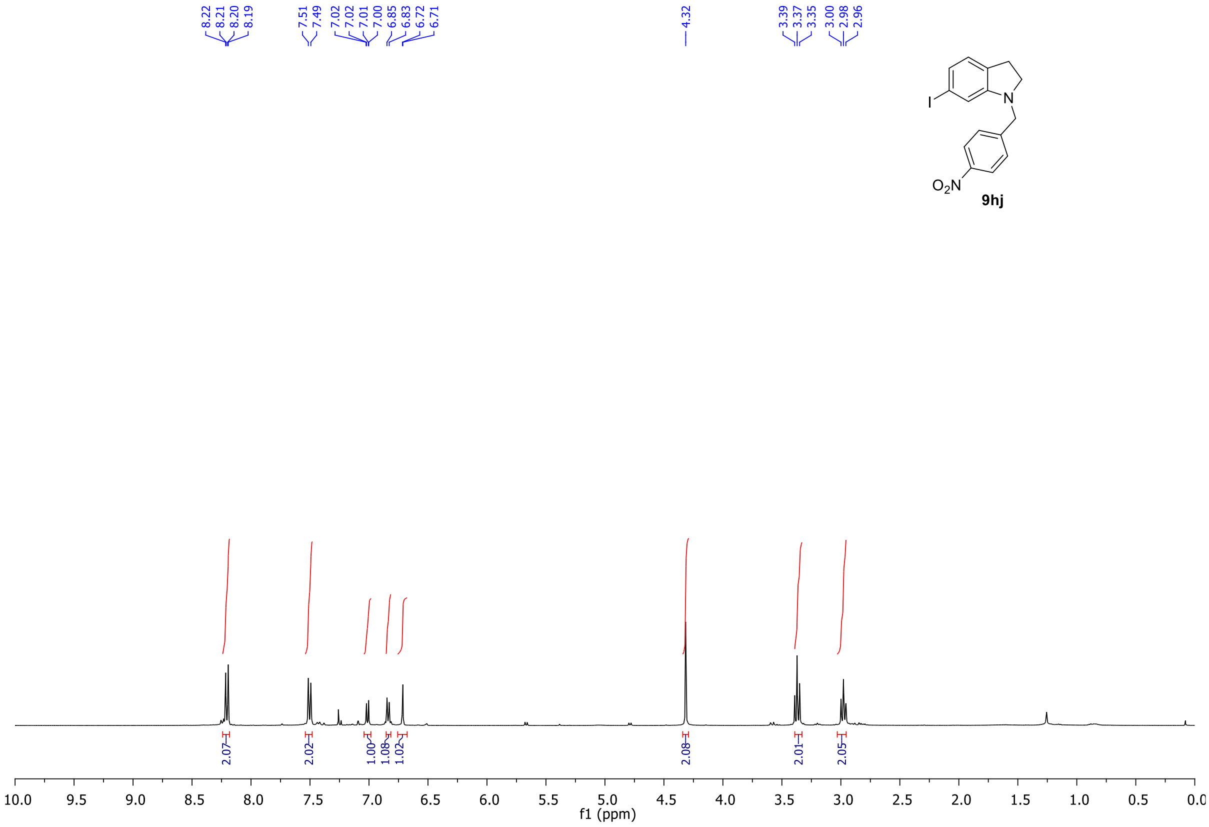

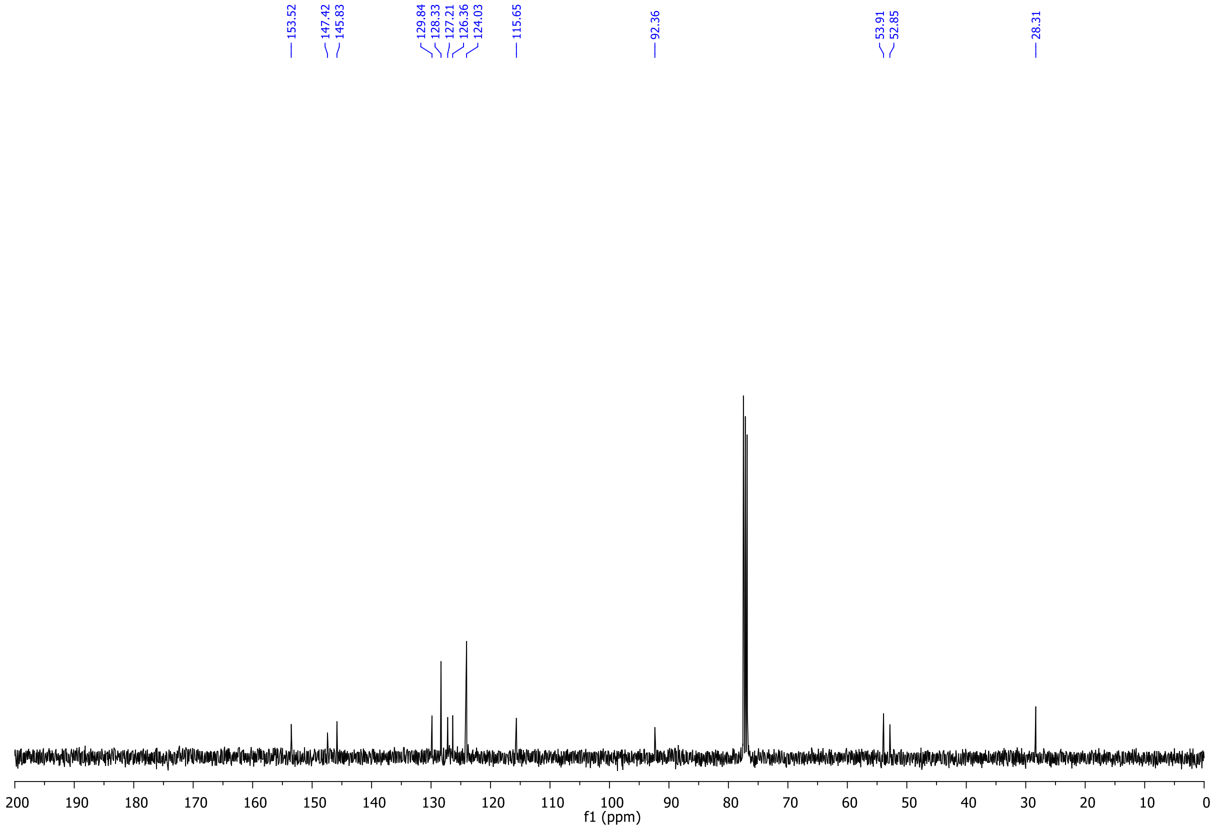

^{1}H NMR (400 MHz) and ^{13}C{^{1}H} NMR (100 MHz) spectra of **9hj** ($CDCl_3$)

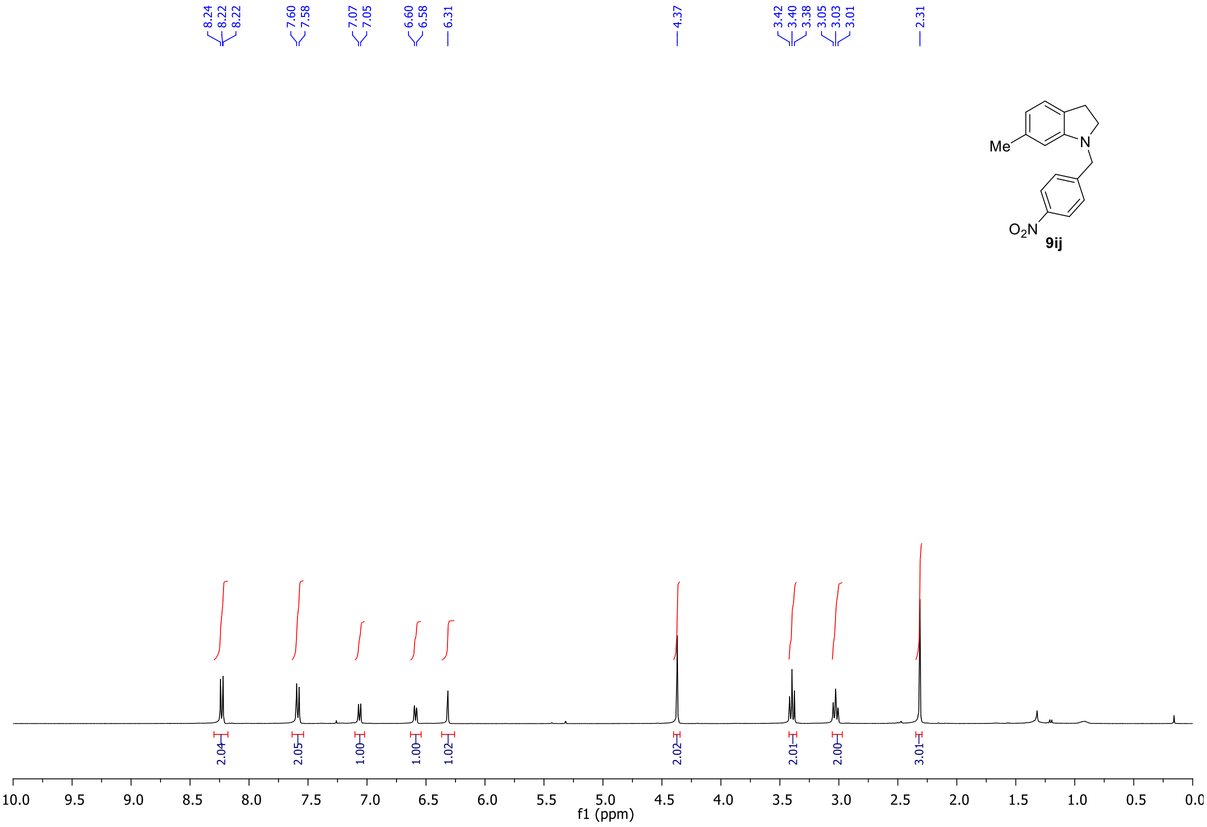

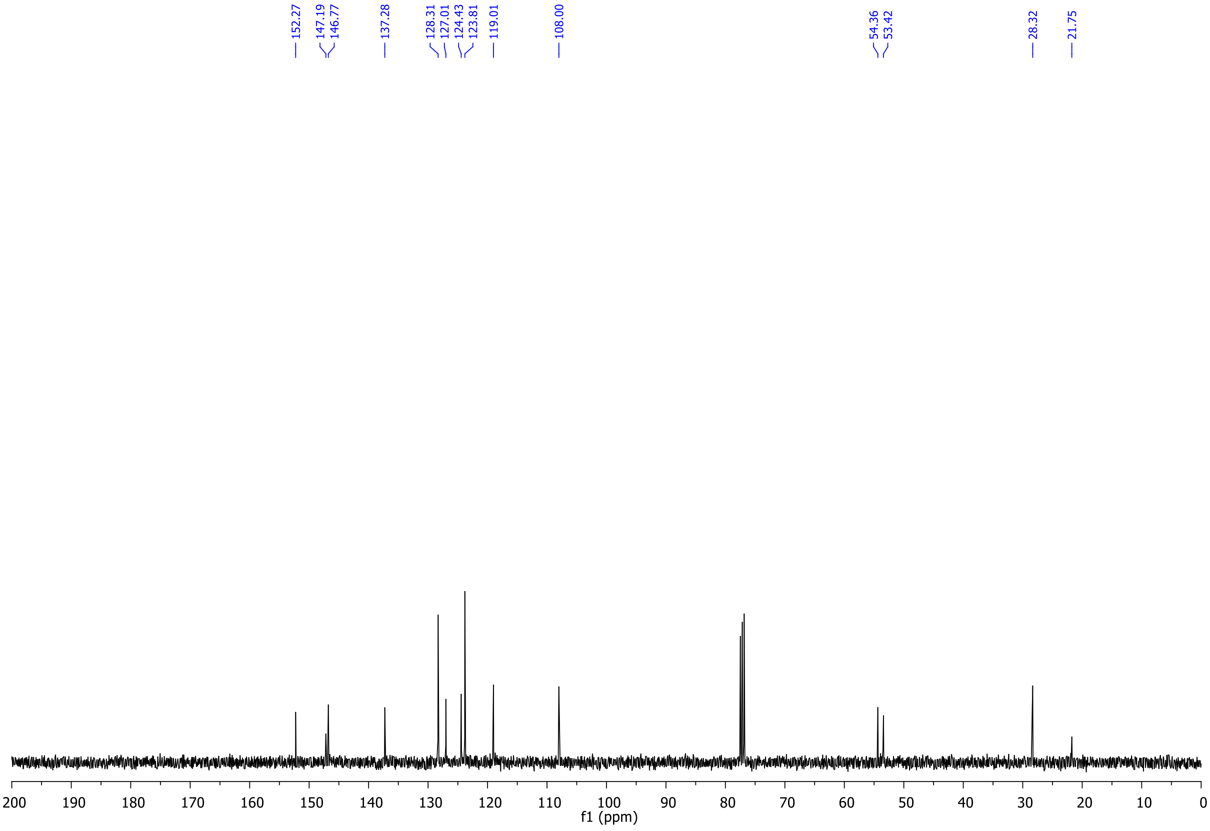

^{1}H NMR (400 MHz) and ^{13}C{^{1}H} NMR (100 MHz) spectra of **9ij** ($CDCl_3$)

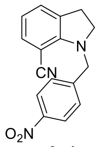

9mj

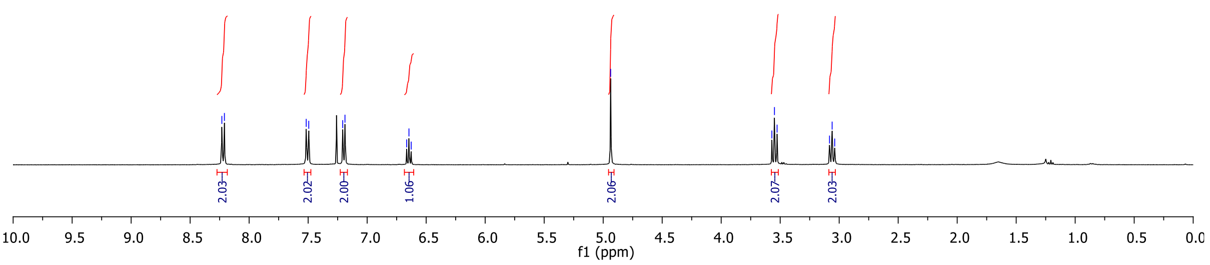

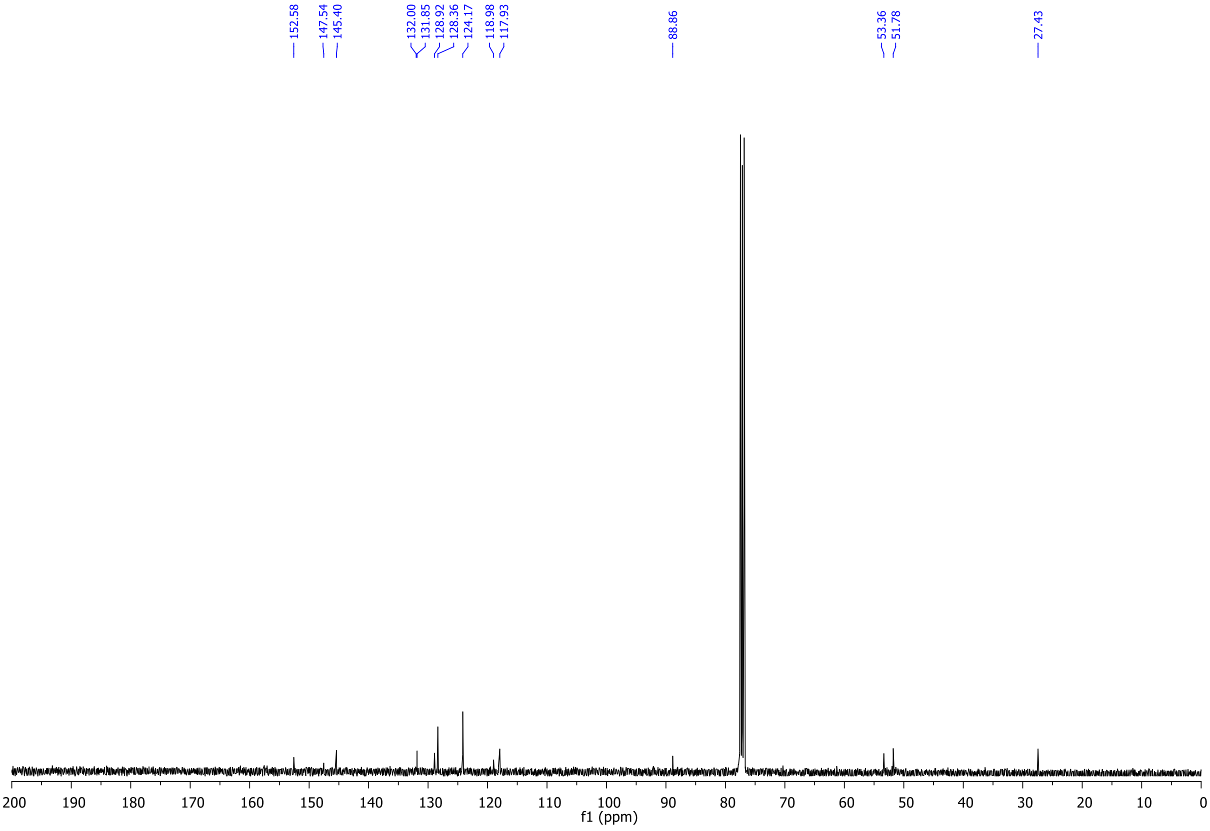

^{1}H NMR (400 MHz) and ^{13}C{^{1}H} NMR (100 MHz) spectra of **9mj** (CDCl$_3$)

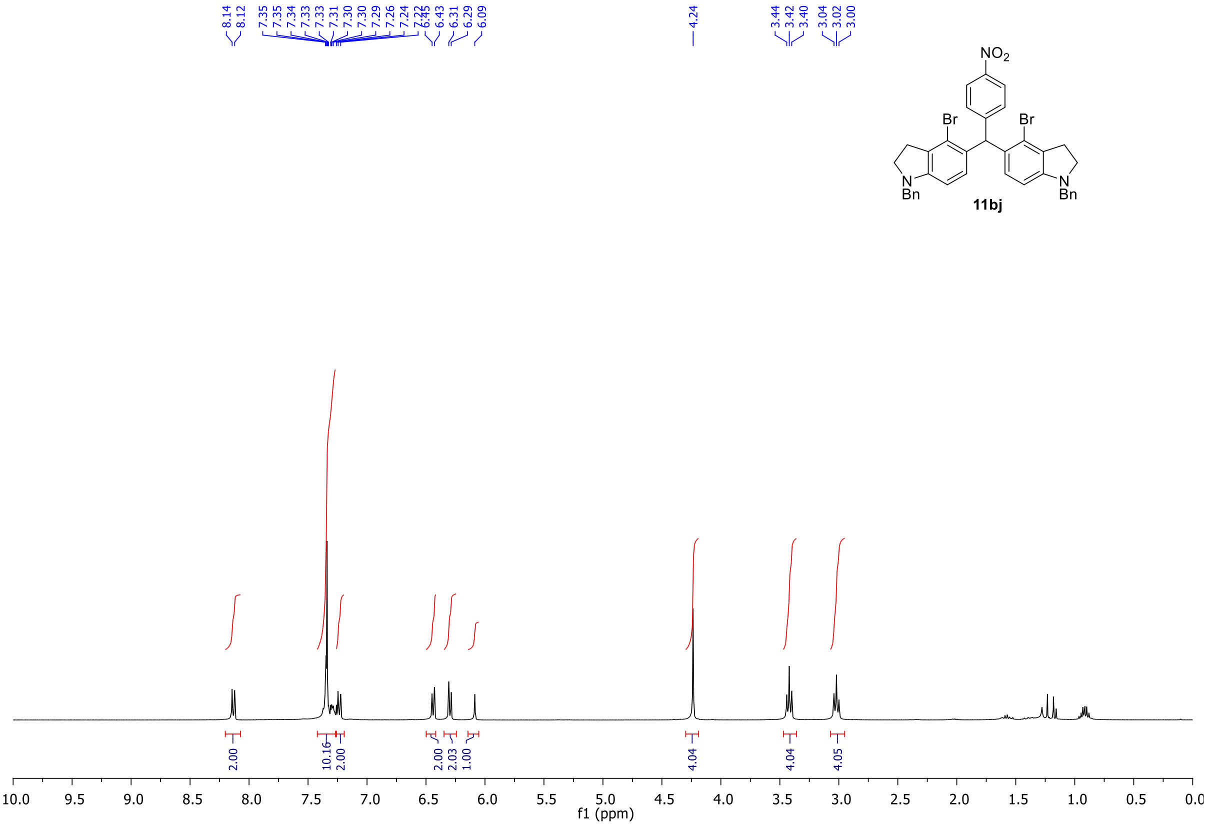

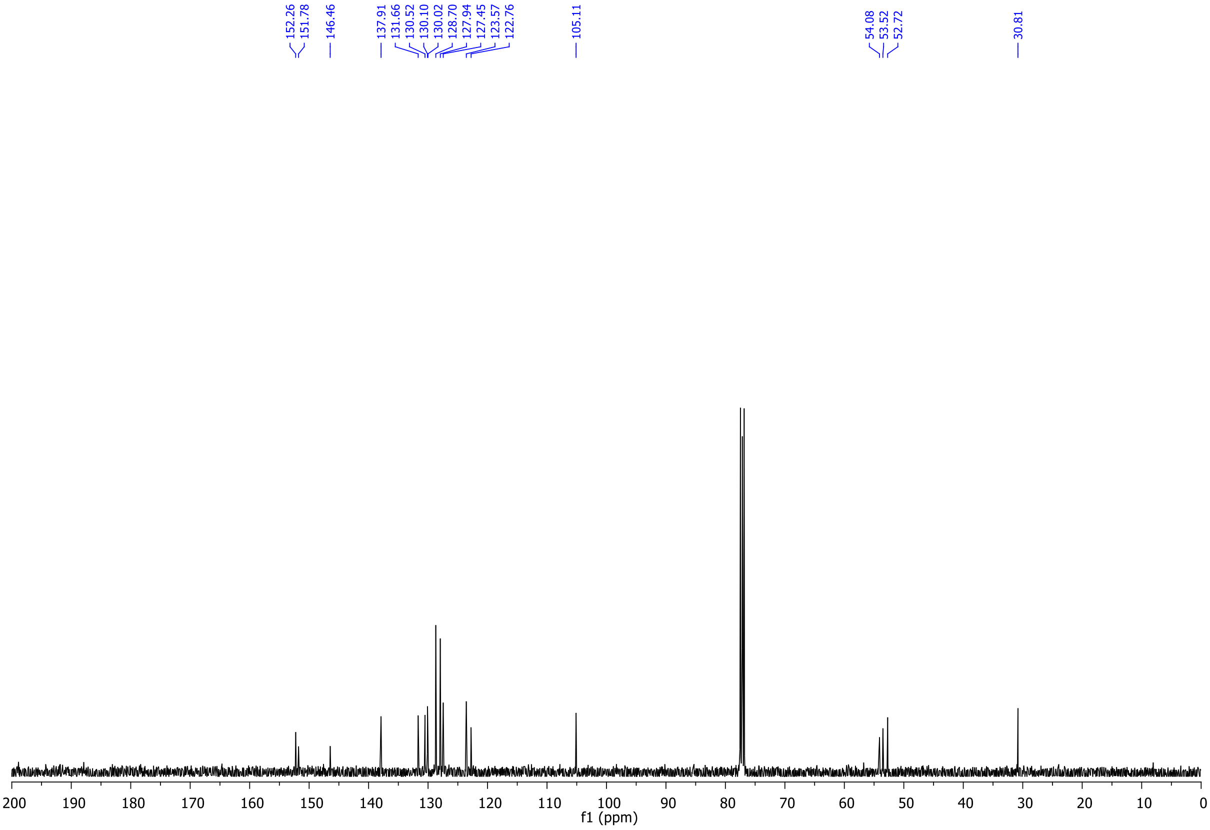

^{1}H NMR (400 MHz) and $^{13}C\{^{1}H\}$ NMR (100 MHz) spectra of **11bj** ($CDCl_3$)

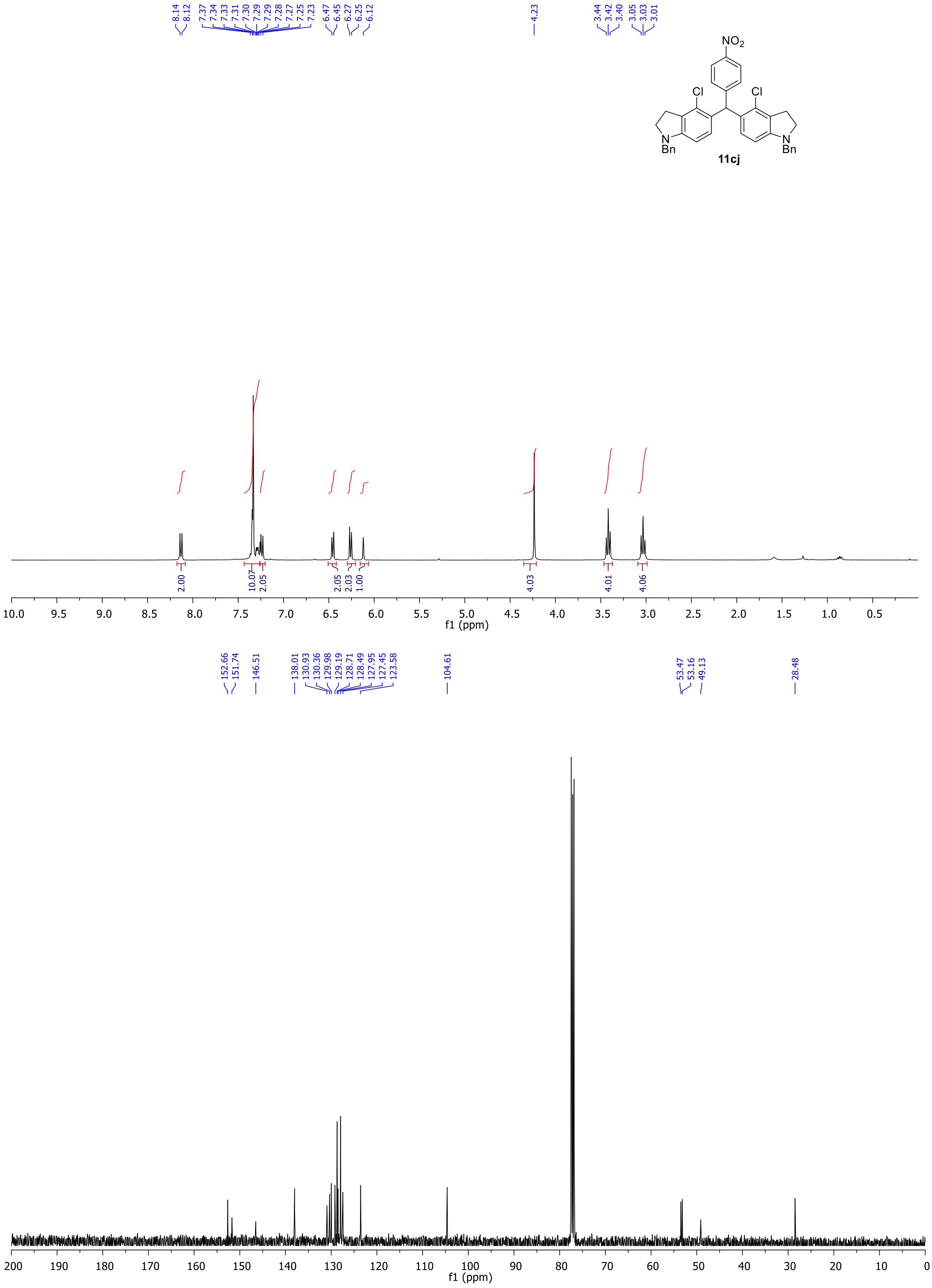

^{1}H NMR (400 MHz) and ^{13}C{^{1}H} NMR (100 MHz) spectra of **11cj** ($CDCl_3$)

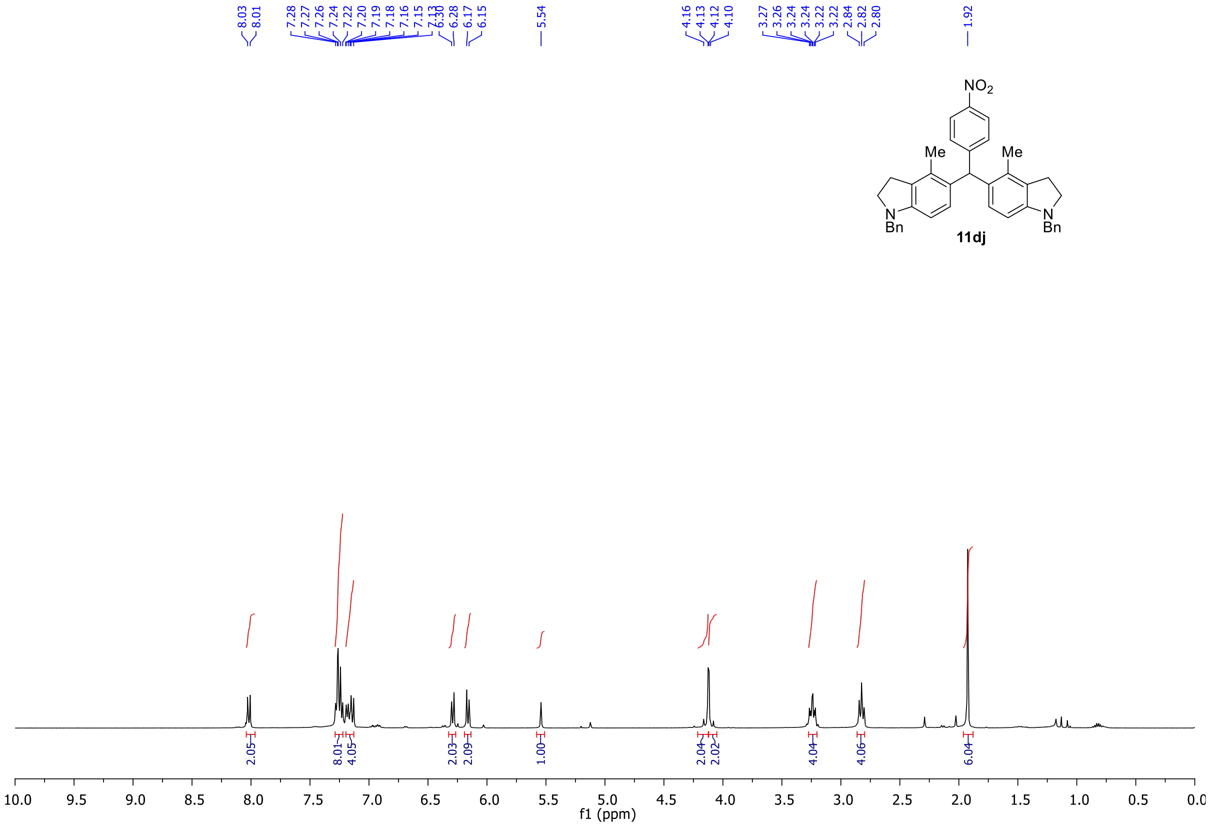

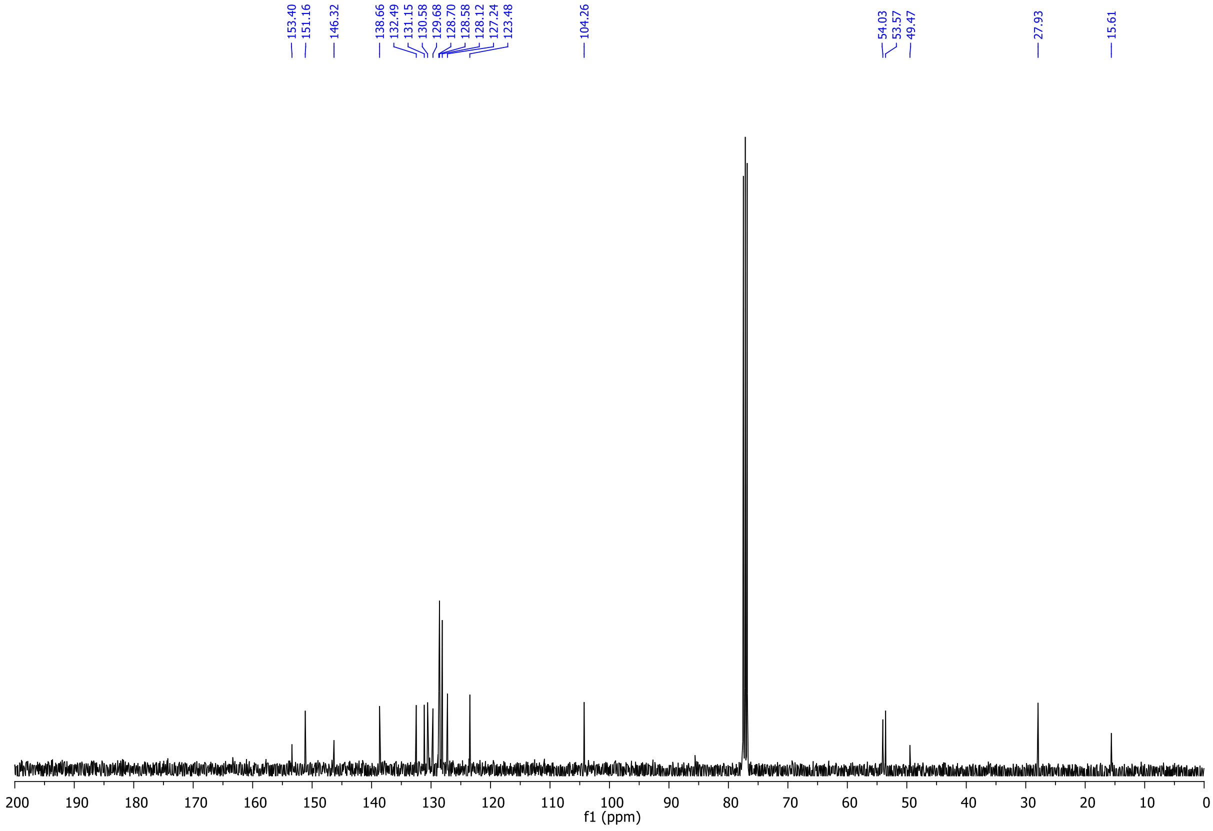

^{1}H NMR (400 MHz) and ^{13}C{^{1}H} NMR (100 MHz) spectra of **11dj** ($CDCl_3$)

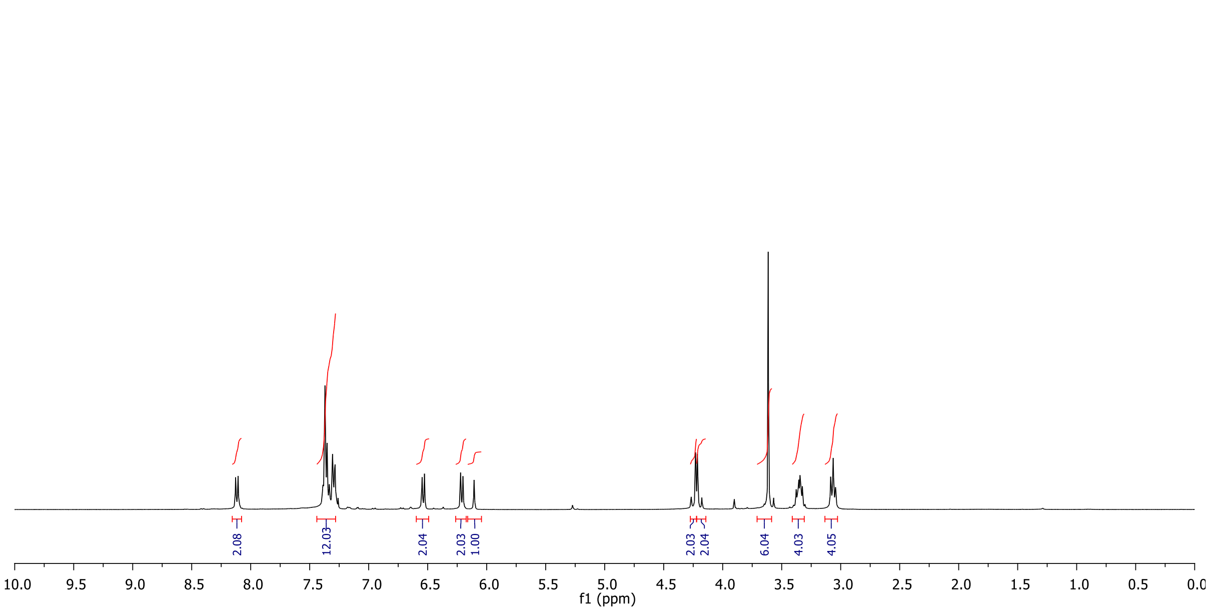

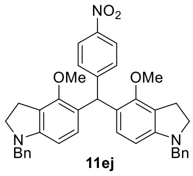

11ej

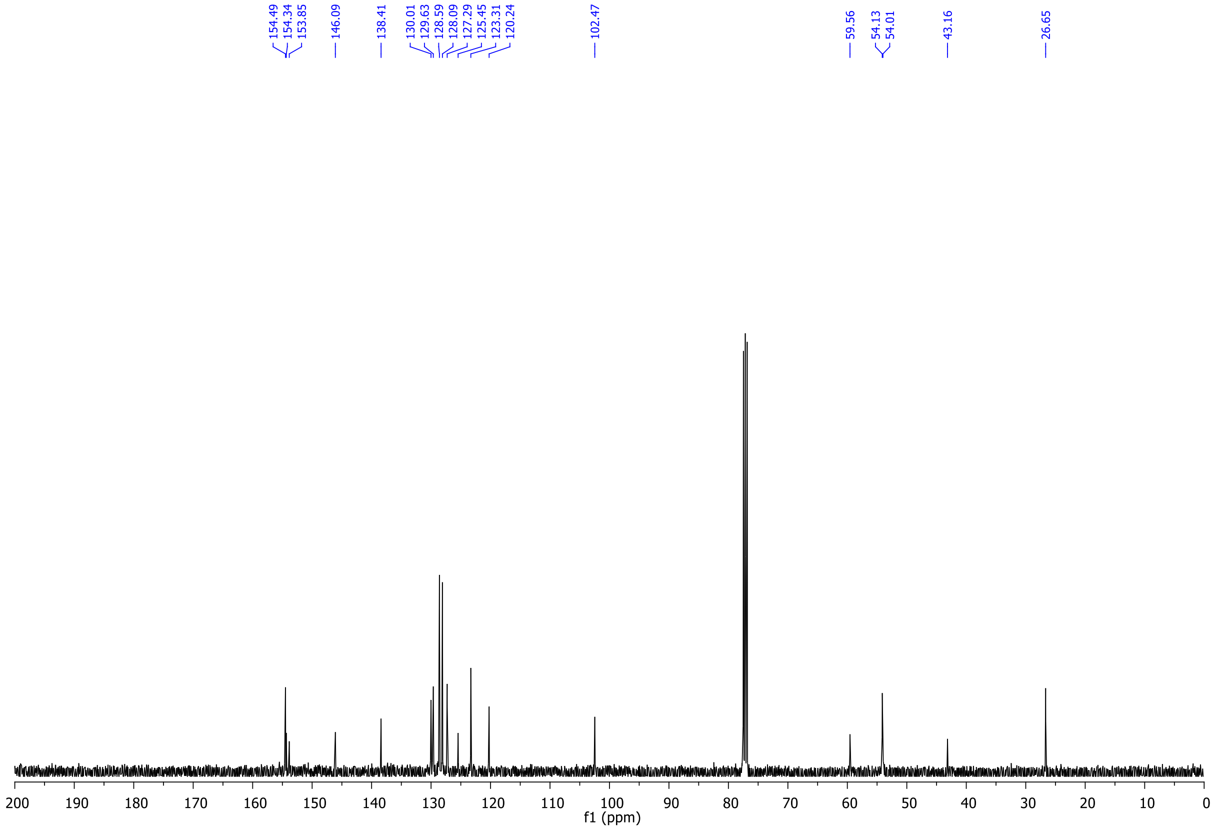

^{1}H NMR (400 MHz) and ^{13}C{^{1}H} NMR (100 MHz) spectra of **11ej** ($CDCl_3$)

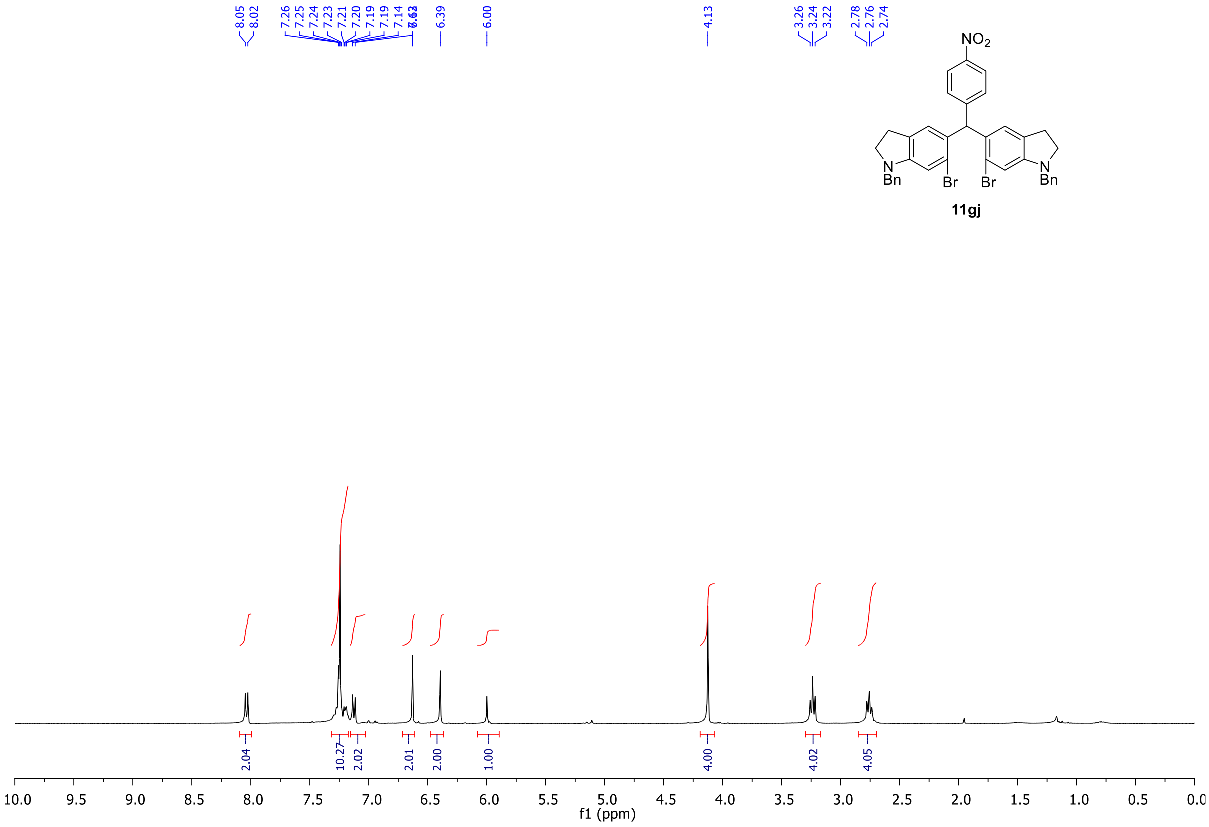

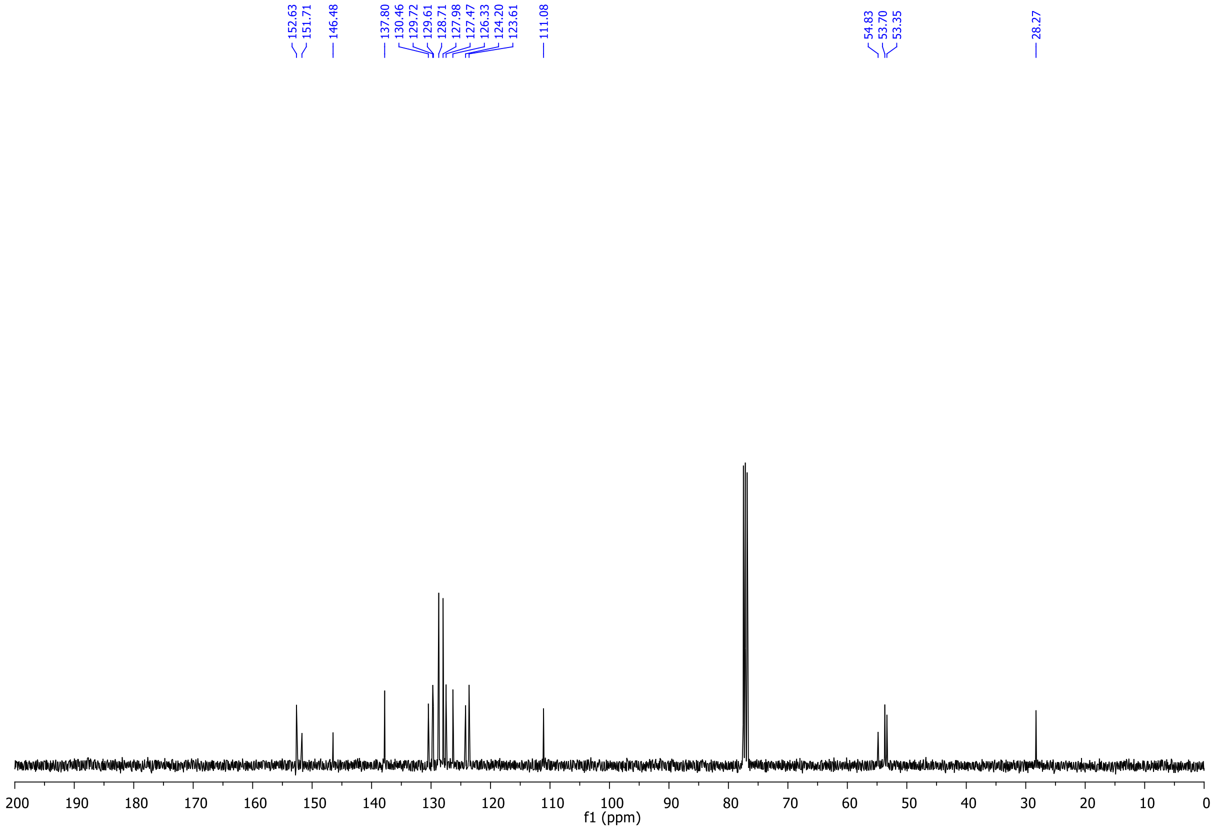

^1H NMR (400 MHz) and $^{13}C\{^1H\}$ NMR (100 MHz) spectra of **11gj** ($CDCl_3$)

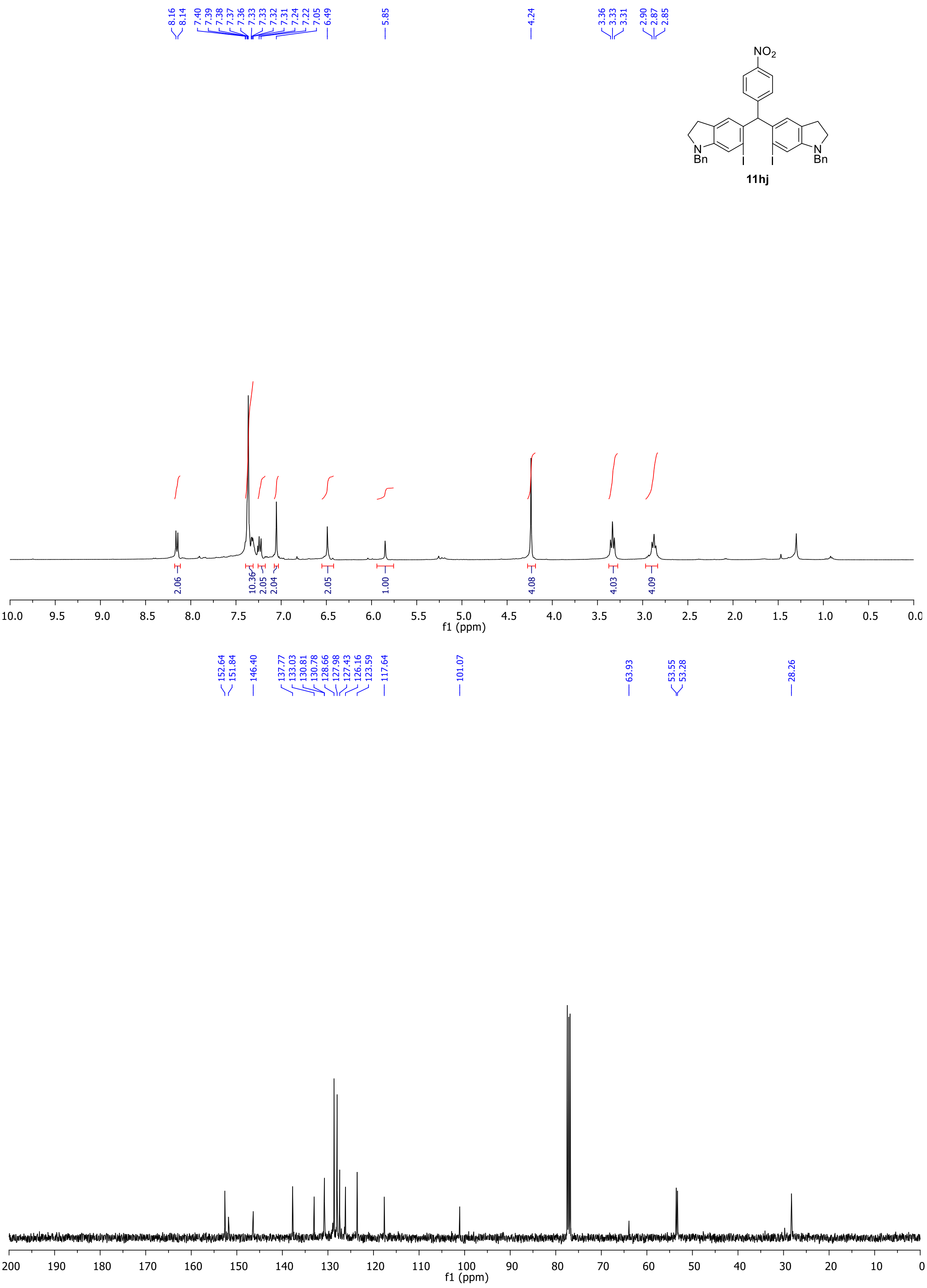

^{1}H NMR (400 MHz) and $^{13}C\{^{1}H\}$ NMR (100 MHz) spectra of **11hj** ($CDCl_3$)

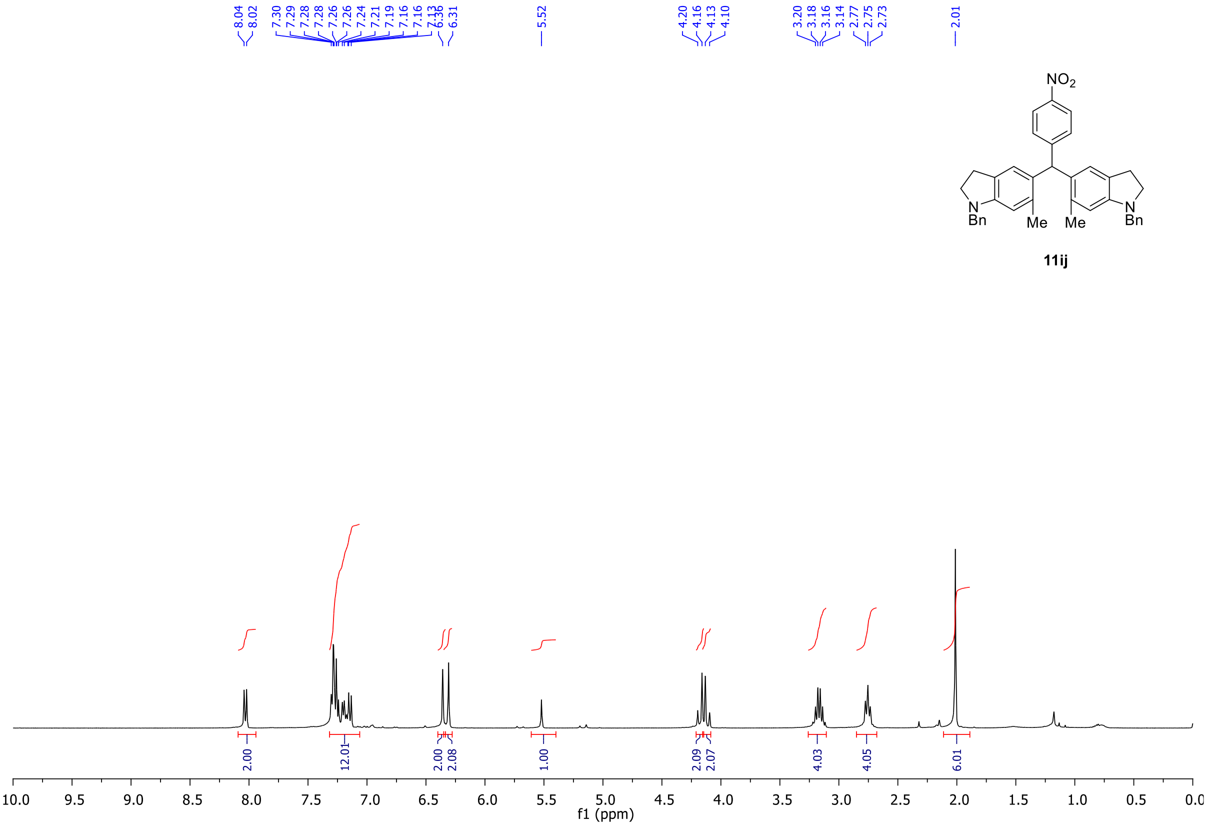

11ij

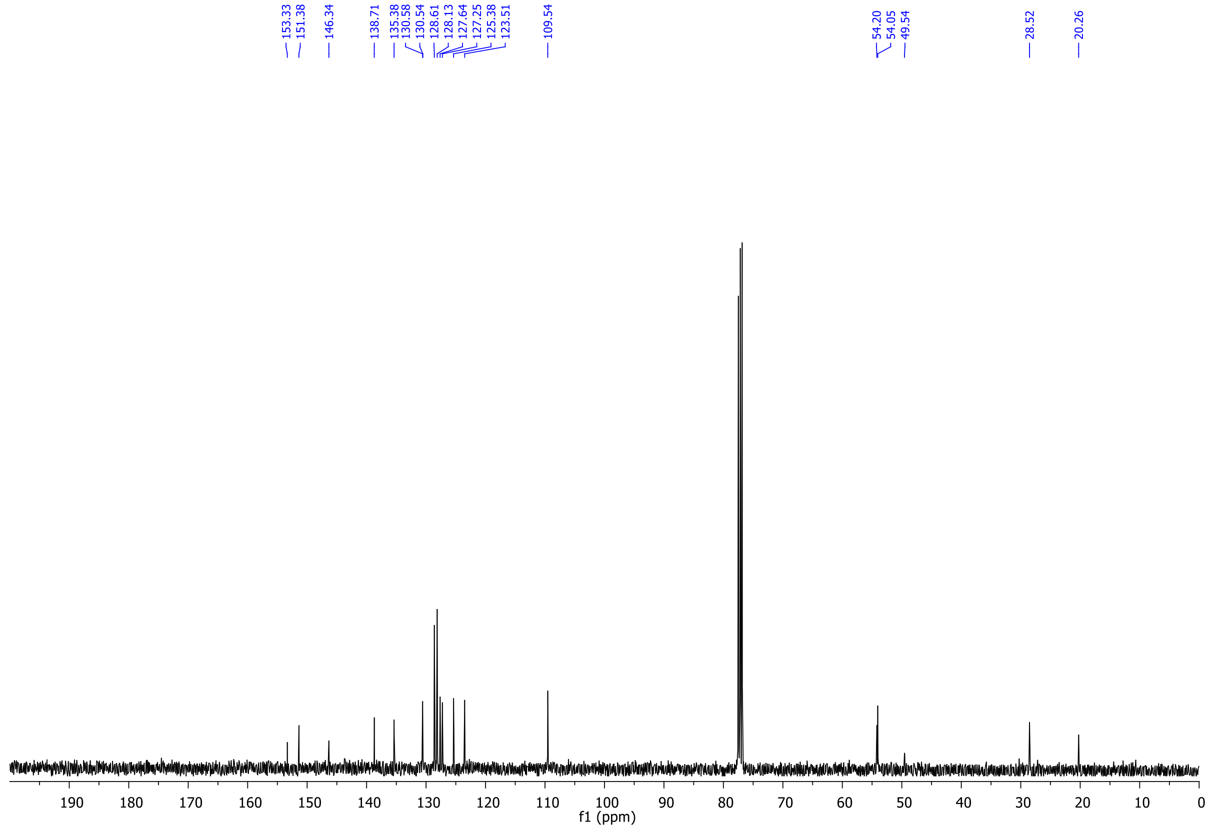

^{1}H NMR (400 MHz) and $^{13}C\{^{1}H\}$ NMR (100 MHz) spectra of **11ij** ($CDCl_3$)

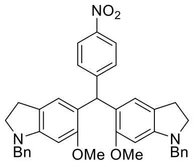

11jj

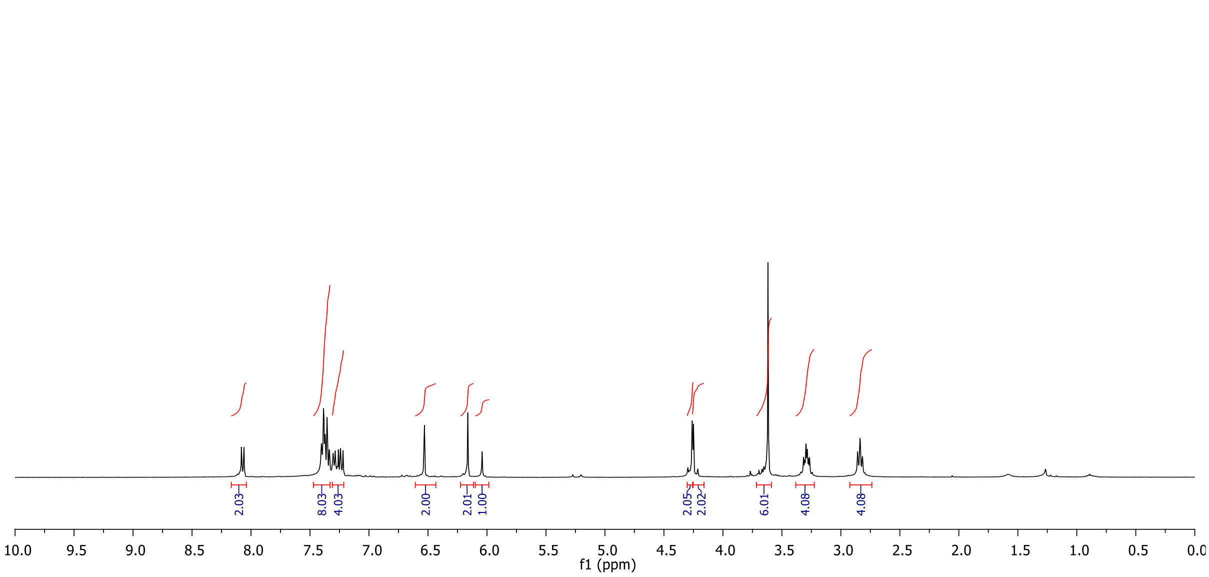

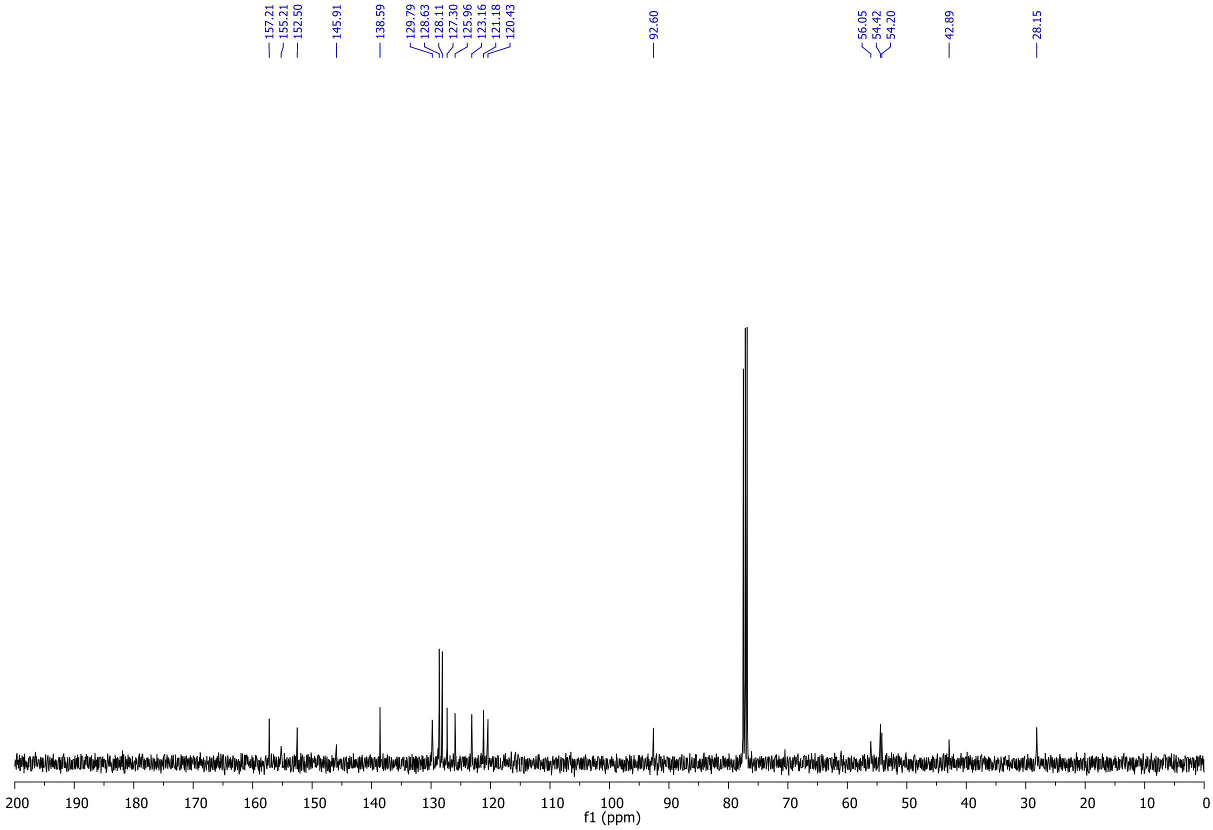

^{1}H NMR (400 MHz) and $^{13}C\{^{1}H\}$ NMR (100 MHz) spectra of **11jj** ($CDCl_3$)

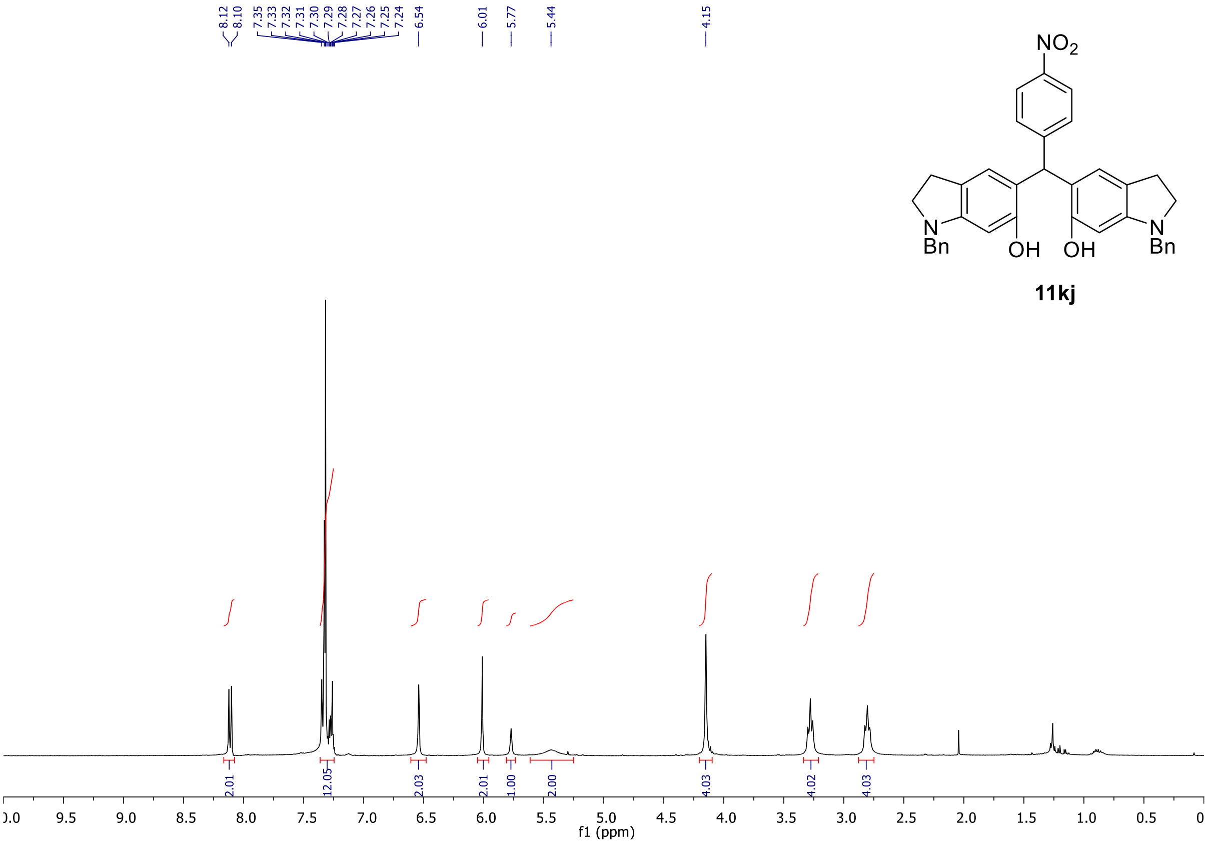

11kj

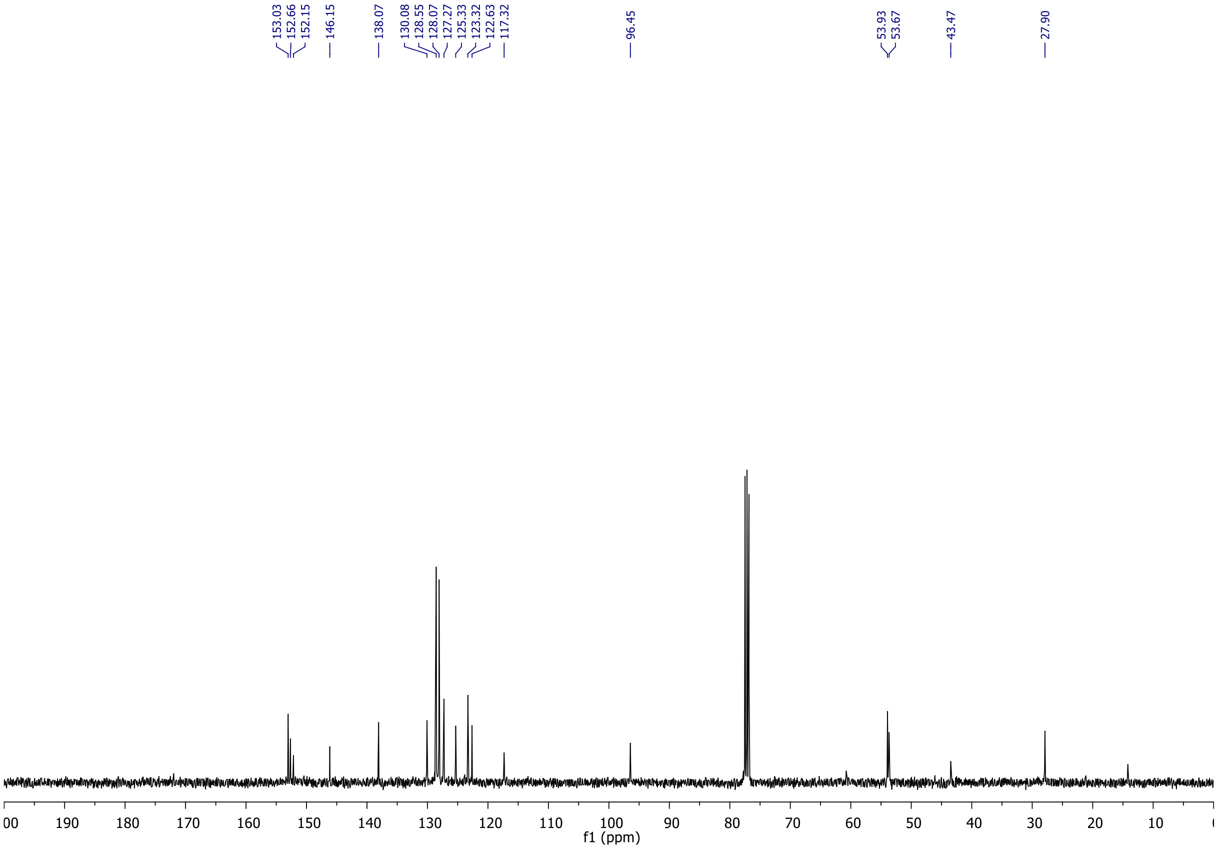

^{1}H NMR (400 MHz) and ^{13}C{^{1}H} NMR (100 MHz) spectra of **11kj** ($CDCl_3$)

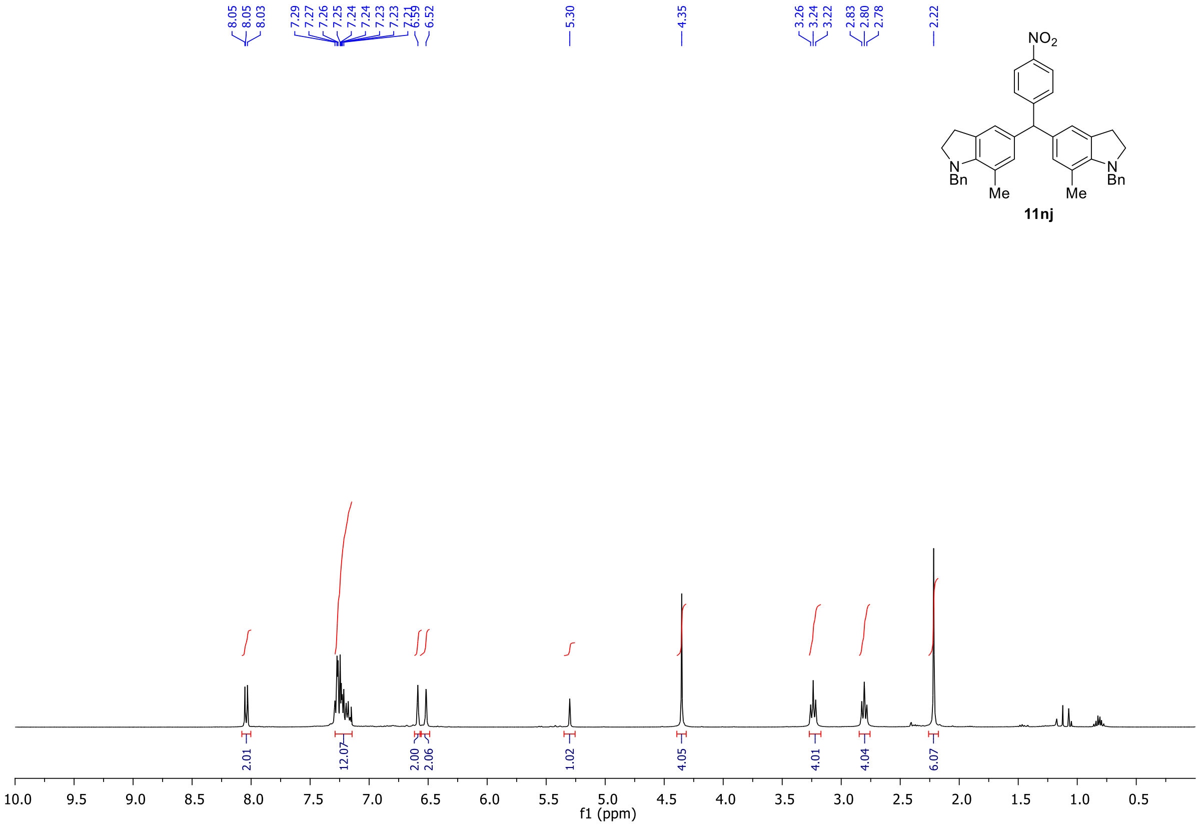

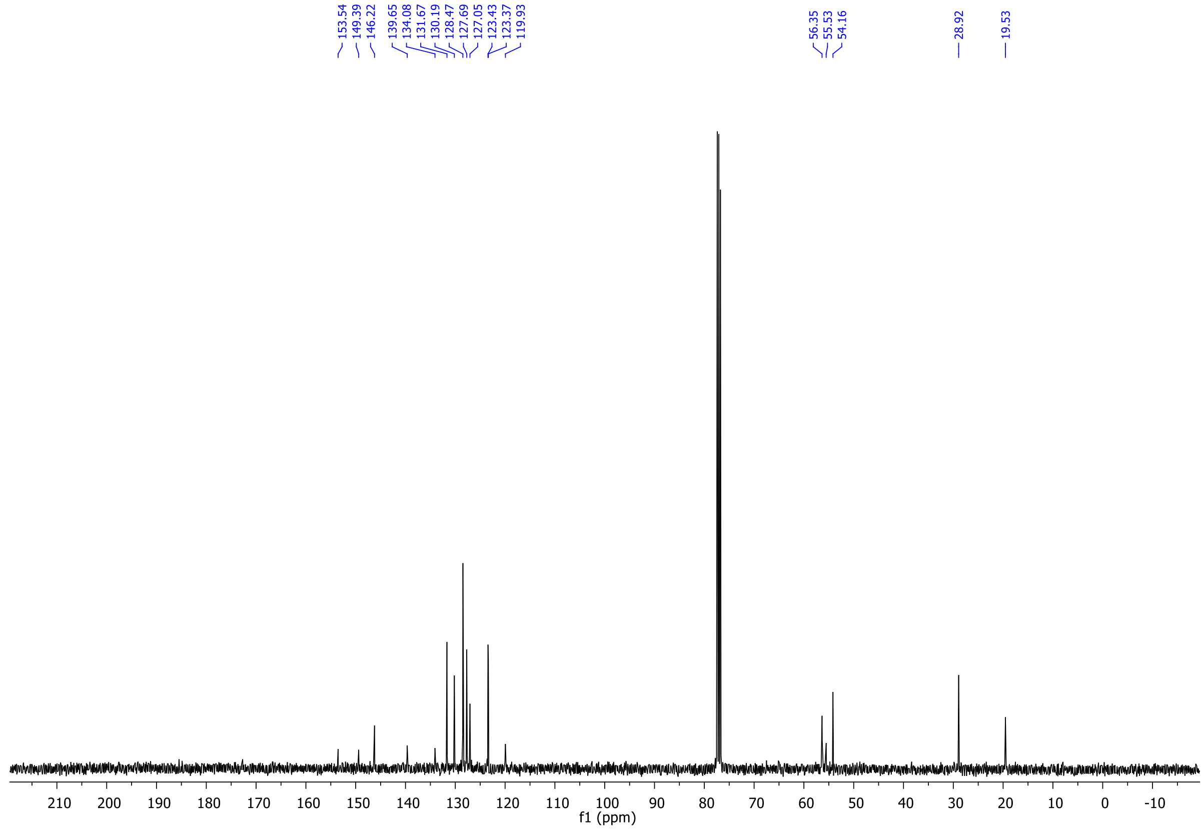

^{1}H NMR (400 MHz) and ^{13}C{^{1}H} NMR (100 MHz) spectra of **11nj** ($CDCl_3$)

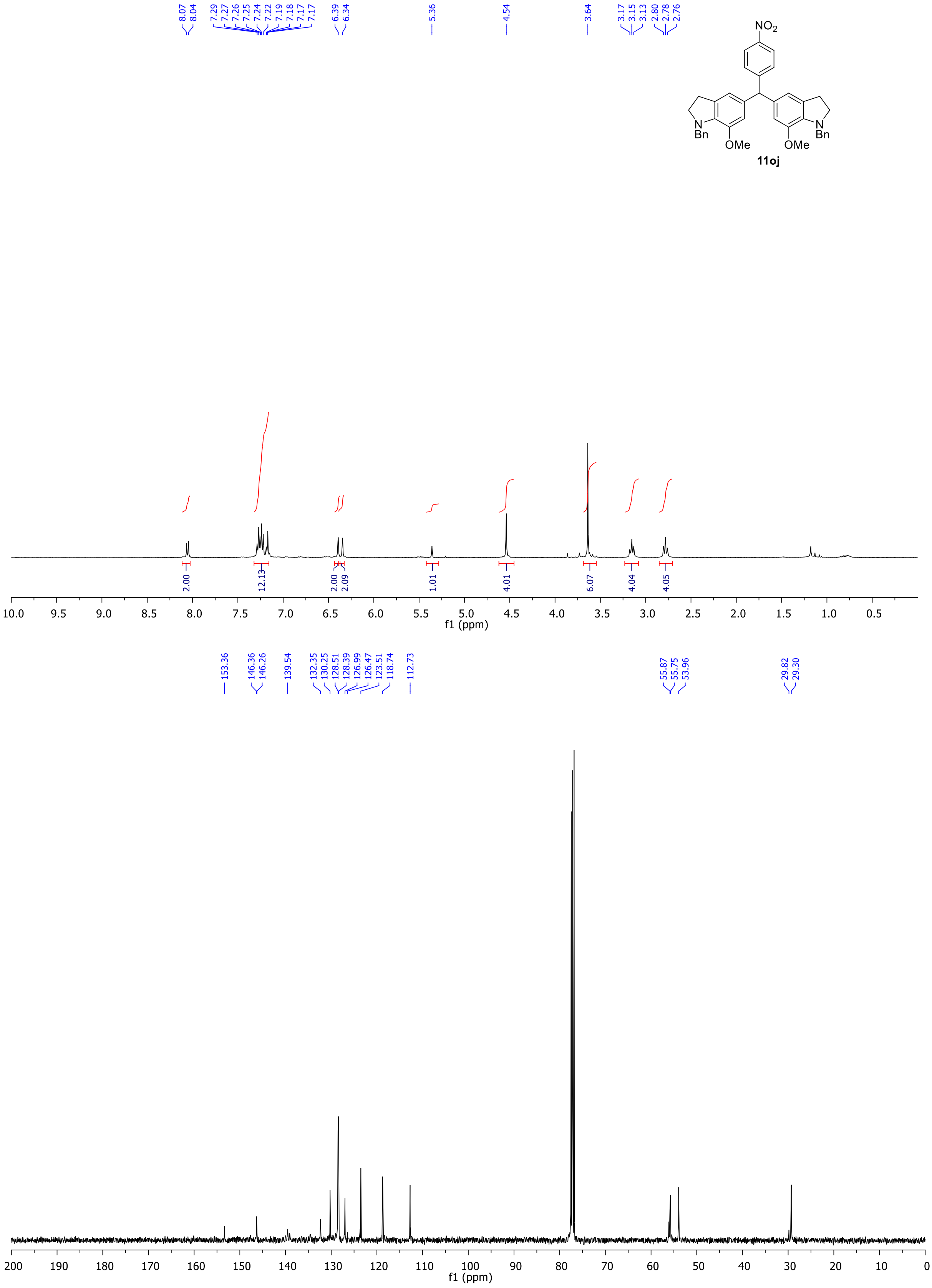

^{1}H NMR (400 MHz) and $^{13}C\{^{1}H\}$ NMR (100 MHz) spectra of **11oj** ($CDCl_3$)

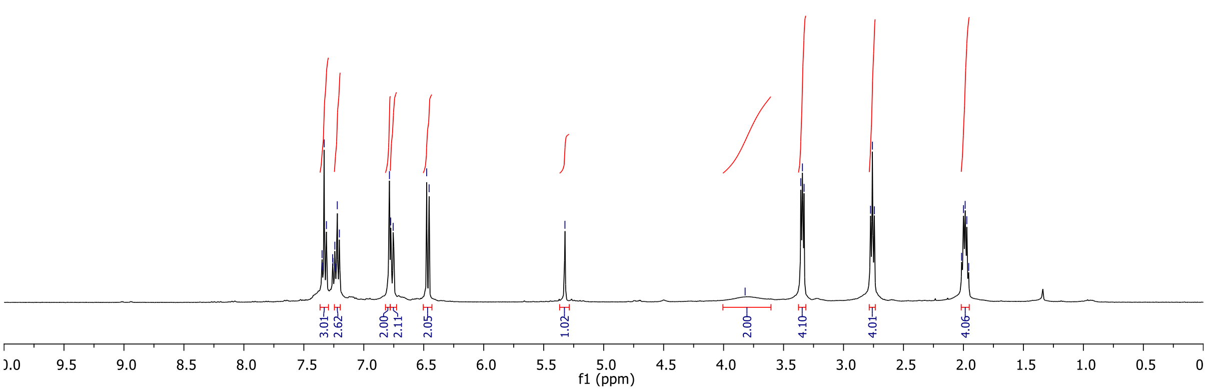

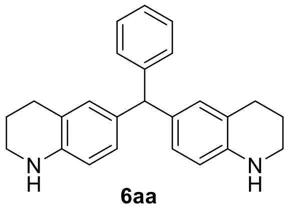

6aa

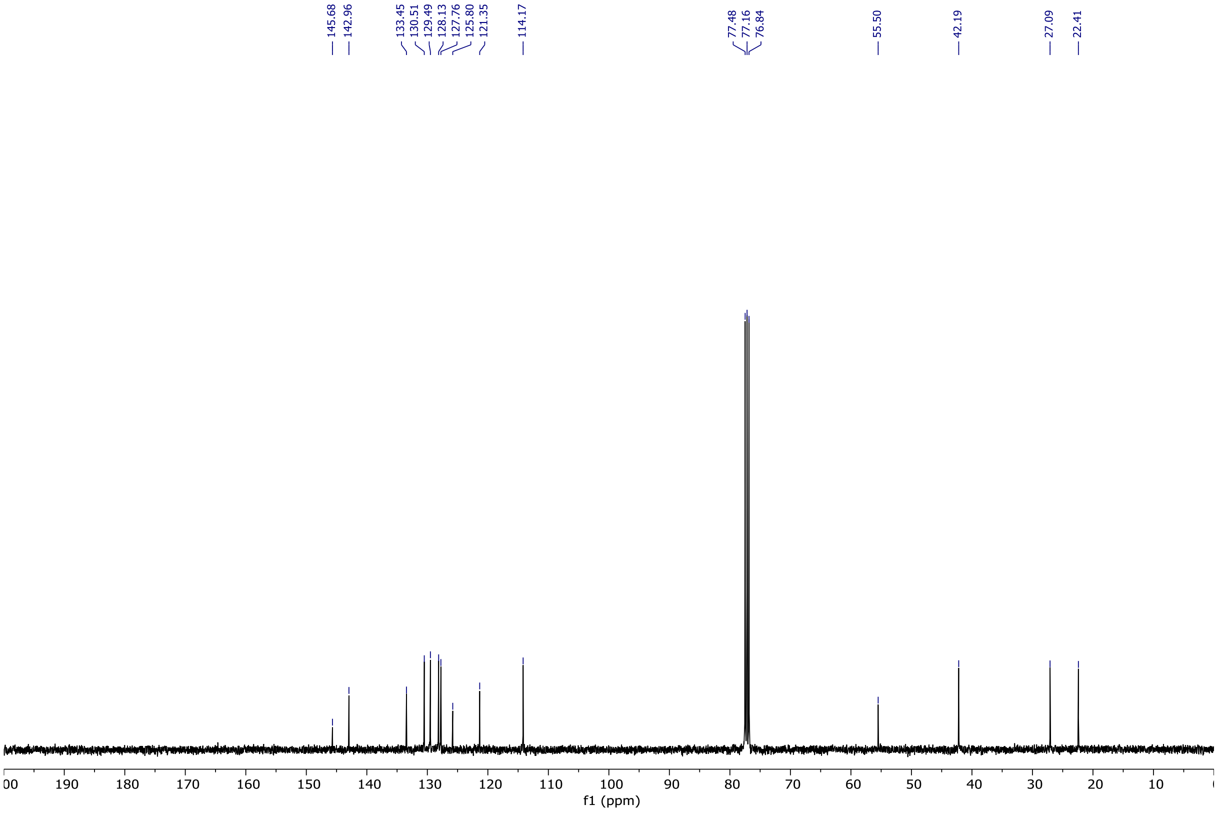

^{1}H NMR (400 MHz) and $^{13}C\{^{1}H\}$ NMR (100 MHz) spectra of **6aa** ($CDCl_3$)

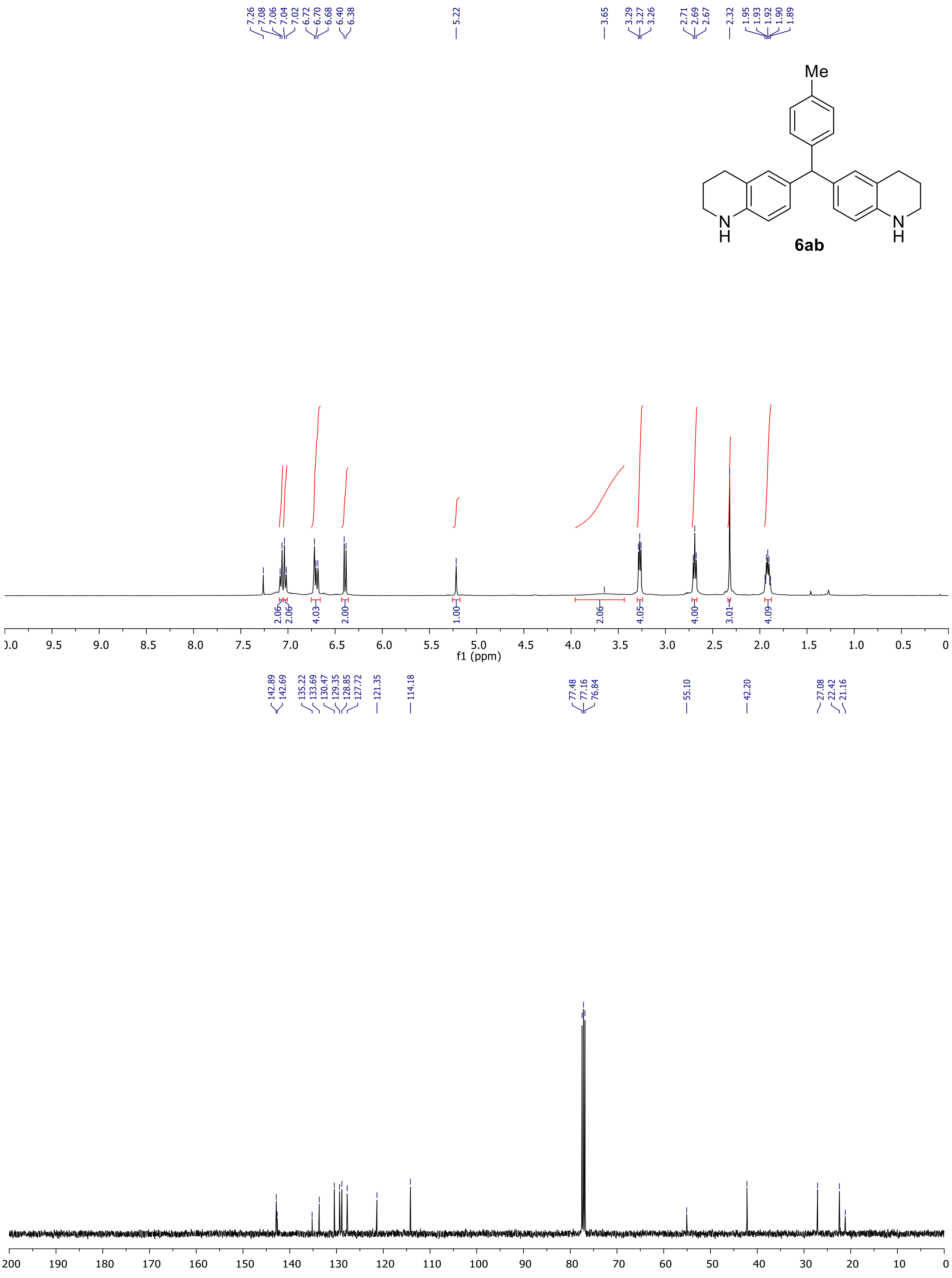

^{1}H NMR (400 MHz) and ^{13}C{^{1}H} NMR (100 MHz) spectra of **6ab** ($CDCl_3$)

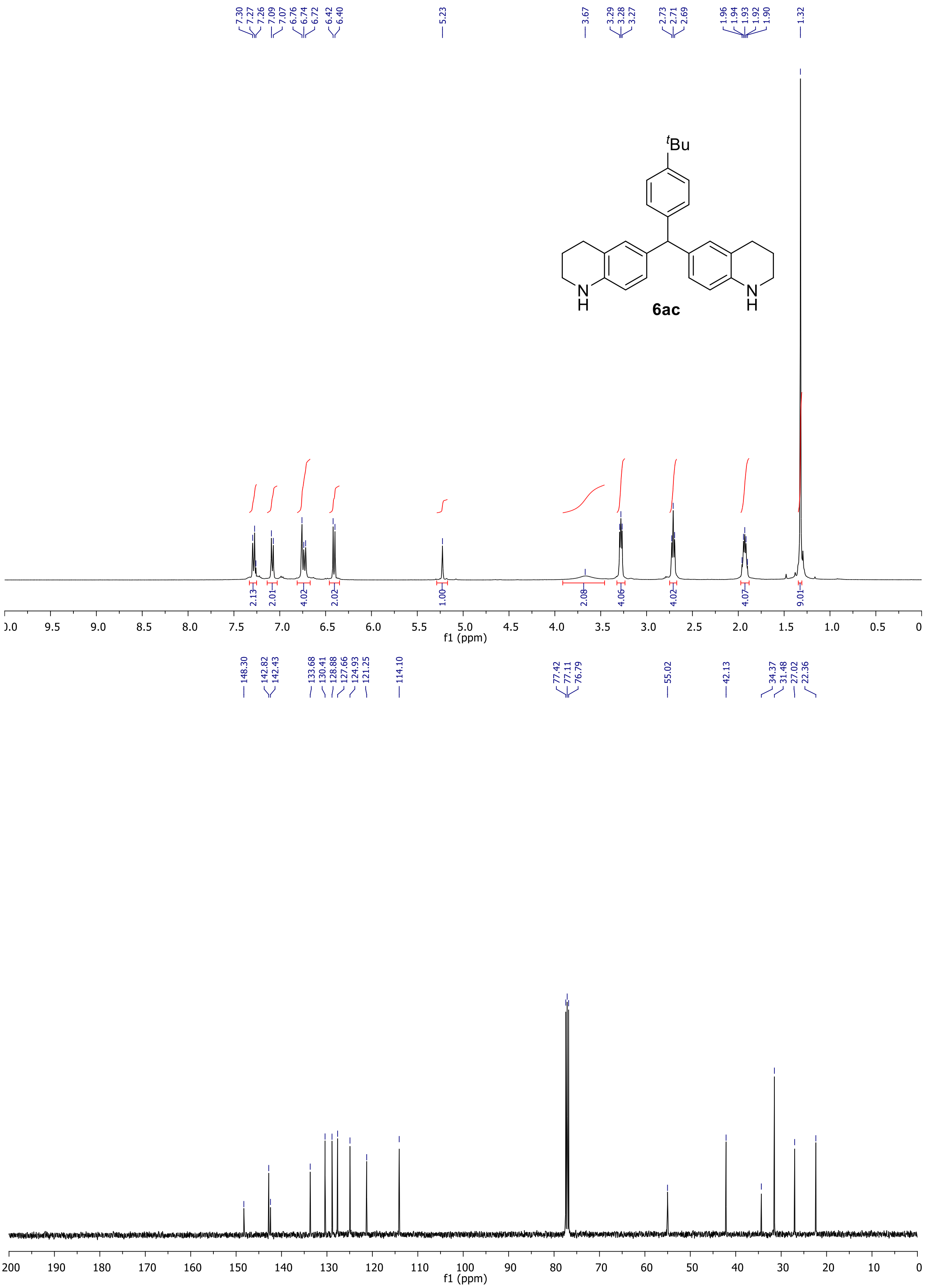

^{1}H NMR (400 MHz) and ^{13}C{^{1}H} NMR (100 MHz) spectra of **6ac** ($CDCl_3$)

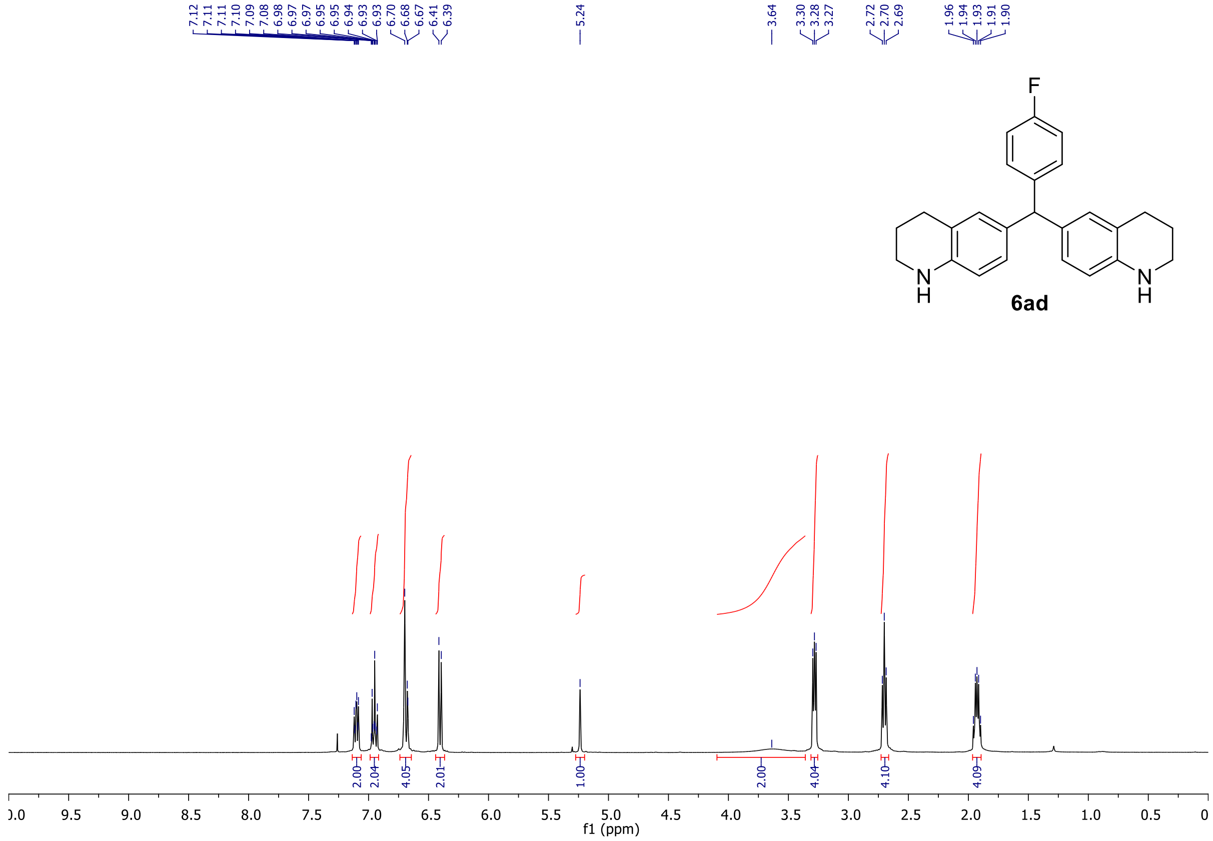

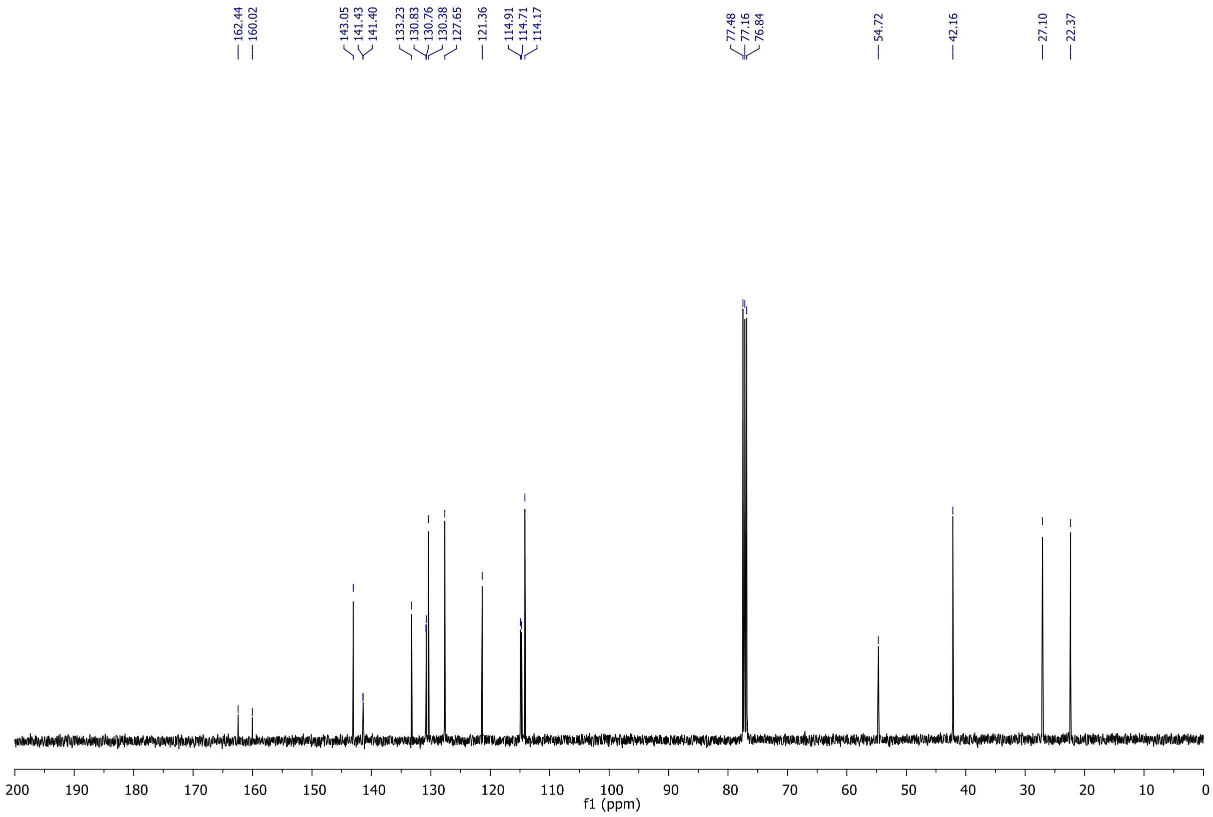

^{1}H NMR (400 MHz) and $^{13}C\{^{1}H\}$ NMR (100 MHz) spectra of **6ad** ($CDCl_3$)

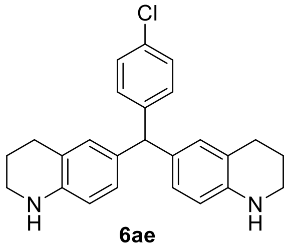

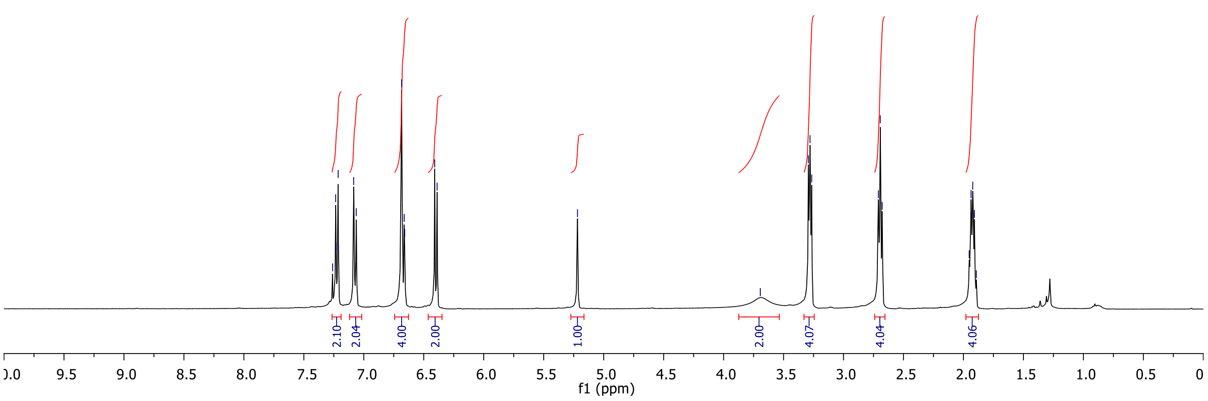

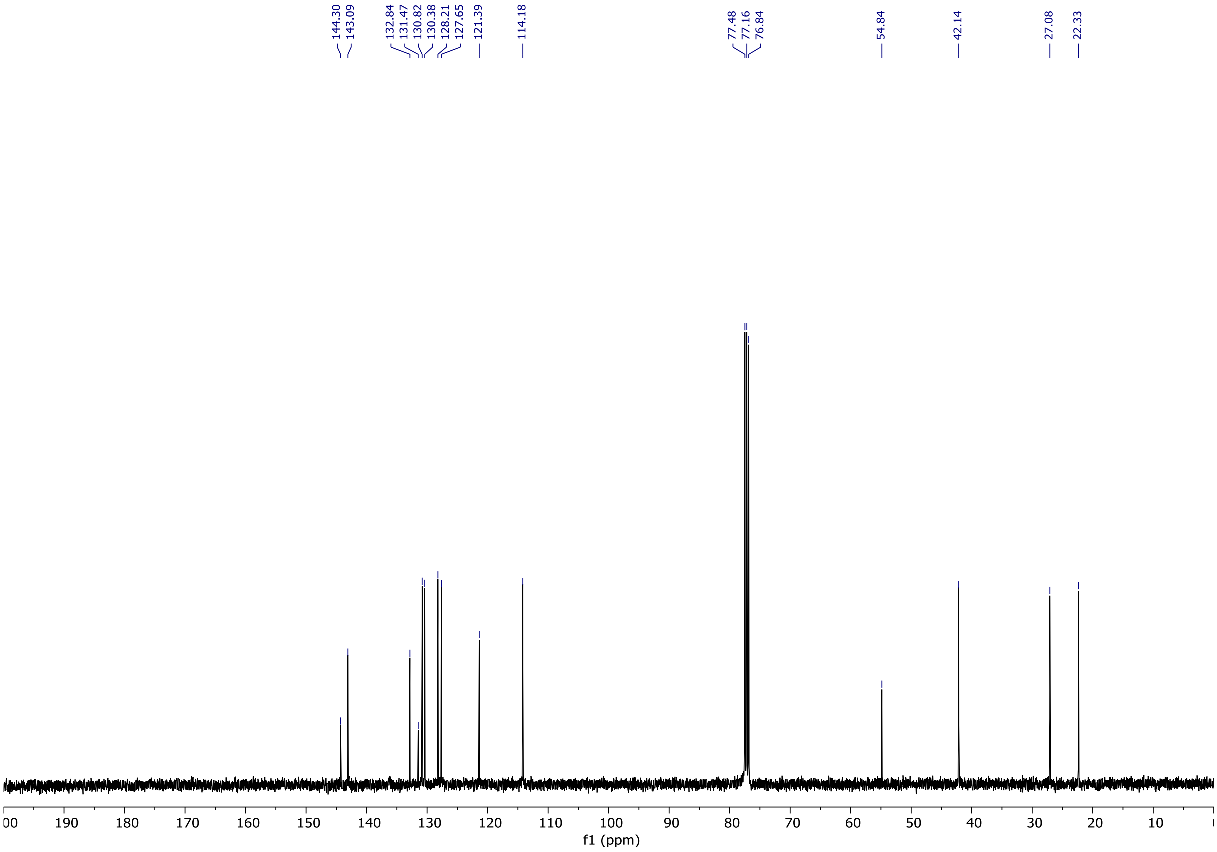

^{1}H NMR (400 MHz) and ^{13}C{^{1}H} NMR (100 MHz) spectra of **6ae** ($CDCl_3$)

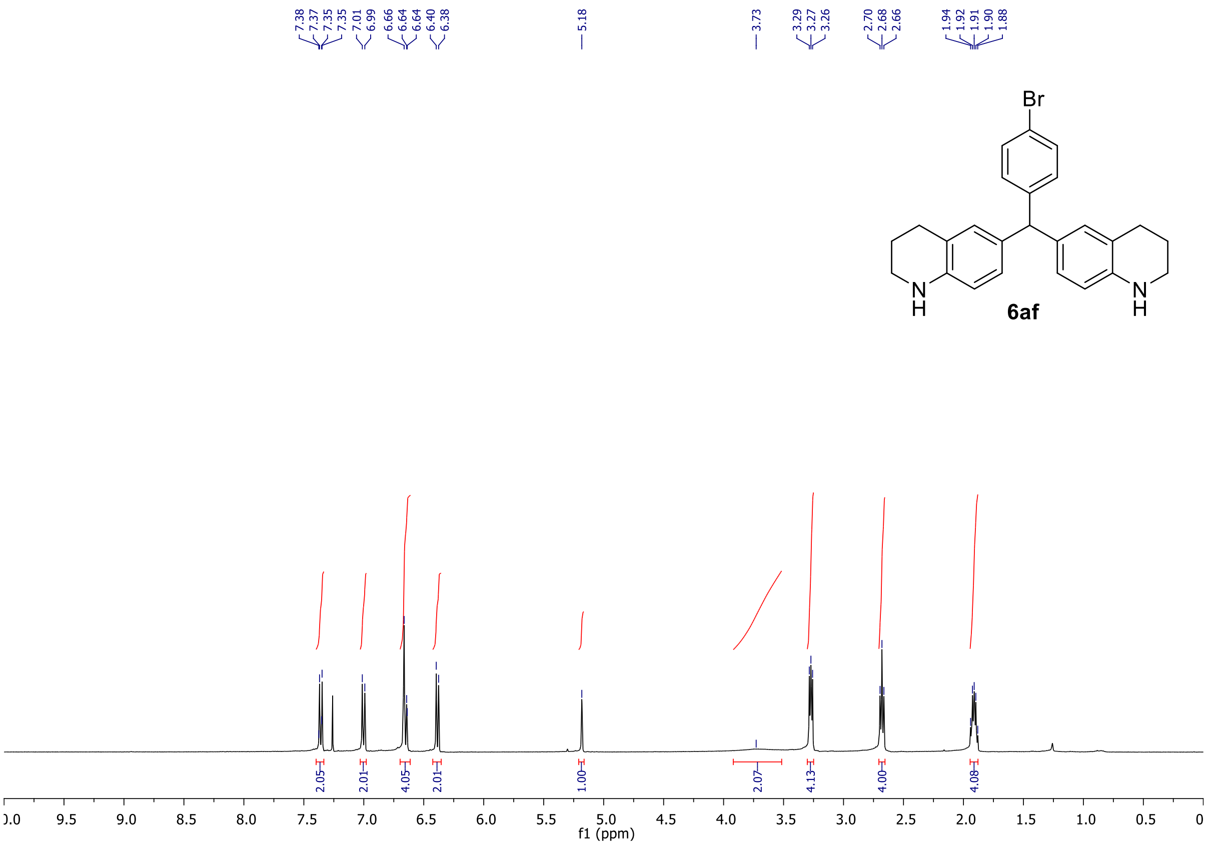

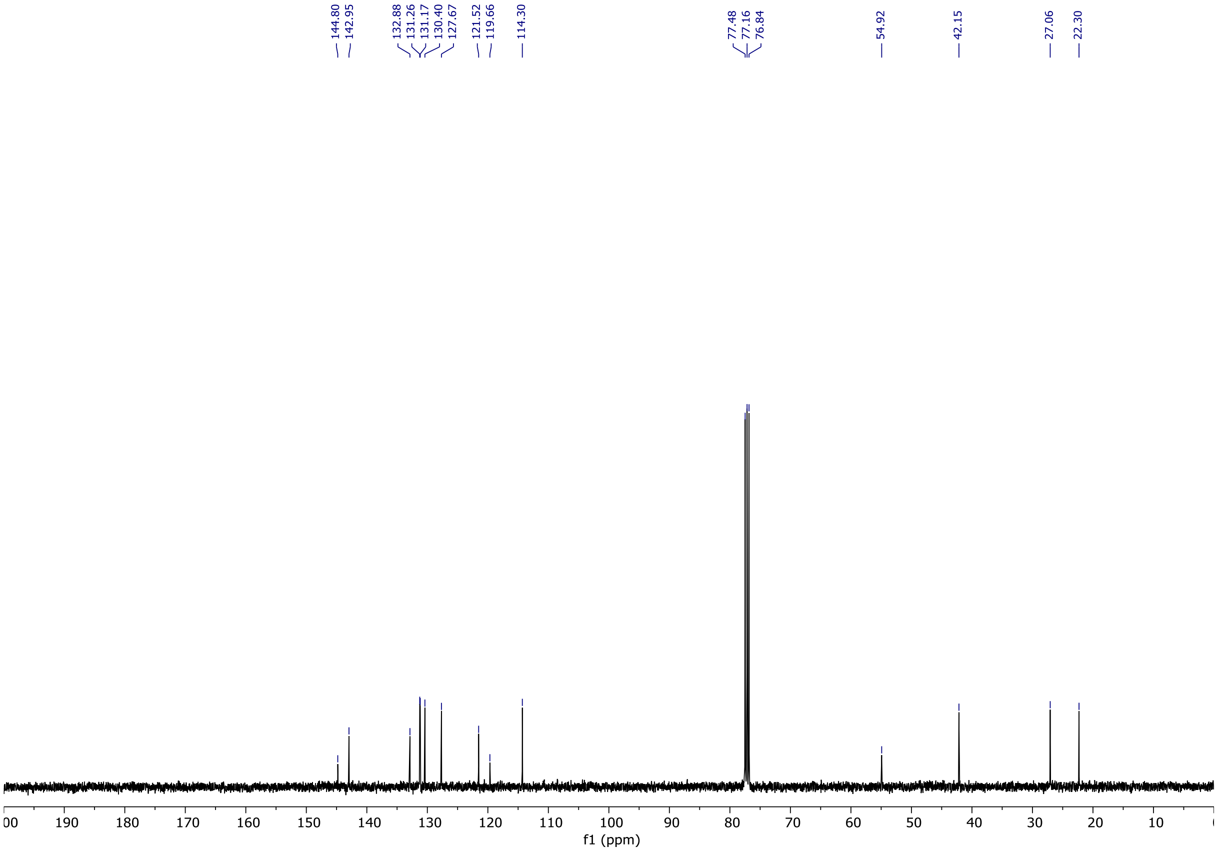

^{1}H NMR (400 MHz) and $^{13}C\{^{1}H\}$ NMR (100 MHz) spectra of **6af** ($CDCl_3$)

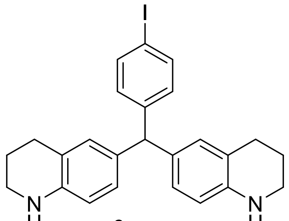

6ag

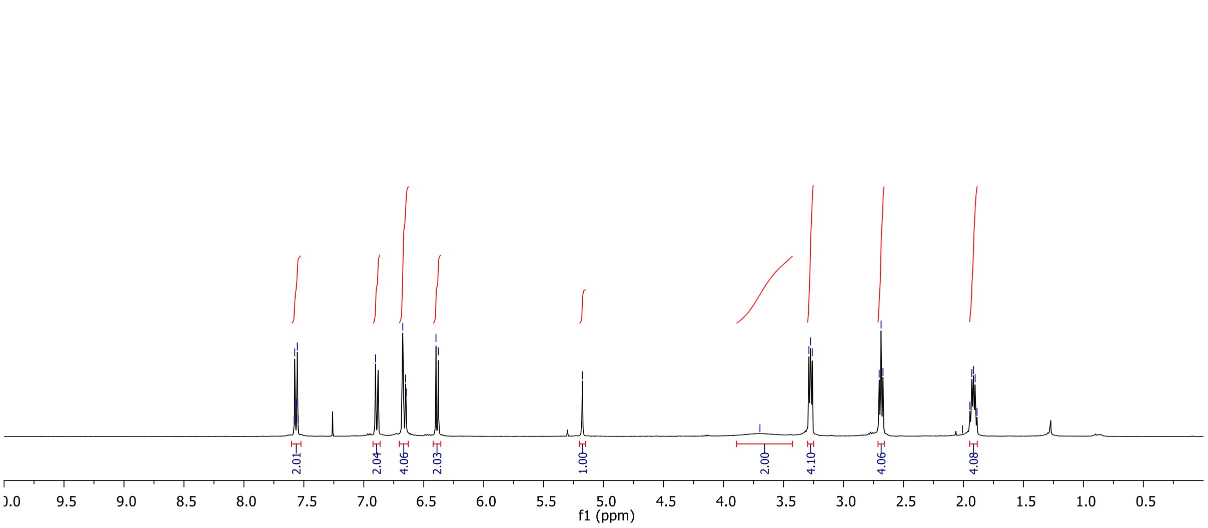

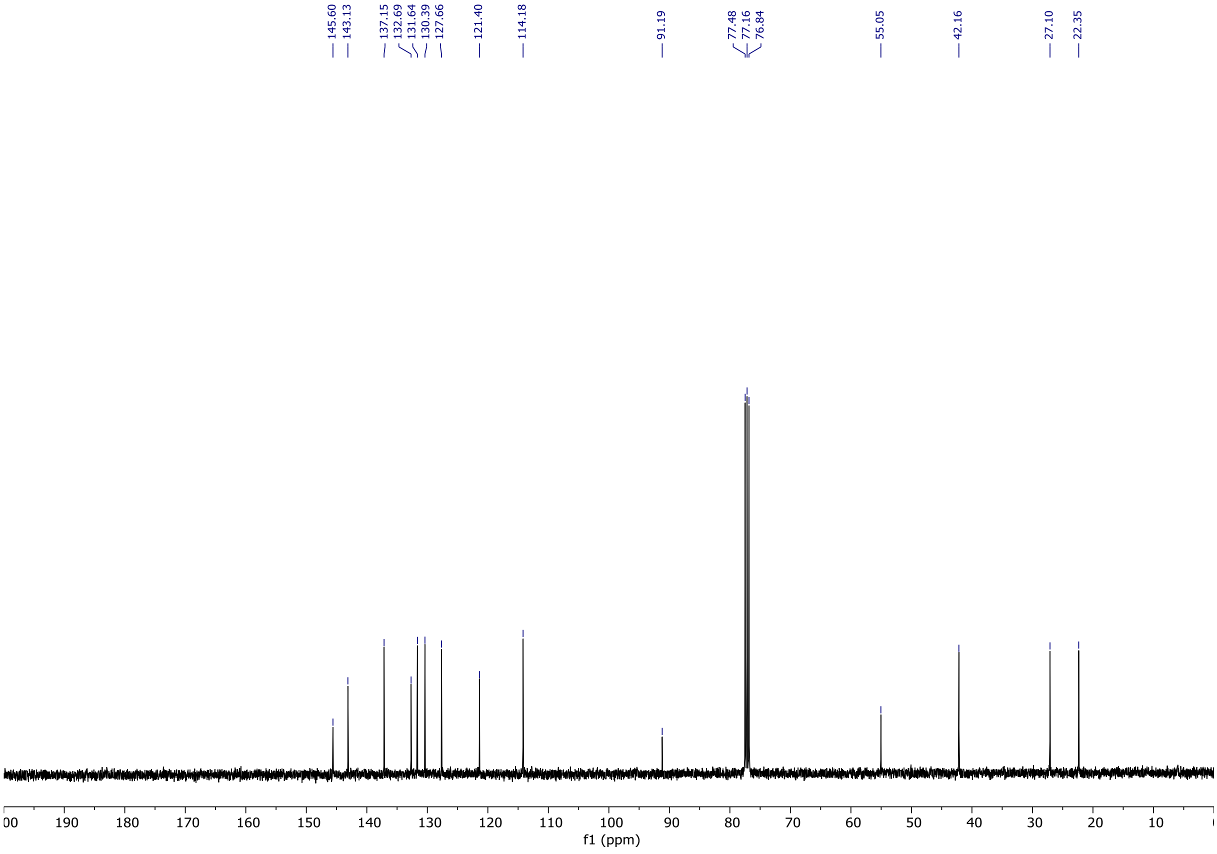

^{1}H NMR (400 MHz) and $^{13}C\{^{1}H\}$ NMR (100 MHz) spectra of **6ag** ($CDCl_3$)

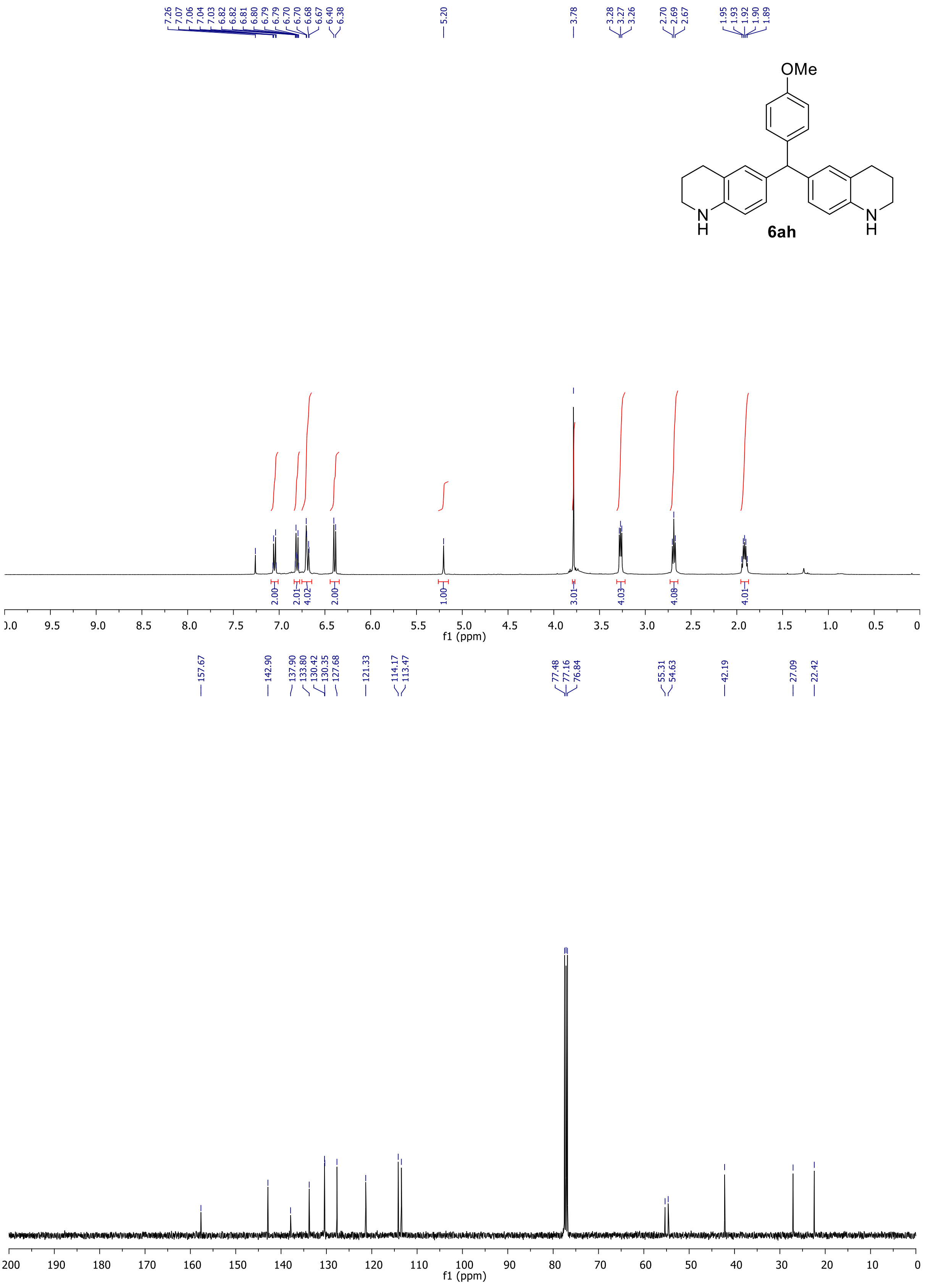

^{1}H NMR (400 MHz) and ^{13}C{^{1}H} NMR (100 MHz) spectra of **6ah** ($CDCl_3$)

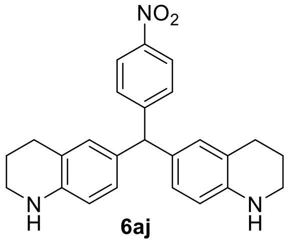

6aj

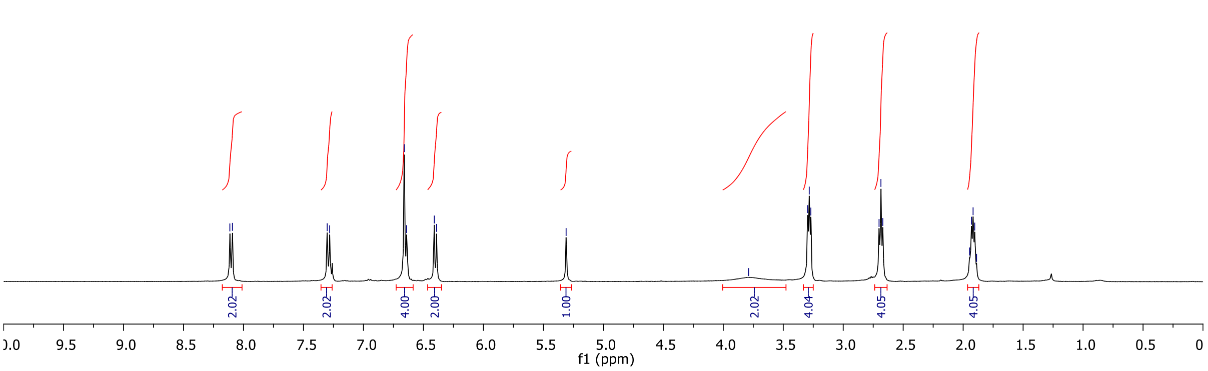

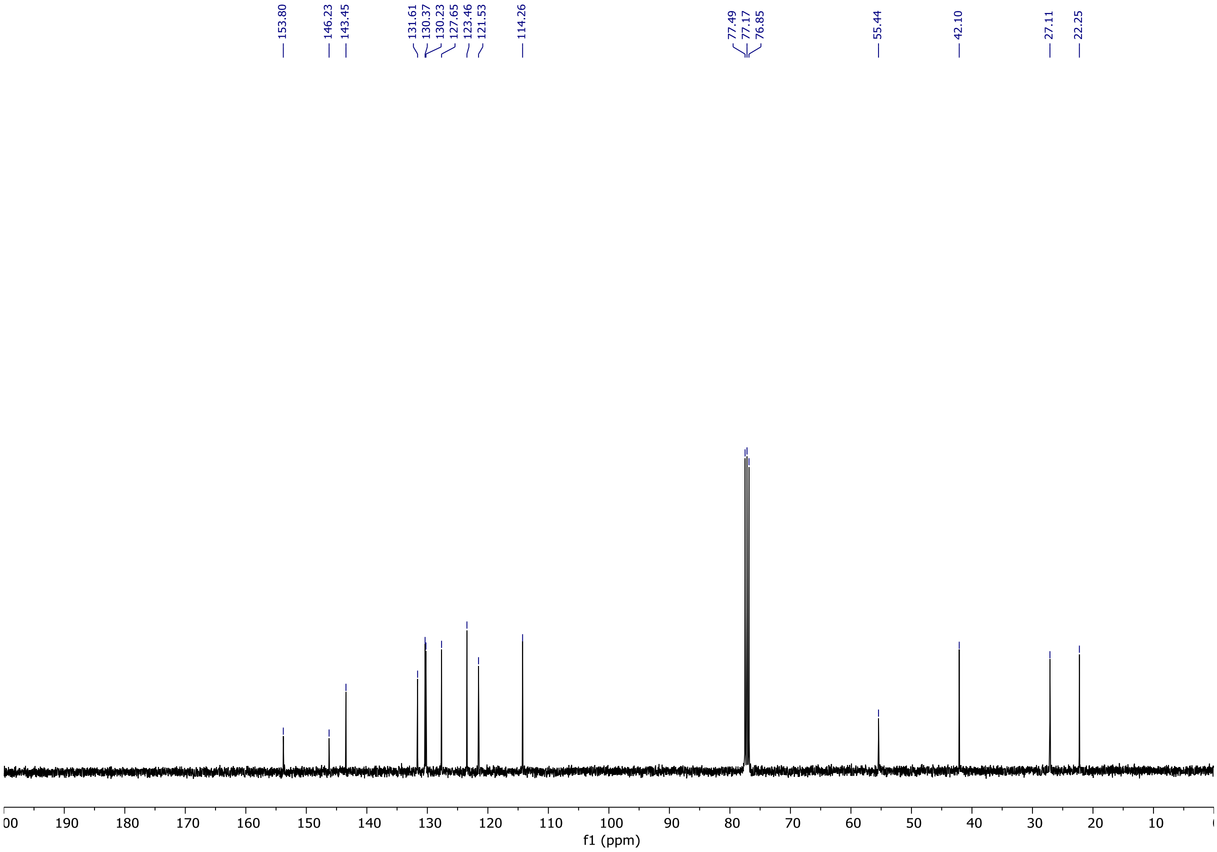

^{1}H NMR (400 MHz) and $^{13}C\{^{1}H\}$ NMR (100 MHz) spectra of **6aj** ($CDCl_3$)

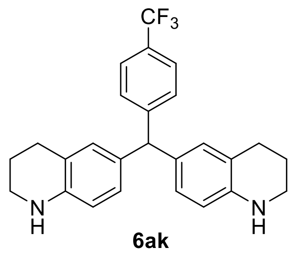

6ak

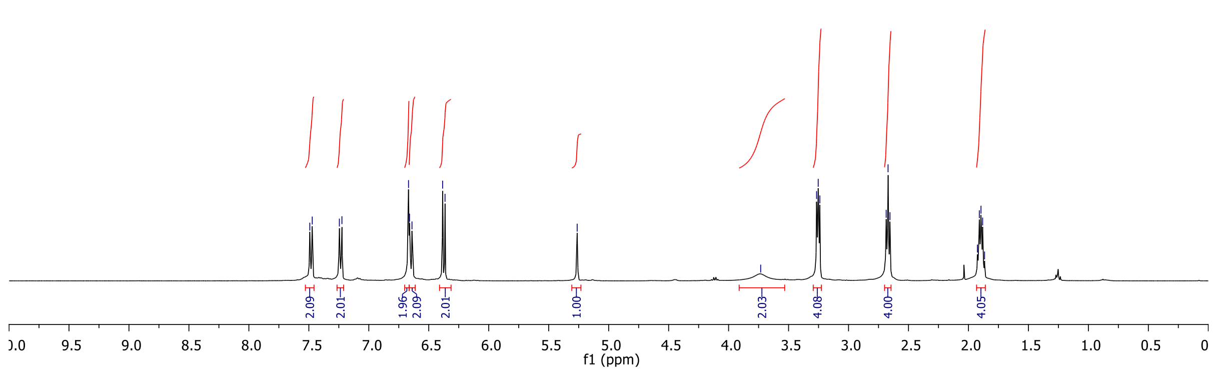

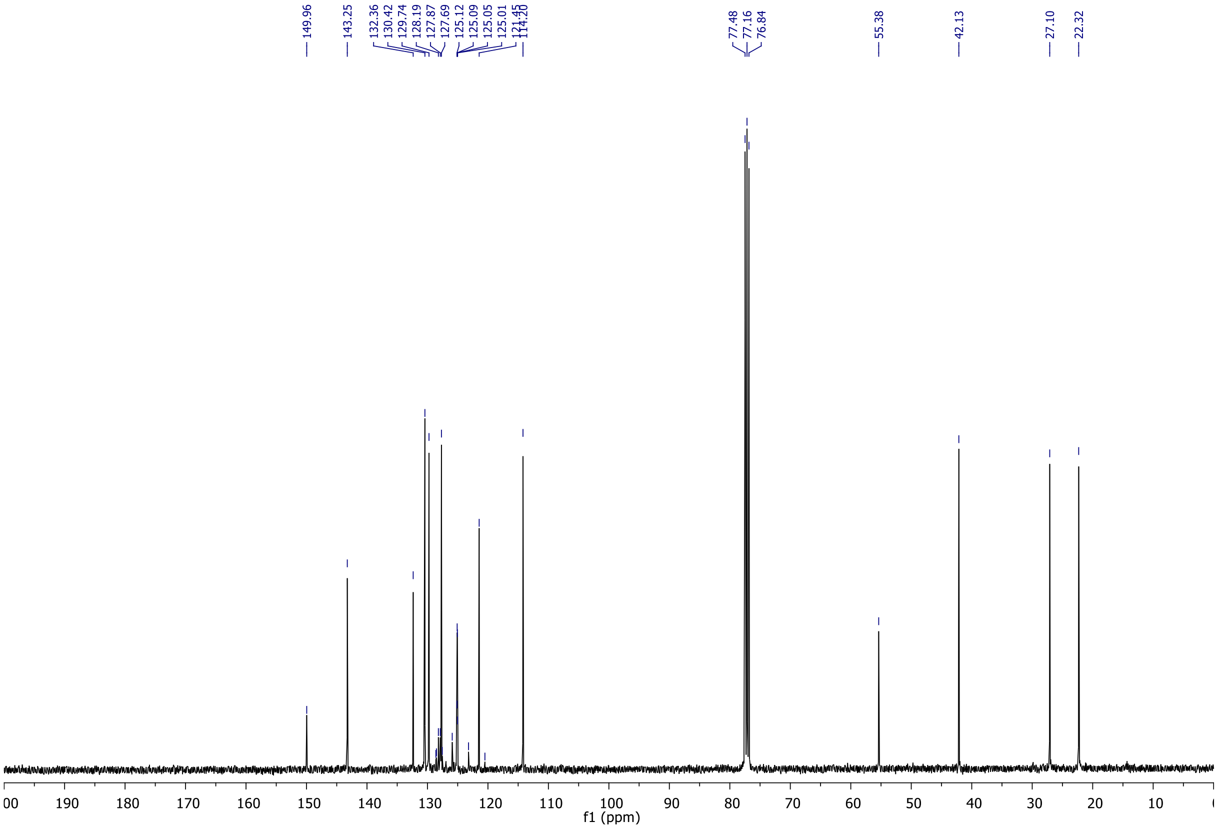

^{1}H NMR (400 MHz) and $^{13}C\{^{1}H\}$ NMR (100 MHz) spectra of **6ak** ($CDCl_3$)

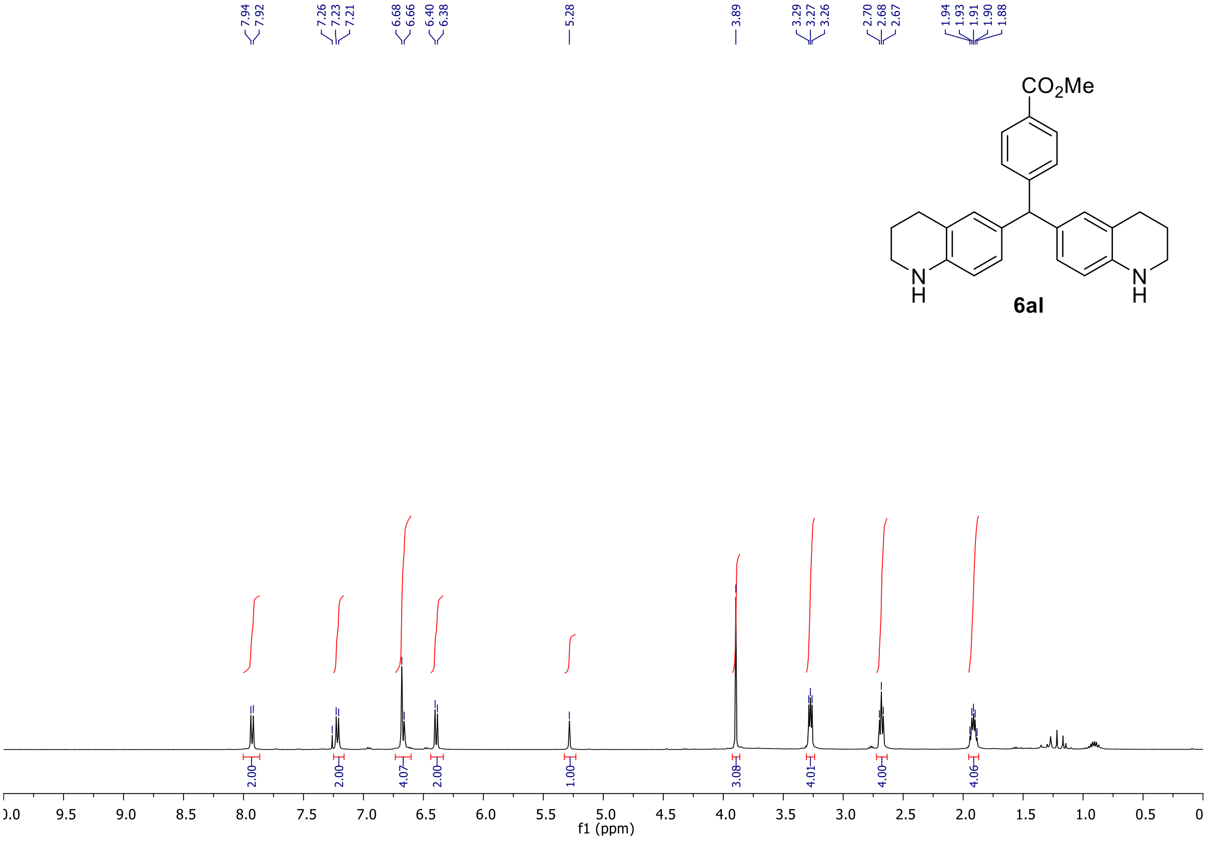

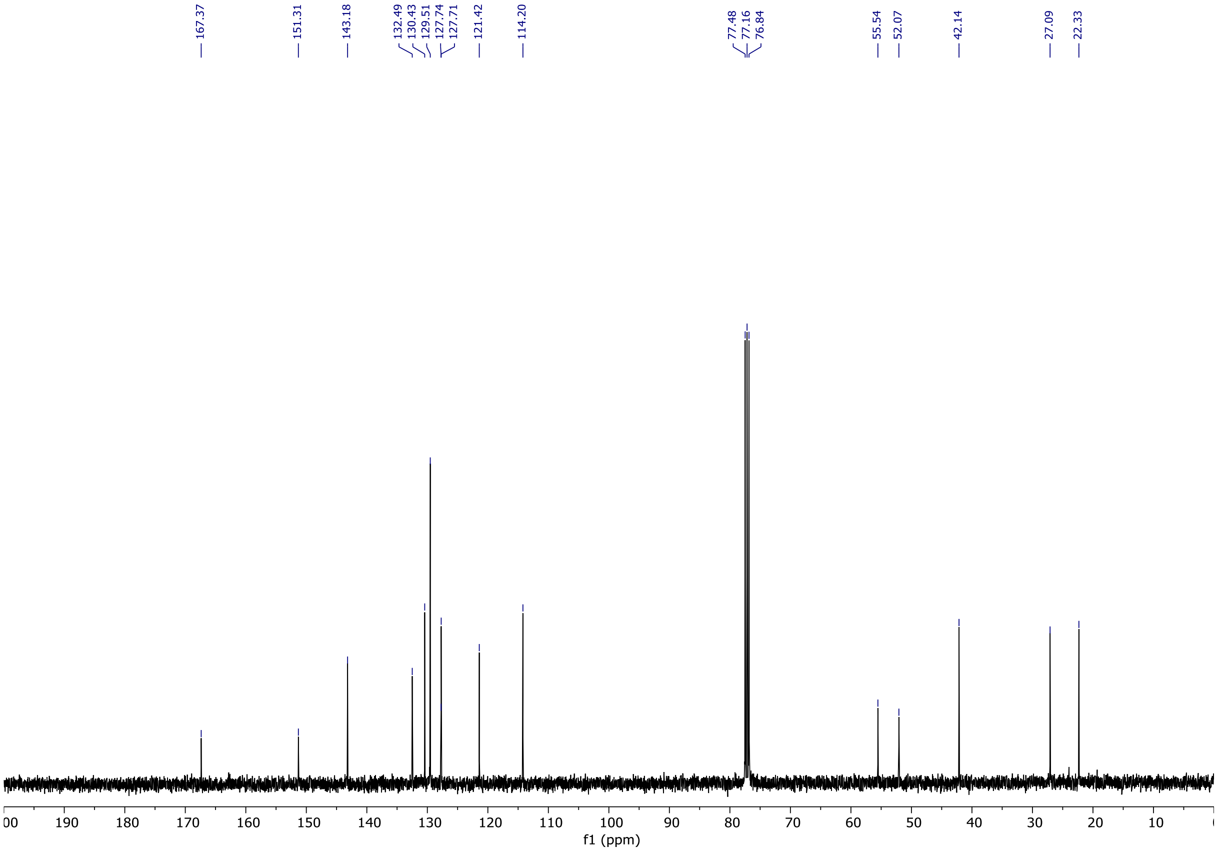

^1H NMR (400 MHz) and $^{13}C\{^1H\}$ NMR (100 MHz) spectra of **6al** ($CDCl_3$)

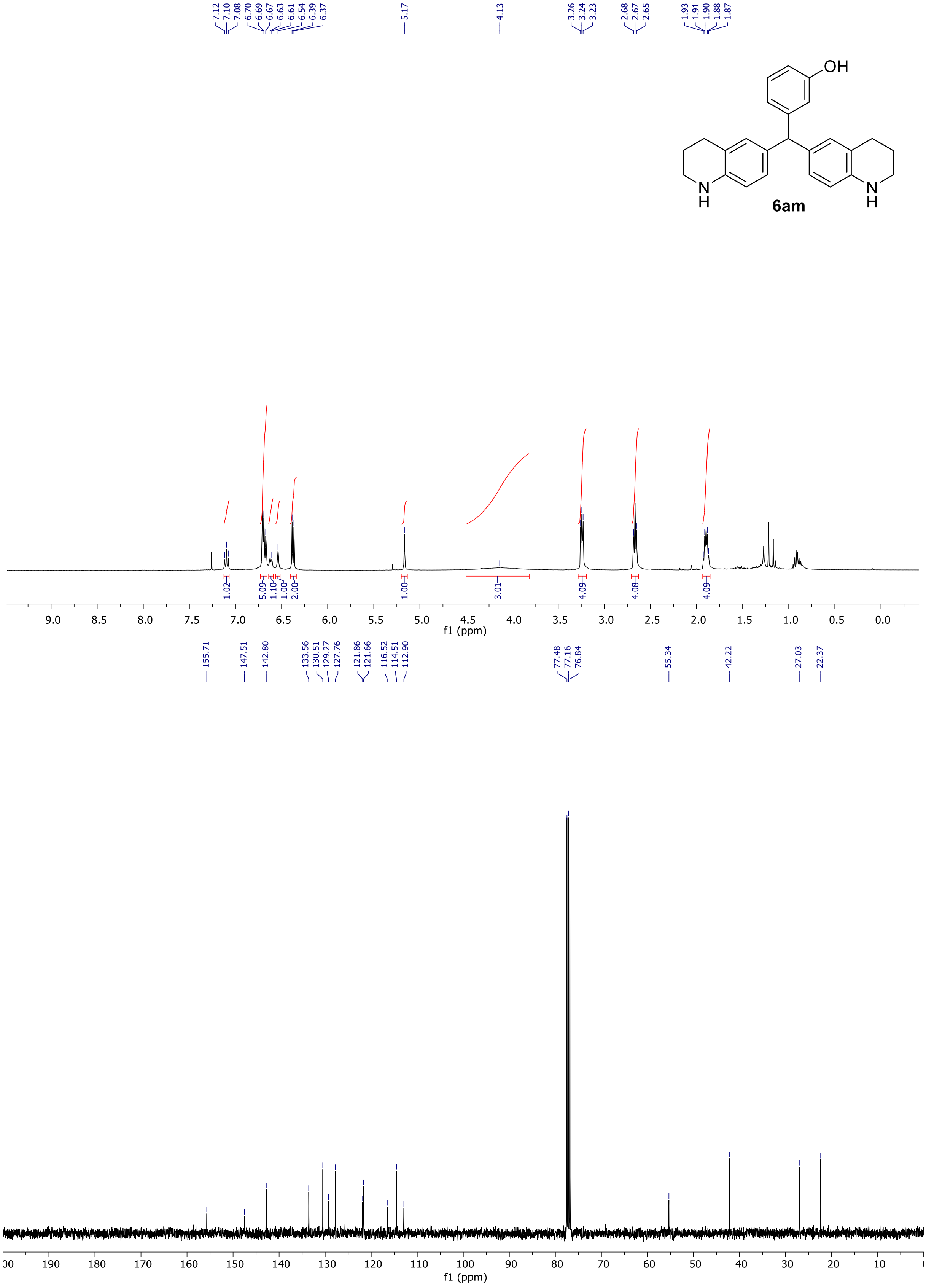

^{1}H NMR (400 MHz) and $^{13}C\{^{1}H\}$ NMR (100 MHz) spectra of **6am** ($CDCl_3$)

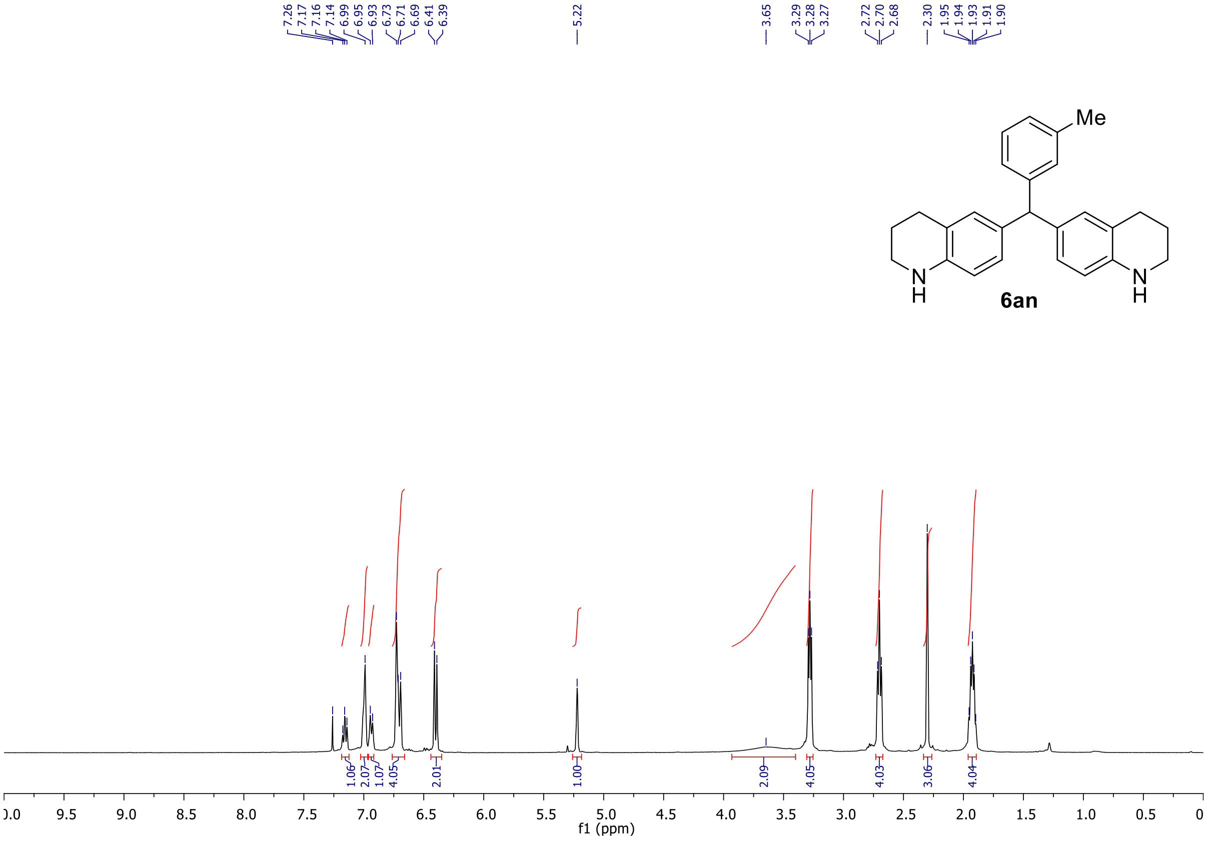

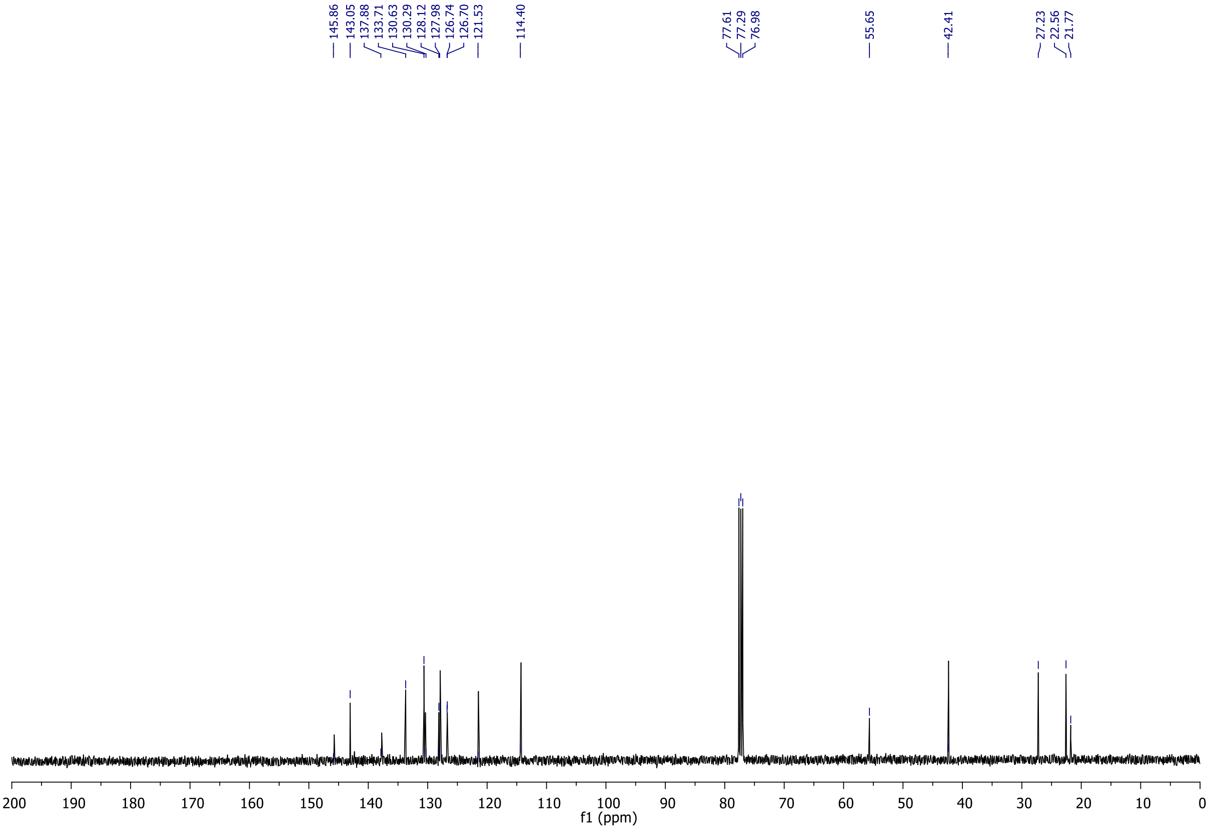

^{1}H NMR (400 MHz) and ^{13}C{^{1}H} NMR (100 MHz) spectra of **6an** ($CDCl_3$)

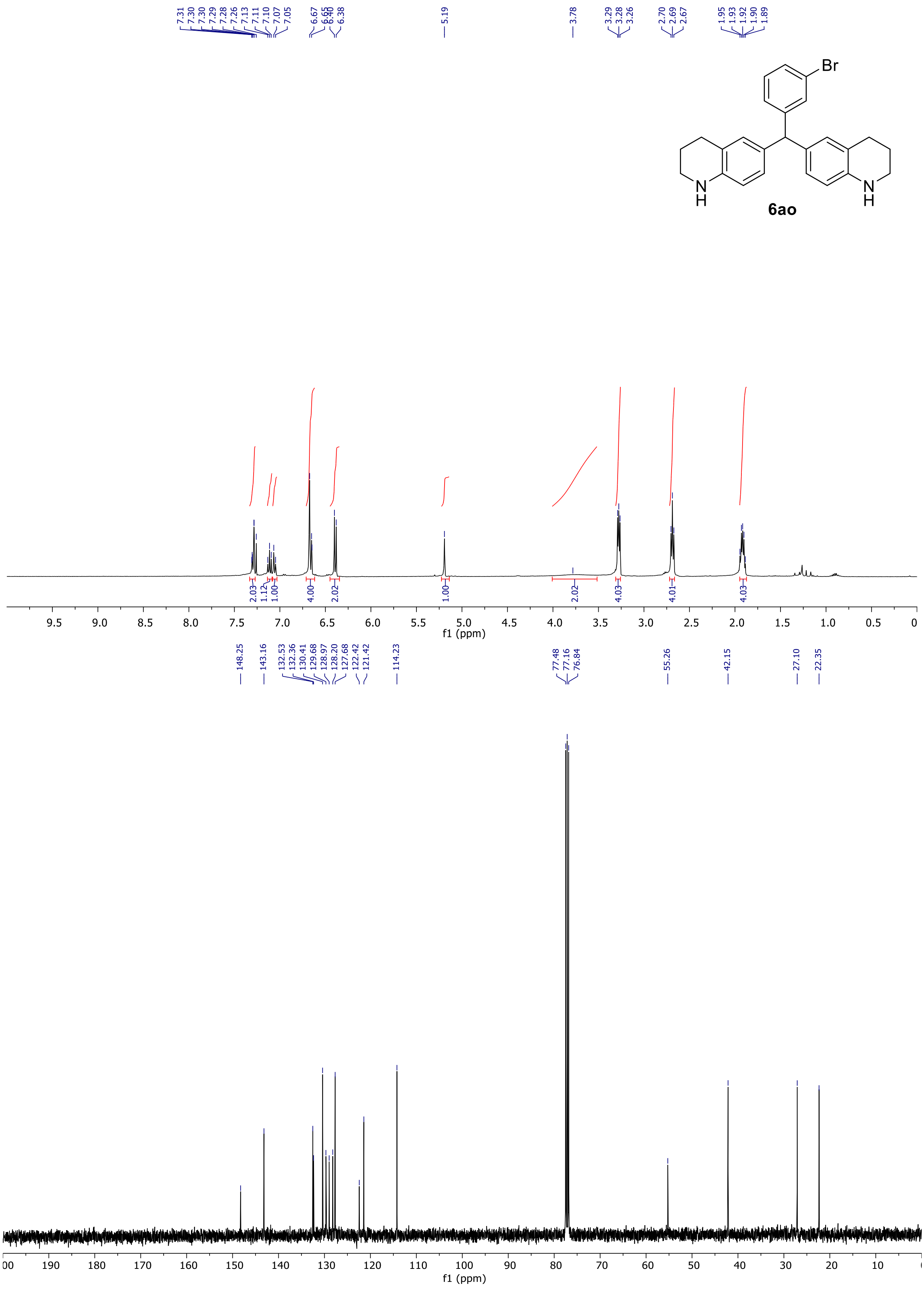

^{1}H NMR (400 MHz) and $^{13}C\{^{1}H\}$ NMR (100 MHz) spectra of **6ao** ($CDCl_3$)

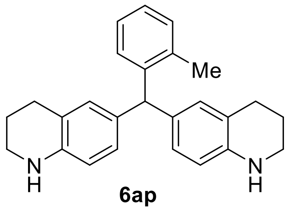

6ap

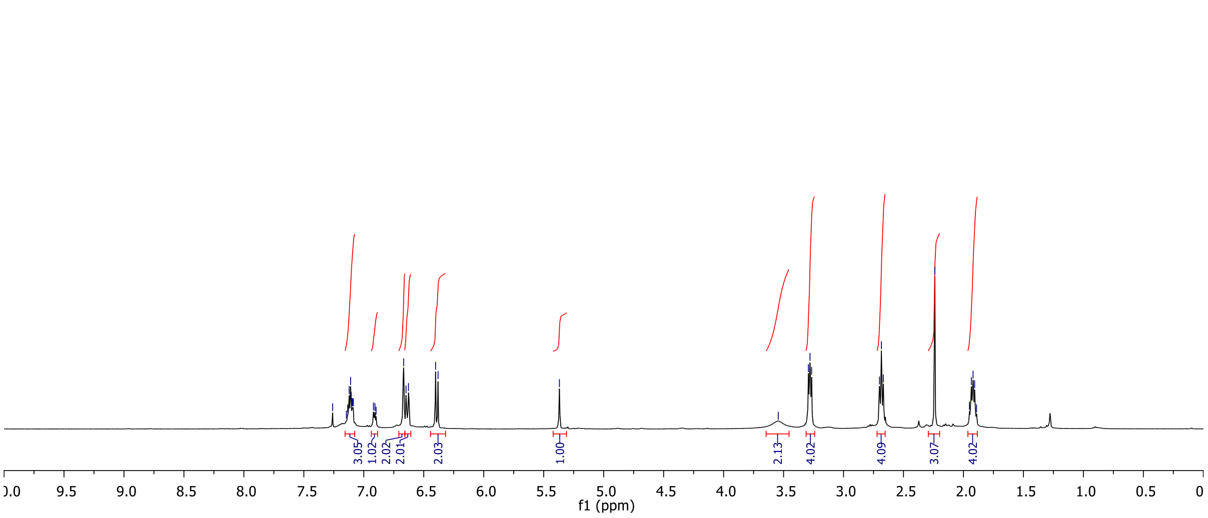

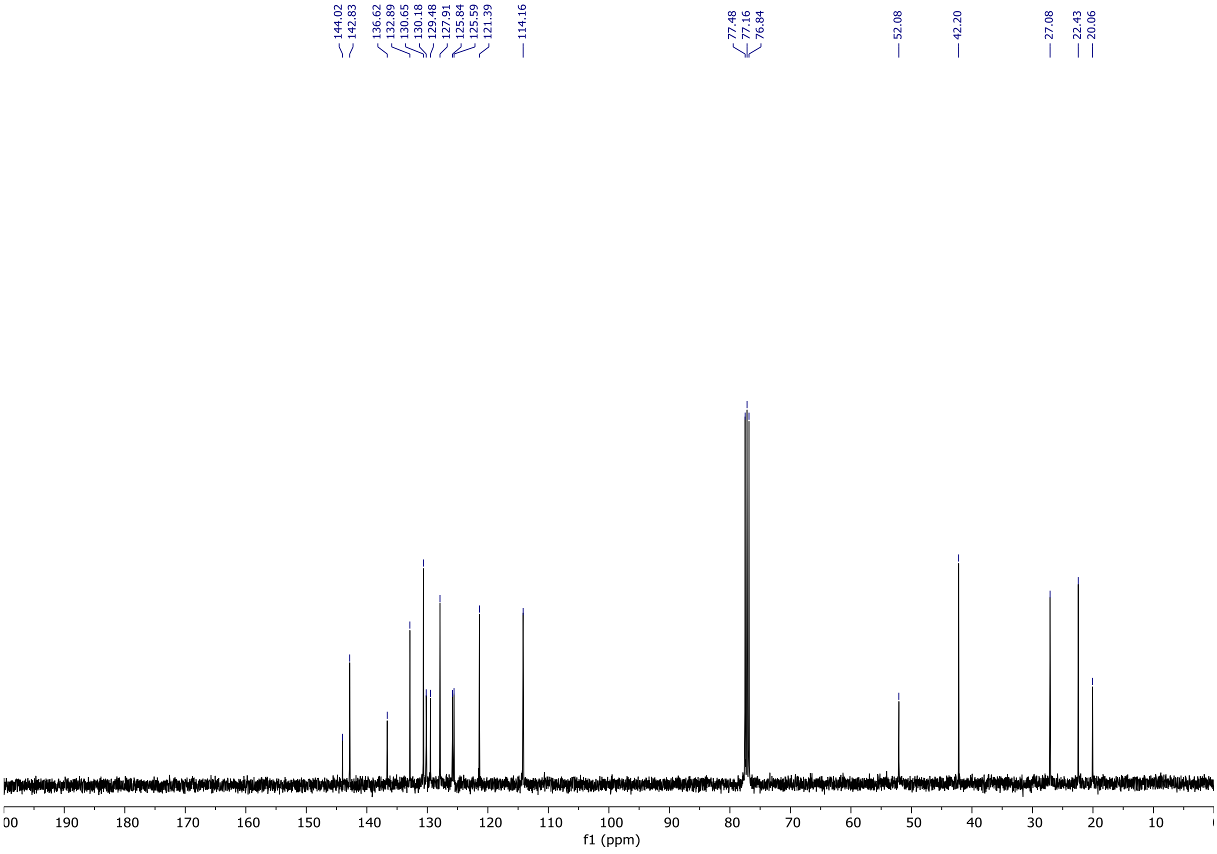

^{1}H NMR (400 MHz) and $^{13}C\{^{1}H\}$ NMR (100 MHz) spectra of **6ap** ($CDCl_3$)

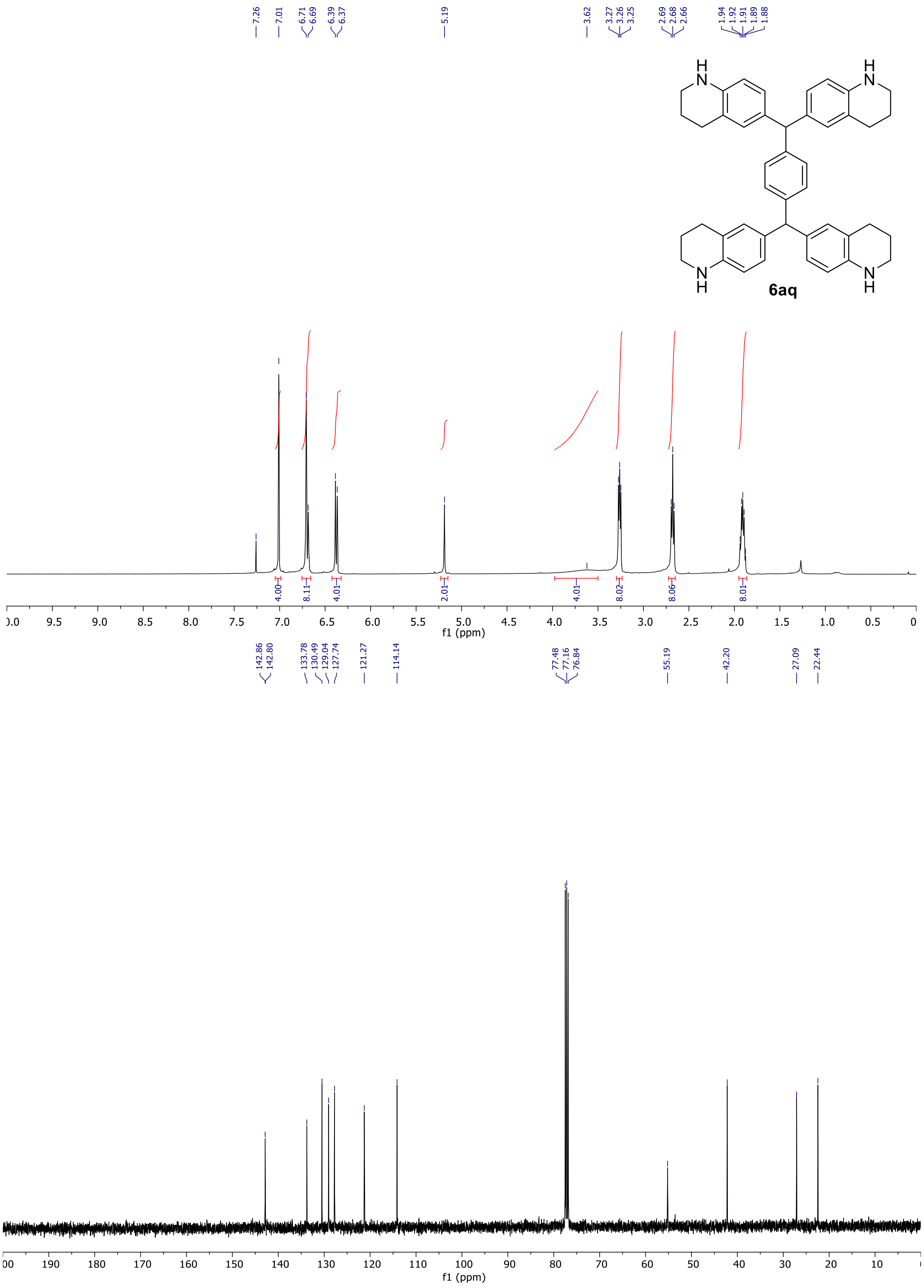

^{1}H NMR (400 MHz) and $^{13}C\{^{1}H\}$ NMR (100 MHz) spectra of **6aq** ($CDCl_3$)

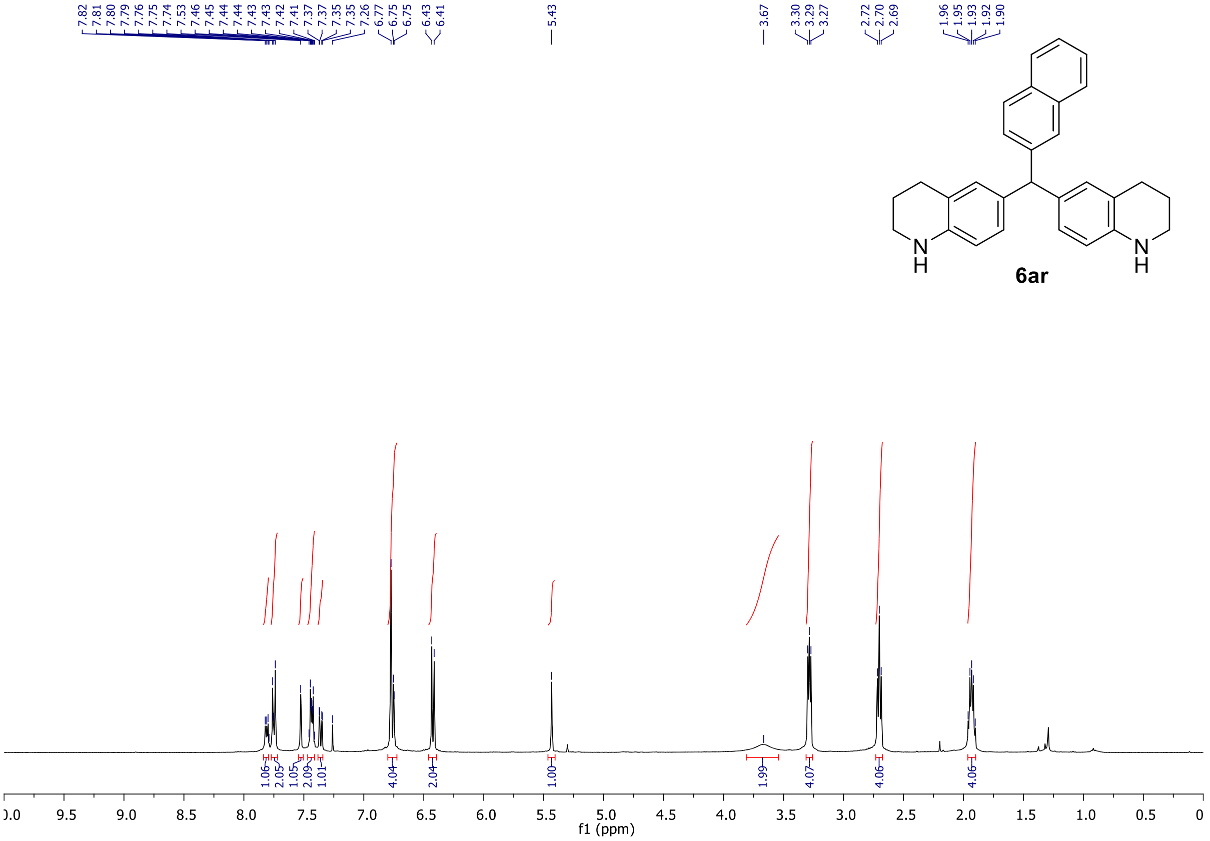

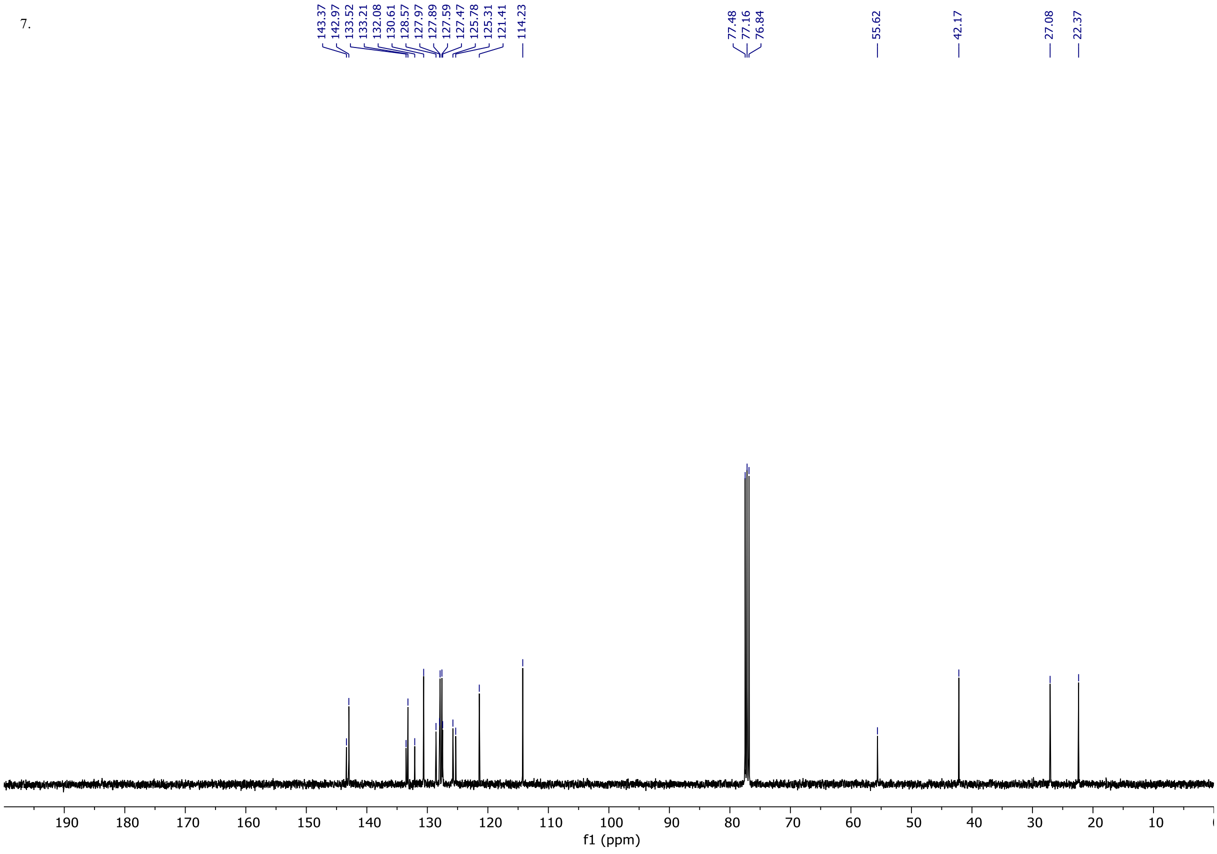

^{1}H NMR (400 MHz) and $^{13}C\{^{1}H\}$ NMR (100 MHz) spectra of **6ar** ($CDCl_3$)

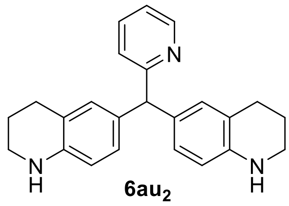

6au₂

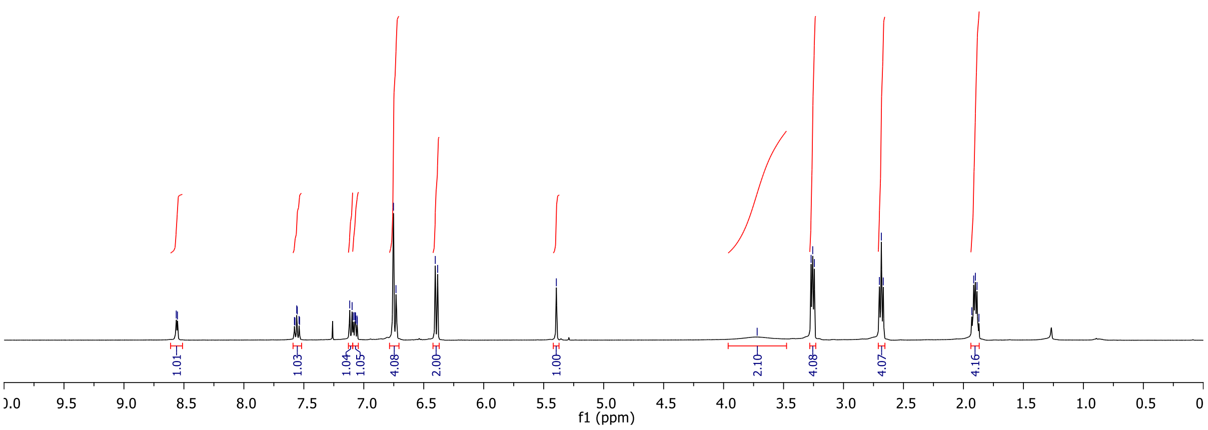

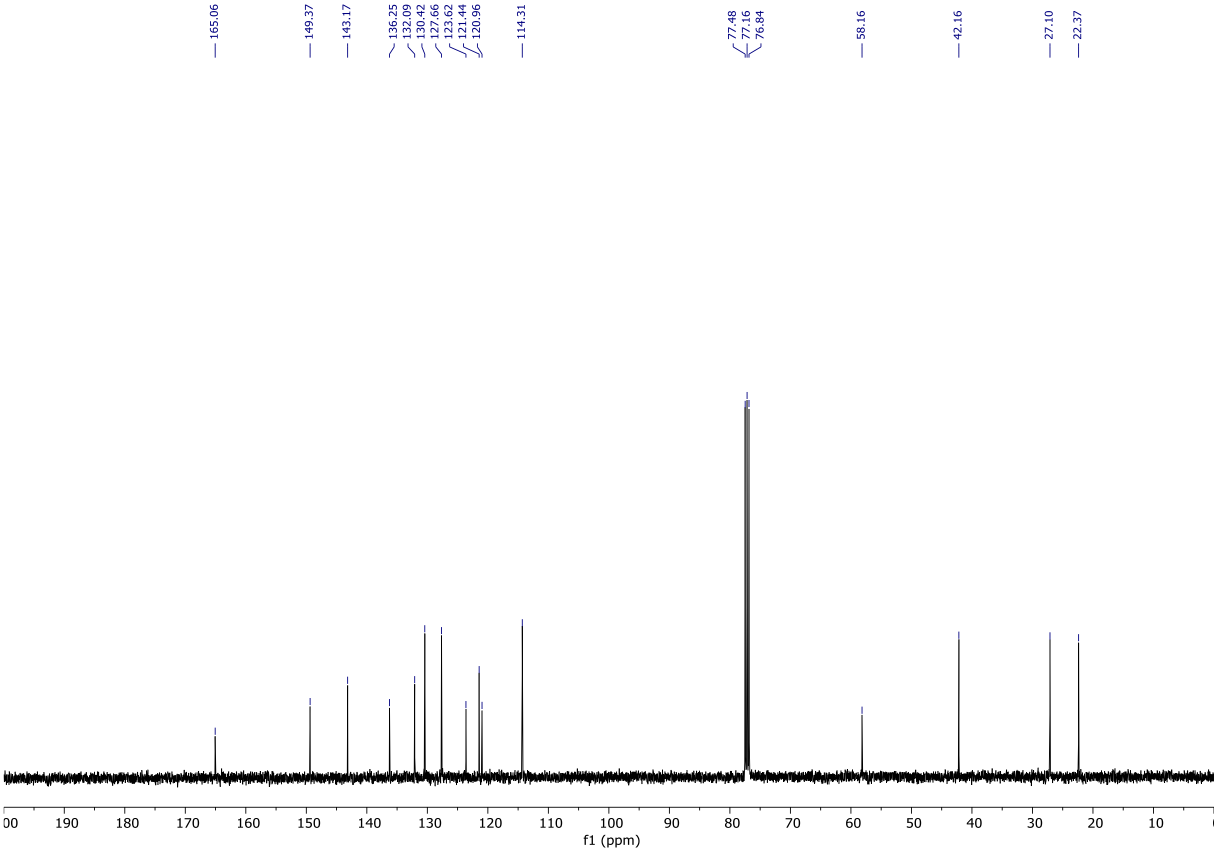

^{1}H NMR (400 MHz) and $^{13}C\{^{1}H\}$ NMR (100 MHz) spectra of **6au₂** ($CDCl_3$)

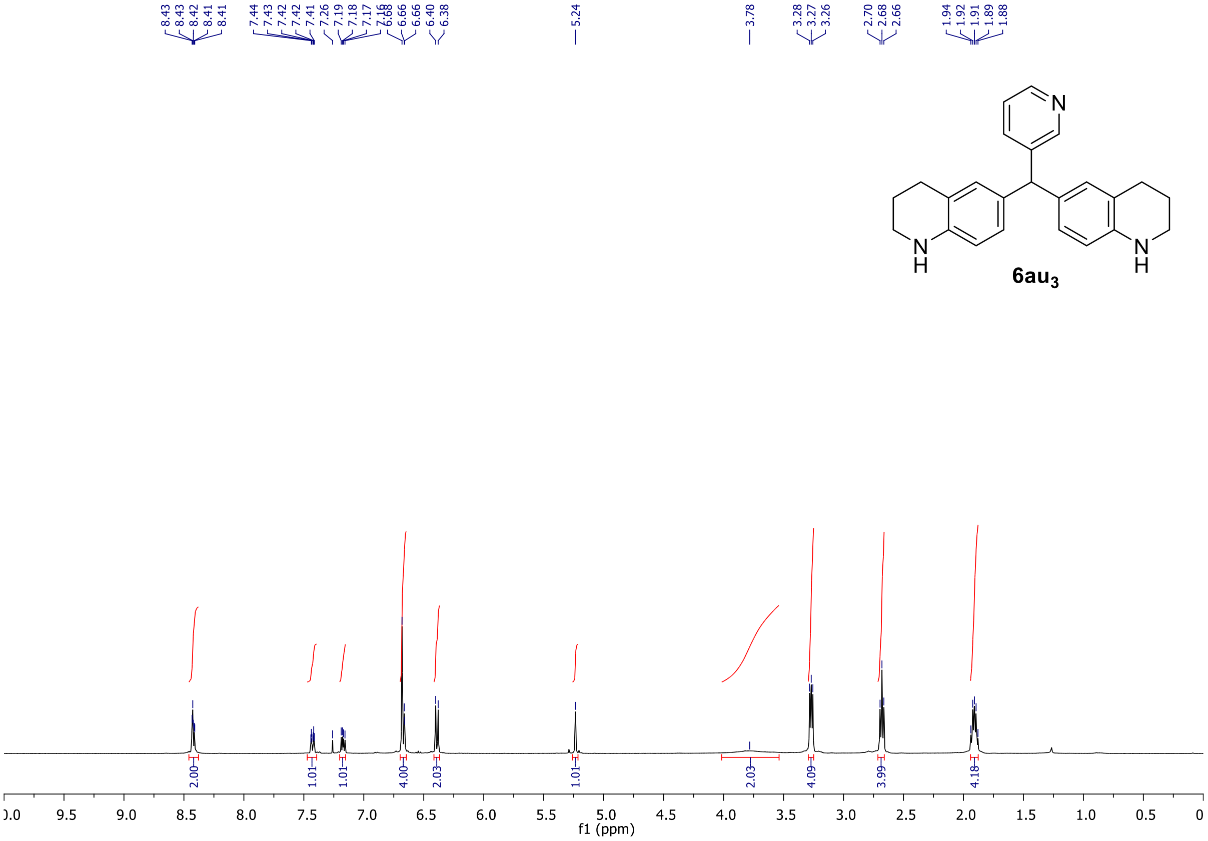

6au$_3$

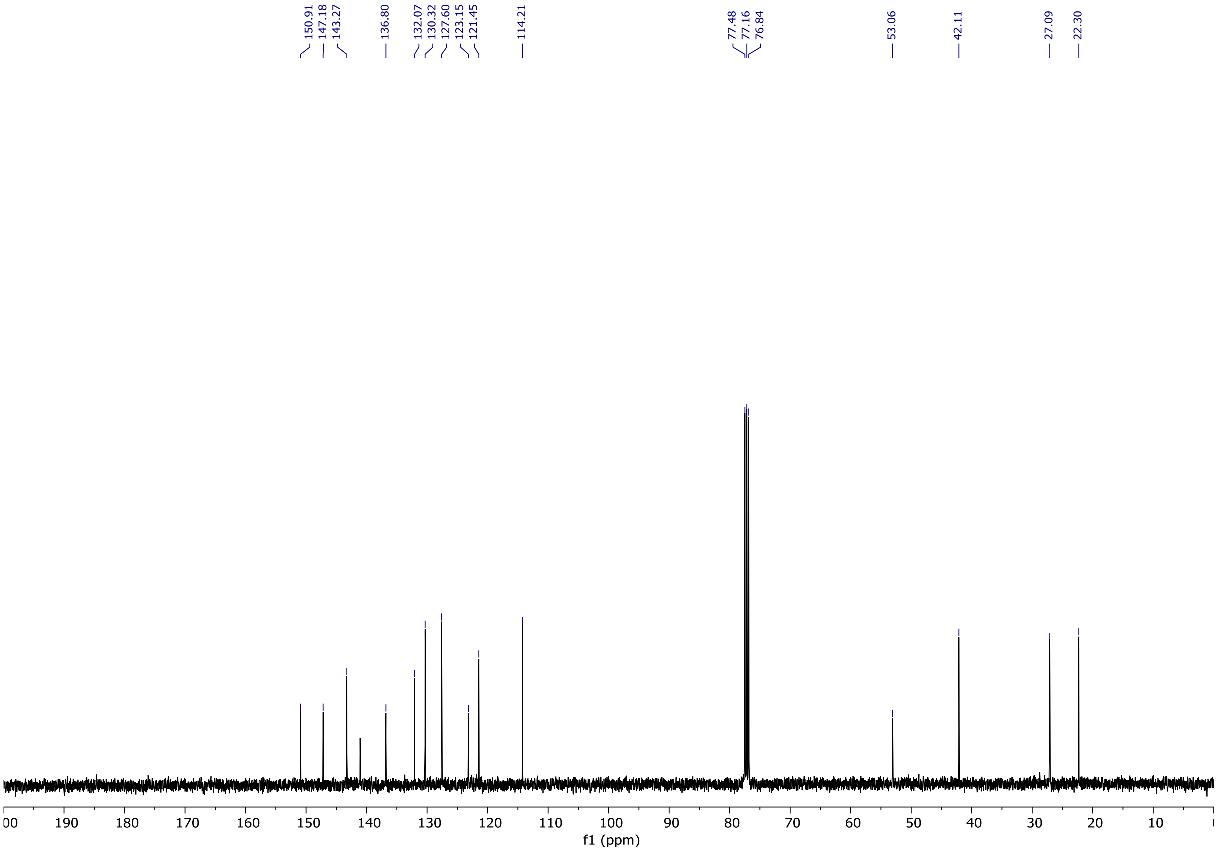

^{1}H NMR (400 MHz) and ^{13}C{^{1}H} NMR (100 MHz) spectra of **6au$_3$** (CDCl$_3$)

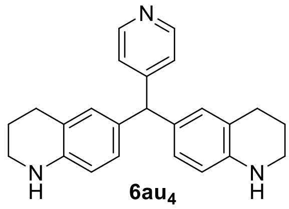

6au₄

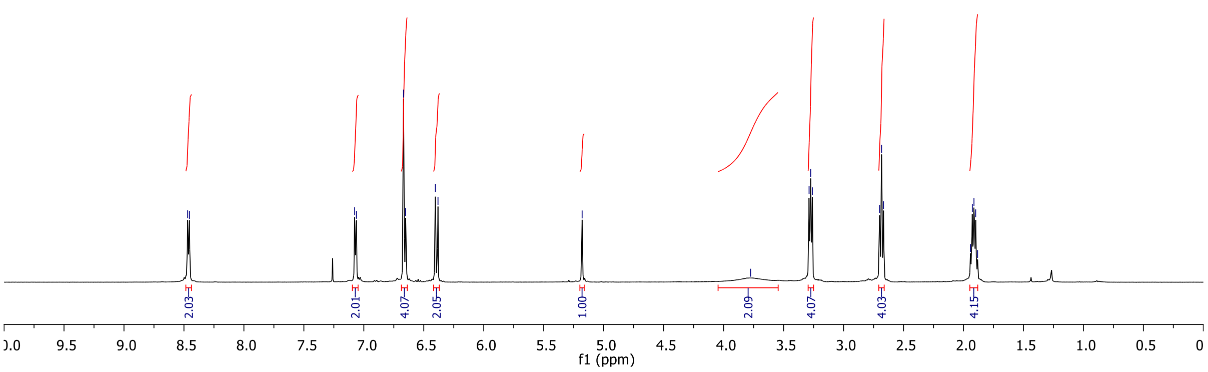

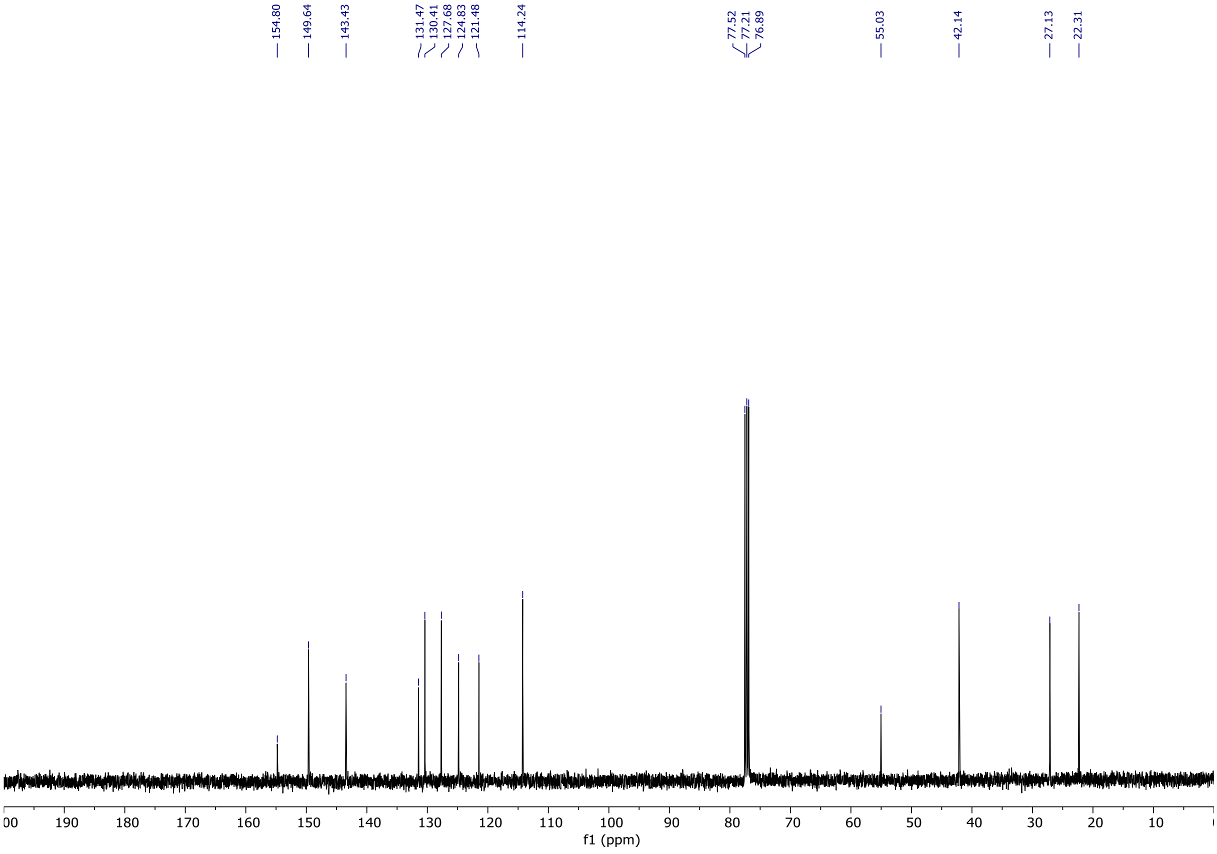

^{1}H NMR (400 MHz) and $^{13}C\{^{1}H\}$ NMR (100 MHz) spectra of **6au$_4$** ($CDCl_3$)

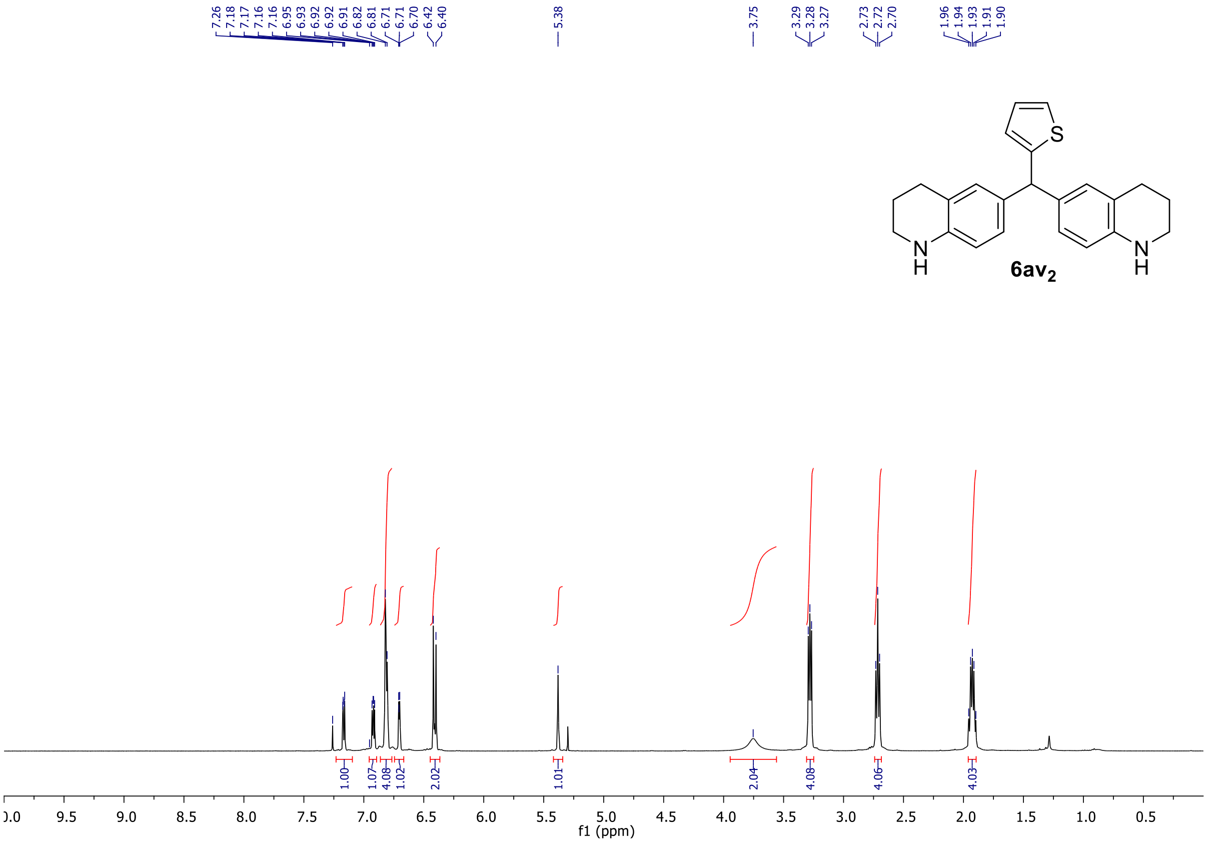

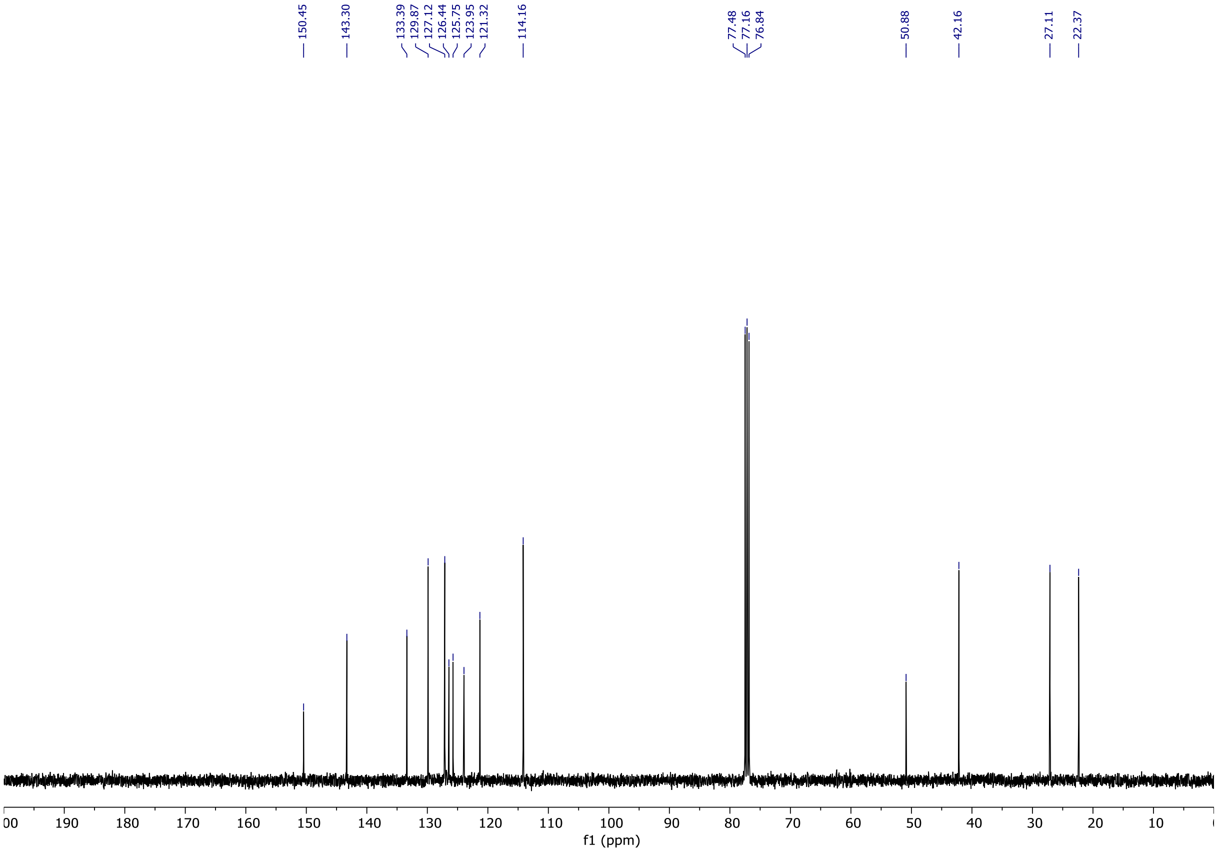

^{1}H NMR (400 MHz) and $^{13}C\{^{1}H\}$ NMR (100 MHz) spectra of **6av$_2$** ($CDCl_3$)

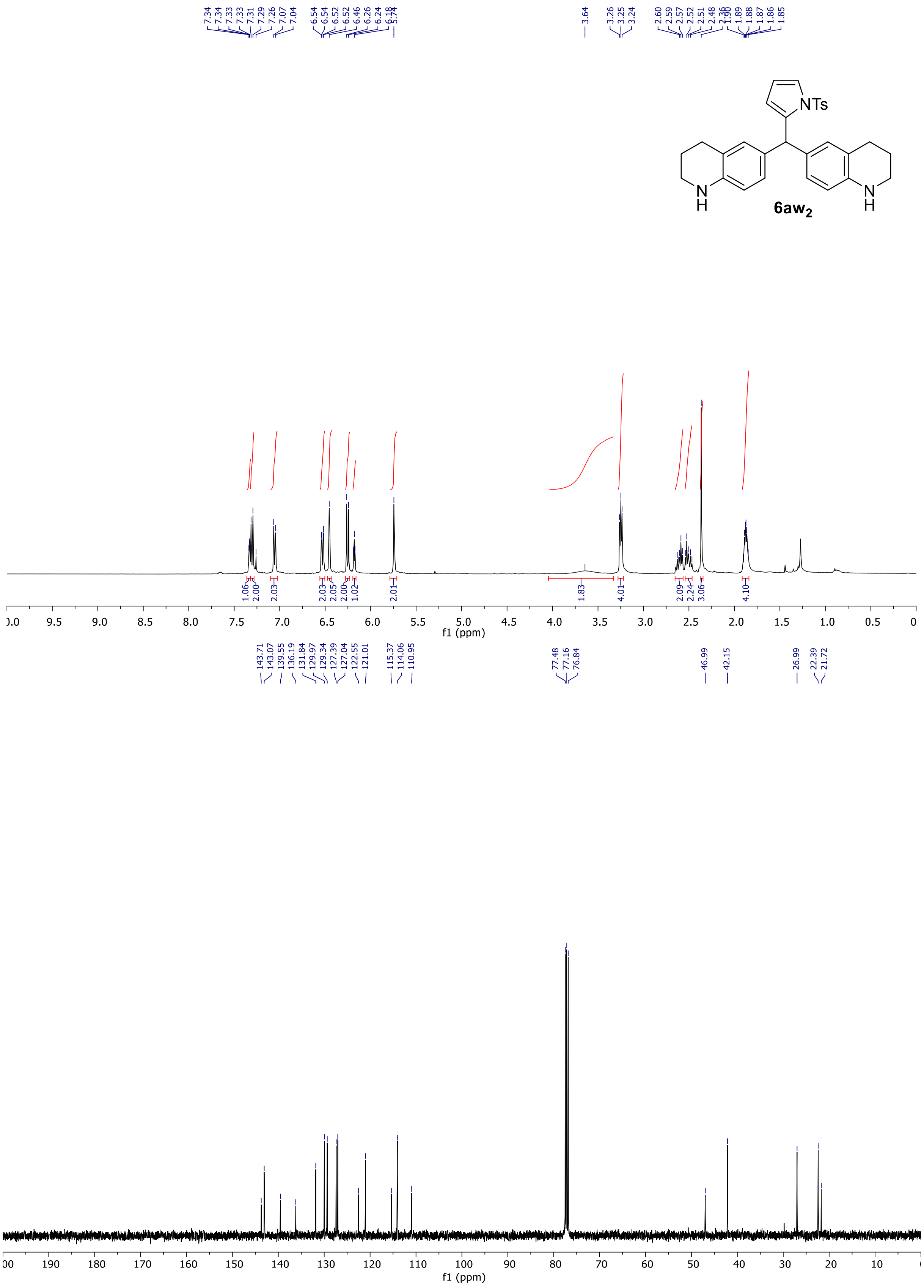

^{1}H NMR (400 MHz) and ^{13}C{^{1}H} NMR (100 MHz) spectra of **6aw$_2$** ($CDCl_3$)

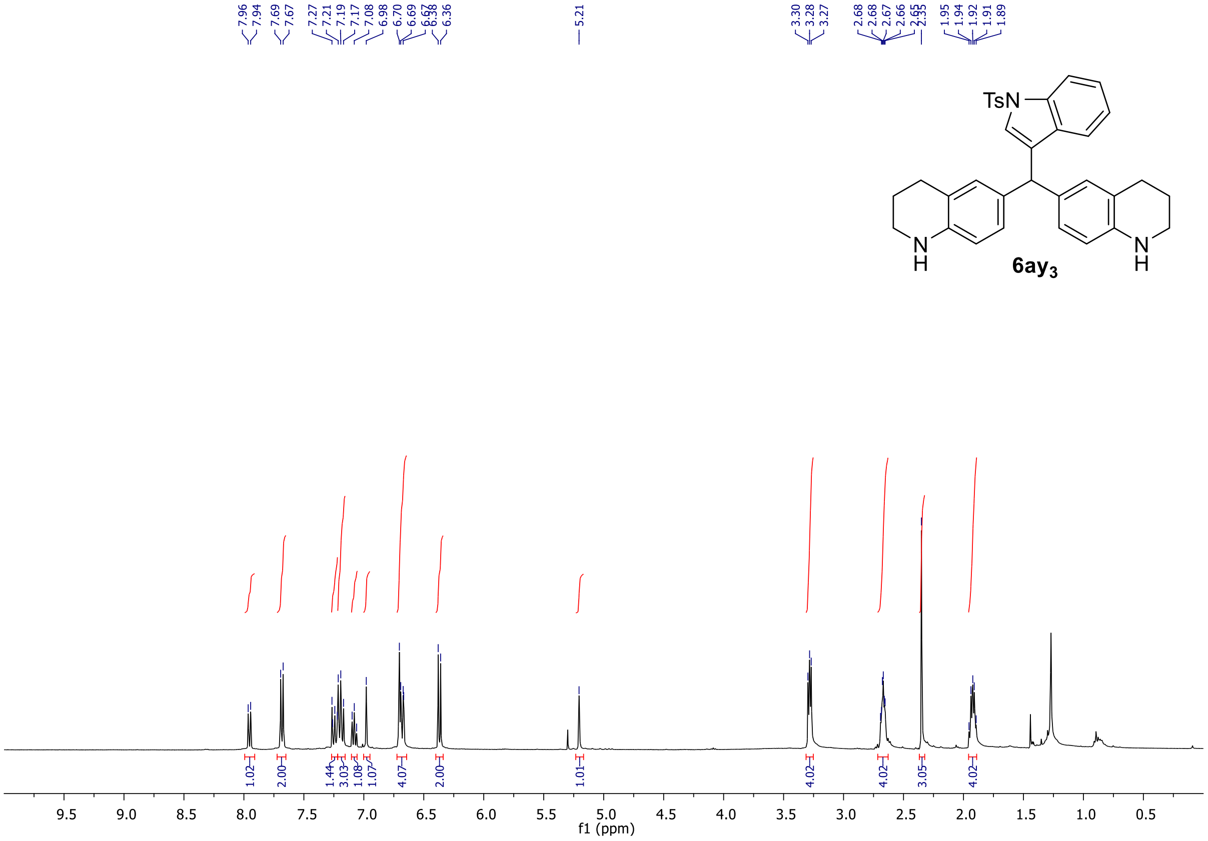

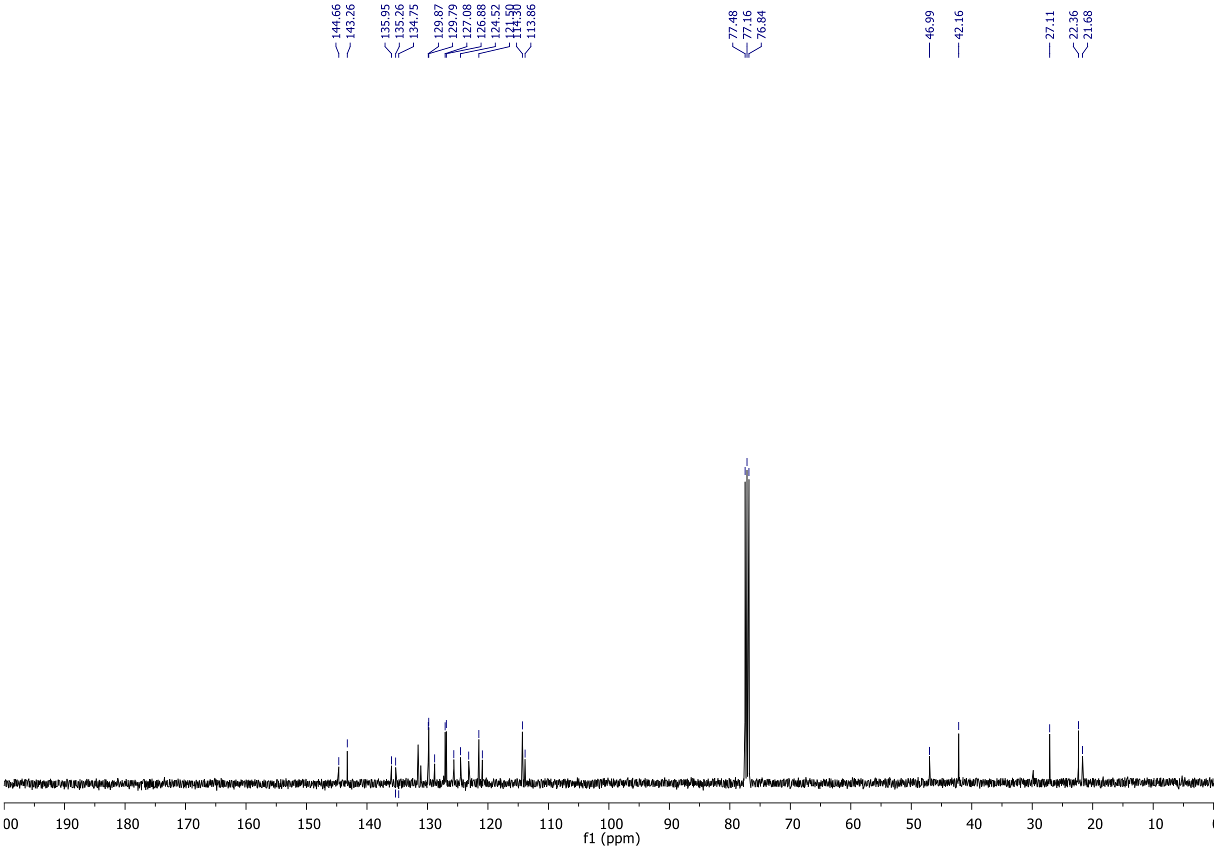

^{1}H NMR (400 MHz) and $^{13}C\{^{1}H\}$ NMR (100 MHz) spectra of **6ay$_3$** ($CDCl_3$)

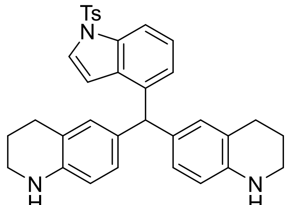

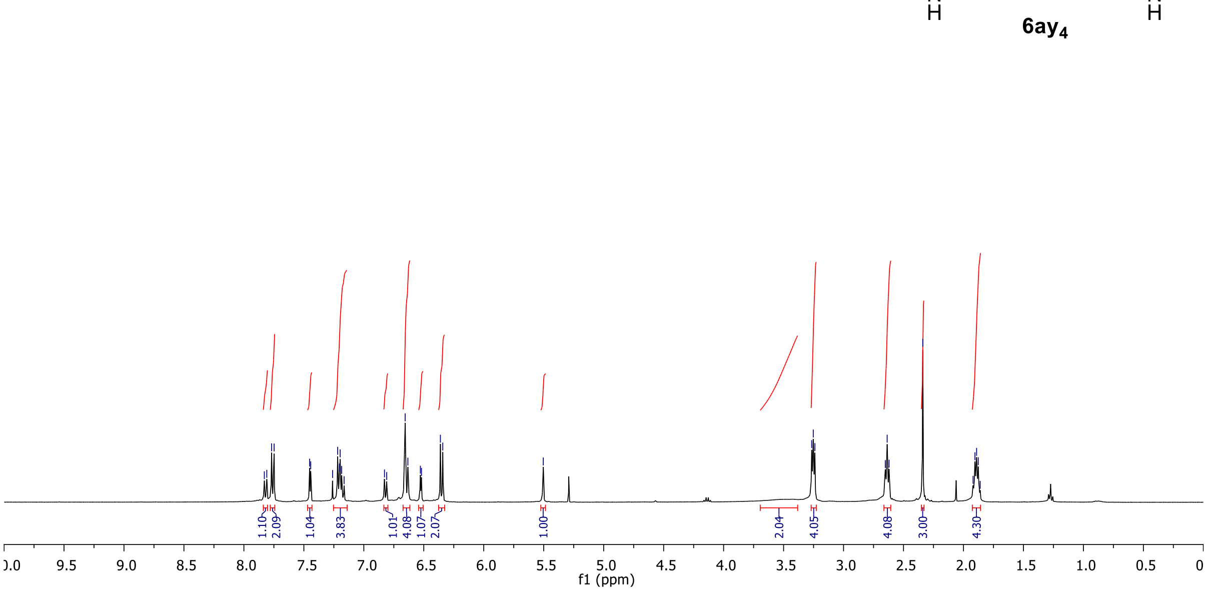

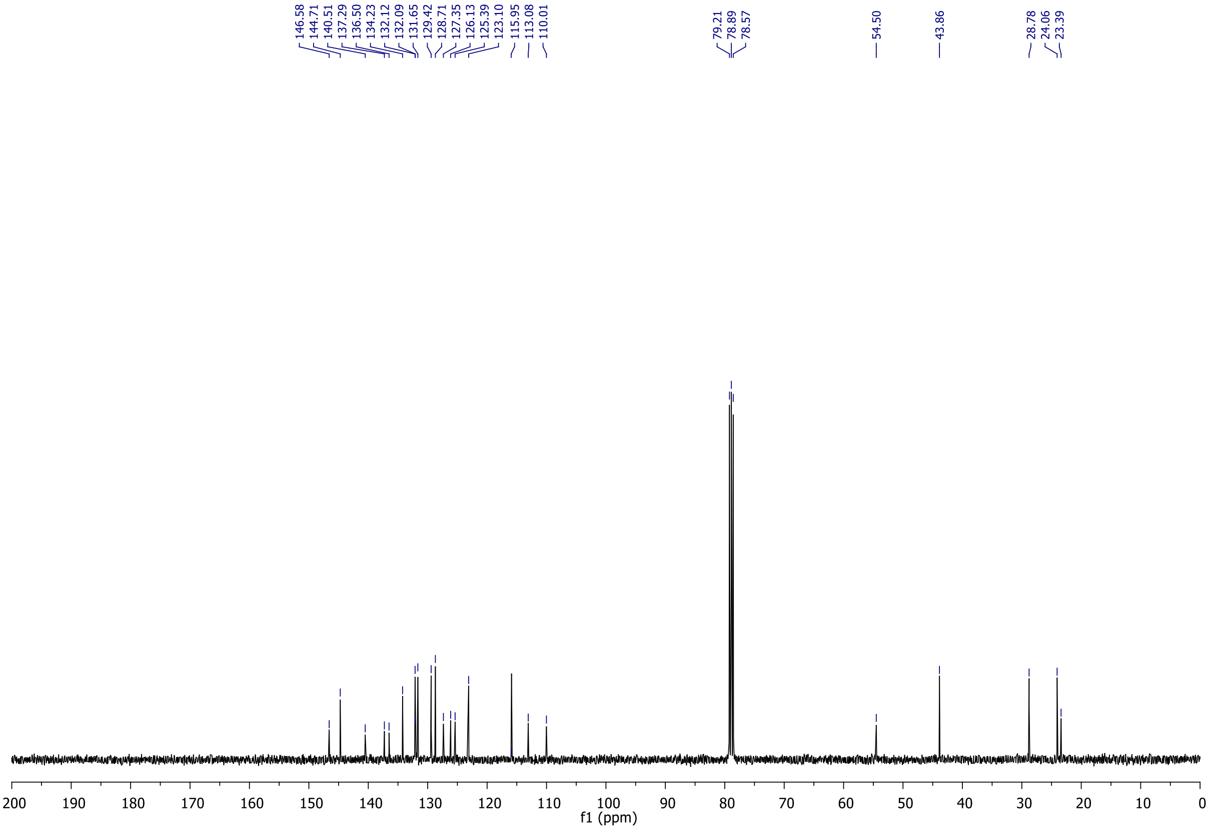

^{1}H NMR (400 MHz) and $^{13}C\{^{1}H\}$ NMR (100 MHz) spectra of **6ay$_4$** ($CDCl_3$)

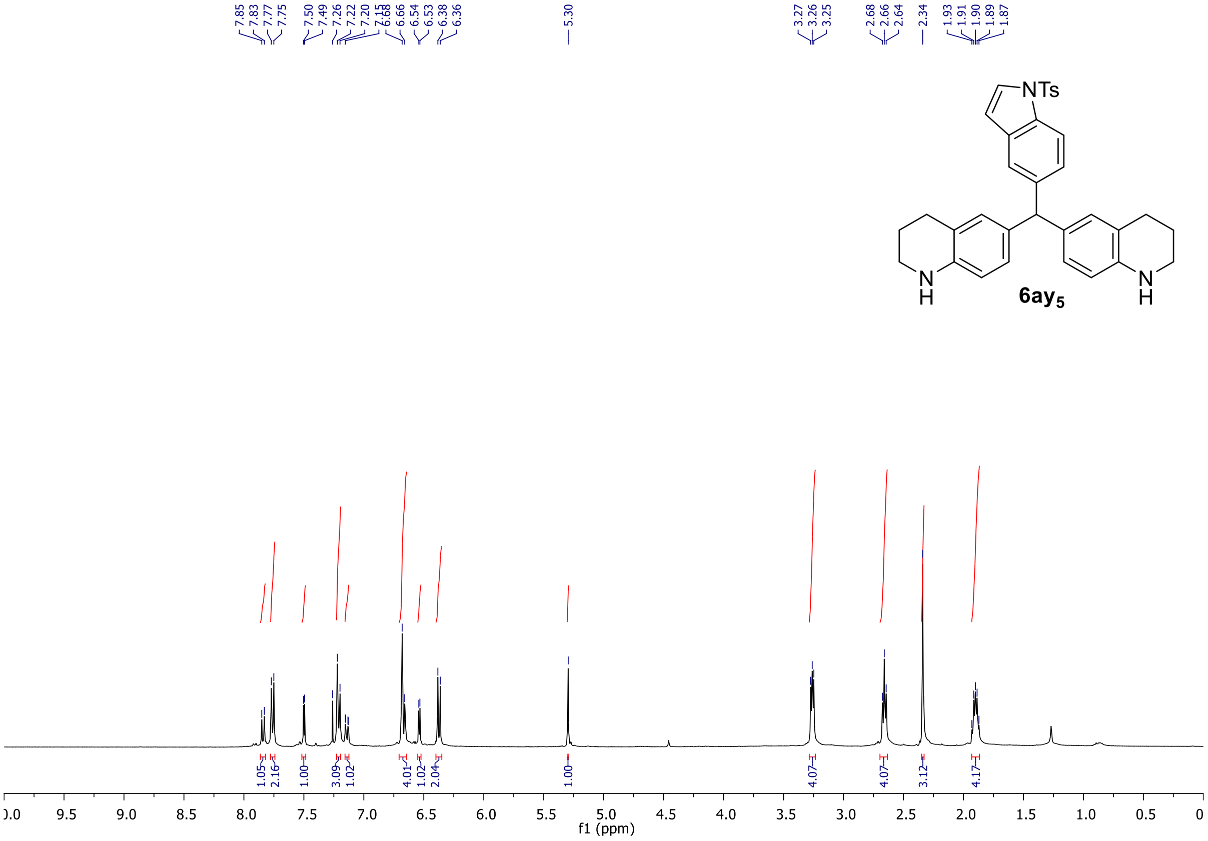

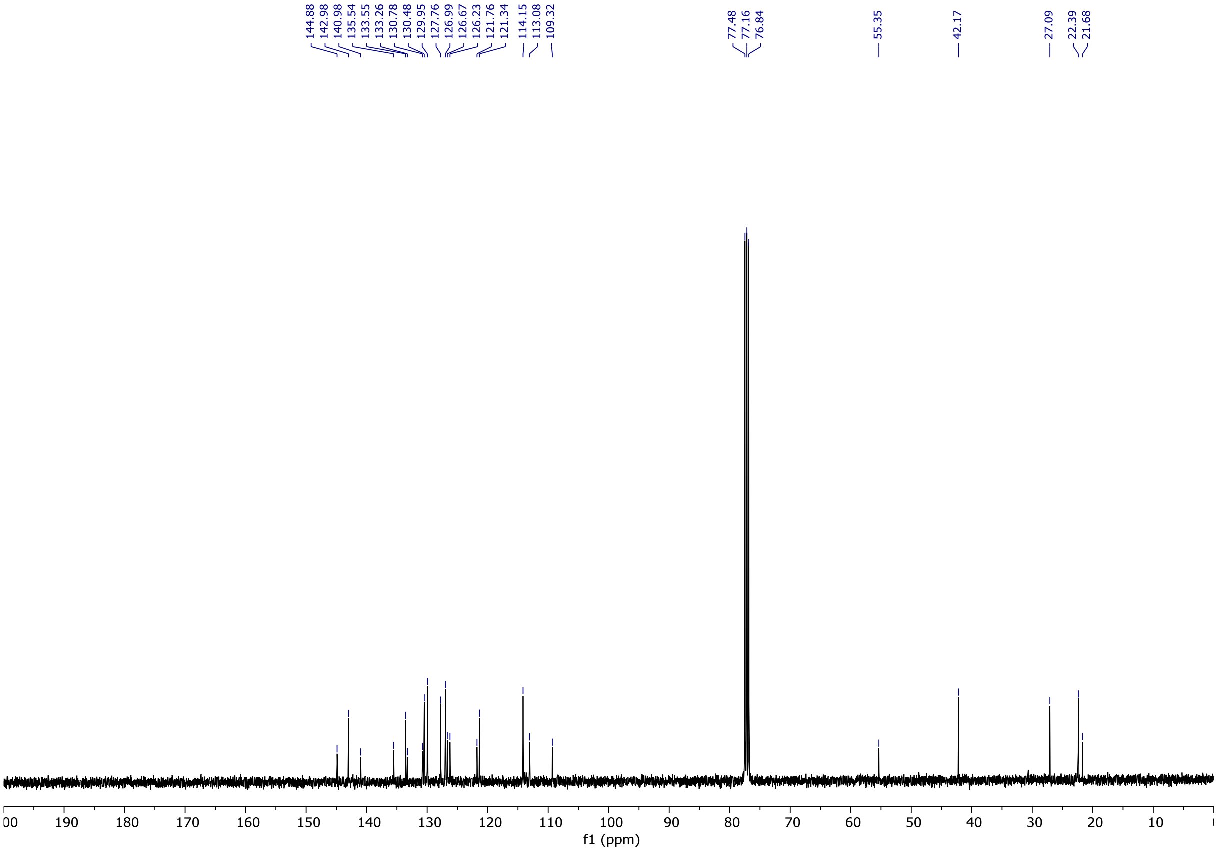

^{1}H NMR (400 MHz) and ^{13}C{^{1}H} NMR (100 MHz) spectra of **6ay$_5$** ($CDCl_3$)

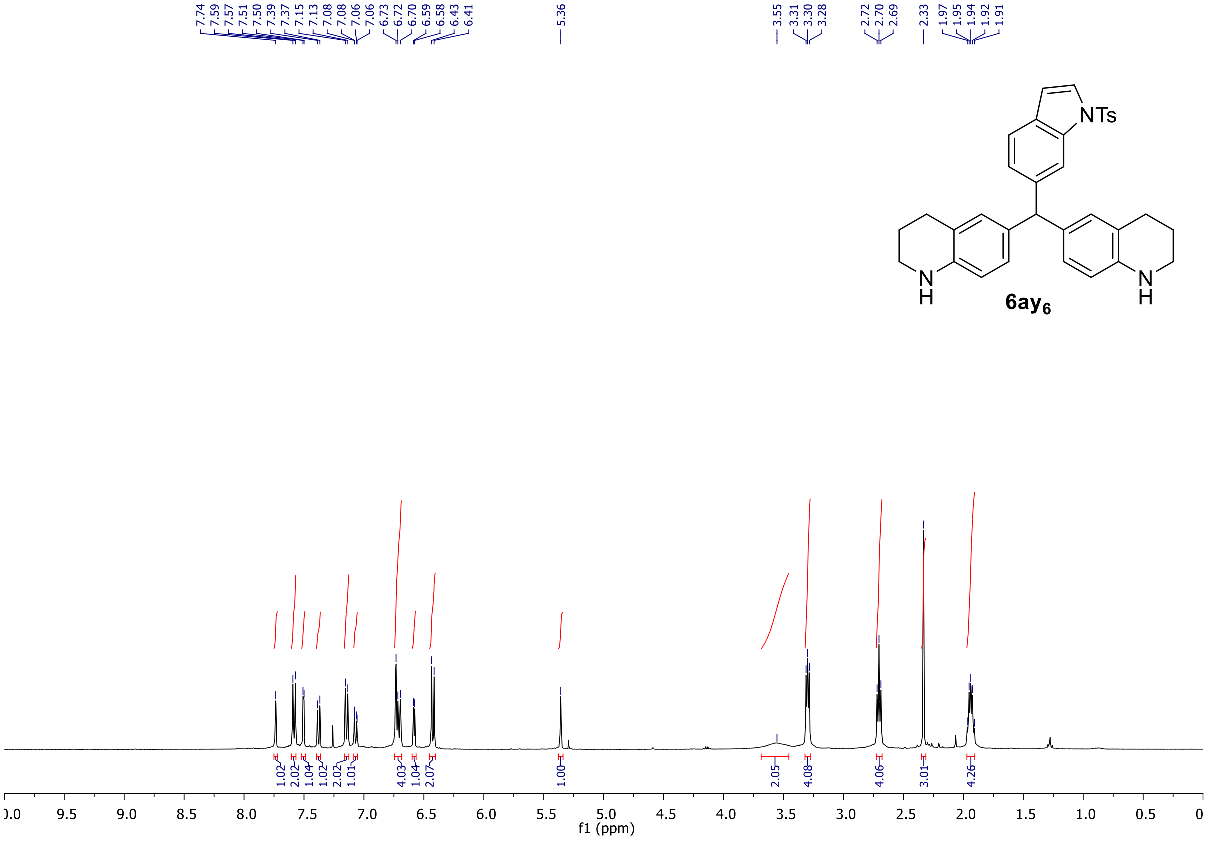

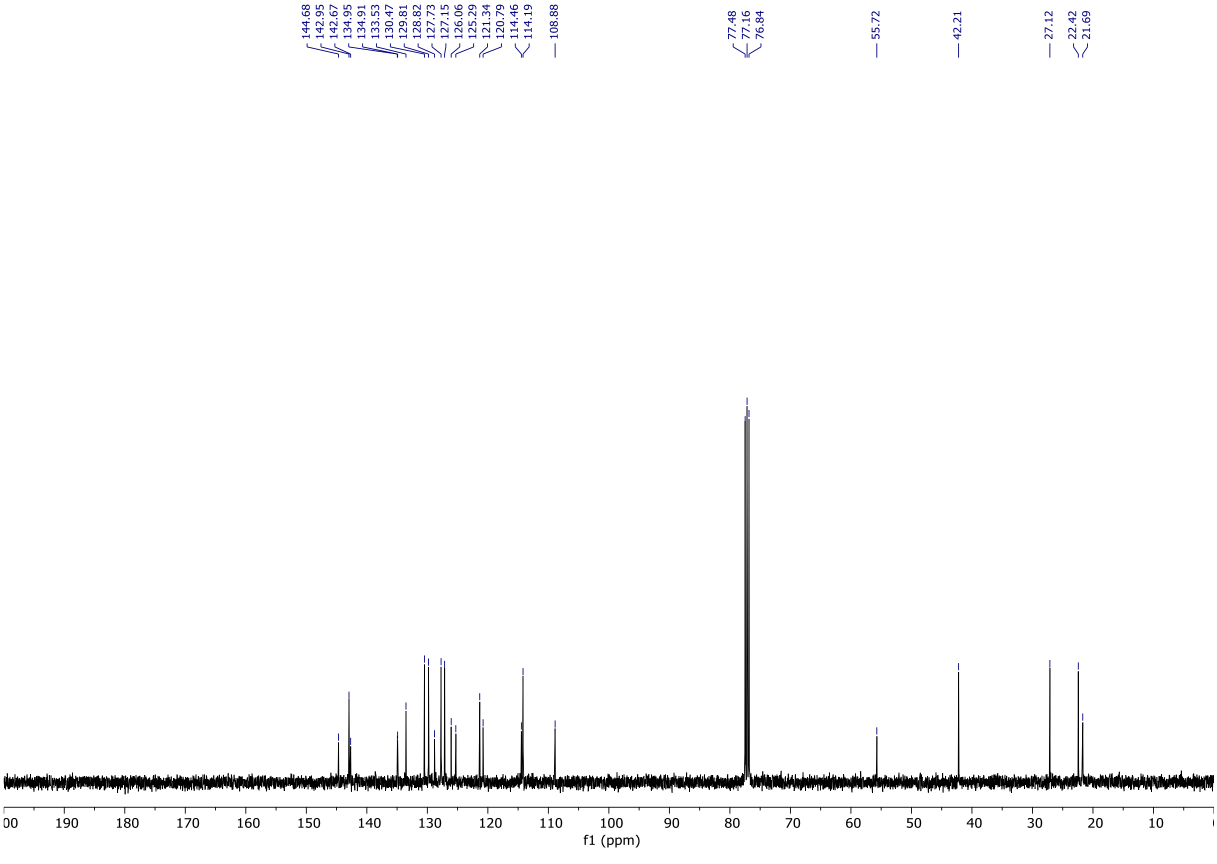

^{1}H NMR (400 MHz) and ^{13}C{^{1}H} NMR (100 MHz) spectra of **6ay$_6$** (CDCl$_3$)

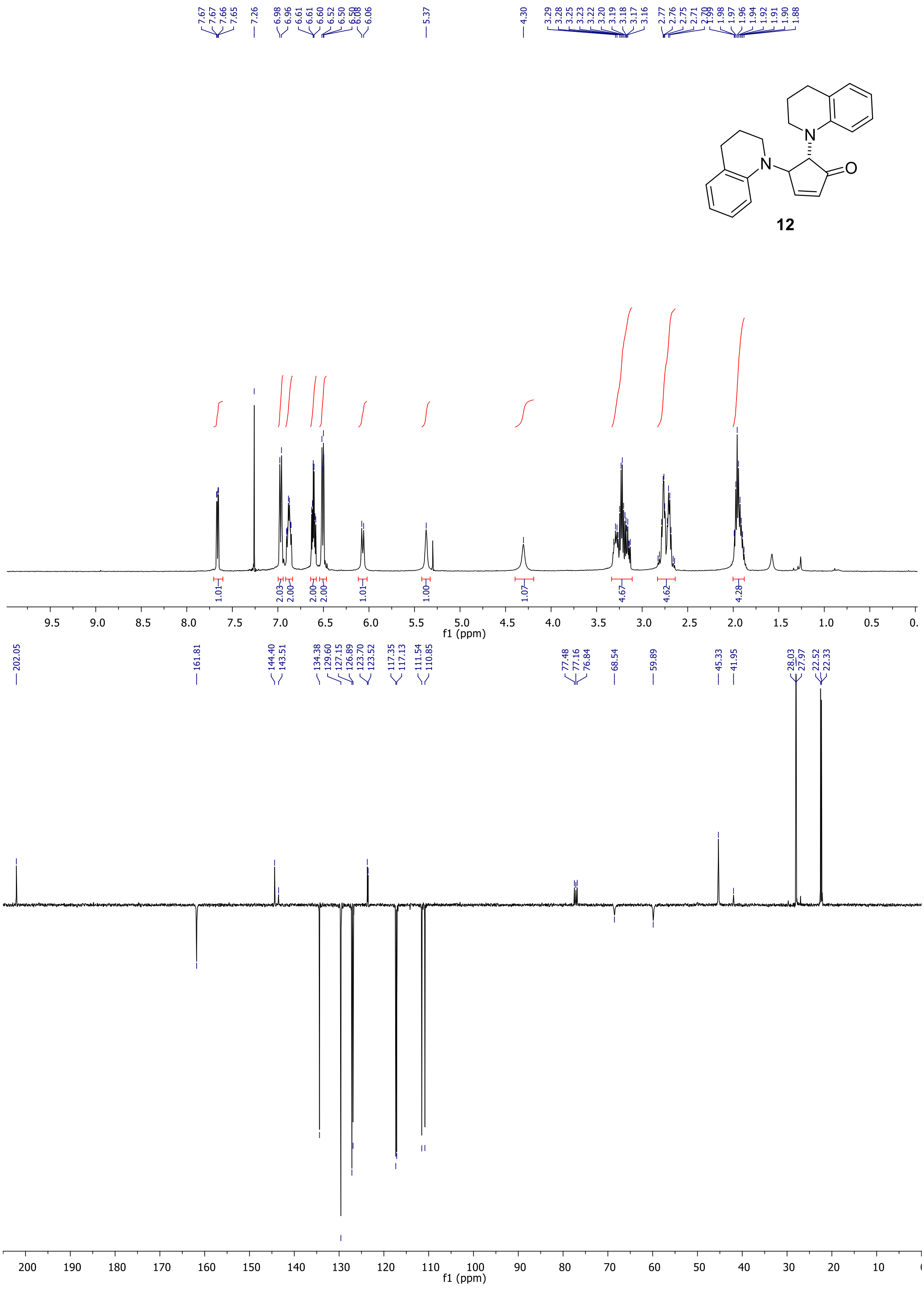

^{1}H NMR (400 MHz) and ^{13}C{^{1}H}-APT-NMR (100 MHz) spectra of **12** ($CDCl_3$)

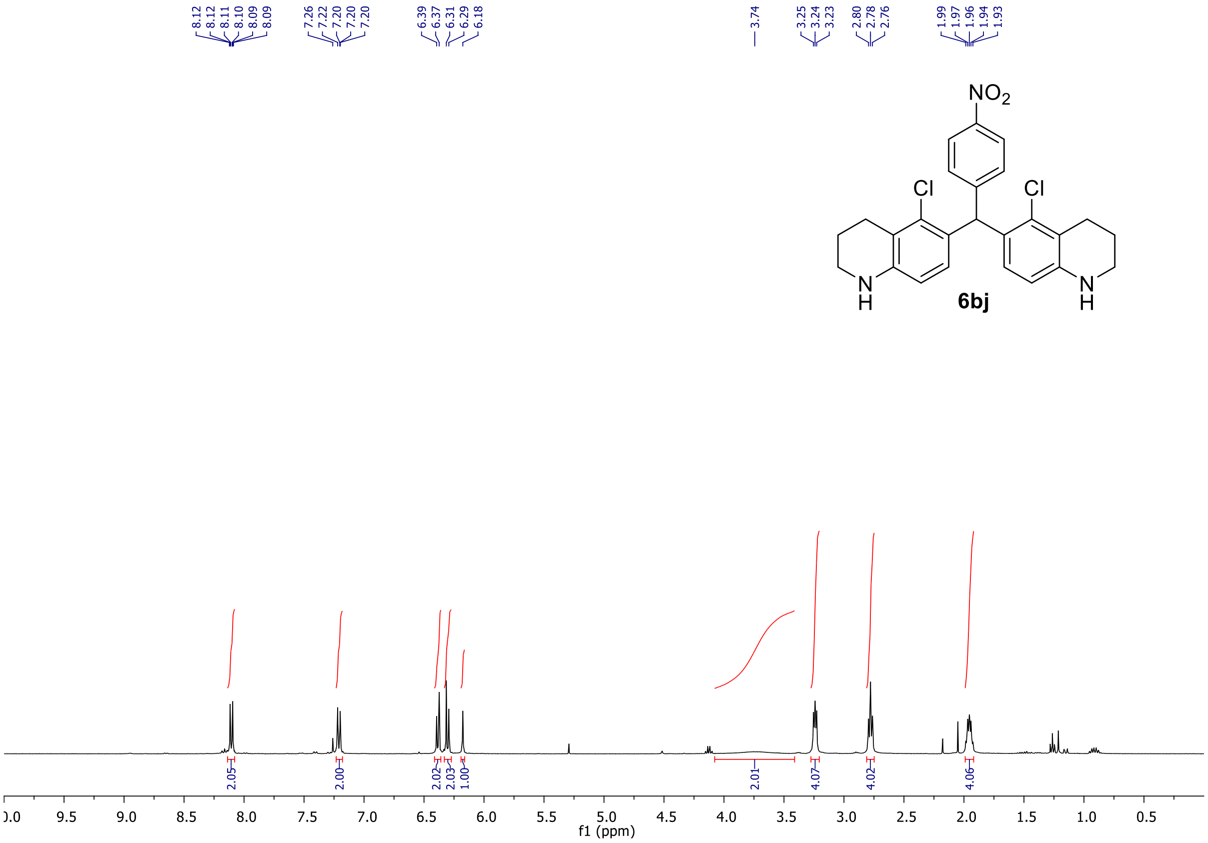

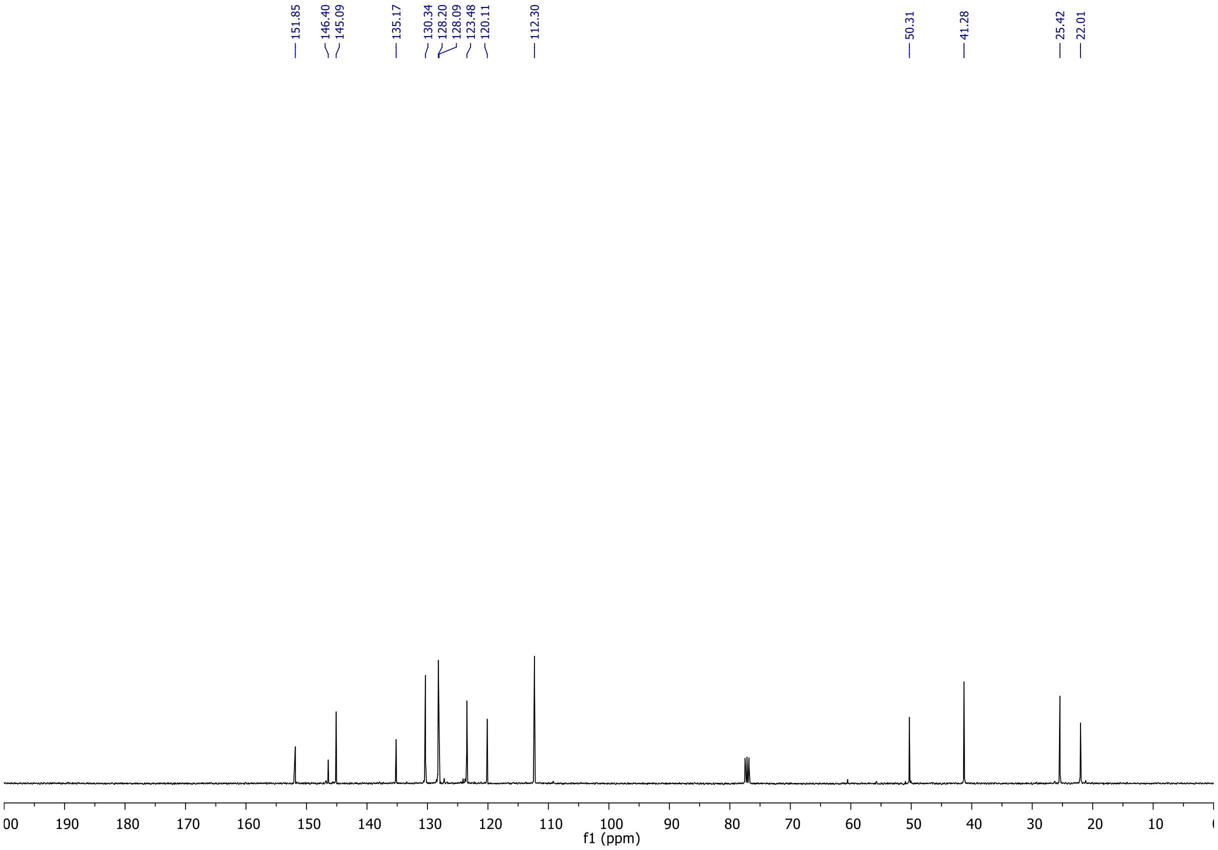

^{1}H NMR (400 MHz) and $^{13}C\{^{1}H\}$ NMR (100 MHz) spectra of **6bj** ($CDCl_3$)

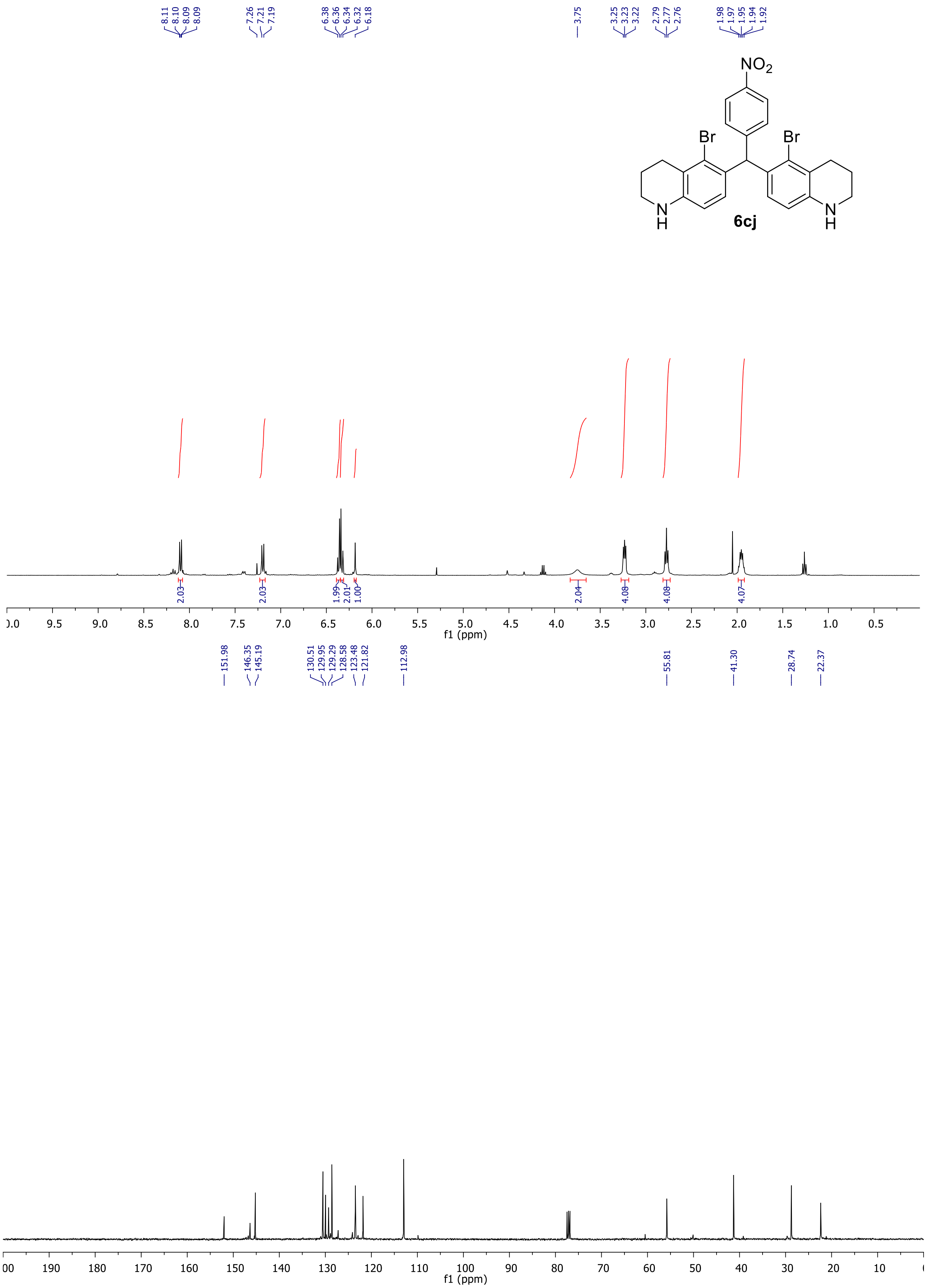

^{1}H NMR (400 MHz) and $^{13}C\{^{1}H\}$ NMR (100 MHz) spectra of **6cj** ($CDCl_3$)

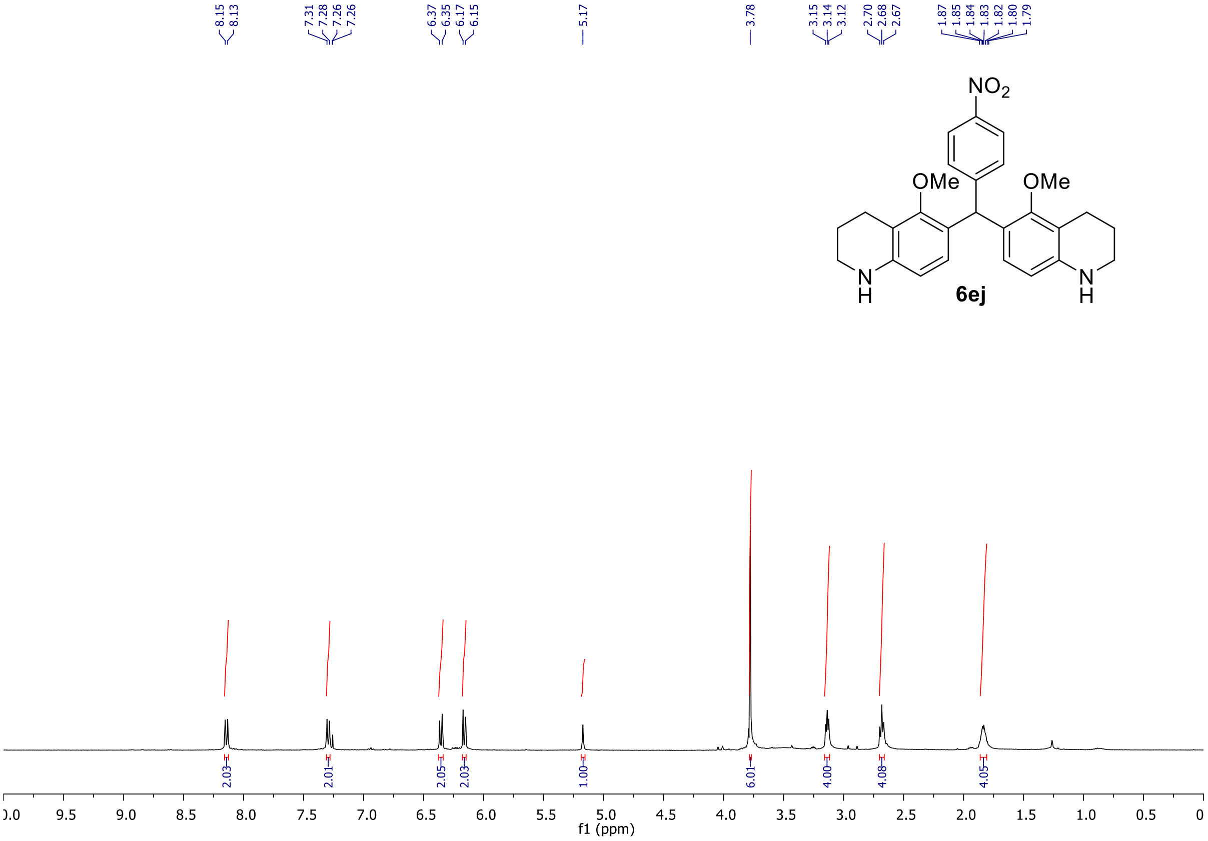

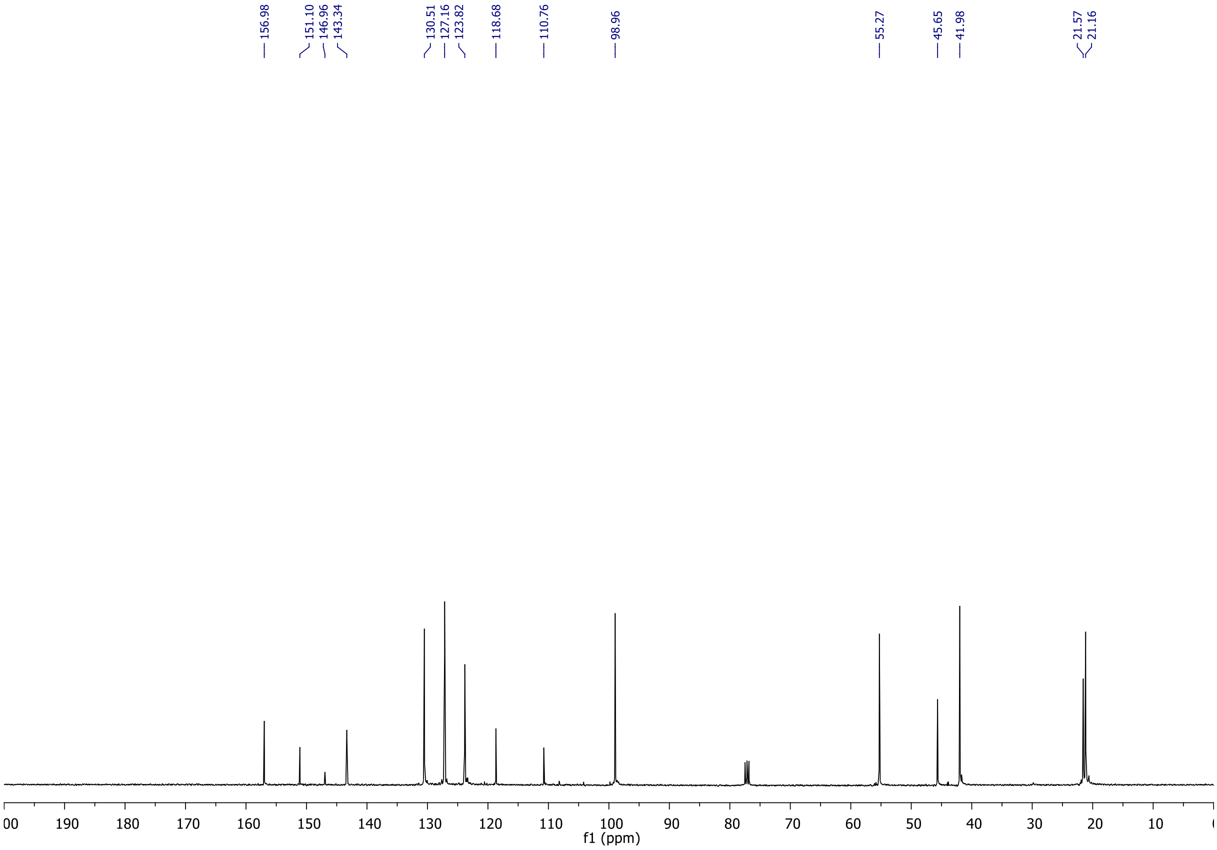

^{1}H NMR (400 MHz) and ^{13}C{^{1}H} NMR (100 MHz) spectra of **6ej** ($CDCl_3$)

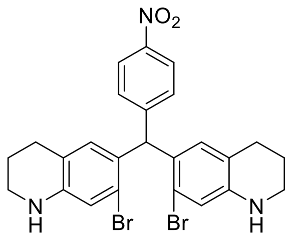

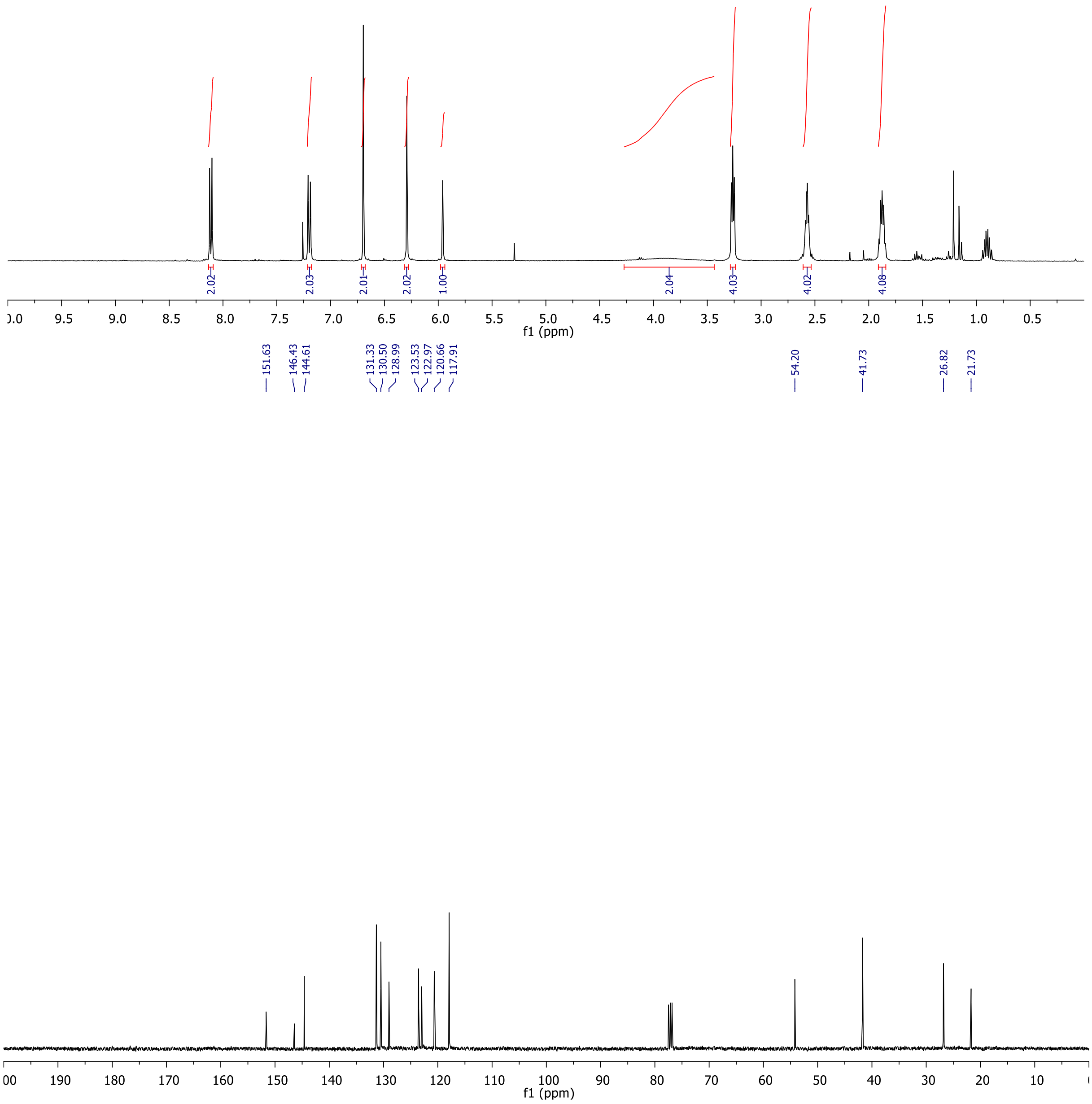

^{1}H NMR (400 MHz) and ^{13}C{^{1}H} NMR (100 MHz) spectra of **6gj** ($CDCl_3$)

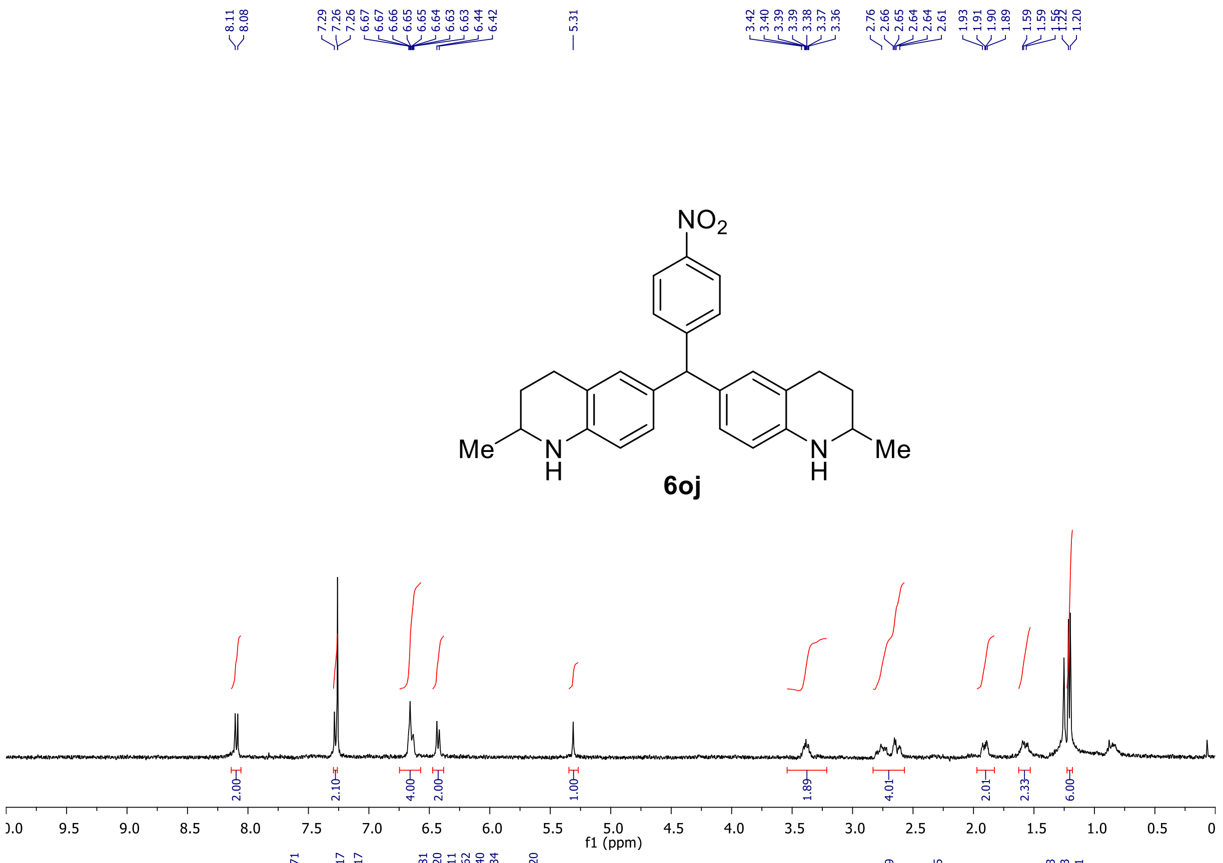

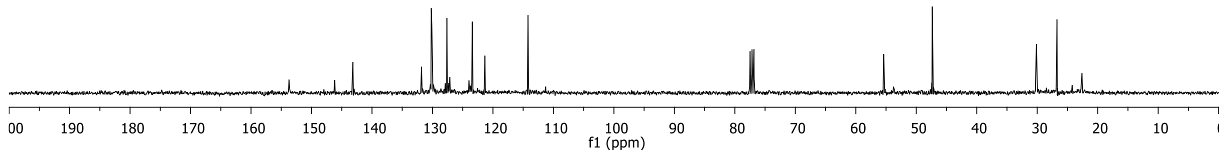

^{1}H NMR (400 MHz) and $^{13}C\{^{1}H\}$ NMR (100 MHz) spectra of **6oj** ($CDCl_3$)

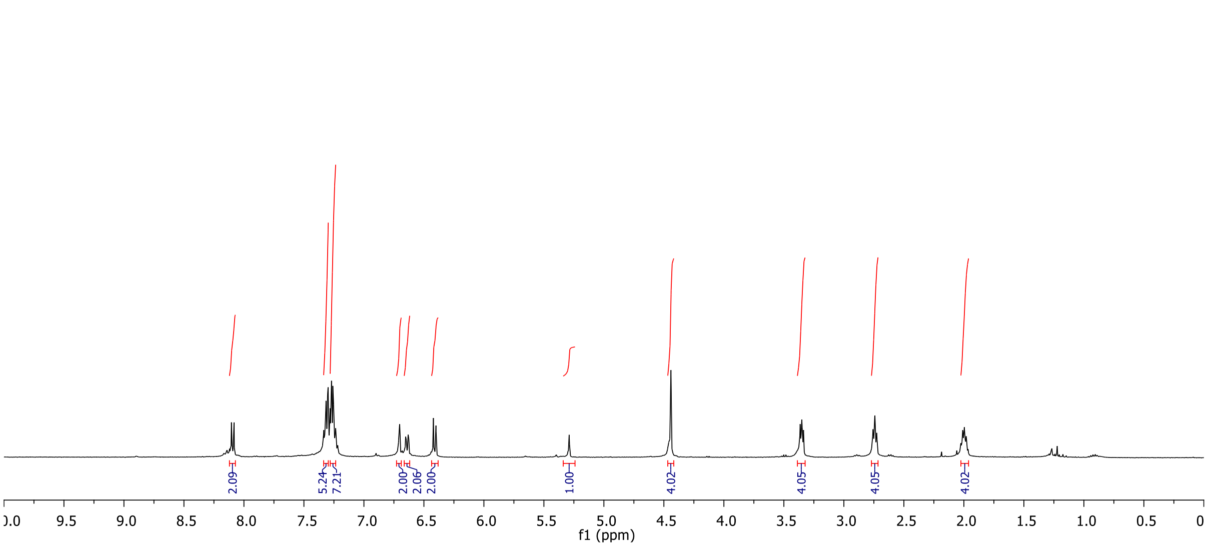

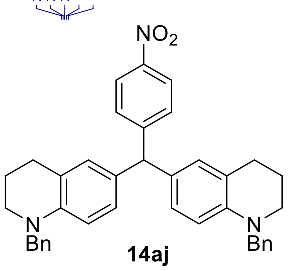

14aj

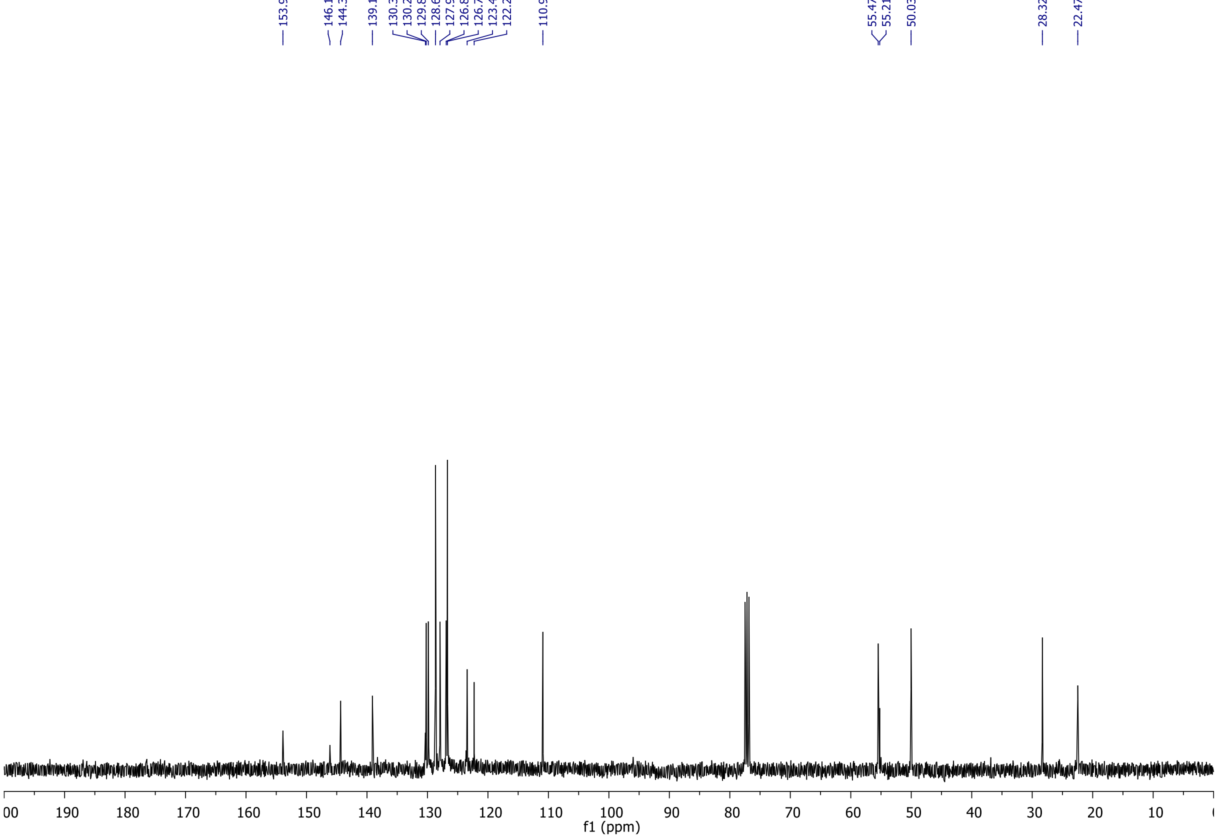

^{1}H NMR (400 MHz) and ^{13}C{^{1}H} NMR (100 MHz) spectra of **14aj** ($CDCl_3$)

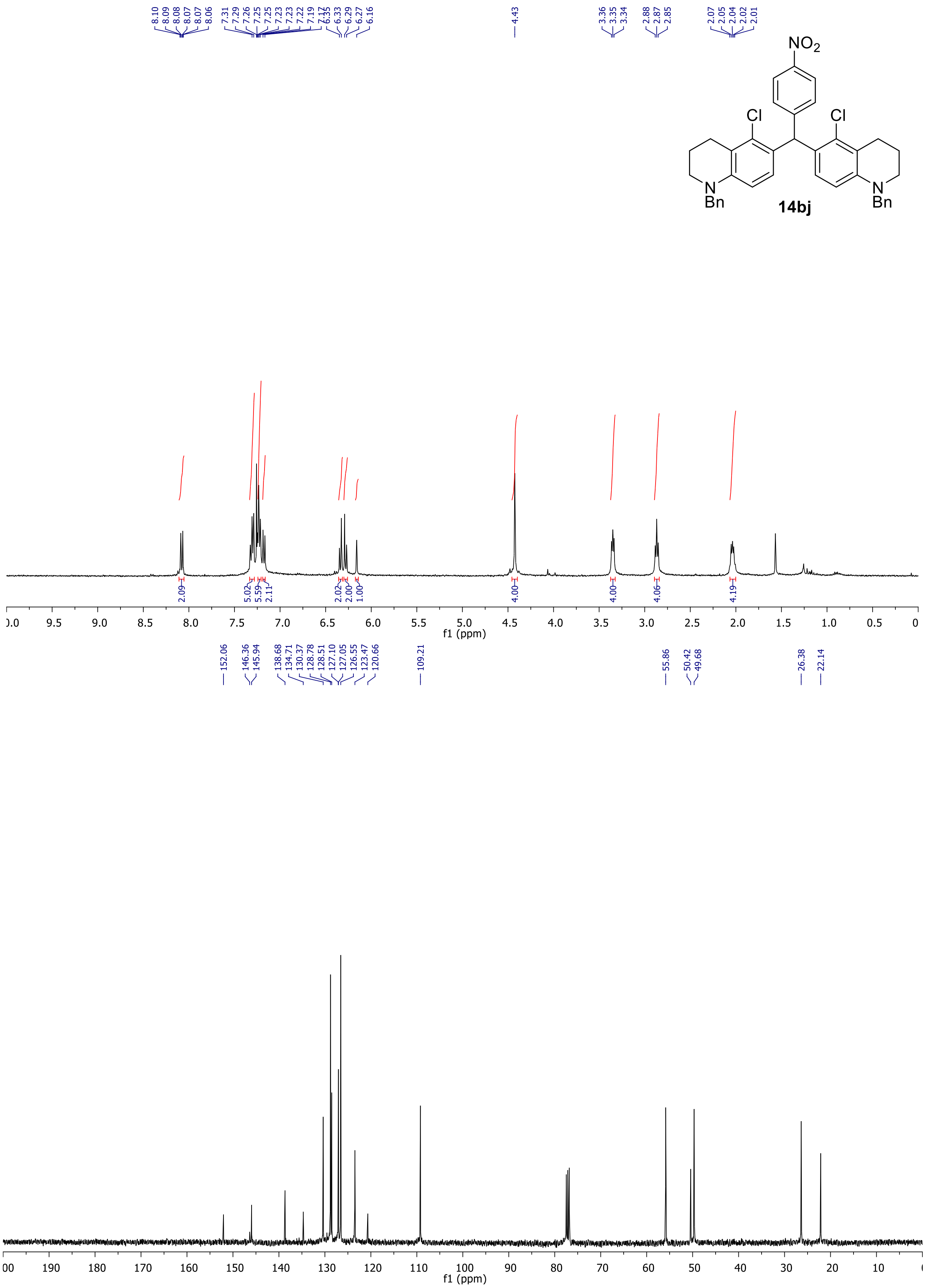

^{1}H NMR (400 MHz) and ^{13}C{^{1}H} NMR (100 MHz) spectra of **14bj** ($CDCl_3$)

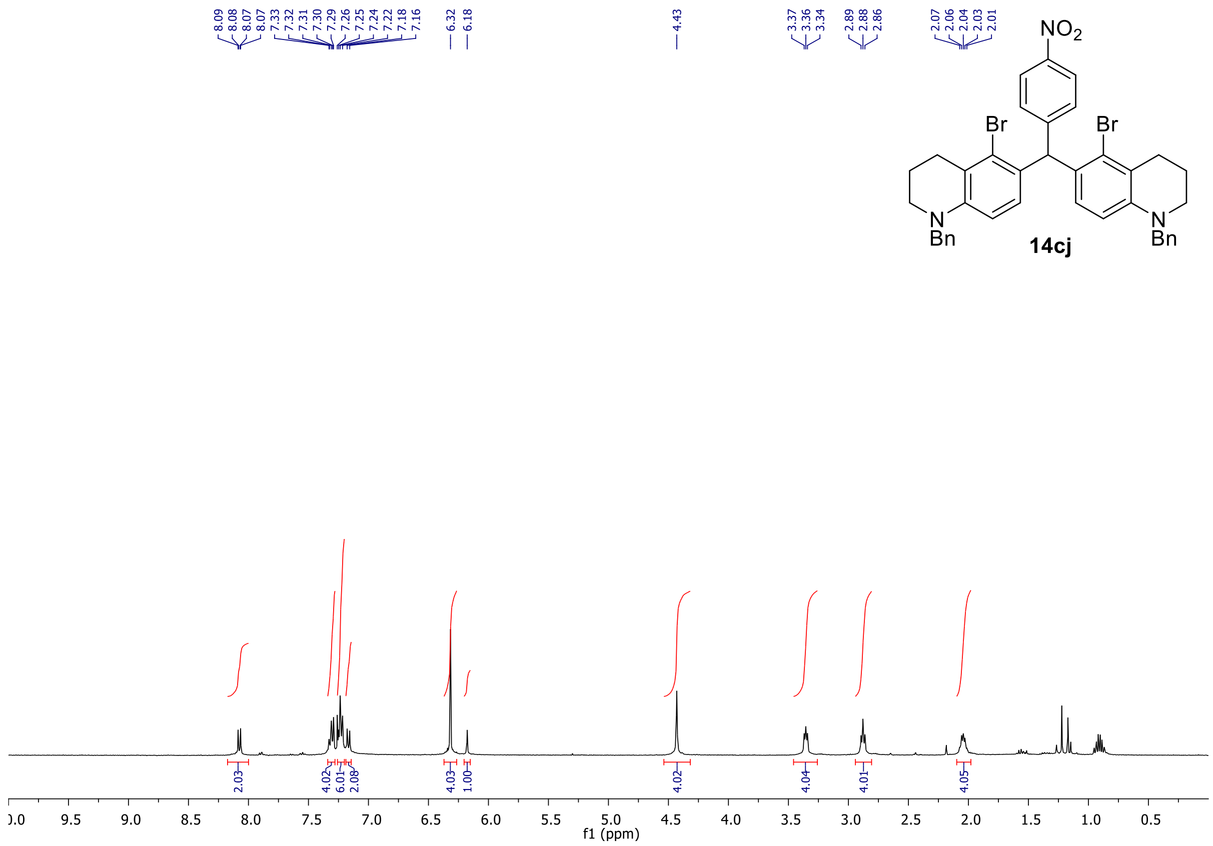

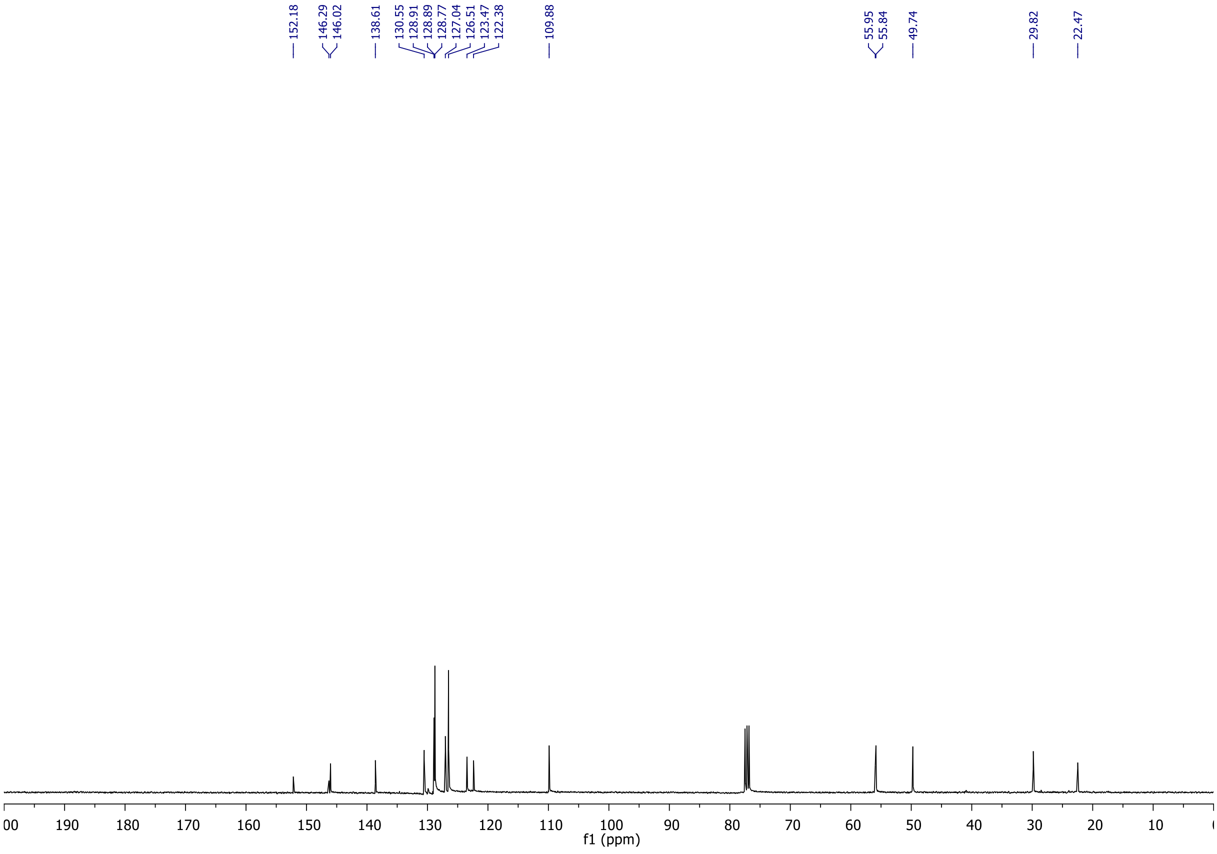

^{1}H NMR (400 MHz) and ^{13}C{^{1}H} NMR (100 MHz) spectra of **14cj** ($CDCl_3$)

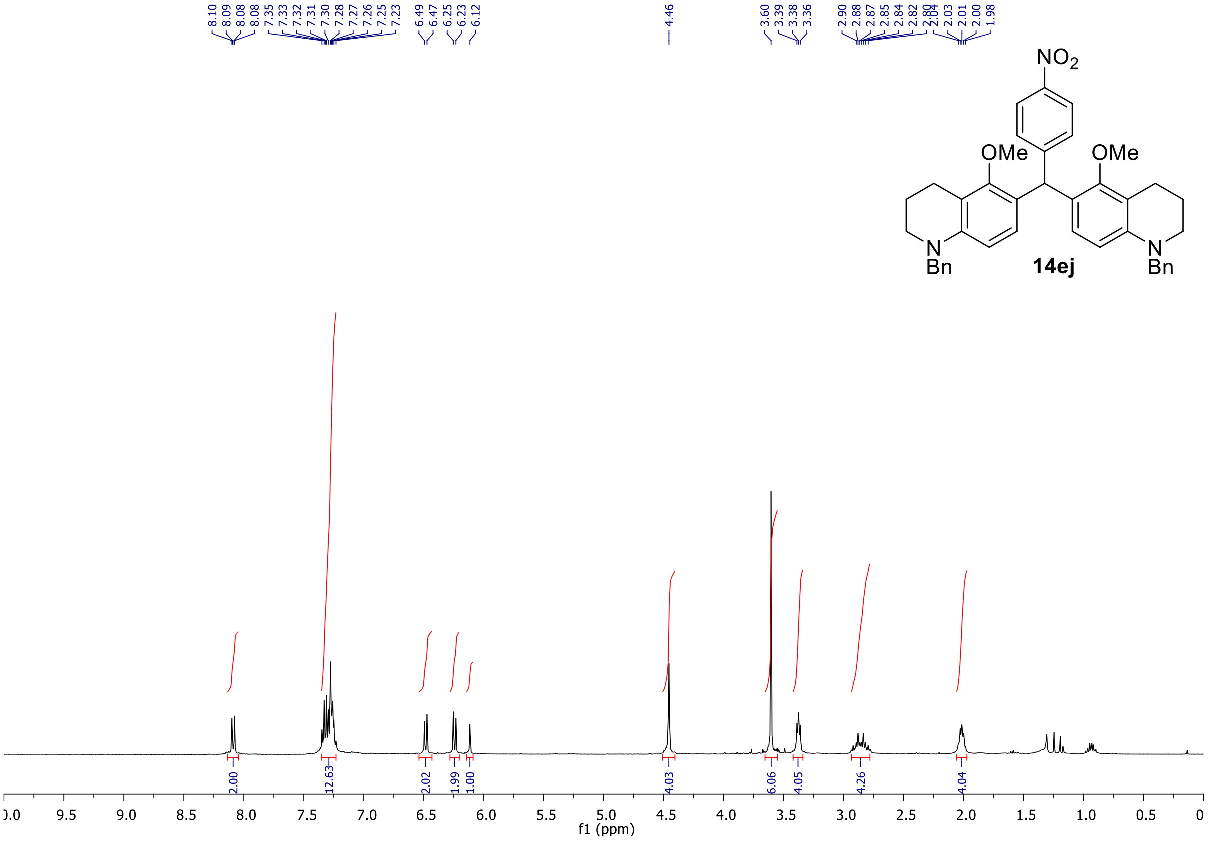

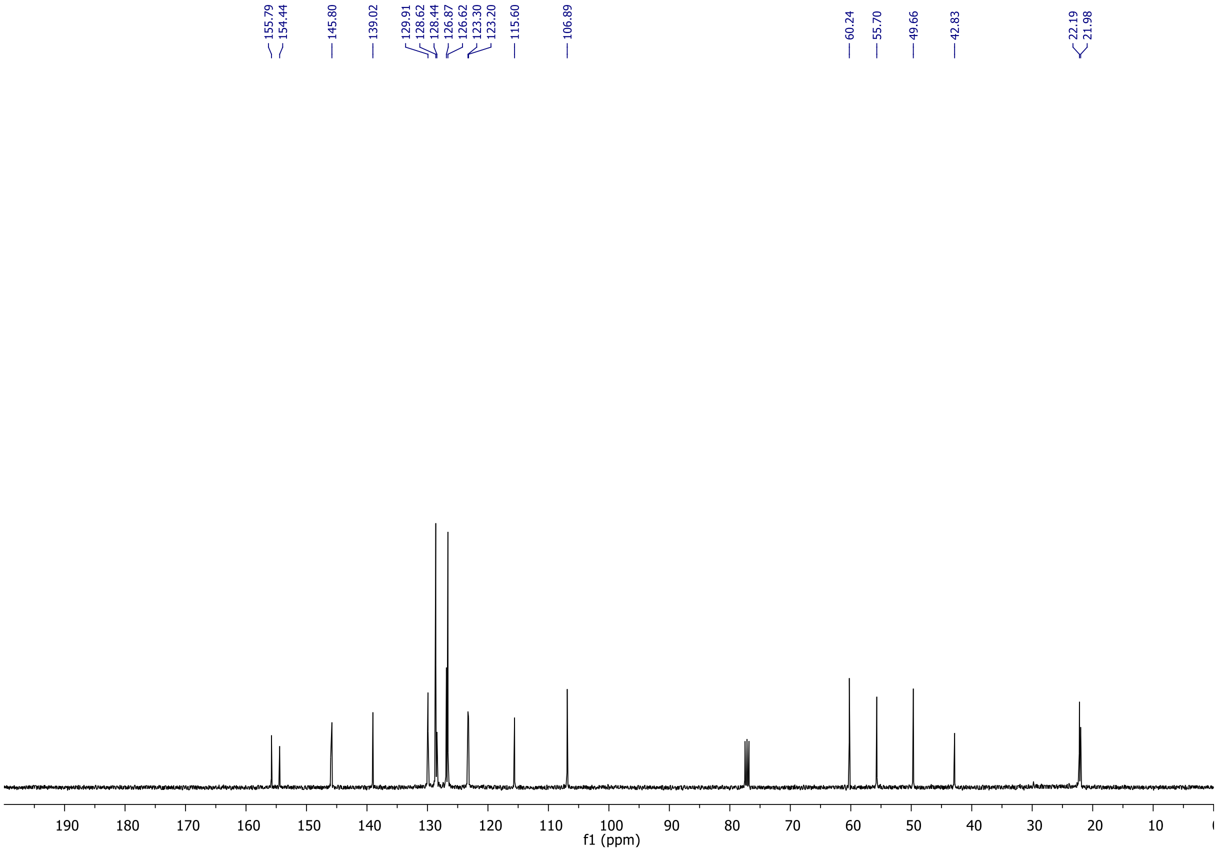

^{1}H NMR (400 MHz) and $^{13}C\{^{1}H\}$ NMR (100 MHz) spectra of **14ej** ($CDCl_3$)

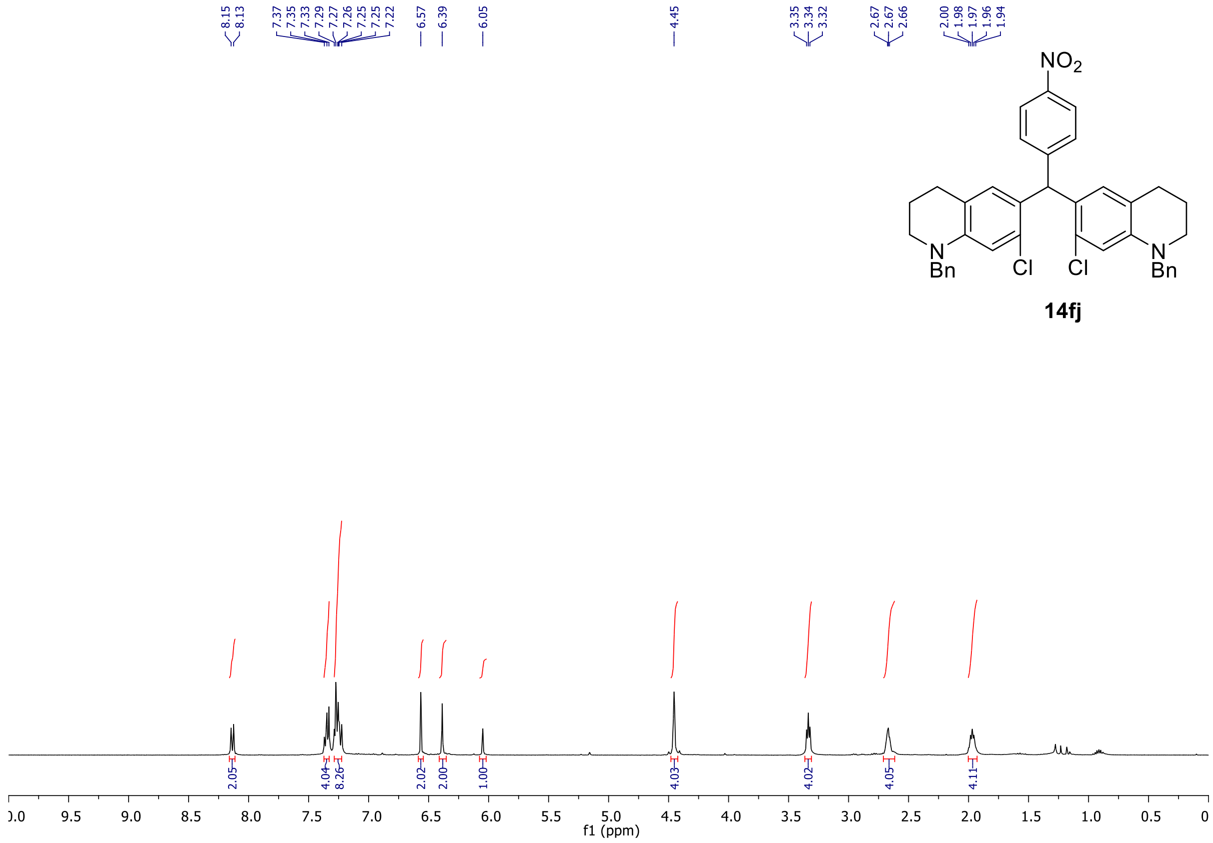

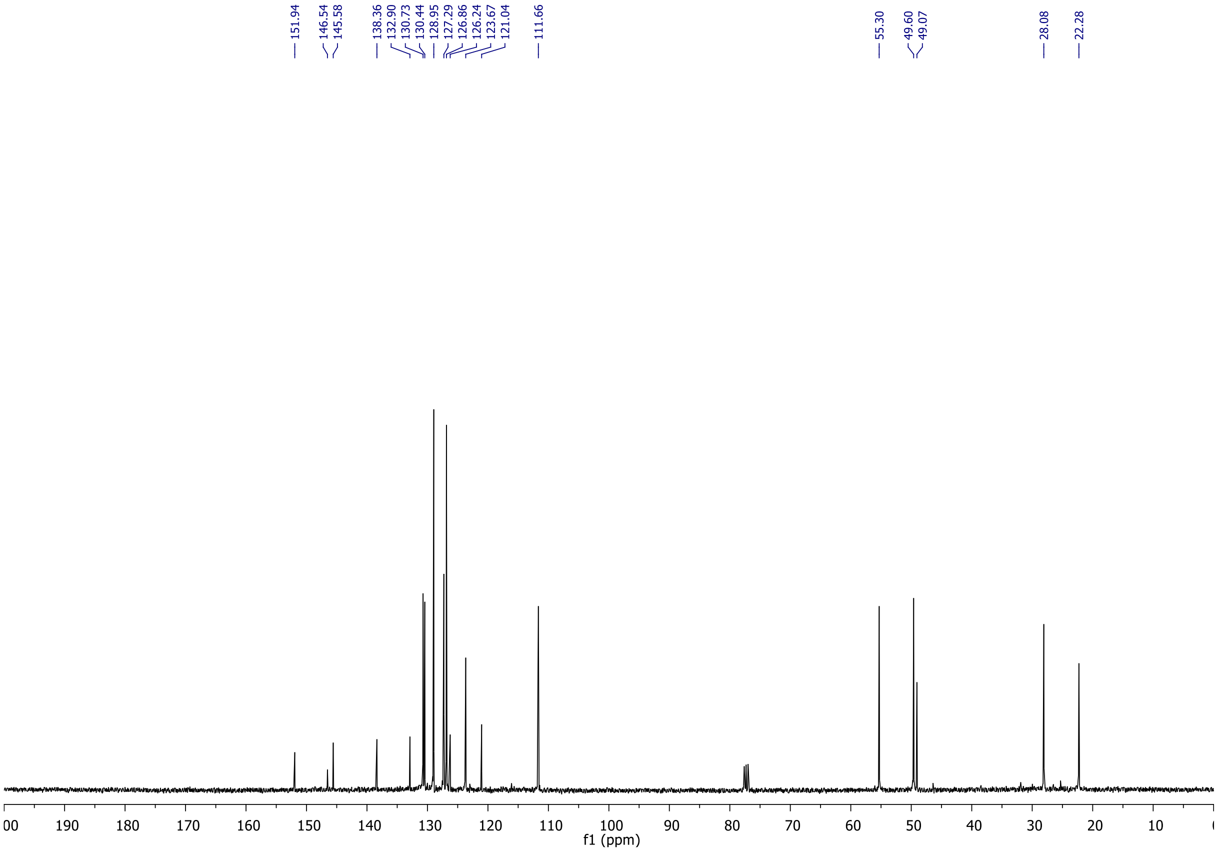

^{1}H NMR (400 MHz) and ^{13}C{^{1}H} NMR (100 MHz) spectra of **14fj** ($CDCl_3$)

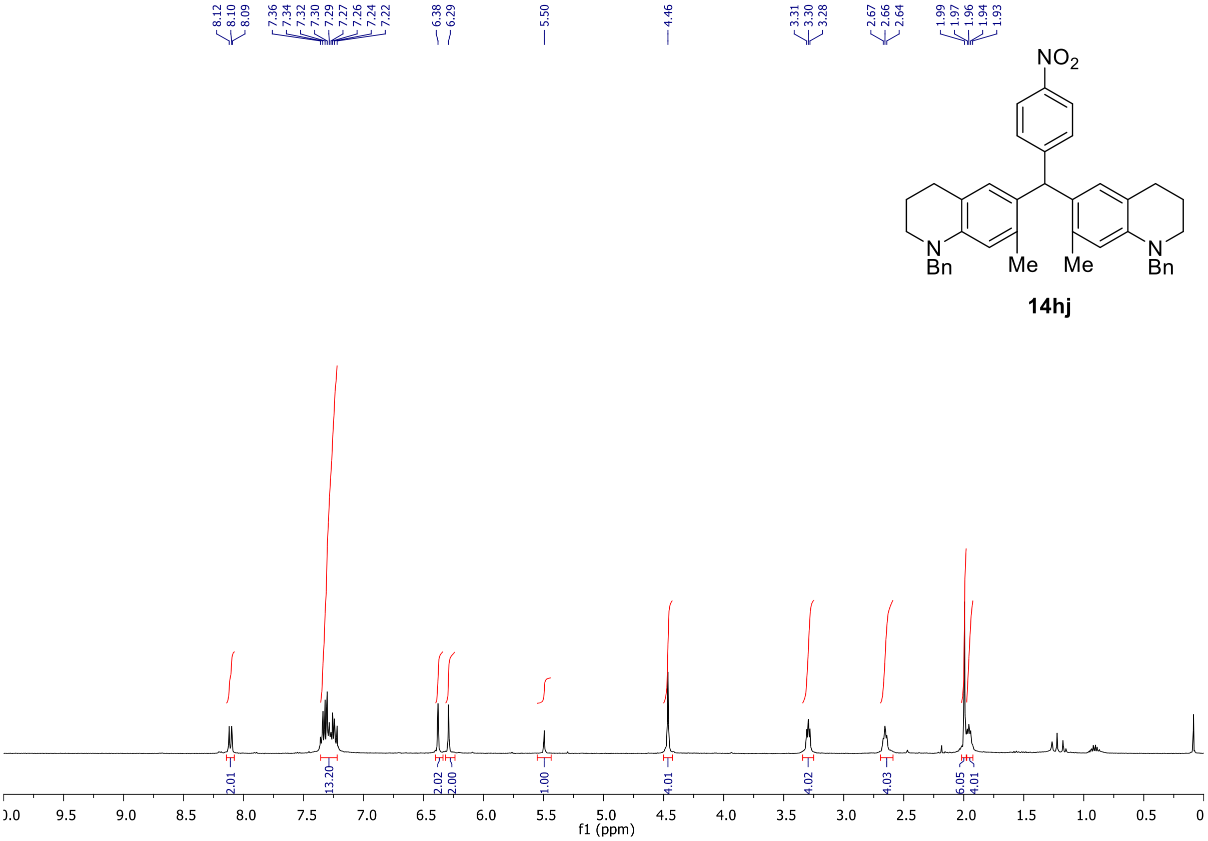

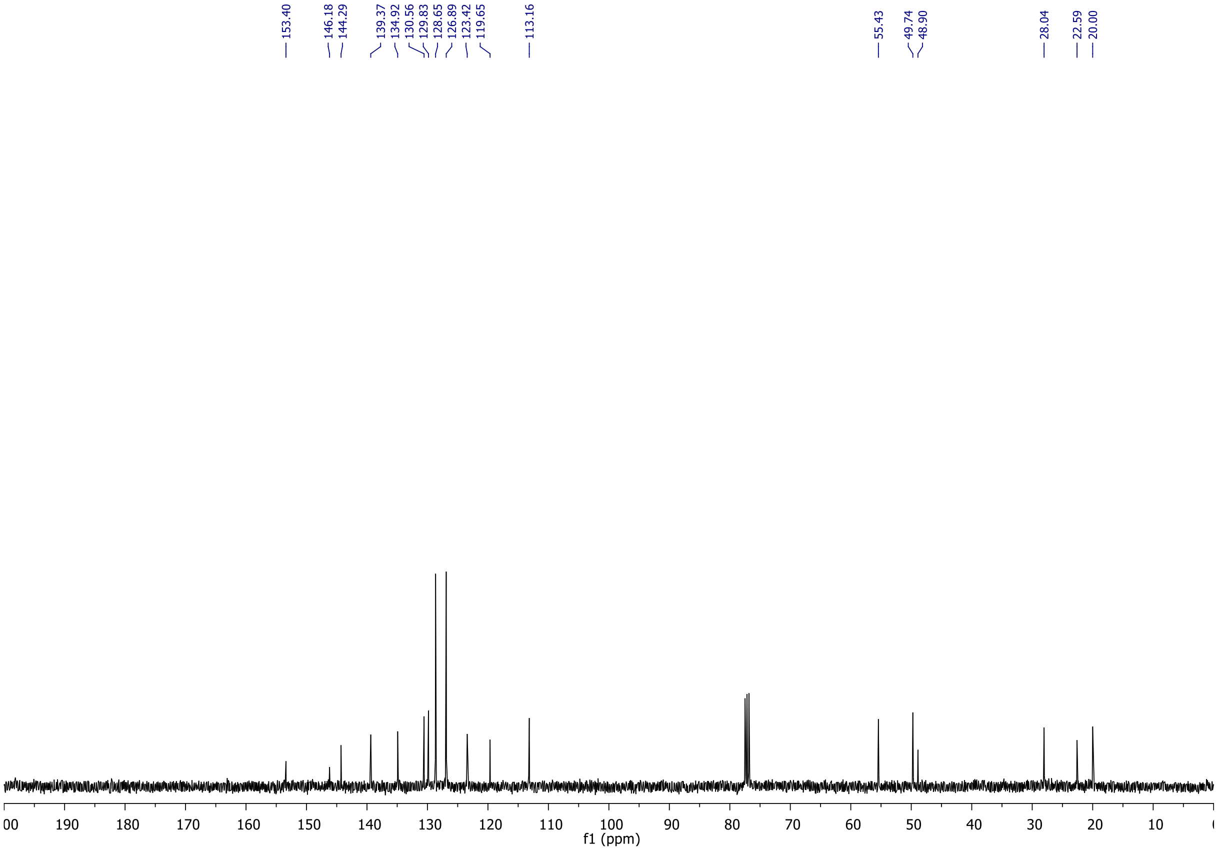

^{1}H NMR (400 MHz) and ^{13}C{^{1}H} NMR (100 MHz) spectra of **14hj** (CDCl$_3$)

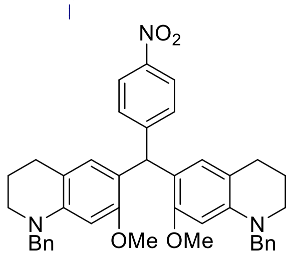

14ij

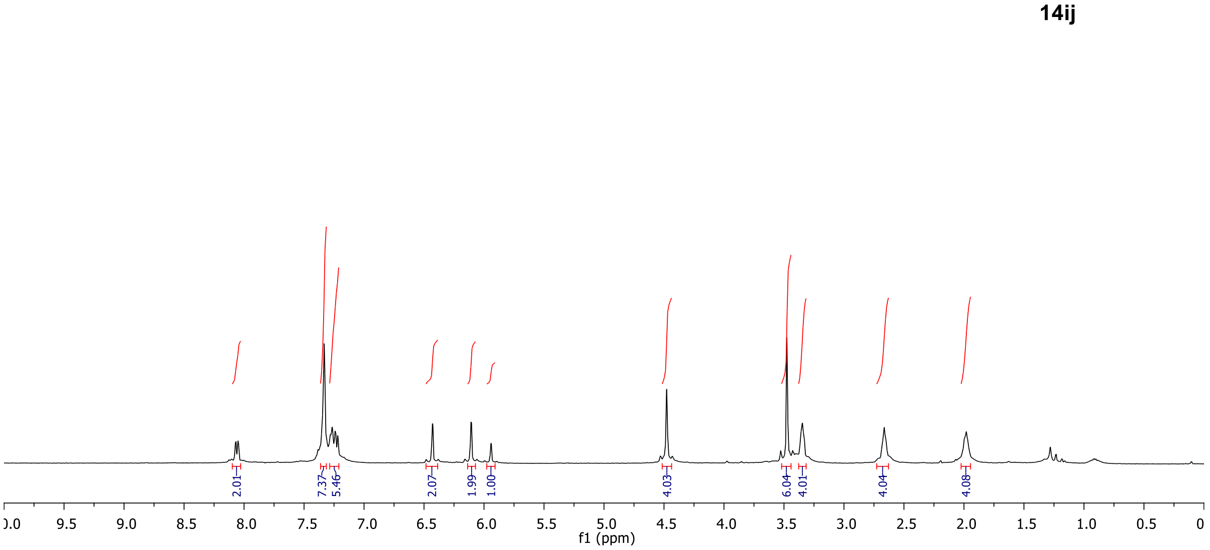

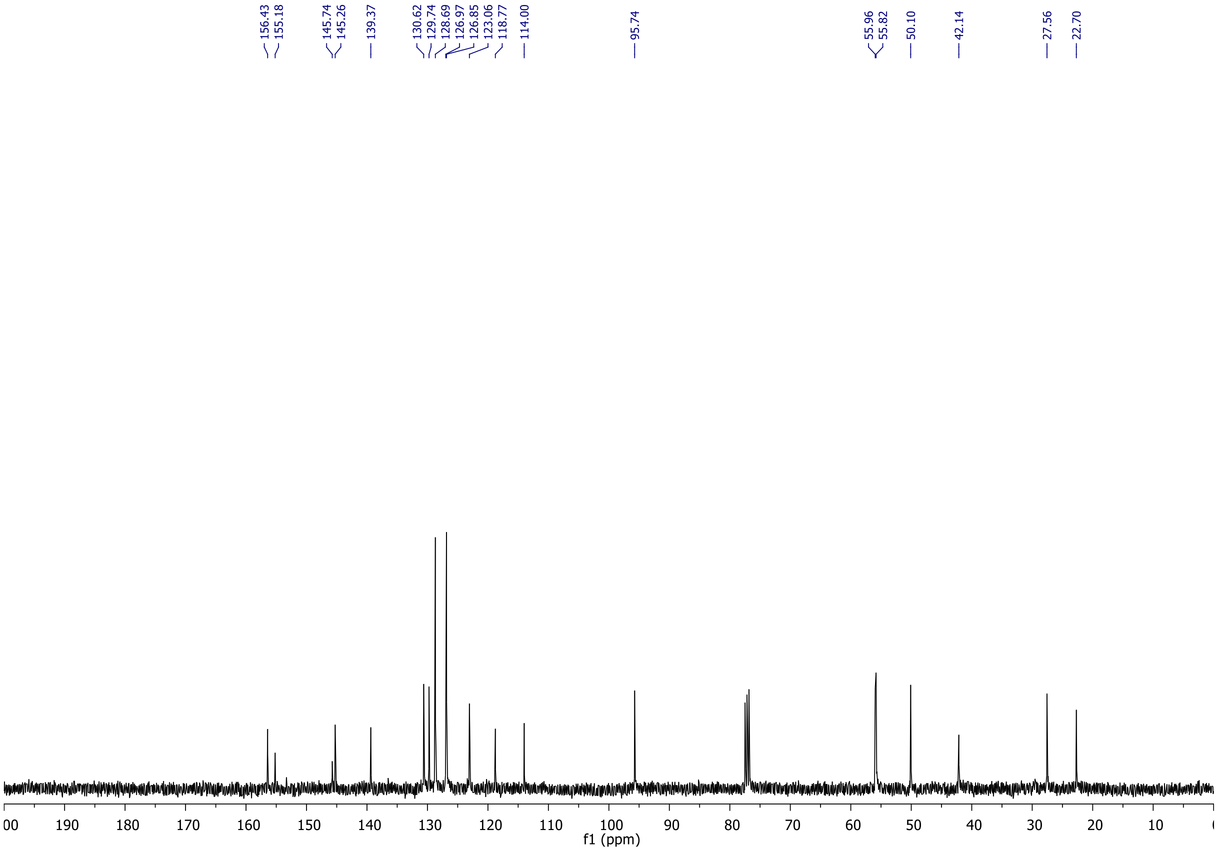

^{1}H NMR (400 MHz) and $^{13}C\{^{1}H\}$ NMR (100 MHz) spectra of **14ij** ($CDCl_3$)

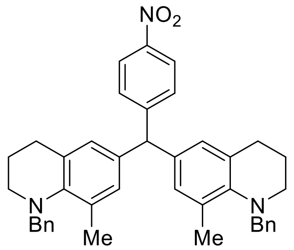

14mj

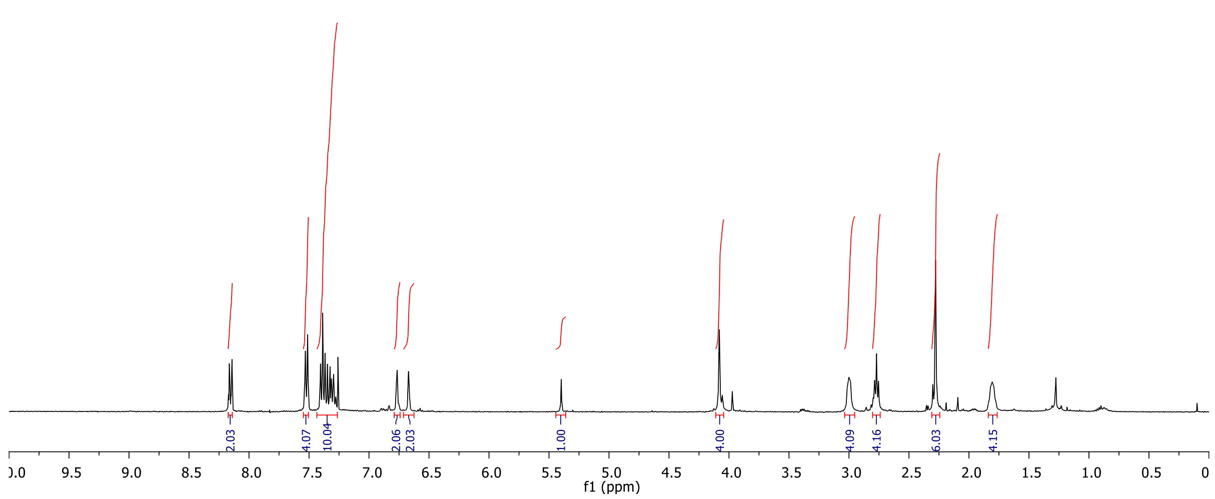

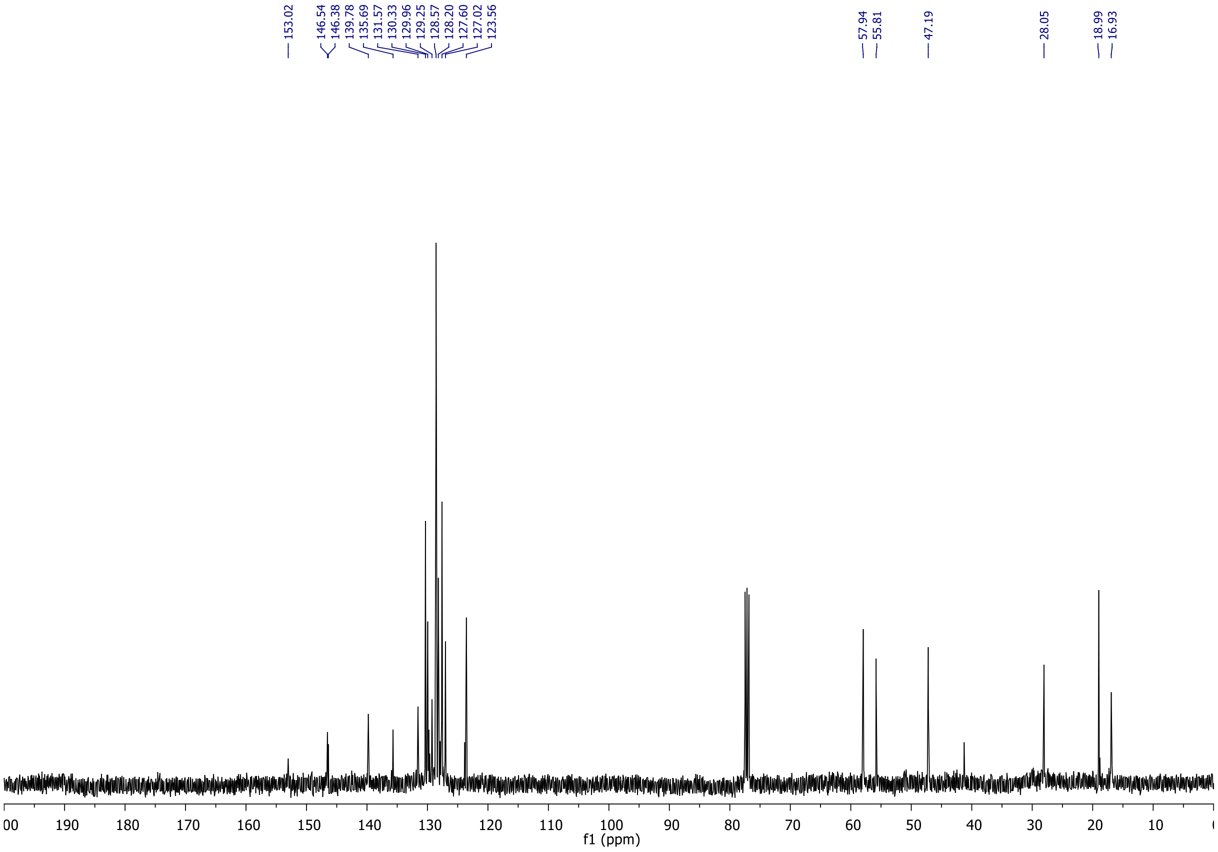

^{1}H NMR (400 MHz) and ^{13}C{^{1}H} NMR (100 MHz) spectra of **14mj** ($CDCl_3$)

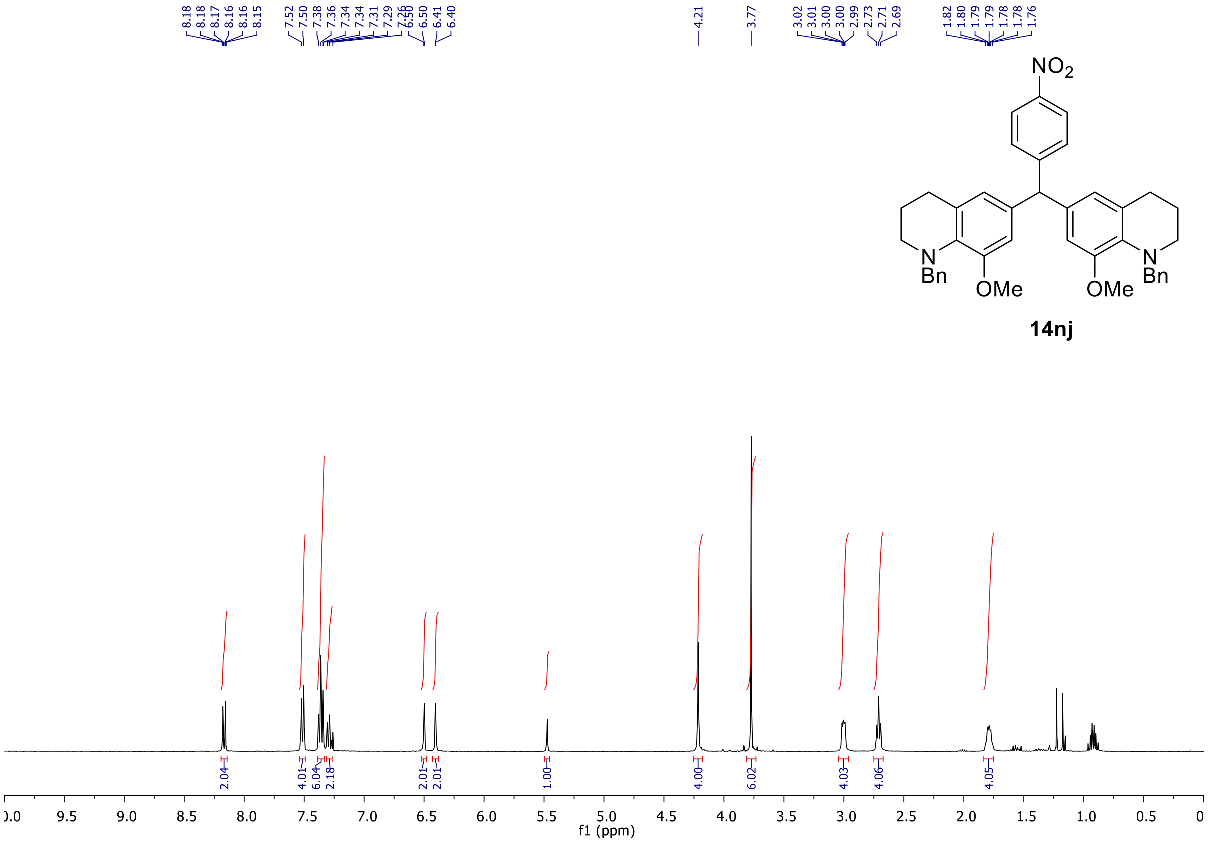

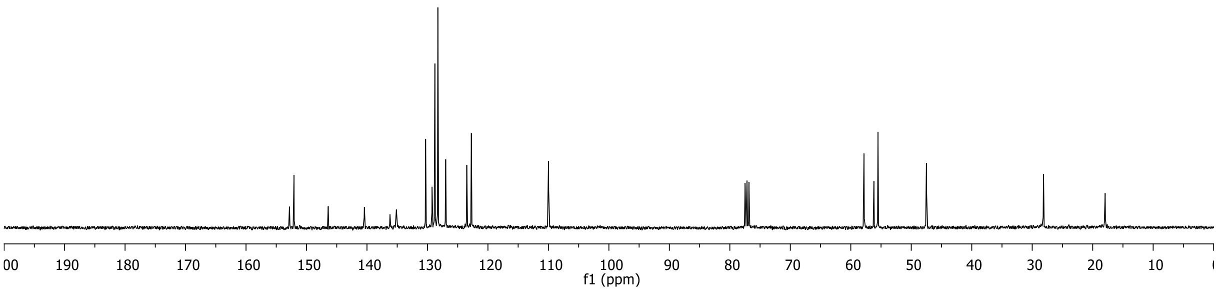

^{1}H NMR (400 MHz) and ^{13}C{^{1}H} NMR (100 MHz) spectra of **14nj** ($CDCl_3$)

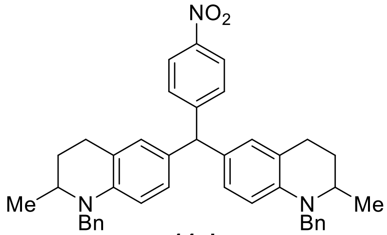

14oj

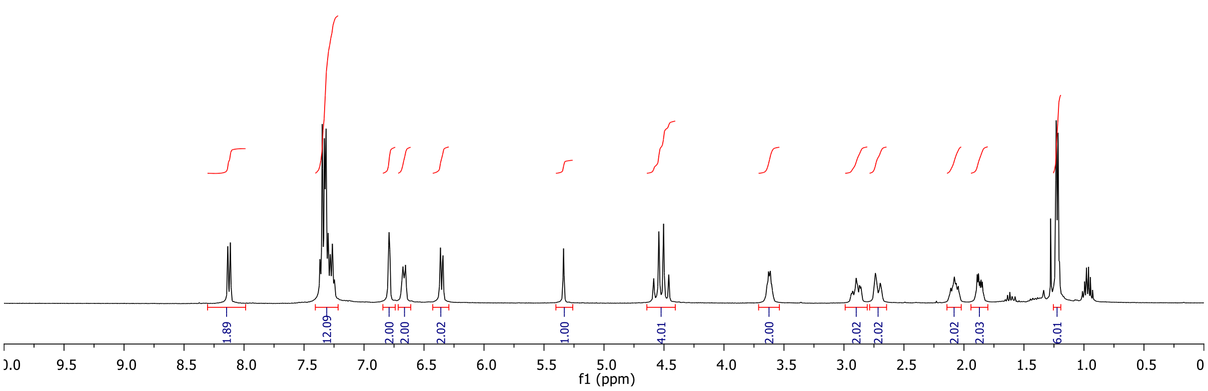

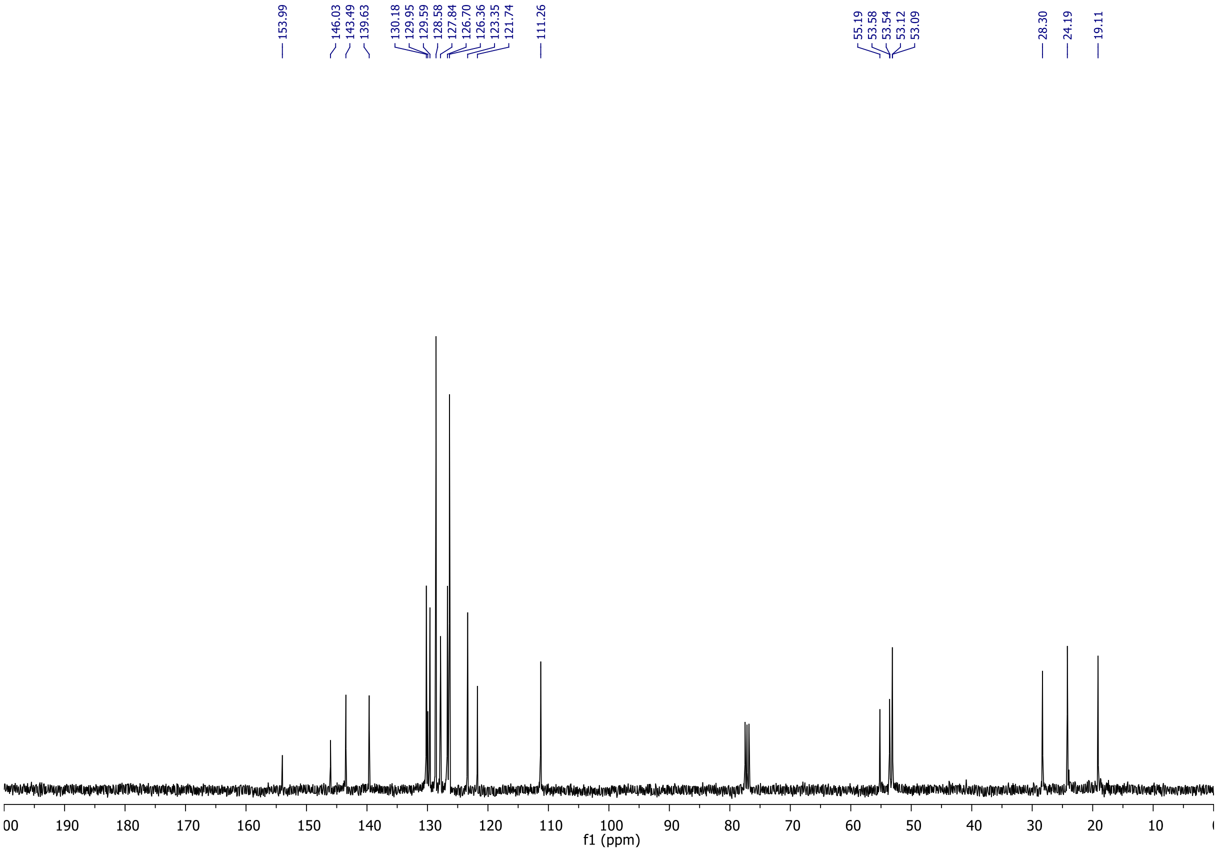

^{1}H NMR (400 MHz) and $^{13}C\{^{1}H\}$ NMR (100 MHz) spectra of **14oj** ($CDCl_3$)

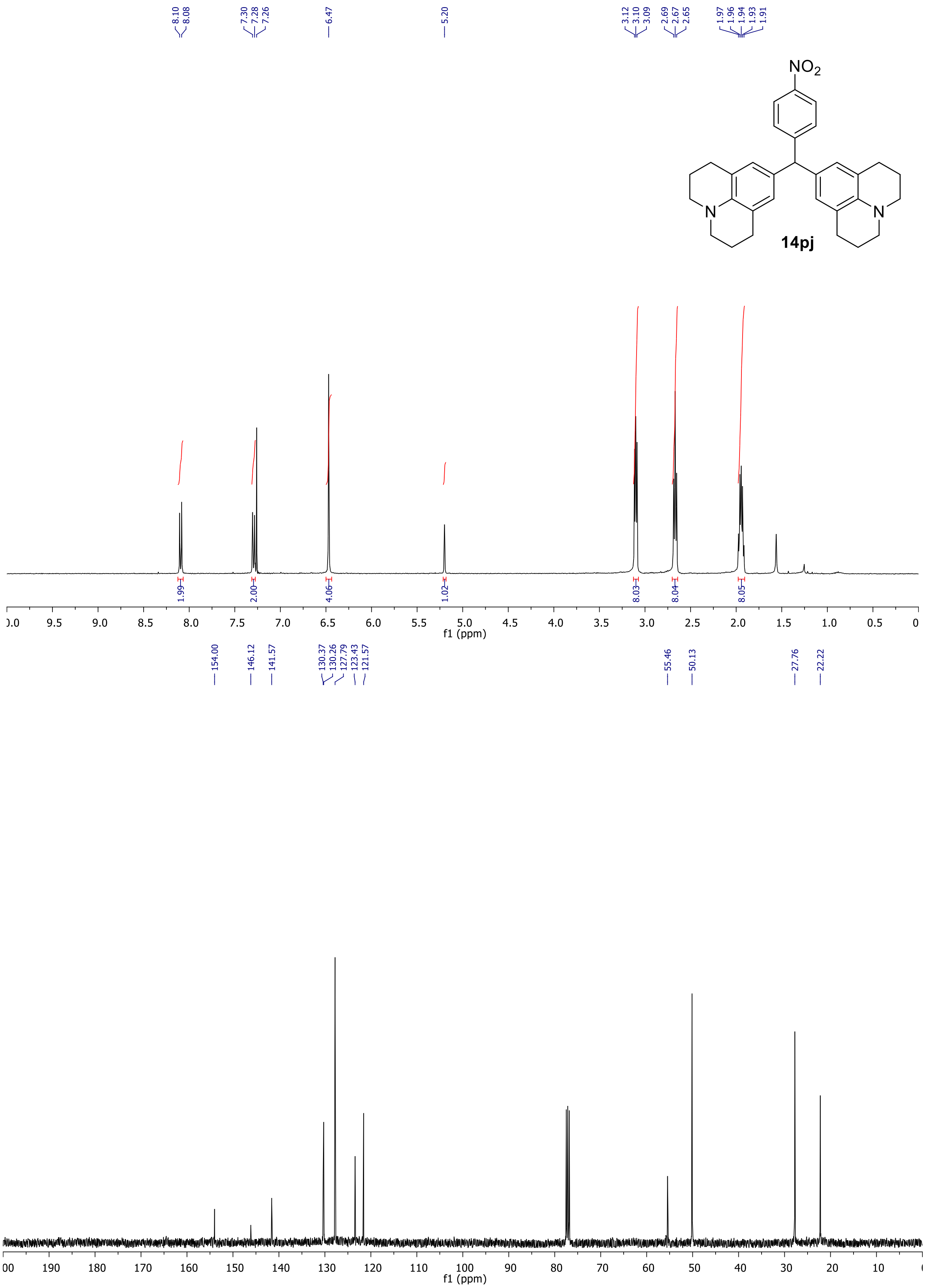

^{1}H NMR (400 MHz) and ^{13}C{^{1}H} NMR (100 MHz) spectra of **14pj** ($CDCl_3$)

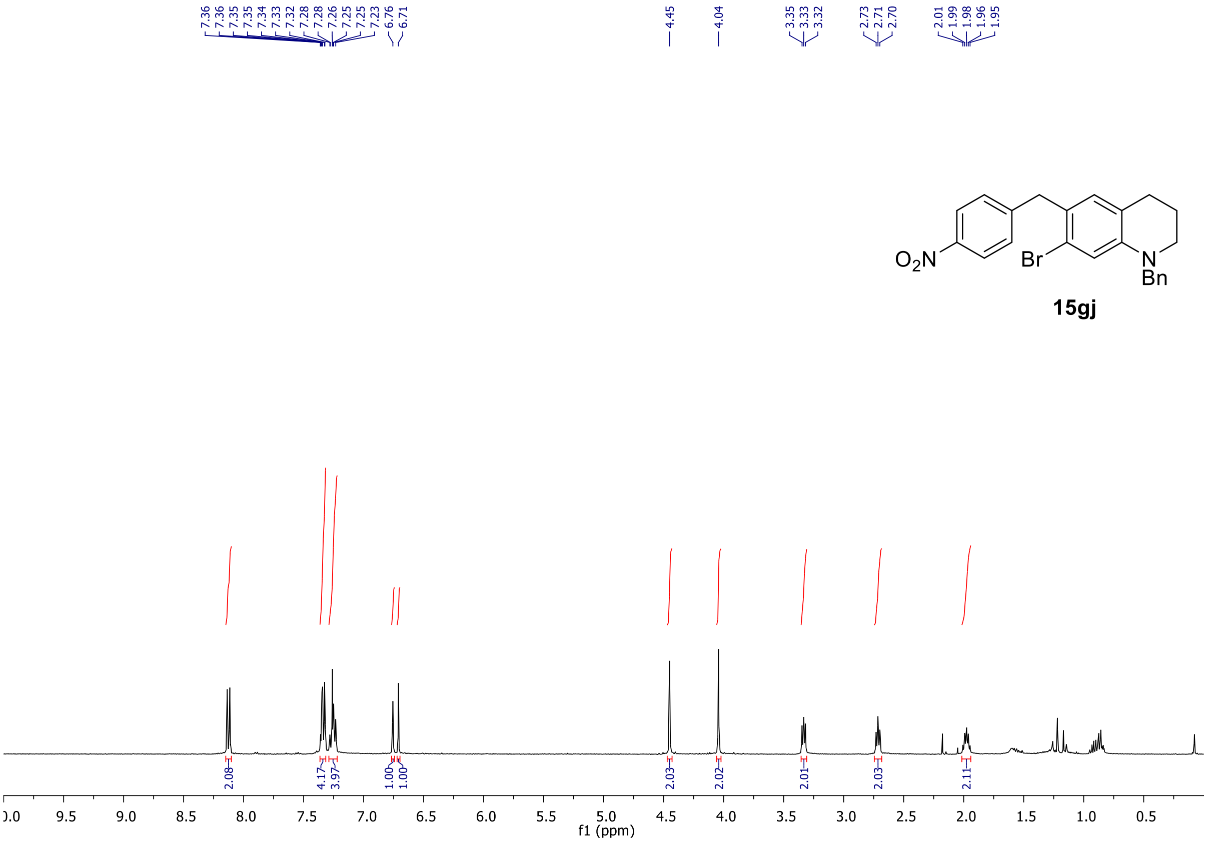

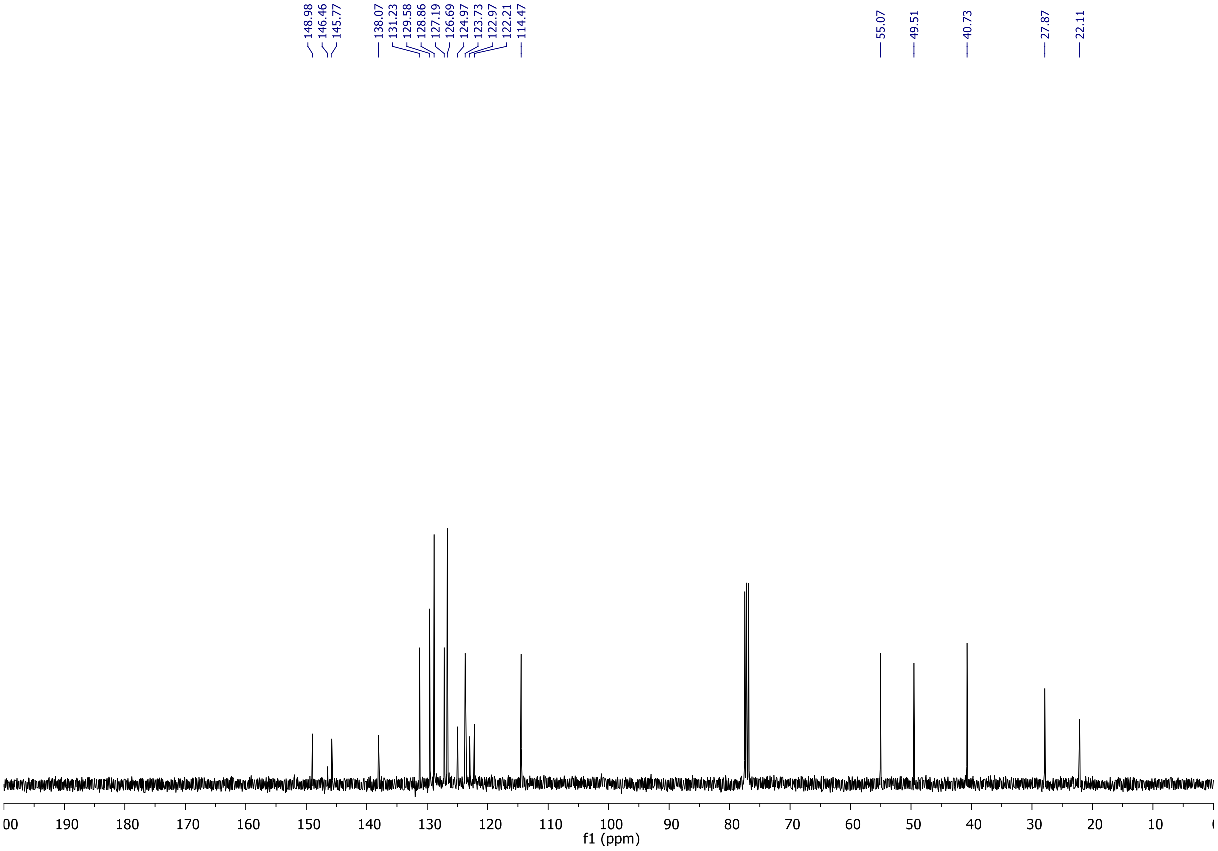

^{1}H NMR (400 MHz) and ^{13}C{^{1}H} NMR (100 MHz) spectra of **15gj** ($CDCl_3$)

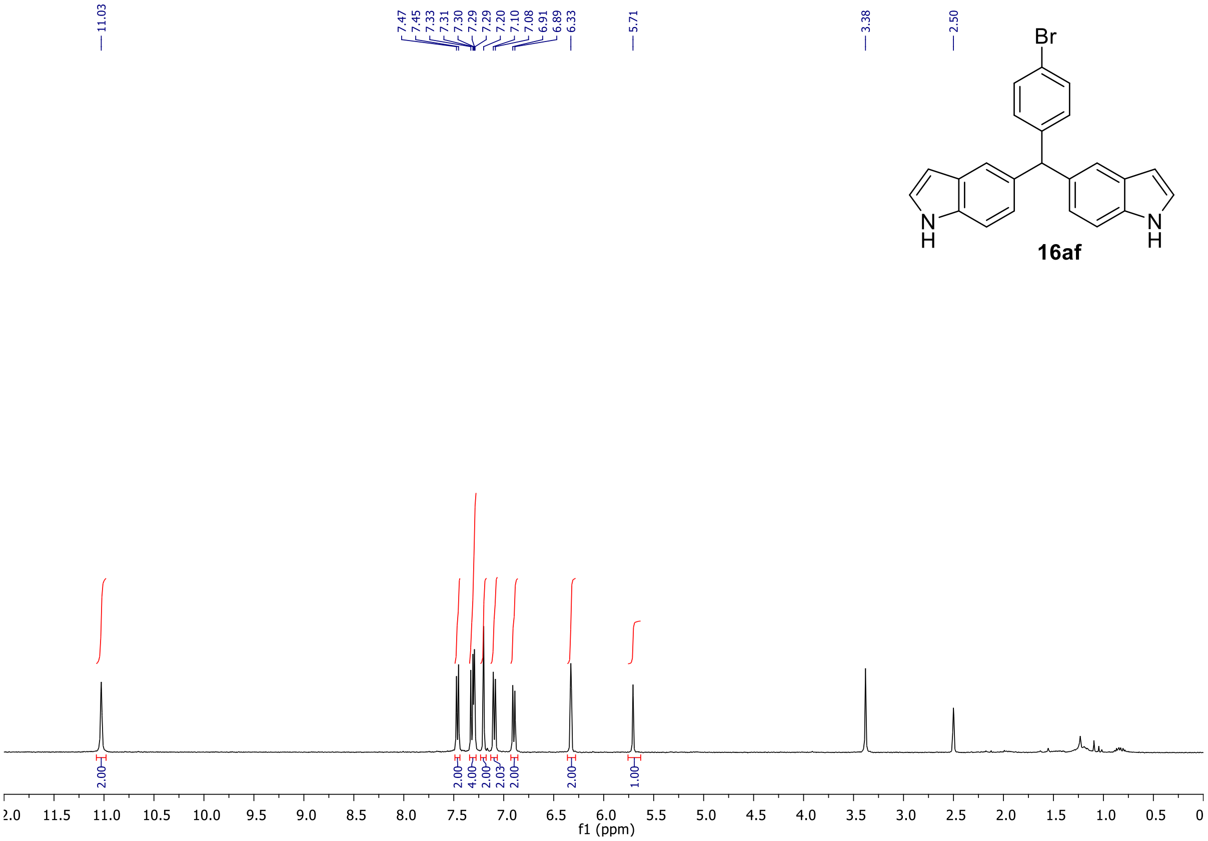

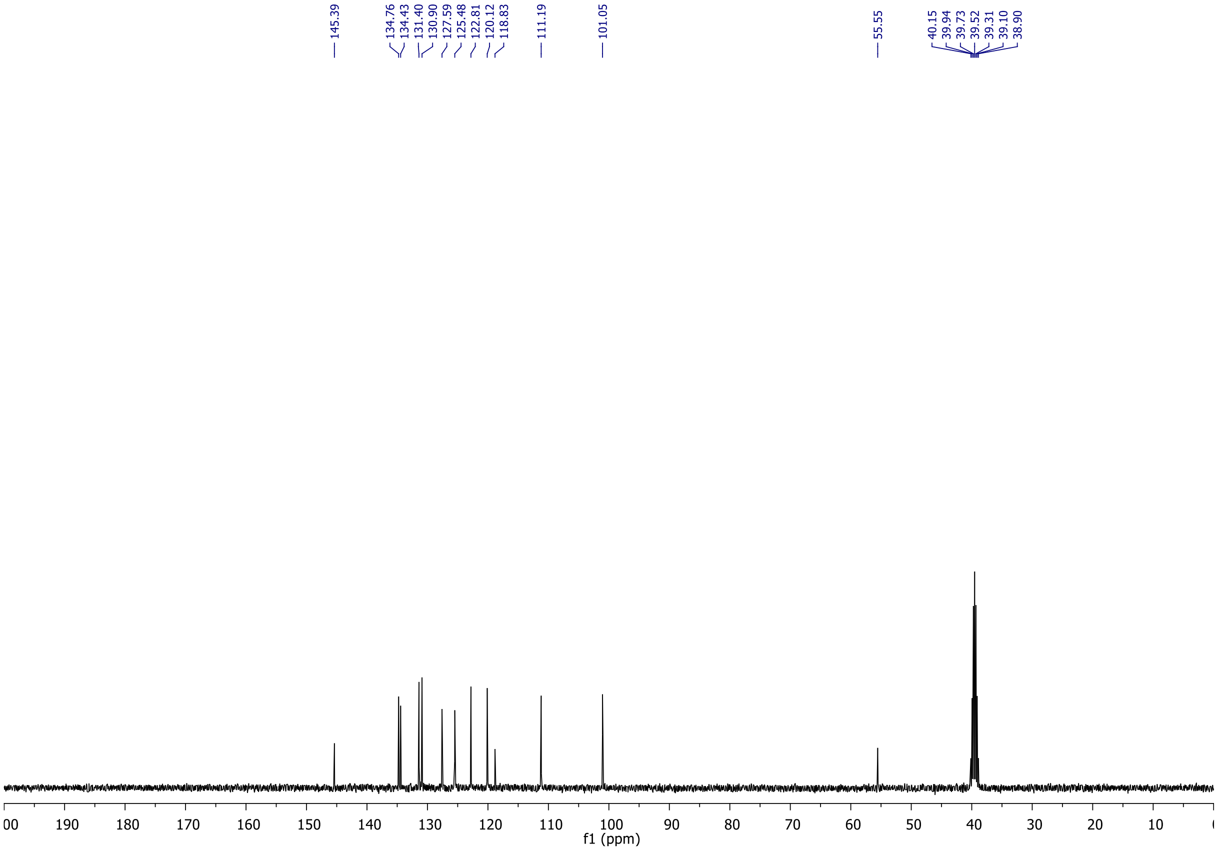

^{1}H NMR (400 MHz) and ^{13}C{^{1}H} NMR (100 MHz) spectra of **16af** (DMSO-d_6)

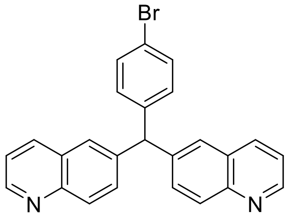

17af

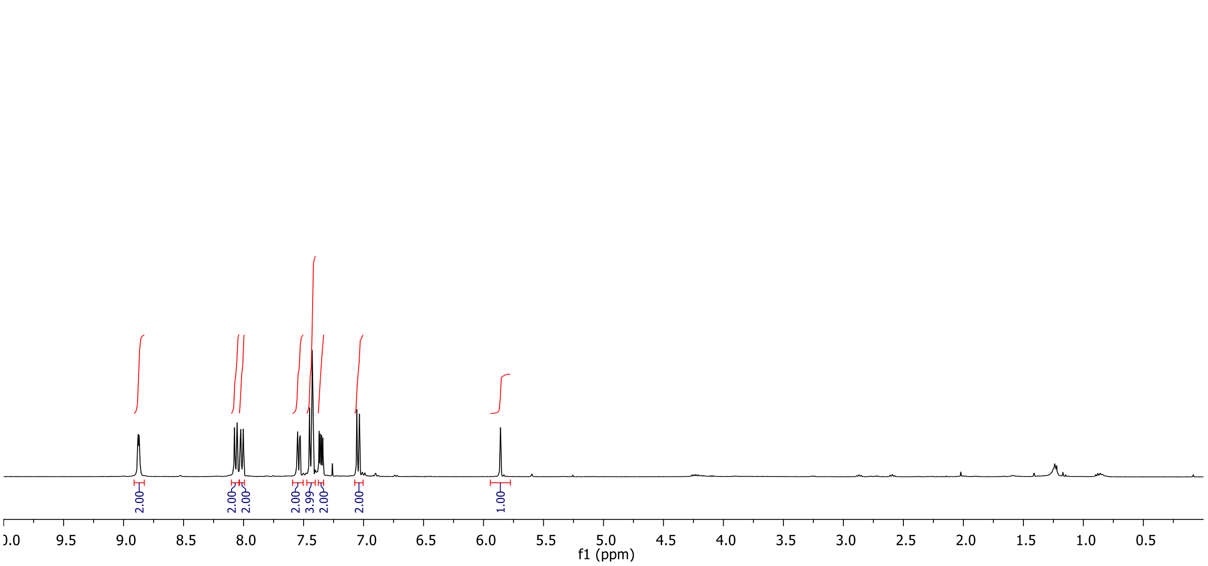

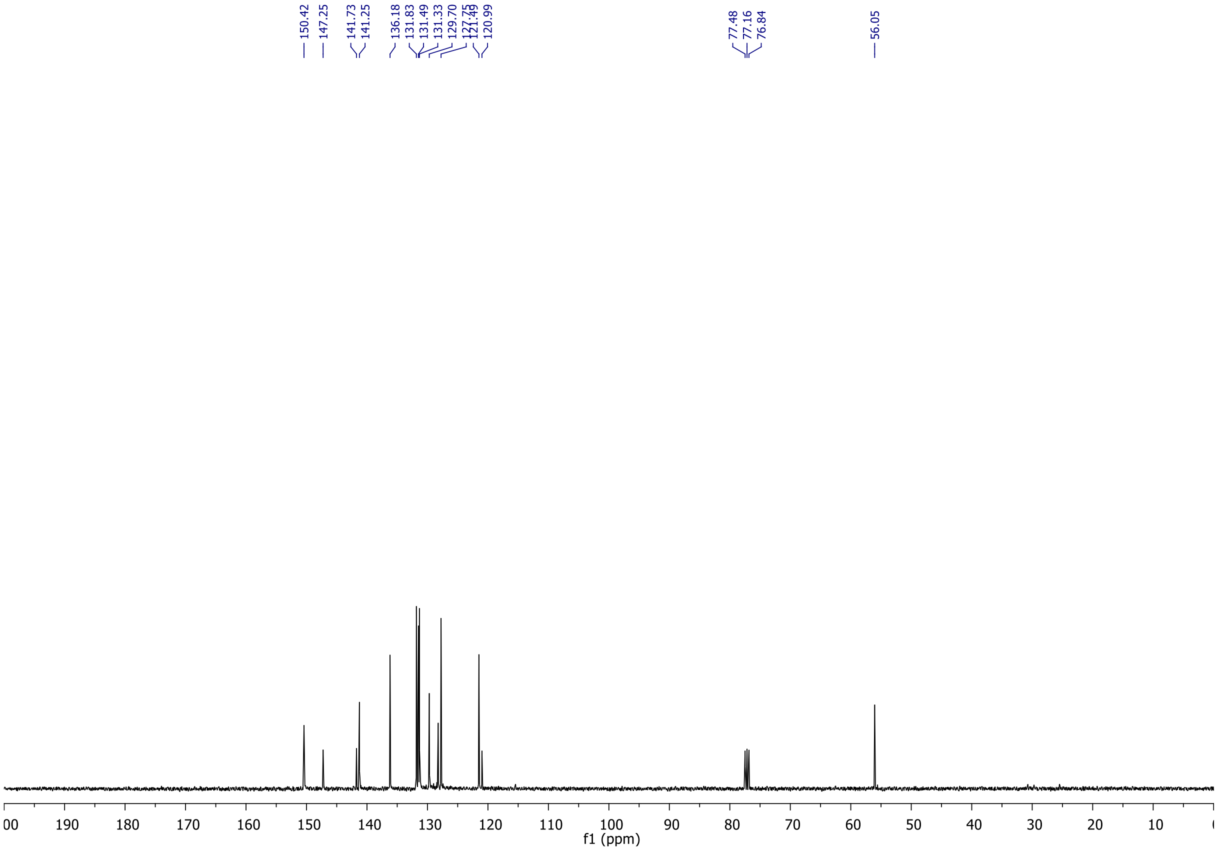

^{1}H NMR (400 MHz) and $^{13}C\{^{1}H\}$ NMR (100 MHz) spectra of **17af** ($CDCl_3$)

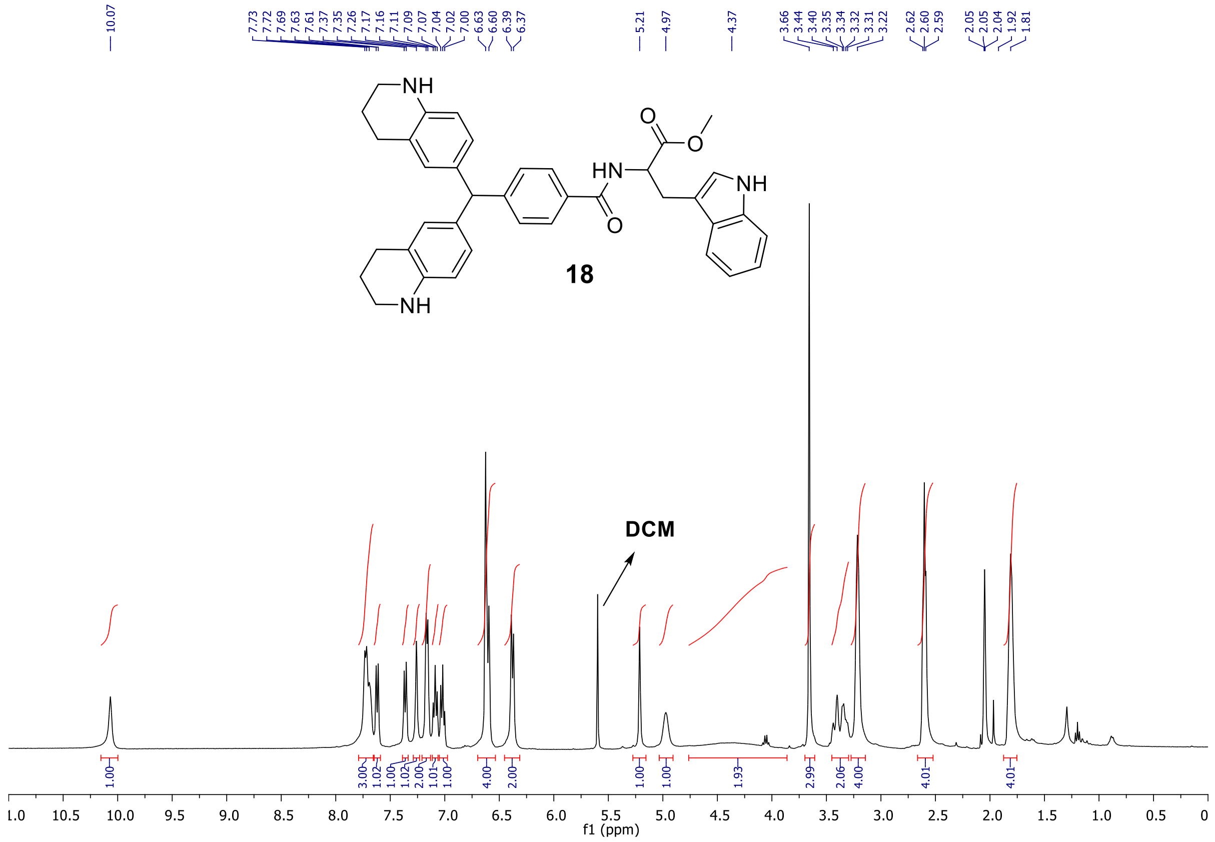

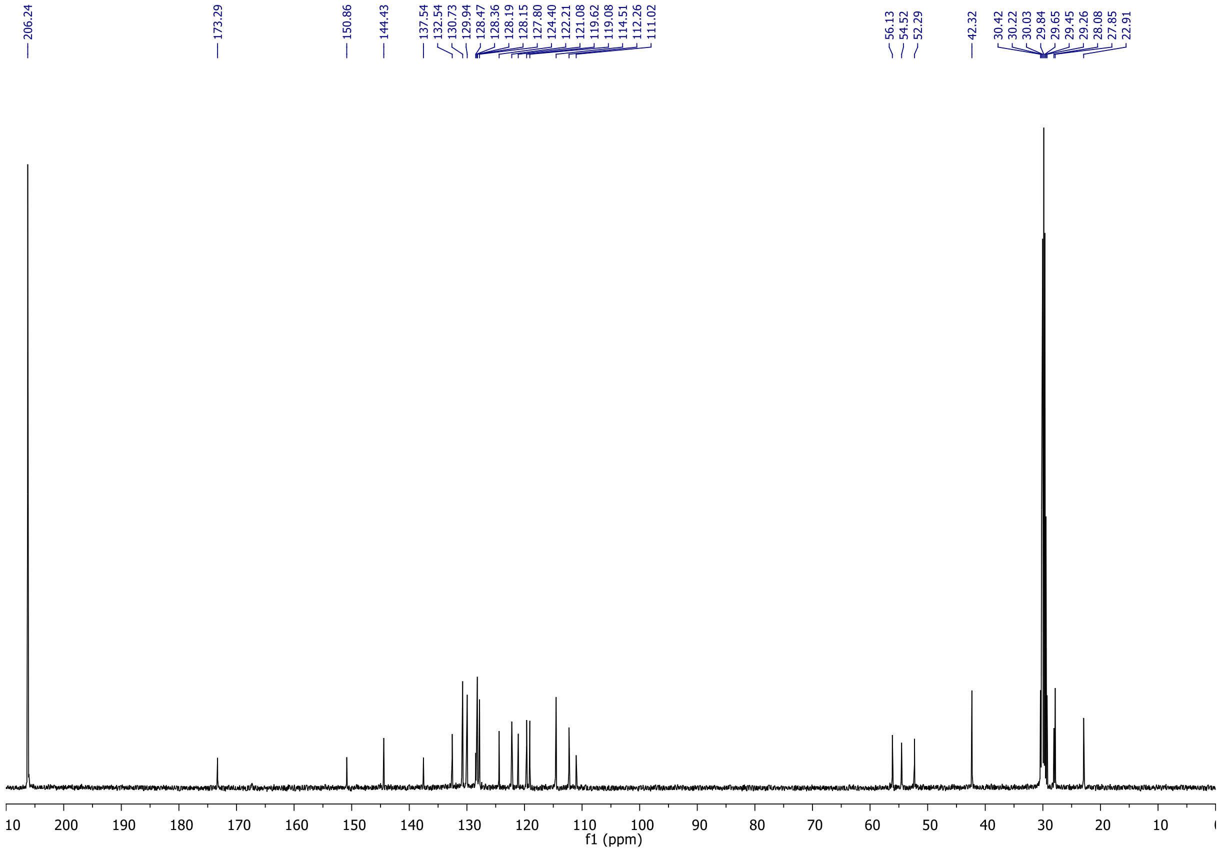

^{1}H NMR (400 MHz) and ^{13}C{^{1}H} NMR (100 MHz) spectra of **18** (Acetone-d_6)

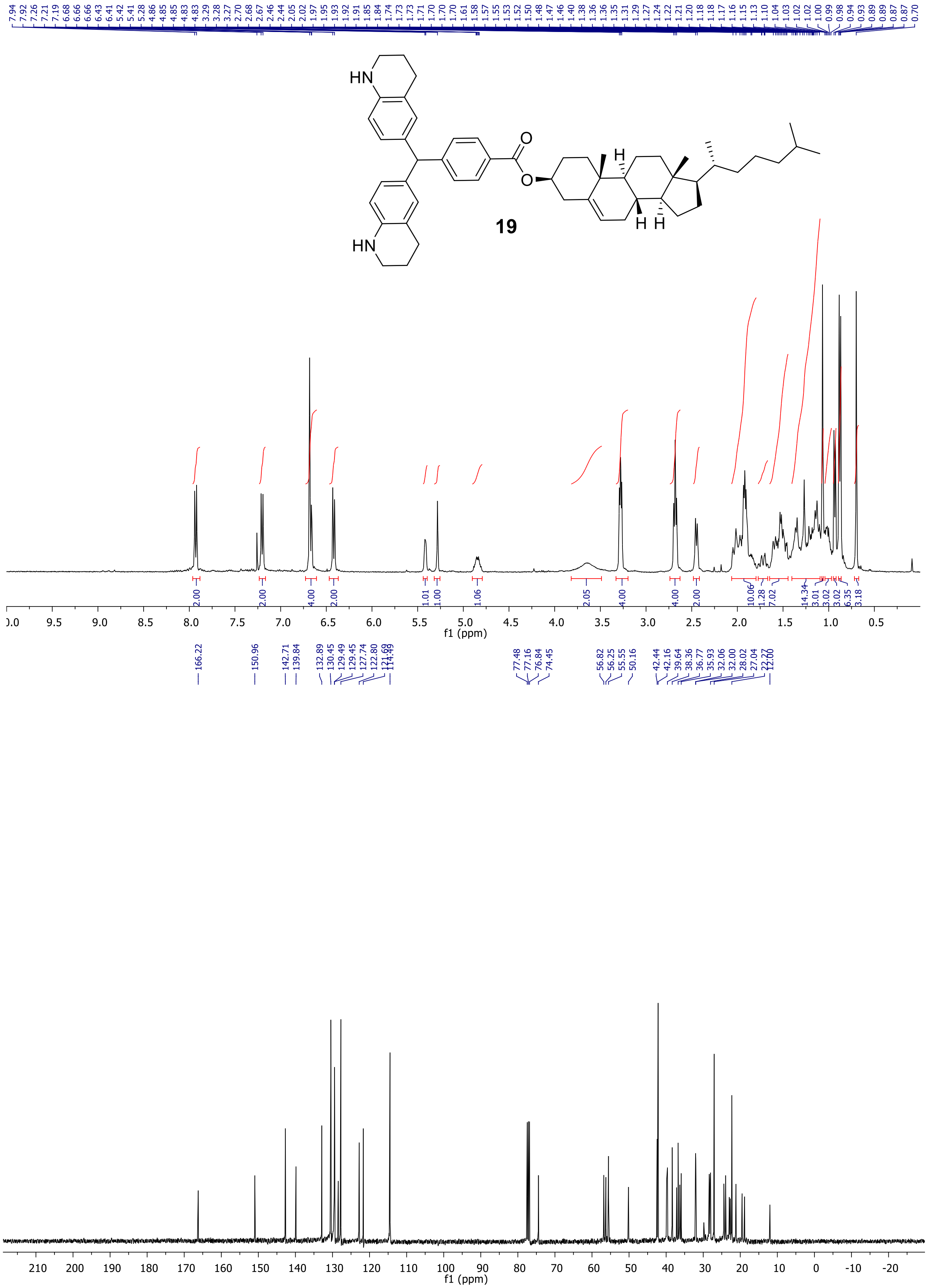

^{1}H NMR (400 MHz) and $^{13}C\{^{1}H\}$ NMR (100 MHz) spectra of **19** ($CDCl_3$)

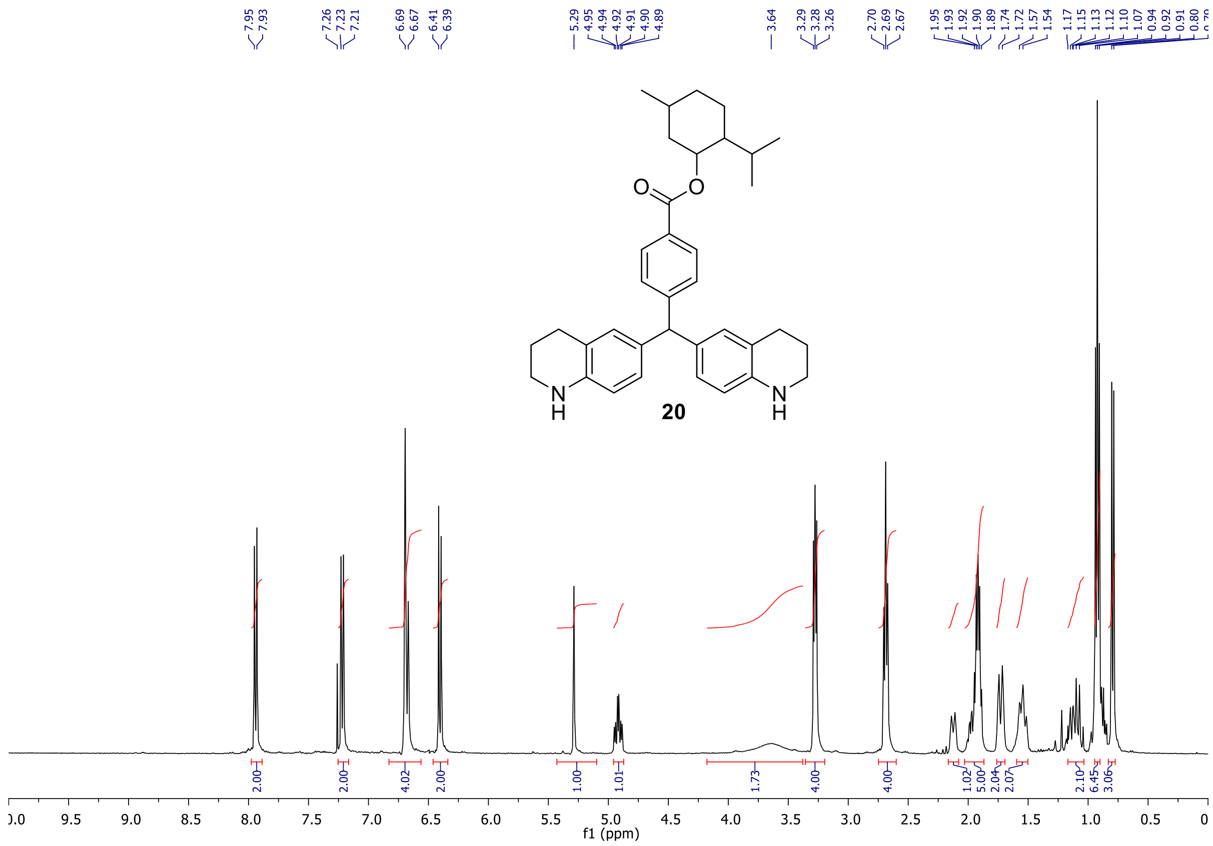

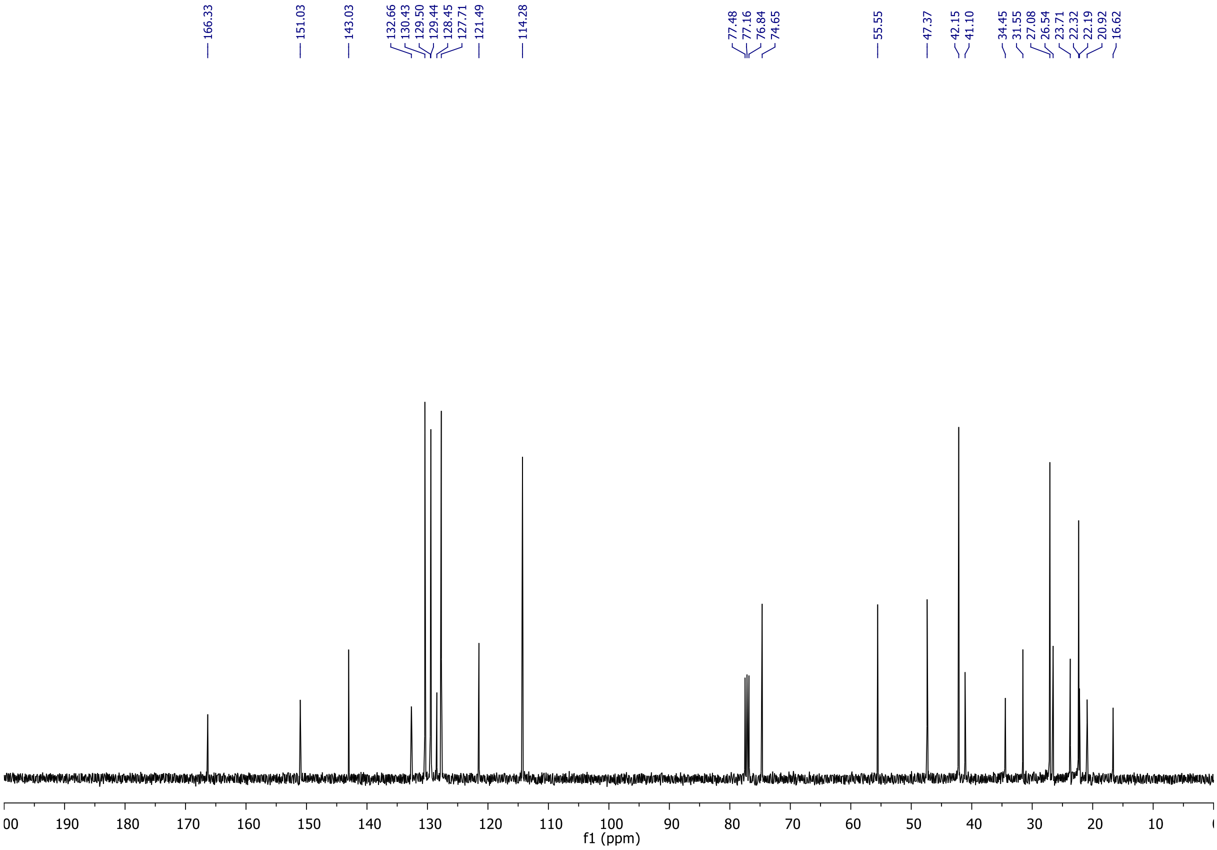

^{1}H NMR (400 MHz) and $^{13}C\{^{1}H\}$ NMR (100 MHz) spectra of **20** ($CDCl_3$)

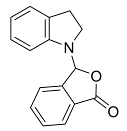

22

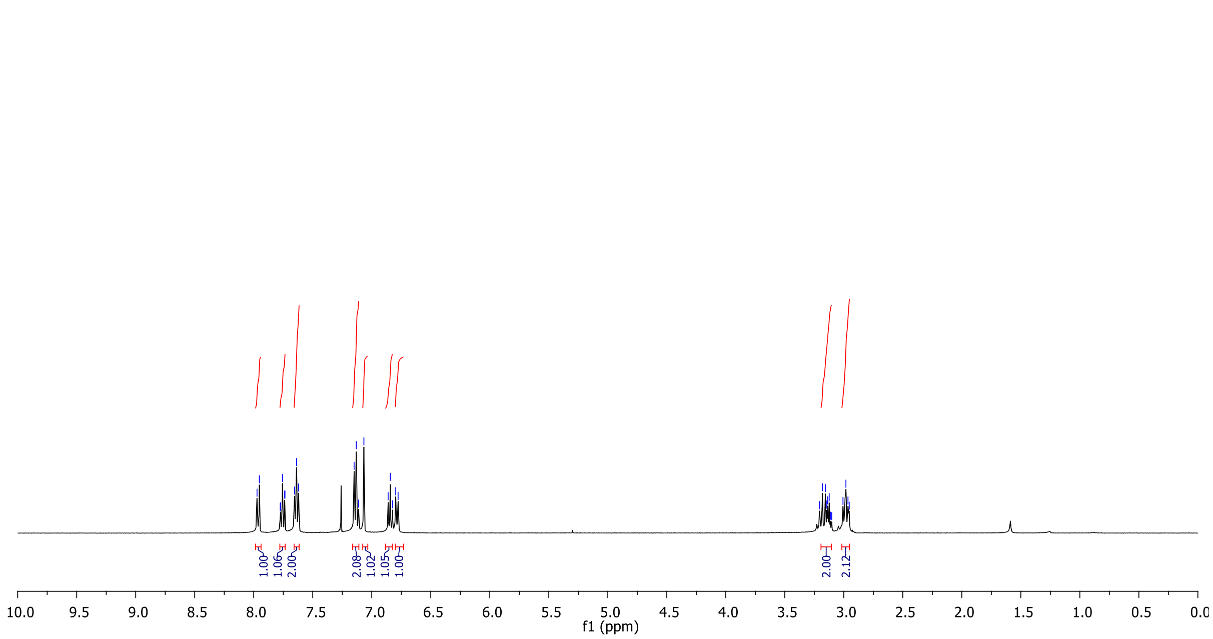

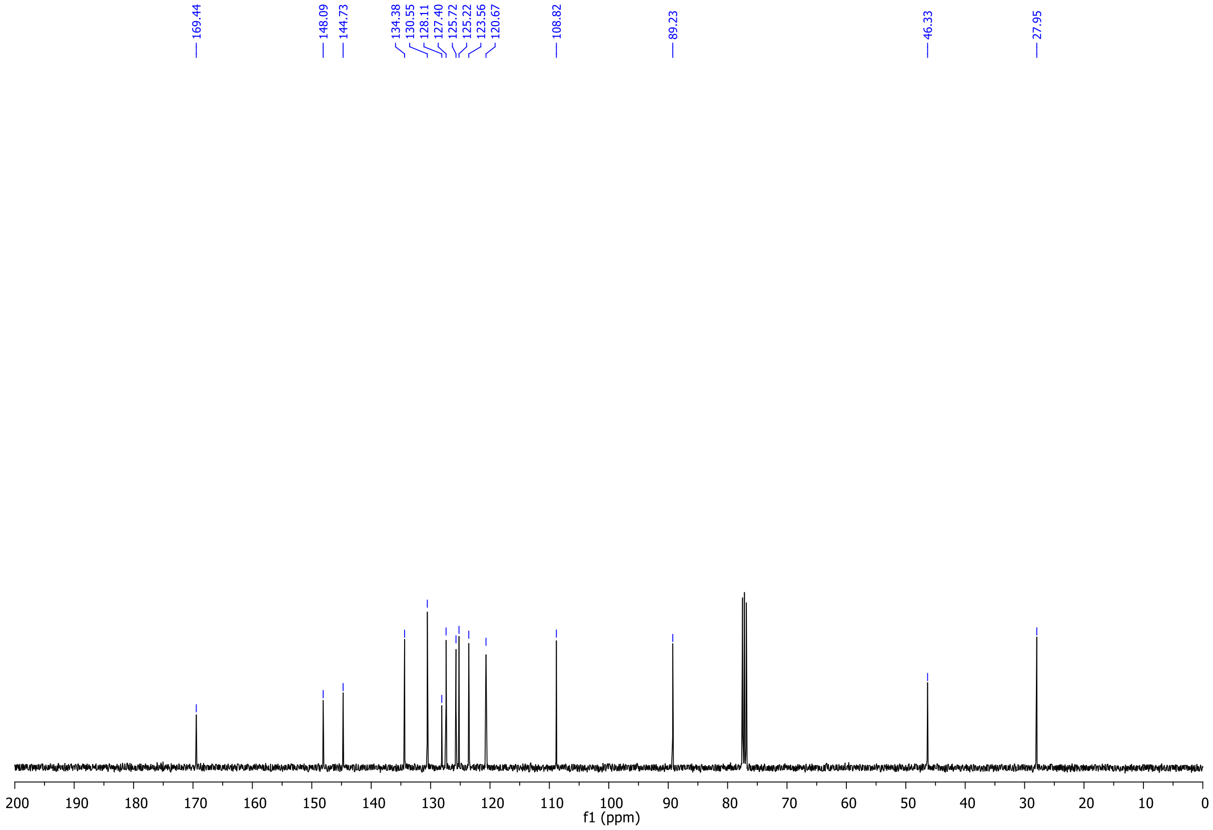

^{1}H NMR (400 MHz) and $^{13}C\{^{1}H\}$ NMR (100 MHz) spectra of **22** ($CDCl_3$)

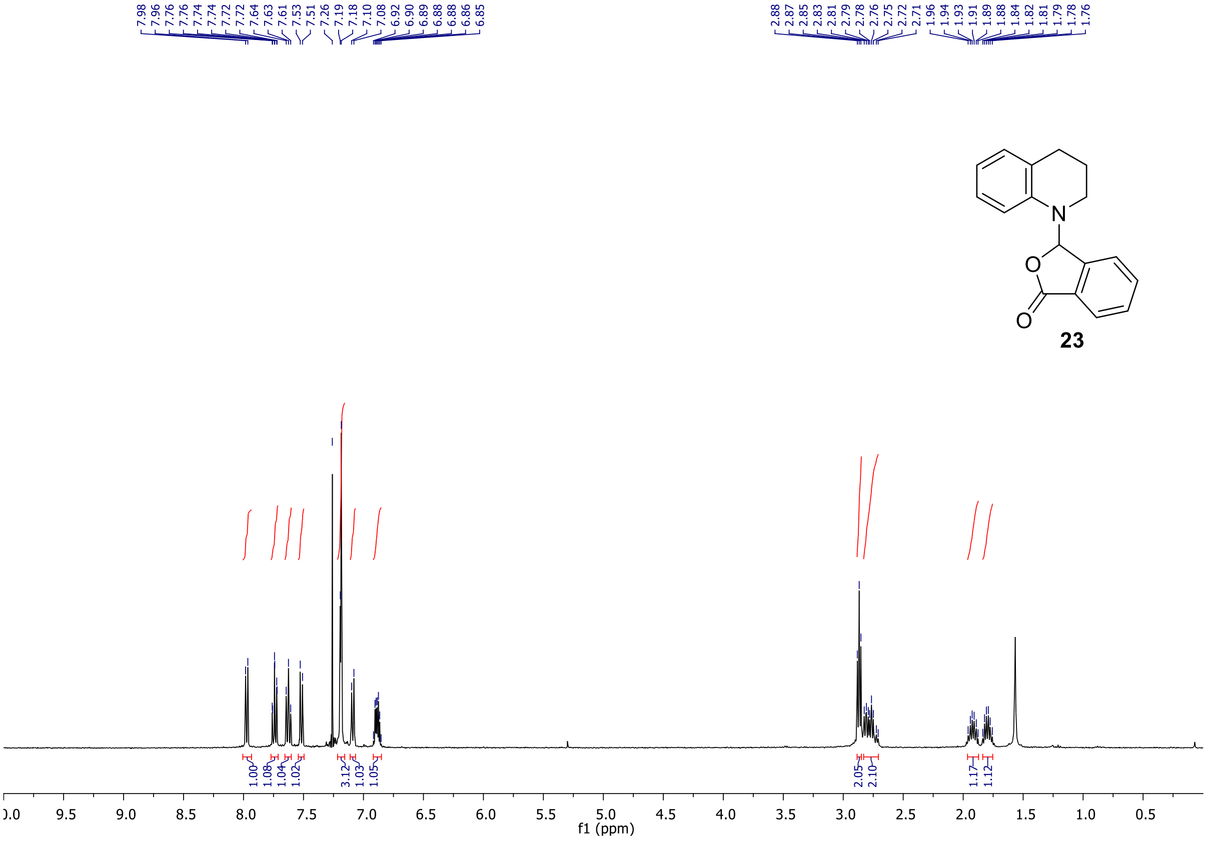

23

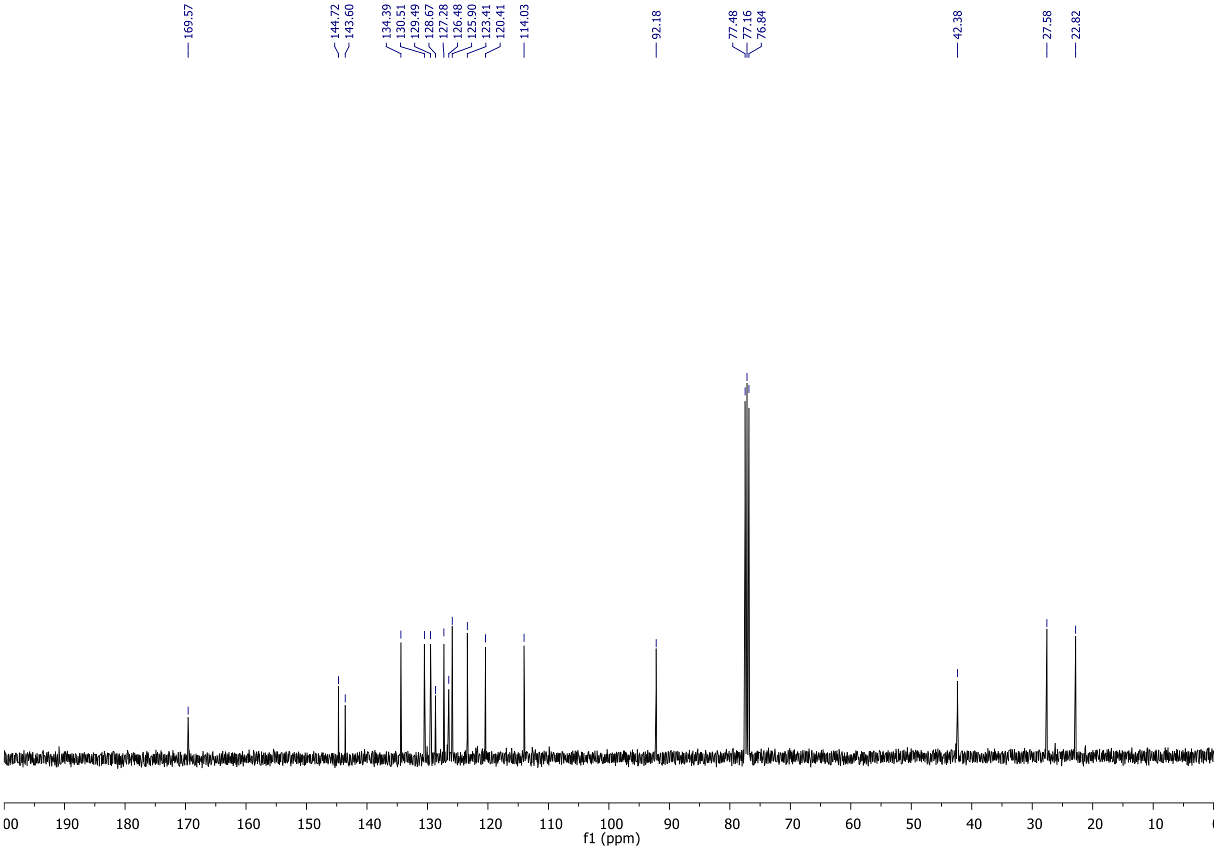

^{1}H NMR (400 MHz) and ^{13}C{^{1}H} NMR (100 MHz) spectra of **23** ($CDCl_3$)

7. HRMS Spectra of Unknown Compounds

HRMS spectrum of 1p

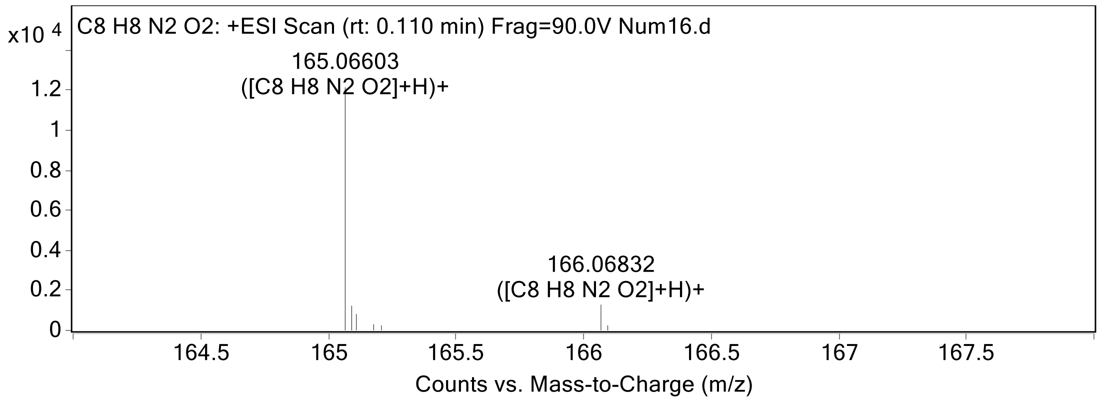

HRMS spectrum of $2y_7$

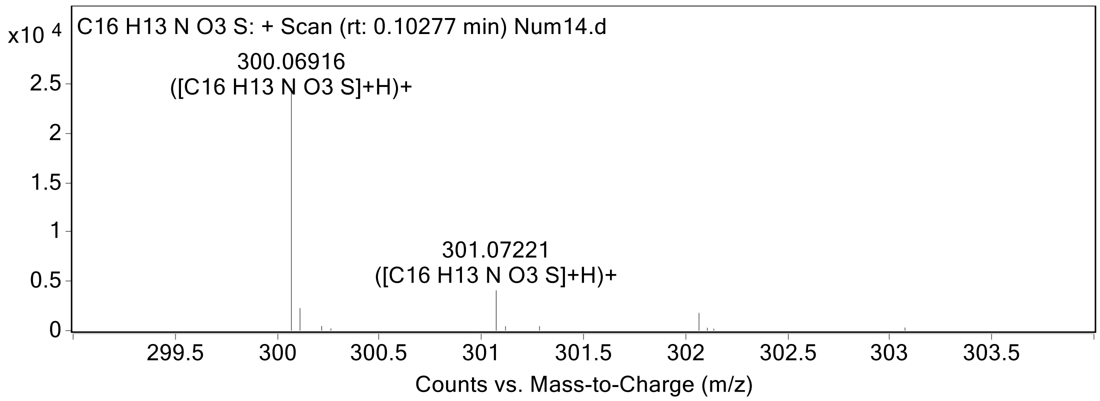

HRMS spectrum of 3aa

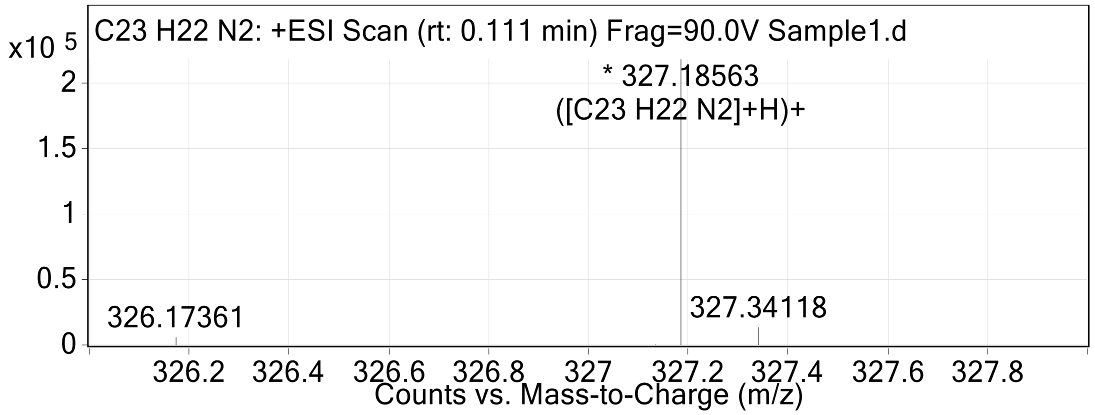

HRMS spectrum of 3ab

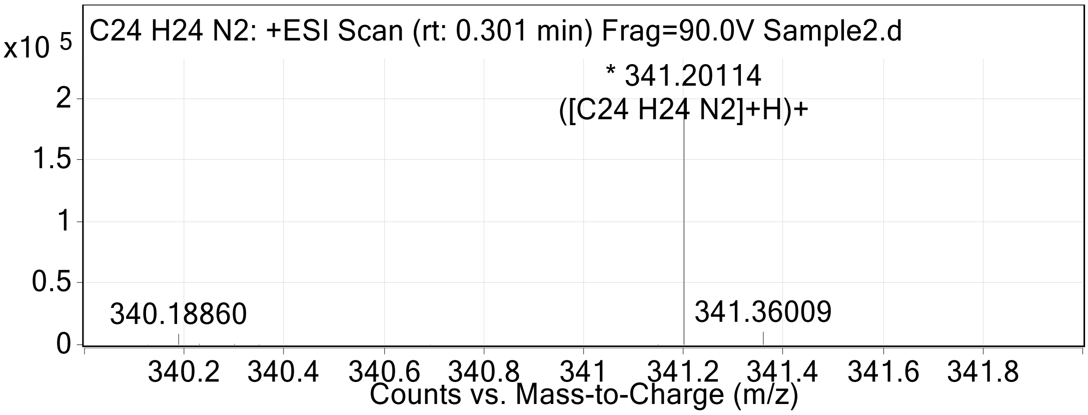

HRMS spectrum of 3ac

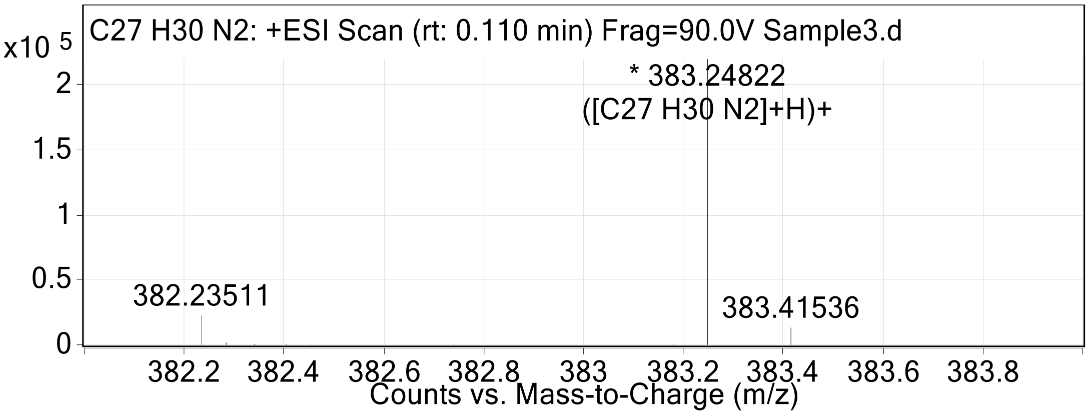

HRMS spectrum of 3ad

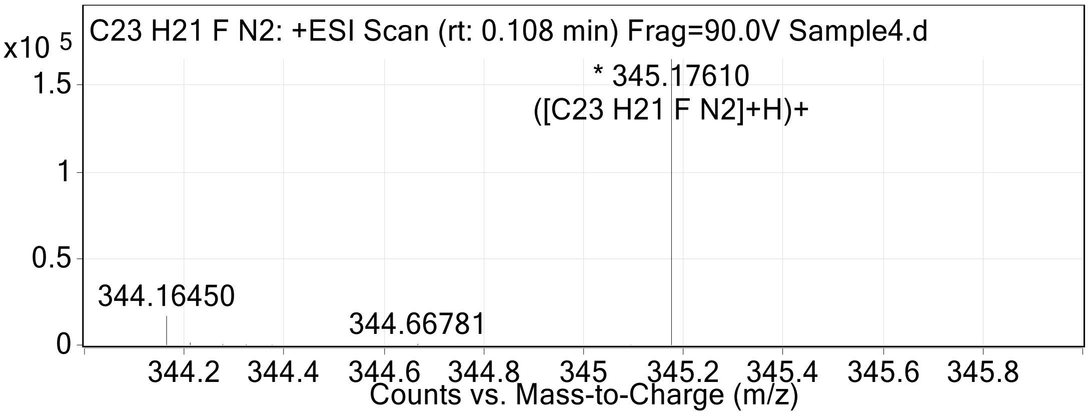

HRMS spectrum of 3ae

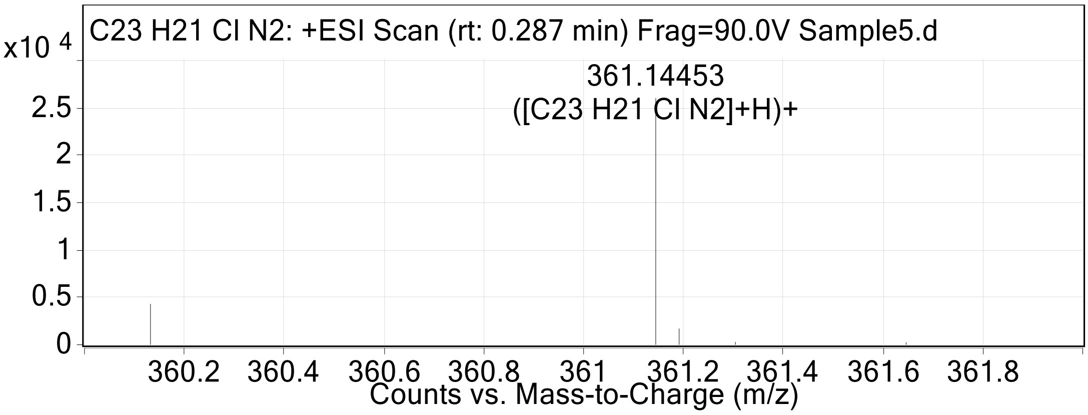

HRMS spectrum of 3af

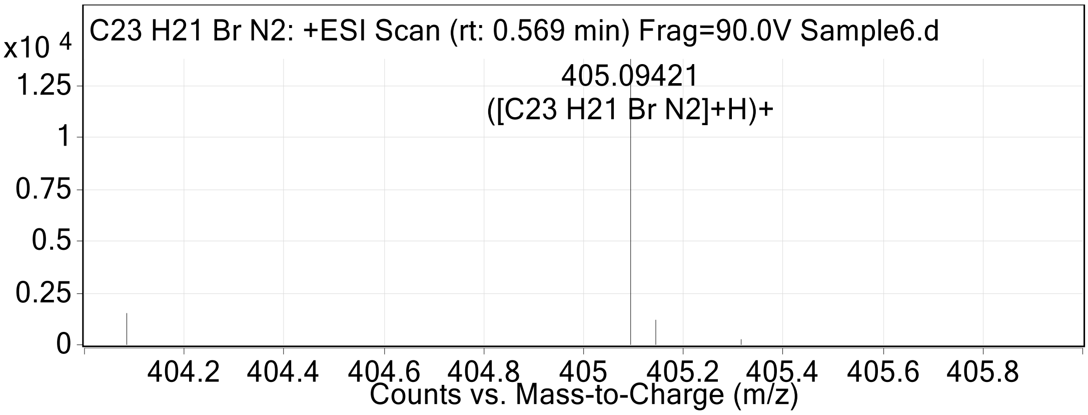

HRMS spectrum of 3ag

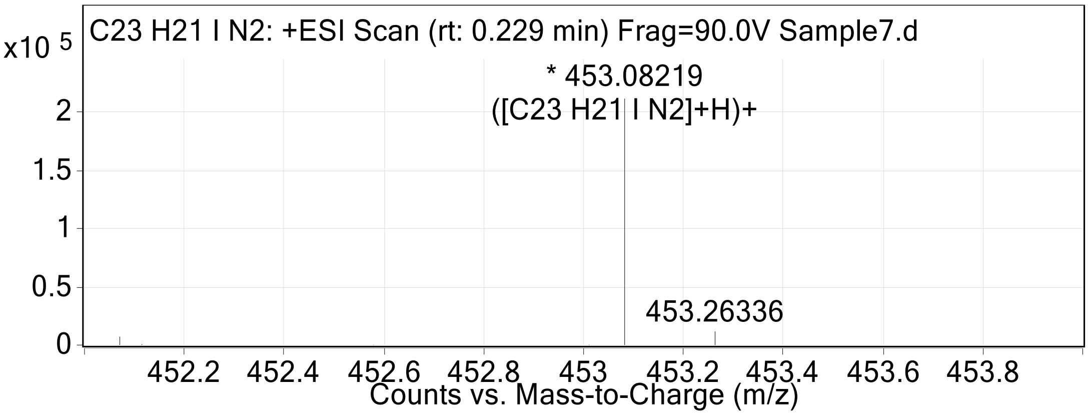

HRMS spectrum of 3ah

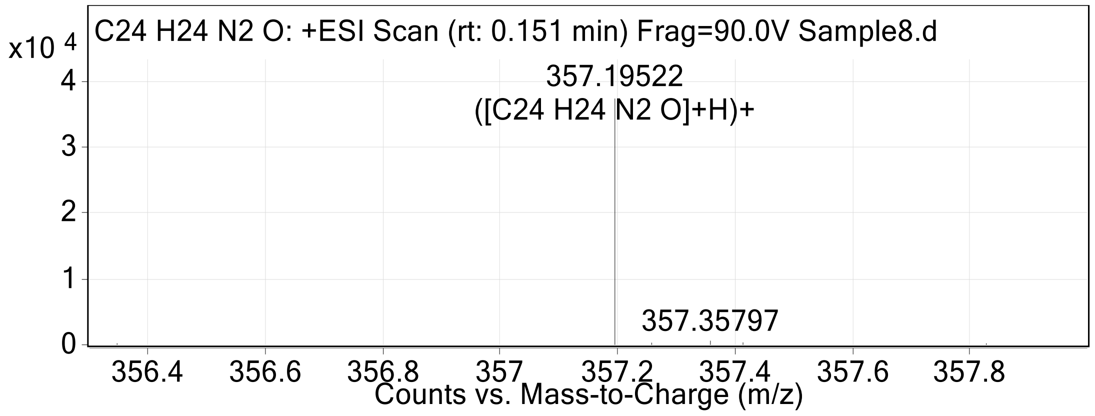

HRMS spectrum of 3aj

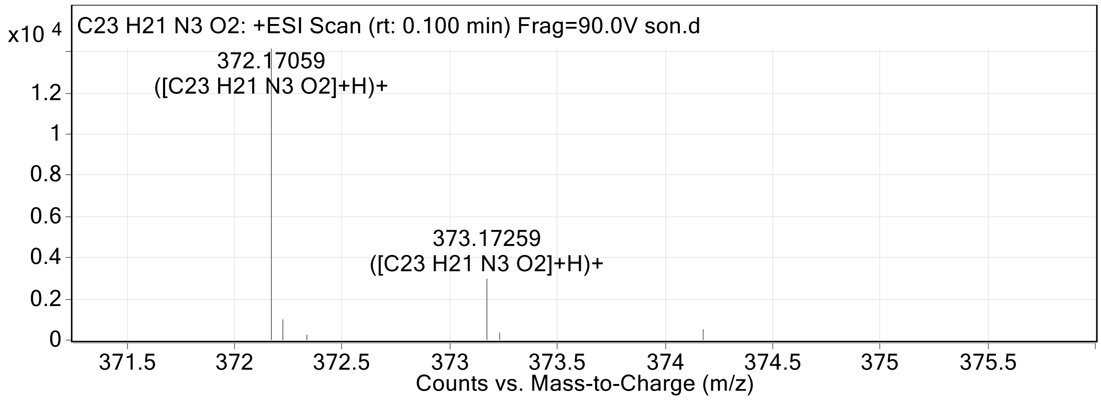

HRMS spectrum of 3ak

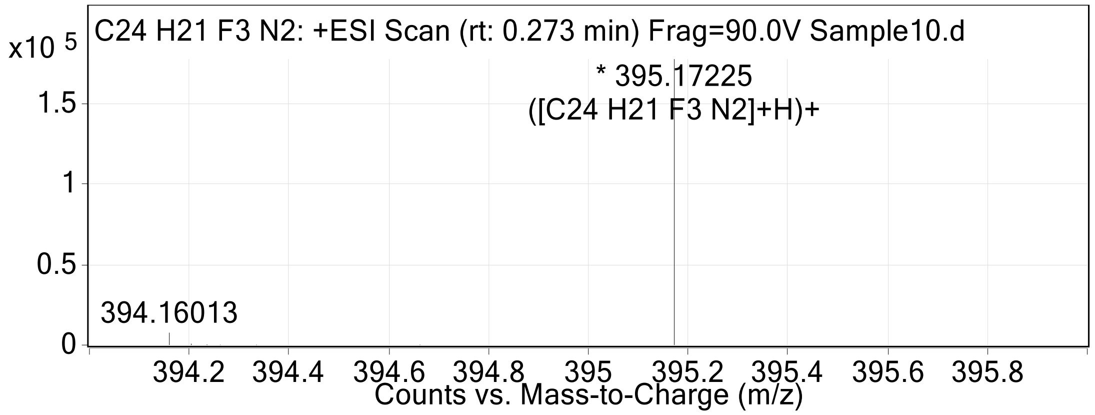

HRMS spectrum of 3al

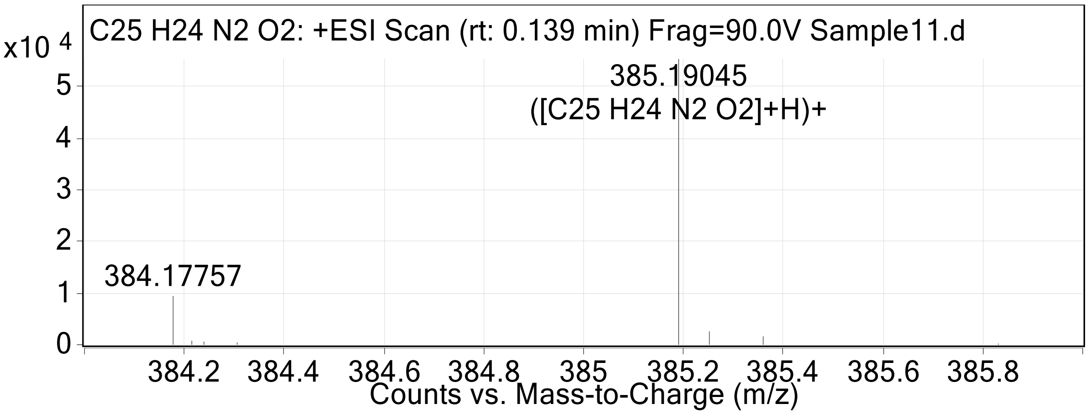

HRMS spectrum of 3am

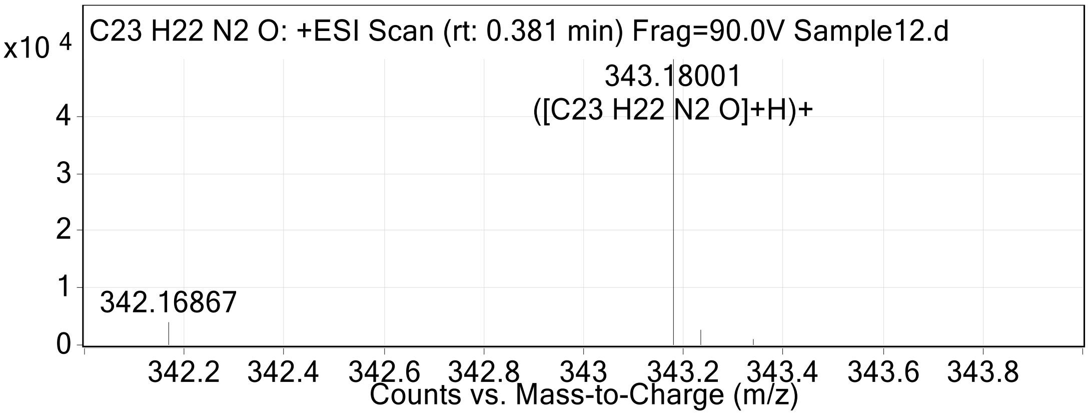

HRMS spectrum of 3an

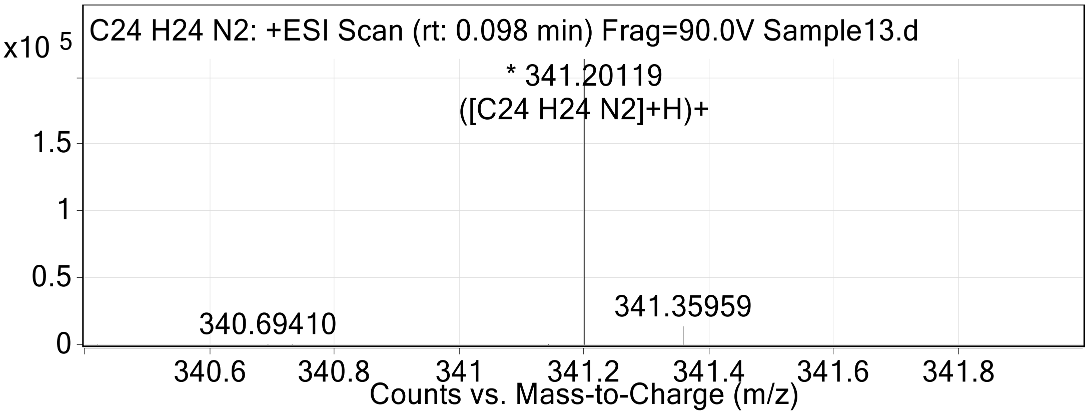

HRMS spectrum of 3ao

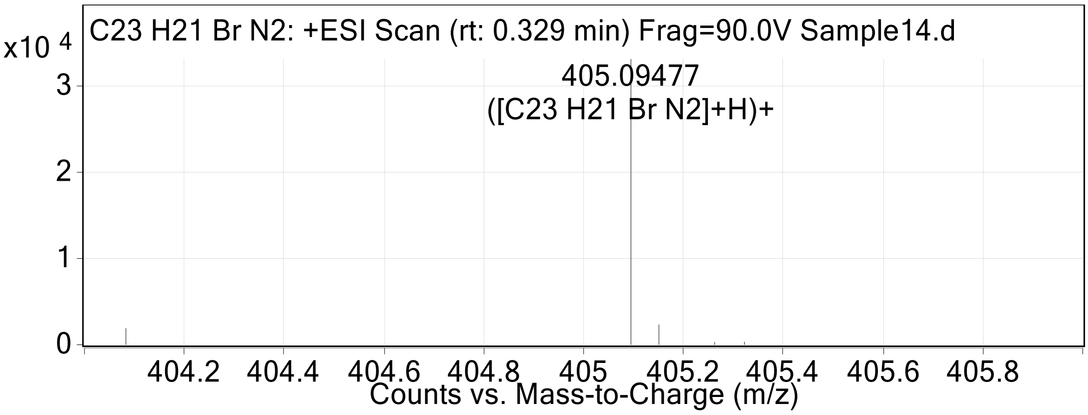

HRMS spectrum of 3ap

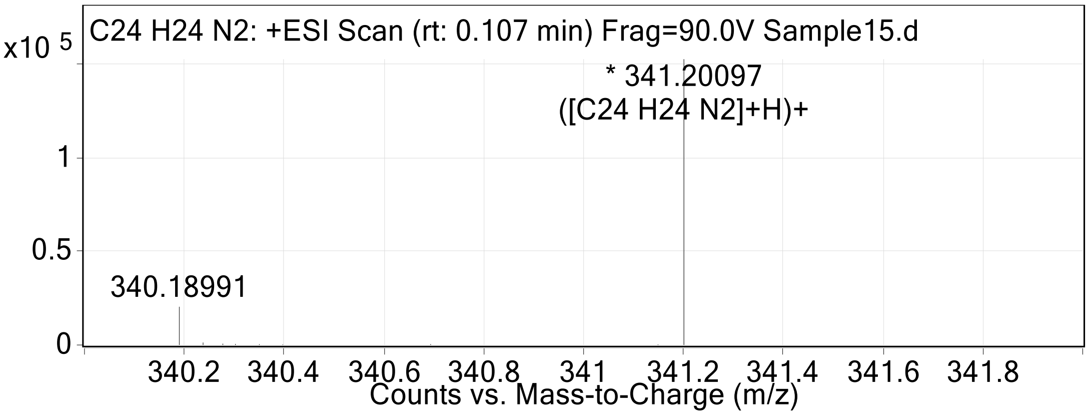

HRMS spectrum of 3aq

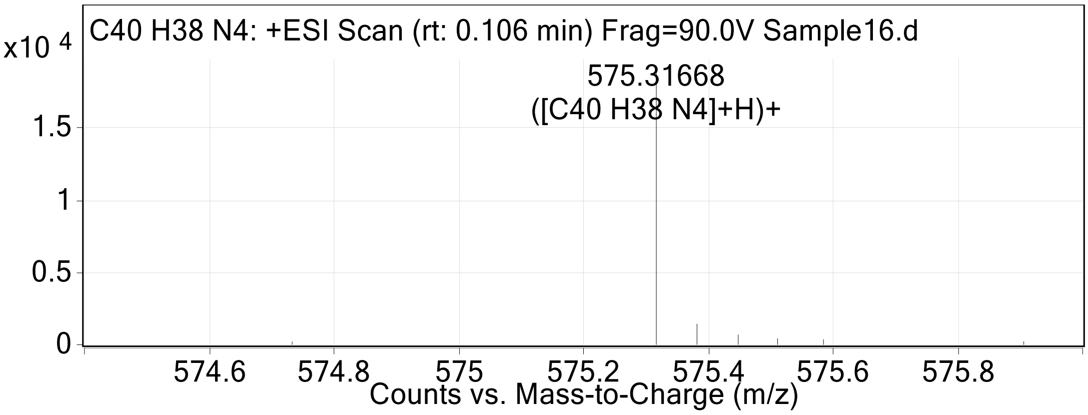

HRMS spectrum of 3ar

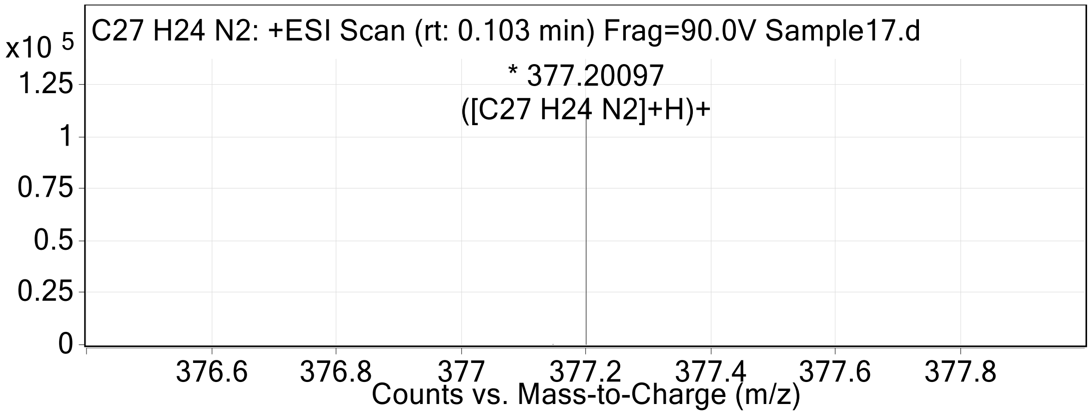

HRMS spectrum of 3au$_2$

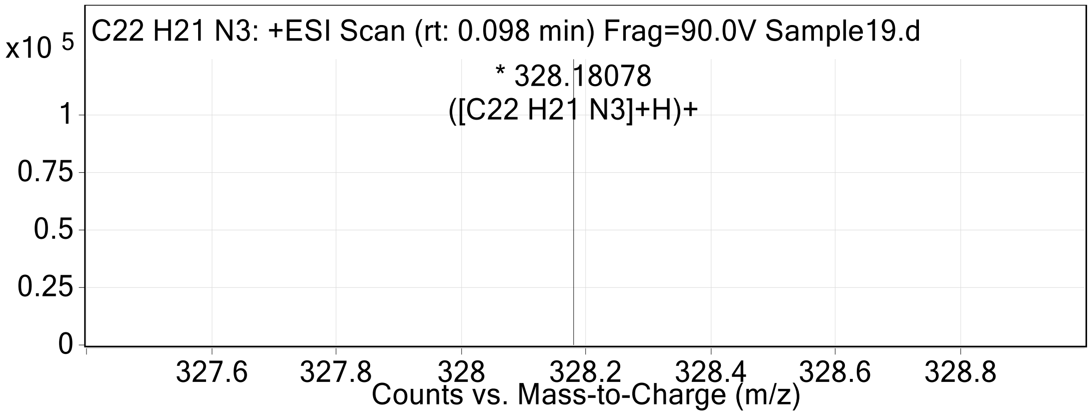

HRMS spectrum of 3au$_3$

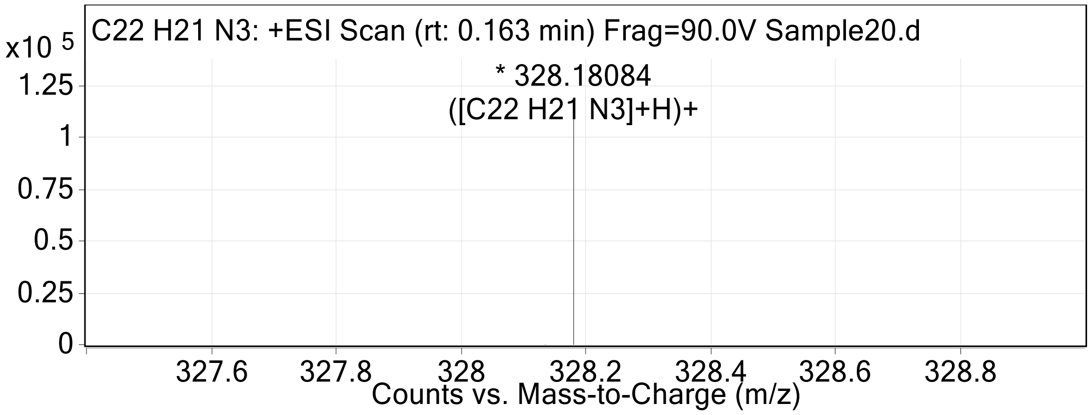

HRMS spectrum of 3au$_4$

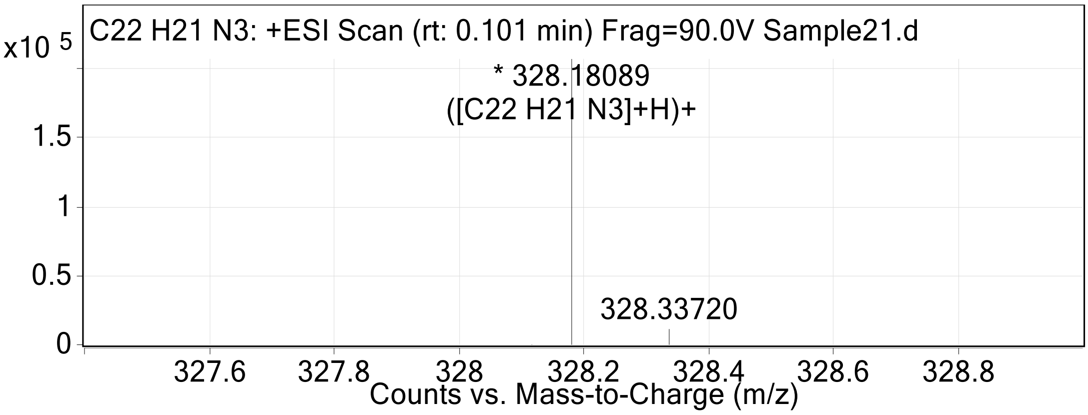

HRMS spectrum of 3av$_2$

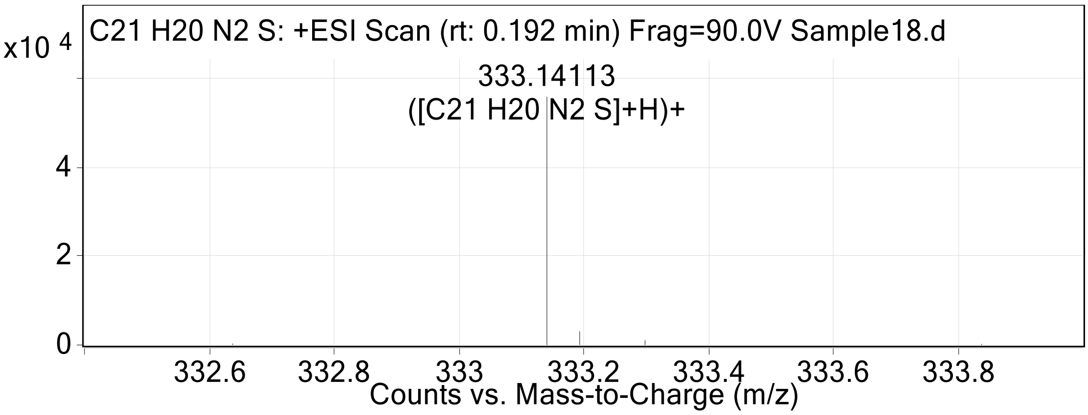

HRMS spectrum of 3ay$_3$

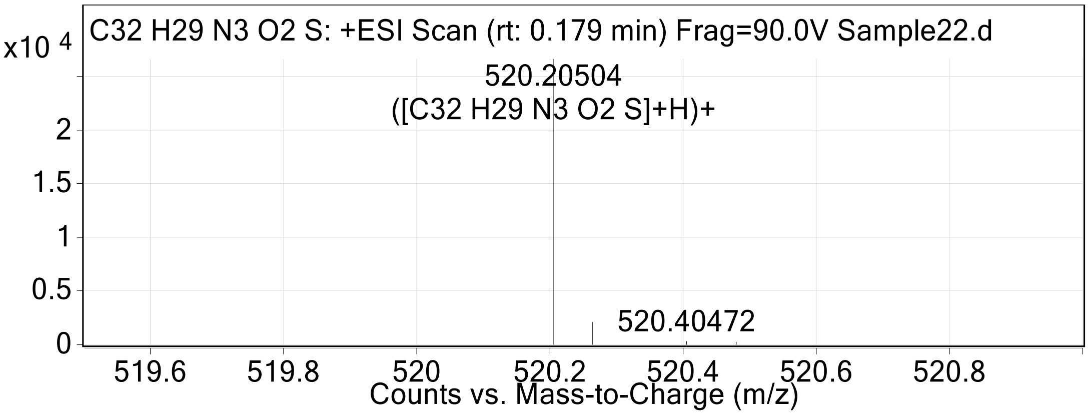

HRMS spectrum of 3ay$_4$

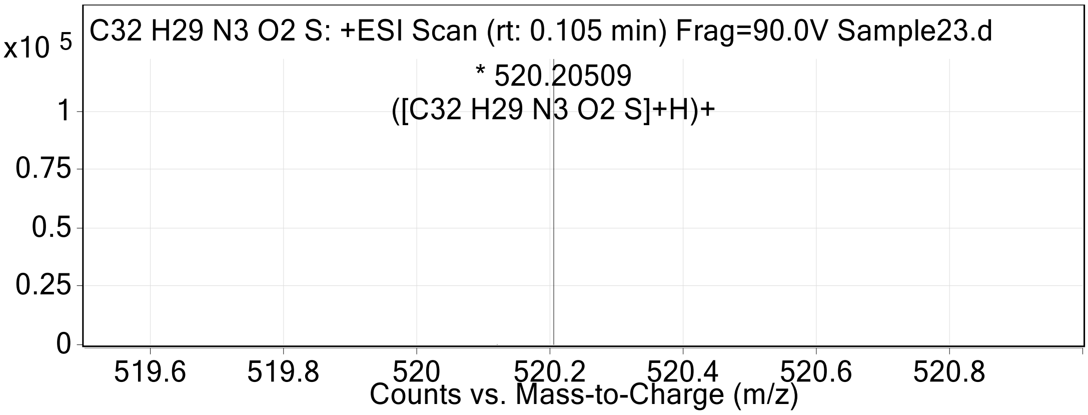

HRMS spectrum of 3ay$_5$

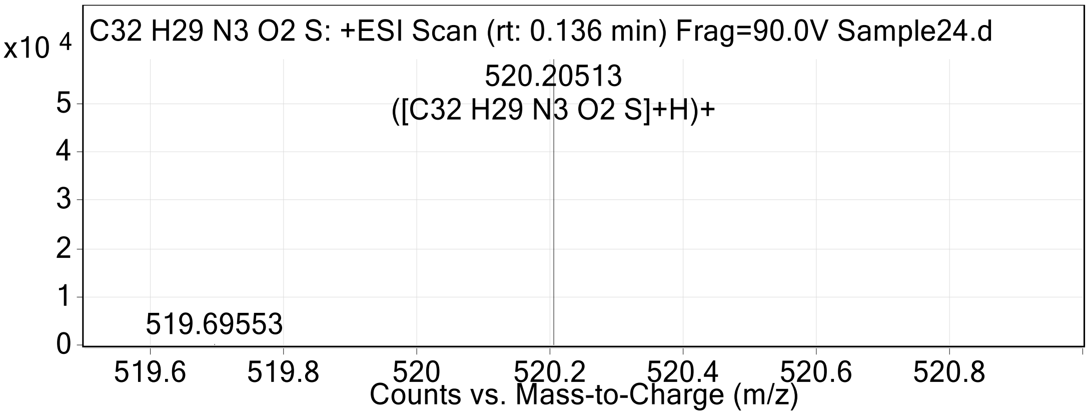

HRMS spectrum of 3ay$_6$

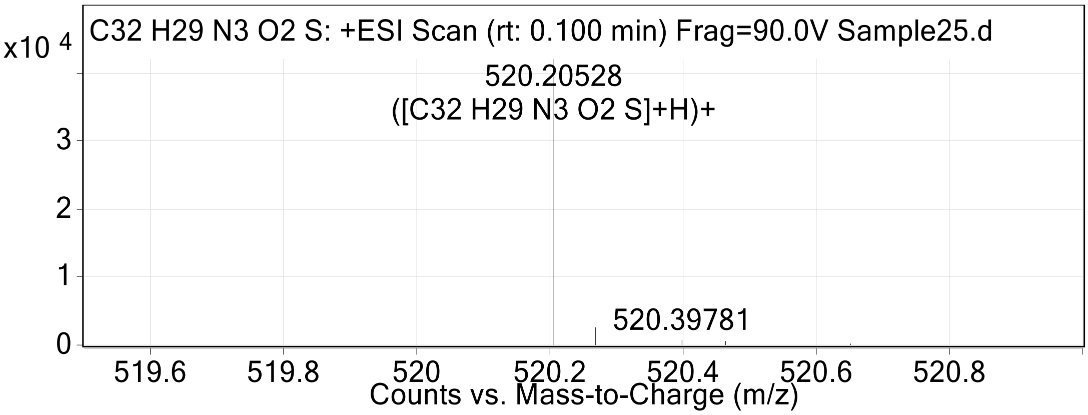

HRMS spectrum of 3cj

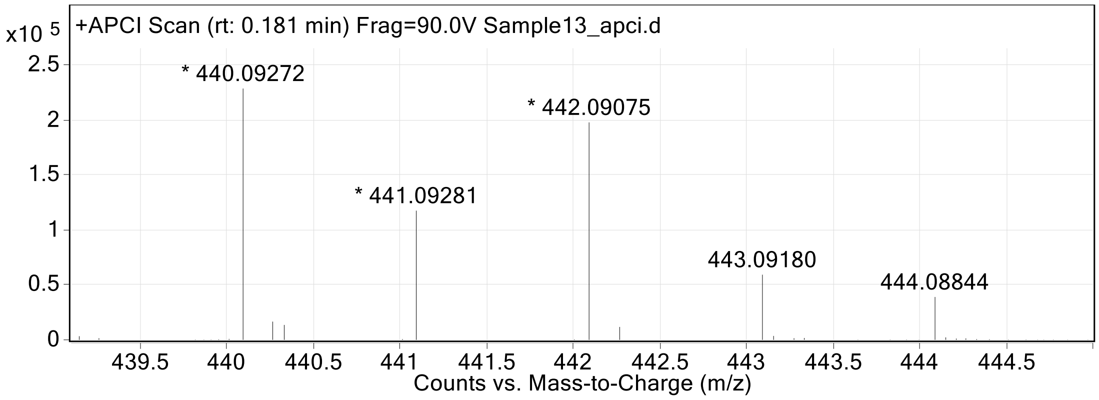

HRMS spectrum of 3gj

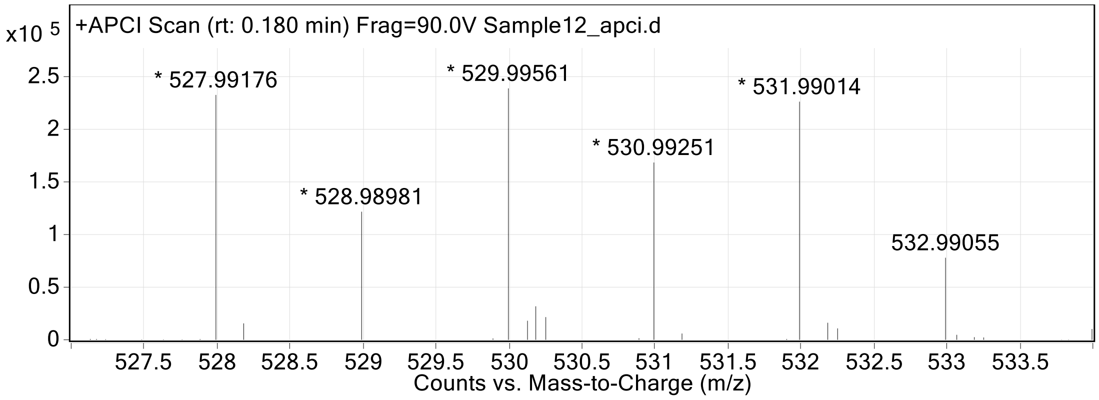

HRMS spectrum of 3jj

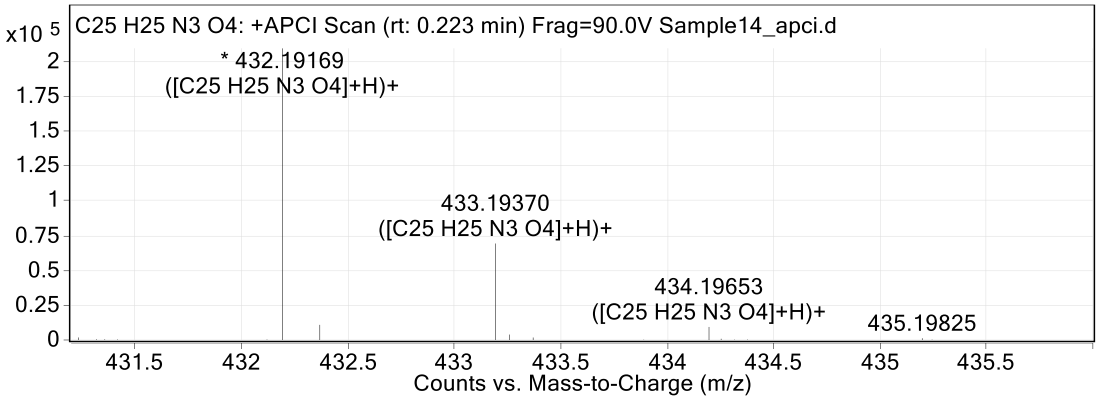

HRMS spectrum of 3nj

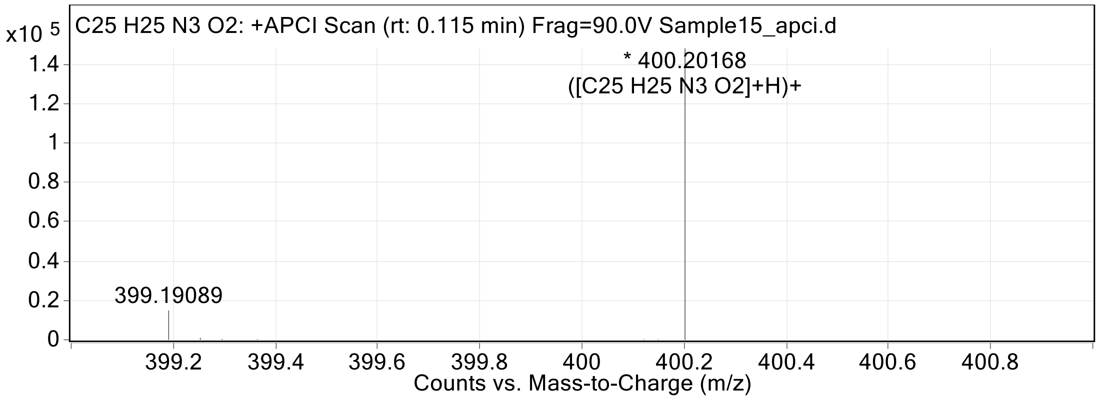

HRMS spectrum of 4af

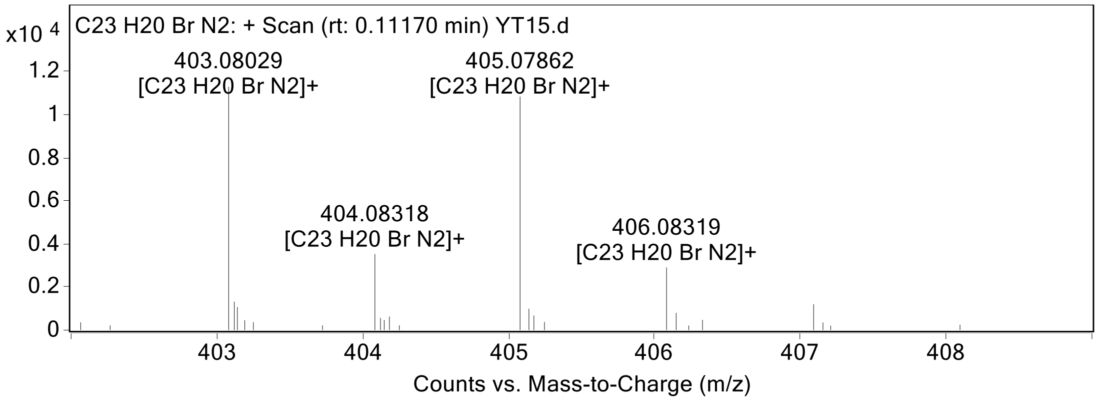

HRMS spectrum of 9ej

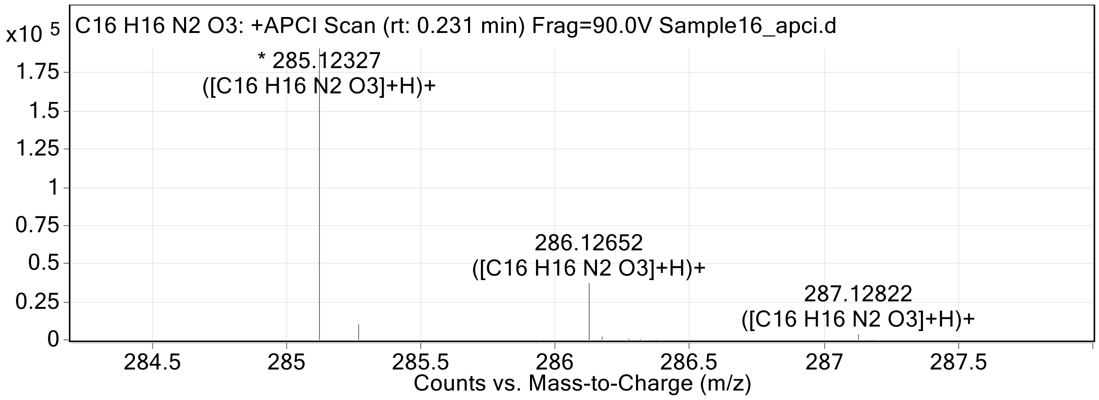

HRMS spectrum of 9hj

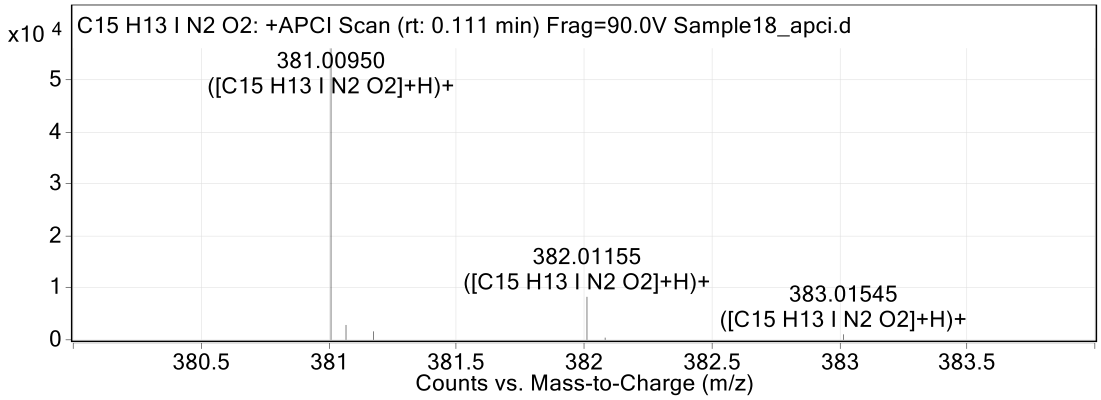

HRMS spectrum of 9ij

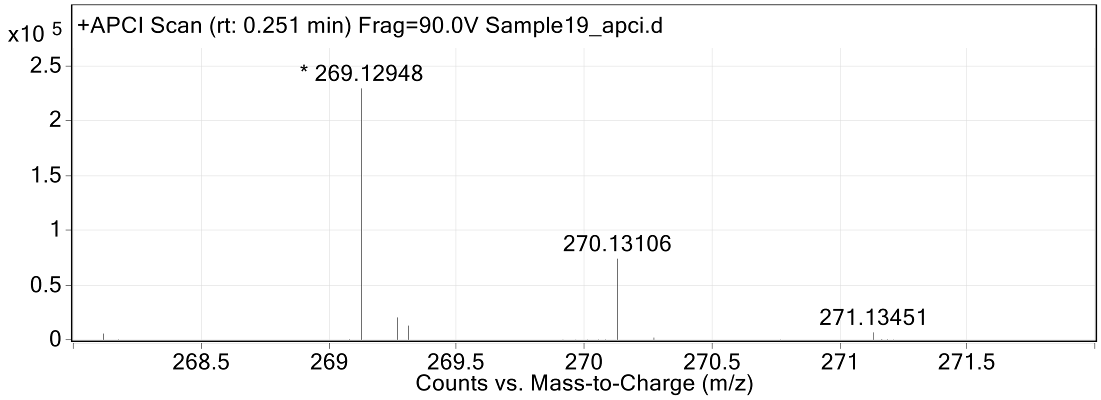

HRMS spectrum of 9mj

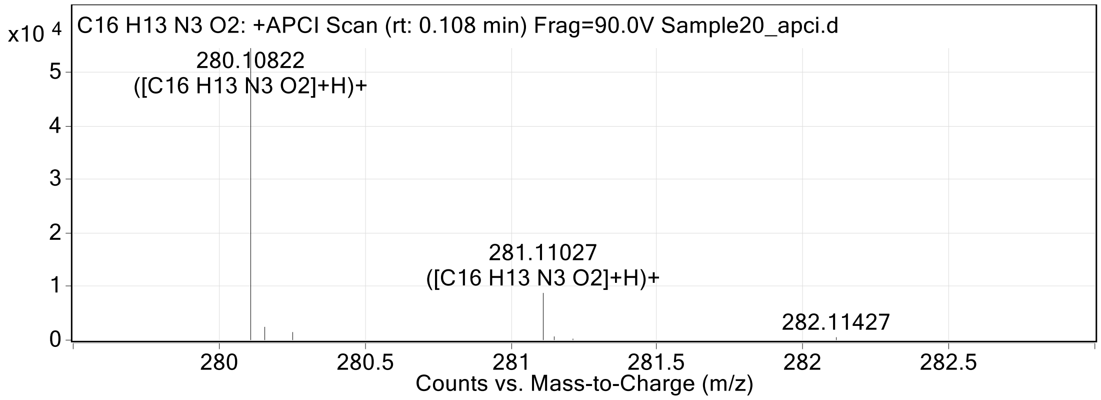

HRMS spectrum of 10b

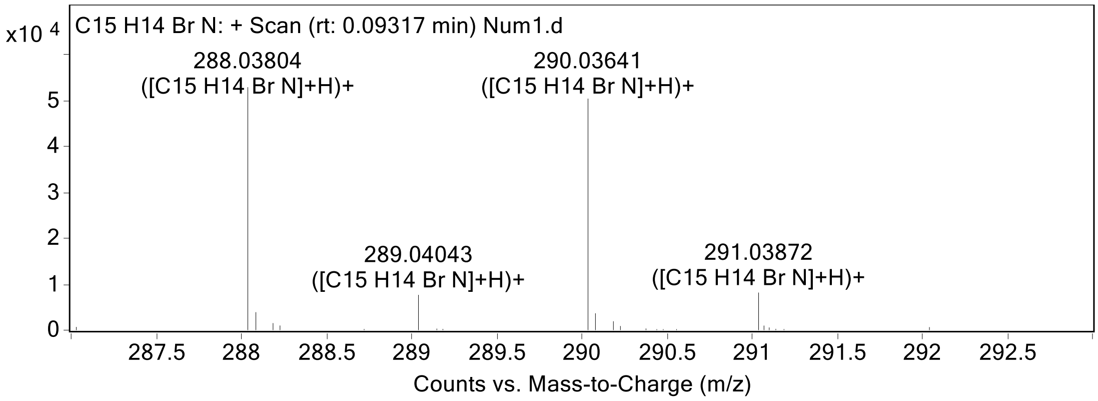

HRMS spectrum of 10c

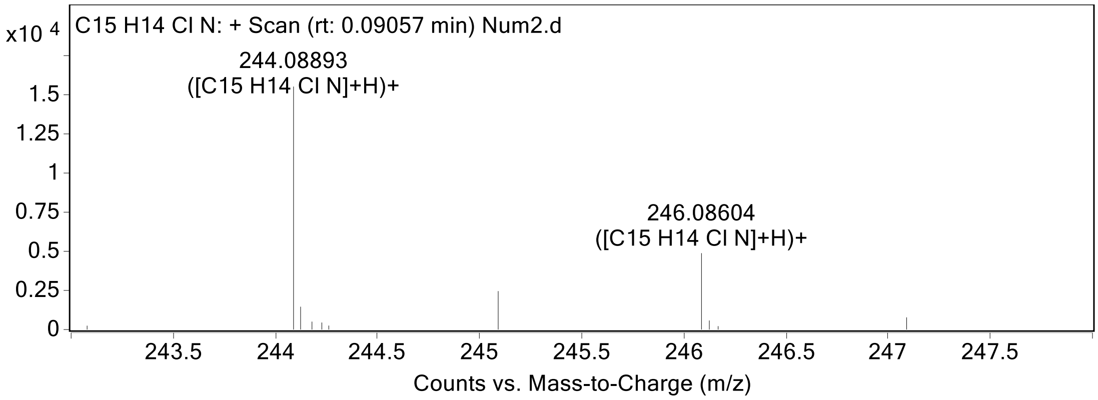

HRMS spectrum of 10e

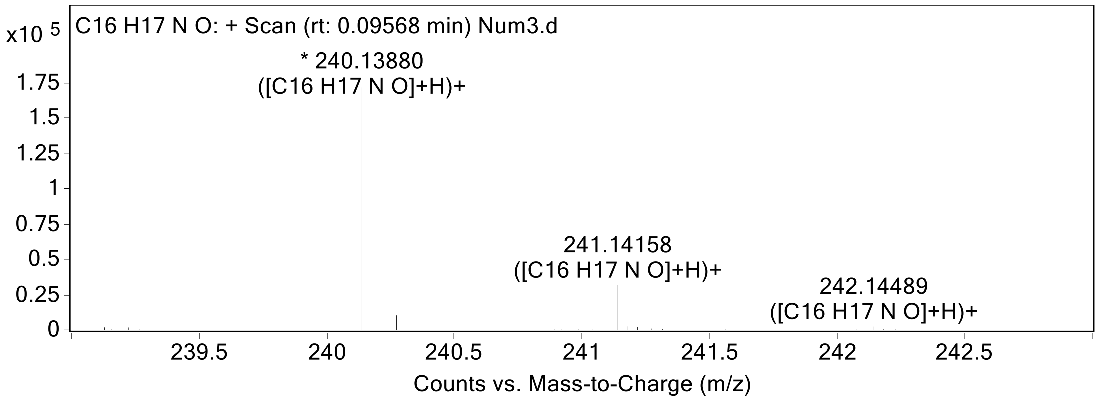

HRMS spectrum of 10f

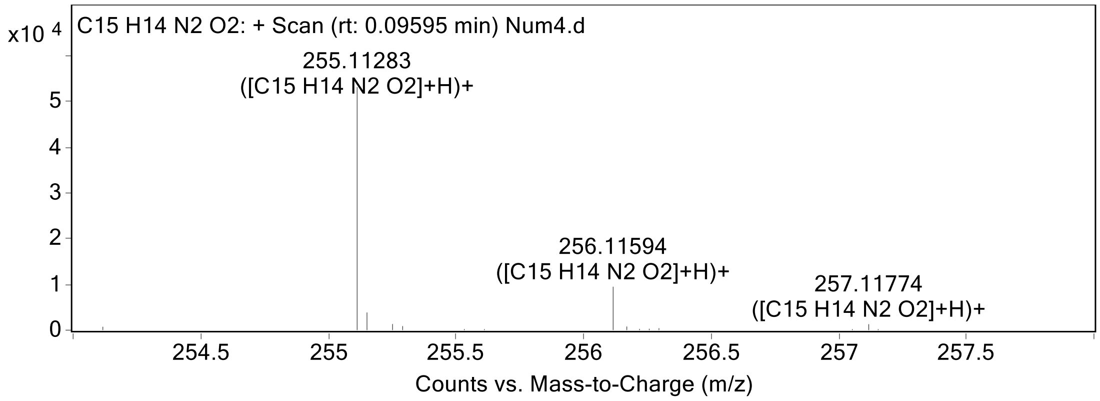

HRMS spectrum of 10h

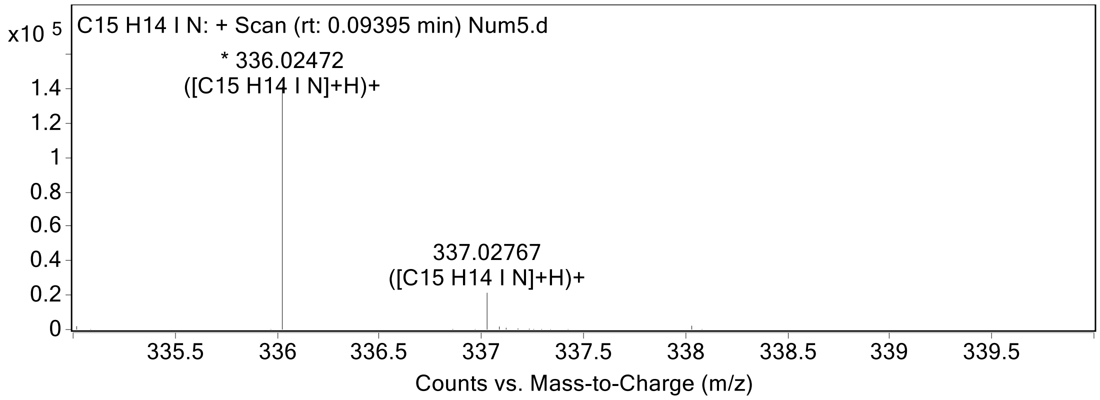

HRMS spectrum of 10k

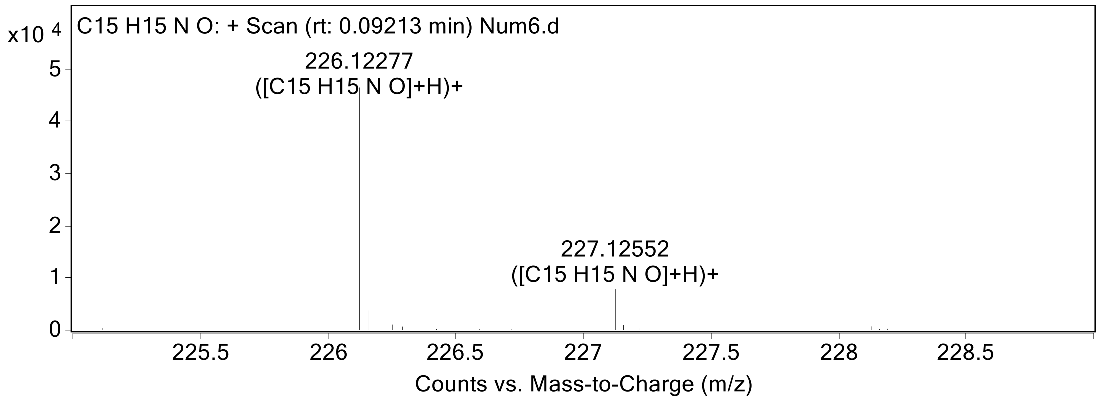

HRMS spectrum of 10l

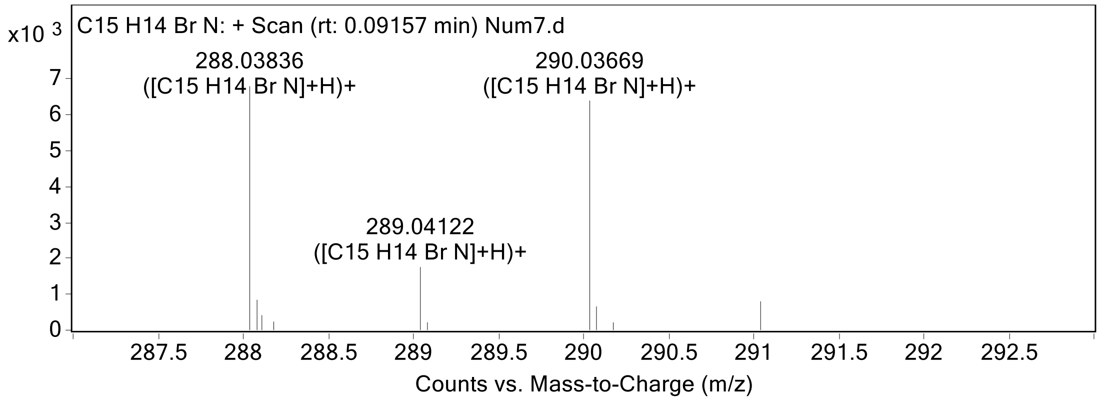

HRMS spectrum of 10m

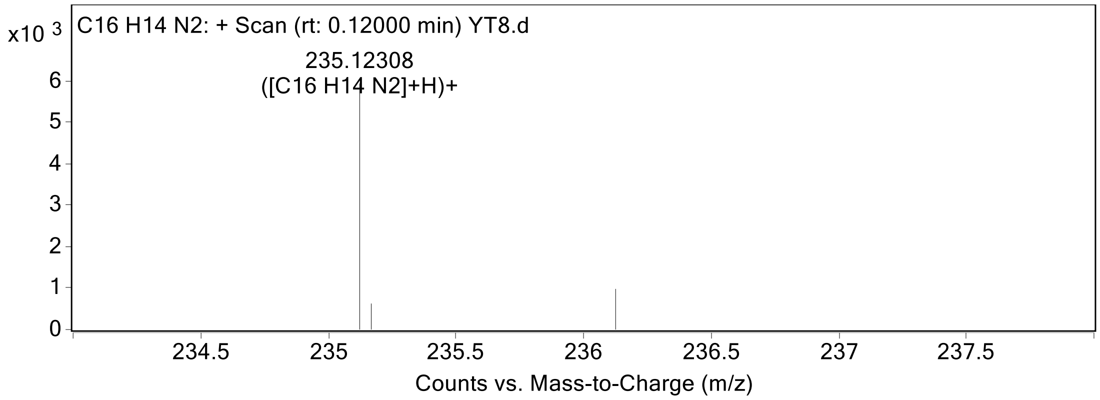

HRMS spectrum of 10n

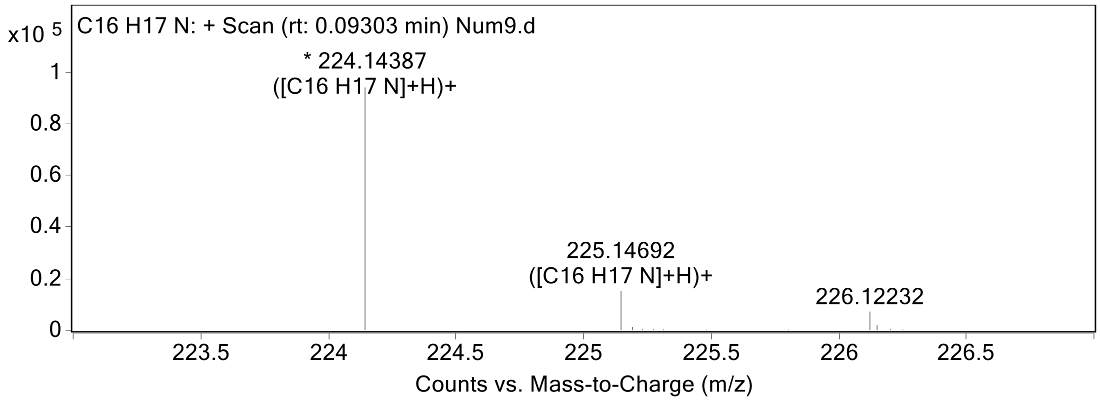

HRMS spectrum of 10p

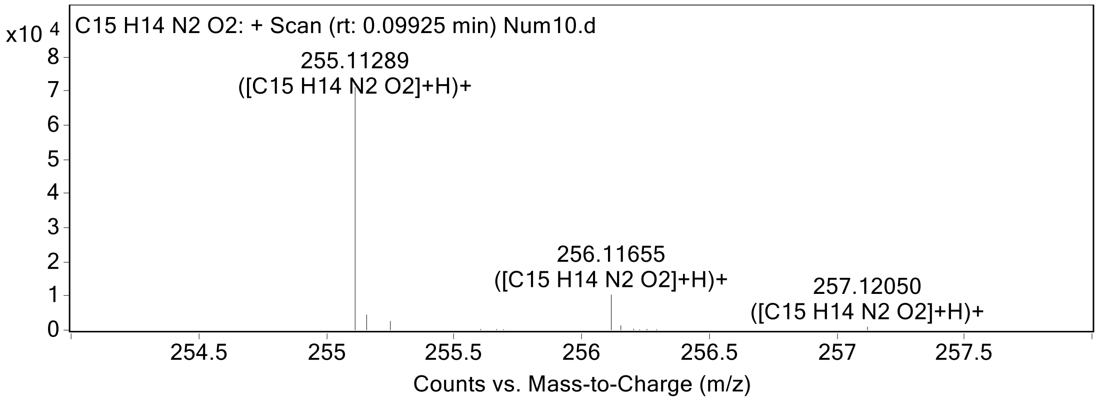

HRMS spectrum of 11bj

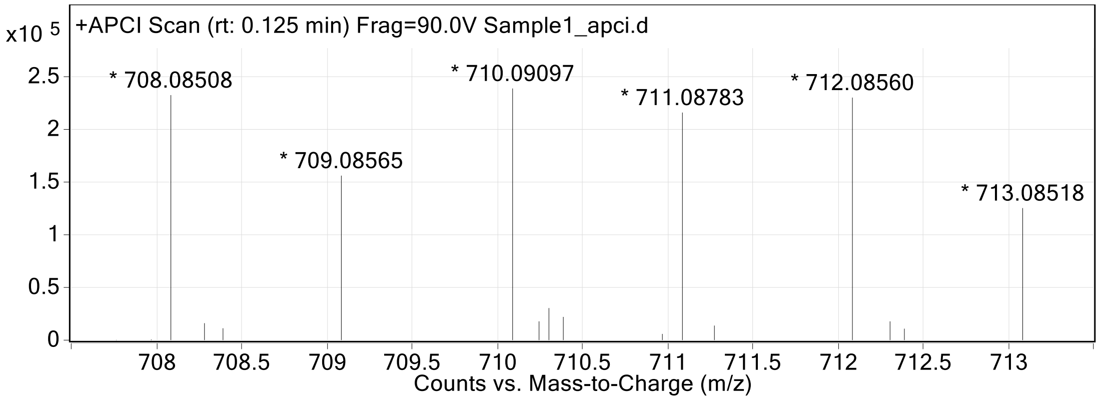

HRMS spectrum of 11cj

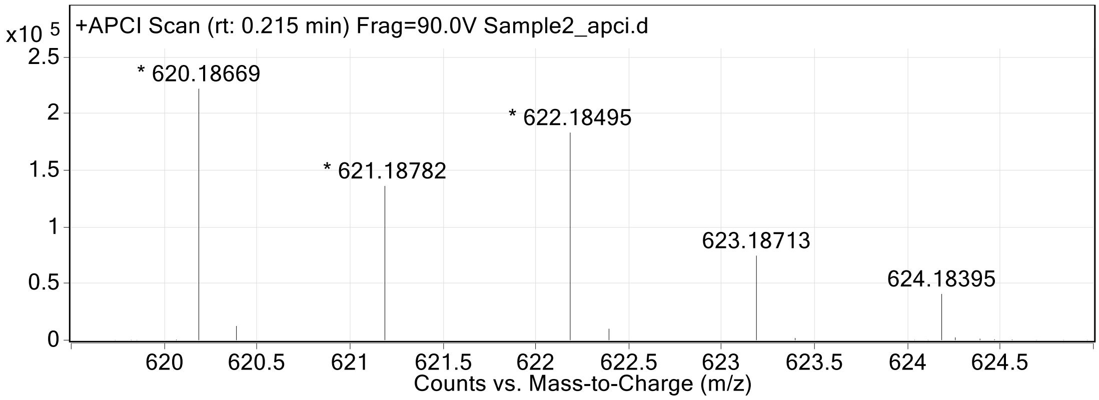

HRMS spectrum of 11dj

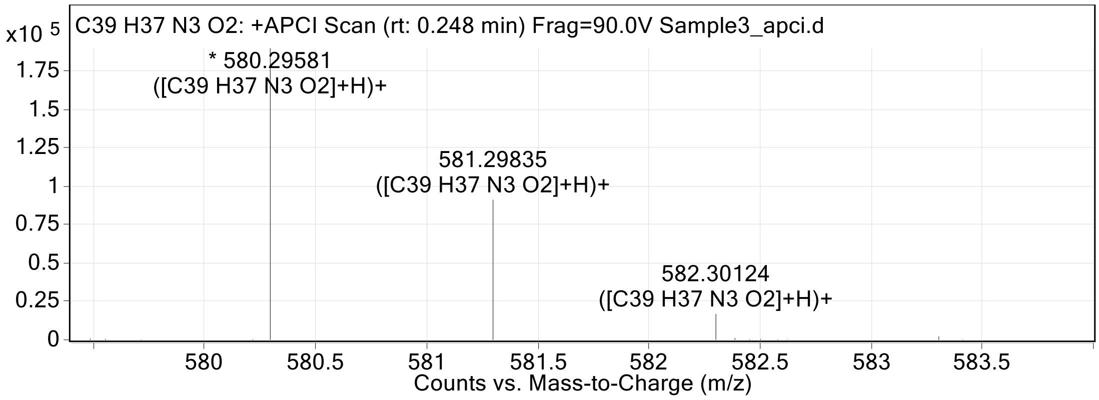

HRMS spectrum of 11ej

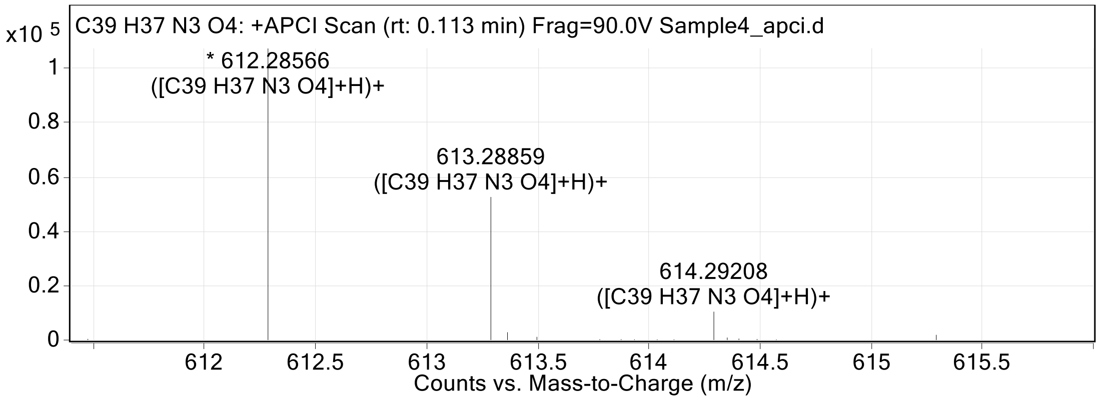

HRMS spectrum of 11gj

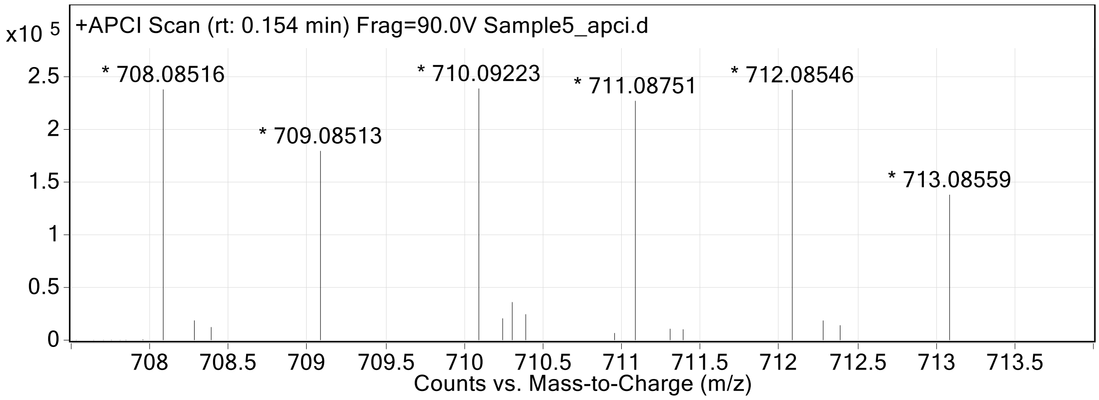

HRMS spectrum of 11hj

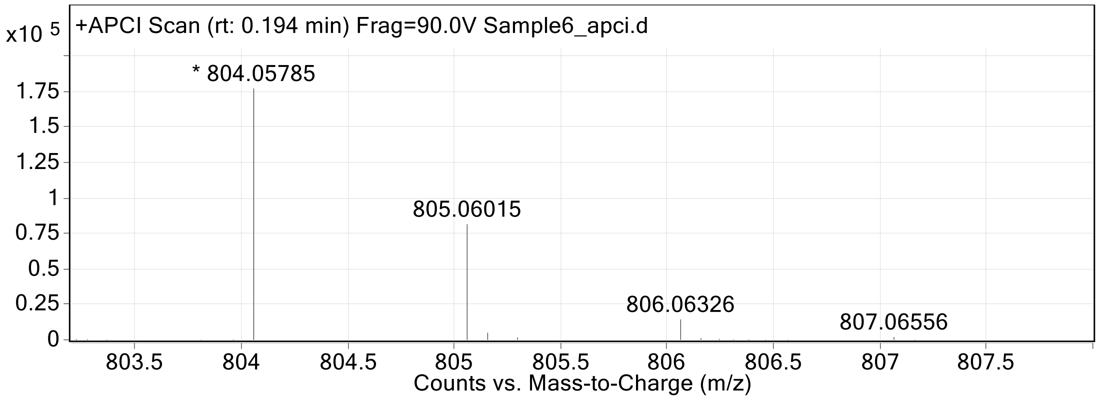

HRMS spectrum of 11ij

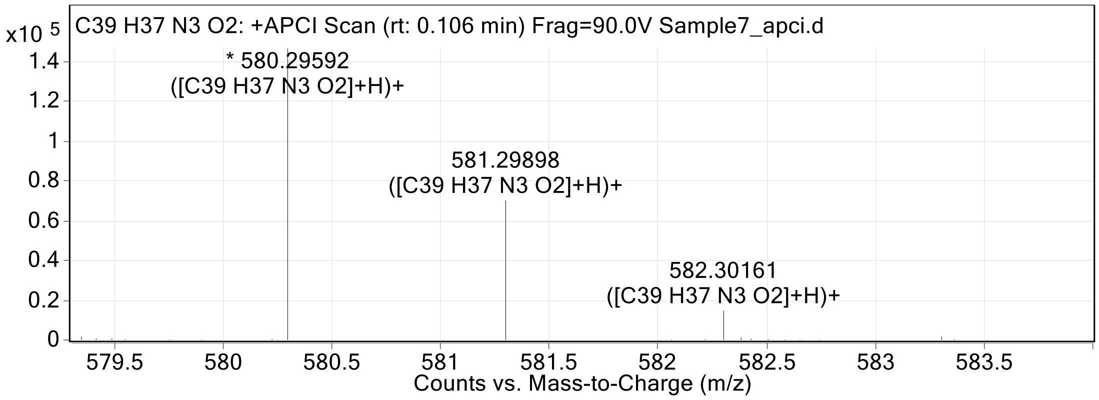

HRMS spectrum of 11jj

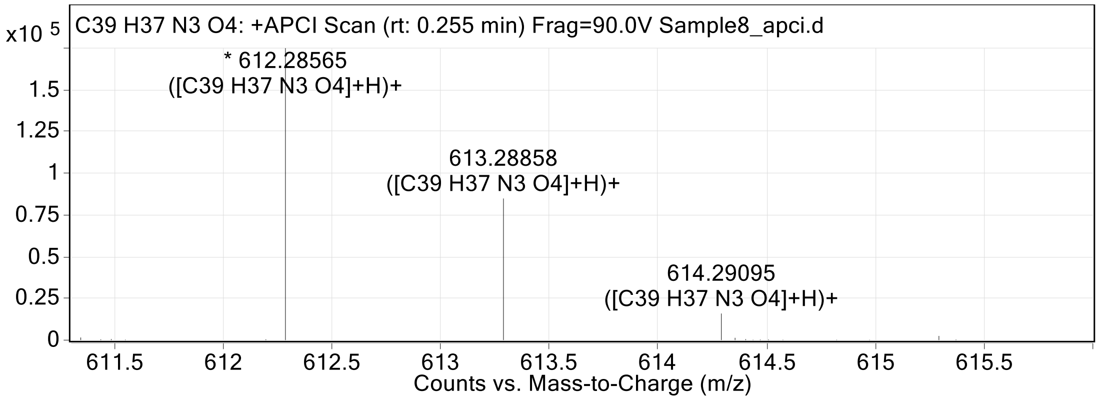

HRMS spectrum of 11kj

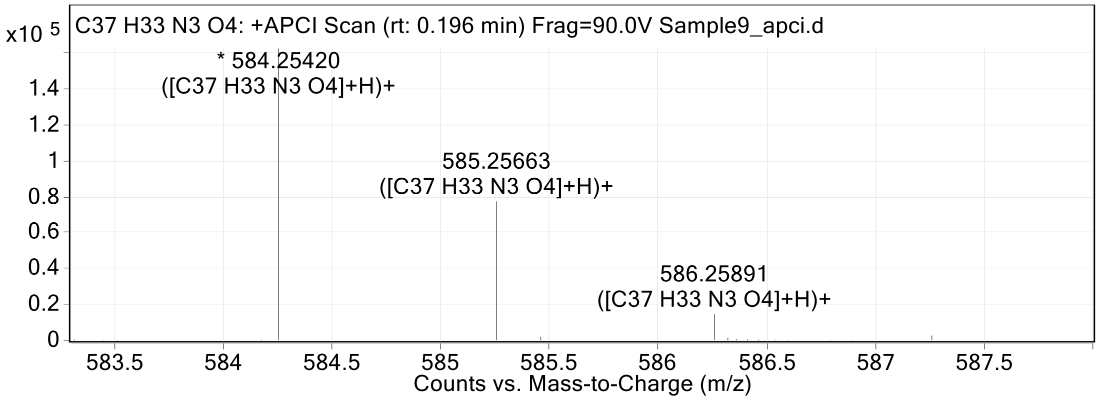

HRMS spectrum of 11nj

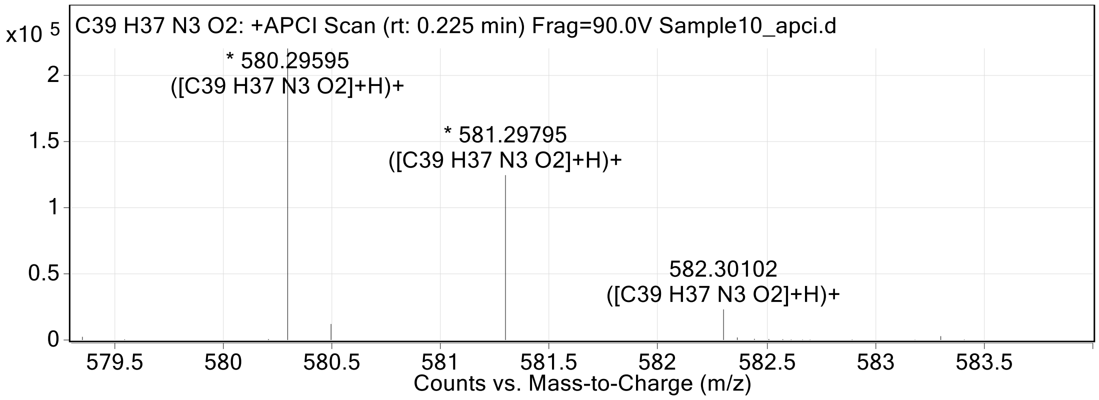

HRMS spectrum of 11oj

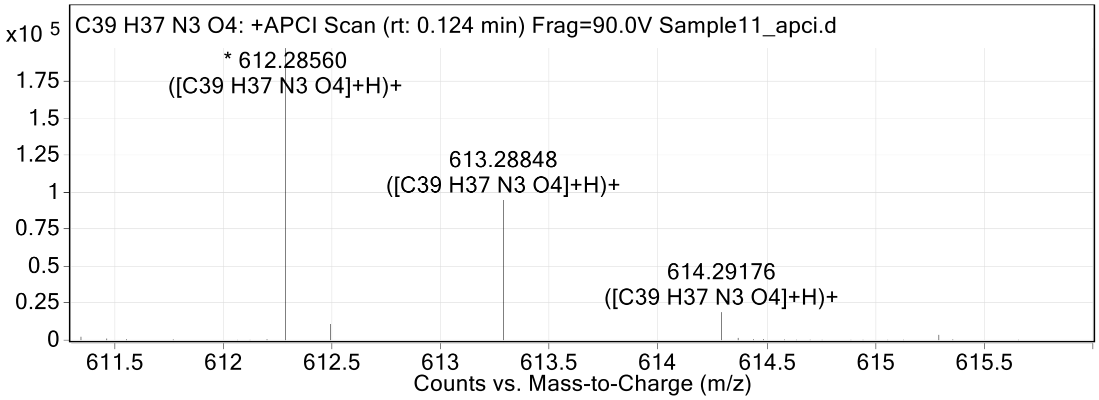

HRMS spectrum of 6aa

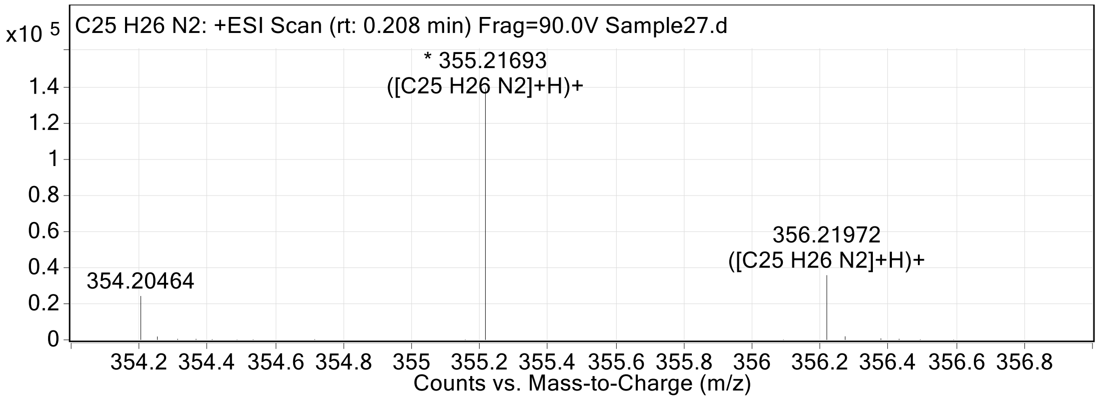

HRMS spectrum of 6ab

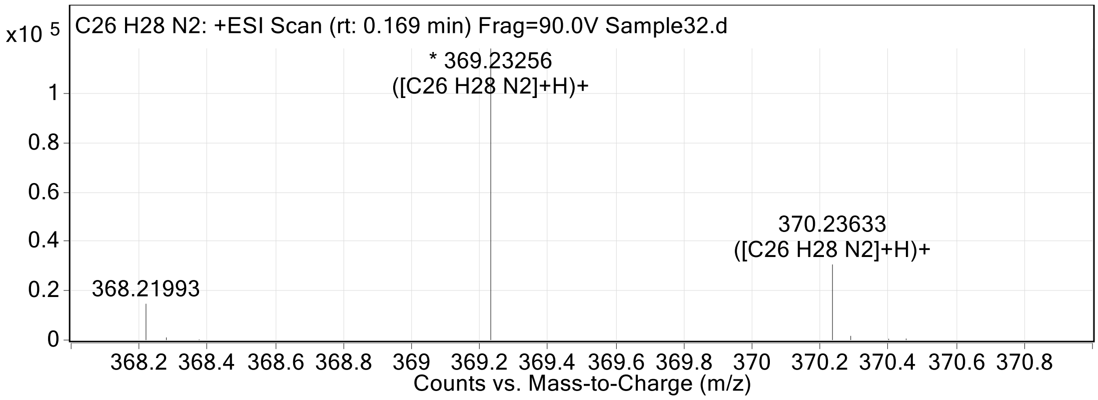

HRMS spectrum of 6ac

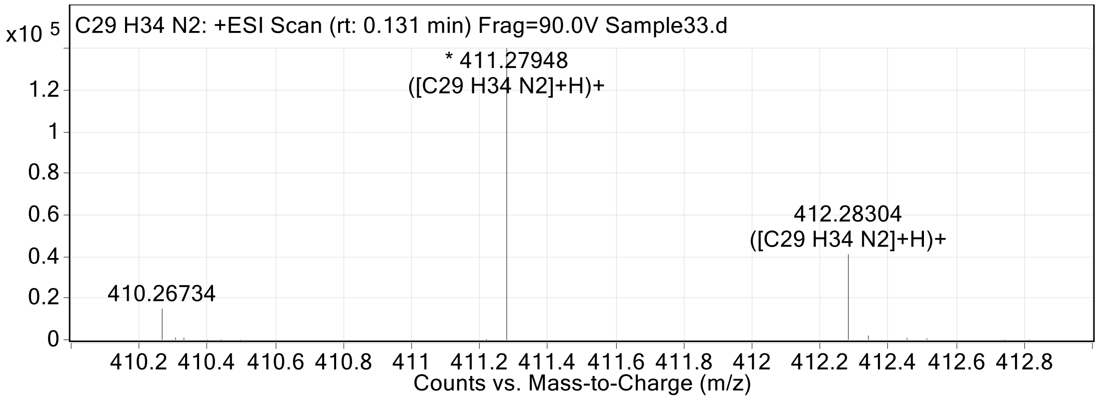

HRMS spectrum of 6ad

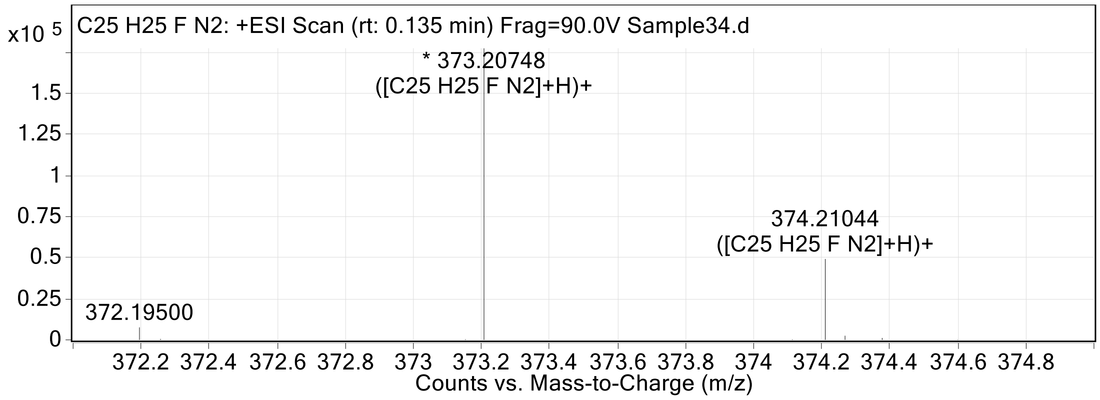

HRMS spectrum of 6ae

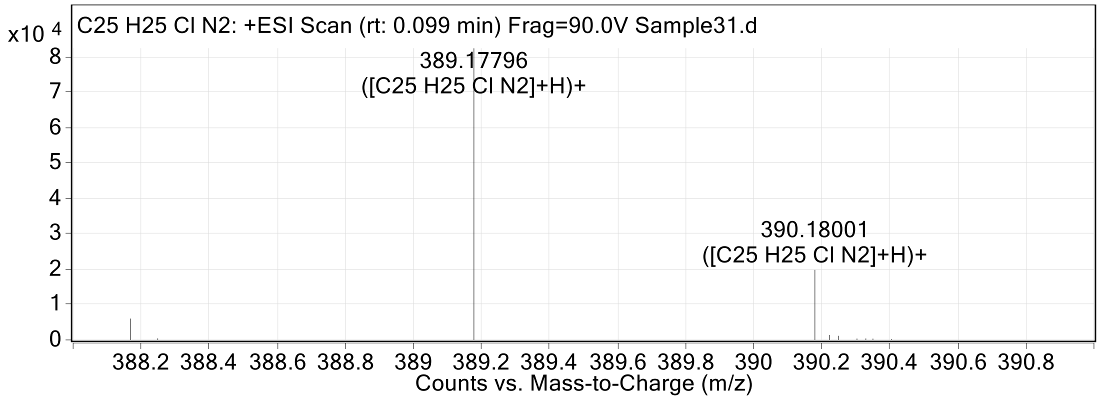

HRMS spectrum of 6af

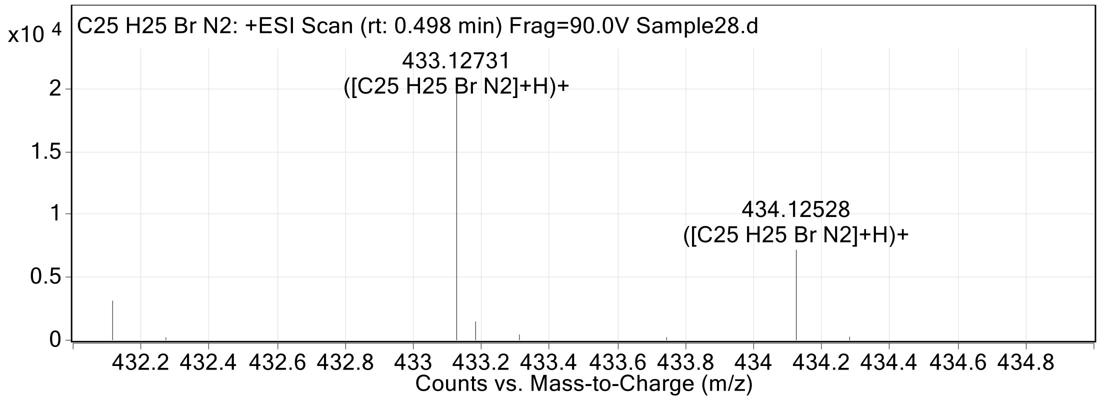

HRMS spectrum of 6ag

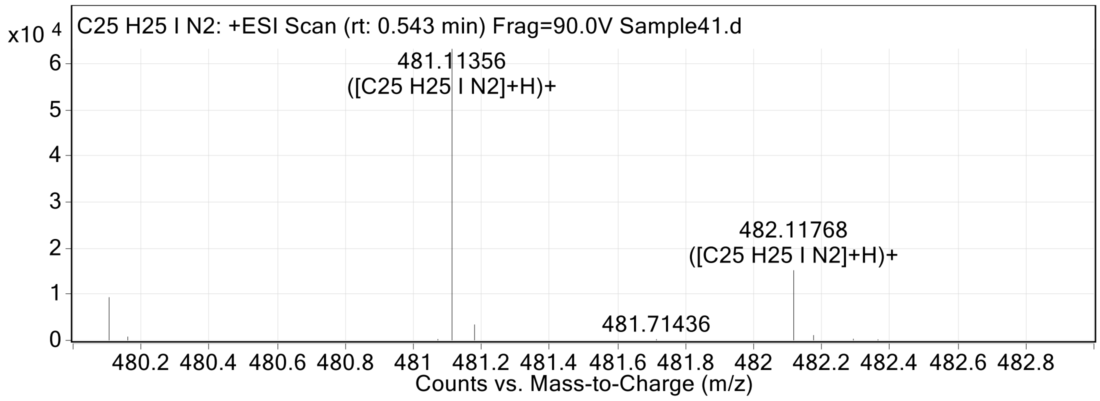

HRMS spectrum of 6ah

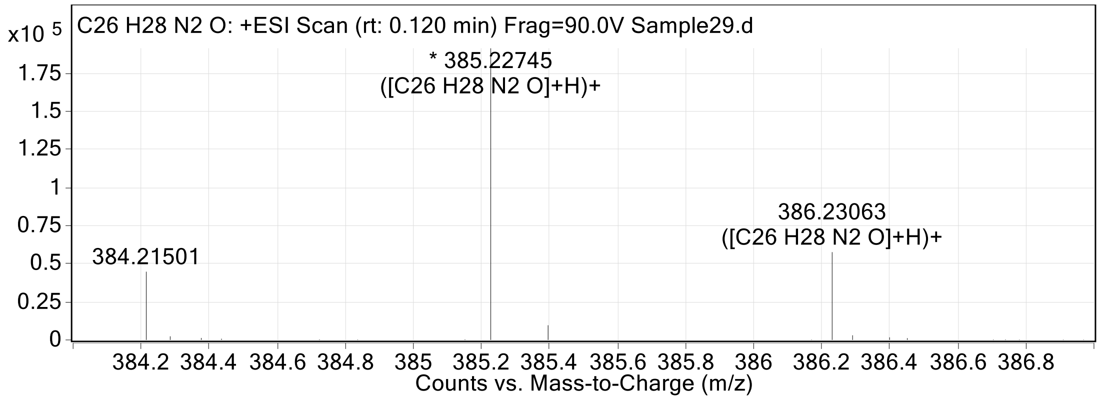

HRMS spectrum of 6aj

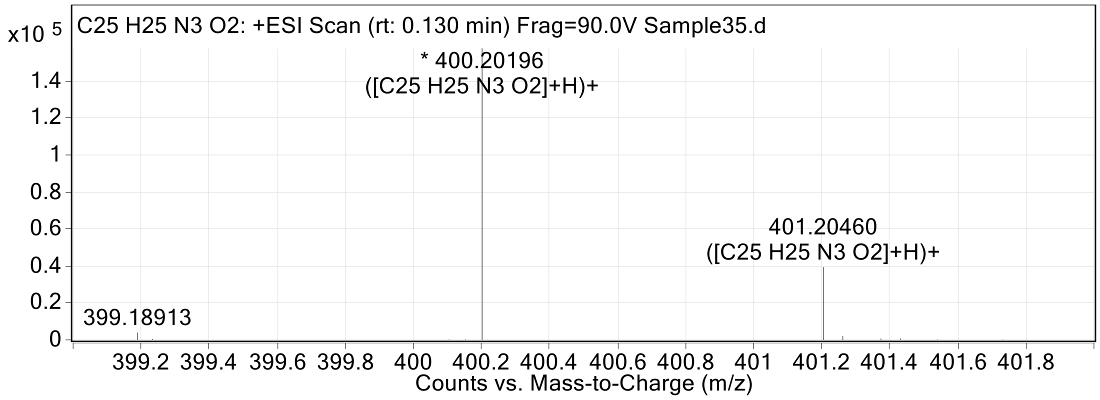

HRMS spectrum of 6ak

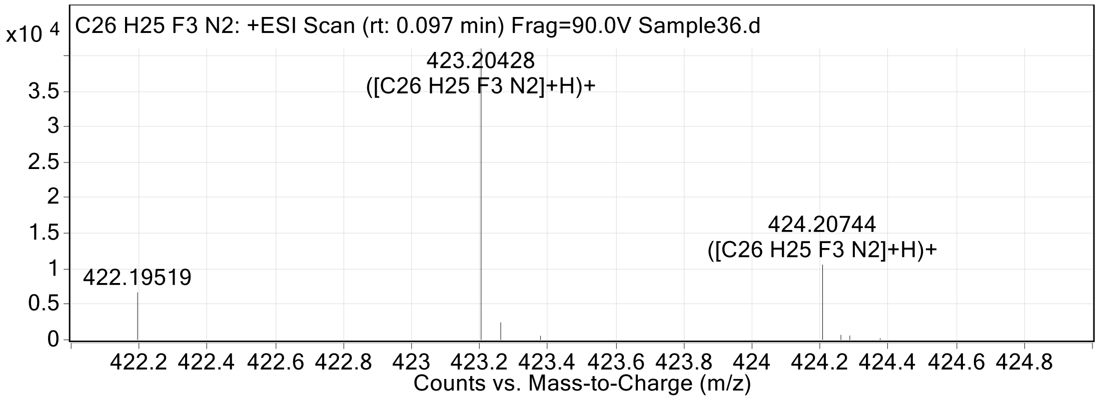

HRMS spectrum of 6al

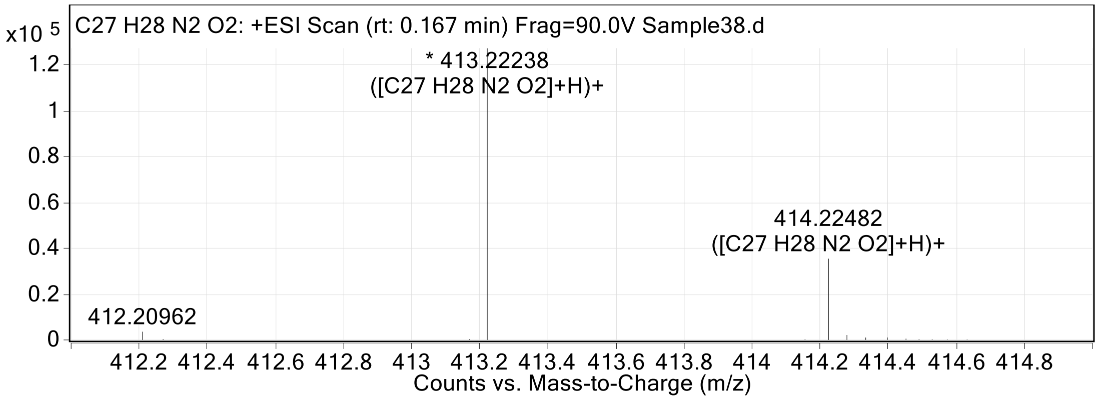

HRMS spectrum of 6am

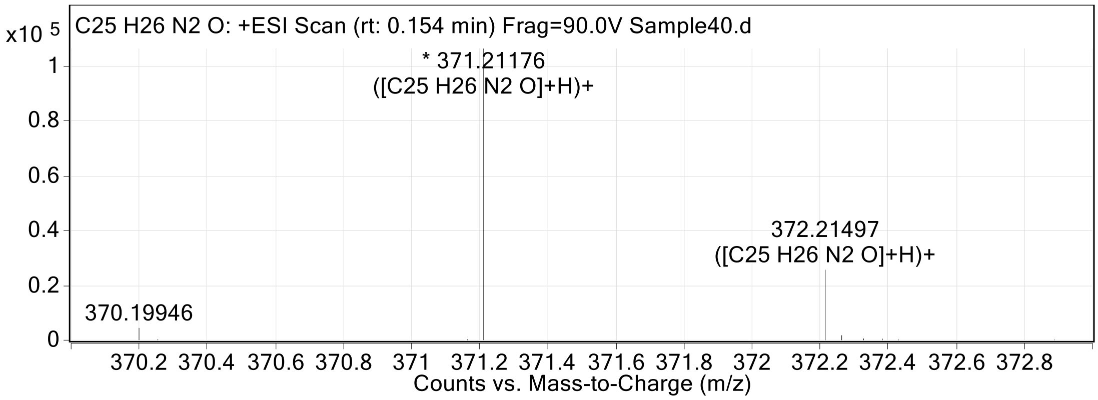

HRMS spectrum of 6an

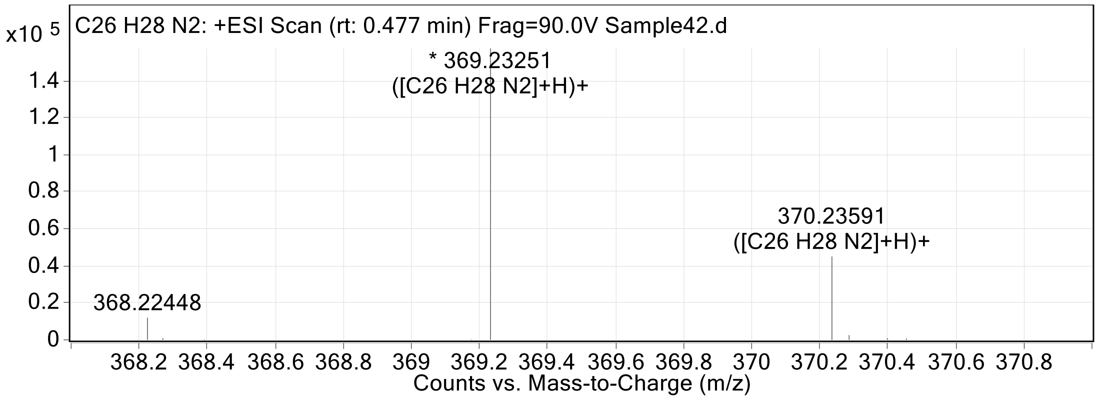

HRMS spectrum of 6ao

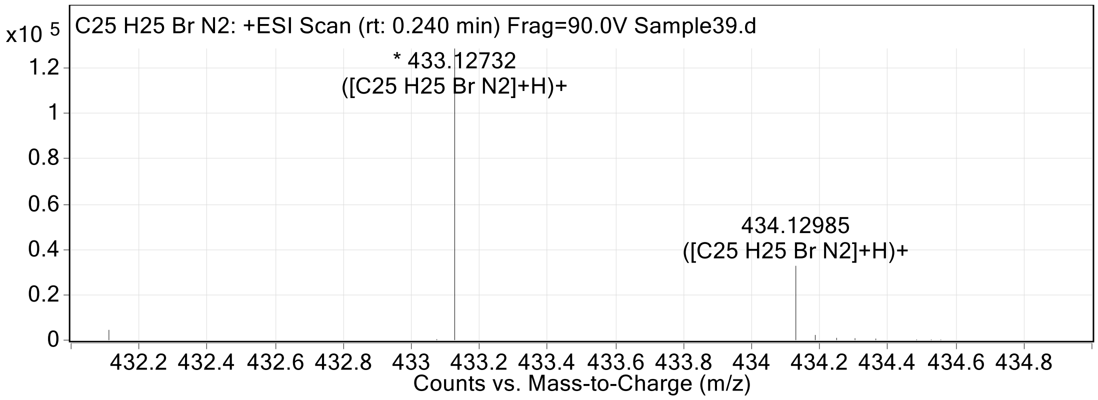

HRMS spectrum of 6ap

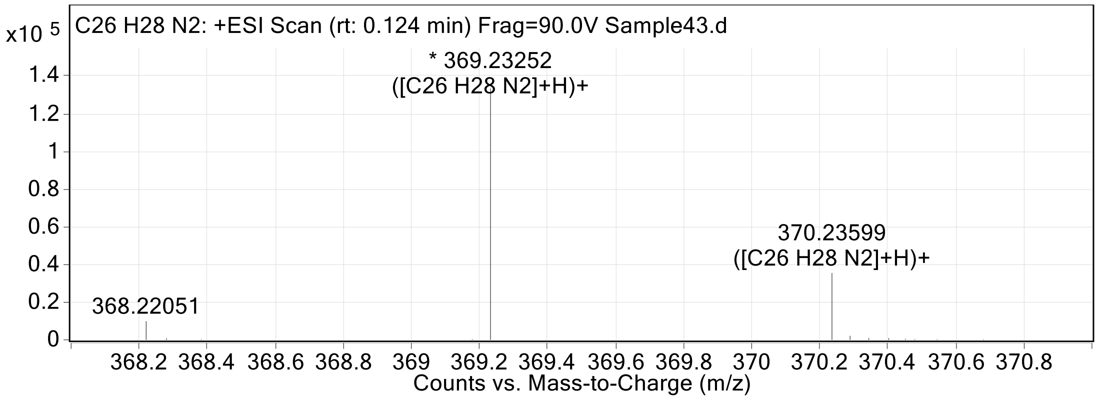

HRMS spectrum of 6aq

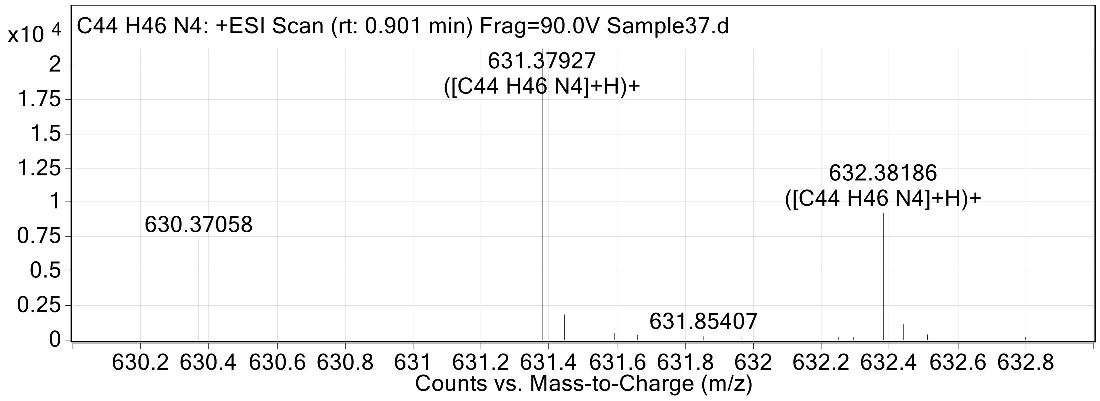

HRMS spectrum of 6ar

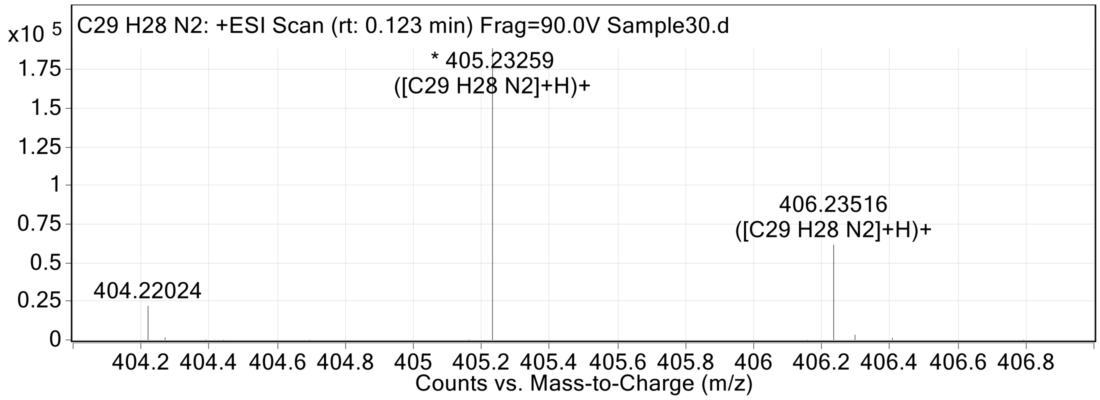

HRMS spectrum of 6au$_2$

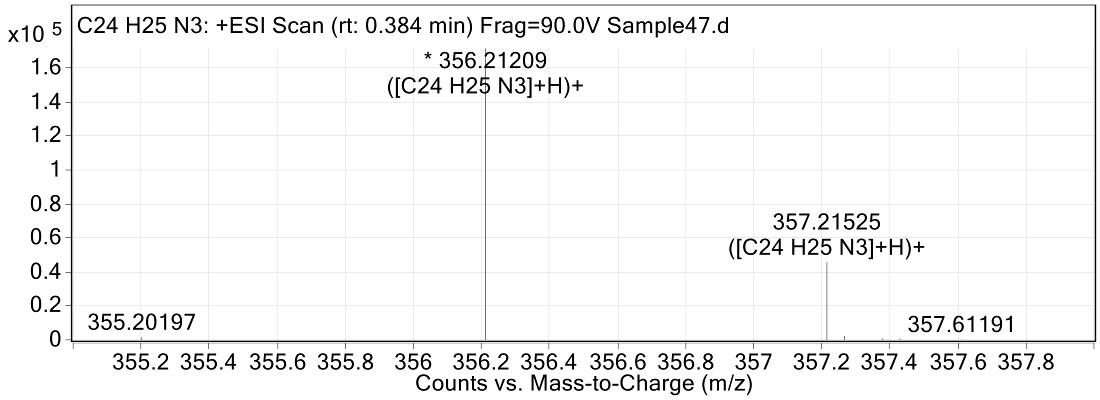

HRMS spectrum of 6au$_3$

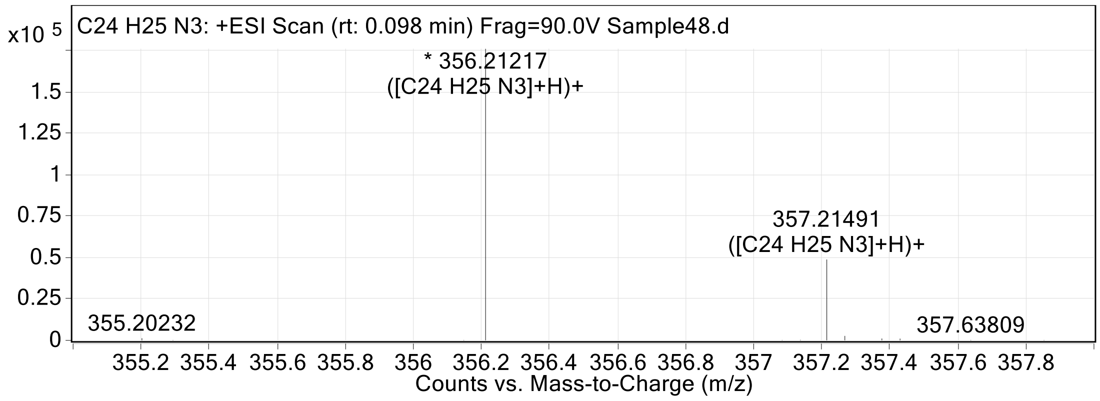

HRMS spectrum of 6au$_4$

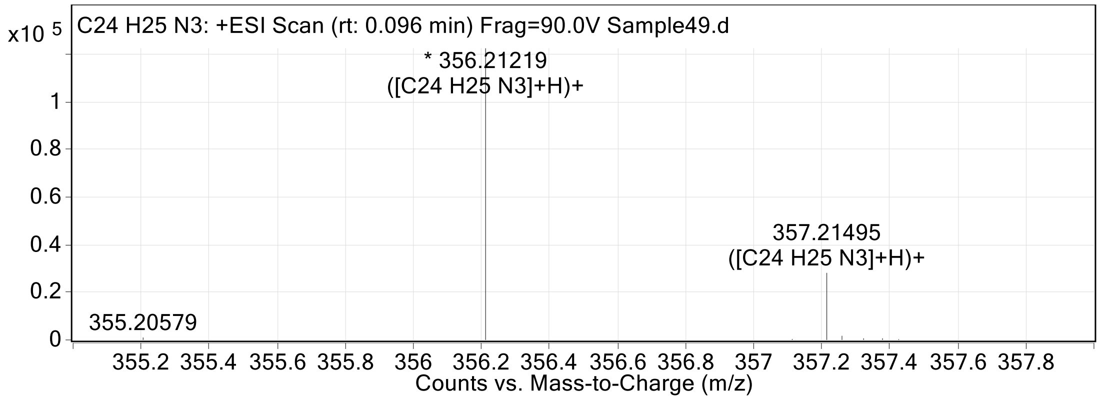

HRMS spectrum of 6av$_2$

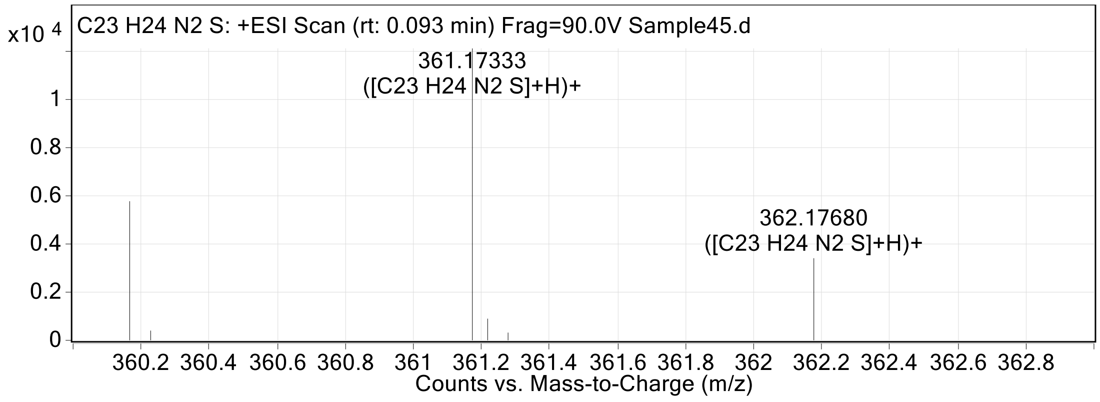

HRMS spectrum of 6aw$_2$

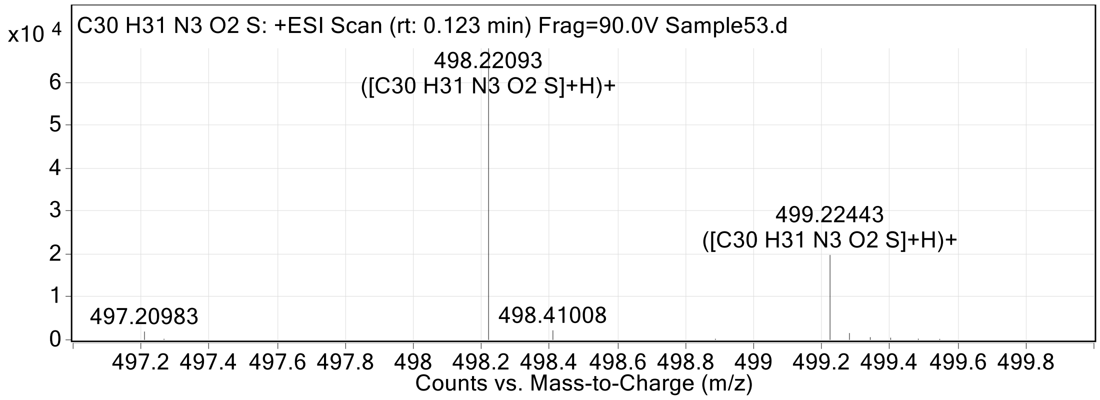

HRMS spectrum of 6ay$_3$

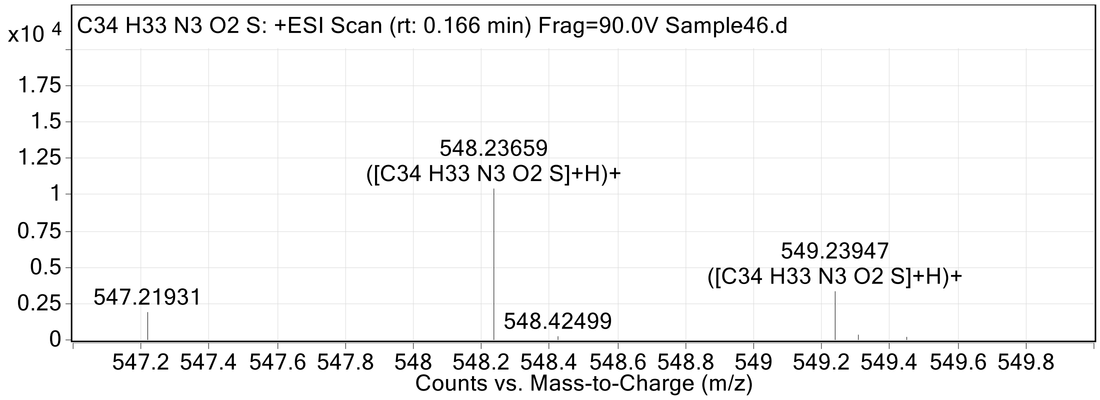

HRMS spectrum of 6ay$_4$

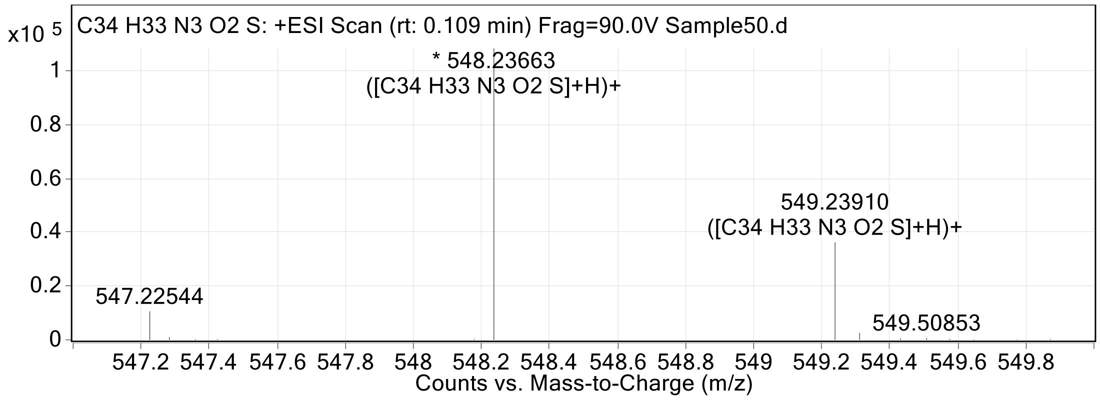

HRMS spectrum of 6ay$_5$

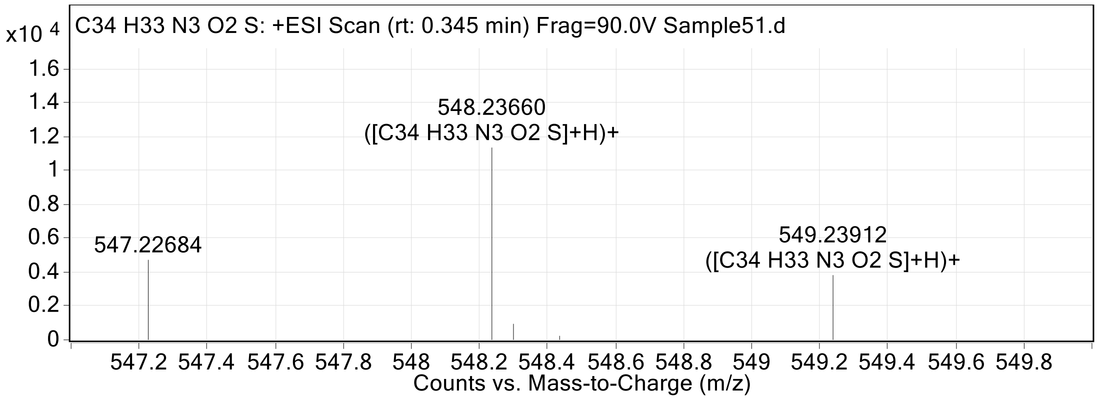

HRMS spectrum of 6ay$_6$

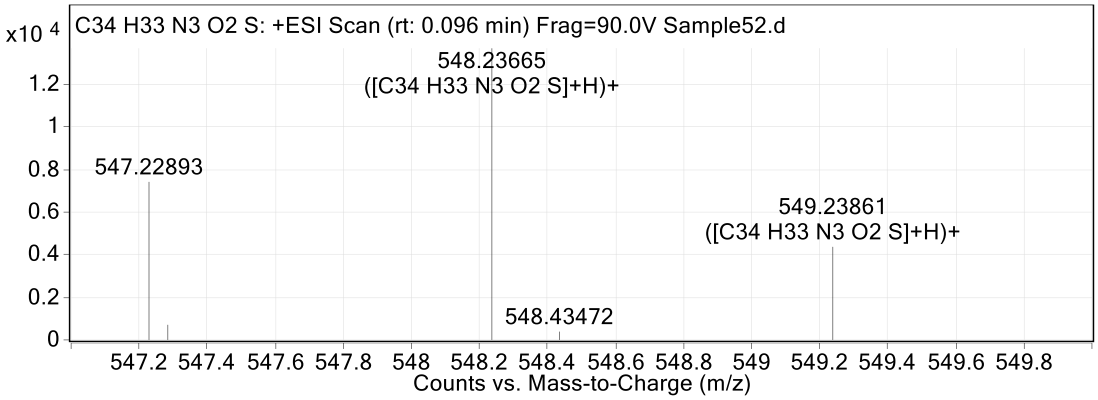

HRMS spectrum of 6bj

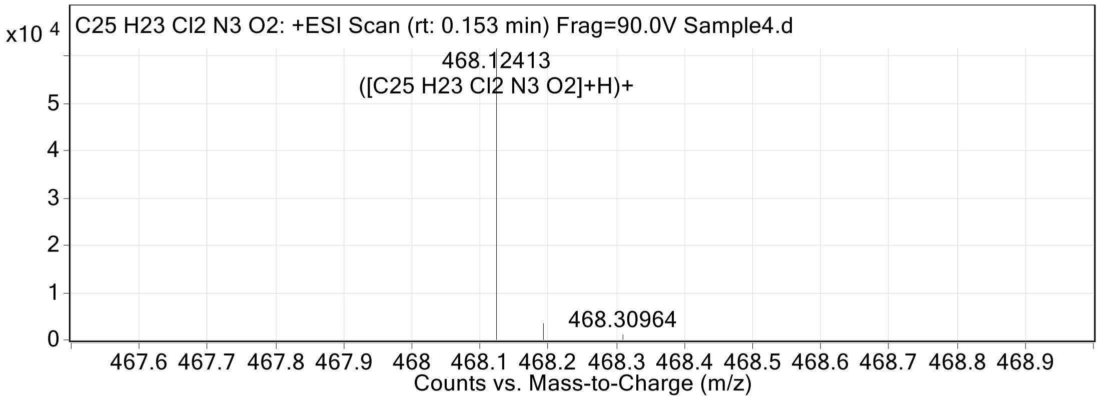

HRMS spectrum of 6cj

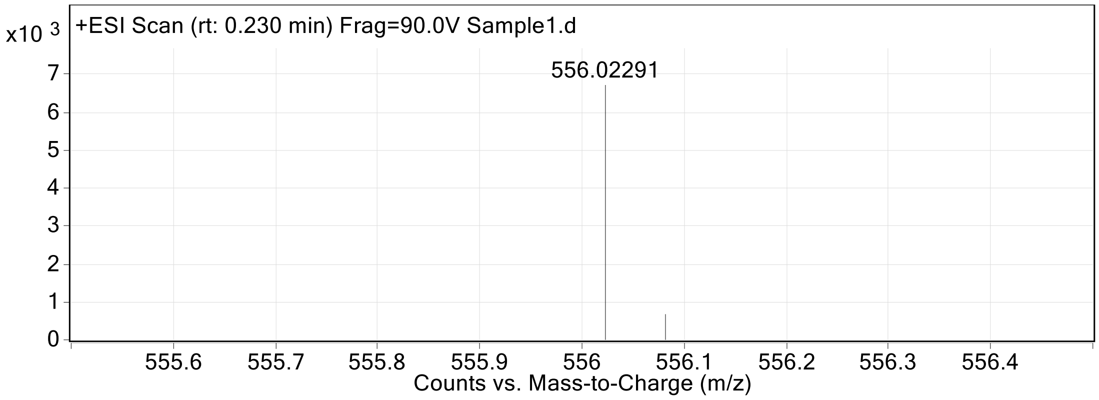

HRMS spectrum of 6ej

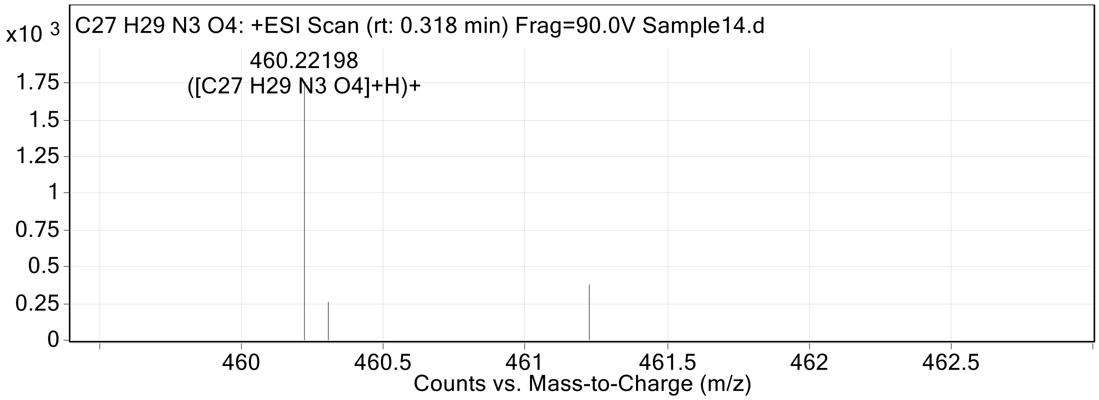

HRMS spectrum of 6gj

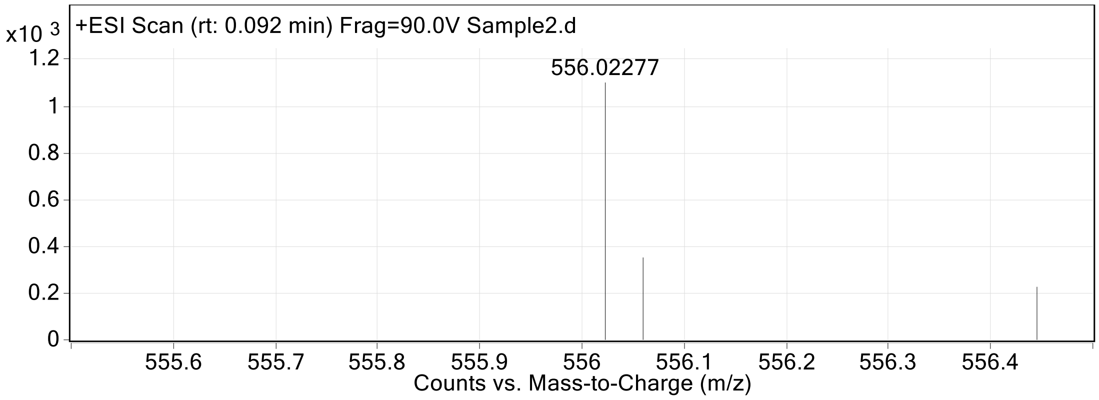

HRMS spectrum of 6oj

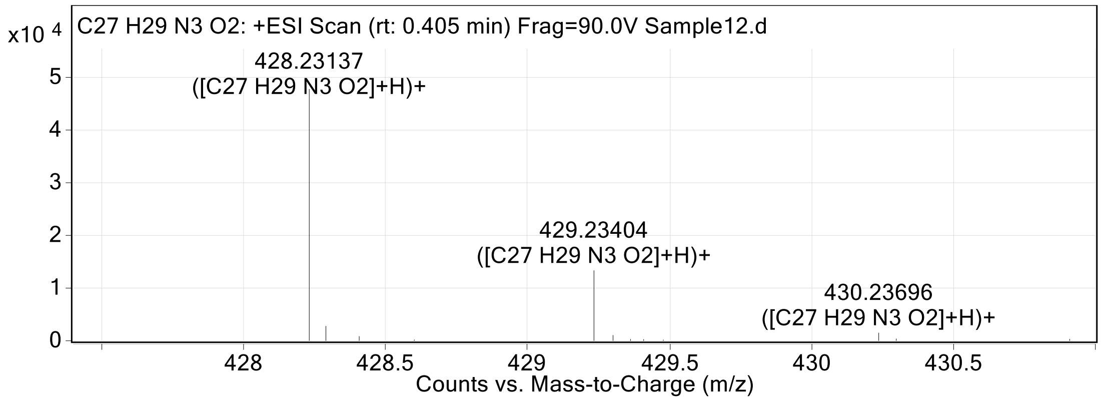

HRMS spectrum of 13e

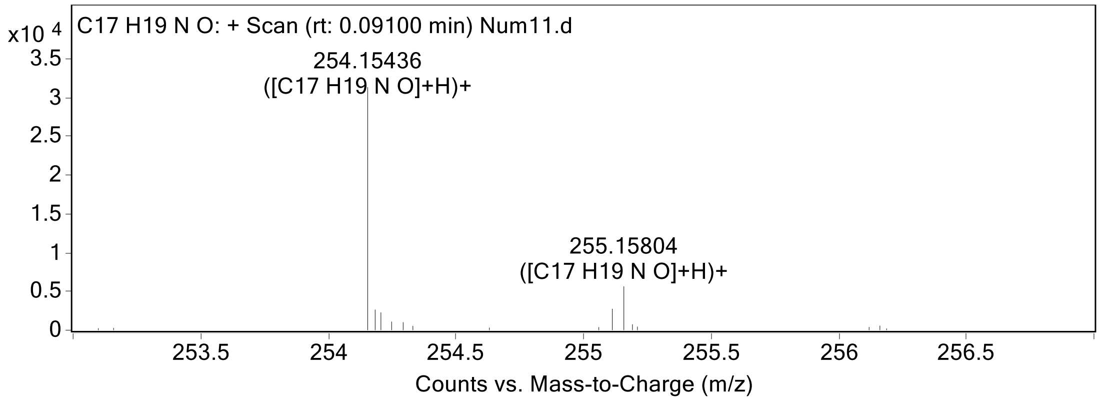

HRMS spectrum of 13i

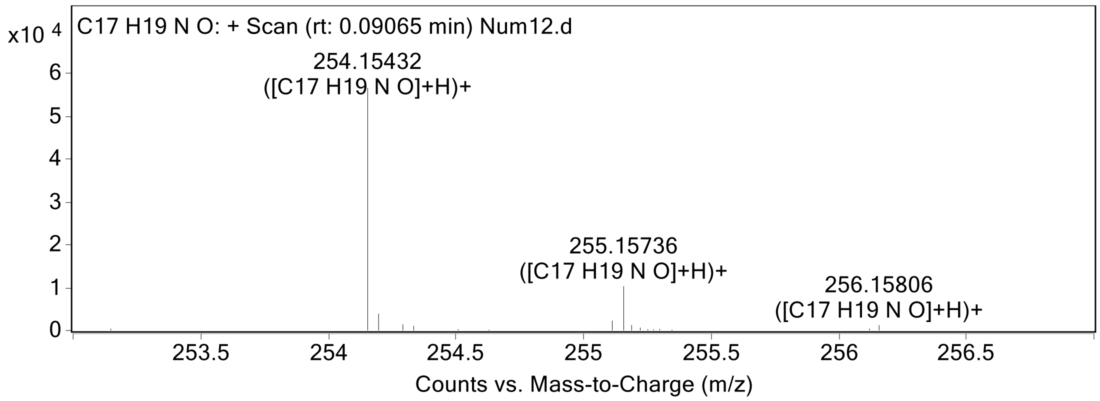

HRMS spectrum of 13k

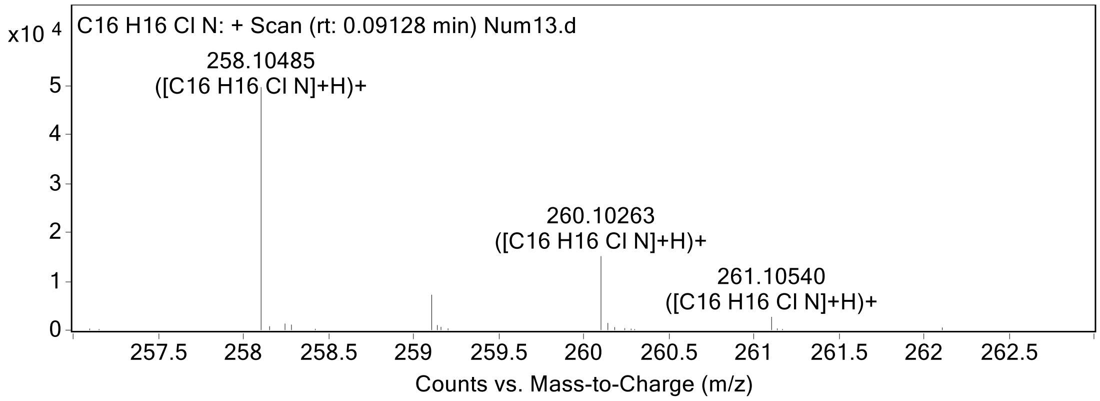

HRMS spectrum of 14aj

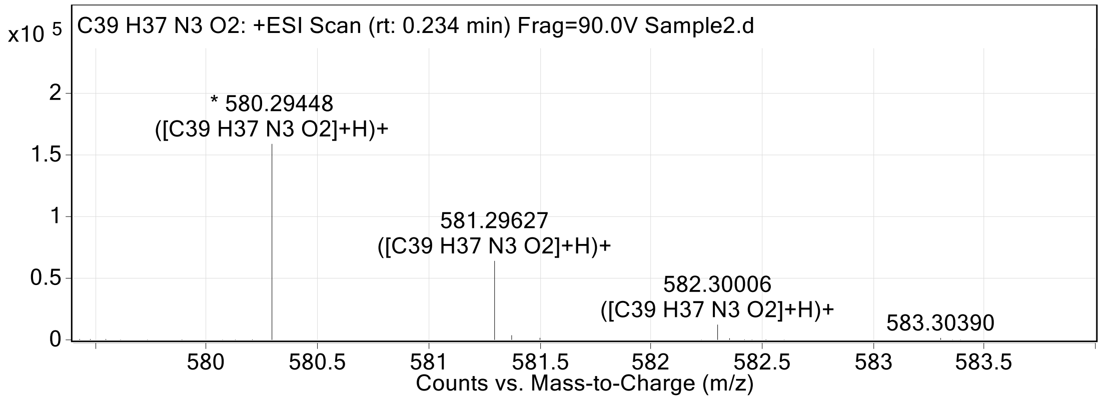

HRMS spectrum of 14bj

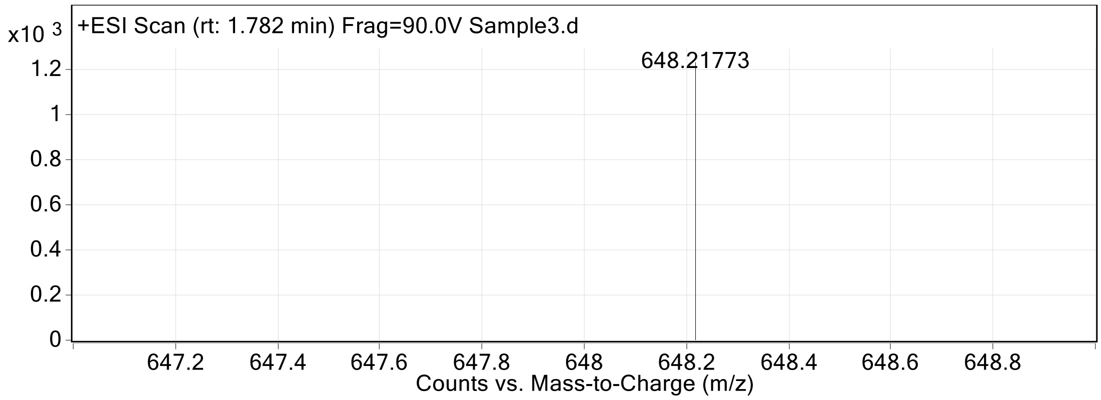

HRMS spectrum of 14cj

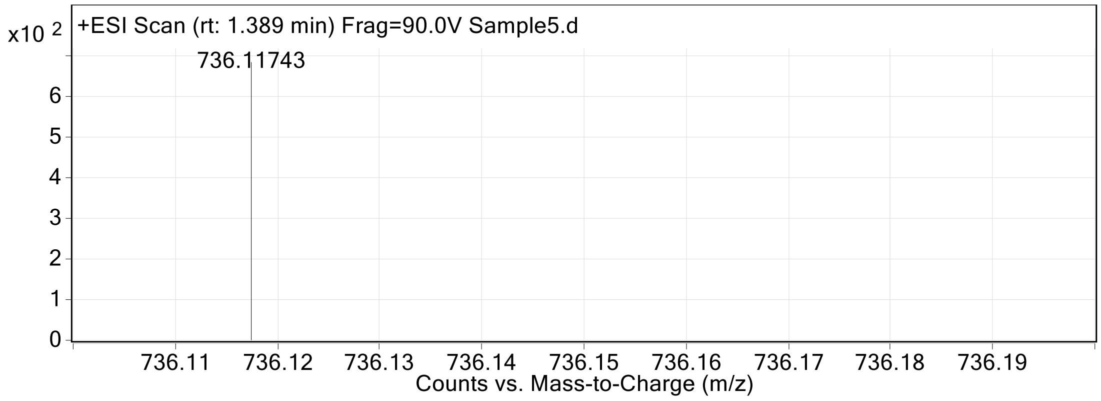

HRMS spectrum of 14ej

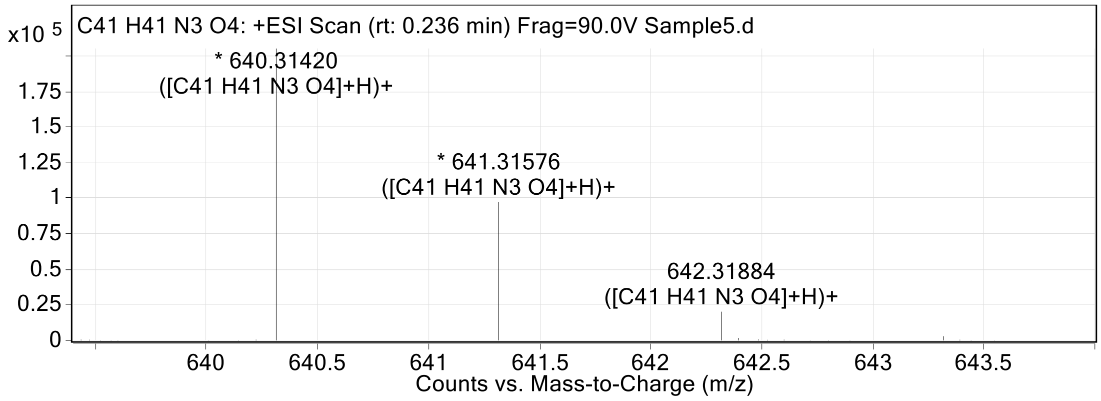

HRMS spectrum of 14fj

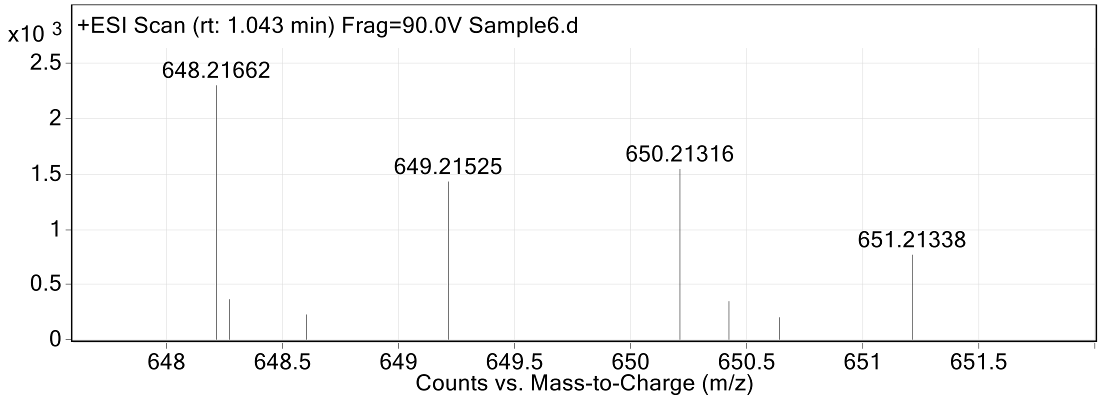

HRMS spectrum of 14hj

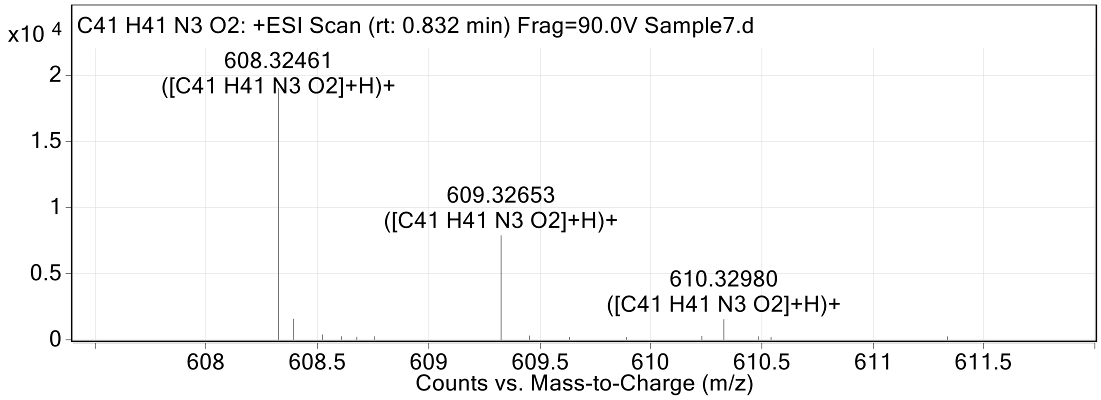

HRMS spectrum of 14ij

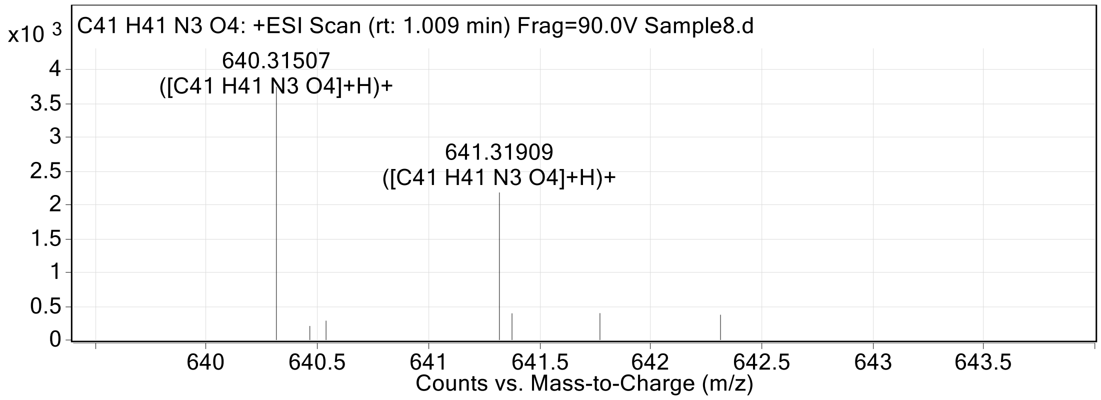

HRMS spectrum of 14mj

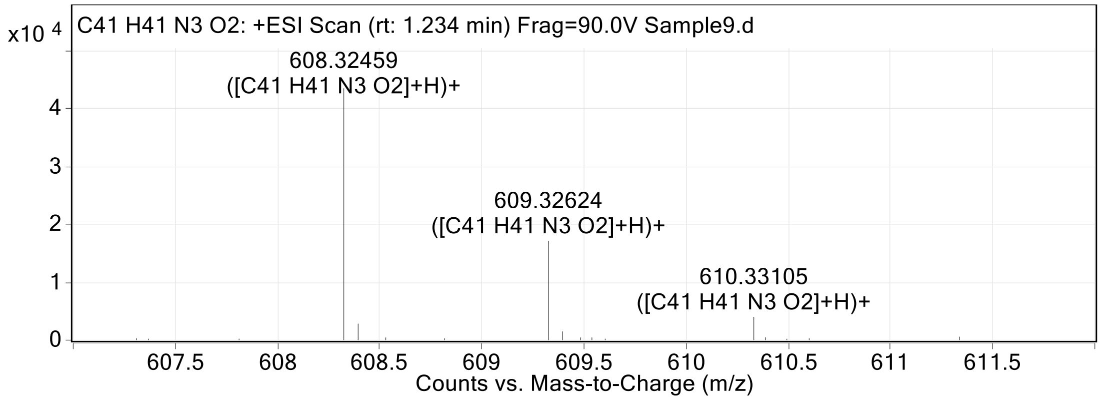

HRMS spectrum of 14nj

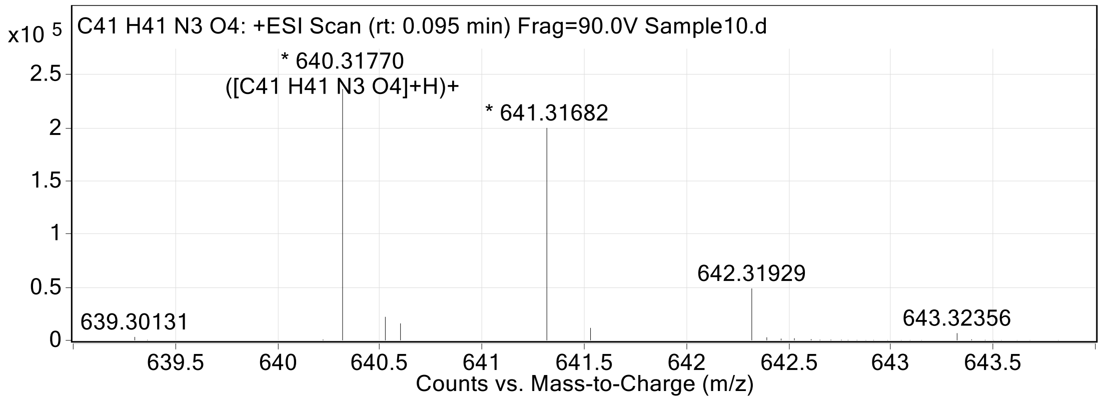

HRMS spectrum of 14oj

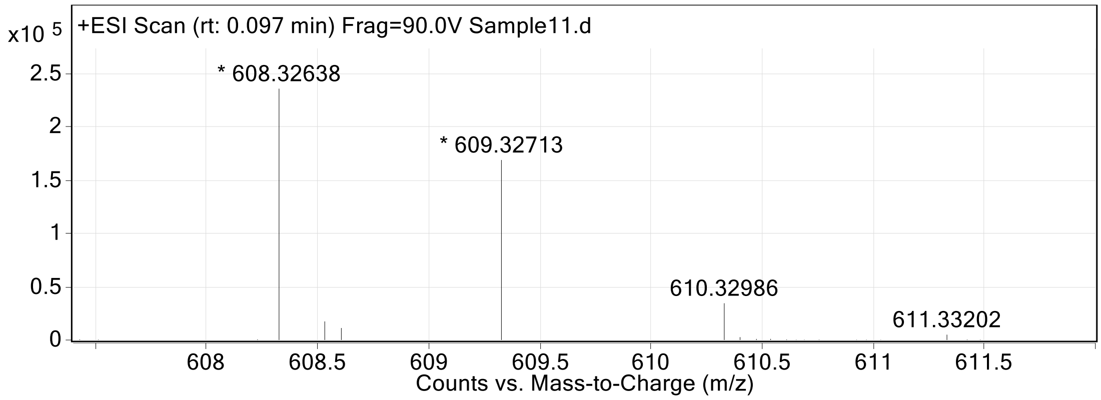

HRMS spectrum of 14pj

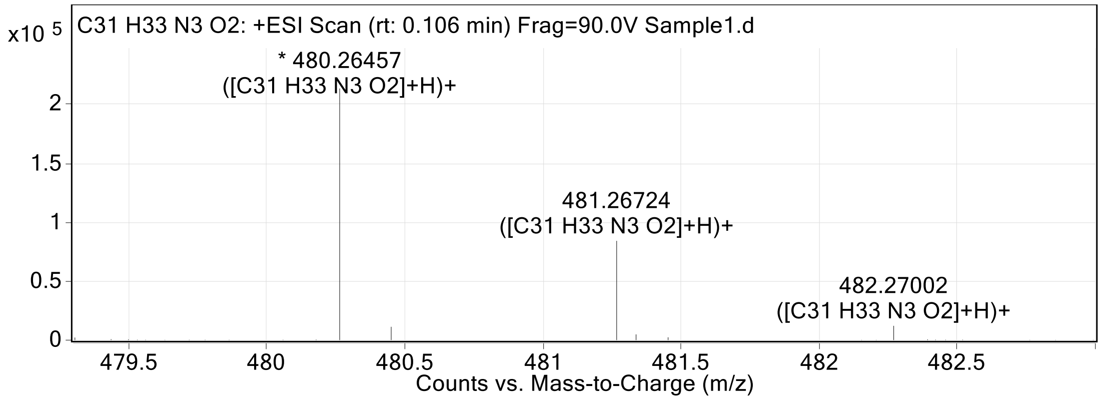

HRMS spectrum of 15gj

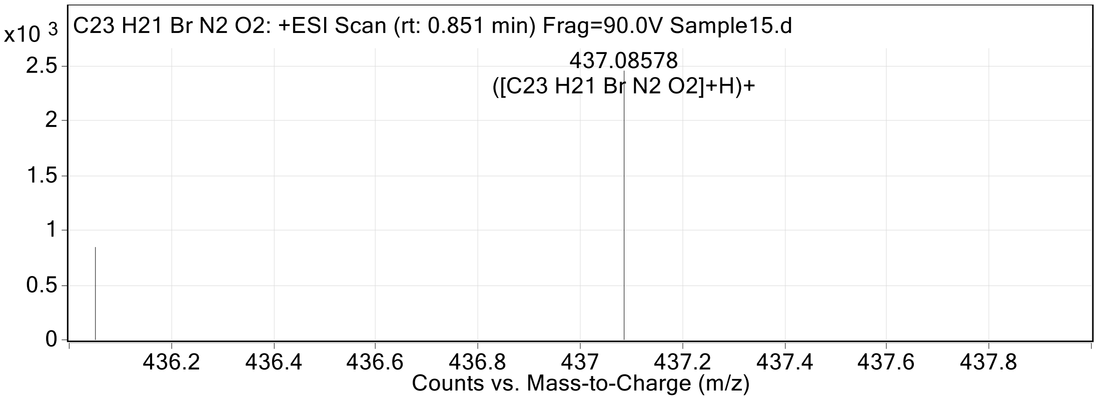

HRMS spectrum of 16af

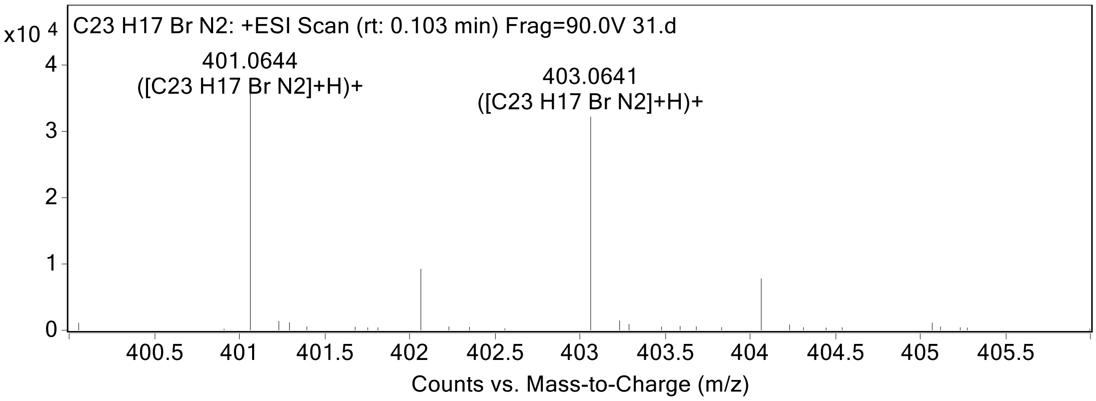

HRMS spectrum of 17af

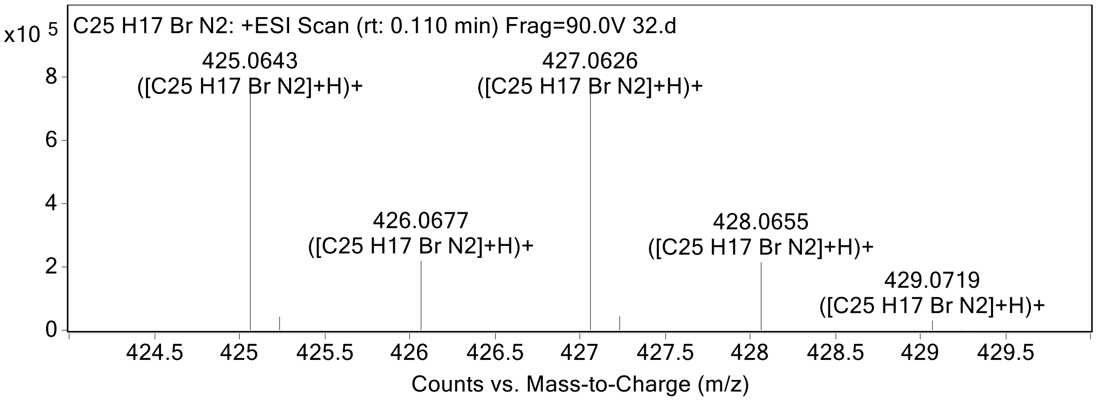

HRMS spectrum of 18

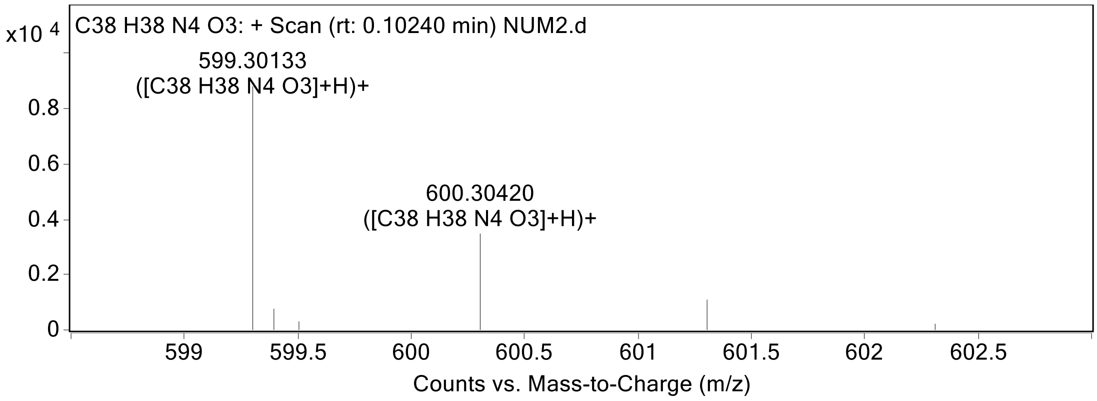

HRMS spectrum of 19

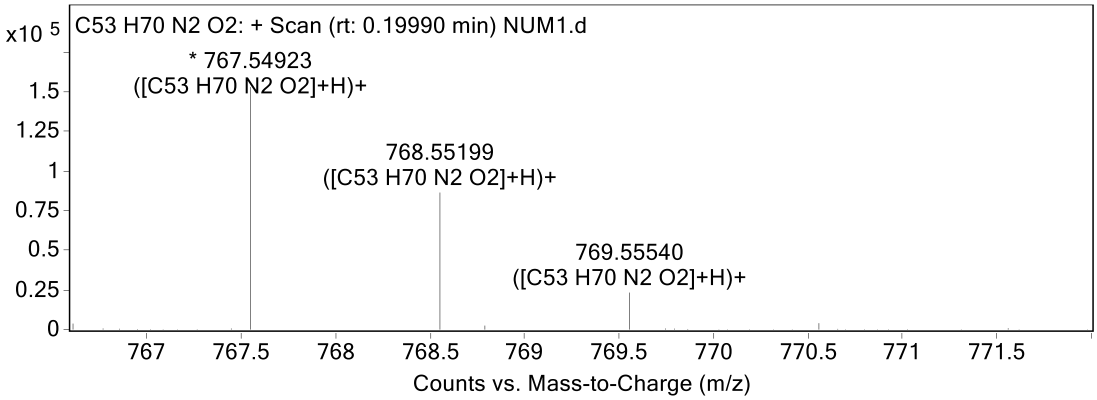

HRMS spectrum of 20

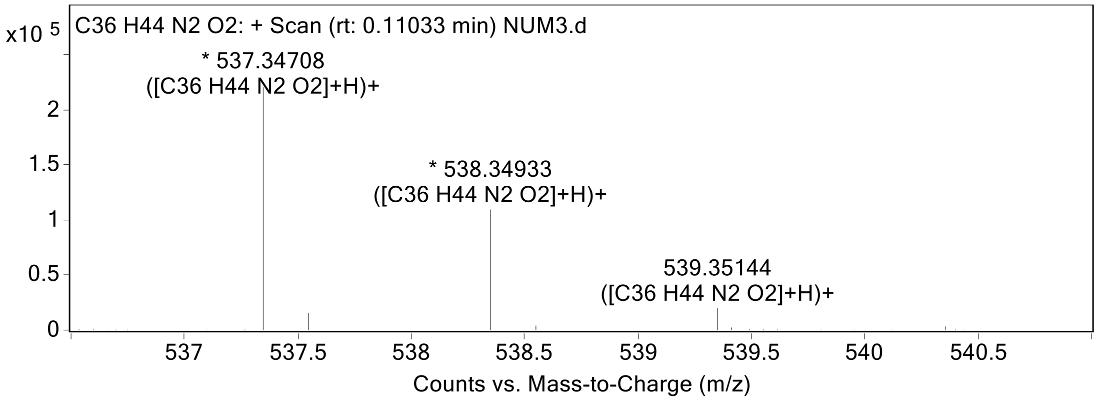

HRMS spectrum of 22

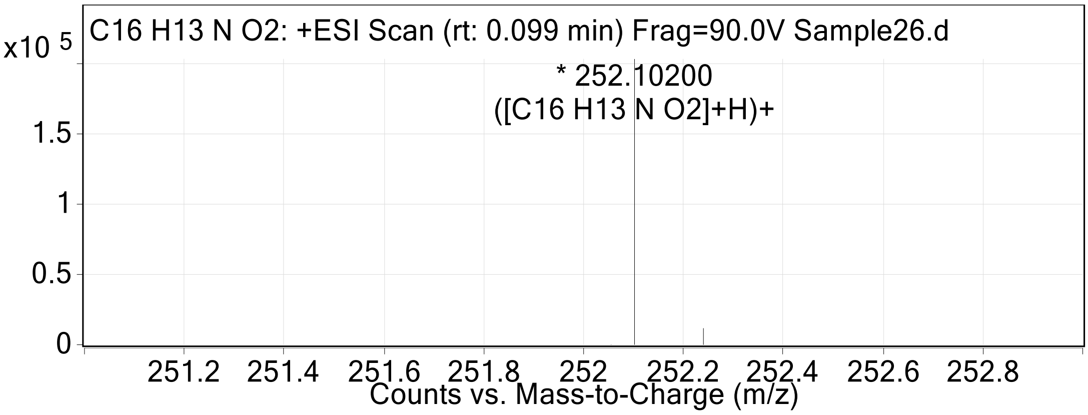

HRMS spectrum of 23

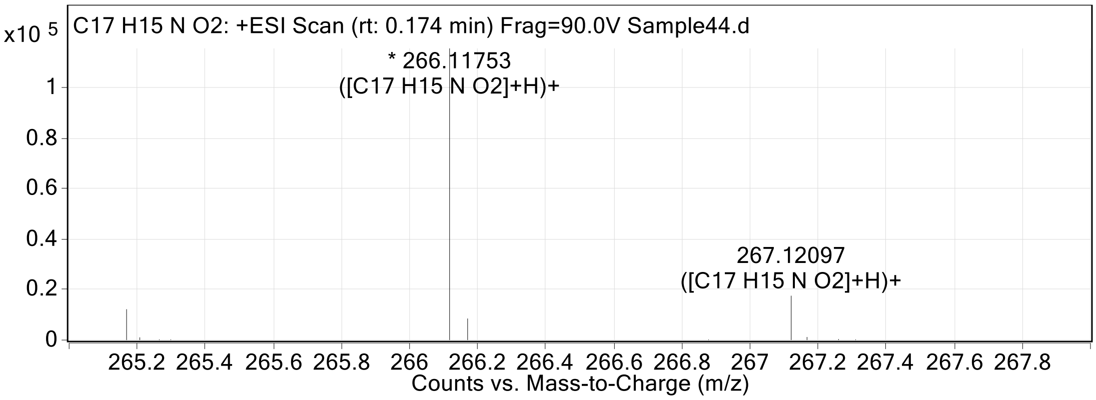

Supplement: Supplementary file 1 [file jo5c00960_si_001.pdf]
